# Silver-catalyzed site-selective C(sp^3^)−H benzylation of ethers with *N*-triftosylhydrazones

**DOI:** 10.1038/s41467-022-29323-3

**Published:** 2022-03-30

**Authors:** Zhaohong Liu, Hongwei Wang, Paramasivam Sivaguru, Steven P. Nolan, Qingmin Song, Weijie Yu, Xinyu Jiang, Edward A. Anderson, Xihe Bi

**Affiliations:** 1 Department of Chemistry, Northeast Normal University, Changchun, 130024 China; 2 Department of Chemistry and Sustainable Chemistry, Ghent University, 281 Krijgslaan S-3, 9000 Ghent, Belgium; 3 Chemistry Research Laboratory, University of Oxford, 12 Mansfield Road, OX1 3TA Oxford, UK; 4 State Key Laboratory of Elemento-Organic Chemistry, Nankai University, Tianjin, 300071 China

**Keywords:** Chemical synthesis, Homogeneous catalysis, Synthetic chemistry methodology

## Abstract

The insertion of carbenes into the α-C–H bonds of ethers represents one of the most powerful approaches to access polysubstituted α-branched ethers. However, intermolecular carbene insertions remain challenging, since current approaches are generally limited to the use of toxic and potentially explosive α-diazocarbonyl compounds. We now report a silver-catalyzed α-C–H benzylation of ethers using bench-stable *N*-triftosylhydrazones as safe and convenient carbene precursors. This approach is well suited for both inter- and intramolecular insertions to deliver medicinally relevant homobenzylic ethers and 5–8-membered oxacycles in good yields. The synthetic utility of this strategy is demonstrated by its easy scalability, broad scope with valuable functional groups, high regioselectivity, and late-stage functionalization of complex oxygen-containing molecules. The relative reactivities of different types of silver carbenes and C−H bonds were also investigated by experments and DFT calculations.

## Abstract (web summary)

A state-of-the-art tool in synthetic organic chemistry is a rhodium diazocarbene species, which despite its flexibility, is derived from expensive and highly reactive species. Here the authors present a methodology for C–H benzylation of ethers via an analogous silver carbene species, obtained from a more common metal and a more stable starting material.

## Introduction

**Fig. 1:**
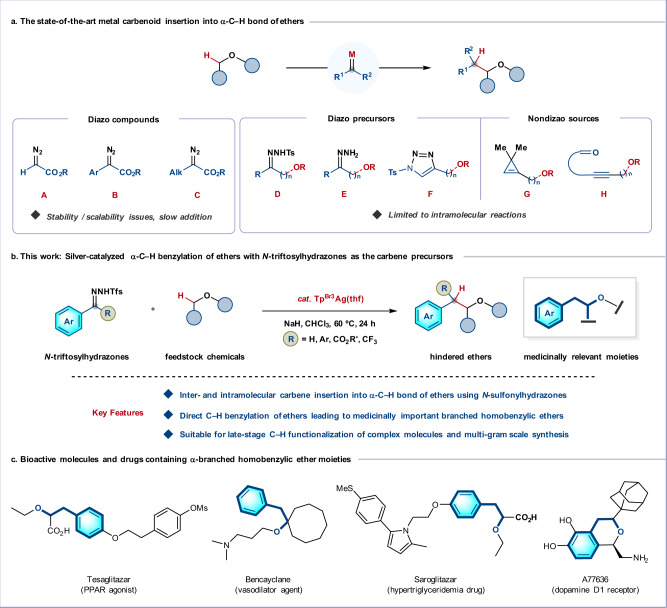
Inspirations towards the development of carbene insertion into α-C–H bonds of ethers using *N*-sulfonylhydrazones. **a** α-C−H functionalization of ethers via carbene insertion process: drawbacks and solutions. **b** This work: Silver-catalyzed α-C–H insertion reactions of ethers with *N*-triftosylhydrazones for the synthesis of medicinally relevant branched homobenzylic ethers. **c** Representative drugs demonstrating the ubiquity of branched homobenzylic ether motifs in bioactive molecules. Ar, aryl; Alk, alkyl; Tfs, triftosyl; thf, tetrahydrofuran; Ms, methylsulfonyl.

Ethers are abundant and low-cost feedstocks for chemical synthesis^1–4^. The direct catalytic α-C–H functionalization of ethers has drawn increasing attention,^5–12^ since the installation of substituents at the α-position can significantly increase the potency of bioactive molecules^13,14^. Among such strategies, the insertion of carbenes into the α-C–H bonds of ethers has become a powerful tool for the selective creation of carbon–carbon bonds, delivering otherwise inaccessible branched polysubstituted ethers (Fig. 1a)^15–19^. Since the pioneering work of Adams and co-workers in 1989^20^, this methodology has been well-established^21–34^; in particular, Davies, Pérez, Hartwig, and Arnold groups have made significant developments with donor/acceptor^26–31^ and acceptor carbenes^21–23,32–34^. Such insertion chemistry has also been successfully applied to the synthesis of complex natural products^18,19^. Nevertheless, the use of toxic and potentially explosive diazo compounds is a significant drawback, which indeed in part explains the typical need for an acceptor group in the carbene precursor. These potentially unstable diazo compounds are often prepared directly before use, requiring controlled addition using a syringe pump, which presents a further obstacle to large-scale applications^31^. This inherent challenge has been partially addressed by the in situ generations of energetic diazo compounds using the continuous flow technique^31^.

An appealing solution to this problem would be to replace the diazo compound with a safe, stable carbene precursor^35^. In this context, Che^36,37^ and Zhang^38^ groups developed *N*-tosylhydrazones (**D**) and exploited them as donor/donor carbene precursors in intramolecular carbene C–H insertions leading to substituted tetrahydrofurans, whereas the Sarpong^39^ group used *N*-sulfonyltriazoles (**F**). Donor/donor carbenes can also be generated in situ by the oxidation of hydrazones (**E**)^40,41^, the ring-opening of 3,3-dimethylcyclopropenes (**G**)^42^, or the cyclization of enynones (**H**)^43^. Although these donor carbene precursors are useful, these methods have been limited to intramolecular reactions and suffer from the need for multiple steps to obtain the starting materials. Intermolecular insertions into ether α-C–H bonds are highly attractive in avoiding this need for multistep substrate synthesis, but to date have typically required the use of α-diazocarbonyl compounds as mentioned above (**A**–**C**, Fig. 1a), and the scope of ethers is largely restricted to strained cyclic ethers^21,23–26^, or activated benzyl and allyl ethers^28,29,33,34^.

Here we report a silver-catalyzed carbene insertion into ether α-C–H bonds with readily available *N*-triftosylhydrazones^44–48^ as donor carbene precursors (Fig. 1b). Hydrazone decomposition proceeds under mild conditions and the resulting silver carbenes undergo high-yielding, selective insertions into C(sp^3^)−H bonds adjacent to ether oxygen atoms in both inter- and intramolecular ways. This operationally simple procedure converts simple ether feedstocks into high-value branched homobenzylic ethers. Such hindered ethers are frequently encountered motifs in bioactive molecules and drugs (Fig. 1c)^13,14^_,_ whereas they are difficult to access through conventional S_N_2 and carbocation-based approaches due to the competing elimination^49^.

## Results and discussion

### Reaction development

**Fig. 2:**
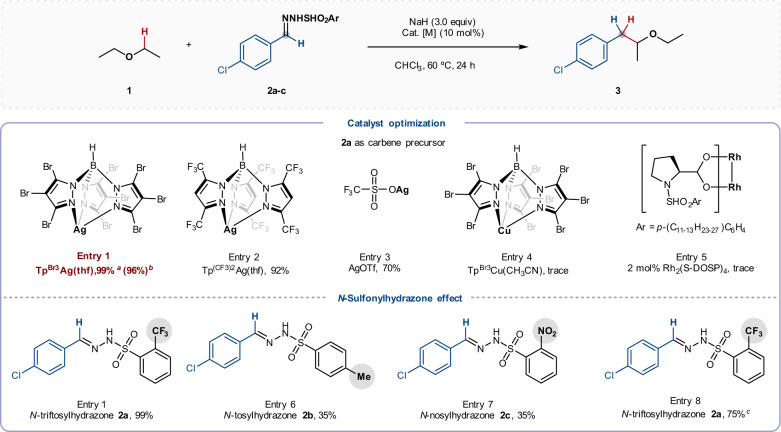
Selected optimization of reaction conditions. Reaction conditions: *N*-Sulfonylhydrazone **1a**–**c** (0.3 mmol), NaH (0.9 mmol, 3.0 equiv), catalyst (10 mol%), Et_2_O (1.0 mL), and CHCl_3_ (5.0 mL), under argon at 60 °C for 24 h unless otherwise indicated. ^*a*^ Yields were determined by ^1^H NMR spectroscopy with dibromomethane as an internal standard. ^*b*^ Isolated yield is given in parentheses. ^*c*^ Et_2_O (2.0 equiv) was used.

Our investigations began with the reaction of diethyl ether **1** and *N*-triftosylhydrazone **2a** with various catalysts capable of promoting carbene insertions (Fig. 2; for full details see Supplementary Table 1). We were pleased to find that use of 5 mol% Tp^Br3^Ag(thf) as the catalyst, with NaH as the base in Et_2_O/CHCl_3_ (1:5) at 60 °C, afforded the corresponding α-C–H insertion product **3** in 96% isolated yield (entry 1, Fig. 2). The related silver congeners, Tp^(CF3)2^Ag(thf) and AgOTf gave slightly inferior results (entries 2 and 3); other catalysts typically used for ether C–H bond functionalization, such as Tp^Br3^Cu(CH_3_CN) (with ethyl diazoacetate)^22^ and Rh_2_(*S*-DOSP)_4_ (with aryl diazo esters)^26^, proved ineffective (entries 4 and 5). The choice of diazo surrogate had a remarkable impact on reaction efficiency, in that much lower yields were obtained with *N*-tosylhydrazone **2b** and *N*‑nosylhydrazone **2c** compared to *N*-triftosylhydrazone **2a** (entries 6 and 7). Pleasingly, we also found that the reaction proceeds well with just 2.0 equiv. of ether, with only a modest reduction in yield (75%, entry 8), demonstrating the potential utility of this strategy for the functionalization of more valuable ethers (e.g., in bioactive compounds) and commodity chemicals. Note that silver catalysts are much less effective than related copper (I) complexes for promoting insertion of acceptor-only carbenes into ether α-C–H bonds^21–23,50^, presumably because of the competition between ethers with ethyl diazoacetate to coordinate to silver center, thus inhibiting the decomposition of the diazo compound to form silver carbene^23^. Nevertheless, Tp^Br3^Ag was found to be most effective in our reactions with donor carbenes, which may be attributed to two factors: (i) the bulky Tp^Br3^ ligand inhibits the carbene dimerization (entries 1 and 3), and (ii) the weak interaction between weakly donating Tp^Br3^ ligand and electron-deficient silver ions makes carbene center more electrophilic, thus favoring the C–H bond insertion (entries 1 and 4)^48,51^.

### Substrate scope

**Fig. 3:**
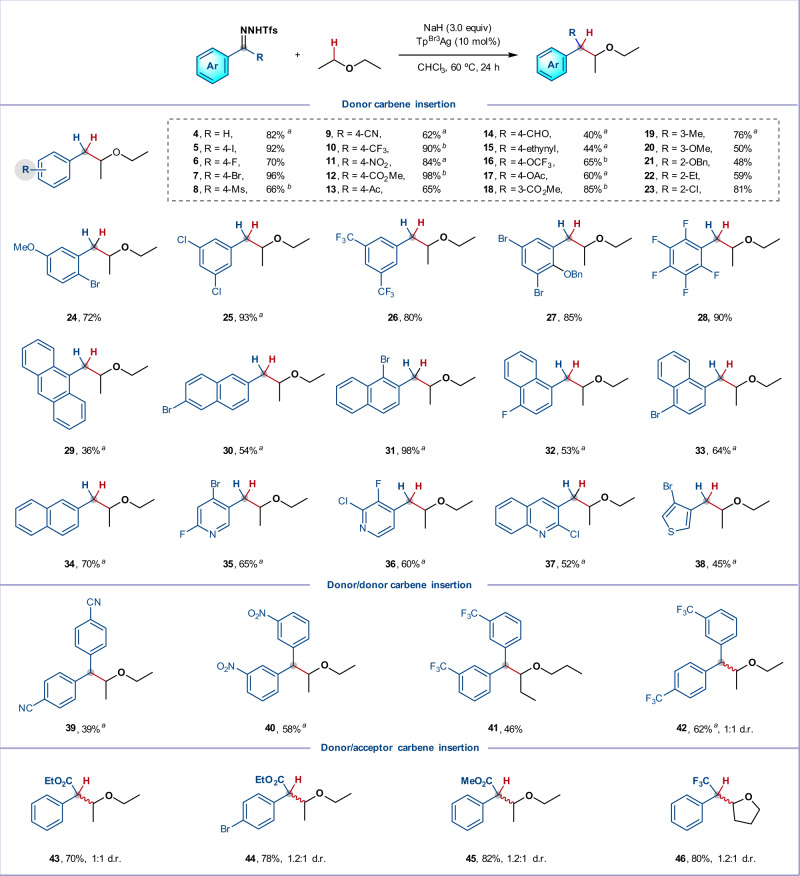
Substrate scope with respective to *N*-triftosylhydrazones. Standard conditions: *N*-Triftosylhydrazone (0.3 mmol), ether (1.0 mL), NaH (0.9 mmol, 3.0 equiv), Tp^Br3^Ag (10 mol%), CHCl_3_ (5.0 mL), under argon atmosphere at 60 °C for 24 h, isolated yield. ^*a*^ Ether (5.0 mL) was used. ^*b*^ PhCF_3_ (5.0 mL) was used. Ms, methylsulfonyl; Bn, benzyl; Ac, acetyl; d.r., diastereomeric ratio.

With a practical protocol for α-C–H insertion in hand, we first examined the structural variation of the *N*-triftosylhydrazone. As presented in Fig. 3, a variety of (hetero)aromatic aldehyde-derived *N*-triftosylhydrazones underwent smooth insertion into the α-C–H bond of diethyl ether, providing products **4**–**38** in good to excellent yields. We explored the influence of substituents at various positions of the aryl ring and found that halides (F, Cl, Br, and I), common electron-withdrawing (Ms, NO_2_, CF_3_, CN, CO_2_Me, OAc, OCF_3_, Ac, CHO, and ethynyl) and electron-donating functional groups (Me, OMe and OBn) were all well-tolerated (**3**–**28**). We found that steric hindrance had minimal effect on the transformation, with *ortho*-, di-, tri-, and poly-substituted *N*-triftosylhydrazones all proceeding well (**21**–**28**). In general, polycyclic aromatic substrates also furnished the corresponding branched ethers in good yields (**30**–**34**, 53–98% yield), except for 9-anthracenyl *N*-triftosylhydrazone, which is likely due to the poor solubility of this hydrazone (**29**, 36% yield). Importantly, the reaction proved compatible with heteroaryl *N*-triftosylhydrazones (pyridyl, quinolyl, and thienyl), in spite of the potential strong coordination of these heteroatoms to silver (**35**–**38**, 45–65% yield), which shows this chemistry is well-suited for the synthesis of bioactive heterocycle-bearing branched ethers. Intermolecular C–H insertion with donor/donor-carbenes is arguably an even greater challenge and has never been reported, since these carbenes are much less electrophilic and more prone to dimerization^35,40^. Gratifyingly, *N*-triftosylhydrazones derived from diarylketones underwent C–H insertion in fair to good yields (**39**–**42**, 39–62% yield) by increasing the concentration of ether (1:1 Et_2_O/CHCl_3_). Extension of the method to *N*-triftosylhydrazones possessing donor and acceptor groups afforded similar results to those observed in the silver-catalyzed ether α-C–H insertions with donor/acceptor diazo compounds (**43**–**45**, 70–82% yield)^52^. Interestingly, the *N*-triftosylhydrazone derived from 2,2,2-trifluoroacetophenone participated smoothly in the reaction, giving **46** in 80% yield (1.2:1 *d.r*.). To our knowledge, there has no report of ether α-C–H insertion using fluorinated diazoalkanes to date^53^.

**Fig. 4:**
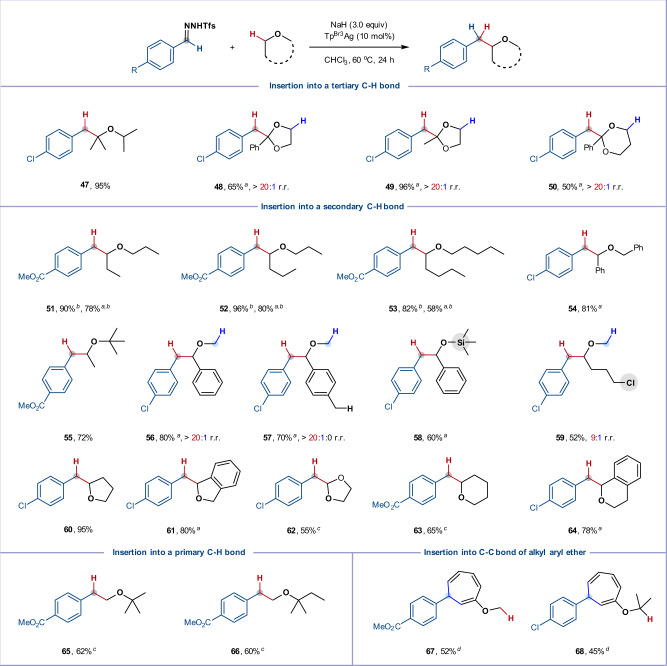
Substrate scope with respective to ethers. Reaction conditions are the same as in Fig. 3. ^*a*^ Ether (2.0 equiv) was used. ^*b*^ PhCF_3_ (5.0 mL) was used. ^*c*^ Ether (5.0 mL) was used. ^*d*^ Ether (5.0 equiv) was used. r.r., regioisomeric ratio.

We next turned our attention to the scope of the ether component. As summarized in Fig. 4, good to excellent yields were achieved on reaction of *N*-triftosylhydrazones derived from 4-chlorobenzaldehyde or 4-(methoxycarbonyl)benzaldehyde with a variety of acyclic and cyclic ethers. In many cases, these reactions could be conducted using just two equivalents of ether, demonstrating the applicability of this method to more valuable complex ethers. Hindered isopropyl ether reacted smoothly and furnished product **47** in 95% yield. Insertion of a variety of acetals proceeded exclusively at benzylic or tertiary alkyl sites, in which the C–H bonds are doubly activated (**48**–**50**, 50–96% yield, >20:1 r.r.). In good agreement with previous reports^20–26^, dialkyl ethers afforded single α-C–H insertion products in high yields, while benzyl ethers reacted exclusively at the benzylic C–H bonds (**51**–**58**). For methyl 4-chlorobutyl ether, insertion occurred into the C–H bonds α- to the ether oxygen; no reaction at or adjacent to the C–Cl bond was observed (**59**)^54^. Excitingly, various saturated cyclic ethers, such as tetrahydrofuran (**60**), 1,3-dihydroisobenzofuran (**61**), 1,3-dioxolane (**62**), tetrahydropyran (**63**), and isochromane (**64**), smoothly furnished the corresponding benzylated oxacycles in good to excellent yields. C–H insertions into methyl ethers proved more challenging, but reasonable yields of **65** and **66** could be obtained by increasing the concentration of ether (1:1 ether/CH_2_Cl_2_). However, for arylalkyl ethers, the major product was instead a norcaradiene (**67** and **68**), generated by a Büchner reaction, rather than the C–H functionalization product. Aside from this limitation, this intermolecular silver-catalyzed carbene insertion enables the synthesis of diverse α-branched ethers, offering an attractive alternative where traditional Williamson ether synthesis and/or Mitsunobu reactions would prove challenging^55,56^. Note that moderate yields were obtained in some cases (**3**, **48**–**54**, **56**–**58**, **61**, and **64**) when using only two equivalents of ethers. The diminished yields were mainly ascribed to the carbene dimerization rather than the C–H over insertion. Over-insertion of C–H bonds on the other side of the oxygen atom indeed occurred, however, this could be effectively avoided by increasing the concentration of ethers (for the details see Supplementary Scheme 1).

### Intramolecular C–H Insertion

**Fig. 5:**
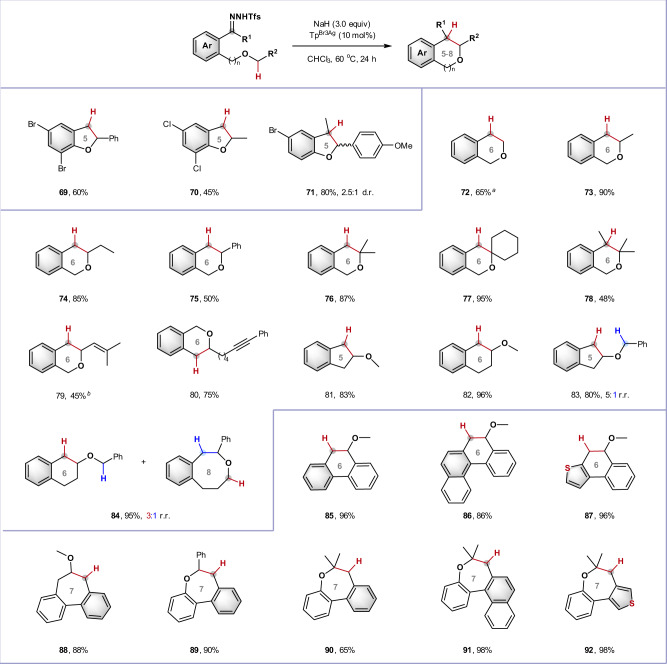
Silver-catalyzed intramolecular carbene insertion into C(sp^3^)–H bonds adjacent to ether oxygen. Standard conditions: *N*-Triftosylhydrazone (0.3 mmol), NaH (0.9 mmol), Tp^Br3^Ag (10 mol%), and CHCl_3_ (5.0 mL), under argon atmosphere at 60 °C for 24 h, isolated yield. ^*a*^ CHCl_3_ (10.0 mL) was used. r.r., regioisomeric ratio; d.r., diastereomeric ratio.

To further expand the potential utility of this silver catalyzed carbene C–H insertion, we turned our attention to intramolecular reactions (Fig. 5). *N*-triftosylhydrazones bearing an ether side-chain underwent the desired intramolecular C–H insertion to give 2,3-dihydrobenzofurans in moderate to good yields (**69**–**71**). The dihydrobenzofuran framework is widely found in biologically important natural products and synthetic compounds^29,36,40^; for example, product **71** could be converted into a natural product (±)-*epi*-conocarpan in two steps^36^. Due to poor reaction kinetics, there are few examples of 1,6-C–H insertions to synthesize six-membered heterocycles^41^; however, on installing the requisite oxygen atom at the 5-position, we found that *N*-triftosylhydrazones underwent successful 1,6-C–H insertion, providing a collection of isochromans in excellent yield (**72**–**80**), with primary (**72**), secondary (**73**–**75**) and tertiary (**78**) C–H bonds being suitable for this transformation. As the carbene insertion into relatively unreactive primary C–H bonds was sluggish under the standard conditions, a more diluted reaction system was required (**72**). Interestingly, an allylic ether afforded the C–H insertion product **79** in 45% yield along with 50% yield of the cyclopropanation product. *N*-triftosylhydrazones bearing an alkyne group gave desired C–H insertion product (**80**) in 75% yield and almost no cyclopropenation or intramolecular 1,3-dipolar cycloaddition products were observed.

We studied the effect of ring size on the cyclization using substrates possessing more than one potential insertion site. Methyl ethers exclusively favored secondary C–H insertion in enabling the formation of 5-membered (indane **81**) and 6-membered rings (tetrahydronaphthalene **83**), while equivalent benzyl ether substrates delivered a mixture of products in which the activated benzylic C–H bond could compete with the kinetically favored secondary C–H bond, even in the case of 8-membered ring formation (**82** and **84**). The ability to form six or seven-membered rings was further exploited using a series of biaryls, prepared by straightforward Suzuki–Miyaura coupling; these underwent regioselective C–H insertion to produce a range of six- and seven-membered tri/tetracyclic products where the ether oxygen atom is located inside or outside the new ring (**85**–**92**).

### Gram-scale reaction and further transformations

**Fig. 6:**
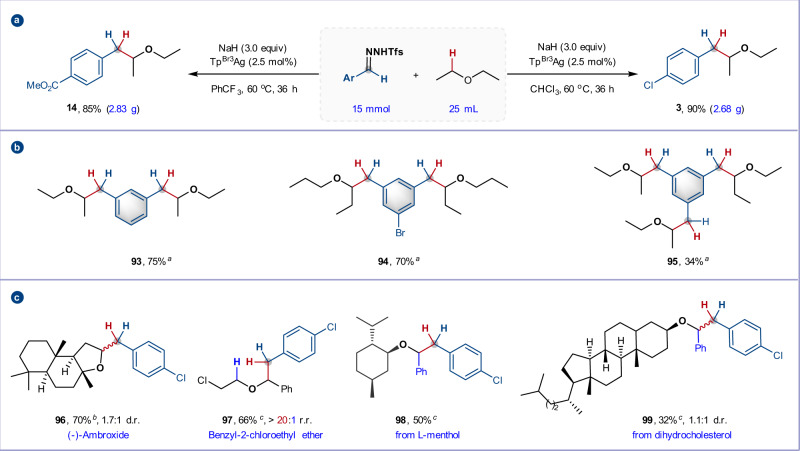
Gram-scale synthesis and synthetic applications. **a** Gram-scale reactions. **b** Application as cross-linker for C(sp^3^)–H bonds. **c** Late-stage C–H benzylation of complex molecules. Isolated yield. ^*a*^ Ether (5.0 mL) was used. ^*b*^ Ether (5.0 equiv.) was used. ^*c*^ Ether (2.0 equiv.) was used. r.r., regioisomeric ratio. d.r., diastereomeric ratio.

We were able to demonstrate the robustness and scalability of the method through multigram-scale preparations of branched ethers. At decreased catalyst loadings (2.5 mol%), use of 15 mmol of *N*-triftosylhydrazone and 25 mL Et_2_O in chloroform or trifluorobenzene furnished C–H insertion products **3** and **14** in 90 and 85% yield, respectively (Fig. 6a). This scalability inspired us to consider potential applications beyond small molecule synthesis, such as in the cross-linking of aliphatic polymers where *bis*-carbene precursors have recently been exploited^57^. As a proof of principle (Fig. 6b), we were pleased to find that *bis-N*-triftosylhydrazones derived from 1,3-benzenedicarbaldehydes underwent double C–H insertion with dialkyl ethers in synthetically useful yields (**93** and **94**). Extension of the method to *tris*-*N*-triftosylhydrazone gave the corresponding triple C–H insertion product in lower yield, potentially due to the insolubility of *N*-triftosylhydrazone (**95**, 34% yield), demonstrating the potential of these poly-hydrazones as bench-stable cross-linking agents.

We were pleased to find that the silver-catalyzed α-C–H insertion could also be applied to highly selective late-stage functionalizations of relatively complex substrates. The natural product (–)-ambroxide, containing two α-C–H ether bonds among numerous other C–H bonds, selectively reacted at the expected C2 position of the tetrahydrofuran ring (70% yield, 1.7:1 d.r.). The reaction of pharmaceutical intermediate benzyl-2-chloroethyl ether exhibited remarkably high site- and chemo-selectivity to provide **97** as a single product in 66% yield. The benzyl ethers of the natural products *L*-menthol and dihydrocholesterol also participated smoothly in the reaction to give products **98** (72%) and **99** (62%) respectively.

### Relative reactivity of donor silver carbenes towards C(sp^3^)–H bonds

**Fig. 7:**
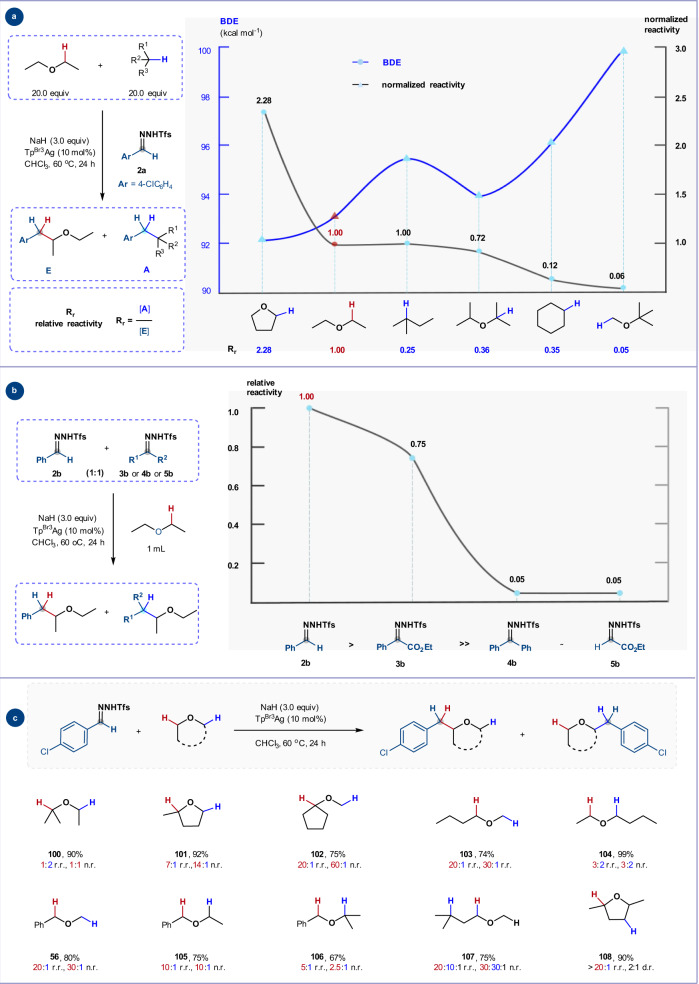
Investigation on relative reactivity and site-selectivity of C(sp^3^)–H bonds with donor silver carbenes. **a** Relative reactivities of C(sp^3^)–H bonds towards the donor silver carbene derived from **2a**. **b** Relative reactivities of different silver carbenes towards ether α-C–H bonds. **c** Site-selectivity investigation of C(sp^3^)–H bonds with subtle electronic and steric differences adjacent to ether oxygen. r.r., regioisomeric ratio; n.r., normalized regioselectivity; d.r., diastereomeric ratio.

To obtain insight into the importance of steric and electronic effects on the site-selectivity of C(sp^3^)–H insertion, we measured the relative reactivities of C(sp^3^)–H bonds in ethers or alkanes towards the donor silver carbene generated in situ from *N*-triftosylhydrazone **2a**, with Et_2_O as the reference. As illustrated in the graph presented in Fig. 7a, the relative reactivities of different C(sp^3^)–H bonds towards the putative silver carbene follow the order α-C–H bonds of THF > α-C–H bonds of Et_2_O ≈ tertiary C–H bonds of 2-methylbutane > tertiary C–H bonds of isopropyl ether > secondary C–H bonds of cyclohexane » α-C–H bond of *tert*-butyl methyl ether (‘normalized’ reactivity was obtained by dividing the observed product ratio by the number of identical C–H bonds in each molecule)^58^. Comparison of these relative reactivities with the corresponding BDEs revealed a weak correlation, which suggests C–H reactivity is controlled by both electronic and steric factors. Competition experiments revealed that the relative reactivities of silver carbenes towards the α-C–H bonds of Et_2_O follow the trend: donor carbene > donor/acceptor carbene » acceptor carbene ≈ donor/donor carbene (Fig. 7b), which is opposite to the known order of the relative reactivities of metal carbenes towards alkane C–H bonds^15,16^.

We further probed the site-selectivity of C–H insertion using ethers that feature different C–H environments at their α-carbon atoms (i.e., internal competition experiments, Fig. 7c). For ethers containing tertiary and secondary C–H bonds, 2-methyltetrahydrofuran (**101**) displayed a tertiary/secondary C–H insertion ratio (*r.r*.) of 7:1 (normalized ratio *n.r*. 14:1), while ethyl isopropyl ether (**100**) exhibited a tertiary/secondary ratio of 1:2 (*n.r*. 1:1). This suggests that in a conformationally restricted environment, there is a strong preference for tertiary C–H activation, while the reduced selectivity in **100** may relate to conformational effects during the insertion process^59^. On the other hand, primary α-C–H insertion is disfavored irrespective of environment, with both methoxycyclopentane **102** and *n-*butyl methyl ether **103** affording the tertiary (*r.r*. 20:1, *n.r*. 60:1) and secondary (*r.r*. 20:1, *n.r*. 30:1) C–H insertion products respectively. Perhaps unsurprisingly, the insertion reaction of *n*-butyl ethyl ether **104** proceeded with little selectivity on both flanks of the oxygen atom.

For benzyl alkyl ethers, we found that electronically-favored benzylic C–H bonds were preferentially functionalized, and this preference decreased with increasing degree of substitution of the alkyl substituent (**56**, **105**, and **106**). Ethyl isoamyl ether **107**, which contains two different types of α-C–H ether bonds (1°, 2°) along with normal 1°, 2°, and 3° C(sp^3^)–H bonds, underwent competing insertion at the 2° α-C–H, 3° C–H and 1° α-C–H bonds with 20:10:1 *r.r*. The normalized reactivity of these C–H bonds is 30:30:1, which is in agreement with the results from the intermolecular competition reactions between Et_2_O with 2-methylbutane, or Et_2_O with *tert*-butyl methyl ether (Fig. 7a). In the case of 2,5-dimethyltetrahydrofuran (a mixture of *cis* and *trans* isomers), the carbene insertion occurs exclusively at 2° α-C–H bonds and affords the product **108** in 90% yield with a 2:1 diastereomeric ratio.

### Mechanistic investigations

**Fig. 8:**
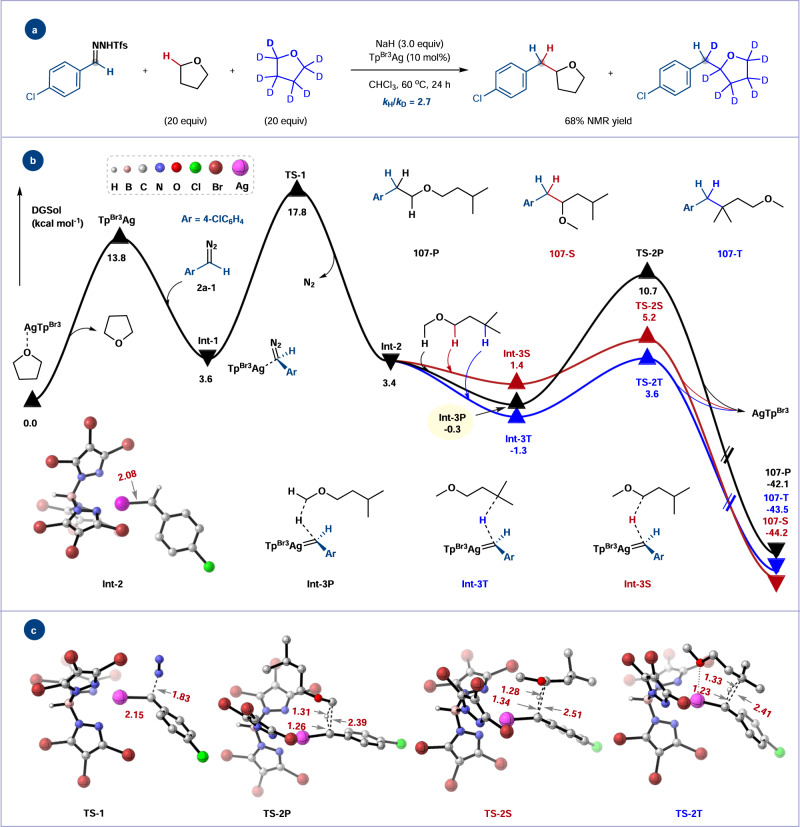
Mechanistic studies. **a** One-pot competition KIE experiment. **b** Computed energy profiles for the silver-catalyzed carbene insertion into C–H bonds of methyl isoamyl ether. **c** Optimized geometrical structures for transition state structures. Calculations were performed at the M06/[6–31 + G(d,p)/SDD(Ag)] level of theory in chloroform within the SMD model. Energies are given in kcal mol^−1^ and distances in angstroms.

To gain insights into the origin of site selectivity of different C–H bonds during C–H functionalization, DFT calculations were conducted at SMD(CHCl_3_)-M06/[6–31 G(d)/SDD(Ag)] level of theory. As shown in Fig. 8b, the reaction between methyl isoamyl ether and diazo compound **2a-1** genertated in situ from *N*-triftosylhydrazone **2a** was chosen as the model reaction, because the formation of the alkyl C–H bond insertion product was not observed in previous C–H insertion reaction of ethers^20–23^. The reaction starts with the dissociation of Tp^Br3^Ag-THF into Tp^Br3^Ag, a process that is uphill by 13.8 kcal mol^−1^. Tp^Br3^Ag is the active catalytic species in the whole catalytic cycle, which is similar to silver-carbene-induced C–H insertion of alkanes^48,58^. Aryl diazo compound **2a-1** coordinates with Tp^Br3^Ag to generate intermediate **Int-1**, which demands an energy barrier of 14.2 kcal mol^−1^ for nitrogen extrusion to form silver carbene **Int-2** via **TS-1**. During this process, methyl isoamyl ether (present in large excess) may compete with **2a-1** to coordinate to the Lewis acidic silver, thus suppressing the formation of silver carbene **Int-2** (see Supplementary Scheme 3 for experimental data supporting the formation of Tp^Br3^Ag-ether complex). The subsequent association and concerted insertion of **Int-2** into various C–H bonds of methyl isoamyl ether occurs via three-membered-ring transition states **TS-2S** (for 2° α-C–H bond)_,_ **TS-2T** (for alkyl 3° C–H bond), and **TS-2P** (for 1° α-C–H bond). The relative potential energies leading to products **107**-**S**, **107**-**T** and **107-P** are respectively 3.8, 4.9 and 11.0 kcal mol^−1^, which is in line with the obtained experimental results (Fig. 7c, compound **107** with 20:10:1 r.r. and 30:30:1 n.r.). According to the energy barrier, the rate-determining step in the whole cycle is the decomposition of **2a-1** to form silver carbene intermediate **Int-2** rather than C–H insertion. Meanwhile, we carried out a one-pot competitive kinetic isotope effect (KIE) experiment of THF and *d*_*8*_-THF, and a primary KIE (*k*_H_/*k*_D_ = 2.7) was observed (Fig. 8a, for details see Supplementary Scheme 2). Under these circumstances, the C–H bond cleavage step is irreversible and should occur after the rate-determining step that does not involve the ether which undergoes C–H bond cleavage^60^. These results suggested a concerted C–H insertion mechanism similar to previously reported rhodium- or silver-catalyzed alkane C–H insertions^19,26,48,61^. The product distributions for both methyl isoamyl ether (compound **107**, Fig. 7c) and the competition experiment between diethyl ether and 2-methylbutane (Fig. 7a) indicate that the normalized reactivity of 2° C–H α to oxygen and aliphatic 3° C–H bonds are almost the same. These results further demonstrate that the coordination of ether to silver does not modulate the reactivity of silver carbene once it is formed. The relative reactivities of 2° α-C–H are much higher than that of aliphatic 2° C–H bonds^48^, which is probably due to the strong electron-donating effect of alkoxy group that is beneficial to the buildup of positive charge^62^. These experimental observations and DFT calculations indicated that ethers (present in large excess) may compete with diazo componds to coordinate to the Lewis acidic silver center, thus suppressing the formation of the silver carbene, but unlikely play a critical role in modulating silver carbenes reactivity and controlling product distribution.

Moreover, further optimization of the calculated geometries using single-point energy calculations was performed at higher level of theory, such as M062X, ωB97XD, and B3LYP-D3(BJ), where the site selectivity trend and the relative energies for transition states and intermediates are unchanged (for details see Supplementary Figs. 10–13).

In summary, we have established a site-selective α-C–H ether insertion reaction using nontoxic and bench stable *N*-triftosylhydrazones as donor, donor/donor, and donor/acceptor carbene precursors. This chemistry provides a convenient and practical method for the synthesis of branched homobenzylic ethers by the selective formation of C(sp^3^)–C(sp^3^) bonds at the α-position of ethers. The ready availability of the starting materials, excellent functional group tolerance, high efficiency on a multi-gram-scale, and predictable regioselectivity in late-stage functionalization of complex molecules demonstrate the potential of this method in practice. Further applications of this silver-catalyzed protocol to other C(sp^3^)–H bonds are under investigations in our laboratory.

## Methods

### General procedures for intermolecular ether α-C–H insertion

To an oven-dried sealed tube was charged with *N*-triftosylhydrazone (0.3 mmol), Tp^Br3^Ag(thf) (32.7 mg, 10 mol%), NaH (36.0 mg, 0.9 mmol, 60 wt% dispersion in mineral oil) in an argon-filled glovebox. Anhydrous CHCl_3_ or PhCF_3_ (5.0 mL) and ether (2.0 equiv or 1.0 mL or 5.0 mL) were added. The tube was sealed and rinsed in an ultrasonic bath for 5 min. The resulting mixture was stirred (700 rpm) at 60 °C for 24 h. When the reaction was completed, the crude reaction mixture was allowed to reach room temperature and filtered through a short pad of silica gel using EtOAc as eluent. The filtrate was concentrated in vacuo and purified by column chromatography on silica gel (petroleum ether/EtOAc) to obtain the product.

### General procedures for intramolecular ether α-C–H insertion

To an oven-dried sealed tube was charged with *N*-triftosylhydrazone (0.3 mmol), Tp^Br3^Ag(thf) (32.7 mg, 10 mol%), NaH (36.0 mg, 0.9 mmol, 60 wt% dispersion in mineral oil) in an argon-filled glovebox. Anhydrous CHCl_3_ (5.0 mL) was added. The tube was sealed and rinsed in an ultrasonic bath for 5 min. The resulting mixture was stirred (700 rpm) at 60 °C for 24 h. When the reaction was completed, the crude reaction mixture was allowed to reach room temperature and filtered through a short pad of silica gel with EtOAc as eluent. The filtrate was concentrated in vacuo and purified by column chromatography on silica gel (petroleum ether/EtOAc) to obtain the product.

## Supplementary information

Supplementary Information

## Data Availability

The data that support the findings of this study are available within the paper and its Supplementary Information files. Raw data are available from the corresponding author on reasonable request. Materials and methods, computational studies, experimental procedures, characterization data, ^1^H, ^13^C, ^19^F NMR spectra, and mass spectrometry data are available in the Supplementary Information.
