# Supplementary material for: Silver-catalyzed site-selective C(sp3)−H benzylation of ethers with N-triftosylhydrazones
Source: Nat Commun. 2022 Mar 30;13:1674. doi: 10.1038/s41467-022-29323-3 (PMC8967862; doi:10.1038/s41467-022-29323-3)
Supplement: Supplementary file 1 — Supplementary Information [file 41467_2022_29323_MOESM1_ESM.pdf]

Supplementary Information for

Silver-catalyzed site-selective C(sp^3)−H benzylation of ethers with *N*-triftosylhydrazones

Zhaohong Liu,†[1] Hongwei Wang,†[1] Paramasivam Sivaguru,†[1] Steven P. Nolan,[2] Qingmin Song,[1] Weijie Yu[1], Xinyu Jiang[1], Edward A. Anderson,[3] and Xihe Bi[1,4]*

[1] Department of Chemistry, Northeast Normal University, Changchun 130024, China;

[2] Chemistry Research Laboratory, University of Oxford, 12 Mansfield Road, Oxford, OX1 3TA, U.K.;

[3] Department of Chemistry and Sustainable Chemistry, Ghent University, 281 Krijgslaan S-3, 9000, Ghent Belgium;

[4] State Key Laboratory of Elemento-Organic Chemistry, Nankai University, Tianjin 300071, China.

†These authors contributed equally: Zhaohong Liu, Hongwei Wang, Paramasivam Sivaguru.

*Corresponding author: Xihe Bi, E-mail: bixh507@nenu.edu.cn.

Table of Contents

I. Supplementary Methods S2

1. General information S2

2. Experimental Procedures and Characterization Data S2

2.1 General Procedures for Intermolecular Ether α-C–H Insertion S2

2.2 General Procedures for Intramolecular Ether α-C–H Insertion S3

2.3 Gram-Scale Experiments S3

2.4 Analytical Data of Compounds S4

3. Preparation of Starting Materials S48

3.1 Preparation of Complex Ethers S48

3.2 Preparation of *N*-Triftosylhydrazones S49

3.3 Characterization Data for Unknown Subtrates S50

4. Detailed Optimization of Conditions S59

5. Competition Experiments S60

6. Substrates Ineffective for α-C-H Insertion of Ethers S63

7. Mechanistic Studies S63

7.1 KIE Experiment S63

7.2 Ether Exchange Experiment S64

7.3 Computational Details S65

8. NMR Spectra of Products S182

II. Supplementary References S329

I. Supplementary Methods

1. General Information

The products were purified by column chromatography over silica gel. NMR spectra were recorded on a Brüker Advance 600 (^{1}H: 600 MHz, ^{13}C:151 MHz) and Brüker Advance 500 (^{1}H: 500 MHz, ^{13}C: 126 MHz) at ambient temperature. The following residual solvent signals were used as references for ^{1}H and ^{13}C NMR spectra: $CDCl_3$, δ_H 0.00 ppm (relative to TMS), δ_C 77.00 ppm; DMSO-d_6, δ_H 2.50 ppm, δ_C 40.00 ppm. ^{19}F NMR chemical shifts were determined relative to $CFCl_3$ as outside standard and low field is positive. The following abbreviations were used to explain the multiplicities: s = singlet, d = doublet, t = triplet, q = quartet, qi = quintet, m = multiplet, br = broad. Thin layer chromatographic (TLC) analysis was performed with glass-backed silica gel plates, visualizing with UV light (254 nm) and/or staining with aqueous $KMnO_4$ stain. Mass spectra were recorded on TSQ 8000 Evo by using EI method. High-resolution mass spectra (HRMS) were recorded on Bruck microTof by using ESI method (Northeast Normal University) and Magnetic Sector High-Resolution Gas Chromatography-Mass Spectra by using ESI method (Guangdong University of Technology). Melting points are uncorrected.

Anhydrous chloroform ($CHCl_3$) was dried and degassed at reflux over CaH in a 250 mL round bottom flask for 3 hours under argon atmosphere, distilled, then stored under argon atmosphere and was used directly. Superdry benzotrifluoride ($PhCF_3$, 99.5%, water ≤ 10 ppm, with molecular sieve) was purchased from J&K Scientific. Ethers were dried following standard methods and freshly distilled to remove inhibitor and peroxide impurities. The others were commercially available and were used as received. Rh_2(*S*-DOSP)$_4$ was purchased from Aldrich. The catalyst Tp^{Br3}Ag(thf) and Tp^{Br3}Cu(NCMe) were prepared in analogy to the literature known procedures developed by the group of Pérez[1,2].

2. Experimental Procedures and Characterization Data

2.1 General Procedures for Intermolecular Ether α-C–H Insertion

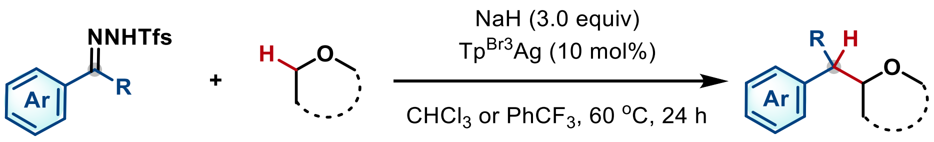

General Procedure A: To an oven-dried sealed tube was charged with *N*-triftosylhydrazone (0.3 mmol, 1.0 equiv), Tp^{Br3}Ag(thf) (32.7 mg, 10 mol%), NaH (36.0 mg, 0.9 mmol, 3.0 equiv, 60 wt% dispersion in mineral oil) in an argon-filled glovebox. Anhydrous $CHCl_3$ or $PhCF_3$ (5.0 mL) and ether (2.0 equiv or 1.0 mL or 5.0 mL) were added. The tube was sealed and rinsed in an ultrasonic bath for 5 min. The resulting mixture was stirred (700 rpm) at 60 °C for 24 h. When the reaction was completed, the crude reaction mixture was allowed to reach room temperature and filtered

through a short pad of silica gel with EtOAc as eluent. The filtrate was concentrated in vacuo and the regioisomer ratio or diastereoisomer ratio was determined by ^{1}H NMR spectroscopy of the crude reaction mixture. The reaction mixture was purified by column chromatography on silica gel (petroleum ether/EtOAc) to afford the corresponding compounds.

2.2 General Procedures for Intramolecular ether α-C–H Insertion

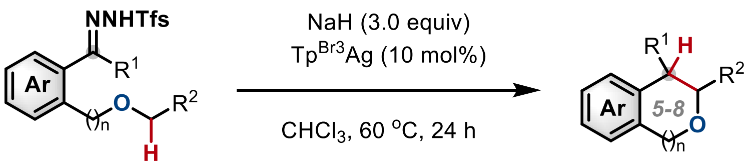

General Procedure B: To an oven-dried sealed tube was charged with *N*-triftosylhydrazone (0.3 mmol, 1.0 equiv), Tp^{Br3}Ag(thf) (32.7 mg, 10 mol%), NaH (36.0 mg, 0.9 mmol, 3.0 equiv, 60 wt% dispersion in mineral oil) in an argon-filled glovebox. Anhydrous $CHCl_3$ (5.0 mL or 10 mL) was added. The tube was sealed and rinsed in an ultrasonic bath for 5 min. The resulting mixture was stirred (700 rpm) at 60 ℃ for 24 h. When the reaction was completed, the crude reaction mixture was allowed to reach room temperature and filtered through a short pad of silica gel with EtOAc as eluent. The filtrate was concentrated in vacuo and purified by column chromatography on silica gel (petroleum ether/EtOAc) to afford the corresponding compounds.

2.3 Gram-Scale Experiments

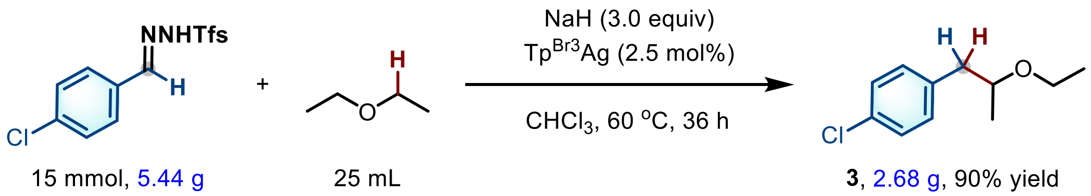

An oven-dried 250 mL Schlenk tube containing a stir bar was charged with *N*-triftosylhydrazone derived from 4-chlorobenzaldehyde (5.44 g, 15.0 mmol, 1.0 equiv), Tp^{Br3}Ag(thf) (412.5 mg, 2.5 mol%), NaH (1.80 g, 0.9 mmol, 3.0 equiv, 60 wt% dispersion in mineral oil) in an argon-filled glovebox. Anhydrous $CHCl_3$ (125 mL) and diethyl ether (25 mL) were added. The tube was sealed and rinsed in an ultrasonic bath for 5 min. The resulting mixture was stirred (700 rpm) at 60 ℃ for 36 h. When the reaction was completed, the crude reaction mixture was allowed to reach room temperature and filtered through a short pad of silica gel with EtOAc as eluent. The filtrate was concentrated in vacuo and purified by column chromatography on silica gel (petroleum ether/EtOAc) to afford **3** (2.68 g, 90% yield) as a colorless oil.

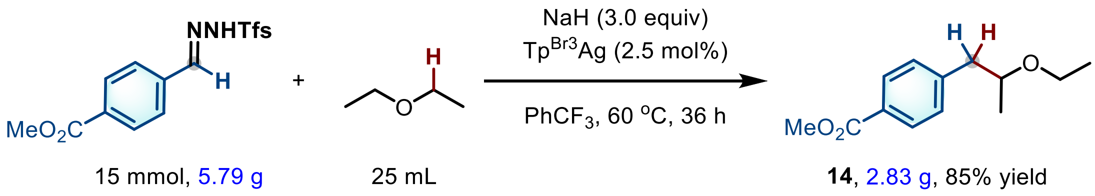

An oven-dried 250 mL Schlenk tube containing a stir bar was charged with *N*-triftosylhydrazone derived from methyl 4-formylbenzoate (5.79 g, 15.0 mmol, 1.0 equiv), $Tp^{Br3}Ag(thf)$ (412.5 mg, 2.5 mol %), NaH (1.80 g, 0.9 mmol, 3.0 equiv, 60 wt% dispersion in mineral oil) in an argon-filled glovebox. Superdry $PhCF_3$ (125 mL) and diethyl ether (25 mL) were added. The tube was sealed and rinsed in an ultrasonic bath for 5 min. The resulting mixture was stirred (700 rpm) at 60 ℃ for 36 h. When the reaction was completed, the crude reaction mixture was allowed to reach room temperature and filtered through a short pad of silica gel with EtOAc as eluent. The filtrate was concentrated in vacuo and purified by column chromatography on silica gel (petroleum ether/EtOAc) to afford **14** (2.83 g, 85% yield) as a colorless oil.

2.4 Analytical Data of Compounds

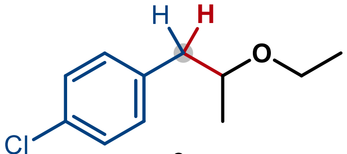

3

(**3**) According to the general procedure A, using *N*-triftosylhydrazone derived from 4-chloro-benzaldehyde (108.8 mg, 0.3 mmol), diethyl ether (1.0 mL) and $CHCl_3$ (5.0 mL) afforded compound **3** (57.2 mg, 96% yield) as a colorless oil. **^{1}H NMR** (600 MHz, $CDCl_3$) δ 7.24 (d, *J* = 8.4 Hz, 2H), 7.13 (d, *J* = 8.4 Hz, 2H), 3.59-3.55 (m, 1H), 3.54-3.50 (m, 1H), 3.41-3.36 (m, 1H), 2.83 (dd, *J* = 13.8 Hz, *J* = 6.6 Hz, 1H), 2.61 (dd, *J* = 13.8 Hz, *J* = 6.6 Hz, 1H), 1.15 (t, *J* = 6.6 Hz, 3H), 1.12 (d, *J* = 6.6 Hz, 3H); **^{13}C NMR** (151 MHz, $CDCl_3$) δ 137.66, 131.78, 130.76, 128.21, 76.11, 63.99, 42.40, 19.60, 15.50; **HRMS** (ESI) m/z calcd for $C_{11}H_{16}OCl$ $[M+H]^+$ 199.0890, Found: 199.0889.

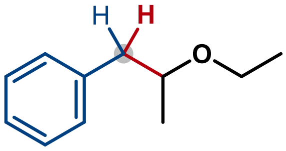

4

(**4**) According to the general procedure A, using *N*-triftosylhydrazone derived from benzaldehyde (98.5 mg, 0.3 mmol), diethyl ether (5.0 mL) and $CHCl_3$ (5.0 mL) afforded compound **4** (40.4 mg, 82% yield) as a colorless oil. **^{1}H NMR** (600 MHz, $CDCl_3$) δ 7.29-7.26 (m, 2H), 7.21-7.18 (m, 3H), 3.63-3.58 (m, 1H), 3.56-3.51 (m, 1H), 3.46-3.41 (m, 1H), 2.92 (dd, *J* = 13.8 Hz, *J* = 6.0 Hz, 1H), 2.61 (dd, *J* = 13.8 Hz, *J* = 6.6 Hz, 1H), 1.16 (t, *J* = 7.2 Hz, 3H), 1.12 (d, *J* = 6.6 Hz, 3H); **^{13}C NMR** (151 MHz, $CDCl_3$) δ 139.18, 129.42, 128.14, 125.97, 76.43, 63.91, 43.11, 19.62, 15.54; **HRMS** (ESI) m/z calcd for $C_{11}H_{17}O$ $[M+H]^+$ 165.1279, Found: 165.1276.

Spectroscopic data are in agreement with those reported in the literature.[3]

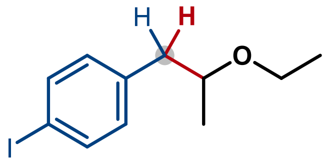

5

(**5**) According to the general procedure A, using *N*-triftosylhydrazone derived from 4-iodo-benzaldehyde (136.3 mg, 0.3 mmol), diethyl ether (1.0 mL) and $CHCl_3$ (5.0 mL) afforded compound **5** (80.0 mg, 92% yield) as a colorless oil. **^{1}H NMR** (600 MHz, $CDCl_3$) δ 7.58 (d, *J* = 8.4 Hz, 2H), 6.95 (d, *J* = 8.4 Hz, 2H), 3.58-3.49 (m, 2H), 3.40-3.35 (m, 1H), 2.80 (dd, *J* = 13.8 Hz, *J* = 6.0 Hz, 1H), 2.57 (dd, *J* = 13.8 Hz, *J* = 6.0 Hz, 1H), 1.14 (t, *J* = 7.2 Hz, 3H), 1.11 (d, *J* = 6.0 Hz, 3H); **^{13}C NMR** (151 MHz, $CDCl_3$) δ 138.75, 137.08, 131.47, 91.17, 75.93, 63.91, 42.48, 19.57, 15.47; **HRMS** (ESI) m/z calcd for $C_{11}H_{16}OI$ $[M+H]^+$ 291.0246, Found: 291.0249.

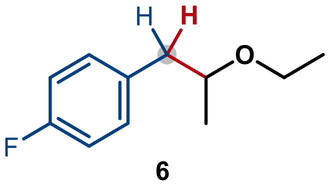

6

(**6**) According to the general procedure A, using *N*-triftosylhydrazone derived from 4-fluoro-benzaldehyde (103.9 mg, 0.3 mmol) , diethyl ether (1.0 mL) and $CHCl_3$ (5.0 mL) afforded compound **6** (38.3 mg, 70% yield) as a colorless oil. **^{1}H NMR** (600 MHz, $CDCl_3$) δ 7.15 (dd, *J* = 8.4 Hz, *J* = 5.4 Hz, 2H), 6.96 (t, *J* = 8.4 Hz, 2H), 3.59-3.50 (m, 2H), 3.42-3.37 (m, 1H), 2.84 (dd, *J* = 13.8 Hz, *J* = 6.6 Hz, 1H), 2.61 (dd, *J* = 13.8 Hz, *J* = 6.0 Hz, 1H), 1.15 (t, *J* = 7.2 Hz, 3H), 1.12 (d, *J* = 6.0 Hz, 3H); **^{13}C NMR** (151 MHz, $CDCl_3$) δ 161.44 (d, *J* = 243.3 Hz), 134.82 (d, *J* = 2.6 Hz), 130.76 (d, *J* = 7.6 Hz), 114.86 (d, *J* = 20.9 Hz), 76.30, 63.98, 42.22, 19.57, 15.52; **^{19}F NMR** (471 MHz, $CDCl_3$) δ (-117.55) − (-117.61) (m, 1F); **HRMS** (ESI) m/z calcd for $C_{11}H_{16}OF$ $[M+H]^+$ 183.1185, Found: 183.1190.

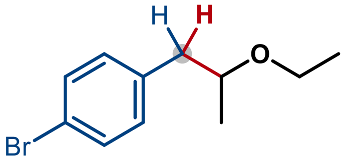

7

(**7**) According to the general procedure A, using *N*-triftosylhydrazone derived from 4-bromo-benzaldehyde (122.2 mg, 0.3 mmol), diethyl ether (1.0 mL) and $CHCl_3$ (5.0 mL) afforded compound **7** (70.0 mg, 96% yield) as a colorless oil. **^{1}H NMR** (600 MHz, $CDCl_3$) δ 7.39 (d, *J* = 8.4 Hz, 2H), 7.08 (d, *J* = 8.4 Hz, 2H), 3.59-3.50 (m, 2H), 3.41-3.36 (m, 1H), 2.82 (dd, *J* = 13.8 Hz, *J* = 6.6 Hz, 1H), 2.59 (dd, *J* = 13.8 Hz, *J* = 6.6 Hz, 1H), 1.15 (t, *J* = 7.2 Hz, 3H), 1.12 (d, *J* = 6.6

Hz, 3H); **^{13}C NMR** (151 MHz, $CDCl_3$) δ 138.19, 131.19 (2C), 119.86, 76.06, 64.01, 42.48, 19.62, 15.51; **HRMS** (ESI) m/z calcd for $C_{11}H_{16}OBr$ $[M+H]^+$ 243.0385, Found: 243.0382.

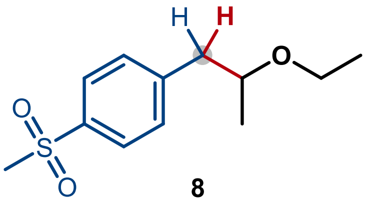

8

(**8**) According to the general procedure A, using *N*-triftosylhydrazone derived from 4-(methylsulfonyl)benzaldehyde (121.9 mg, 0.3 mmol), diethyl ether (1.0 mL) and $PhCF_3$ (5.0 mL) afforded compound **8** (47.9 mg, 66% yield) as a colorless oil. **^{1}H NMR** (500 MHz, $CDCl_3$) δ 7.86 (d, *J* = 8.0 Hz, 2H), 7.42 (d, *J* = 8.0 Hz, 2H), 3.67-3.61 (m, 1H), 3.58-3.52 (m, 1H), 3.40-3.34 (m, 1H), 3.06 (s, 3H), 2.92 (dd, *J* = 13.5 Hz, *J* = 7.0 Hz, 1H), 2.78 (dd, *J* = 13.5 Hz, *J* = 5.5 Hz, 1H), 1.16-1.13 (m, 6H); **^{13}C NMR** (126 MHz, $CDCl_3$) δ 145.82, 138.11, 130.38, 127.15, 75.57, 64.03, 44.54, 42.82, 19.67, 15.42; **HRMS** (ESI) m/z calcd for $C_{12}H_{19}O_3S$ $[M+H]^+$ 243.1055, Found: 243.1053.

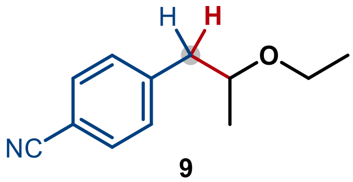

9

(**9**) According to the general procedure A, using *N*-triftosylhydrazone derived from 4-formylbenzonitrile (106.0 mg, 0.3 mmol), diethyl ether (5.0 mL) and $CHCl_3$ (5.0 mL) afforded compound **9** (35.2 mg, 62% yield) as a colorless oil. **^{1}H NMR** (600 MHz, $CDCl_3$) δ 7.57 (d, *J* = 8.4 Hz, 2H), 7.32 (d, *J* = 8.4 Hz, 2H), 3.64-3.59 (m, 1H), 3.56-3.51 (m, 1H), 3.37-3.32 (m, 1H), 2.88 (dd, *J* = 13.8 Hz, *J* = 7.2 Hz, 1H), 2.73 (dd, *J* = 13.8 Hz, *J* = 5.4 Hz, 1H), 1.15 (d, *J* = 6.0 Hz, 3H), 1.13 (d, *J* = 6.6 Hz, 3H); **^{13}C NMR** (151 MHz, $CDCl_3$) δ 144.94, 131.87, 130.23, 119.07, 109.91, 75.59, 64.05, 43.17, 19.70, 15.40; **HRMS** (ESI) m/z calcd for $C_{12}H_{16}NO$ $[M+H]^+$ 190.1232, Found: 190.1236.

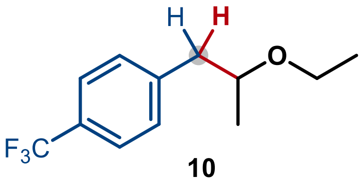

10

(**10**) According to the general procedure A, using *N*-triftosylhydrazone derived from 4-(trifluoromethyl)- benzaldehyde (118.9 mg, 0.3 mmol), diethyl ether (1.0 mL) and $PhCF_3$ (5.0 mL) afforded compound **10** (62.7 mg, 90% yield) as a colorless oil. **^{1}H NMR** (600 MHz, $CDCl_3$) δ 7.53 (d, *J* = 8.4 Hz, 2H), 7.32 (d, *J* = 8.4 Hz, 2H), 3.64-3.59 (m, 1H), 3.56-3.51 (m, 1H), 3.41-3.36 (m, 1H), 2.91 (dd, *J* = 13.8 Hz, *J* = 6.6 Hz, 1H), 2.71 (dd, *J* = 13.8 Hz, *J* = 6.0 Hz, 1H), 1.16-1.13 (m, 6H); **^{13}C NMR** (151 MHz, $CDCl_3$) δ 143.36, 129.74, 128.39 (q, *J* = 32.2 Hz), 125.01 (q, *J* = 4.1 Hz), 124.38 (q, *J* = 271.7 Hz), 75.89, 64.03, 42.89, 19.66, 15.46; **^{19}F NMR** (471 MHz, $CDCl_3$) δ -62.33 (s, 3F); **HRMS** (ESI) m/z calcd for $C_{12}H_{16}OF_3$ $[M+H]^+$ 233.1153, Found: 233.1152.

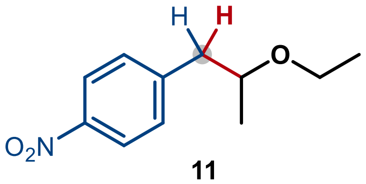

11

(**11**) According to the general procedure A, using *N*-triftosylhydrazone derived from 4-nitrobenzaldehyde (112.0 mg, 0.3 mmol), diethyl ether (5.0 mL) and $CHCl_3$ afforded compound **11** (52.7 mg, 84% yield) as a colorless oil. **^{1}H NMR** (600 MHz, $CDCl_3$) δ 8.14 (d, *J* = 8.4 Hz, 2H), 7.38 (d, *J* = 8.4 Hz, 2H), 3.67-3.62 (m, 1H), 3.57-3.52 (m, 1H), 3.37-3.32 (m, 1H), 2.92 (dd, *J* = 13.8 Hz, *J* = 7.2 Hz, 1H), 2.79 (dd, *J* = 13.8 Hz, *J* = 5.4 Hz, 1H), 1.15 (d, *J* = 6.0 Hz, 3H), 1.13 (t, *J* = 7.2 Hz, 3H); **^{13}C NMR** (151 MHz, $CDCl_3$) δ 147.17, 146.49, 130.23, 123.27, 75.52, 64.06, 42.90, 19.71, 15.38; **HRMS** (ESI) m/z calcd for $C_{11}H_{16}NO_3$ $[M+H]^+$ 210.1130, Found: 210.1131.

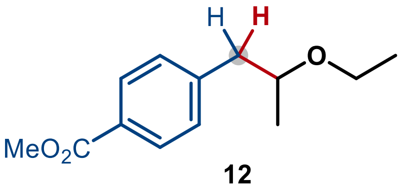

12

(**12**) According to the general procedure A, using *N*-triftosylhydrazone derived from methyl 4-formylbenzoate (115.9 mg, 0.3 mmol), diethyl ether (1.0 mL) and $PhCF_3$ (5.0 mL) afforded compound **12** (65.3 mg, 98% yield) as a colorless oil. **^{1}H NMR** (600 MHz, $CDCl_3$) δ 7.95 (d, *J* = 8.4 Hz, 2H), 7.27 (d, *J* = 8.4 Hz, 2H), 3.90 (s, 3H), 3.65-3.59 (m, 1H), 3.55-3.50 (m, 1H), 3.40-3.35 (m, 1H), 2.92 (dd, *J* = 13.8 Hz, *J* = 6.6 Hz, 1H), 2.70 (dd, *J* = 13.8 Hz, *J* = 6.0 Hz, 1H), 1.15 -1.13 (m, 6H); **^{13}C NMR** (151 MHz, $CDCl_3$) δ 167.12, 144.76, 129.43, 127.99, 75.97, 64.01, 51.91, 43.12, 19.72, 15.44; **HRMS** (ESI) m/z calcd for $C_{13}H_{19}O_3$ $[M+H]^+$ 223.1334, Found: 223.1337.

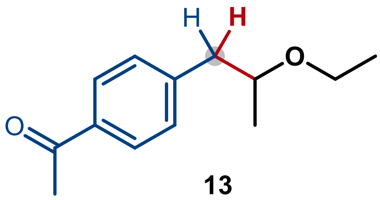

13

(**13**) According to the general procedure A, using *N*-triftosylhydrazone derived from 4-acetylbenzaldehyde (111.1 mg, 0.3 mmol), diethyl ether (1.0 mL) and $CHCl_3$ (5.0 mL) afforded compound **13** (40.2 mg, 65% yield) as a colorless oil. **^{1}H NMR** (600 MHz, $CDCl_3$) δ 7.88 (d, *J* = 8.4 Hz, 2H), 7.30 (d, *J* = 8.4 Hz, 2H), 3.66-3.60 (m, 1H), 3.56-3.51 (m, 1H), 3.41-3.36 (m, 1H), 2.92 (dd, *J* = 13.8 Hz, *J* = 6.6 Hz, 1H), 2.71 (dd, *J* = 13.8 Hz, *J* = 6.0 Hz, 1H), 2.59 (s, 3H), 1.16-1.13 (m, 6H); **^{13}C NMR** (151 MHz, $CDCl_3$) δ 197.90, 145.08, 135.24, 129.66, 128.29, 75.93, 64.05, 43.11, 26.54, 19.75, 15.49; **HRMS** (ESI) m/z calcd for $C_{13}H_{19}O_2$ $[M+H]^+$ 207.1385, Found: 207.1390.

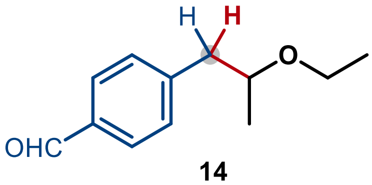

14

(**14**) According to the general procedure A, using *N*-triftosylhydrazone derived from 4-formylbenzal- dehyde (106.9 mg, 0.3 mmol), diethyl ether (5.0 mL) and $CHCl_3$ (5.0 mL) afforded compound **14** (23.1 mg, 40% yield) as a colorless oil. **^{1}H NMR** (600 MHz, $CDCl_3$) δ 9.98 (s, 1H), 7.80 (d, *J* = 8.4 Hz, 2H), 7.38 (d, *J* = 8.4 Hz, 2H), 3.67-3.62 (m, 1H), 3.56-3.51 (m, 1H), 3.40-3.35 (m, 1H), 2.94 (dd, *J* = 13.2 Hz, *J* = 6.6 Hz, 1H), 2.74 (dd, *J* = 13.2 Hz, *J* = 6.0 Hz, 1H), 1.16 -1.13 (m, 6H); **^{13}C NMR** (151 MHz, $CDCl_3$) δ 192.02, 146.72, 134.65, 130.12, 129.66, 75.83, 64.06, 43.31, 19.77, 15.44; **HRMS** (ESI) m/z calcd for $C_{12}H_{17}O_2$ $[M+H]^+$ 193.1229, Found: 193.1225.

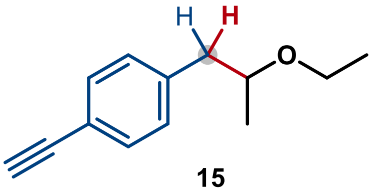

15

(**15**) According to the general procedure A, using *N*-triftosylhydrazone derived from 4-ethynylbenzaldehyde (105.7 mg, 0.3 mmol), diethyl ether (5.0 mL) and $CHCl_3$ (5.0 mL) afforded compound **15** (24.8 mg, 44% yield) as a colorless oil. **^{1}H NMR** (600 MHz, $CDCl_3$) δ 7.41 (d, *J* = 7.8 Hz, 2H), 7.16 (d, *J* = 7.8 Hz, 2H), 3.60-3.56 (m, 1H), 3.55-3.50 (m, 1H), 3.41-3.36

(m, 1H), 3.04 (s, 1H), 2.88 (dd, J = 13.8 Hz, J = 6.0 Hz, 1H), 2.63 (dd, J = 13.8 Hz, J = 6.0 Hz, 1H), 1.14 (t, J = 7.2 Hz, 3H), 1.12 (d, J = 6.0 Hz, 3H); **^{13}C NMR** (151 MHz, $CDCl_3$) δ 140.27, 131.93, 129.43, 119.69, 83.76, 76.67, 76.12, 64.01, 43.04, 19.68, 15.50; **HRMS** (ESI) m/z calcd for $C_{13}H_{17}O$ [M+H]$^+$ 189.1279, Found: 189.1275.

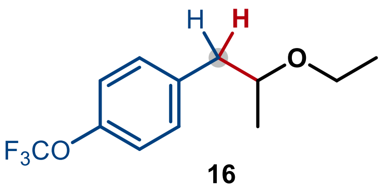

16

(**16**) According to the general procedure A, using *N*-triftosylhydrazone derived from 4-(trifluoromethoxy)- benzaldehyde (123.7 mg, 0.3 mmol), diethyl ether (1.0 mL) and $PhCF_3$ (5.0 mL) afforded compound **16** (48.4 mg, 65% yield) as a colorless oil. **^{1}H NMR** (600 MHz, $CDCl_3$) δ 7.22 (d, J = 8.4 Hz, 2H), 7.12 (d, J = 8.4 Hz, 2H), 3.61-3.56 (m, 1H), 3.55-3.51 (m, 1H), 3.42-3.37 (m, 1H), 2.86 (dd, J = 13.8 Hz, J = 6.0 Hz, 1H), 2.65 (dd, J = 13.8 Hz, J = 6.0 Hz, 1H), 1.16-1.12 (m, 6H); **^{13}C NMR** (151 MHz, $CDCl_3$) δ 147.62, 137.95, 130.67, 120.65, 120.56 (q, J = 256.6 Hz), 76.06, 64.00, 42.33, 19.60, 15.48; **^{19}F NMR** (471 MHz, $CDCl_3$) δ -57.93 (s, 3F); **HRMS** (ESI) m/z calcd for $C_{12}H_{16}O_2F_3$ [M+H]$^+$ 249.1102, Found: 249.1097.

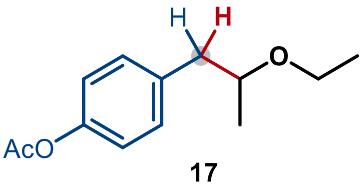

17

(**17**) According to the general procedure A, using *N*-triftosylhydrazone derived from 4-formylphenyl acetate (115.9 mg, 0.3 mmol), diethyl ether (5.0 mL) and $CHCl_3$ (5.0 mL) afforded compound **17** (40.0 mg, 60% yield) as a colorless oil. **^{1}H NMR** (600 MHz, $CDCl_3$) δ 7.20 (d, J = 8.4 Hz, 2H), 6.99 (d, J = 8.4 Hz, 2H), 3.61-3.56 (m, 1H), 3.55-3.52 (m, 1H), 3.44-3.39 (m, 1H), 2.88 (dd, J = 13.8 Hz, J = 6.6 Hz, 1H), 2.61 (dd, J = 13.8 Hz, J = 6.6 Hz, 1H), 2.28 (s 3H), 1.16 (t, J = 7.2 Hz, 3H), 1.13 (d, J = 6.0 Hz, 3H); **^{13}C NMR** (151 MHz, $CDCl_3$) δ 169.55, 148.94, 136.76, 130.31, 121.12, 76.25, 63.92, 42.44, 21.12, 19.60, 15.52; **HRMS** (ESI) m/z calcd for $C_{13}H_{19}O_3$ [M+H]$^+$ 223.1334, Found: 223.1337.

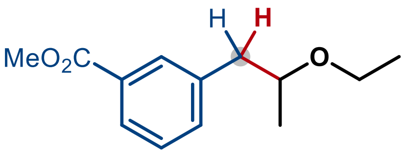

18

(**18**) According to the general procedure A, using *N*-triftosylhydrazone derived from methyl methyl 3-formylbenzoate (115.9 mg, 0.3 mmol), diethyl ether (1.0 mL) and $PhCF_3$ (5.0 mL) afforded compound **18** (56.7 mg, 85% yield) as a colorless oil. **^{1}H NMR** (600 MHz, $CDCl_3$) δ 7.89-7.87 (m, 2H), 7.41 (d, *J* = 7.8 Hz, 1H), 7.35 (t, *J* = 7.8 Hz, 1H), 3.91 (s, 3H), 3.65-3.59 (m, 1H), 3.56-3.51 (m, 1H), 3.43-3.38 (m, 1H), 2.93 (dd, *J* = 13.8 Hz, *J* = 6.6 Hz, 1H), 2.69 (dd, *J* = 13.8 Hz, *J* = 6.6 Hz, 1H), 1.16-1.13 (m, 6H); **^{13}C NMR** (151 MHz, $CDCl_3$) δ 167.26, 139.55, 134.17, 130.47, 130.03, 128.17, 127.34, 76.11, 64.02, 52.03, 42.80, 19.64, 15.48; **HRMS** (ESI) m/z calcd for $C_{13}H_{19}O_3$ $[M+H]^+$ 223.1334, Found: 223.1332.

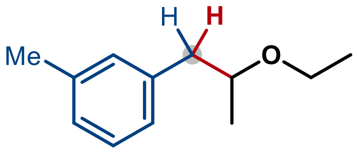

19

(**19**) According to the general procedure A, using *N*-triftosylhydrazone derived from 3-methylbenzaldehyde (102.7 mg, 0.3 mmol), diethyl ether (5.0 mL) and $CHCl_3$ (5.0 mL) afforded compound **19** (40.7 mg, 76% yield) as a colorless oil. **^{1}H NMR** (500 MHz, $CDCl_3$) δ 7.17 (t, *J* = 7.5 Hz, 1H), 7.02-7.00 (m, 3H), 3.62-3.57 (m, 1H), 3.55-3.51 (m, 1H), 3.48-3.41 (m, 1H), 2.90 (dd, *J* = 13.5 Hz, *J* = 6.0 Hz, 1H), 2.56 (dd, *J* = 13.5 Hz, *J* = 7.0 Hz, 1H), 2.33 (s, 3H), 1.17 (t, *J* = 7.0 Hz, 3H), 1.12 (d, *J* = 6.5 Hz, 3H); **^{13}C NMR** (151 MHz, $CDCl_3$) δ 139.09, 137.66, 130.24, 128.04, 126.71, 126.42, 76.50, 63.88, 43.03, 21.37, 19.64, 15.56; **HRMS** (ESI) m/z calcd for $C_{12}H_{19}O$ $[M+H]^+$ 179.1436, Found: 179.1437.

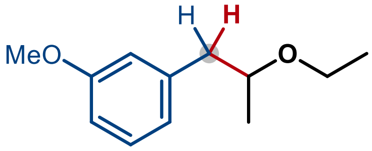

20

(**20**) Following the general procedure **A**, *N*-triftosylhydrazone derived from 3-methoxybenzal-dehyde (107.5 mg, 0.3 mmol), diethyl ether (1.0 mL) and $CHCl_3$ (5.0 mL) afforded compound **20** (29.1 mg, 50% yield) as a colorless oil. **^{1}H NMR** (600 MHz, $CDCl_3$) δ 7.19 (t, *J* = 7.8 Hz, 1H), 6.79 (d, *J* = 7.8 Hz, 1H), 6.77-6.74 (m, 2H), 3.80 (s, 3H), 3.63-3.58 (m, 1H), 3.56-3.51 (m, 1H), 3.46-3.41 (m, 1H), 2.90 (dd, *J* = 13.2 Hz, *J* = 6.0 Hz, 1H), 2.58 (dd, *J* = 13.2 Hz, *J* = 6.6 Hz, 1H), 1.17 (t, *J* = 7.2 Hz, 3H), 1.13 (d, *J* = 6.0 Hz, 3H); **^{13}C NMR** (151 MHz, $CDCl_3$) δ 159.47, 140.82, 129.08, 121.85, 115.15, 111.35, 76.37, 63.92, 55.12, 43.16, 19.69, 15.58; **HRMS** (ESI) m/z calcd for $C_{12}H_{19}O_2$ $[M+H]^+$ 195.1385, Found: 195.1380.

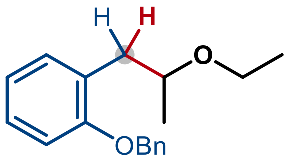

21

(**21**) According to the general procedure A, using *N*-triftosylhydrazone derived from 2-(benzyloxy)benzal- dehyde (130.3 mg, 0.3 mmol), diethyl ether (1.0 mL) and $CHCl_3$ (5.0 mL) afforded compound **21** (38.9 mg, 48% yield) as a colorless oil. **^{1}H NMR** (600 MHz, $CDCl_3$) δ 7.43 (d, *J* = 7.2 Hz, 2H), 7.38 (t, *J* = 7.2 Hz, 2H), 7.31 (t, *J* = 7.2 Hz, 1H), 7.18-7.16 (m, 2H), 6.91-6.88 (m, 2H), 5.06 (s, 2H), 3.72-3.66 (m, 1H), 3.47-3.39 (m, 2H), 3.07 (dd, *J* = 13.2 Hz, *J* = 5.4 Hz, 1H), 2.63 (dd, *J* = 13.2 Hz, *J* = 7.2 Hz, 1H), 1.11-1.09 (m, 6H); **^{13}C NMR** (151 MHz, $CDCl_3$) δ 156.68, 137.31, 131.45, 128.44, 127.95, 127.76, 127.31, 127.21, 120.59, 111.47, 75.01, 69.88, 63.77, 37.62, 19.89, 15.55; **HRMS** (ESI) m/z calcd for $C_{18}H_{23}O_2$ $[M+H]^+$ 271.1698, Found: 271.1701.

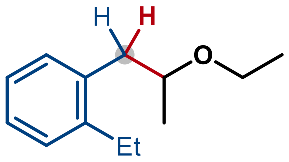

22

(**22**) According to the general procedure A, using *N*-triftosylhydrazone derived from 2-ethylbenzaldehyde (106.9 mg, 0.3 mmol), diethyl ether (1.0 mL) and $CHCl_3$ (5.0 mL) afforded compound **22** (34.0 mg, 59% yield) as a colorless oil. **^{1}H NMR** (500 MHz, $CDCl_3$) δ 7.18-7.10 (m, 4H), 3.64-3.58 (m, 1H), 3.57-3.50 (m, 1H), 3.44-3.38 (m, 1H), 3.00 (dd, *J* = 13.5 Hz, *J* = 6.5 Hz, 1H), 2.73-2.65 (m, 2H), 2.61 (dd, *J* = 13.5 Hz, *J* = 7.5 Hz, 1H), 1.22 (t, *J* = 7.5 Hz, 3H), 1.17-1.14 (m, 6H); **^{13}C NMR** (151 MHz, $CDCl_3$) δ 142.38, 136.73, 130.38, 128.33, 126.32, 125.57, 76.25, 64.02, 39.73, 25.63, 19.79, 15.58, 15.33; **HRMS** (ESI) m/z calcd for $C_{13}H_{21}O$ $[M+H]^+$ 193.1592, Found: 193.1592.

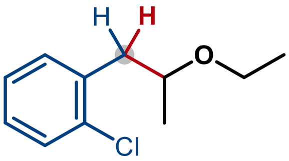

23

(**23**) According to the general procedure A, using *N*-triftosylhydrazone derived from 2-chlorobenzaldehyde (108.8 mg, 0.3 mmol), diethyl ether (1.0 mL) and $CHCl_3$ (5.0 mL) afforded compound **23** (48.3 mg, 81% yield) as a colorless oil. **^{1}H NMR** (600 MHz, $CDCl_3$) δ 7.34-7.32 (m,

1H), 7.26-7.24 (m, 1H), 7.19-7.13 (m, 2H), 3.73-3.68 (m, 1H), 3.56-3.51 (m, 1H), 3.45-3.40 (m, 1H), 3.05 (dd, *J* = 13.2 Hz, *J* = 6.6 Hz, 1H), 2.77 (dd, *J* = 13.2 Hz, *J* = 6.6 Hz, 1H), 1.16 (d, *J* = 6.6 Hz, 3H), 1.13 (t, *J* = 7.2 Hz, 3H); **^{13}C NMR** (151 MHz, $CDCl_3$) δ 136.88, 134.16, 131.95, 129.32, 127.54, 126.47, 74.60, 64.05, 40.68, 19.85, 15.50; **HRMS** (ESI) m/z calcd for $C_{11}H_{16}OCl$ $[M+H]^+$ 199.0890, Found: 199.0889.

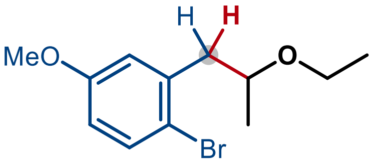

24

(**24**) According to the general procedure A, using *N*-triftosylhydrazone derived from 2-bromo-5-methoxy- benzaldehyde (131.2 mg, 0.3 mmol), diethyl ether (1.0 mL) and $CHCl_3$ (5.0 mL) afforded compound **24** (59.0 mg, 72% yield) as a colorless oil. **^{1}H NMR** (600 MHz, $CDCl_3$) δ 7.40 (d, *J* = 9.0 Hz, 1H), 6.83 (d, *J* = 3.0 Hz, 1H), 6.65 (dd, *J* = 9.0 Hz, *J* = 3.0 Hz, 1H), 3.78 (s, 3H), 3.73-3.68 (m, 1H), 3.57-3.52 (m, 1H), 3.45-3.40 (m, 1H), 3.00 (dd, *J* = 13.8 Hz, *J* = 6.6 Hz, 1H), 2.74 (dd, *J* = 13.8 Hz, *J* = 6.6 Hz, 1H), 1.17 (d, *J* = 6.0 Hz, 3H), 1.14 (t, *J* = 7.2 Hz, 3H); **^{13}C NMR** (151 MHz, $CDCl_3$) δ 158.64, 139.58, 133.07, 117.40, 115.22, 113.68, 74.64, 64.10, 55.40, 43.33, 19.87, 15.55; **HRMS** (ESI) m/z calcd for $C_{12}H_{18}O_2Br$ $[M+H]^+$ 273.0490, Found: 273.0482.

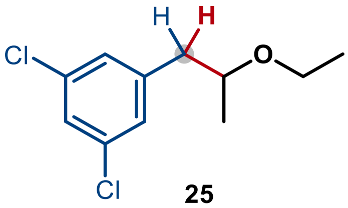

25

(**25**) Following the general procedure **A**, *N*-triftosylhydrazone derived from 3,5-dichlorobenzal-dehyde (119.2 mg, 0.3 mmol), diethyl ether (5.0 mL) and $CHCl_3$ (5.0 mL) afforded compound **25** (65.0 mg, 93% yield) as a colorless oil. **^{1}H NMR** (600 MHz, $CDCl_3$) δ 7.21 (t, *J* = 1.8 Hz, 1H), 7.10 (d, *J* = 1.8 Hz, 2H), 3.60-3.52 (m, 2H), 3.39-3.34 (m, 1H), 2.79 (dd, *J* = 13.8 Hz, *J* = 6.6 Hz, 1H), 2.60 (dd, *J* = 13.8 Hz, *J* = 6.0 Hz, 1H), 1.15 (t, *J* = 7.2 Hz, 3H), 1.14 (d, *J* = 6.6 Hz, 3H); **^{13}C NMR** (151 MHz, $CDCl_3$) δ 142.59, 134.47, 127.95, 126.28, 75.64, 64.11, 42.48, 19.67, 15.45; **HRMS** (ESI) m/z calcd for $C_{11}H_{15}OCl_2$ $[M+H]^+$ 233.0500, Found: 233.0501.

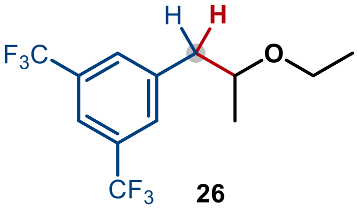

26

(**26**) According to the general procedure A, using *N*-triftosylhydrazone derived from 3,5-*bis*-(trifluoromethyl)benzaldehyde (139.3 mg, 0.3 mmol), diethyl ether (1.0 mL) and $CHCl_3$ (5.0 mL) afforded compound **26** (72.0 mg, 80% yield) as a colorless oil. **^{1}H NMR** (600 MHz, $CDCl_3$) δ 7.73 (s, 1H), 7.69 (s, 2H), 3.63-3.54 (m, 2H), 3.31-3.26 (m, 1H), 2.90 (dd, *J* = 13.8 Hz, *J* = 7.2 Hz, 1H), 2.81 (dd, *J* = 13.8 Hz, *J* = 4.8 Hz, 1H), 1.19 (d, *J* = 6.0 Hz, 3H), 1.12 (t, *J* = 7.2 Hz, 3H); **^{13}C NMR** (151 MHz, $CDCl_3$) δ 141.72, 131.20 (q, *J* = 33.4 Hz), 129.69 (q, *J* = 2.5 Hz), 123.48 (q, *J* = 272.5 Hz), 120.14-120.03 (m), 75.44, 64.20, 42.70, 19.72, 15.27; **^{19}F NMR** (471 MHz, $CDCl_3$) δ -62.84 (s, 6F); **HRMS** (ESI) m/z calcd for $C_{13}H_{15}OF_6$ $[M+H]^+$ 301.1027, Found: 301.1018.

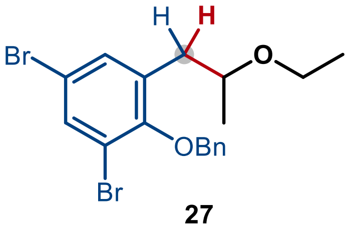

27

(**27**) According to the general procedure A, using *N*-triftosylhydrazone derived from 2-(benzyloxy)-3,5-dibromobenzaldehyde (177.7 mg, 0.3 mmol), diethyl ether (1.0 mL) and $CHCl_3$ (5.0 mL) afforded compound **27** (109.2 mg, 85% yield) as a colorless oil. **^{1}H NMR** (600 MHz, $CDCl_3$) δ 7.59 (d, *J* = 2.4 Hz, 1H), 7.49 (d, *J* = 7.2 Hz, 2H), 7.40 (t, *J* = 7.2 Hz, 2H), 7.37-7.34 (m, 2H), 4.96 (ABq, *J* = 10.8 Hz, 1H), 3.62-3.56 (m, 1H), 3.49-3.44 (m, 1H), 3.31-3.25 (m, 1H), 2.83 (dd, *J* = 13.8 Hz, *J* = 7.2 Hz, 1H), 2.61 (dd, *J* = 13.8 Hz, *J* = 6.0 Hz, 1H), 1.11-1.08 (m, 6H); **^{13}C NMR** (151 MHz, $CDCl_3$) δ 153.49, 136.78, 136.58, 133.79, 133.47, 128.51, 128.31, 128.12, 118.30, 117.02, 75.16, 75.14, 64.05, 37.69, 19.90, 15.44; **HRMS** (ESI) m/z calcd for $C_{18}H_{21}O_2Br_2$ $[M+H]^+$ 426.9908, Found: 426.9908.

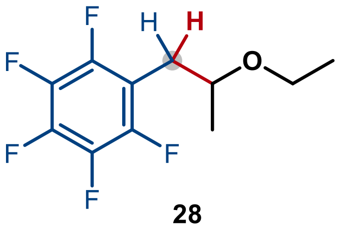

28

(**28**) According to the general procedure A, using *N*-triftosylhydrazone derived from 2,3,4,5,6-pentafluorobenzaldehyde (125.5 mg, 0.3 mmol), diethyl ether (1.0 mL) and $CHCl_3$ (5.0 mL) afforded compound **28** (68.6 mg, 90% yield) as a colorless oil. **^{1}H NMR** (600 MHz, $CDCl_3$) δ 3.66-3.61 (m, 1H), 3.55-3.51 (m, 1H), 3.44-3.39 (m, 1H), 2.91 (dd, *J* = 13.8 Hz, *J* = 6.6 Hz, 1H), 2.81 (dd, *J* = 13.8 Hz, *J* = 6.0 Hz, 1H), 1.17 (d, *J* = 6.0 Hz, 3H), 1.12 (d, *J* = 7.2 Hz, 3H); **^{13}C NMR** (151 MHz, $CDCl_3$) δ 146.29-144.48 (m), 140.68-138.77 (m), 138.29-136.40 (m), 112.31 (td, *J* = 19.0 Hz, *J* = 3.6 Hz), 73.95, 64.19, 29.28, 19.63, 15.35; **^{19}F NMR** (471 MHz, $CDCl_3$) δ (-142.54)−(-142.61) (m, 2F), (-157.36)−(-157.45) (m, 1F), (-163.09)−(-163.20) (m, 2F); **HRMS** (ESI) m/z calcd for $C_{11}H_{12}F_5O_2$ $[M+H]^+$ 255.0808, Found: 255.0812.

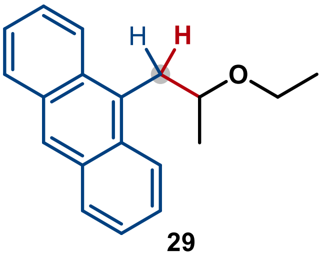

29

(**29**) According to the general procedure A, using *N*-triftosylhydrazone derived from anthracene-9-carbaldehyde (128.5 mg, 0.3 mmol), diethyl ether (5.0 mL) and $CHCl_3$ (5.0 mL) afforded compound **29** (28.6 mg, 36% yield) as a yellow oil. **^{1}H NMR** (600 MHz, $CDCl_3$) δ 8.36-8.33 (m, 3H), 7.99 (d, *J* = 9.0 Hz, 2H), 7.52-7.49 (m, 2H), 7.46-7.44 (m, 2H), 3.97-3.91 (m, 2H), 3.73-3.69 (m, 1H), 3.55-3.50 (m, 1H), 3.36-3.31 (m, 1H), 1.18 (d, *J* = 6.0 Hz, 3H), 1.07 (t, *J* = 7.2 Hz, 3H); **^{13}C NMR** (151 MHz, $CDCl_3$) δ 131.69, 131.57, 130.45, 129.09, 126.16, 125.37, 125.01, 124.77, 76.71, 64.41, 35.01, 20.40, 15.61; **HRMS** (ESI) m/z calcd for $C_{19}H_{21}O$ $[M+H]^+$ 265.1592, Found: 265.1602.

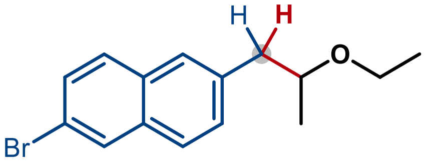

30

(**30**) According to the general procedure A, using *N*-triftosylhydrazone derived from 6-bromo-2-naphthaldehyde (137.2 mg, 0.3 mmol), diethyl ether (1.0 mL) and $CHCl_3$ (5.0 mL) afforded compound **30** (47.5 mg, 54% yield) as a colorless oil. **^{1}H NMR** (600 MHz, $CDCl_3$) δ 7.96 (d, *J* = 0.6 Hz, 1H), 7.67 (d, *J* = 8.4 Hz, 1H), 7.64 (d, *J* = 8.4 Hz, 1H), 7.61 (s, 1H), 7.51 (dd, *J* = 8.4 Hz, *J* = 1.8 Hz, 1H), 7.38 (dd, *J* = 8.4 Hz, *J* = 1.8 Hz, 1H), 3.71-3.66 (m, 1H), 3.57-3.52 (m, 1H), 3.44-3.39 (m, 1H), 3.03 (dd, *J* = 13.8 Hz, *J* = 6.6 Hz, 1H), 2.78 (dd, *J* = 13.8 Hz, *J* = 6.6 Hz, 1H), 1.16-1.14 (m, 6H); **^{13}C NMR** (151 MHz, $CDCl_3$) δ 137.43, 133.15, 131.91, 129.60, 129.17,

127.67, 126.71, 119.02, 76.22, 64.05, 43.25, 19.76, 15.55; **HRMS** (ESI) m/z calcd for $C_{15}H_{18}OBr$ $[M+H]^+$ 293.0541, Found: 293.0532.

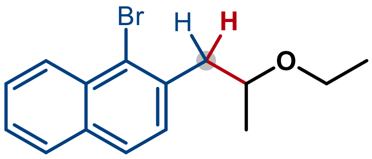

31

(**31**) According to the general procedure A, using *N*-triftosylhydrazone derived from 1-bromo-2-naphthaldehyde (137.2 mg, 0.3 mmol), diethyl ether (5.0 mL) and $CHCl_3$ (5.0 mL) afforded compound **31** (86.2 mg, 98% yield) as a colorless oil. **^{1}H NMR** (600 MHz, $CDCl_3$) δ 8.32 (d, *J* = 8.4 Hz, 1H), 7.80 (d, *J* = 8.4 Hz, 1H), 7.72 (d, *J* = 8.4 Hz, 1H), 7.57 (t, *J* = 8.4 Hz, 1H), 7.48 (t, *J* = 7.2 Hz, 1H), 7.39 (d, *J* = 8.4 Hz, 1H), 3.85-3.80 (m, 1H), 3.58-3.53 (m, 1H), 3.45-3.40 (m, 1H), 3.29 (dd, *J* = 13.2 Hz, *J* = 6.6 Hz, 1H), 3.04 (dd, *J* = 13.2 Hz, *J* = 6.6 Hz, 1H), 1.21 (d, *J* = 6.0 Hz, 3H), 1.13 (t, *J* = 7.2 Hz, 3H); **^{13}C NMR** (151 MHz, $CDCl_3$) δ 137.04, 133.34, 132.53, 129.54, 127.99, 127.33, 127.23, 127.10, 125.93, 124.23, 75.04, 64.18, 44.37, 20.06, 15.55; **HRMS** (ESI) m/z calcd for $C_{15}H_{18}BrO$ $[M+H]^+$ 293.0541, Found: 293.0532.

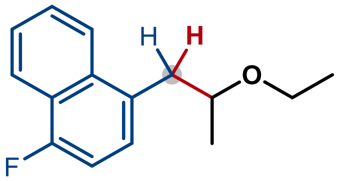

32

(**32**) According to the general procedure A, using *N*-triftosylhydrazone derived from 4-fluoro-1-naphthaldehyde (118.9 mg, 0.3 mmol), diethyl ether (5.0 mL) and $CHCl_3$ (5.0 mL) afforded compound **32** (36.9 mg, 53% yield) as a colorless oil. **^{1}H NMR** (600 MHz, $CDCl_3$) δ 8.13 (d, *J* = 7.8 Hz, 1H), 8.05 (d, *J* = 8.4 Hz, 1H), 7.58-7.52 (m, 2H), 7.27 (dd, *J* = 7.8 Hz, *J* = 6.0 Hz, 1H), 7.07 (dd, *J* = 10.2 Hz, *J* = 7.8 Hz, 1H), 3.78-3.73 (m, 1H), 3.57-3.52 (m, 1H), 3.39-3.37 (m, 2H), 2.99 (dd, *J* = 13.8 Hz, *J* = 6.6 Hz, 1H), 1.16 (d, *J* = 6.0 Hz, 3H), 1.13 (t, *J* = 7.2 Hz, 3H); **^{13}C NMR** (151 MHz, $CDCl_3$) δ 157.76 (d, *J* = 250.3 Hz), 133.31 (d, *J* = 3.9 Hz), 131.19 (d, *J* = 4.4 Hz), 127.08 (d, *J* = 8.1 Hz), 126.60, 125.66 (d, *J* = 1.9 Hz),124.08 (d, *J* = 2.3 Hz), 123.93 (d, *J* = 16.0 Hz), 121.13 (d, *J* = 5.8 Hz), 108.81 (d, *J* = 19.3 Hz), 75.68, 64.15, 39.99, 20.00, 15.56; **^{19}F NMR** (471 MHz, $CDCl_3$) δ (-125.48)−(-125.51) (m, 1F); **HRMS** (ESI) m/z calcd for $C_{15}H_{18}FO$ $[M+H]^+$ 233.1342, Found: 233.1338.

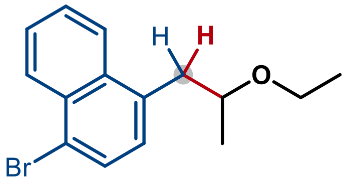

33

(**33**) According to the general procedure A, using *N*-triftosylhydrazone derived from 4-bromo-1-naphthaldehyde (137.2 mg, 0.3 mmol), diethyl ether (5.0 mL) and $CHCl_3$ (5.0 mL) afforded compound **33** (56.3 mg, 64% yield) as a colorless oil. **^{1}H NMR** (600 MHz, $CDCl_3$) δ 8.29-8.27 (m, 1H), 8.08-8.06 (m, 1H), 7.70 (d, *J* = 7.8 Hz, 1H), 7.60-7.55 (m, 2H), 7.21 (d, *J* = 7.8 Hz, 1H), 3.79-3.74 (m, 1H), 3.56-3.51 (m, 1H), 3.40-3.35 (m, 2H), 3.01 (dd, *J* = 13.8 Hz, *J* = 6.6 Hz, 1H), 1.17 (d, *J* = 6.0 Hz, 3H), 1.12 (d, *J* = 7.2 Hz, 3H); **^{13}C NMR** (151 MHz, $CDCl_3$) δ 135.61, 133.50, 132.09, 129.51, 128.03, 127.91, 126.81, 126.50, 124.49, 121.37, 75.54, 64.18, 40.30, 20.08, 15.55; **HRMS** (ESI) m/z calcd for $C_{15}H_{18}BrO$ $[M+H]^+$ 293.0541, Found: 293.0532.

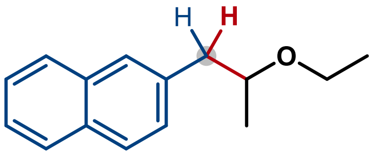

34

(**34**) According to the general procedure A, using *N*-triftosylhydrazone derived from 2-naphthaldehyde (113.5 mg, 0.3 mmol), diethyl ether (5.0 mL) and $CHCl_3$ (5.0 mL) afforded compound **34** (45.0 mg, 70% yield) as a colorless oil. **^{1}H NMR** (600 MHz, $CDCl_3$) δ 7.81-7.77 (m, 2H), 7.76 (d, *J* = 8.4 Hz, 1H), 7.64 (m, 1H), 7.46-7.40 (m, 2H), 7.36 (dd, *J* = 8.4 Hz, *J* = 1.8 Hz, 1H), 3.73-3.67 (m, 1H), 3.58-3.53 (m, 1H), 3.47-3.42 (m, 1H), 3.08 (dd, *J* = 13.2 Hz, *J* = 6.6 Hz, 1H), 2.78 (dd, *J* = 13.2 Hz, *J* = 6.6 Hz, 1H), 1.18-1.15 (m, 6H); **^{13}C NMR** (151 MHz, $CDCl_3$) δ 136.80, 133.53, 132.08, 128.09, 127.70, 127.62, 127.56, 127.46, 125.81, 125.17, 76.45, 64.01, 43.31, 19.75, 15.57; **HRMS** (ESI) m/z calcd for $C_{15}H_{19}O$ $[M+H]^+$ 215.1436, Found: 215.1431.

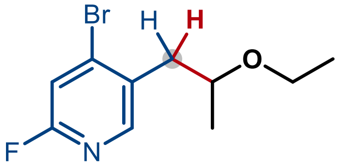

35

(**35**) According to the general procedure A, using *N*-triftosylhydrazone derived from 4-bromo-6-fluoronicotinaldehyde (127.9 mg, 0.3 mmol), diethyl ether (5.0 mL) and $CHCl_3$ (5.0 mL)

afforded compound **35** (51.1 mg, 65% yield) as a colorless oil. **^{1}H NMR** (600 MHz, $CDCl_3$) δ 8.12-8.11 (m, 1H), 7.80 (dd, *J* = 8.4 Hz, *J* = 2.4 Hz, 1H), 3.67-3.62 (m, 1H), 3.58-3.53 (m, 1H), 3.37-3.32 (m, 1H), 2.77-2.71 (m, 2H), 1.19 (d, *J* = 6.0 Hz, 3H), 1.13 (t, *J* = 7.2 Hz, 3H); **^{13}C NMR** (151 MHz, $CDCl_3$) δ 161.00 (d, *J* = 239.2 Hz), 145.97 (d, *J* = 15.7 Hz), 144.61 (d, *J* = 6.1 Hz), 123.09 (d, *J* = 33.1 Hz), 116.10 (d, *J* = 3.9 Hz), 74.08, 64.12, 35.63, 19.72, 15.37; **^{19}F NMR** (471 MHz, $CDCl_3$) δ -124.25 (d, *J* = 7.9 Hz, 1F); **HRMS** (ESI) m/z calcd for $C_{10}H_{14}NOBrF$ $[M+H]^+$ 262.0243, Found: 262.0246.

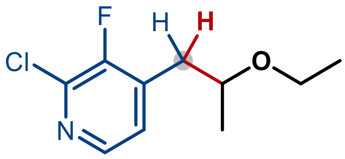

36

(**36**) According to the general procedure A, using *N*-triftosylhydrazone derived from 2-chloro-3-fluoroisonicotinaldehyde (114.5 mg, 0.3 mmol), diethyl ether (5.0 mL) and $CHCl_3$ (5.0 mL) afforded compound **36** (39.2 mg, 60% yield) as a yellow oil. **^{1}H NMR** (500 MHz, $CDCl_3$) δ 8.10 (d, *J* = 5.0 Hz, 1H), 7.17 (t, *J* = 5.0 Hz, 1H), 3.71-3.65 (m, 1H), 3.58-3.52 (m, 1H), 3.40-3.34 (m, 1H), 2.87 (dd, *J* = 14.0 Hz, *J* = 7.0 Hz, 1H), 2.82 (dd, *J* = 14.0 Hz, *J* = 5.5 Hz, 1H), 1.19 (d, *J* = 6.0 Hz, 3H), 1.13 (t, *J* = 7.0 Hz, 3H); **^{13}C NMR** (151 MHz, $CDCl_3$) δ 153.66 (d, *J* = 258.1 Hz), 143.84 (d, *J* = 6.9 Hz), 138.81 (d, *J* = 20.0 Hz), 137.16 (d, *J* = 14.7 Hz), 125.92, 74.01, 64.13, 35.62, 19.79, 15.36; **^{19}F NMR** (471 MHz, $CDCl_3$) δ -124.57 (d, *J* = 4.4 Hz, 1F); **HRMS** (ESI) m/z calcd for $C_{10}H_{14}NOFCl$ $[M+H]^+$ 218.0748, Found: 218.0752.

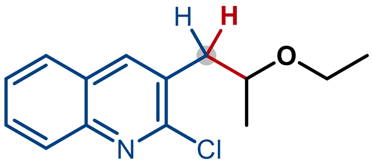

37

(**37**) According to the general procedure A, using *N*-triftosylhydrazone derived from 2-chloroquinoline-3-carbaldehyde (124.1 mg, 0.3 mmol), diethyl ether (5.0 mL) and $CHCl_3$ (5.0 mL) afforded compound **37** (38.9 mg, 52% yield) as a colorless oil. **^{1}H NMR** (500 MHz, $CDCl_3$) δ 8.05 (s, 1H), 8.00 (d, *J* = 8.0 Hz, 1H), 7.79 (d, *J* = 8.0 Hz, 1H), 7.71-7.68 (m, 1H), 7.56-7.53 (m, 1H), 3.85-3.79 (m, 1H), 3.60-3.54 (m, 1H), 3.40-3.33 (m, 1H), 3.11 (dd, *J* = 14.0 Hz, *J* = 7.0 Hz, 1H), 2.97 (dd, *J* = 14.0 Hz, *J* = 5.5 Hz, 1H), 1.25 (d, *J* = 6.5 Hz, 3H), 1.10 (t, *J* = 7.0 Hz, 3H); **^{13}C NMR** (126 MHz, $CDCl_3$) δ 151.49, 146.59, 139.45, 131.01, 129.79, 128.18, 127.41, 127.12, 126.95, 74.07, 64.20, 40.69, 19.99, 15.46; **HRMS** (ESI) m/z calcd for $C_{14}H_{17}NOCl$ $[M+H]^+$ 250.0999, Found: 250.1002.

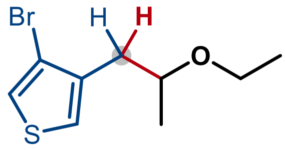

38

(**38**) According to the general procedure A, using *N*-triftosylhydrazone derived from 4-bromothiophene-3-carbaldehyde (123.9 mg, 0.3 mmol), diethyl ether (5.0 mL) and $CHCl_3$ (5.0 mL) afforded compound **38** (33.6 mg, 45% yield) as a yellow oil. **^{1}H NMR** (600 MHz, $CDCl_3$) δ 7.22 (d, *J* = 3.6 Hz, 1H), 7.08 (d, *J* = 3.6 Hz, 1H), 3.70-3.65 (m, 1H), 3.58-3.54 (m, 1H), 3.47-3.43 (m, 1H), 2.88 (dd, *J* = 14.4 Hz, *J* = 6.6 Hz, 1H), 2.68 (dd, *J* = 14.4 Hz, *J* = 6.6 Hz, 1H), 1.18-1.16 (m, 6H); **^{13}C NMR** (151 MHz, $CDCl_3$) δ 137.96, 122.66, 122.49, 112.97, 74.36, 64.01, 36.70, 19.75, 15.56; **HRMS** (ESI) m/z calcd for $C_9H_{14}OSBr$ $[M+H]^+$ 248.9949, Found: 248.9942.

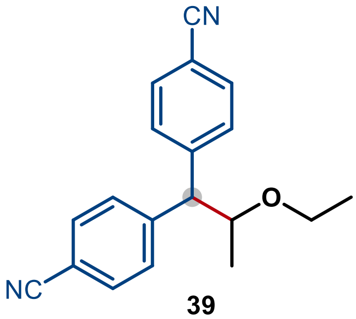

39

(**39**) According to the general procedure A, using *N*-triftosylhydrazone derived from 4,4'-carbonyldibenzonitrile (136.3 mg, 0.3 mmol), diethyl ether (5.0 mL) and $CHCl_3$ (5.0 mL) afforded compound **39** (33.9 mg, 39% yield) as a colorless oil. **^{1}H NMR** (500 MHz, $CDCl_3$) δ 7.61-7.58 (m, 4H), 7.44 (d, *J* = 8.5 Hz, 2H), 7.35 (d, *J* = 8.5 Hz, 2H), 4.10-4.05 (m, 1H), 3.99 (d, *J* = 7.0 Hz, 1H), 3.63-3.57 (m, 1H), 3.26-3.20 (m, 1H), 1.13 (d, *J* = 6.5 Hz, 3H), 1.06 (t, *J* = 7.0 Hz, 3H); **^{13}C NMR** (151 MHz, $CDCl_3$) δ 147.05, 146.31, 132.43, 132.10, 129.88, 129.31, 118.77, 118.56, 110.90, 110.68, 76.62, 64.62, 58.40, 18.35, 15.30; **HRMS** (ESI) m/z calcd for $C_{19}H_{19}N_2O$ $[M+H]^+$ 291.1497, Found: 291.1499.

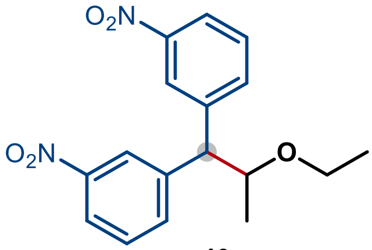

40

(**40**) According to the general procedure A, using *N*-triftosylhydrazone derived from bis(3-nitrophenyl)-methanone (148.3 mg, 0.3 mmol), diethyl ether (5.0 mL) and $CHCl_3$ (5.0 mL) afforded compound **40** (57.5 mg, 58% yield) as a colorless oil. **^{1}H NMR** (600 MHz, $CDCl_3$) δ 8.25 (t, *J* = 1.8 Hz, 1H), 8.19 (t, *J* = 1.8 Hz, 1H), 8.12-8.09 (m, 2H), 7.70 (d, *J* = 7.8 Hz, 1H), 7.60 (d, *J* = 7.8 Hz, 1H), 7.49 (t, *J* = 7.8 Hz, 2H), 4.16-4.13 (m, 1H), 4.11 (d, *J* = 6.6 Hz, 1H), 3.68-3.63 (m, 1H), 3.32-3.27 (m, 1H), 1.17 (d, *J* = 6.0 Hz, 3H), 1.11 (t, *J* = 7.2 Hz, 3H); **^{13}C NMR** (151 MHz, $CDCl_3$) δ 148.46, 148.25, 143.87, 142.77, 135.51, 134.76, 129.61, 129.23, 124.06, 123.34, 122.05, 121.92, 76.62, 64.66, 57.38, 18.31, 15.30; **HRMS** (ESI) m/z calcd for $C_{17}H_{19}N_2O_5$ $[M+H]^+$ 331.1294, Found: 331.1299.

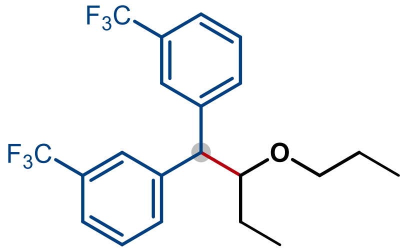

41

(**41**) According to the general procedure A, using *N*-triftosylhydrazone derived from *bis*(3-(trifluoromethyl)phenyl)methanone (162.1 mg, 0.3 mmol), 1-propoxypropane (1.0 mL) and $CHCl_3$ (5.0 mL) afforded compound **41** (55.8 mg, 46% yield) as a colourless oil. **^{1}H NMR** (600 MHz, $CDCl_3$) δ 7.67 (s, 1H), 7.58 (d, *J* = 7.8 Hz, 1H), 7.54 (s, 1H), 7.48-7.44 (m, 3H), 7.40 (t, *J* = 7.8 Hz, 2H), 4.13 (d, *J* = 7.2 Hz, 1H), 3.85 (dt, *J* = 7.2 Hz, *J* = 5.4 Hz, 1H), 3.42 (dt, *J* = 9.0 Hz, *J* = 6.0 Hz, 1H), 2.98 (dt, *J* = 9.0 Hz, *J* = 6.0 Hz, 1H), 1.53-1.45 (m, 2H), 1.43-1.37 (m, 2H), 0.92 (t, *J* = 7.2 Hz, 3H), 0.73 (t, *J* = 7.2 Hz, 3H); **^{13}C NMR** (151 MHz, $CDCl_3$) δ 143.19, 142.24, 132.61, 131.95, 130.86 (q, *J* = 32.3 Hz), 130.51 (q, *J* = 32.0 Hz), 128.97, 128.64, 126.07 (q, *J* = 4.0 Hz), 125.18 (q, *J* = 4.2 Hz), 124.20 (q, *J* = 272.9 Hz), 124.08 (q, *J* = 272.4 Hz), 123.51 (q, *J* = 3.9 Hz), 123.40 (q, *J* = 4.1 Hz), 82.97, 72.17, 55.02, 24.94, 23.13, 10.53, 9.31; **^{19}F NMR** (471 MHz, $CDCl_3$) δ -62.56 (s, 6F). **HRMS** (ESI) m/z calcd for $C_{21}H_{23}OF_6$ $[M+H]^+$ 405.1653, Found: 405.1647.

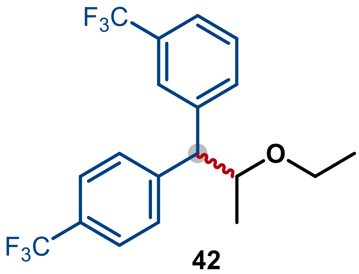

42

(**42**) According to the general procedure A, using *N*-triftosylhydrazone derived from (3-(trifluoromethyl)-phenyl)(4-(trifluoromethyl)phenyl)methanone (162.1 mg, 0.3 mmol), diethyl ether (5.0 mL) and $CHCl_3$ (5.0 mL) afforded compound **42** (70.0 mg, 62% yield) as a colorless oil.

The d.r. was 1:1 from ^{1}H NMR analysis of crude residue and isolated product by chromatography. **^{1}H NMR** (600 MHz, $CDCl_3$) δ 7.61 (s, 1H), 7.56-7.53 (m, 5H), 7.50 (d, *J* = 7.8 Hz, 1H), 7.48-7.45 (m, 4H), 7.43-7.36 (m, 5H), 4.12-4.07 (m, 2H), 4.01-3.99 (m, 2H), 3.63-3.58 (m, 2H), 3.27-3.22 (m, 2H), 1.14-1.12 (m, 6H), 1.08-1.04 (m, 6H); **^{13}C NMR** (151 MHz, $CDCl_3$) δ 146.18, 145.47, 143.22, 142.42, 132.37, 131.98, 130.86 (q, *J* = 31.7 Hz), 130.52 (q, *J* = 32.2 Hz), 129.35, 128.99, 128.88, 128.62, 125.66 (q, *J* = 3.6 Hz), 125.54 (q, *J* = 3.6 Hz), 125.15 (q, *J* = 3.9 Hz, 2C), 124.10 (q, *J* = 272.0 Hz), 124.07 (q, *J* = 272.4 Hz), 123.58 (q, *J* = 3.5 Hz), 123.35 (q, *J* = 3.4 Hz), 64.64, 64.61, 58.48 (2C), 58.10, 58.03, 18.44, 18.42, 15.30, 15.26; **^{19}F NMR** (471 MHz, $CDCl_3$) δ -62.42 (s, 3F), -62.50 (s, 3F), -62.55 (s, 3F), -62.56 (s, 3F). **HRMS** (ESI) m/z calcd for $C_{21}H_{23}OF_6$ $[M+H]^+$ 405.1653, Found: 405.1647.

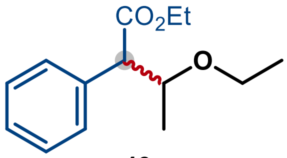

43

(**43**) According to the general procedure A, using *N*-triftosylhydrazone derived from methyl 2-oxo-2-phenylacetate (120.1 mg, 0.3 mmol), diethyl ether (1.0 mL) and $CHCl_3$ (5.0 mL) afforded compound **43** (49.6 mg, 70% yield) as a colorless oil. The d.r. was 1:1 from ^{1}H NMR analysis of crude residue and isolated product by chromatography. (**43-major**) **^{1}H NMR** (600 MHz, $CDCl_3$) δ 7.39 (d, *J* = 7.2 Hz, 2H), 7.30 (t, *J* = 7.2 Hz, 2H), 7.27-7.25 (m, 1H), 4.20-4.06 (m, 2H), 4.05-3.94 (m, 1H), 3.59 (d, *J* = 9.0 Hz, 1H), 3.49-3.41 (m, 1H), 3.15-3.10 (m, 1H), 1.24-1.21 (m, 6H), 0.95-0.93 (m, 3H); **^{13}C NMR** (151 MHz, $CDCl_3$) δ 172.94, 136.67, 128.92, 128.59, 127.13, 76.72, 64.97, 60.56, 59.52, 18.64, 15.21, 14.12; (**43-minor**) **^{1}H NMR** (600 MHz, $CDCl_3$) δ 7.35 (d, *J* = 7.2 Hz, 2H), 7.30 (t, *J* = 7.2 Hz, 2H), 7.27-7.25 (m, 1H), 4.20-4.06 (m, 2H), 4.05-3.94 (m, 1H), 3.69-3.64 (m, 1H), 3.51 (d, *J* = 10.2 Hz, 1H), 3.49-3.41 (m, 1H), 1.24-1.21 (m, 3H), 1.18 (t, *J* = 7.2 Hz, 3H), 0.95-0.93 (m, 3H); **^{13}C NMR** (151 MHz, $CDCl_3$) δ 172.31, 136.11, 128.56, 128.10, 127.54, 77.38, 64.77, 60.64, 58.52, 17.14, 15.50, 14.07; **HRMS** (ESI) m/z calcd for $C_{14}H_{21}O_3$ $[M+H]^+$ 237.1491, Found: 237.1486.

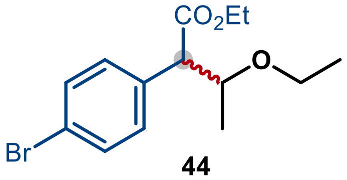

44

(**44**) According to the general procedure A, using *N*-triftosylhydrazone derived from ethyl 2-(4-bromophenyl)-2-oxoacetate (143.8 mg, 0.3 mmol), diethyl ether (1.0 mL) and $CHCl_3$ (5.0 mL) afforded compound **44** (73.8 mg, 78% yield) as a colorless oil. The d.r. was 1.2:1 from ^{1}H- NMR analysis of crude residue and 1.4:1 from isolated product by chromatography. (**44-major**) **^{1}H NMR** (600 MHz, $CDCl_3$) δ 7.45-7.42 (m, 2H), 7.28-7.26 (m, 2H), 4.21-4.13 (m, 1H), 4.12-4.06

(m, 1H), 4.00-3.93 (m, 1H), 3.53 (d, J = 8.4 Hz, 1H), 3.49-3.43 (m, 1H), 3.17-3.12 (m, 1H), 1.24-1.16 (m, 6H), 0.97 (t, J = 7.2 Hz, 3H); **^{13}C NMR** (151 MHz, $CDCl_3$) δ 171.88, 135.64, 131.17, 130.77, 121.19, 76.37, 64.92, 60.84, 57.95, 18.49, 15.22, 14.06; (**44-minor**) **^{1}H NMR** (600 MHz, $CDCl_3$) δ 7.45-7.42 (m, 2H), 7.23 (d, J = 7.8 Hz, 2H), 4.21-4.13 (m, 1H), 4.12-4.06 (m, 1H), 4.00-3.93 (m, 1H), 3.69-3.63 (m, 1H), 3.49-3.43 (m, 2H), 1.24-1.16 (m, 6H), 0.93 (d, J = 6.0 Hz, 3H); **^{13}C NMR** (151 MHz, $CDCl_3$) δ 172.51, 135.08, 131.73, 130.27, 121.64, 77.16, 64.81, 60.75, 58.83, 17.10, 15.45, 14.11; **HRMS** (ESI) m/z calcd for $C_{14}H_{20}BrO_3$ $[M+H]^+$ 315.0596, Found: 315.0589.

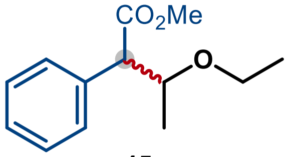

45

(**45**) According to the general procedure A, using *N*-triftosylhydrazone derived from methyl 2-oxo-2-phenylacetate (115.9 mg, 0.3 mmol), diethyl ether (1.0 mL) and $CHCl_3$ (5.0 mL) afforded compound **45** (54.7 mg, 82% yield) as a colorless oil. The d.r. was 1.2:1 from ^{1}H- NMR analysis of crude residue and 1.3:1 from isolated product by chromatography. (**45-major**) **^{1}H NMR** (500 MHz, $CDCl_3$) δ 7.38 (d, J = 7.0 Hz, 2H), 7.35-7.25 (m, 3H), 4.00-3.94 (m, 1H), 3.66 (s, 3H), 3.61 (d, J = 8.5 Hz, 1H), 3.50-3.41 (m, 1H), 3.15-3.09 (m, 1H), 1.22 (d, J = 6.0 Hz, 3H), 0.94 (t, J = 7.0 Hz, 3H); **^{13}C NMR** (151 MHz, $CDCl_3$) δ 172.82, 136.53, 128.91, 128.16, 127.22, 76.65, 65.01, 58.34, 51.88, 18.66, 15.22; (**45-minor**) **^{1}H NMR** (500 MHz, $CDCl_3$) δ 7.35-7.25 (m, 5H), 4.06-4.01 (m, 1H), 3.70-3.64 (m, 1H), 3.67 (s, 3H), 3.54 (d, J = 10.0 Hz, 1H), 3.50-3.41 (m, 1H), 1.18 (t, J = 7.0 Hz, 3H), 0.94 (d, J = 6.0 Hz, 3H); **^{13}C NMR** (151 MHz, $CDCl_3$) δ 173.45, 135.95, 128.65, 128.55, 127.63, 77.27, 64.78, 59.31, 51.80, 17.16, 15.53; **HRMS** (ESI) m/z calcd for $C_{13}H_{19}O_3$ $[M+H]^+$ 223.1334, Found: 133.1324.

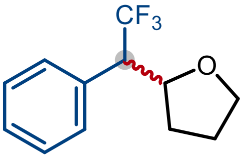

46

(**46**) According to the general procedure A, using *N*-triftosylhydrazone derived from 2,2,2-trifluoro-1-phenylethan-1-one (118.9 mg, 0.3 mmol), THF (1.0 mL) and $CHCl_3$ (5.0 mL) afforded compound **46** (55.2 mg, 80% yield) as a colorless oil. The d.r. was 1.5:1 from ^{1}H- NMR analysis of crude residue and 1.7:1 from isolated product by chromatography. (**46-major**) **^{1}H NMR** (600 MHz, $CDCl_3$) δ 7.39-7.34 (m, 5H), 4.44-4.39 (m, 1H), 3.75-3.68 (m, 2H), 3.38-3.27 (m, 1H), 2.15-2.09 (m, 1H), 1.87-1.60 (m, 3H); **^{13}C NMR** (151 MHz, $CDCl_3$) δ 132.77, 130.07, 128.39, 128.18, 125.90 (q, J = 280.5 Hz), 76.66 (q, J = 1.8 Hz), 68.38, 54.55 (q, J = 24.5 Hz),

30.42, 25.57; **^{19}F NMR** (471 MHz, CDCl3) δ -65.46 (d, J = 8.4 Hz, 3F), -66.15 (d, J = 8.4 Hz, 5F); (**46'-minor**) **^{1}H NMR** (600 MHz, $CDCl_3$) δ 7.39-7.34 (m, 3H), 7.28-7.26 (m, 2H), 4.44-4.39 (m, 1H), 3.92-3.89 (m, 1H), 3.87-3.83 (m, 1H), 3.38-3.27 (m, 1H), 1.87-1.60 (m, 3H), 1.46-1.40 (m, 1H); **^{13}C NMR** (151 MHz, $CDCl_3$) δ 133.48, 129.14, 128.68, 128.21, 126.57 (q, J = 280.9 Hz), 77.67, 68.65, 54.87 (q, J = 24.5 Hz), 30.64, 25.02; **^{19}F NMR** (471 MHz, $CDCl_3$) δ -65.46 (d, J = 8.4 Hz, 3F); **HRMS** (ESI) m/z calcd for $C_{12}H_{14}OF_3$ $[M+H]^+$ 231.0997, Found: 231.1000.

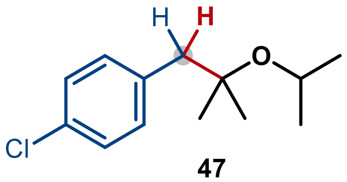

47

(**47**) According to the general procedure A, using N-triftosylhydrazone derived from 4-chlorobenzaldehyde (108.8 mg, 0.3 mmol), 2-isopropoxypropane (1.0 mL) and $CHCl_3$ (5.0 mL) afforded **47** (64.6 mg, 95% yield) as a colorless oil. **^{1}H NMR** (600 MHz, $CDCl_3$) δ 7.22 (d, J = 8.4 Hz, 2H), 7.16 (d, J = 8.4 Hz, 2H), 3.87-3.80 (m, 1H), 2.71 (s, 2H), 1.11 (d, J = 6.6 Hz, 6H), 1.10 (s, 6H); **^{13}C NMR** (151 MHz, $CDCl_3$) δ 137.22, 131.90, 131.83, 127.75, 75.44, 63.46, 48.19, 25.51, 25.09; **HRMS** (ESI) m/z calcd for $C_{13}H_{20}OCl$ $[M+H]^+$ 227.1203, Found: 227.1201.

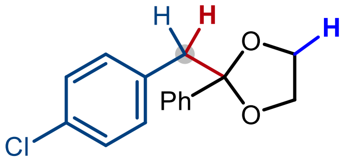

48

(**48**) According to the general procedure A, using N-triftosylhydrazone derived from 4-chlorobenzaldehyde (108.8 mg, 0.3 mmol), 2-phenyl-1,3-dioxolane (90.1 mg, 0.6 mmol, 2.0 equiv) and $CHCl_3$ (5.0 mL) afforded compound **48** (53.6 mg, 65% yield) as a colorless oil. **^{1}H NMR** (600 MHz, $CDCl_3$) δ 7.38-7.35 (m, 2H), 7.31-7.25 (m, 3H), 7.17 (d, J = 8.4 Hz, 2H), 7.05 (d, J = 8.4 Hz, 2H), 3.84-3.80 (m, 2H), 3.75-3.71 (m, 2H), 3.12 (s, 2H); **^{13}C NMR** (151 MHz, $CDCl_3$) δ 142.15, 134.41, 132.24, 132.11, 127.96, 127.89, 127.73, 125.75, 109.64, 64.70, 46.29; **HRMS** (ESI) m/z calcd for $C_{16}H_{16}O_2Cl$ $[M+H]^+$ 275.0839, Found: 275.0846.

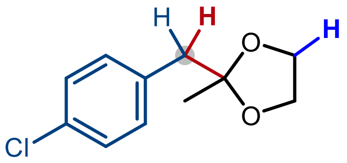

49

(**49**) According to the general procedure A, using *N*-triftosylhydrazone derived from 4-chlorobenzaldehyde (108.8 mg, 0.3 mmol), 2-methyl-1,3-dioxolane (52.9 mg, 0.6 mmol, 2.0 equiv) and $CHCl_3$ (5.0 mL) afforded compound **49** (61.2 mg, 96% yield) as a colorless oil. **^{1}H NMR** (600 MHz, $CDCl_3$) δ 7.24 (d, *J* = 8.4 Hz, 2H), 7.20 (d, *J* = 8.4 Hz, 2H), 3.91-3.86 (m, 2H), 3.74-3.69 (m, 2H), 2.89 (s, 2H), 1.30 (s, 3H); **^{13}C NMR** (151 MHz, $CDCl_3$) δ 135.33, 132.29, 131.80, 128.01, 109.43, 64.84, 44.66, 24.37; **HRMS** (ESI) m/z calcd for $C_{11}H_{14}$ O_2Cl $[M+H]^+$ 213.0682, Found: 213.0683.

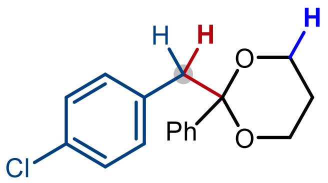

50

(**50**) According to the general procedure A, using *N*-triftosylhydrazone derived from 4-chlorobenzaldehyde (108.8 mg, 0.3 mmol) and 2-phenyl-1,3-dioxane (98.5 mg, 0.6 mmol, 2.0 equiv) and $CHCl_3$ (5.0 mL) afforded compound **50** (43.3 mg, 50% yield) as a white solid, m.p. 59-61 ^{o}C. **^{1}H NMR** (600 MHz, $CDCl_3$) δ 7.31-7.25 (m, 3H), 7.16-7.14 (m, 2H), 7.08 (d, *J* = 8.4 Hz, 2H), 6.82 (d, *J* = 8.4 Hz, 2H), 3.87-3.84 (m, 2H), 3.80-3.76 (m, 2H), 2.96 (s, 2H), 2.12-2.04 (m, 1H), 1.21-1.19 (m, 1H); **^{13}C NMR** (151 MHz, $CDCl_3$) δ 138.72, 134.27, 132.30, 131.99, 128.25, 127.80, 127.71, 127.36, 101.68, 61.15, 50.31, 25.50; **HRMS** (ESI) m/z calcd for $C_{17}H_{18}O_2Cl$ $[M+H]^+$ 289.0995, Found: 289.0996.

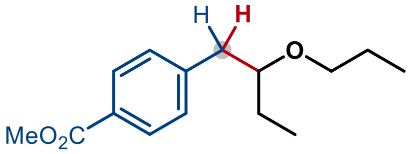

51

(**51**) According to the general procedure A, using *N*-triftosylhydrazone derived from methyl 4-formylbenzoate (115.9 mg, 0.3 mmol), 1-propoxypropane (1.0 mL) and $CHCl_3$ (5.0 mL) afforded compound **51** (67.6 mg, 90% yield) as a colorless oil. **^{1}H NMR** (500 MHz, $CDCl_3$) δ 7.95 (d, *J* = 8.0 Hz, 2H), 7.28 (d, *J* = 8.0 Hz, 2H), 3.90 (s, 3H), 3.40-3.33 (m, 2H), 3.29-3.25 (m, 1H), 2.84 (dd, *J* = 13.5 Hz, *J* = 7.0 Hz, 1H), 2.77 (dd, *J* = 13.5 Hz, *J* = 5.5 Hz, 1H), 1.54-1.46 (m, 4H), 0.94 (t, *J* = 7.5 Hz, 3H), 0.85 (t, *J* = 7.5 Hz, 3H); **^{13}C NMR** (151 MHz, $CDCl_3$) δ 167.17, 145.11, 129.49, 129.42, 127.90, 81.68, 71.32, 51.92, 40.54, 26.80, 23.23, 10.64, 9.68; **HRMS** (ESI) m/z calcd for $C_{15}H_{23}O_3$ $[M+H]^+$ 251.1647, Found:251.1654.

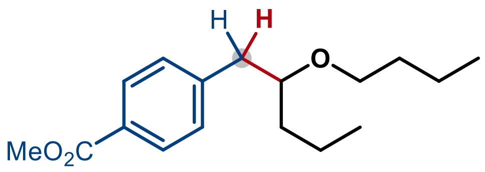

52

(**52**) According to the general procedure A, using *N*-triftosylhydrazone derived from methyl 4-formylbenzoate (115.9 mg, 0.3 mmol), 1-butoxybutane (1.0 mL) and $CHCl_3$ (5.0 mL) afforded compound **52** (80.2 mg, 96% yield) as a colorless oil. **^{1}H NMR** (600 MHz, $CDCl_3$) δ 7.95 (d, *J* = 8.4 Hz, 2H), 7.27 (d, *J* = 8.4 Hz, 2H), 3.90 (s, 3H), 3.45-3.41 (m, 1H), 3.39-3.31 (m, 2H), 2.84 (dd, *J* = 13.8 Hz, *J* = 6.6 Hz, 1H), 2.76 (dd, *J* = 13.8 Hz, *J* = 6.0 Hz, 1H), 1.49-1.26 (m, 8H), 0.89 (t, *J* = 7.2 Hz, 3H), 0.86 (t, *J* = 7.2 Hz, 3H); **^{13}C NMR** (151 MHz, $CDCl_3$) δ 167.17, 145.07, 129.49, 129.43, 127.91, 80.32, 69.36, 51.92, 40.95, 36.49, 32.17, 19.32, 18.71, 14.10, 13.83; **HRMS** (ESI) m/z calcd for $C_{17}H_{27}O_3$ $[M+H]^+$ 279.1960, Found: 279.1954.

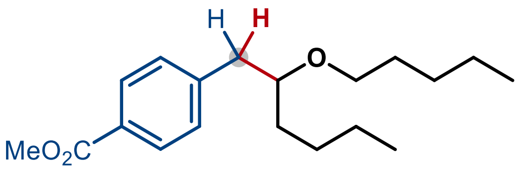

53

(**53**) According to the general procedure A, using *N*-triftosylhydrazone derived from methyl 4-formylbenzoate (115.9 mg, 0.3 mmol), 1-(pentyloxy)pentane (1.0 mL) and $CHCl_3$ (5.0 mL) afforded compound **53** (75.4 mg, 82% yield) as a colorless oil. **^{1}H NMR** (600 MHz, $CDCl_3$) δ 7.95 (d, *J* = 8.4 Hz, 2H), 7.27 (d, *J* = 8.4 Hz, 2H), 3.90 (s, 3H), 3.44-3.40 (m, 1H), 3.38-3.35 (m, 1H), 3.32-3.29 (m, 1H), 2.84 (dd, *J* = 13.8 Hz, *J* = 6.6 Hz, 1H), 2.77 (dd, *J* = 13.8 Hz, *J* = 5.4 Hz, 1H), 1.50-1.39 (m, 5H), 1.32-1.20 (m, 7H), 0.89-0.85 (m, 6H); **^{13}C NMR** (151 MHz, $CDCl_3$) δ 167.22, 145.16, 129.53, 129.47, 127.94, 80.59, 69.70, 51.97, 41.00, 33.96, 29.78, 28.35, 27.69, 22.77, 22.49, 14.07, 14.01; **HRMS** (ESI) m/z calcd for $C_{19}H_{31}O_3$ $[M+H]^+$ 307.2273, Found: 307.2282.

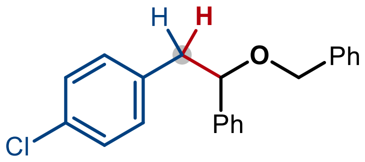

54

(**54**) According to the general procedure A, using *N*-triftosylhydrazone derived from 4-chlorobenzaldehyde (108.8 mg, 0.3 mmol), dibenzyl ether (118.9 mg, 0.6 mmol, 2.0 equiv) and $CHCl_3$ (5.0 mL) afforded compound **54** (78.4 mg, 81% yield) as a white solid, m.p. 55-57 °C. ^{1}**H NMR** (600 MHz, $CDCl_3$) δ 7.36-7.33 (m, 2H), 7.31-7.24 (m, 6H), 7.19 (d, *J* = 8.4 Hz, 2H), 7.15-7.13 (m, 2H), 7.03 (d, *J* = 8.4 Hz, 2H), 4.47-4.44 (m, 2H), 4.20 (d, *J* = 12.0 Hz, 1H), 3.10 (dd, *J* = 13.8 Hz, *J* = 7.8 Hz, 1H), 2.89 (dd, *J* = 13.8 Hz, *J* = 5.4 Hz, 1H); ^{13}**C NMR** (151 MHz, $CDCl_3$) δ 141.49, 138.29, 136.97, 131.95, 130.99, 128.44, 128.26, 128.08, 127.79, 127.56, 127.43, 126.84, 82.23, 70.45, 44.27; **HRMS** (ESI) m/z calcd for $C_{21}H_{20}Ocl$ $[M+H]^+$ 323.1203, Found: 323.1196.

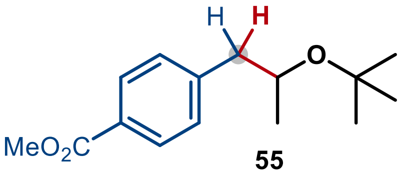

55

(**55**) According to the general procedure A, using *N*-triftosylhydrazone derived from 4-chlorobenzaldehyde (108.8 mg, 0.3 mmol), *tert*-butyl ethyl ether (1.0 mL) and $CHCl_3$ (5.0 mL) afforded compound **55** (54.1 mg, 72% yield) as a colorless oil. ^{1}**H NMR** (600 MHz, $CDCl_3$) δ 7.95 (d, *J* = 7.8 Hz, 2H), 7.26 (d, *J* = 7.8 Hz, 2H), 3.90 (s, 3H), 3.81-3.76 (m, 1H), 2.79 (dd, *J* = 13.2 Hz, *J* = 6.6 Hz, 1H), 2.68 (dd, *J* = 13.2 Hz, *J* = 6.0 Hz, 1H), 1.13 (d, *J* = 6.0 Hz, 3H), 1.05 (s, 9H); ^{13}**C NMR** (151 MHz, $CDCl_3$) δ 167.17, 145.31, 129.76, 129.34, 127.91, 73.47, 68.50, 51.91, 45.52, 28.25, 23.02; **HRMS** (ESI) m/z calcd for $C_{15}H_{23}O_3$ $[M+H]^+$ 251.1647, Found: 215.1654.

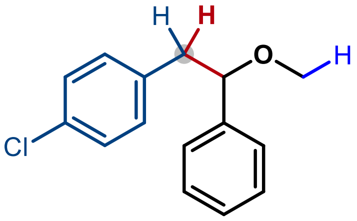

56

(**56**) According to the general procedure A, using *N*-triftosylhydrazone derived from 4-chlorobenzaldehyde (108.8 mg, 0.3 mmol), methyl benzyl ether (73.3 mg, 2.0 equiv) and $CHCl_3$ (5.0 mL) afforded compound **56** (59.2 mg, 80% yield) as a colorless oil. ^{1}**H NMR** (600 MHz, $CDCl_3$) δ 7.34-7.31 (m, 2H), 7.29-7.26 (m, 1H), 7.21-7.18 (m, 4H), 7.01 (d, *J* = 8.4 Hz, 2H), 4.27 (dd, *J* = 7.2 Hz, *J* = 6.0 Hz, 1H), 3.18 (s, 3H), 3.06 (dd, *J* = 13.8 Hz, *J* = 7.2 Hz, 1H), 2.86 (dd, *J* = 13.8 Hz, *J* = 6.0 Hz, 1H); ^{13}**C NMR** (151 MHz, $CDCl_3$) δ 141.25, 136.90, 131.95, 130.82, 128.35, 128.15, 127.72, 126.75, 84.80, 56.76, 44.06; **HRMS** (ESI) m/z calcd for $C_{15}H_{16}OCl$ $[M+H]^+$ 247.0890, Found: 247.0894.

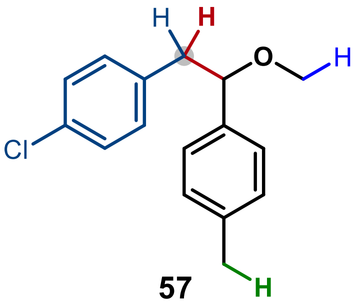

57

(**57**) According to the general procedure A, using *N*-triftosylhydrazone derived from 4-chlorobenzaldehyde (108.8 mg, 0.3 mmol), 1-(methoxymethyl)-4-methylbenzene (81.7 mg, 2.0 equiv) and $CHCl_3$ (5.0 mL) afforded compound **57** (54.8 mg, 70% yield) as a colorless oil.; **^{1}H NMR** (600 MHz, $CDCl_3$) δ 7.19 (d, *J* = 8.4 Hz, 2H), 7.13 (d, *J* = 7.8 Hz, 2H), 7.10 (d, *J* = 7.2 Hz, 2H), 7.03 (d, *J* = 8.4 Hz, 2H), 4.24 (dd, *J* = 7.2 Hz, *J* = 6.0 Hz, 1H), 3.16 (s, 3H), 3.05 (dd, *J* = 13.8 Hz, *J* = 7.2 Hz, 1H), 2.84 (dd, *J* = 13.8 Hz, *J* = 6.0 Hz, 1H), 2.34 (s, 3H); **^{13}C NMR** (151 MHz, $CDCl_3$) δ 138.20, 137.37, 137.06, 131.89, 130.81, 129.05, 128.13, 126.70, 84.61, 56.63, 44.05, 21.14; **HRMS** (ESI) m/z calcd for $C_{16}H_{18}OCl$ $[M+H]^+$ 261.1046, Found: 261.1042.

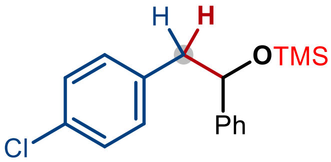

58

(**58**) According to the general procedure A, using *N*-triftosylhydrazone derived from 4-chlorobenzaldehyde (108.8 mg, 0.3 mmol), (benzyloxy)trimethylsilane (108.2 mg, 2.0 equiv) and $CHCl_3$ (5.0 mL) afforded compound **58** (54.9 mg, 60% yield) as a colorless oil. **^{1}H NMR** (600 MHz, $CDCl_3$) δ 7.54-7.46 (m, 5H), 7.45 (d, *J* = 8.4 Hz, 2H), 7.29 (d, *J* = 8.4 Hz, 2H), 4.96 (dd, *J* = 7.8 Hz, *J* = 6.0 Hz, 1H), 3.14-3.08 (m, 2H), 0.10 (s, 9H); **^{13}C NMR** (151 MHz, $CDCl_3$) δ 144.53, 137.42, 131.91, 131.14, 128.10, 128.01, 127.15, 125.82, 76.10, 46.74, -0.22; **HRMS** (ESI) m/z calcd for $C_{17}H_{22}OClSi$ $[M+H]^+$ 305.1128, Found: 305.1131.

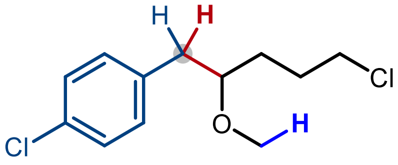

59

(**59**) According to the general procedure A, using *N*-triftosylhydrazone derived from 4-chlorobenzaldehyde (108.8 mg, 0.3 mmol), 1-chloro-4-methoxybutane (1.0 mL) and $CHCl_3$ (5.0 mL) afforded compound **59** (38.6 mg, 52% yield, 9:1 r.r.) as a yellow oil. **^{1}H NMR** (500 MHz, $CDCl_3$) δ 7.26 (d, *J* = 8.0 Hz, 2H), 7.13 (d, *J* = 8.0 Hz, 2H), 3.54-3.51 (m, 2H), 3.39-3.34 (m, 1H), 3.32 (s, 3H), 2.82 (dd, *J* = 14.0 Hz, *J* = 6.5 Hz, 1H), 2.69 (dd, *J* = 14.0 Hz, *J* = 6.0 Hz, 1H), 1.97-1.88 (m, 1H), 1.84-1.77 (m, 1H), 1.66-1.59 (m, 1H), 1.55-1.47 (m, 1H); **^{13}C NMR** (151 MHz, $CDCl_3$) δ 137.08, 132.02, 130.72, 128.42, 81.33, 57.08, 45.12, 39.32, 30.80, 28.48; **HRMS** (ESI) m/z calcd for $C_{12}H_{17}OCl_2$ $[M+H]^+$ 247.0656, Found: 247.0663.

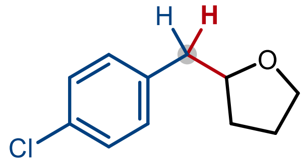

60

(**60**) According to the general procedure A, using *N*-triftosylhydrazone derived from 4-chlorobenzaldehyde (108.8 mg, 0.3 mmol), tetrahydrofuran (1.0 mL) and $CHCl_3$ (5.0 mL) afforded compound **60** (56.1 mg, 95% yield) as a colorless oil. **^{1}H NMR** (600 MHz, $CDCl_3$) δ 7.25 (d, *J* = 7.8 Hz, 2H), 7.16 (d, *J* = 7.8 Hz, 2H), 4.05-4.01 (m, 1H), 3.89-3.86 (m, 1H), 3.75-3.71 (m, 1H), 2.84 (dd, *J* = 13.8 Hz, *J* = 6.6 Hz, 1H), 2.74 (dd, *J* = 13.8 Hz, *J* = 6.0 Hz, 1H), 1.95-1.90 (m, 1H), 1.89-1.82 (m, 2H), 1.56-1.50 (m, 1H); **^{13}C NMR** (151 MHz, $CDCl_3$) δ 137.45, 131.94, 130.55, 128.37, 79.69, 67.95, 41.16, 30.94, 25.58; **HRMS** (ESI) m/z calcd for $C_{11}H_{14}OCl$ $[M+H]^+$ 197.0733, Found: 197.0742.

Spectroscopic data are in agreement with those reported in the literature.[4]

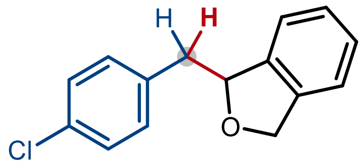

61

(**61**) According to the general procedure A, using *N*-triftosylhydrazone derived from 4-chlorobenzaldehyde (108.8 mg, 0.3 mmol), 1,3-dihydroisobenzofuran (108.2 mg, 2.0 equiv) and $CHCl_3$ (5.0 mL) afforded compound **61** (58.7 mg, 80% yield) as a white solid, m.p. 55-57 °C; **^{1}H NMR** (600 MHz, $CDCl_3$) δ 7.27-7.21 (m, 4H), 7.18-7.16 (m, 1H), 7.14 (d, *J* = 8.4 Hz, 2H), 7.04 (d, *J* = 7.2 Hz, 1H), 5.48-5.44 (m, 1H), 5.03-4.98 (m, 2H), 3.09 (dd, *J* = 13.8 Hz, *J* = 4.8 Hz, 1H), 3.00 (dd, *J* = 13.8 Hz, *J* = 7.2 Hz, 1H); **^{13}C NMR** (151 MHz, $CDCl_3$) δ 141.00, 139.40, 136.16,

132.17, 130.97, 128.28, 127.62, 127.11, 121.38, 121.01, 84.20, 72.63, 42.00; **HRMS** (ESI) m/z calcd for $C_{15}H_{14}OCl$ $[M+H]^+$ 245.0733, Found: 245.0731.

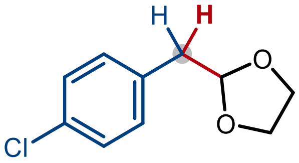

62

(**62**) According to the general procedure A, using *N*-triftosylhydrazone derived from 4-chlorobenzaldehyde (108.8 mg, 0.3 mmol), 1,3-dioxolane (5.0 mL) and $CHCl_3$ (5.0 mL) afforded compound **62** (32.8 mg, 55% yield) as a colorless oil. **^{1}H NMR** (600 MHz, $CDCl_3$) δ 7.27 (d, *J* = 8.4 Hz, 2H), 7.20 (d, *J* = 8.4 Hz, 2H), 5.04 (t, *J* = 4.8 Hz, 1H), 3.94-3.89 (m, 2H), 3.87-3.81 (m, 2H), 2.93 (d, *J* = 4.8 Hz, 2H); **^{13}C NMR** (151 MHz, $CDCl_3$) δ 134.52, 132.50, 131.10, 128.40, 104.24, 65.03, 40.04; **HRMS** (ESI) m/z calcd for $C_{10}H_{12}O_2Cl$ $[M+H]^+$ 199.0526, Found: 199.0524.

Spectroscopic data are in agreement with those reported in the literature.[5]

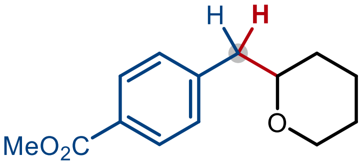

63

(**63**) According to the general procedure A, using *N*-triftosylhydrazone derived from methyl 4-formylbenzoate (115.9 mg, 0.3 mmol), tetrahydro-2*H*-pyran (5.0 mL) and $CHCl_3$ (5.0 mL) afforded compound **63** (45.7 mg, 65% yield) as a colorless oil. **^{1}H NMR** (600 MHz, $CDCl_3$) δ 7.95 (d, *J* = 8.4 Hz, 2H), 7.28 (d, *J* = 8.4 Hz, 2H), 3.97-3.95 (m, 1H), 3.90 (s, 3H), 3.52-3.48 (m, 1H), 3.39 (td, *J* = 12.0 Hz, *J* = 2.4 Hz, 1H), 2.89 (dd, *J* = 13.8 Hz, *J* = 6.6 Hz, 1H), 2.71 (dd, *J* = 13.8 Hz, *J* = 6.0 Hz, 1H), 1.83-1.80 (m, 1H), 1.59-1.53 (m, 2H), 1.50-1.41 (m, 2H), 1.34-1.27 (m, 1H); **^{13}C NMR** (151 MHz, $CDCl_3$) δ 167.13, 144.44, 129.51, 129.37, 128.07, 78.30, 68.61, 51.94, 43.10, 31.54, 25.92, 23.44; **HRMS** (ESI) m/z calcd for $C_{14}H_{19}O_3$ $[M+H]^+$ 235.1334, Found: 235.1338.

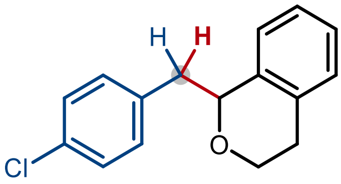

64

(**64**) According to the general procedure A, using *N*-triftosylhydrazone derived from methyl 4-chlorobenzaldehyde (108.8 mg, 0.3 mmol), isochromane (80.5 mg, 2.0 equiv) and $CHCl_3$ (5.0 mL) afforded compound **64** (60.6 mg, 78% yield) as a white solid, m.p. 77-78 °C **^{1}H NMR** (600 MHz, $CDCl_3$) δ 7.25-7.22 (m, 2H), 7.20-7.15 (m, 4H), 7.13-7.11 (m, 1H), 7.09 (d, *J* = 6.6 Hz, 1H), 4.99 (dd, *J* = 9.0 Hz, *J* = 3.6 Hz, 1H), 4.12-4.09 (m, 1H), 3.74-3.70 (m, 1H), 3.19 (dd, *J* = 14.4 Hz, *J* = 3.6 Hz, 1H), 3.01 (dd, *J* = 14.4 Hz, *J* = 3.0 Hz, 1H), 2.91-2.86 (m, 1H), 2.66 (dt, *J* = 16.2 Hz, *J* = 3.6 Hz, 1H); **^{13}C NMR** (151 MHz, $CDCl_3$) δ 137.39, 137.16, 134.24, 131.99, 130.87, 128.96, 128.20, 126.44, 126.05, 124.94, 76.36, 62.99, 41.81, 29.06; **HRMS** (ESI) m/z calcd for $C_{16}H_{16}OCl$ $[M+H]^+$ 259.0890, Found: 259.0890.

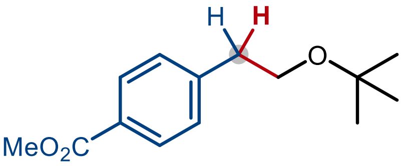

65

(**65**) According to the general procedure A, using *N*-triftosylhydrazone derived from methyl 4-formylbenzoate (115.9 mg, 0.3 mmol), isobutyl methyl ether (5.0 mL) and $CHCl_3$ (5.0 mL) afforded compound **65** (43.9 mg, 62% yield) as a colorless oil. **^{1}H NMR** (600 MHz, $CDCl_3$) δ 7.95 (d, *J* = 8.4 Hz, 2H), 7.30 (d, *J* = 8.4 Hz, 2H), 3.90 (s, 3H), 3.56 (t, *J* = 7.2 Hz, 2H), 2.87 (t, *J* = 7.2 Hz, 2H), 1.15 (s, 9H); **^{13}C NMR** (151 MHz, $CDCl_3$) δ 167.16, 145.17, 129.50, 129.05, 128.02, 72.93, 62.33, 51.95, 37.37, 27.45; **HRMS** (ESI) m/z calcd for $C_{14}H_{21}O_3$ $[M+H]^+$ 237.1491, Found: 237.1488.

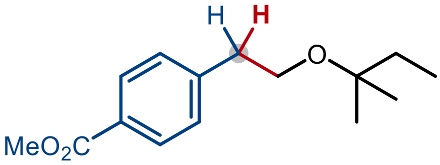

66

(**66**) According to the general procedure A, using *N*-triftosylhydrazone derived from methyl 4-formylbenzoate (115.9 mg, 0.3 mmol), 2-methoxy-2-methylbutane (5.0 mL) and $CHCl_3$ (5.0 mL) afforded compound **66** (45.1 mg, 60% yield) as a colorless oil. **^{1}H NMR** (600 MHz, $CDCl_3$) δ 7.95 (d, *J* = 7.8 Hz, 2H), 7.30 (d, *J* = 7.8 Hz, 2H), 3.90 (s, 3H), 3.52 (t, *J* = 7.2 Hz, 2H), 2.87 (t, *J* = 7.2 Hz, 2H), 1.45 (q, *J* = 7.2 Hz, 2H), 1.08 (s, 6H), 0.80 (t, *J* = 7.2 Hz, 3H); **^{13}C NMR** (151 MHz, $CDCl_3$) δ 167.18, 145.31, 129.48, 129.09, 127.99, 74.91, 61.87, 51.94, 37.34, 32.71, 24.93, 8.16; **HRMS** (ESI) m/z calcd for $C_{15}H_{23}O_3$ $[M+H]^+$ 251.1647, Found: 251.1640.

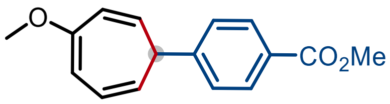

67

(**67**) According to the general procedure A, using *N*-triftosylhydrazone derived from methyl 4-formylbenzoate (115.9 mg, 0.3 mmol), anisole (162.2 mg, 5.0 equiv) and $CHCl_3$ (5.0 mL) afforded compound **67** (40.0 mg, 52% yield) as a colorless oil. **^{1}H NMR** (600 MHz, $CDCl_3$) δ 8.04 (d, *J* = 8.4 Hz, 2H), 7.43 (d, *J* = 8.4 Hz, 2H), 6.23 (t, *J* = 7.8 Hz, 1H), 6.12 (d, *J* = 10.2 Hz, 1H), 5.96 (dd, *J* = 6.6 Hz, *J* = 1.8 Hz, 1H), 5.54 (dd, *J* = 10.2 Hz, *J* = 6.0 Hz, 1H), 5.26 (dd, *J* = 9.6 Hz, *J* = 5.4 Hz, 1H), 3.92 (s, 3H), 3.71 (s, 3H), 3.00 (t, *J* = 5.4 Hz, 1H); **^{13}C NMR** (151 MHz, $CDCl_3$) δ 166.99, 160.20, 148.99, 130.01, 128.60, 127.67, 127.60, 123.96, 122.03, 121.02, 104.49, 54.73, 52.04, 45.08; **HRMS** (ESI) m/z calcd for $C_{16}H_{17}O_3$ $[M+H]^+$ 257.1178, Found:257.1184.

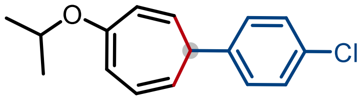

68

(**68**) According to the general procedure A, using *N*-triftosylhydrazone derived from 4-chlorobenzaldehyde (108.8 mg, 0.3 mmol), isopropoxybenzene (204.3 mg, 5.0 equiv) and $CHCl_3$ (5.0 mL) afforded compound **68** (35.2 mg, 45% yield) as a yellow oil. **^{1}H NMR** (600 MHz, $CDCl_3$) δ 7.33 (d, *J* = 8.4 Hz, 2H), 7.29 (d, *J* = 8.4 Hz, 2H), 6.21-6.18 (m, 1H), 6.09 (d, *J* = 9.6 Hz, 1H), 6.05 (dd, *J* = 7.2 Hz, *J* = 1.8 Hz, 1H), 5.50 (dd, *J* = 9.6 Hz, *J* = 6.0 Hz, 1H), 5.21 (dd, *J* = 9.0 Hz, *J* = 5.4 Hz, 1H), 4.42-4.36 (m, 1H), 2.88 (t, *J* = 5.4 Hz, 1H), 1.32 (d, *J* = 6.0 Hz, 3H), 1.27 (d, *J* = 6.0 Hz, 3H); **^{13}C NMR** (151 MHz, $CDCl_3$) δ 158.14, 142.23, 132.29, 128.91, 128.72, 127.64, 123.98, 122.60, 121.46, 108.75, 69.86, 44.64, 22.26, 21.68; **HRMS** (ESI) m/z calcd for $C_{16}H_{18}OCl$ $[M+H]^+$ 261.1046, Found: 261.1052.

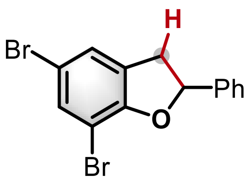

69

(**69**) According to the general procedure B, using *N*-triftosylhydrazone **S69** (176.8 mg, 0.3 mmol), $Tp^{Br3}Ag(thf)$ (33.0 mg, 10 mol%) and $CHCl_3$ (5 mL) afforded compound **69** (63.4 mg, 60% yield) as a white solid, m.p. 92-94 °C**;** ^{1}**H NMR** (600 MHz, $CDCl_3$) δ 7.46 (s, 1H), 7.39-7.36 (m, 4H), 7.34-7.31 (m, 1H), 7.22 (s, 1H), 5.86 (dd, *J* = 9.6 Hz, *J* = 7.8 Hz, 1H), 3.73 (dd, *J* = 16.2 Hz, *J* = 9.6 Hz, 1H), 3.29 (dd, *J* = 16.2 Hz, *J* = 7.8 Hz, 1H); ^{13}**C NMR** (151 MHz, $CDCl_3$) δ 156.42, 140.67, 133.39, 129.63, 128.77, 128.41, 126.85, 125.65, 112.60, 103.11, 84.85, 39.09; **HRMS** (ESI) m/z calcd for $C_{14}H_{11}OBr_2$ $[M+H]^+$ 352.9177, Found: 352.9179.

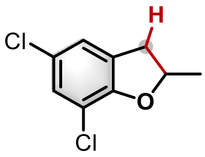

70

(**70**) According to the general procedure B, using *N*-triftosylhydrazone **S70** (132.4 mg, 0.3 mmol), $Tp^{Br3}Ag(thf)$ (33.0 mg, 10 mol%) and $CHCl_3$ (10 mL) afforded compound **70** (27.4 mg, 45% yield) as a colorless oil. ^{1}**H NMR** (600 MHz, $CDCl_3$) δ 7.12 (s, 1H), 7.01 (s, 1H), 5.07-5.01 (m, 1H), 3.36 (dd, *J* = 15.6 Hz, *J* = 8.4 Hz, 1H), 2.87 (dd, *J* = 15.6 Hz, *J* = 7.8 Hz, 1H), 1.51 (d, *J* = 6.0 Hz, 3H); ^{13}**C NMR** (151 MHz, $CDCl_3$) δ 154.42, 130.01, 127.84, 125.04, 123.52, 115.23, 81.17, 37.63, 21.60; **HRMS** (ESI) m/z calcd for $C_9H_9OCl_2$ $[M+H]^+$ 203.0030, Found: 203.0036.

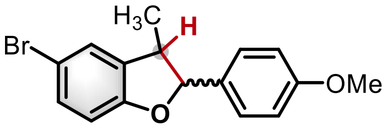

71

(**71**) According to the general procedure B, using *N*-triftosylhydrazone **S71** (167.2 mg, 0.3 mmol), $Tp^{Br3}Ag(thf)$ (33.0 mg, 10 mol%) and $CHCl_3$ (10 mL) afforded product **71** (76.6 mg, 80% yield, 2.5:1 d.r.) as a colorless oil. (**71-major**) ^{1}**H NMR** (500 MHz, $CDCl_3$) δ 7.26-7.22 (m, 2H), 7.19 (d, *J* = 8.5 Hz, 2H), 6.88 (d, *J* = 8.5 Hz, 2H), 6.76 (d, *J* = 8.5 Hz, 1H), 5.76 (d, *J* = 9.0 Hz, 1H), 3.79 (s, 3H), 3.67-3.60 (m, 1H), 0.81 (d, *J* = 7.0 Hz, 3H); ^{13}**C NMR** (151 MHz, $CDCl_3$) δ 159.20, 158.31, 135.24, 130.93, 130.84, 127.49, 127.41, 113.61, 112.39, 110.94, 88.12, 55.20, 40.86, 16.70; (**71-minor**) ^{1}**H NMR** (500 MHz, $CDCl_3$) δ 7.32 (d, *J* = 8.5 Hz, 2H), 7.26-7.22 (m, 2H),

6.90 (d, *J* = 8.5 Hz, 2H), 6.71 (d, *J* = 8.5 Hz, 1H),5.11 (d, *J* = 9.0 Hz, 1H), 3.80 (s, 3H), 3.45-3.39 (m, 1H), 1.37 (d, *J* = 6.5 Hz, 3H); **^{13}C NMR** (151 MHz, $CDCl_3$) δ 159.74, 158.27, 134.51, 132.03, 129.46, 127.56, 126.69, 114.04, 112.42, 111.02, 92.84, 55.27, 45.20, 17.68; **HRMS** (ESI) m/z calcd for $C_{16}H_{16}BrO_2$ $[M+H]^+$ 319.0334, Found: 319.0342.

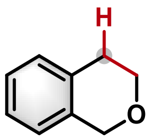

72

(**72**) According to the general procedure B, using *N*-triftosylhydrazone **S72** (111.7 mg, 0.3 mmol), Tp^{Br3}Ag(thf) (16.5 mg, 5 mol%) and $CHCl_3$ (5 mL) afforded product **72** (17.2 mg, 65% yield) as a colorless oil. **^{1}H NMR** (600 MHz, $CDCl_3$) δ 7.16-7.13 (m, 2H), 7.12-7.10 (m, 1H), 6.99-6.96 (m, 1H), 4.77 (s, 2H), 3.97 (d, *J* = 6.0 Hz, 2H), 2.85 (d, *J* = 6.0 Hz, 2H); **^{13}C NMR** (151 MHz, $CDCl_3$) δ 134.86, 133.14, 128.86, 126.29, 125.91, 124.34, 67.90, 65.34, 28.26.

Spectroscopic data are in agreement with those reported in the literature.[6]

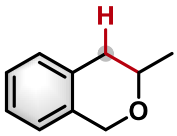

73

(**73**) According to the general procedure B, using *N*-triftosylhydrazone **S73** (115.9 mg, 0.3 mmol), Tp^{Br3}Ag(thf) (16.5 mg, 5 mol%) and $CHCl_3$ (5 mL) afforded product **73** (40.0 mg, 90% yield) as yellow oil; **^{1}H NMR** (600 MHz, $CDCl_3$) δ 7.17-7.14 (m, 2H), 7.10-7.08 (m, 1H), 7.00-6.98 (m, 1H), 4.86-4.80 (m, 2H), 3.85-3.79 (m, 1H), 2.71 (d, *J* = 6.6 Hz, 2H), 1.36 (d, *J* = 6.0 Hz, 3H); **^{13}C NMR** (151 MHz, $CDCl_3$) δ 134.64, 133.50, 128.72, 126.33, 125.92, 124.14, 70.97, 68.16, 35.79, 21.62; **HRMS** (ESI) m/z calcd for $C_{10}H_{13}O$ $[M+H]^+$ 149.0966, Found: 149.0974.

Spectroscopic data are in agreement with those reported in the literature.[7]

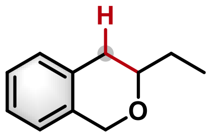

74

(**74**) According to the general procedure B, using *N*-triftosylhydrazone **S74** (120.1 mg, 0.3 mmol), $Tp^{Br3}Ag(thf)$ (16.5 mg, 5 mol%) and $CHCl_3$ (5 mL) afforded product **74** (41.4 mg, 85% yield) as a yellow oil. **^{1}H NMR** (600 MHz, $CDCl_3$) δ 7.16-7.13 (m, 2H), 7.11-7.08 (m, 1H), 6.99-6.97 (m, 1H), 4.82 (ABq, *J* = 15.0 Hz, 2H), 3.59-3.55 (m, 1H), 2.74-2.67 (m, 2H), 1.75-1.67 (m, 1H), 1.65-1.58 (m, 1H), 1.03 (t, *J* = 7.8 Hz, 3H); **^{13}C NMR** (151 MHz, $CDCl_3$) δ 134.92, 133.53, 128.84, 126.28, 125.84, 124.12, 76.30, 68.24, 33.69, 28.86, 9.89; **HRMS** (ESI) m/z calcd for $C_{11}H_{15}O$ $[M+H]^+$ 163.1123, Found: 163.1117.

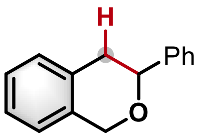

75

(**75**) According to the general procedure B, using *N*-triftosylhydrazone **S75** (134.6, 0.3 mmol), $Tp^{Br3}Ag(thf)$ (16.5 mg, 5 mol%) and $CHCl_3$ (5 mL) afforded product **75** (31.5 mg, 50% yield) as a white solid, m.p. 75-76 ^oC. **^{1}H NMR** (600 MHz, $CDCl_3$) δ 7.28 (t, *J* = 7.2 Hz, 2H), 7.25-7.20 (m, 5H), 7.18 (d, *J* = 7.2 Hz, 1H), 7.00 (d, *J* = 7.2 Hz, 1H), 5.49 (t, *J* = 6.0 Hz, 1H), 5.06-5.01 (m, 2H), 3.11-3.05 (m, 2H); **^{13}C NMR** (151 MHz, $CDCl_3$) δ 141.44, 139.44, 137.75, 129.59, 128.23, 127.49, 127.01, 126.32, 121.54, 120.92, 84.48, 72.54, 42.78; **HRMS** (ESI) m/z calcd for $C_{15}H_{15}O$ $[M+H]^+$ 211.1123, Found: 211.1125.

Spectroscopic data are in agreement with those reported in the literature.[8]

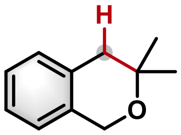

76

(**76**) According to the general procedure B, using *N*-triftosylhydrazone **S76** (120.1 mg, 0.3 mmol), $Tp^{Br3}Ag(thf)$ (16.5 mg, 5 mol%) and $CHCl_3$ (5 mL) afforded product **76** (42.3 mg, 87% yield) as a colorless oil. **^{1}H NMR** (600 MHz, $CDCl_3$) δ 7.17-7.14 (m, 2H), 7.08-7.05 (m, 1H), 7.02-6.98 (m, 1H), 4.79 (s, 2H), 2.71 (s, 2H), 1.29 (s, 6H); **^{13}C NMR** (151 MHz, $CDCl_3$) δ 133.90, 132.97, 129.13, 126.28, 125.77, 123.88, 70.78, 63.04, 39.64, 26.41; **HRMS** (ESI) m/z calcd for $C_{11}H_{15}O$ $[M+H]^+$ 163.1123, Found: 163.1122.

Spectroscopic data are in agreement with those reported in the literature.[9]

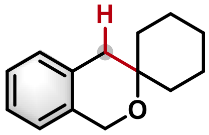

77

(**77**) Following the general procedure **B**, using *N*-triftosylhydrazone **S77** (132.2 mg, 0.3 mmol), $Tp^{Br3}Ag(thf)$ (16.5 mg, 5 mol%) and $CHCl_3$ (5 mL) afforded product **77** (57.7 mg, 95% yield); **^{1}H NMR** (600 MHz, $CDCl_3$) δ 7.15-7.12 (m, 2H), 7.07-7.05 (m, 1H), 6.99-6.97 (m, 1H), 4.75 (s, 2H), 2.67 (s, 2H), 1.75-1.72 (m, 2H), 1.69-1.62 (m, 2H), 1.61-1.56 (m, 1H), 1.50-1.45 (m, 2H), 1.43-1.39 (m, 2H), 1.37-1.31 (m, 1H); **^{13}C NMR** (151 MHz, $CDCl_3$) δ 134.29, 132.70, 129.22, 126.21, 125.66, 123.85, 71.60, 62.10, 38.82, 34.80, 26.01, 21.92; **HRMS** (ESI) m/z calcd for $C_{14}H_{19}O$ $[M+H]^+$ 203.1436, Found: 203.1439.

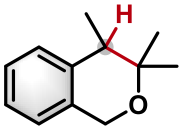

78

(**78**) According to the general procedure B, using *N*-triftosylhydrazone **S78** (124.3 mg, 0.3 mmol), $Tp^{Br3}Ag(thf)$ (16.5 mg, 5 mol%) and $CHCl_3$ (5 mL) afforded product **78** (25.4 mg, 48% yield) as a colorless oil. **^{1}H NMR** (600 MHz, $CDCl_3$) δ 7.20-7.17 (m, 2H), 7.16-7.13 (m, 1H), 6.97 (d, *J* = 7.8 Hz, 1H), 4.83-4.77 (m, 2H), 2.65 (q, *J* = 7.2 Hz, 1H), 1.27 (s, 3H), 1.24 (d, *J* = 7.2 Hz, 3H), 1.21 (s, 3H); **^{13}C NMR** (151 MHz, $CDCl_3$) δ 139.09, 133.42, 128.22, 126.41, 125.57, 123.59, 73.16, 63.08, 40.76, 25.73, 23.77, 17.66; **HRMS** (ESI) m/z calcd for $C_{12}H_{17}O$ $[M+H]^+$ 177.1279, Found: 177.1269.

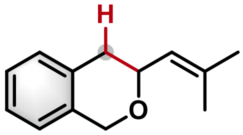

79

(**79**) According to the general procedure B, using *N*-triftosylhydrazone **S79** (128.0 mg, 0.3 mmol), $Tp^{Br3}Ag(thf)$ (16.5 mg, 5 mol%) and $CHCl_3$ (5 mL) afforded product **79** (25.4 mg, 45% yield) as a colorless oil. **^{1}H NMR** (500 MHz, $CDCl_3$) δ 7.29-7.24 (m, 2H), 7.20 (t, *J* = 7.5 Hz, 2H), 5.93 (dd, *J* = 17.5 Hz, *J* = 6.0 Hz, 1H), 5.12 (dd, *J* = 12.0 Hz, *J* = 3.0 Hz, 1H), 5.07 (dd, *J* = 11.0 Hz, *J* = 1.5 Hz, 1H), 5.05-5.00 (m, 3H), 1.12 (s, 3H), 0.98 (s, 3H); **^{13}C NMR** (126 MHz, $CDCl_3$) 144.80,

140.24, 139.35, 127.39, 126.54, 123.10, 120.70, 112.83, 90.92, 73.23, 43.00, 24.13, 21.16; **HRMS** (ESI) m/z calcd for $C_{13}H_{17}O$ $[M+H]^+$ 189.1279, Found: 189.1270.

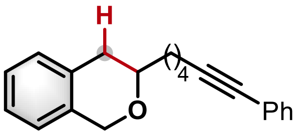

80

(**80**) According to the general procedure B, using *N*-triftosylhydrazone **S80** (158.6 mg, 0.3 mmol), $Tp^{Br3}Ag(thf)$ (16.5 mg, 5 mol%) and $CHCl_3$ (5 mL) afforded product **80** (65.3 mg, 75% yield) as a colorless oil. **^{1}H NMR** (600 MHz, $CDCl_3$) δ 7.40-7.39 (m, 2H), 7.29-7.24 (m, 3H), 7.15-7.13 (m, 2H), 7.09-7.07 (m, 1H), 6.99-6.97 (m, 1H), 4.85-4.78 (m, 2H), 3.68-3.64 (m, 1H), 2.72 (d, *J* = 7.2 Hz, 2H), 2.44 (t, *J* = 7.2 Hz, 2H), 1.75-1.57 (m, 6H); **^{13}C NMR** (151 MHz, $CDCl_3$) δ 134.84, 133.43, 131.51, 128.81, 128.15, 127.47, 126.29, 125.88, 124.12, 123.97, 90.11, 80.72, 74.77, 68.21, 35.48, 34.10, 28.75, 24.86, 19.38; **HRMS** (ESI) m/z calcd for $C_{21}H_{23}O$ $[M+H]^+$ 291.1749, Found: 291.1745.

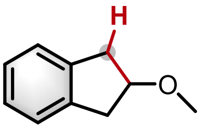

81

(**81**) According to the general procedure B, using *N*-triftosylhydrazone **S81** (115.9 mg, 0.3 mmol), $Tp^{Br3}Ag(thf)$ (16.5 mg, 5 mol%) and $CHCl_3$ (5 mL) afforded product **81** (36.9 mg, 83% yield) as a colorless oil. **^{1}H NMR** (600 MHz, $CDCl_3$) δ 7.22-7.20 (m, 2H), 7.16-7.13 (m, 2H), 4.26-4.23 (m, 1H), 3.38 (s, 3H), 3.16 (dd, *J* = 16.2 Hz, *J* = 6.6 Hz, 2H), 2.98 (dd, *J* = 16.2 Hz, *J* = 4.8 Hz, 2H); **^{13}C NMR** (151 MHz, $CDCl_3$) δ 140.82, 126.51, 124.70, 81.87, 56.58, 38.95; **HRMS** (ESI) m/z calcd for $C_{10}H_{13}O$ $[M+H]^+$ 149.0966, Found: 149.0963.

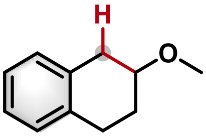

82

(**82**) According to the general procedure B, using *N*-triftosylhydrazone **S82** (120.0 mg, 0.3 mmol), $Tp^{Br3}Ag(thf)$ (16.5 mg, 5 mol%) and $CHCl_3$ (5 mL) afforded product **82** (46.7 mg, 96% yield) as a colorless oil. **^{1}H NMR** (600 MHz, $CDCl_3$) δ 7.11-7.06 (m, 4H), 3.68-3.63 (m, 1H), 3.42 (s, 3H), 3.07 (dd, *J* = 16.2 Hz, *J* = 4.8 Hz, 1H), 2.93 (dt, *J* = 16.2 Hz, *J* = 6.0 Hz, 1H), 2.80-2.75 (m, 2H), 2.10-2.06 (m, 1H), 1.85-1.79 (m, 1H); **^{13}C NMR** (151 MHz, $CDCl_3$) δ 136.04, 134.45, 129.42, 128.50, 125.81, 125.74, 75.75, 55.81, 35.15, 27.75, 26.93.

Spectroscopic data are in agreement with those reported in the literature.[10]

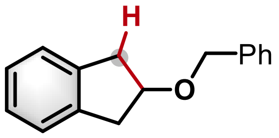

83

(**83**) According to the general procedure B, using *N*-triftosylhydrazone **S83** (138.8 mg, 0.3 mmol), $Tp^{Br3}Ag(thf)$ (16.5 mg, 5 mol%) and $CHCl_3$ (5 mL) afforded product **83** (53.8 mg, 80% yield) as a yellow oil. **^{1}H NMR** (600 MHz, $CDCl_3$) δ 7.38-7.31 (m, 4H), 7.29-7.26 (m, 1H), 7.22-7.19 (m, 2H), 7.16-7.13 (m, 2H), 4.59 (s, 2H), 4.46-4.43 (m, 1H), 3.18 (dd, *J* = 16.2 Hz, *J* = 6.6 Hz, 2H), 3.05 (dd, *J* = 16.2 Hz, *J* = 4.8 Hz, 2H); **^{13}C NMR** (151 MHz, $CDCl_3$) δ 140.86, 138.50, 128.37, 127.69, 127.54, 126.50, 124.68, 79.81, 71.05, 39.33; **HRMS** (ESI) m/z calcd for $C_{16}H_{17}O$ $[M+H]^+$ 225.1279, Found: 225.1272.

Spectroscopic data are in agreement with those reported in the literature. [10]

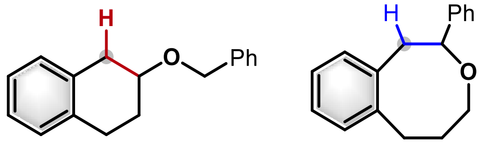

84-major **84-minor**

(**84-major**) According to the general procedure B, using *N*-triftosylhydrazone **S84** (142.9 mg, 0.3 mmol), $Tp^{Br3}Ag(thf)$ (16.5 mg, 5 mol%) and $CHCl_3$ (5 mL) afforded product **84** (68.1 mg, 95% yield, major : minor = 3 : 1) as a colorless oil. **^{1}H NMR** (600 MHz, $CDCl_3$) δ 7.37-7.31 (m, 4H), 7.28-7.24 (m, 1H), 7.11-7.06 (m, 4H), 4.63 (ABq, *J* = 12.0 Hz, 2H), 3.86-3.82 (m, 1H), 3.12-3.09 (m, 1H), 2.99-2.93 (m, 1H), 2.86 (dd, *J* = 16.2 Hz, *J* = 7.8 Hz, 1H), 2.81-2.74 (m, 1H), 2.15-2.10 (m, 1H), 1.90-1.85 (m, 1H); **^{13}C NMR** (151 MHz, $CDCl_3$) δ 138.83, 136.04, 134.59, 129.38, 128.47, 128.33, 127.53, 127.43, 125.78, 125.71, 73.78, 70.00, 35.58, 28.31, 27.23; (**84-minor**) **^{1}H NMR** (600 MHz, $CDCl_3$) δ 7.37-7.31 (m, 4H), 7.28-7.24 (m, 1H), 7.22-7.19 (m, 1H), 7.17 (d, *J* = 6.0 Hz, 1H), 7.15-7.12 (m, 1H), 7.01 (d, *J* = 7.2 Hz, 1H), 4.52 (dd, *J* = 9.0 Hz, *J* = 1.8 Hz, 1H), 4.00-3.96 (m, 1H), 3.21 (dd, *J* = 14.4 Hz, *J* = 9.6 Hz, 1H), 3.14 (td, *J* = 12.0 Hz, *J* = 6.0 Hz, 1H),

3.12-3.09 (m, 1H), 2.99-2.93 (m, 1H), 2.81-2.74 (m, 1H), 2.21-2.16 (m, 1H), 1.63-1.57 (m, 1H); **^{13}C NMR** (151 MHz, $CDCl_3$) δ 143.36, 140.72, 138.07, 130.06, 129.26, 128.25, 127.24, 127.05, 126.29, 126.06, 84.62, 68.23, 43.28, 33.12, 30.24; **HRMS** (ESI) m/z calcd for $C_{17}H_{19}O$ $[M+H]^+$ 239.1436, Found: 239.1430.

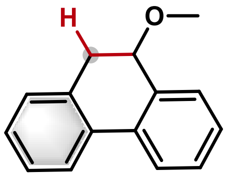

85

(**85**) According to the general procedure B, using *N*-triftosylhydrazone **S85** (134.6 mg, 0.3 mmol), $Tp^{Br3}Ag(thf)$ (16.5 mg, 5 mol%) and $CHCl_3$ (5 mL) afforded product **85** (60.6 mg, 96% yield) as a colorless oil. **^{1}H NMR** (600 MHz, $CDCl_3$) δ 7.81 (d, J = 7.8 Hz, 1H), 7.79 (d, J = 7.8 Hz, 1H), 7.42-7.39 (m, 2H), 7.32-7.23 (m, 4H), 4.35 (t, J = 4.8 Hz, 1H), 3.31 (s, 3H), 3.16-3.08 (m, 2H); **^{13}C NMR** (151 MHz, $CDCl_3$) δ 134.91, 133.80, 133.69, 133.28, 129.05, 128.79, 128.29, 127.80, 127.14, 124.11, 123.47, 76.66, 56.20, 34.74; **HRMS** (ESI) m/z calcd for $C_{15}H_{15}O$ $[M+H]^+$ 211.1123, Found: 211.1122.

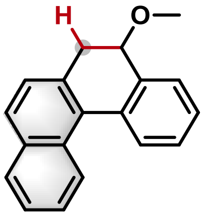

86

(**86**) According to the general procedure B, using *N*-triftosylhydrazone **S86** (149.6 mg, 0.3 mmol), $Tp^{Br3}Ag(thf)$ (16.5 mg, 5 mol%) and $CHCl_3$ (5 mL) afforded product **86** (67.2 mg, 86% yield) as a yellow oil. **^{1}H NMR** (600 MHz, $CDCl_3$) δ 8.57 (d, J = 8.4 Hz, 1H), 8.00 (d, J = 7.8 Hz, 1H), 7.85 (d, J = 8.4 Hz, 1H), 7.75 (d, J = 8.4 Hz, 1H), 7.54 (d, J = 7.2 Hz, 1H), 7.49 (td, J = 7.8 Hz, J = 1.2 Hz, 1H), 7.45-7.43 (m, 2H), 7.40 (d, J = 8.4 Hz, 1H), 7.36 (t, J = 7.2 Hz, 1H), 4.39 (dd, J = 6.6 Hz, J = 4.2 Hz, 1H), 3.41 (s, 3H), 3.17 (dd, J = 15.6 Hz, J = 4.2 Hz, 1H), 3.12 (dd, J = 15.6 Hz, J = 6.6 Hz, 1H); **^{13}C NMR** (151 MHz, $CDCl_3$) δ 137.67, 134.06, 133.48, 132.40, 130.79, 129.76, 128.98, 128.68, 128.04, 127.58, 127.03, 126.99, 126.90, 126.15, 125.56, 124.94, 76.75, 56.70, 36.04; **HRMS** (ESI) m/z calcd for $C_{19}H_{17}O$ $[M+H]^+$ 261.1279, Found: 261.1269.

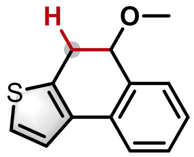

87

(**87**) According to the general procedure B, using *N*-triftosylhydrazone **S87** (136.4 mg, 0.3 mmol), $Tp^{Br3}Ag(thf)$ (16.5 mg, 5 mol%) and $CHCl_3$ (5 mL) afforded product **87** (62.3 mg, 96% yield) as a colorless oil. **^{1}H NMR** (600 MHz, $CDCl_3$) δ 7.66 (d, *J* = 7.2 Hz, 1H), 7.50 (d, *J* = 3.0 Hz, 1H), 7.39 (d, *J* = 7.2 Hz, 1H), 7.35 (td, *J* = 7.2 Hz, *J* = 1.2 Hz, 1H), 7.25 (td, *J* = 7.2 Hz, *J* = 1.2 Hz, 1H), 7.06-7.05 (m, 1H), 4.37 (t, *J* = 4.2 Hz, 1H), 3.29 (s, 3H), 3.18 (dd, *J* = 15.6 Hz, *J* = 4.8 Hz, 1H), 3.00 (ddd, *J* = 15.6 Hz, *J* = 4.2 Hz, *J* = 1.2 Hz, 1H); **^{13}C NMR** (151 MHz, $CDCl_3$) δ 136.29, 134.93, 133.91, 131.59, 128.79, 128.71, 126.64, 124.31, 120.53, 118.10, 77.75, 56.18, 31.01; **HRMS** (ESI) m/z calcd for $C_{13}H_{13}OS$ $[M+H]^+$ 217.0687, Found: 217.0690.

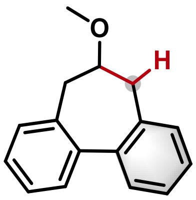

88

(**88**) According to the general procedure B, using *N*-triftosylhydrazone **S88** (138.8 mg, 0.3 mmol), $Tp^{Br3}Ag(thf)$ (33.0 mg, 10 mol%) and $CHCl_3$ (5 mL) afforded product **88** (59.2 mg, 88% yield) as a colorless oil. **^{1}H NMR** (600 MHz, $CDCl_3$) δ 7.41 (d, *J* = 7.8 Hz, 2H), 7.34 (t, *J* = 7.2 Hz, 2H), 7.28 (t, *J* = 7.2 Hz, 2H), 7.25 (d, *J* = 7.2 Hz, 2H), 4.04-4.00 (m, 1H), 3.44 (s, 3H), 3.08-2.19 (m, 4H); **^{13}C NMR** (151 MHz, $CDCl_3$) δ 140.63, 136.41, 129.33, 128.26, 127.35, 126.99, 85.60, 56.24, 37.19; **HRMS** (ESI) m/z calcd for $C_{16}H_{17}O$ $[M+H]^+$ 225.1279, Found: 225.1277.

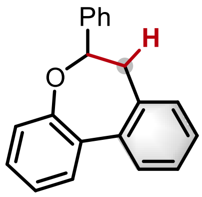

89

(**89**) According to the general procedure B, using *N*-triftosylhydrazone **S89** (153.2 mg, 0.3 mmol), $Tp^{Br3}Ag(thf)$ (16.5 mg, 5 mol%) and $CHCl_3$ (5 mL) afforded product **89** (73.5 mg, 90% yield) as a white solid, m.p. 119-120 °C; **^{1}H NMR** (500 MHz, $CDCl_3$) δ 7.77 (dd, *J* = 8.0 Hz, *J* = 1.5 Hz, 1H),

7.75 (d, *J* = 8.0 Hz, 1H), 7.37 (td, *J* = 7.5 Hz, *J* = 1.0 Hz, 1H), 7.30-7.20 (m, 5H), 7.13 (d, *J* = 7.0 Hz, 2H), 7.06 (td, *J* = 7.5 Hz, *J* = 1.0 Hz, 1H), 6.97-6.93 (m, 2H), 5.36 (dd, *J* = 9.0 Hz, *J* = 5.5 Hz, 1H), 3.19 (dd, *J* = 14.0 Hz, *J* = 8.5 Hz, 1H), 2.88 (dd, *J* = 14.0 Hz, *J* = 5.5 Hz, 1H); **^{13}C NMR** (125 MHz, $CDCl_3$) δ 152.23, 137.58, 133.98, 129.61, 129.52, 128.83, 128.27, 127.43, 126.46, 125.36, 122.99, 122.31, 122.14, 121.86, 118.40, 78.79, 41.42; **HRMS** (ESI) m/z calcd for $C_{20}H_{17}O$ $[M+H]^+$ 273.1279, Found: 273.1285.

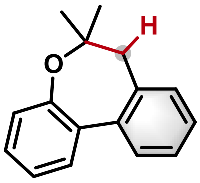

90

(**90**) According to the general procedure B, using *N*-triftosylhydrazone **S90** (138.8 mg, 0.3 mmol), $Tp^{Br3}Ag(thf)$ (33.0 mg, 10 mol%) and $CHCl_3$ (5 mL) afforded product **90** (43.7 mg, 65% yield) as a colorless oil. **^{1}H NMR** (600 MHz, $CDCl_3$) δ 7.46 (d, *J* = 7.8 Hz, 1H), 7.44 (dd, *J* = 7.8 Hz, *J* = 1.8 Hz, 1H), 7.37 (t, *J* = 7.8 Hz, 1H), 7.31-7.28 (m, 2H), 7.24-7.20 (m, 2H), 7.05 (d, *J* = 7.8 Hz, 1H), 2.59 (s, 2H), 1.40 (s, 6H); **^{13}C NMR** (151 MHz, $CDCl_3$) δ 153.19, 138.64, 136.54, 135.43, 129.28, 128.96, 128.53, 127.88, 127.20, 127.13, 124.28, 124.04, 89.12, 44.55, 26.60; **HRMS** (ESI) m/z calcd for $C_{16}H_{17}O$ $[M+H]^+$ 225.1279, Found: 225.1274.

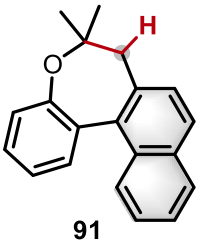

91

(**91**) According to the general procedure B, using *N*-triftosylhydrazone **S91** (153.8 mg, 0.3 mmol), $Tp^{Br3}Ag(thf)$ (16.5 mg, 5 mol%) and $CHCl_3$ (5 mL) afforded product **91** (80.7 mg, 98% yield) as a white solid, m.p. 132-133 ^oC**;** **^{1}H NMR** (600 MHz, $CDCl_3$) δ 8.15-8.13 (m, 1H), 7.88-7.86 (m, 1H), 7.79 (d, *J* = 8.4 Hz, 1H), 7.60 (dd, *J* = 7.8 Hz, *J* = 1.2 Hz, 1H), 7.44-7.40 (m, 2H), 7.38-7.34 (m, 2H), 7.27 (t, *J* = 7.8 Hz, 1H), 7.15 (d, *J* = 7.8 Hz, 1H), 2.76 (d, *J* = 7.8 Hz, 1H), 2.52 (d, *J* = 7.8 Hz, 1H), 1.43 (s, 3H), 1.29 (s, 3H); **^{13}C NMR** (151 MHz, $CDCl_3$) δ 154.16, 134.12, 134.06, 133.28, 132.97, 131.70, 131.20, 128.40, 128.26, 127.79, 127.38, 126.06, 125.77, 125.03, 124.44, 123.36, 89.86, 44.80, 26.72, 26.32; **HRMS** (ESI) m/z calcd for $C_{20}H_{19}O$ $[M+H]^+$ 275.1436, Found: 275.1445.

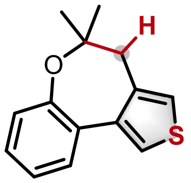

92

(**92**) According to the general procedure B, using *N*-triftosylhydrazone **S92** (140.6 mg, 0.3 mmol), $Tp^{Br3}Ag(thf)$ (16.5 mg, 5 mol%) and $CHCl_3$ (5 mL) afforded product **92** (67.7 mg, 98% yield) as a colorless oil. **^{1}H NMR** (600 MHz, $CDCl_3$) δ 7.43 (dd, *J* = 7.8 Hz, *J* = 1.8 Hz, 1H), 7.30 (d, *J* = 3.0 Hz, 1H), 7.26 (td, *J* = 7.8 Hz, *J* = 1.8 Hz, 1H), 7.15 (td, *J* = 7.8 Hz, *J* = 0.6 Hz, 1H), 7.06 (d, *J* = 3.0 Hz, 1H), 7.05 (d, *J* = 8.4 Hz, 1H), 2.62 (s, 2H), 1.40 (s, 6H); **^{13}C NMR** (151 MHz, $CDCl_3$) δ 152.46, 140.32, 137.19, 131.40, 128.48, 128.46, 124.63, 124.25, 121.31, 120.80, 85.46, 39.86, 27.02; **HRMS** (ESI) m/z calcd for $C_{14}H_{15}OS$ $[M+H]^+$ 231.0844, Found:231.0838.

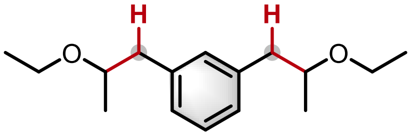

93

(**93**) According to the general procedure A, using *N*-triftosylhydrazone derived from isophthalaldehyde (173.6 mg, 0.3 mmol), $Tp^{Br3}Ag(thf)$ (33.0 mg, 10 mol%), Et_2O (5.0 mL) and $CHCl_3$ (5.0 mL) afforded compound **93** (56.3 mg, 75% yield) as a colorless oil. **^{1}H NMR** (600 MHz, $CDCl_3$) δ 7.19 (t, *J* = 7.8 Hz, 1H), 7.05-7.02 (m, 3H), 3.62-3.56 (m, 2H), 3.55-3.50 (m, 2H), 3.45-3.40 (m, 2H), 2.90 (dd, *J* = 13.2 Hz, *J* = 6.0 Hz, 2H), 2.58 (dd, *J* = 13.2 Hz, *J* = 6.6 Hz, 2H), 1.16 (t, *J* = 7.2 Hz, 6H), 1.12 (d, *J* = 6.0 Hz, 6H); **^{13}C NMR** (151 MHz, $CDCl_3$) δ 139.03, 139.01, 130.68, 130.65, 127.98, 127.10, 127.08, 76.54, 63.92, 43.10, 19.66, 19.64, 15.56; **HRMS** (ESI) m/z calcd for $C_{16}H_{27}O_2$ $[M+H]^+$ 251.2011, Found: 251.2014.

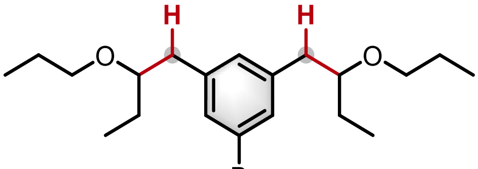

94

(**94**) According to the general procedure A, using *N*-triftosylhydrazone derived from isophthalaldehyde (197.2 mg, 0.3 mmol), $Tp^{Br3}Ag(thf)$ (33.0 mg, 10 mol%), 1-propoxypropane

(5.0 mL) and $CHCl_3$ (5.0 mL) afforded compound **94** (80.9 mg, 70% yield) as a colorless oil. **^{1}H NMR** (600 MHz, $CDCl_3$) δ 7.20 (d, *J* = 1.8 Hz, 2H), 6.98 (t, *J* = 1.8 Hz, 1H), 3.37-3.28 (m, 6H), 2.74 (dd, *J* = 13.8 Hz, *J* = 6.6 Hz, 2H), 2.63 (dd, *J* = 13.8 Hz, *J* = 6.0 Hz, 2H), 1.55-1.43 (m, 8H), 0.93 (t, *J* = 7.2 Hz, 6H), 0.87 (t, *J* = 7.2 Hz, 6H); **^{13}C NMR** (151 MHz, $CDCl_3$) δ 141.42, 141.39, 129.99, 129.95, 129.49, 121.82, 81.83, 81.79, 71.33, 40.10, 26.72, 26.68, 23.28, 10.70, 9.70; **HRMS** (ESI) m/z calcd for $C_{20}H_{34}O_2Br$ $[M+H]^+$ 385.1742, Found: 385.1752.

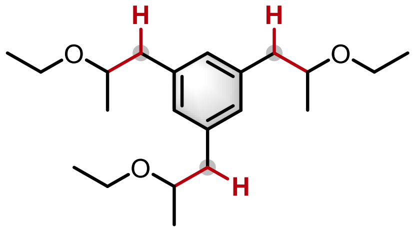

95

(**95**) According to the general procedure A, using *N*-triftosylhydrazone derived from benzene-1,3,5-tricarbaldehyde (248.6 mg, 0.3 mmol), $Tp^{Br3}Ag(thf)$ (33.0 mg, 10 mol%), Et_2O (5.0 mL) and $CHCl_3$ (5.0 mL) afforded compound **95** (34.3 mg, 34% yield) as a colorless oil. **^{1}H NMR** (600 MHz, $CDCl_3$) δ 6.87 (s, 3H), 3.60-3.50 (m, 6H), 3.45-3.41 (m, 3H), 2.90-2.87 (m, 3H), 2.54 (dd, *J* = 13.2 Hz, *J* = 7.2 Hz, 3H), 1.16 (t, *J* = 7.2 Hz, 9H), 1.11 (d, *J* = 6.6 Hz, 9H); **^{13}C NMR** (151 MHz, $CDCl_3$) δ 138.87, 138.86, 138.84, 128.37, 128.33, 76.64, 63.94, 43.08, 19.70, 19.68, 19.65, 15.58; **HRMS** (ESI) m/z calcd for $C_{21}H_{37}O_3$ $[M+H]^+$ 337.2743, Found: 337.2748.

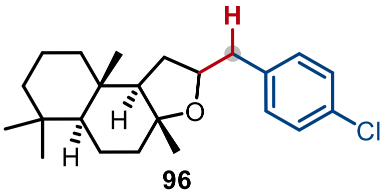

96

(**96**) According to the general procedure A, using *N*-triftosylhydrazone derived from 4-chlorobenzaldehyde (108.8 mg, 0.3 mmol), $Tp^{Br3}Ag(thf)$ (33.0 mg, 10 mol%), (-)-ambroxide (354.6 mg, 5.0 equiv) and $CHCl_3$ (5.0 mL) afforded compound **96** (75.8 mg, 70% yield, 1.7:1 d.r.) as a white solid, m.p. 83-85 °C. (**96-major**) **^{1}H NMR** (600 MHz, $CDCl_3$) δ 7.27-7.22 (m, 2H), 7.16-7.10 (m, 2H), 4.30-4.26 (m, 1H), 2.86-2.82 (m, 1H), 2.67-2.63 (m, 1H), 1.98-1.89 (m, 1H), 1.80-1.70 (m, 2H), 1.67-1.59 (m, 1H), 1.53-1.50 (m, 1H), 1.45-1.35 (m, 4H), 1.32-1.22 (m, 2H), 1.18-1.16 (m, 1H), 1.11 (d, *J* = 5.4 Hz, 3H), 0.98-0.73 (m, 11H); **^{13}C NMR** (151 MHz, $CDCl_3$) δ 137.16, 131.90, 130.87, 128.24, 81.16, 76.09, 58.78, 57.25, 42.47, 42.39, 39.86, 36.01, 33.53, 33.02, 27.25, 21.57, 21.08, 20.51, 18.33, 14.89; (**96-minor**) **^{1}H NMR** (600 MHz, $CDCl_3$) δ 7.27-7.22 (m, 2H), 7.16-7.10 (m, 2H), 4.13-4.09 (m, 1H), 3.01-2.96 (m, 1H), 2.73-2.68 (m, 1H), 1.98-1.89 (m, 1H), 1.80-1.70 (m, 2H), 1.67-1.59 (m, 1H), 1.45-1.35 (m, 5H), 1.32-1.22 (m, 2H), 1.18-1.16 (m, 3H), 0.98-0.73 (m, 12H); **^{13}C NMR** (151 MHz, $CDCl_3$) δ 137.55, 131.87, 130.52,

128.38, 80.80, 79.22, 60.85, 50.04, 43.93, 40.45, 39.94, 36.20, 33.48, 33.06, 29.18, 24.90, 21.04, 20.80, 18.36, 15.54; **HRMS** (ESI) m/z calcd for $C_{23}H_{34}OCl$ $[M+H]^+$ 361.2298, Found: 361.2298.

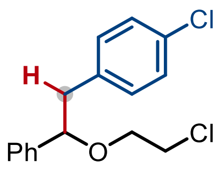

97

(**97**) According to the general procedure A, using *N*-triftosylhydrazone derived from 4-chlorobenzaldehyde (108.8 mg, 0.3 mmol), $Tp^{Br3}Ag(thf)$ (33.0 mg, 10 mol%), benxyl 2-chloroethyl ether (102.4 mg, 2.0 equiv) and $CHCl_3$ (5.0 mL) afforded compound **97** (58.4 mg, 66% yield) as a colorless oil. **^{1}H NMR** (600 MHz, $CDCl_3$) δ 7.32 (t, *J* = 7.2 Hz, 2H), 7.29-7.26 (m, 1H), 7.23-7.21 (m, 2H), 7.19 (d, *J* = 7.8 Hz, 2H), 7.04 (d, *J* = 7.8 Hz, 2H), 4.41 (dd, *J* = 7.2 Hz, *J* = 5.4 Hz, 1H), 3.59-3.55 (m, 1H), 3.52 (t, *J* = 5.4 Hz, 2H), 3.47-3.43 (m, 1H), 3.09 (dd, *J* = 13.8 Hz, *J* = 7.8 Hz, 1H), 2.86 (dd, *J* = 13.8 Hz, *J* = 5.4 Hz, 1H); **^{13}C NMR** (151 MHz, $CDCl_3$) δ 141.09, 136.68, 132.04, 130.95, 128.44, 128.12, 127.90, 126.65, 83.64, 68.94, 44.09, 42.89; **HRMS** (ESI) m/z calcd for $C_{16}H_{17}Cl_2O$ $[M+H]^+$ 295.0656, Found: 295.0651.

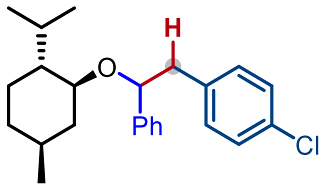

98

(**98**) According to the general procedure A, using *N*-triftosylhydrazone derived from 4-chlorobenzaldehyde (108.8 mg, 0.3 mmol), $Tp^{Br3}Ag(thf)$ (33.0 mg, 10 mol%), 2-isopropyl-5-methylcyclohexyl)oxy)- methyl)benzene (147.8 mg, 2.0 equiv) and $CHCl_3$ (5.0 mL) afforded compound **98** (55.6 mg, 50% yield) as a colorless oil. **^{1}H NMR** (600 MHz, $CDCl_3$) δ 7.29-7.27 (m, 2H), 7.25-7.23 (m, 1H), 7.21 (d, *J* = 7.8 Hz, 2H), 7.15 (d, *J* = 7.8 Hz, 2H), 6.99 (d, *J* = 7.8 Hz, 2H), 4.50 (t, *J* = 6.6 Hz, 1H), 3.07 (dd, *J* = 13.2 Hz, *J* = 7.2 Hz, 1H), 2.90-2.84 (m, 2H), 2.30-2.25 (m, 1H), 1.98-1.94 (m, 1H), 1.57-1.50 (m, 2H), 1.19-1.12 (m, 2H), 0.86 (d, *J* = 7.2 Hz, 3H), 0.85 (d, *J* = 6.6 Hz, 3H), 0.79-0.75 (m, 2H), 0.64 (q, *J* = 12.0 Hz, 1H), 0.28 (d, *J* = 7.2 Hz, 3H); **^{13}C NMR** (151 MHz, $CDCl_3$) δ 141.91, 137.26, 131.74, 131.03, 128.08, 127.92, 127.59, 127.50, 78.94, 74.95, 48.45, 44.26, 39.76, 34.46, 31.30, 24.85, 22.74, 22.36, 21.31, 15.45; **HRMS** (ESI) m/z calcd for $C_{24}H_{32}ClO$ $[M+H]^+$ 371.2142, Found: 371.2140.

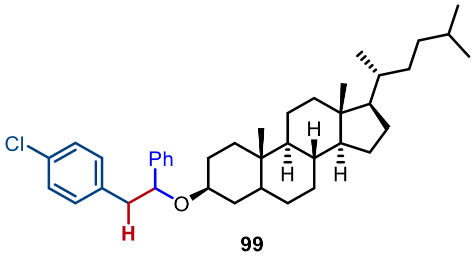

99

(**99**) According to the general procedure A, using *N*-triftosylhydrazone derived from 4-chlorobenzaldehyde (108.8 mg, 0.3 mmol), $Tp^{Br3}Ag(thf)$ (33.0 mg, 10 mol%), ether (278.9 mg, 2.0 equiv) and $CHCl_3$ (5.0 mL) afforded compound **99** (56.6 mg, 32% yield, 1.1:2 d.r.) as a colorless oil. ^{1}**H NMR** (500 MHz, $CDCl_3$) δ 7.31-7.20 (m, 5H), 7.21 (d, *J* = 8.0 Hz, 2H), 7.17 (d, *J* = 8.0 Hz, 2H), 4.55-4.50 (m, 1H), 3.10-2.94 (m, 2H), 2.79 (dd, *J* = 13.5 Hz, *J* = 5.5Hz, 1H), 1.92-1.87 (m, 1H), 1.81-1.70 (m, 1H), 1.62-1.45 (m, 6H), 1.40-0.95 (m, 19H), 0.88-0.84 (m, 10H), 0.75 (s, 3H), 0.61 (s, 3H), 0.51-0.46 (m, 1H); ^{13}**C NMR** (126 MHz, $CDCl_3$) δ 142.93, 142.87, 137.25, 137.16, 131.72 (2C), 130.92 (2C), 128.16, 128.13, 127.93 (2C), 127.33 (2C), 126.57, 126.53, 79.96 (2C), 76.41, 76.39, 56.41 (2C), 56.17, 54.30, 54.28, 44.82, 44.77, 44.53 (2C), 42.51 (2C), 39.96 (2C), 39.47 (2C), 36.98, 36.75, 36.12, 35.93, 35.74 (2C), 35.61 (2C), 35.37 (2C), 34.05 (2C), 32.04, 31.99, 29.32 (2C), 28.83 (2C), 28.63, 28.21, 27.98, 27.39, 24.16 (2C), 23.77 (2C), 22.82 (2C), 22.56 (2C), 21.15, 21.12, 18.63 (2C), 12.24, 12.03. **HRMS** (EI) m/z calcd for $C_{40}H_{58}ClO$ $[M+H]^+$ 589.4176, Found: 589.4180.

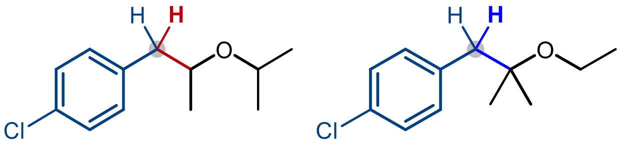

100-major **100-minor**

(**100**) According to the general procedure A, using *N*-triftosylhydrazone derived from 4-chlorobenzaldehyde (108.8 mg, 0.3 mmol), $Tp^{Br3}Ag(thf)$ (33.0 mg, 10 mol%), 2-ethoxypropane (1 mL) and $CHCl_3$ (5.0 mL) afforded compound **100** (57.4 mg, 90% yield, major: minor = 2:1) as a colorless oil. (**100-major**) ^{1}**H NMR** (600 MHz, $CDCl_3$) δ 7.25-7.21 (m, 2H), 7.15-7.11 (m, 2H), 3.63-3.58 (m, 1H), 3.54-3.49 (m, 1H), 2.76 (dd, *J* = 13.2 Hz, *J* = 6.6 Hz, 1H), 2.60 (dd, *J* = 13.2 Hz, *J* = 6.6 Hz, 1H), 1.12-1.10 (m, 6H), 0.98 (d, *J* = 6.0 Hz, 3H); ^{13}**C NMR** (151 MHz, $CDCl_3$) δ 137.88, 131.74, 130.85, 128.13, 73.95, 69.64, 43.18, 22.92, 22.42, 20.78. (**100-minor**) ^{1}**H NMR** (600 MHz, $CDCl_3$) δ 7.25-7.21 (m, 2H), 7.15-7.11 (m, 2H), 3.46 (q, *J* = 7.0 Hz, 2H), 2.73 (s, 2H), 1.18 (t, *J* = 7.2 Hz, 3H), 1.12 (s, 6H); ^{13}**C NMR** (151 MHz, $CDCl_3$) δ 137.03, 131.88, 131.77, 127.86, 74.74, 56.65, 46.09, 25.28, 16.02. **HRMS** (ESI) m/z calcd for $C_{12}H_{18}ClO$ $[M+H]^+$ 213.1046, Found: 213.1042.

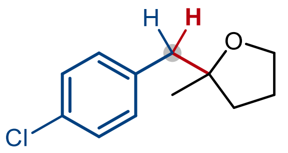

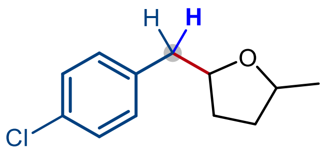

101-major **101-minor**

(**101**) According to the general procedure A, using *N*-triftosylhydrazone derived from 4-chlorobenzaldehyde (108.8 mg, 0.3 mmol), 2-methyltetrahydrofuran (1 mL) and $CHCl_3$ (5.0 mL) afforded product **101** (58.1 mg, 92% yield, major : minor = 7:1) as a colorless oil. (**101-major**) **^{1}H NMR** (500 MHz, $CDCl_3$) δ 7.24 (d, *J* = 8.0 Hz, 2H), 7.16 (d, *J* = 8.0 Hz, 2H), 3.86-3.82 (m, 1H), 3.76-7.72 (m, 1H), 2.75 (s, 2H), 1.96-1.68 (m, 3H), 1.65-1.60 (m, 1H), 1.16 (s, 3H); **^{13}C NMR** (151 MHz, $CDCl_3$) δ 136.98, 131.71, 130.68, 127.96, 82.51, 67.44, 46.16, 36.31, 26.36, 26.00; (**101-minor**) **^{1}H NMR** (500 MHz, $CDCl_3$) δ 7.24 (d, *J* = 8.0 Hz, 2H), 7.16 (d, *J* = 8.0 Hz, 2H), 4.05-3.99 (m, 1H), 3.98-3.92 (m, 1H), 2.90-2.86 (m, 1H), 2.73-2.69 (m, 1H), 1.96-1.68 (m, 3H), 1.65-1.60 (m, 1H), 1.22 (d, *J* = 6.5 Hz, 3H); **^{13}C NMR** (151 MHz, $CDCl_3$) δ 137.36, 131.98, 130.64, 128.27, 79.71, 75.52, 41.73, 32.69, 30.71, 21.41; **HRMS** (ESI) m/z calcd for $C_{12}H_{16}OCl$ $[M+H]^+$ 211.0890, Found: 211.0896.

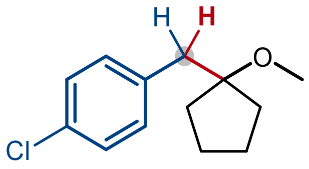

102

(**102**) According to the general procedure A, using *N*-triftosylhydrazone derived from 4-chlorobenzaldehyde (108.8 mg, 0.3 mmol), methoxycyclopentane (1.0 mL) and $CHCl_3$ (5.0 mL) afforded product **102** (50.6 mg, 75% yield) as a colorless oil. **^{1}H NMR** (600 MHz, $CDCl_3$) δ 7.23 (d, *J* = 8.4 Hz, 2H), 7.14 (d, *J* = 8.4 Hz, 2H), 3.26 (s, 3H), 2.83 (s, 2H), 1.74-1.66 (m, 4H), 1.59-1.53 (m, 2H), 1.46-1.41 (m, 2H); **^{13}C NMR** (151 MHz, $CDCl_3$) δ 137.18, 131.83, 131.28, 128.03, 86.90, 49.60, 40.08, 35.11, 23.24; **HRMS** (ESI) m/z calcd for $C_{13}H_{18}OCl$ $[M+H]^+$ 225.1046, Found:225.1055.

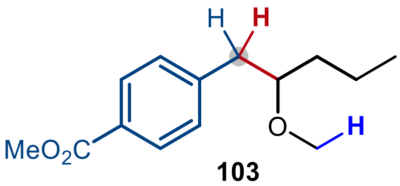

103

(**103**) Following the general procedure **A**, using *N*-triftosylhydrazone derived from methyl 4-formylbenzoate (115.9 mg, 0.3 mmol), 1-methoxybutane (1.0 mL) and $CHCl_3$ (5.0 mL) afforded product **103** (52.4 mg, 74% yield) as a colorless oil. **^{1}H NMR** (600 MHz, $CDCl_3$) δ 7.96 (d, *J* =

7.8 Hz, 2H), 7.28 (d, *J* = 7.8 Hz, 2H), 3.90 (s, 3H), 3.40-3.36 (m, 1H), 3.29 (s, 3H), 2.86 (dd, *J* = 13.8 Hz, *J* = 6.6 Hz, 1H), 2.77 (dd, *J* = 13.8 Hz, *J* = 6.0 Hz, 1H), 1.47-1.30 (m, 4H), 0.89 (t, *J* = 7.2 Hz, 3H); ^{13}**C NMR** (151 MHz, $CDCl_3$) δ 167.14, 144.82, 129.52, 129.43, 128.01, 81.79, 57.08, 51.95, 40.27, 35.87, 18.53, 14.10; **HRMS** (ESI) m/z calcd for $C_{14}H_{21}O_3$ $[M+H]^+$ 237.1491, Found: 237.1487.

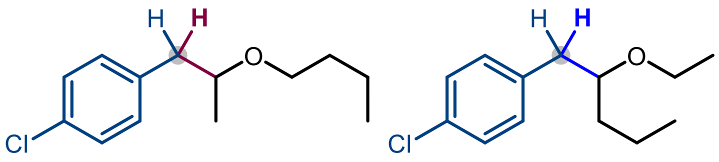

104-major **104-minor**

(**104**) According to the general procedure A, using *N*-triftosylhydrazone derived from 4-chlorobenzaldehyde (108.8 mg, 0.3 mmol), 1-ethoxybutane (1.0 mL) and $CHCl_3$ (5.0 mL) afforded product **104** (67.3 mg, 99% yield) as a colorless oil. The r.r. was 3:2 from ^{1}H- NMR analysis of crude residue and 2:1 in the isolated product. (**104-major**) ^{1}**H NMR** (600 MHz, $CDCl_3$) δ 7.24-7.23 (m, 2H), 7.14-7.12 (m, 2H), 3.57-3.51 (m, 1H), 3.48-3.44 (m, 1H), 3.33-3.29 (m, 1H), 2.82 (dd, *J* = 13.8 Hz, *J* = 6.6 Hz, 1H), 2.61 (dd, *J* = 13.8 Hz, *J* = 6.0 Hz, 1H), 1.52-1.46 (m, 2H), 1.34-1.30 (m, 2H), 1.11 (d, *J* = 6.0 Hz, 3H), 0.88 (t, *J* = 7.8 Hz, 3H); ^{13}**C NMR** (151 MHz, $CDCl_3$) δ 137.74, 131.76, 130.79, 128.19, 76.31, 68.55, 42.45, 32.14, 19.54, 19.33, 13.85. (**104-minor**) ^{1}**H NMR** (600 MHz, $CDCl_3$) δ 7.24-7.23 (m, 2H), 7.14-7.12 (m, 2H), 3.44-3.34 (m, 3H), 2.75 (dd, *J* = 13.8 Hz, *J* = 6.6 Hz, 1H), 2.68 (dd, *J* = 13.8 Hz, *J* = 5.4 Hz, 1H), 1.44-1.38 (m, 2H), 1.28-1.25 (m, 2H), 1.13-1.11 (m, 3H), 0.89 (t, *J* = 7.8 Hz, 3H); ^{13}**C NMR** (151 MHz, $CDCl_3$) δ 137.85, 131.72, 130.79, 128.22, 80.31, 64.88, 40.25, 36.47, 18.76, 15.49, 14.12; **HRMS** (ESI) m/z calcd for $C_{13}H_{20}OCl$ $[M+H]^+$ 227.1203, Found: 227.1201.

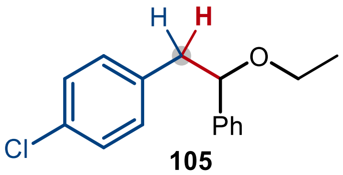

105

(**105**) According to the general procedure A, using *N*-triftosylhydrazone derived from 4-chlorobenzaldehyde (108.8 mg, 0.3 mmol), (ethoxymethyl)benzene (81.7 mg, 2.0 equiv) and $CHCl_3$ (5.0 mL) afforded product **105** (53.2 mg, 68% yield) as a yellow oil. ^{1}**H NMR** (600 MHz, $CDCl_3$) δ 7.30 (t, *J* = 7.2 Hz, 2H), 7.27-7.24 (m, 1H), 7.21-7.20 (m, 2H), 7.18 (d, *J* = 8.4 Hz, 2H), 7.02 (d, *J* = 8.4 Hz, 2H), 4.36 (dd, *J* = 7.2 Hz, *J* = 6.0 Hz, 1H), 3.39-3.34 (m, 1H), 3.28-3.23 (m, 1H), 3.07 (dd, *J* = 13.8 Hz, *J* = 7.2 Hz, 1H), 2.84 (dd, *J* = 13.8 Hz, *J* = 6.0 Hz, 1H), 1.12 (t, *J* = 6.6 Hz, 3H); ^{13}**C NMR** δ 142.02, 137.09, 131.86, 130.88, 128.27, 128.05, 127.54, 126.64, 82.95, 64.27, 44.21, 15.22; **HRMS** (ESI) m/z calcd for $C_{16}H_{18}OCl$ $[M+H]^+$ 261.1046, Found: 261.1042.

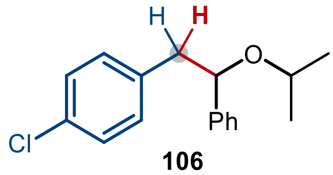

106

(**106**) According to the general procedure A, using *N*-triftosylhydrazone derived from 4-chlorobenzaldehyde (108.8 mg, 0.3 mmol), (isopropoxymethyl)benzene (90.1 mg, 2.0 equiv.) and $CHCl_3$ (5.0 mL) afforded product **106** (41.3 mg, 50% yield) as a colorless oil. **^{1}H NMR** (600 MHz, $CDCl_3$) δ 7.30 (t, *J* = 7.2 Hz, 2H), 7.26-7.23 (m, 3H), 7.18 (d, *J* = 7.8 Hz, 2H), 7.04 (d, *J* = 8.4 Hz, 2H), 4.45 (dd, *J* = 7.8 Hz, *J* = 5.4 Hz, 1H), 3.44-3.38 (m, 1H), 2.99 (dd, *J* = 13.2 Hz, *J* = 7.8 Hz, 1H), 2.81 (dd, *J* = 13.2 Hz, *J* = 5.4 Hz, 1H), 1.04 (d, *J* = 6.6 Hz, 3H), 0.98 (d, *J* = 6.0 Hz, 3H); **^{13}C NMR** (151 MHz, $CDCl_3$) δ 142.87, 137.31, 131.80, 130.98, 128.20, 127.96, 127.39, 126.61, 80.32, 69.26, 44.73, 23.31, 21.11; **HRMS** (ESI) m/z calcd for $C_{17}H_{20}OCl$ $[M+H]^+$ 275.1203, Found: 275.1194.

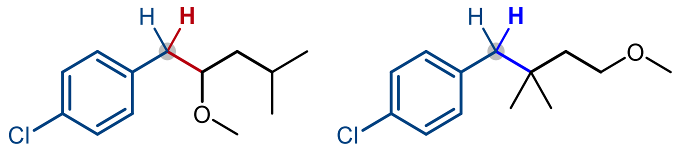

107-major **107-minor**

(**107**) According to the general procedure A, using *N*-triftosylhydrazone derived from 4-chlorobenzaldehyde (108.8 mg, 0.3 mmol), 1-methoxy-3-methylbutane (1.0 mL) and $CHCl_3$ (5.0 mL) afforded product **107** (51.0 mg, 75% yield, major = minor = 2 : 1 d.r.) as a colorless oil. (**107-major**) **^{1}H NMR** (600 MHz, $CDCl_3$) δ 7.25 (d, *J* = 8.4 Hz, 2H), 7.13 (d, *J* = 8.4 Hz, 2H), 3.42-3.36 (m, 1H), 3.30 (s, 3H), 2.76 (dd, *J* = 13.8 Hz, *J* = 6.6 Hz, 1H), 2.69 (dd, *J* = 13.8 Hz, *J* = 6.0 Hz, 1H), 1.78-1.71 (m, 1H), 1.42-1.38 (m, 1H), 1.18-1.14 (m, 1H), 0.88 (d, *J* = 6.6 Hz, 3H), 0.86 (d, *J* = 6.6 Hz, 3H); **^{13}C NMR** (151 MHz, $CDCl_3$) δ 137.53, 131.80, 130.78, 128.30, 80.29, 56.98, 43.24, 39.73, 24.61, 23.22, 22.34; (**107-minor**) **^{1}H NMR** (600 MHz, $CDCl_3$) δ 7.22 (d, *J* = 8.4 Hz, 2H), 7.05 (d, *J* = 8.4 Hz, 2H), 3.46 (t, *J* = 7.2 Hz, 2H), 3.30 (s, 3H), 2.50 (s, 2H), 1.52 (t, *J* = 7.2 Hz, 2H), 0.88 (s, 6H); **^{13}C NMR** (151 MHz, $CDCl_3$) δ 137.40, 131.89, 131.77, 127.80, 69.73, 58.62, 48.38, 40.87, 33.43, 26.85; **HRMS** (ESI) m/z calcd for $C_{13}H_{20}OCl$ $[M+H]^+$ 227.1203, Found: 227.1198.

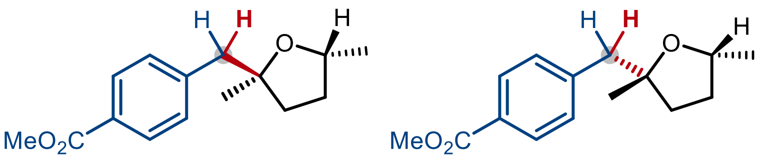

108-major **108-minor**

(**108**) According to the general procedure A, using *N*-triftosylhydrazone derived from methyl 4-formylbenzoate (115.9 mg, 0.3 mmol), 2,5-dimethyltetrahydrofuran (1.0 mL) and $PhCF_3$ (5.0 mL) afforded product **108** (67.0 mg, 90% yield, major : minor = 2 : 1) as a colorless oil. The regioselectivity was 1.9 :1 from ^{1}H NMR analysis of crude residue. (**108-major**) **^{1}H NMR** (600 MHz, $CDCl_3$) δ 7.94 (d, *J* = 8.4 Hz, 2H), 7.31 (d, *J* = 8.4 Hz, 2H), 3.95-3.91 (m, 1H), 3.901 (s, 3H), 2.83 (s, 2H), 1.91-1.84 (m, 2H), 1.71-1.67 (m, 1H), 1.54-1.48 (m, 1H), 1.21-1.20 (m, 6H); **^{13}C NMR** (151 MHz, $CDCl_3$) δ 167.26, 144.16, 130.54, 129.20, 128.05, 82.79, 75.28, 51.97, 47.09, 36.77, 36.62, 28.24, 21.86; **DEPT-135** (600 MHz, $CDCl_3$) δ 130.54, 129.20, 75.29, 51.99, 47.08, 36.75, 33.62, 28.24, 21.86. **(108-minor)** **^{1}H NMR** (600 MHz, $CDCl_3$) δ 7.95 (d, *J* = 8.4 Hz, 2H), 7.32 (d, *J* = 8.4 Hz, 2H), 4.09-4.04 (m, 1H), 3.903 (s, 3H), 2.87 (d, *J* = 13.2 Hz, 1H), 2.79 (d, *J* = 13.2 Hz, 1H), 1.91-1.83 (m, 2H), 1.66-1.63 (m, 1H), 1.21-1.20 (m, 3H), 1.12 (d, *J* = 6.6 Hz, 3H), 1.13-1.06 (m, 1H); **^{13}C NMR** (151 MHz, $CDCl_3$) δ 167.27, 144.02, 130.73, 129.03, 128.08, 82.73, 74.66, 51.97, 48.04, 36.59, 33.74, 27.37, 21.18; **DEPT-135** (600 MHz, $CDCl_3$) δ 130.73, 129.03, 74.67, 51.99, 48.04, 36.59, 33.74, 27.39, 21.17**.** **HRMS** (ESI) m/z calcd for $C_{15}H_{21}O_3$ $[M+H]^+$ 249.1491, Found: 249.1490.

The stereochemistry was confirmed by NOESY experiments.

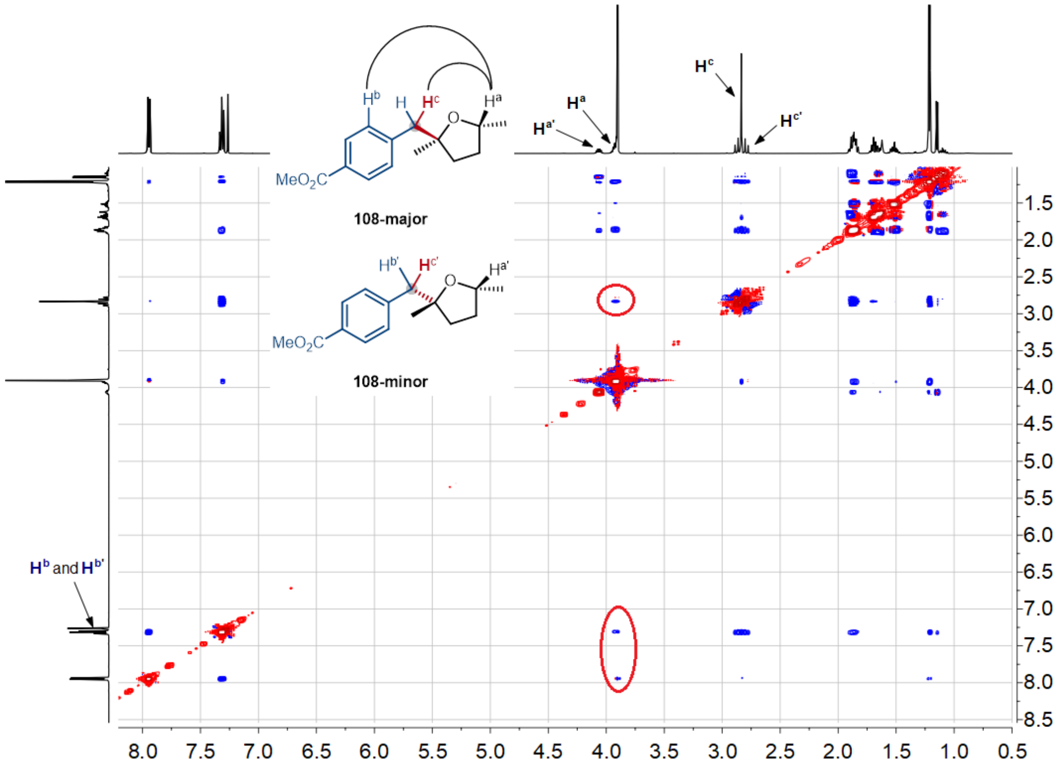

Supplementary Fig. 1. NOE spectra of compound 108

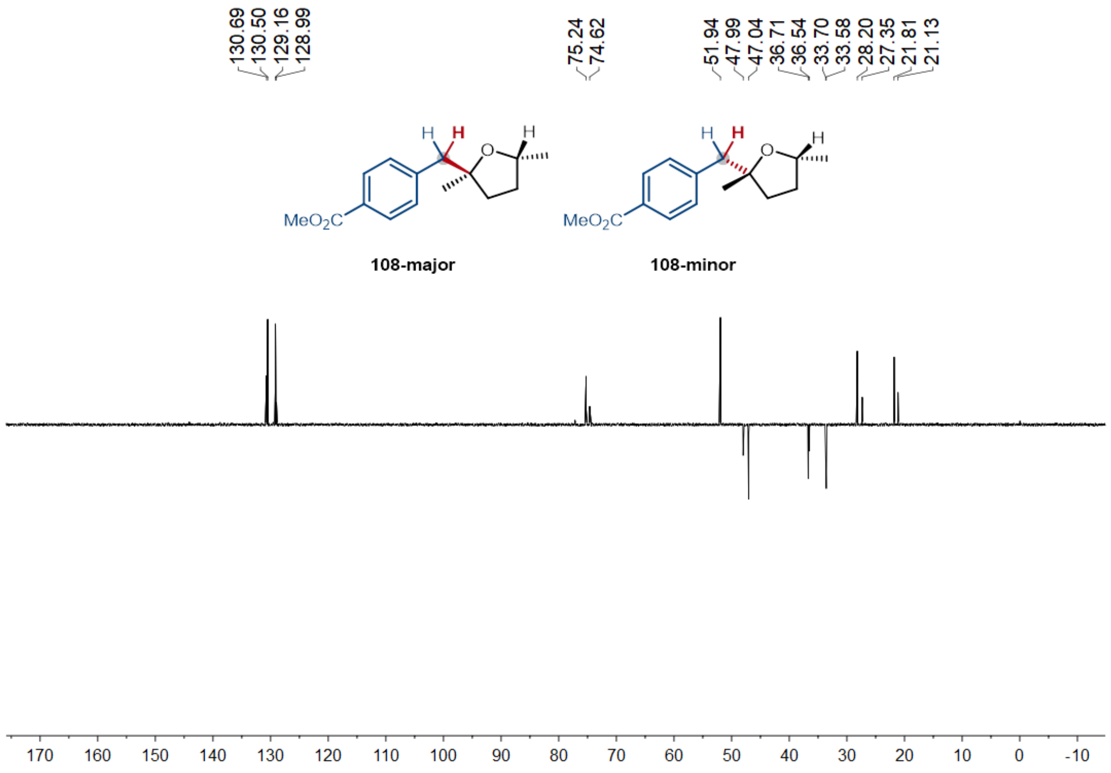

Supplementary Fig. 2 DEPT 135 spectra of compound 108

---

3. Preparation of Starting Materials

3.1 Preparation of Complex Ethers

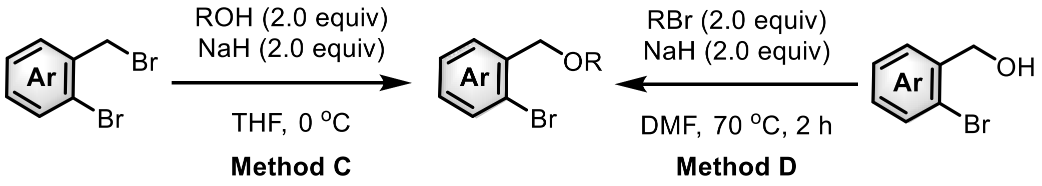

Method C: To a solution of ROH (1.5 equiv) in THF was slowly added NaH (2.0 equiv) at 0 ℃. After stirring for 30 min, benzyl bromide (1.0 equiv) was added. The mixture was then warmed up to r.t. and the resulting reaction mixture was monitored by TLC. The reaction was quenched by H_2O and extracted with Et_2O. The combined organic extracts were washed with brine, dried over anhydrous $MgSO_4$, filtered, concentrated and purified on silica gel chromatography (EtOAc / petroleum ether as eluent) to give the product. The spectroscopic data match the literature report.[11]

Method D: Sodium hydride (60%, 160 mg, 4.0 mmol, 2.0 equiv) was added to a solution of RBr (4 mmol, 2.0 equiv) and 2-bromobenzyl alcohol (2 mmol, 1.0 equiv.) in DMF (5.0 mL). The mixture was heated at 70 ℃ for 2 h. The mixture was cooled to room temperature, then and quenched by water (15 mL) and diethyl ether (20 mL). The organic layer was extracted with water,

and washed with brine, dried over $MgSO_4$ and concentrated in vacuo. The crude product was purified by flash column chromatography.[12]

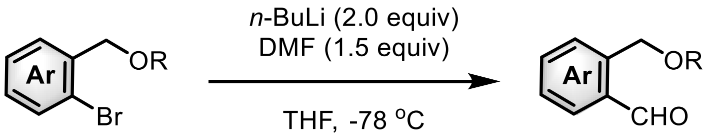

Method E: Under argon, a dry 50 mL round-bottom flask was charged with anhydrous THF (4.0 mL) and 1-bromo-2-(methoxymethyl)benzene (402.0 mg, 2.0 mmol). The reaction mixture was cooled to -78 °C and was slowly added *n*-BuLi (2.4 mmol, 1.6 M in hexanes, 1.5 mL). The reaction was stirred at -78 °C for 30 min and DMF (219.3 mg, 3.0 mmol) was dropwise added. The reaction was allowed to warm to room temperature over 1 h before it was quenched by saturated aqueous NH_4Cl at 0 °C. The reaction mixture was diluted by Et_2O (30 mL), washed with saturated aqueous NH_4Cl (10 mL) and brine (10 mL), dried over Na_2SO_4, and concentrated in vacuo. The crude product was purified by flash column chromatography. The spectroscopic data match the literature report.[13]

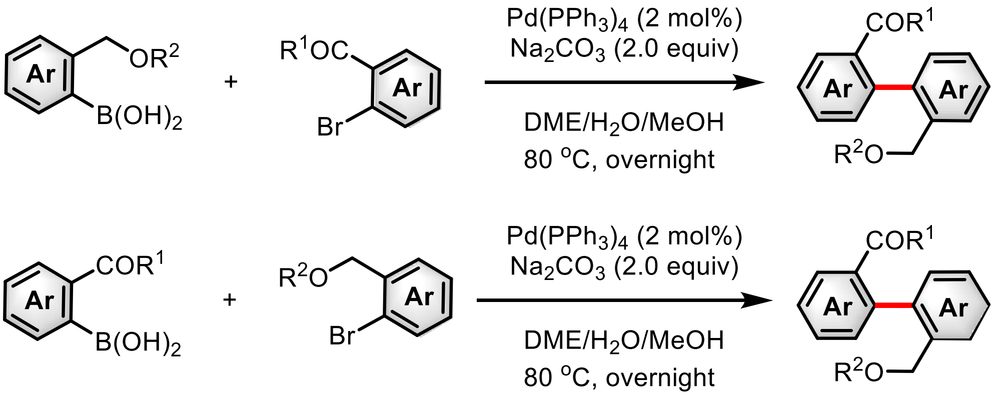

Method F:[14] A Schlenk tube was charged with a solution of 2-bromobenzaldehyde (10.5 mmol) and $Pd(PPh_3)_4$ (346.7 mg, 3 mol%) in DME (25 mL). A solution of Na_2CO_3 (2.2 g, 21.0 mmol) in H_2O (10 mL) and a solution of the corresponding boronic acid (11.5 mmol) in MeOH (8 mL) were sequentially added. The reaction mixture was stirred at 80 ℃ overnight. After cooling to room temperature, the reaction mixture was quenched with H_2O (20 mL) and extracted with CH_2Cl_2 (3 × 20 mL). The combined organic layers were dried over Na_2SO_4, filtered, concentrated, and the residue was purified by silica gel column chromatography (petroleum ether/ethyl acetate = 10:1).

3.2 Preparation of *N*-Triftosylhydrazones

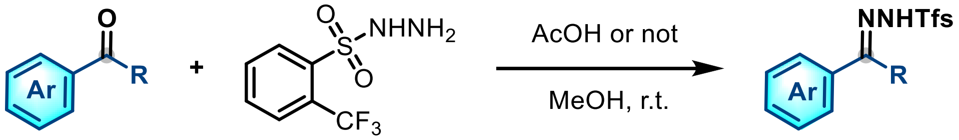

Method G:[15] *N*-Triftosylhydrazones were prepared according to literature procedures developed by our group[1]. To a stirred solution of 2-(trifluoromethyl)benzenesulfonohydrazide (2.0 mmol, 1.0

equiv) in methanol (2.0 mL) were added carbonyl compounds (2.2 mmol, 1.1 equiv). AcOH (one drop) was added when ketone was used. The reaction mixture was stirred for 1-2 h at room temperature. The mixture was concentrated in vacuo and the crude product was purified by column chromatography. Compounds have been confirmed by NMR analysis and matched the reported literature data.

3.3 Characterization data for Unknown Substrates

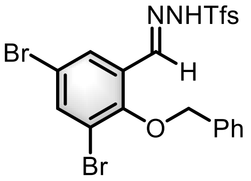

S69

(**S69**) Yellow solid, m.p. 170-172 °C; **^{1}H NMR** (600 MHz, DMSO) δ 12.42 (s, 1H), 8.21 (s, 1H), 8.14 (d, *J* = 7.8 Hz, 1H), 8.01 (d, *J* = 7.8 Hz, 1H), 7.93-7.91 (m, 2H), 7.87 (t, *J* = 7.8 Hz, 1H), 7.65 (d, *J* = 2.4 Hz, 1H), 7.50 (d, *J* = 7.2 Hz, 2H), 7.41 (t, *J* = 7.2 Hz, 2H), 7.38-7.35 (m, 1H), 4.89 (s, 2H); **^{13}C NMR** (151 MHz, DMSO) δ 153.49, 140.51, 138.34, 136.85, 135.96, 134.14, 133.88, 131.76, 131.27, 129.19, 129.03, 128.95 (q, *J* = 6.2 Hz), 128.86, 127.64, 126.81 (q, *J* = 33.0 Hz), 123.20 (q, *J* = 274.2 Hz), 119.57, 117.88, 76.62; **^{19}F NMR** (471 MHz, DMSO) δ -56.27 (s, 3F); **HRMS** (ESI) m/z calcd for $C_{21}H_{15}$ Br_2N_2 F_3O_3S $[M+Na]^+$ 612.9014, Found: 612.9006.

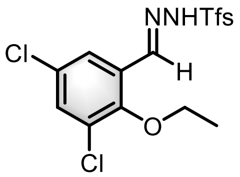

S70

(**S70**) White solid, m.p. 101-103 °C; **^{1}H NMR** (600 MHz, DMSO) δ 12.35 (s, 1H), 8.24 (s, 1H), 8.17 (d, *J* = 8.4 Hz, 1H), 7.99 (d, *J* = 7.8 Hz, 1H), 7.92 (t, *J* = 7.8 Hz, 1H), 7.86 (t, *J* = 7.8 Hz, 1H), 7.59-7.56 (m, 1H), 7.44 (d, *J* = 2.4 Hz, 1H), 3.92 (q, *J* = 7.2 Hz, 2H), 1.32 (t, *J* = 7.2 Hz, 3H); **^{13}C NMR** (151 MHz, DMSO) δ 152.81, 140.95, 138.31, 134.13, 133.87, 131.87, 131.26, 130.65, 129.46, 129.31, 128.92 (q, *J* = 6.2 Hz), 126.85 (q, *J* = 32.8 Hz), 124.03, 123.20 (q, *J* = 274.1 Hz), 71.32, 15.47; **^{19}F NMR** (471 MHz, DMSO) δ -56.38 (s, 3F); **HRMS** (ESI) m/z calcd for $C_{16}H_{13}Cl_2F_3N_2NaO_3S$ $[M+Na]^+$ 462.9868, Found: 462.9873.

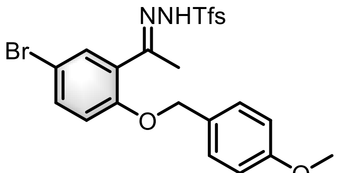

S71

(**S71**) Yellow solid, m.p. 132-133 °C; **^{1}H NMR** (600 MHz, DMSO) δ 11.24 (s, 1H), 8.03-8.00 (m, 2H), 7.88-7.84 (m, 2H), 7.49 (dd, *J* = 10.2 Hz, *J* = 2.4 Hz, 1H), 7.34 (d, *J* = 10.2 Hz, 2H), 7.11 (d, *J* = 10.2 Hz, 1H), 7.08 (d, *J* = 2.4 Hz, 1H), 6.95 (d, *J* = 10.2 Hz, 2H), 5.05 (s, 2H), 3.77 (s, 3H), 2.19 (s, 3H); **^{13}C NMR** (151 MHz, DMSO) δ 159.57, 155.82, 154.36, 138.71, 133.71, 133.52, 133.12, 131.77, 131.09, 130.94, 129.98, 128.85 (q, *J* = 6.0 Hz), 128.64, 127.16 (q, *J* = 32.6 Hz), 123.27 (q, *J* = 274.3 Hz), 115.78, 114.32, 112.16, 70.24, 55.53, 18.67; **^{19}F NMR** (471 MHz, DMSO) δ -56.12 (s, 3F). **HRMS** (ESI) m/z calcd for $C_{23}H_{20}BrF_3N_2NaO_4S$ $[M+Na]^+$ 579.0172, Found: 579.0170.

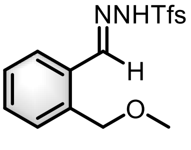

S72

(**S72**) White solid, m.p. 118-119 °C; **^{1}H NMR** (600 MHz, DMSO) δ 12.11 (s, 1H), 8.32 (s, 1H), 8.18 (d, *J* = 7.2 Hz, 1H), 8.00 (d, *J* = 7.2 Hz, 1H), 7.94-7.91 (m, 1H), 7.87-7.81 (m, 1H), 7.60 (d, *J* = 6.6 Hz, 1H), 7.36-7.30 (m, 3H), 4.43 (s, 2H), 3.25 (s, 3H); **^{13}C NMR** (151 MHz, DMSO) δ 146.25, 138.51, 137.30, 133.97, 133.75, 132.18, 131.93, 130.15, 129.58, 128.86 (q, *J* = 6.0 Hz), 128.46, 126.93 (q, *J* = 32.7 Hz), 126.90, 123.27 (q, *J* = 274.1 Hz), 72.18, 58.12; **^{19}F NMR** (471 MHz, DMSO) δ -56.30 (s, 3F); **HRMS** (ESI) m/z calcd for $C_{16}H_{15}F_3N_2NaO_3S$ $[M+Na]^+$ 395.0648, Found: 395.0644.

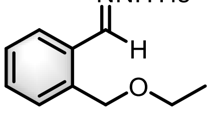

S73

(**S73**) White solid, m.p. 110-111 °C; **^{1}H NMR** (600 MHz, DMSO) δ 12.13 (s, 1H), 8.34 (s, 1H), 8.19 (d, *J* = 7.8 Hz, 1H), 8.00 (d, *J* = 7.8 Hz, 1H), 7.92 (t, *J* = 7.8 Hz, 1H), 7.85 (t, *J* = 7.8 Hz, 1H), 7.61 (d, *J* = 7.2 Hz, 1H), 7.37 (d, *J* = 7.2 Hz, 1H), 7.34 (t, *J* = 7.2 Hz, 1H), 7.30 (t, *J* = 7.8 Hz, 1H), 4.47 (s, 2H), 3.44 (q, *J* = 7.2 Hz, 2H), 1.12 (t, *J* = 7.2 Hz, 3H); **^{13}C NMR** (151 MHz, DMSO) δ

146.28, 138.55, 137.66, 133.93, 133.69, 132.20, 131.91, 130.12, 129.48, 128.83 (q, *J* = 6.1 Hz), 128.33, 126.96 (q, *J* = 32.8 Hz), 126.86, 123.27 (q, *J* = 274.3 Hz), 70.20, 65.71, 15.46.; **^{19}F NMR** (471 MHz, DMSO) δ -56.27 (s, 3F); **HRMS** (ESI) m/z calcd for $C_{17}H_{17}F_3N_2NaO_3S$ $[M+Na]^+$ 409.0804, Found: 409.0811.

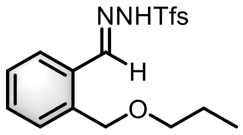

S74

(**S74**) White solid, m.p.106-108 °C; **^{1}H NMR** (600 MHz, DMSO) δ 12.12 (s, 1H), 8.34 (s, 1H), 8.20 (d, *J* = 8.4 Hz, 1H), 7.99 (d, *J* = 7.2 Hz, 1H), 7.91 (t, *J* = 7.2 Hz, 1H), 7.84 (t, *J* = 7.8 Hz, 1H), 7.62 (dd, *J* = 7.8 Hz, *J* = 1.2 Hz, 1H), 7.37 (d, *J* = 7.2 Hz, 1H), 7.33 (td, *J* = 7.2 Hz, *J* = 1.2 Hz, 1H), 7.29 (td, *J* = 7.2 Hz, *J* = 1.2 Hz, 1H), 4.47 (s, 2H), 3.32 (t, *J* = 6.6 Hz, 2H), 1.53-1.47 (m, 2H), 0.83 (t, *J* = 6.6 Hz, 3H); **^{13}C NMR** (151 MHz, DMSO) δ 146.25, 138.57, 137.73, 133.91, 133.66, 132.22, 131.95, 130.10, 129.47, 128.81 (q, *J* = 6.1 Hz), 128.32, 126.96 (q, *J* = 32.8 Hz), 126.90, 123. 28 (q, *J* = 274.1 Hz), 71.92, 70.41, 22.87, 10.94; **^{19}F NMR** (471 MHz, DMSO) δ -56.37 (s, 3F); **HRMS** (ESI) m/z calcd for $C_{18}H_{20}N_2O_3F_3S$ $[M+H]^+$ 401.1147, Found: 401.1144.

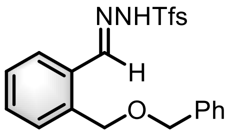

S75

(**S75**) White solid, m.p. 119-120 °C; **^{1}H NMR** (600 MHz, DMSO) δ 12.16 (s, 1H), 8.38 (s, 1H), 8.17 (d, *J* = 7.8 Hz, 1H), 8.00 (d, *J* = 7.2 Hz, 1H), 7.88-7.82 (m, 2H), 7.62 (d, *J* = 7.2 Hz, 1H), 7.42 (d, *J* = 7.8 Hz, 1H), 7.37-7.26 (m, 7H), 4.60 (s, 2H), 4.50 (s, 2H); **^{13}C NMR** (151 MHz, DMSO) δ 146.14, 138.60, 138.50, 137.33, 133.94, 133.69, 132.24, 131.88, 130.20, 129.59, 128.86 (q, *J* = 6.3 Hz), 128.70, 128.49, 128.05, 127.97, 126.97, 126.92 (q, *J* = 32.6 Hz), 123.27 (q, *J* = 274.1 Hz), 72.22, 70.23; **^{19}F NMR** (471 MHz, DMSO) δ -56.28 (s, 3F); **HRMS** (ESI) m/z calcd for $C_{22}H_{20}N_2O_3F_3S$ $[M+H]^+$ 449.1142, Found: 449.1149.

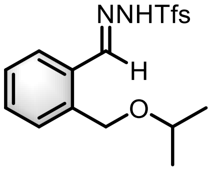

S76

(**S76**) White solid, m.p. 104-105 ºC; **^{1}H NMR** (600 MHz, DMSO) δ 12.16 (s, 1H), 8.32 (s, 1H), 8.18 (d, *J* = 7.8 Hz, 1H), 7.99 (d, *J* = 7.2 Hz, 1H), 7.91 (t, *J* = 7.8 Hz, 1H), 7.84 (t, *J* = 7.8 Hz, 1H), 7.59 (d, *J* = 7.8 Hz, 1H), 7.38 (d, *J* = 7.2 Hz, 1H), 7.32 (t, *J* = 7.2 Hz, 1H), 7.28 (t, *J* = 7.8 Hz, 1H), 4.46 (s, 2H), 3.60-3.54 (m, 1H), 1.09 (d, *J* = 6.6 Hz, 6H); **^{13}C NMR** (151 MHz, DMSO) δ 146.37, 138.58, 138.12, 133.91, 133.67, 132.15, 131.83, 130.09, 129.39, 128.84 (q, *J* = 6.3 Hz), 128.18, 126.98 (q, *J* = 32.9 Hz), 126.96, 123.27 (q, *J* = 274.1 Hz), 71.17, 67.84, 22.36; **^{19}F NMR** (471 MHz, DMSO) δ -56.35 (s, 3F); **HRMS** (ESI) m/z calcd for $C_{18}H_{19}F_3N_2NaO_3S$ [M+Na]$^+$ 423.0961, Found: 423.0965.

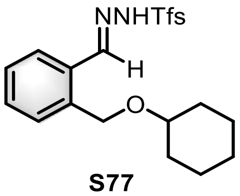

S77

(**S77**) White solid, m.p. 96-97 ºC; **^{1}H NMR** (600 MHz, DMSO) δ 12.12 (s, 1H), 8.30 (s, 1H), 8.13 (d, *J* = 7.8 Hz, 1H), 8.02 (d, *J* = 7.2 Hz, 1H), 7.91 (t, *J* = 7.8 Hz, 1H), 7.87 (t, *J* = 7.8 Hz, 1H), 7.57 (d, *J* = 7.8 Hz, 1H), 7.40 (d, *J* = 7.8 Hz, 1H), 7.35 (t, *J* = 7.2 Hz, 1H), 7.30 (t, *J* = 7.2 Hz, 1H), 4.48 (s, 2H), 3.28-3.24 (m, 1H), 1.83-1.75 (m, 2H), 1.67-1.60 (m, 2H), 1.50-1.45 (m, 1H), 1.24-1.15 (m, 5H); **^{13}C NMR** (151 MHz, DMSO) δ 146.50, 138.50, 138.28, 133.96, 133.73, 132.06, 131.73, 130.14, 129.30, 128.89 (q, *J* = 6.0 Hz), 128.18, 127.10, 126.96 (q, *J* = 33.1 Hz), 123.25 (q, *J* = 274.1 Hz), 76.84, 67.53, 32.08, 25.83, 23.93; **^{19}F NMR** (471 MHz, DMSO) δ -56.30 (s, 3F); **HRMS** (EI) m/z calcd for $C_{21}H_{23}F_3N_2NaO_3S$ [M+Na]$^+$ 463.1274, Found: 463.1275.

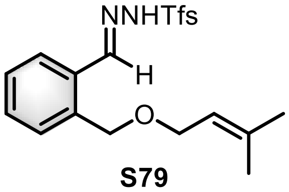

S79

(**S79**) White solid, m.p. 96-97 ºC; **^{1}H NMR** (600 MHz, DMSO) δ 11.12 (s, 1H), 8.34 (s, 1H), 8.18 (d, *J* = 7.8 Hz, 1H), 7.99 (d, *J* = 7.8 Hz, 1H), 7.91 (t, *J* = 7.8 Hz, 1H), 7.84 (t, *J* = 7.8 Hz, 1H), 7.60 (d, *J* = 7.8 Hz, 1H), 7.36 (d, *J* = 7.2 Hz, 1H), 7.33 (td, *J* = 7.2 Hz, *J* = 1.2 Hz, 1H), 7.29 (t, *J* = 7.2 Hz, 1H), 5.30 (t, *J* = 6.6 Hz, 1H), 4.47 (s, 2H), 3.92 (d, *J* = 7.2 Hz, 2H), 1.68 (s, 3H), 1.57 (s, 3H); **^{13}C NMR** (151 MHz, DMSO) δ 146.19, 138.58, 137.67, 136.41, 133.90, 133.65, 132.22, 131.91, 130.11, 129.57, 128.81 (q, *J* = 6.1 Hz), 128.33, 126.91 (q, *J* = 33.3 Hz), 126.84, 123.27 (q, *J* = 274.1 Hz), 121.68, 69.57, 66.72, 25.83, 18.22; **^{19}F NMR** (471 MHz, DMSO) δ -56.34 (s, 3F). **HRMS** (ESI) m/z calcd for $C_{20}H_{21}F_3N_2NaO_3S$ [M+Na]$^+$ 449.1118, Found: 449.1129.

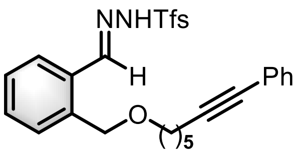

S80

(**S80**) Yellow solid, m.p. 70-71 ^oC; **^{1}H NMR** (500 MHz, DMSO) δ 12.13 (s, 1H), 8.32 (s, 1H), 8.18 (d, *J* = 7.5 Hz, 1H), 7.98 (d, *J* = 7.5 Hz, 1H), 7.90 (t, *J* = 7.5 Hz, 1H), 7.82 (t, *J* = 7.5 Hz, 1H), 7.60 (d, *J* = 7.0 Hz, 1H), 7.39-7.25 (m, 8H), 4.47 (s, 2H), 3.39 (t, *J* = 6.5 Hz, 2H), 2.39 (t, *J* = 6.5 Hz, 2H), 1.57-1.49 (m, 4H), 1.46-1.41 (m, 2H); **^{13}C NMR** (151 MHz, DMSO) δ 146.25, 138.54, 137.72, 133.93, 133.69, 132.12, 131.95, 131.68, 130.12, 129.38, 128.98, 128.84 (q, *J* = 6.0 Hz), 128.31, 128.28, 126.99, 126.97 (q, *J* = 32.9 Hz), 123.84, 123.46 (q, *J* = 274.2 Hz), 91.06, 81.09, 70.40, 70.20, 29.13, 28.48, 25.50, 19.08; **^{19}F NMR** (471 MHz, DMSO) δ -56.32 (s, 3F). **HRMS** (ESI) m/z calcd for $C_{28}H_{28}F_3N_2O_3S$ $[M+H]^+$ 529.1773, Found: 529.1779.

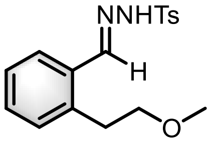

S81

(**S81**) White solid, m.p. 110-111 ^oC; **^{1}H NMR** (600 MHz, DMSO) δ 11.99 (s, 1H), 8.37 (s, 1H), 8.18 (d, *J* = 8.4 Hz, 1H), 7.99 (d, *J* = 7.8 Hz, 1H), 7.91 (t, *J* = 7.8 Hz, 1H), 7.84 (t, *J* = 7.8 Hz, 1H), 7.54 (d, *J* = 7.8 Hz, 1H), 7.28 (t, *J* = 7.2 Hz, 1H), 7.22-7.18 (m, 2H), 3.38 (t, *J* = 7.2 Hz, 2H), 3.17 (s, 3H), 2.90 (t, *J* = 7.2 Hz, 2H); **^{13}C NMR** (151 MHz, DMSO) δ 146.62, 138.56, 138.48, 133.91, 133.70, 132.12, 131.89, 131.45, 130.28, 128.84 (q, *J* = 6.3 Hz), 127.07, 126.95 (q, *J* = 32.7 Hz), 126.94, 123.28 (q, *J* = 274.2 Hz), 72.94, 58.34, 32.96; **^{19}F NMR** (471 MHz, DMSO) δ -56.34 (s, 3F); **HRMS** (ESI) m/z calcd for $C_{17}H_{17}F_3N_2NaO_3S$ $[M+Na]^+$ 409.0804, Found: 409.0806.

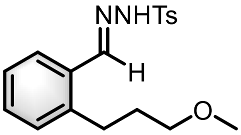

S82

(**S82**) White solid, m.p. 98-99 ^oC; **^{1}H NMR** (600 MHz, DMSO) δ 12.00 (s, 1H), 8.35 (s, 1H), 8.15 (d, *J* = 7.8 Hz, 1H), 7.99 (d, *J* = 7.8 Hz, 1H), 7.91 (t, *J* = 7.8 Hz, 1H), 7.84 (t, *J* = 7.8 Hz, 1H), 7.53 (d, *J* = 7.8 Hz, 1H), 7.28 (t, *J* = 7.2 Hz, 1H), 7.18 (t, *J* = 7.8 Hz, 2H), 3.22 (t, *J* = 6.6 Hz, 2H), 3.19 (s, 3H), 2.69 (t, *J* = 7.2 Hz, 2H), 1.66-1.61 (m, 2H); **^{13}C NMR** (151 MHz, DMSO) δ 146.46, 141.39, 138.58, 133.91, 133.70, 131.74, 131.61, 130.73, 130.40, 128.86 (q, *J* = 6.0 Hz), 126.96, 126.91 (q, *J* = 33.1 Hz), 126.78, 123.27 (q, *J* = 274.1 Hz), 71.42, 58.22, 31.41, 29.32; **^{19}F NMR**

(471 MHz, DMSO) δ -56.31 (s, 3F); **HRMS** (ESI) m/z calcd for $C_{18}H_{19}F_3N_2NaO_3S[M+Na]^+$ 423.0961, Found: 423.0961.

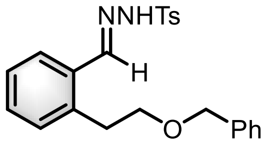

S83

(**S83**) White solid, m.p. 89-90 °C; **^{1}H NMR** (600 MHz, DMSO) δ 12.00 (s, 1H), 8.40 (s, 1H), 8.17 (d, *J* = 7.8 Hz, 1H), 7.98 (d, *J* = 7.2 Hz, 1H), 7.87 (t, *J* = 7.8 Hz, 1H), 7.83 (t, *J* = 7.8 Hz, 1H), 7.55 (d, *J* = 7.8 Hz, 1H), 7.32-7.28 (m, 3H), 7.26-7.20 (m, 5H), 4.42 (s, 2H), 3.53 (t, *J* = 6.6 Hz, 2H), 2.98 (t, *J* = 6.6 Hz, 2H); **^{13}C NMR** (151 MHz, DMSO) δ 146.54, 138.88, 138.54, 138.49, 133.91, 133.68, 132.12, 131.84, 131.47, 130.28, 128.85 (q, *J* = 6.1 Hz), 128.63, 127.73, 127.63, 127.10, 126.91, 126.90 (q, *J* = 32.5 Hz), 123.26 (q, *J* = 274.1 Hz), 72.28, 70.75, 33.09; **^{19}F NMR** (471 MHz, DMSO) δ -56.28 (s, 3F); **HRMS** (ESI) m/z calcd for $C_{23}H_{21}F_3N_2NaO_3S$ $[M+Na]^+$ 485.1117, Found: 485.1123.

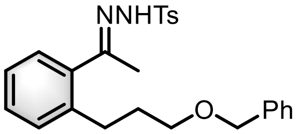

S84

(**S84**) Yellow oil; **^{1}H NMR** (600 MHz, DMSO) δ 12.04 (s, 1H), 8.39 (s, 1H), 8.18 (d, *J* = 7.8 Hz, 1H), 7.97 (d, *J* = 7.8 Hz, 1H), 7.87 (t, *J* = 7.8 Hz, 1H), 7.81 (t, *J* = 7.8 Hz, 1H), 7.56 (d, *J* = 7.8 Hz, 1H), 7.35-7.31 (m, 4H), 7.28-7.24 (m, 2H), 7.19-7.15 (m, 2H), 4.42 (s, 2H), 3.35 (t, *J* = 6.6 Hz, 2H), 2.74 (t, *J* = 7.2 Hz, 2H), 1.73-1.68 (m, 2H); **^{13}C NMR** (151 MHz, DMSO) δ 146.51, 141.35, 139.04, 138.62, 133.85, 133.64, 131.78, 131.62, 130.75, 130.36, 128.84 (q, *J* = 6.2 Hz), 128.69, 127.97, 127.82, 127.04, 126.95 (q, *J* = 33.0 Hz), 126.78, 123.29 (q, *J* = 274.1 Hz), 72.41, 69.27, 31.55, 29.47; **^{19}F NMR** (471 MHz, DMSO) δ -56.31 (s, 3F); **HRMS** (ESI) m/z calcd for $C_{24}H_{23}F_3N_2NaO_3S$ $[M+Na]^+$ 499.1274, Found:499.1277.

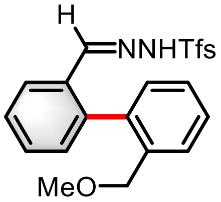

S85

(**S85**) White solid, m.p. 80-81 ºC; **^{1}H NMR** (600 MHz, DMSO) δ 11.87 (s, 1H), 8.11 (d, *J* = 7.8 Hz, 1H), 7.98 (d, *J* = 7.8 Hz, 1H), 7.91 (t, *J* = 7.8 Hz, 1H), 7.85 (t, *J* = 7.8 Hz, 1H), 7.76-7.75 (m, 1H), 7.69 (s, 1H), 7.51 (d, *J* = 7.8 Hz, 1H), 7.46-7.37 (m, 4H), 7.17 (dd, *J* = 7.8 Hz, *J* = 1.2 Hz, 1H), 7.10-7.08 (m, 1H), 4.02 (ABq, *J* = 12.0 Hz, 2H), 3.03 (s, 3H); **^{13}C NMR** (151 MHz, DMSO) δ 145.61, 140.75, 138.54, 138.48, 136.65, 133.93, 133.69, 131.96, 131.86, 130.56, 130.45, 130.07, 128.80 (q, *J* = 6.0 Hz), 128.78, 128.39, 128.32, 127.93, 126.85 (q, *J* = 33.1 Hz), 124.95, 123.20 (q, *J* = 274.1 Hz), 71.89, 57.99; **^{19}F NMR** (471 MHz, DMSO) δ -56.34 (s, 3F); **HRMS** (EI) m/z calcd for $C_{22}H_{19}F_3N_2NaO_3S$ $[M+Na]^+$ 471.0961, Found: 471.0969.

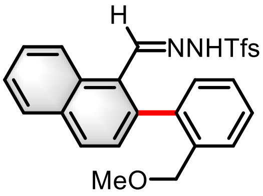

S86

(**S86**) White solid, m.p. 160-161 ºC; **^{1}H NMR** (600 MHz, DMSO) δ 11.96 (s, 1H), 8.15 (d, *J* = 7.8 Hz, 1H), 7.99 (d, *J* = 7.8 Hz, 1H), 7.94-7.87 (m, 4H), 7.85 (t, *J* = 7.8 Hz, 1H), 7.76 (s, 1H), 7.62 (d, *J* = 7.8 Hz, 1H), 7.56 (t, *J* = 7.8 Hz, 1H), 7.51 (t, *J* = 7.2 Hz, 1H), 7.48 (t, *J* = 7.8 Hz, 1H), 7.39 (t, *J* = 7.8 Hz, 1H), 7.15 (d, *J* = 7.2 Hz, 1H), 7.12 (d, *J* = 7.4 Hz, 1H), 3.85 (ABq, *J* = 12.6 Hz, 1H), 2.91 (s, 3H); **^{13}C NMR** (151 MHz, DMSO) δ 145.88, 138.62, 138.53, 137.65, 135.60, 133.96, 133.71, 132.33, 131.89, 131.09, 129.65, 128.82 (q, *J* = 5.9 Hz), 128.81, 128.62, 128.58, 128.22, 127.51, 127.41, 126.84 (q, *J* = 32.7 Hz), 126.24, 123.19 (q, *J* = 274.0 Hz), 121.84, 71.75, 57.92; **^{19}F NMR** (471 MHz, DMSO) δ -56.30 (s, 3F); **HRMS** (ESI) m/z calcd for $C_{26}H_{21}F_3N_2NaO_3S$ $[M+Na]^+$ 521.1117, Found:521.1121.

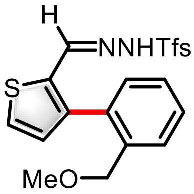

S87

(**S87**) White solid, m.p. 127-129 ºC; **^{1}H NMR** (600 MHz, DMSO) δ 11.70 (s, 1H), 7.97 (d, *J* = 7.2 Hz, 1H), 7.92-7.91 (m, 1H), 7.87-7.83 (m, 3H), 7.69-7.68 (m, 1H), 7.48-7.44 (m, 2H), 7.40 (t, *J* = 7.2 Hz, 1H), 7.33 (t, *J* = 7.2 Hz, 1H), 7.10 (d, *J* = 7.2 Hz, 1H), 4.10 (s, 2H), 3.08 (s, 3H); **^{13}C NMR** (151 MHz, DMSO) δ 142.32, 139.94, 138.43, 137.31, 134.91, 134.78, 133.81, 133.66, 131.84, 130.69, 128.76, 128.73 (q, *J* = 6.1 Hz), 128.29, 127.74, 126.68 (q, *J* = 32.8 Hz), 125.94, 125.86, 123.20 (q, *J* = 274.1 Hz), 71.97, 58.02; **^{19}F NMR** (471 MHz, DMSO) δ -56.29 (s, 3F); **HRMS** (EI) m/z calcd for $C_{20}H_{17}N_2NaO_3S_2$ $[M+Na]^+$ 447.0525, Found: 477.0534.

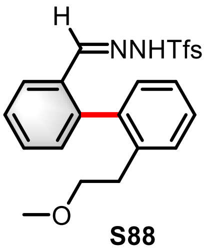

S88

(**S88**) White solid, m.p. 129-130 °C; **^{1}H NMR** (600 MHz, DMSO) δ 11.92 (s, 1H), 8.13 (d, *J* = 7.8 Hz, 1H), 7.98 (d, *J* = 7.2 Hz, 1H), 7.90 (t, *J* = 7.8 Hz, 1H), 7.84 (t, *J* = 7.8 Hz, 1H), 7.77 (d, *J* = 7.8 Hz, 1H), 7.73 (s, 1H), 7.43-7.36 (m, 4H), 7.29 (t, *J* = 7.8 Hz, 1H), 7.17 (d, *J* = 7.8 Hz, 1H), 7.05 (d, *J* = 7.8 Hz, 1H), 3.25 (t, *J* = 7.2 Hz, 2H), 2.99 (s, 3H), 2.59-2.54 (m, 1H), 2.49-2.44 (m, 1H); **^{13}C NMR** (151 MHz, DMSO) δ 145.66, 141.61, 139.13, 138.56, 137.34, 133.89, 133.65, 131.98, 131.84, 130.82, 130.43, 130.13, 128.78 (q, *J* = 6.2 Hz), 128.43, 128.19, 126.87 (q, *J* = 32.6 Hz), 126.61, 124.95, 123.20 (q, *J* = 274.1 Hz), 72.25, 57.97, 33.03; **^{19}F NMR** (471 MHz, DMSO) δ -56.35 (s, 3F); **HRMS** (ESI) m/z calcd for $C_{23}H_{22}N_2O_3F_3S$ $[M+H]^+$ 463.1303, Found: 463.1310.

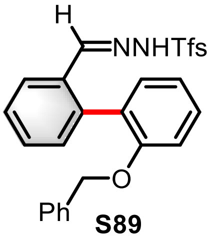

S89

(**S89**) White solid, m.p. 70-71 °C; **^{1}H NMR** (500 MHz, DMSO) δ 12.05 (s, 1H), 8.20 (d, *J* = 8.0 Hz, 1H), 7.98 (s, 1H), 7.95 (d, *J* = 7.5 Hz, 1H), 7.86-7.79 (m, 3H), 7.45-7.39 (m, 2H), 7.33 (t, *J* = 7.5 Hz, 1H), 7.26-7.22 (m, 7H), 7.16 (d, *J* = 7.5 Hz, 1H), 7.09 (t, *J* = 7.5 Hz, 1H), 5.08 (s, 2H); **^{13}C NMR** (126 MHz, DMSO) δ 155.74, 146.72, 139.30, 138.62, 137.32, 133.90, 133.67, 132.18, 132.07, 131.80, 131.29, 130.37, 130.07, 128.87 (q, *J* = 6.5 Hz), 128.78, 128.46, 128.16, 128.07, 127.81, 126.91 (q, *J* = 32.9 Hz), 124.60, 123.34 (q, *J* = 274.7 Hz), 121.51, 113.15, 69.99; **^{19}F NMR** (471 MHz, DMSO) δ -56.31 (s, 3F); **HRMS** (ESI) m/z calcd for $C_{27}H_{21}F_3N_2NaO_3S$ $[M+Na]^+$ 533.1117, Found: 533.1124.

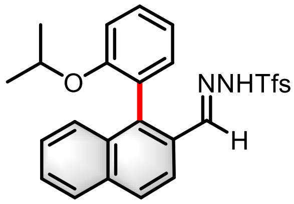

S91

(**S91**) White solid, m.p. 149-150 °C; **^{1}H NMR** (600 MHz, DMSO) δ 11.37 (s, 1H), 8.07-8.05 (m, 2H), 7.98 (d, *J* = 8.4 Hz, 1H), 7.95-7.91 (m, 2H), 7.88 (t, *J* = 7.8 Hz, 1H), 7.57 (s, 1H), 7.53 (t, *J* = 7.8 Hz, 1H), 7.45 (t, *J* = 7.8 Hz, 1H), 7.39-7.36 (m, 2H), 7.30 (d, *J* = 7.2 Hz, 1H), 7.21 (d, *J* = 7.2 Hz, 1H), 6.99 (t, *J* = 7.8 Hz, 1H), 6.78 (d, *J* = 8.4 Hz, 1H), 3.98-3.92 (m, 1H), 0.89 (d, *J* = 6.0 Hz, 3H), 0.72 (d, *J* = 6.0 Hz, 3H); **^{13}C NMR** (151 MHz, DMSO) δ 155.06, 148.52, 138.89, 136.30, 134.24, 133.76, 133.59, 132.34, 132.11, 131.83, 131.13, 130.35, 130.15, 129.88, 128.75 (q, *J* = 5.8 Hz), 128.73, 127.55, 127.00 (q, *J* = 32.8 Hz), 125.99, 125.58, 123.27 (q, *J* = 274.2 Hz), 120.85, 113.19, 70.13, 22.67, 21.89; **^{19}F NMR** (471 MHz, DMSO) δ -56.14 (s, 3F); **HRMS** (ESI) m/z calcd for $C_{27}H_{23}F_3N_2NaO_3S$ $[M+Na]^+$ 535.1274, Found: 535.1282.

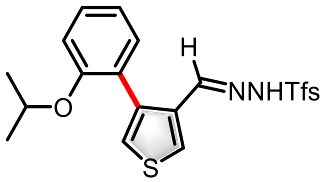

S92

(**S92**) White solid, m.p. 143-144 °C; **^{1}H NMR** (600 MHz, DMSO) δ 11.68 (s, 1H), 8.09 (d, *J* = 7.8 Hz, 1H), 7.97 (d, *J* = 7.8 Hz, 1H), 7.88 (t, *J* = 7.8 Hz, 1H), 7.83 (t, *J* = 7.8 Hz, 1H), 7.77 (d, *J* = 3.6 Hz, 1H), 7.72 (s, 1H), 7.38 (d, *J* = 3.6 Hz, 1H), 7.37-7.34 (m, 1H), 7.17 (dd, *J* = 7.2 Hz, *J* = 1.8 Hz, 1H), 7.05 (d, *J* = 8.4 Hz, 1H), 6.97 (t, *J* = 7.8 Hz, 1H), 4.52-4.45 (m, 1H), 1.05 (d, *J* = 6.0 Hz, 6H); **^{13}C NMR** (151 MHz, DMSO) 154.95, 143.38, 139.53, 138.55, 135.04, 133.78, 133.60, 132.14, 131.99, 129.91, 128.68 (q, *J* = 6.0 Hz), 126.78 (q, *J* = 32.5 Hz), 125.25, 125.01, 124.26, 123.26 (q, *J* = 274.2 Hz), 120.76, 113.84, 69.92, 22.03; **^{19}F NMR** (471 MHz, DMSO) δ -56.24 (s, 3F); **HRMS** (ESI) m/z calcd for $C_{21}H_{19}F_3N_2NaO_3S_2$ $[M+Na]^+$ 491.0681, Found: 491.0687.

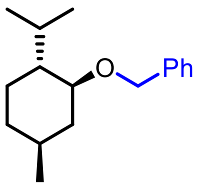

S98

(**S98**) Colorless oil; ^{1}**H NMR** (600 MHz, $CDCl_3$) δ 7.34-7.30 (m, 4H), 7.24 (t, *J* = 7.2 Hz, 1H), 4.64 (d, *J* = 14.4 Hz, 1H), 4.39 (d, *J* = 14.4 Hz, 1H), 3.16 (td, *J* = 10.8 Hz, *J* = 4.2 Hz, 1H), 2.33-2.27 (m, 1H), 2.20-2.17 (m, 1H), 1.66-1.60 (m, 2H), 1.38-1.29 (m, 2H), 0.98-0.85 (m, 9H), 0.71 (d, *J* = 6.6 Hz, 3H); ^{13}**C NMR** (151 MHz, $CDCl_3$) δ 139.13, 128.27, 127.83, 127.37, 78.71, 70.41, 48.32, 40.30, 34.58, 31.57, 25.50, 23.24, 22.37, 21.02, 16.03; **HRMS** (ESI) m/z calcd for $C_{17}H_{27}O$ $[M+H]^+$ 247.2062, Found: 247.2061.

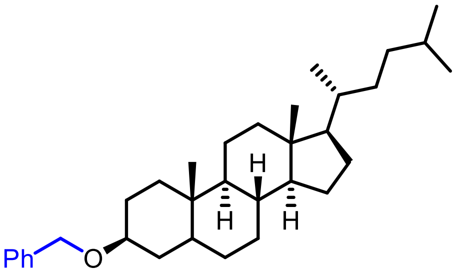

s99

(**S99**) White solid, m.p. 103-104 °C; ^{1}**H NMR** (600 MHz, $CDCl_3$) δ 7.35-7.31 (m, 4H), 7.27-7.24 (m, 1H), 4.57-4.52 (m, 2H), 3.35-3.30 (m, 1H), 1.96 (dt, *J* = 12.6 Hz, *J* = 3.6 Hz, 1H), 1.92-1.88 (m, 1H), 1.83-1.77 (m, 1H), 1.72 (dt, *J* = 12.6 Hz, *J* = 3.6 Hz, 1H), 1.68-1.63 (m, 2H), 1.57-1.41 (m, 4H), 1.37-1.20 (m, 9H), 1.16-0.93 (m, 9H), 0.90 (d, *J* = 6.6 Hz, 3H), 0.87-0.85 (m, 6H), 0.81 (s, 3H), 0.64 (s, 3H), 0.62-0.58 (m, 1H); ^{13}**C NMR** (151 MHz, $CDCl_3$) δ 139.22, 128.29, 127.51, 127.29, 78.04, 69.76, 56.50, 56.29, 54.42, 44.86, 42.59, 40.05, 39.51, 37.02, 36.17, 35.78, 35.49, 34.84, 32.12, 28.87, 28.29, 28.24, 28.00, 24.21, 23.83, 22.81, 22.55, 21.23, 18.66, 12.28, 12.06; **HRMS** (ESI) m/z calcd for $C_{33}H_{53}O$ $[M+H]^+$ 465.4096, Found: 465.4090.

---

4. Detailed Optimization of Conditions

Supplementary Table 1 Optimization of reaction conditions

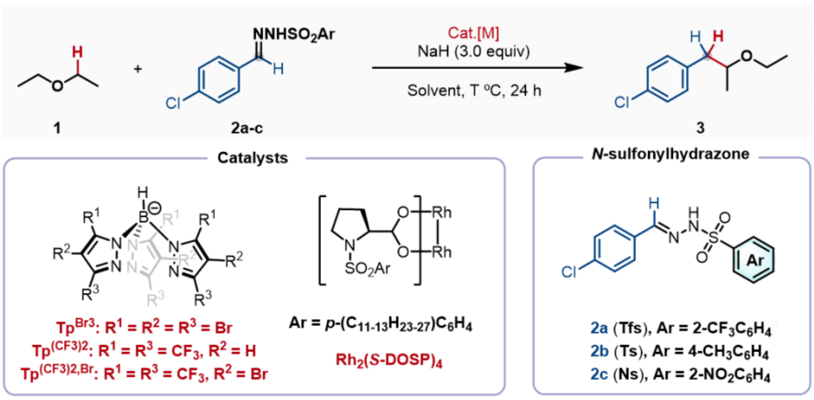

**Entry**	**2**	**[Cat.]** (mol%)	**Et_2O**	**Solvent**	***T*** (℃)	Yield of **3**
1[a]	**2a**	Tp^{Br3}Ag(thf) (5)	5.0 mL	CH_2Cl_2	60	54%
2[a]	**2a**	Tp^{Br3}Ag(thf) (5)	1.0 mL	CH_2Cl_2	60	75%
3	**2a**	Tp^{Br3}Ag(thf) (10)	1.0 mL	CH_2Cl_2	60	85%
4	**1a**	Tp^{Br3}Ag(thf) (10)	1.0 mL	$CHCl_3$	60	99% (96%)[b]
5	**2a**	Tp^{Br3}Ag(thf) (10)	1.0 mL	DCE	60	90%
6	**2a**	Tp^{Br3}Ag(thf) (10)	1.0 mL	$PhCF_3$	60	95%
7	**2a**	$Tp^{(CF3)2,Br}$Ag(thf) (10)	1.0 mL	$CHCl_3$	60	83%
8	**2a**	$Tp^{(CF3)2}$Ag(thf) (10)	1.0 mL	$CHCl_3$	60	92%
8	**2a**	AgOTf (10)	1.0 mL	$CHCl_3$	60	70%
9	**2a**	Tp^{Br3} Cu(NCMe) (10)	1.0 mL	$CHCl_3$	60	trace
10	**2a**	Rh_2(*S*-DOSP)$_4$ (2)	1.0 mL	$CHCl_3$	60	trace
11	**2a**	Tp^{Br3}Ag(thf) (10)	1.0 mL	$CHCl_3$	30	30%
12	**2a**	Tp^{Br3}Ag(thf) (10)	1.0 mL	$CHCl_3$	80	80%
13	**2b**	Tp^{Br3}Ag(thf) (10)	1.0 mL	$CHCl_3$	60	35%
14	**2c**	Tp^{Br3}Ag(thf) (10)	1.0 mL	$CHCl_3$	60	33%
15[d]	**2a**	Tp^{Br3}Ag(thf) (10)	2.0 equiv.	$CHCl_3$	60	75%

Reaction conditions: *N*-Sulfonylhydrazone **1a-1c** (0.3 mmol), NaH (0.9 mmol, 3.0 equiv), Et_2O (1.0 mL), catalyst, and solvent (5.0 mL), under argon at 60 ℃ for 24 h unless otherwise mentioned. Yields were determined by ^1H NMR with dibromomethane as the internal standard. [a] NaH (0.6 mmol, 2.0 equiv) was used. [b] The isolated yield is given in parentheses.

5. Competition Experiments

5.1 Competition Experiments Between α-C−H Bonds of Ether and other C(sp^3)−H Bonds

To an oven-dried sealed tube was charged with *N*-triftosylhydrazone **2a** (108.8 mg, 0.3 mmol), Tp^{Br3}Ag(thf) (32.7 mg, 10 mol%), NaH (36.0 mg, 0.9 mmol, 60 wt% dispersion in mineral oil) in an argon-filled glovebox. Anhydrous $CHCl_3$ (5.0 mL) and Et_2O (20.0 equiv) and other ether / alkane (20.0 equiv) were added. The tube was sealed and rinsed in an ultrasonic bath for 5 min. The resulting mixture was stirred (700 rpm) at 60 ℃ for 24 h. When the reaction was completed, the crude reaction mixture was allowed to reach room temperature and filtered through a short pad of silica gel with EtOAc as eluent. The filtrate was concentrated in vacuo to leave a crude mixture, which was determined by ^1H NMR to measure the product distribution. The result is the average of three parallel experiments.

5.2 Competition Experiments of Different Types of *N*-triftosylhydrazones

To an oven-dried sealed tube was charged with *N*-triftosylhydrazone **2a** (54.4 mg, 0.15 mmol) and **3b** / **4b** / **5b** (0.15 mmol). Tp^{Br3}Ag(thf) (32.7 mg, 10 mol%), NaH (36.0 mg, 0.9 mmol, 60 wt% dispersion in mineral oil) in an argon-filled glovebox. Anhydrous $CHCl_3$ (5.0 mL) and Et_2O (1 mL) were added. The tube was sealed and rinsed in an ultrasonic bath for 5 min. The resulting mixture was stirred (700 rpm) at 60 ℃ for 24 h. When the reaction was completed, the crude reaction mixture was allowed to reach room temperature and filtered through a short pad of silica

gel with EtOAc as eluent. The filtrate was concentrated in vacuo to leave a crude mixture, which was determined by ^{1}H NMR to measure the product distribution. The result is the average of three parallel experiments.

5.3 Competition Experiments between Ether and Product 3

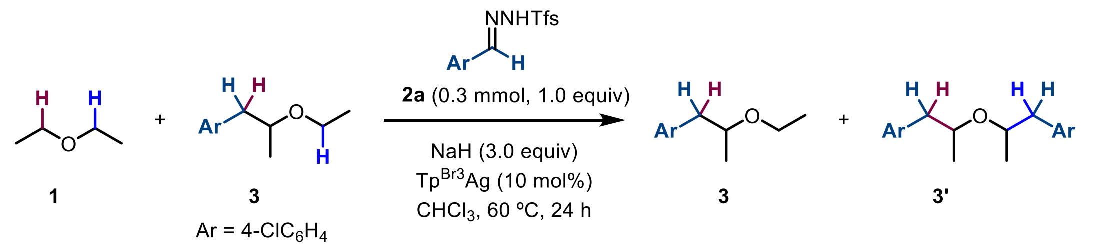

Entry	Reactant		Product (NMR yield)	
	**1**	**3**	**3**	**3'**
1	2.0 equiv.	0 equiv.	75% (newly)	<5%
2	2.0 equiv.	1.0 equiv.	100% (recovery) + 70% (newly)	5-10%
3	0 equiv	2.0 equiv.	175-180% (recovery)	20-25%
4	20.0 equiv.	1.0 equiv	100% (newly)	not detected

Supplementary Scheme 1. Competition Experiments between Ether and Product **3**.

Procedure: To an oven-dried sealed tube was charged with *N*-triftosylhydrazone **2a** (108.8 mg, 0.3 mmol), Tp^{Br3}Ag(thf) (32.7 mg, 10 mol%), NaH (36.0 mg, 0.9 mmol, 60 wt% dispersion in mineral oil) in an argon-filled glovebox. Anhydrous $CHCl_3$ (5.0 mL) and Et_2O (20.0 / 2.0 / 0 equiv) and compound **3** (1.0 equiv) were added. The tube was sealed and rinsed in an ultrasonic bath for 5 min. The resulting mixture was stirred (700 rpm) at 60 ℃ for 24 h. When the reaction was completed, the crude reaction mixture was allowed to reach room temperature and filtered through a short pad of silica gel with EtOAc as eluent. The filtrate was concentrated in vacuo to leave a crude mixture, which was determined by ^{1}H NMR to measure the product distribution.

The obtained results show that over-insertion of C–H bonds on the other side of the oxygen could indeed when 2.0 equiv diethyl ether was used (entries 1 and 2).The structure of over insertion product **3'** was confirmed by H NMR and HRMS, although it can not be isolated from compound **3** (entry 3). However, the relative reactivity of α-C–H bond in compound **3** is much lower than α-C–H bond of diethyl ether. Fortunately, over functionalization could be avoided by improving the concentration of substrate ethers (entry 4). A probable explanation is that compound **3** has fewer α-C–H bonds and more steric hindrance compared to diethyl ether. But, the exact reason for these unique reactivity differences has not been fully explored at this point.

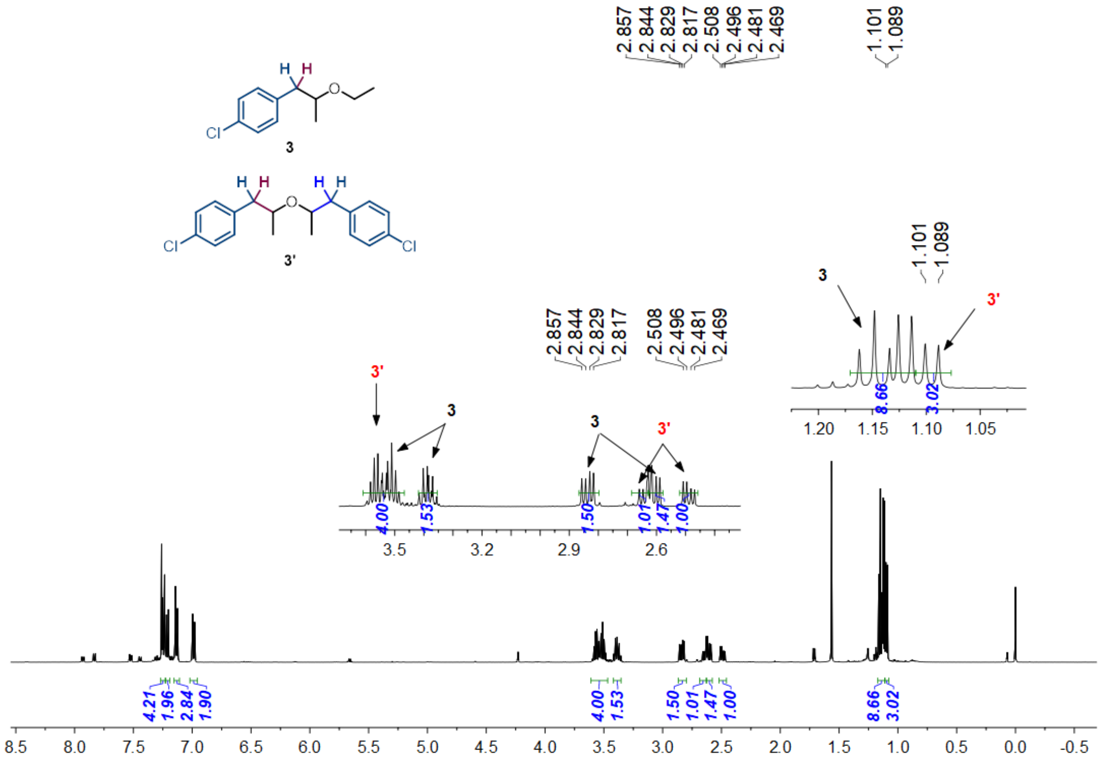

Supplementary Fig. 3 ^{1}H NMR (600 MHz, $CDCl_3$) spectra of compound **3** and **3'**.

Elemental Composition Report **Page 1**

Single Mass Analysis

Tolerance = 5.0 PPM / DBE: min = -1.5, max = 50.0

Element prediction: Off

Number of isotope peaks used for i-FIT = 3

Monoisotopic Mass, Even Electron Ions

41 formula(e) evaluated with 1 results within limits (up to 50 best isotopic matches for each mass)

Elements Used:

C: 1-18 H: 1-50 O: 1-4 Na: 0-1 Cl: 1-2

5200 123 (1.128)

1: TOF MS ES+

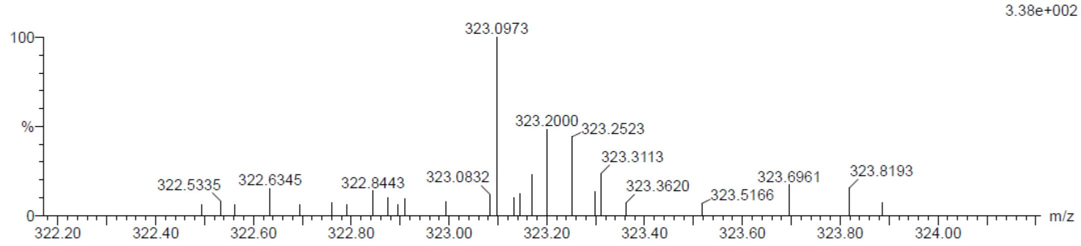

Minimum:				-1.5				
Maximum:		5.0	5.0	50.0				
Mass	Calc. Mass	mDa	PPM	DBE	i-FIT	Norm	Conf(%)	Formula
323.0973	323.0969	0.4	1.2	7.5	117.0	n/a	n/a	C18 H21 O Cl2

Supplementary Fig. 4 HRMS (ESI) spectra of compound **3'**. (m/z calcd for $C_{18}H_{21}OCl_{12}$ [M+H]$^+$ 2323.0969, Found: 323.0973).

6. Substrates Ineffective for α-C-H Insertion of Ethers

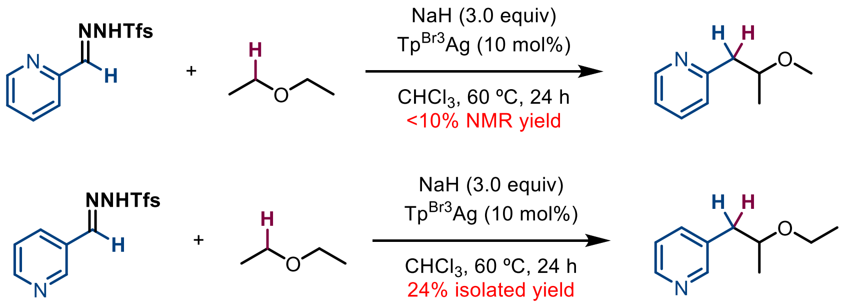

Supplementary Fig. 4 More substrates tested for α-C–H insertion of ethers

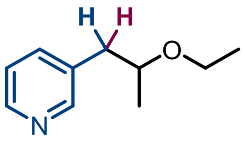

Yellow oil; **^{1}H NMR** (500 MHz, $CDCl_3$) δ 8.47-8.44 (m, 2H), 7.56 (d, J = 8.0 Hz, 1H), 7.22 (dd, J = 8.0 Hz, J = 4.5 Hz, 1H), 3.62-3.51 (m, 2H), 3.39-3.33 (m, 1H), 2.82 (dd, J = 14.0 Hz, J = 7.0 Hz, 1H), 2.68 (dd, J = 14.0 Hz, J = 5.5 Hz, 1H), 1.16-1.12 (m, 6H); **^{13}C NMR** (126 MHz, $CDCl_3$) δ 150.58, 147.42, 137.08, 134.60, 123.10, 75.68, 64.04, 40.13, 19.60, 15.43; **HRMS** (ESI) m/z calcd for $C_{10}H_{16}NO$ $[M+H]^+$ 166.2436, Found: 1662.2441.

7. Mechanistic Studies

7.1 KIE Experiment

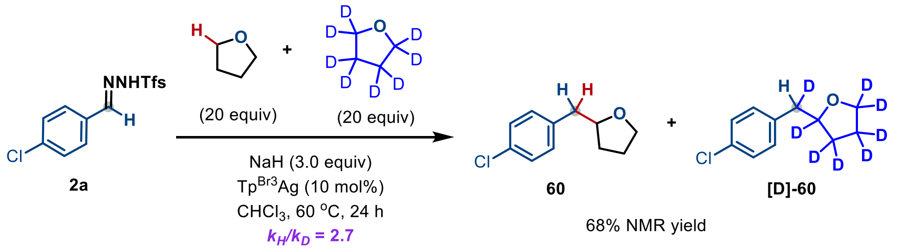

Supplementary Scheme 2 KIE experiment

To a flame-dried sealed screw-cap tube were added *N*-triftosylhydrazone **2a** (108.8 mg, 0.3 mmol), NaH (36.0 mg, 60 wt% dispersion in mineral oil, 0.9 mmol, 3.0 equiv), $Tp^{Br3}Ag(thf)$ (33.0 mg, 10 mol%) and anhydrous $CHCl_3$ (5 mL) inside a glove box. Anhydrous THF and THF- d_8 were added. The tube was sealed and rinsed in an ultrasonic bath for 5 min. The resulting mixture was stirred (700 rpm) at 60 ℃ for 24 h. When the reaction was completed, the crude reaction mixture was allowed to reach room temperature and filtered through a short pad of silica gel with EtOAc as eluent. The filtrate was concentrated in vacuo to leave a crude mixture, which was analyzed by ^{1}H NMR for k_H/k_D.

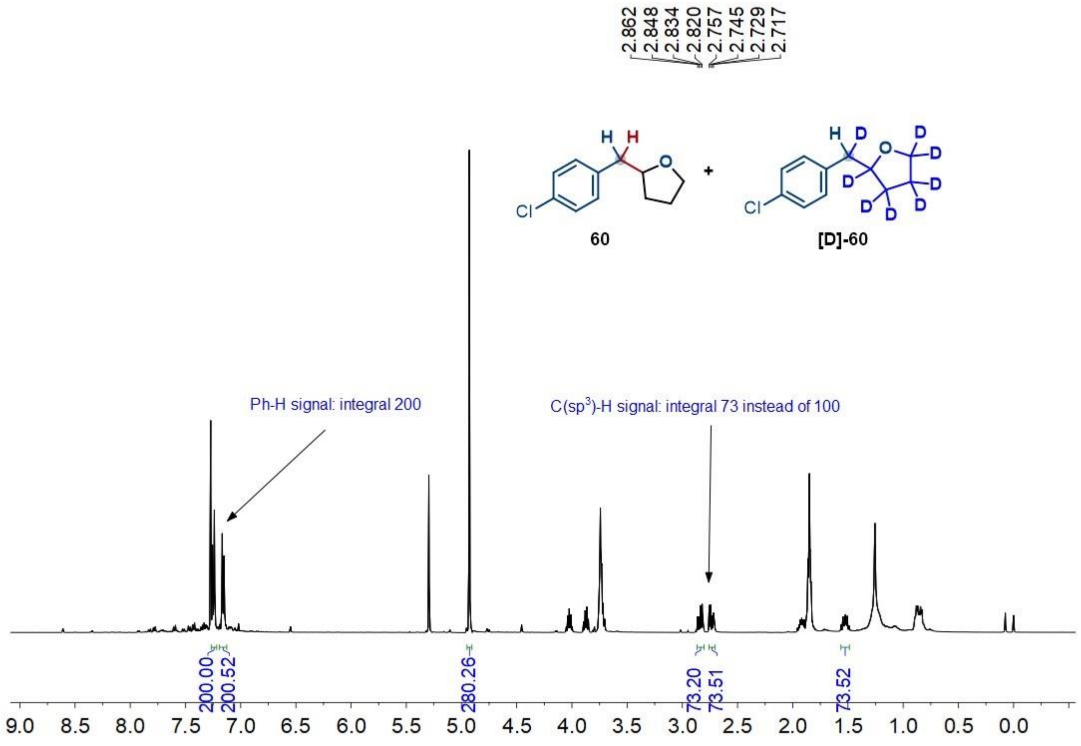

Supplementary Fig. 5 ^{1}H NMR of crude mixture of compound **60** and **[D]-60**

7.2 Ether Exchange Experiment

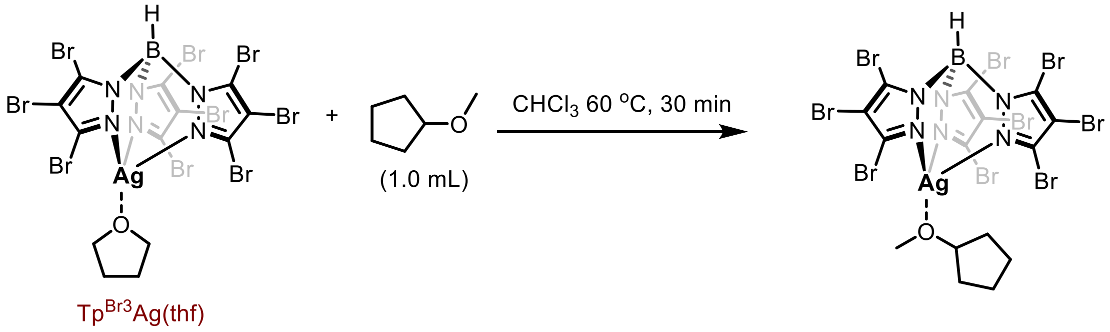

Supplementary Scheme 3 Ether exchange experiment.

To an oven-dried sealed tube was charged with $Tp^{Br3}Ag(thf)$ (32.7 mg, 0.03 mmol) and anhydrous $CHCl_3$ (5.0 mL) in an argon-filled glovebox. The tube was sealed and stirred (700 rpm) at 60 ^{o}C for 0.5 h. The reaction mixture was concentrated in vacuo to leave a crude mixture, which was analyzed by ^{1}H NMR.

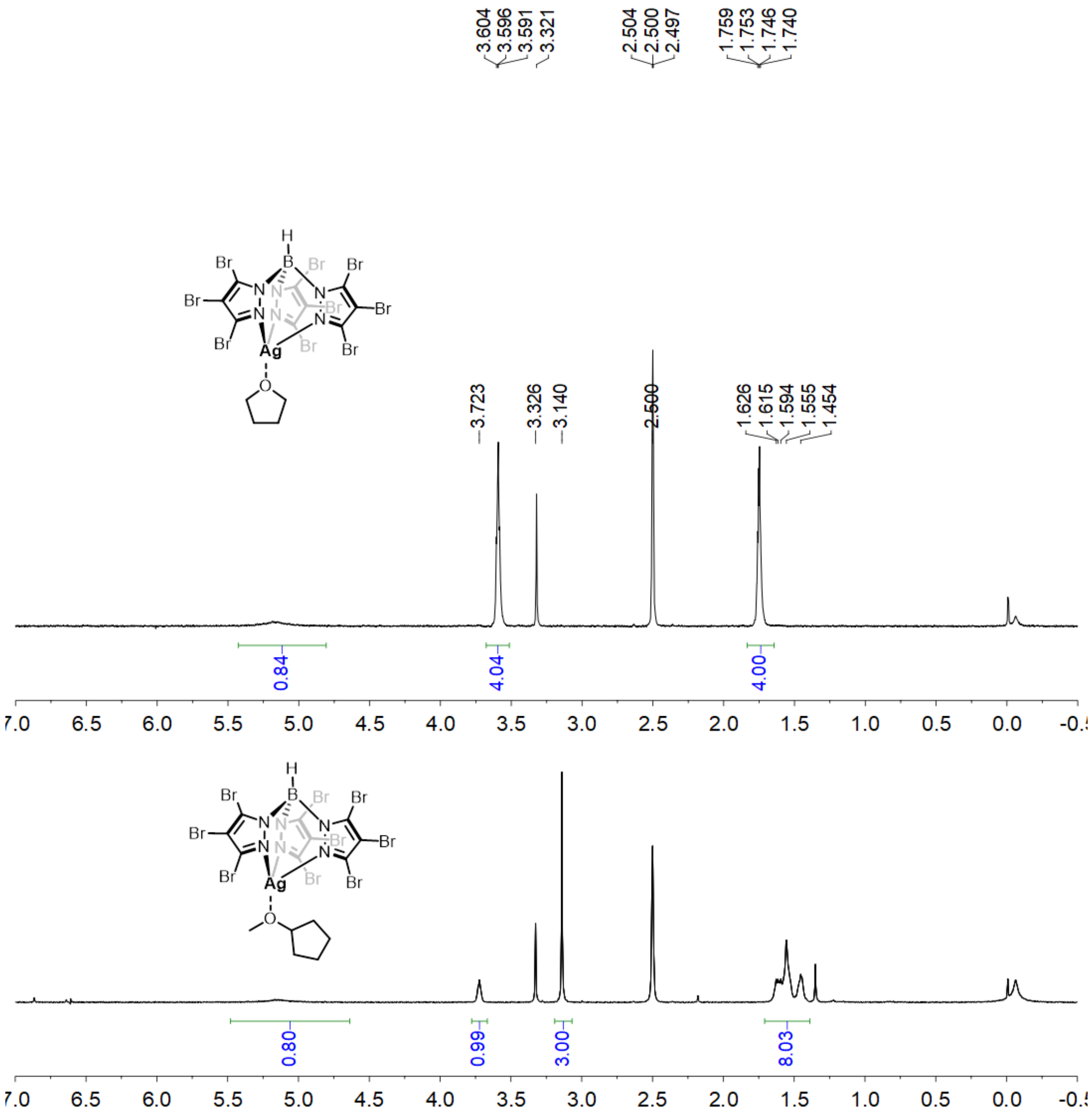

Supplementary Fig. 6 Analytical data of ^{1}H NMR of $Tp^{Br3}AgL$.

7.3 Computational Details

All quantum mechanical calculations were performed using the Gaussian 09 suite of program[16] using the M06[17] functional. The Ag atom was represented with the Stuttgart-Dresden relativistic effective core potential associated with their adapted basis set.[18] All the other atoms were described with 6-31G(d) basis set.[19,20] Geometry optimizations were computed without any symmetry constraints using the SMD[21] solvent model for chloroform. Frequency calculations at the same level were used to confirm the presence of local minima (no imaginary frequencies) and transition states (one imaginary frequency). Intrinsic reaction coordinate (IRC)[22,23] were traced from the various transition structures to obtain the connected intermediates. The single-point energies were subsequently obtained at the same functional with the 6-31+G(d,p) basis set. The key 3D structures of transition states were draw by CYLview visualization software[24].

Possible structures for C–H bond insertion reaction of methyl isoamyl ether

We have carefully searched for the possible structures for each type of C–H bond insertion reaction of methyl isoamyl ether, and ascertained that the geometry conformers given in Figure 8c were the most favorable. The results are as follows:

1) For 2° α-C–H insertion of methyl isoamyl ether, as shown in Supplementary Fig. 8, we have searched for four possible conformers **a**, **b**, **c**, and **d** with different alkoxy orientations. Based on their energies, we choosed the most stable conformer **a** as our model for the calculation of 2° α-C–H insertion of ethyl isoamyl ether. In addition, the relatively less stable conformers **c** and **d** were excluded from searching for possible conformers of 1° α-C–H and alkane 3° C–H bonds insertion.

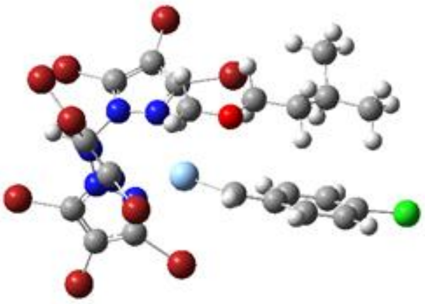

G = -25026.169705 a.u.

(a) favored

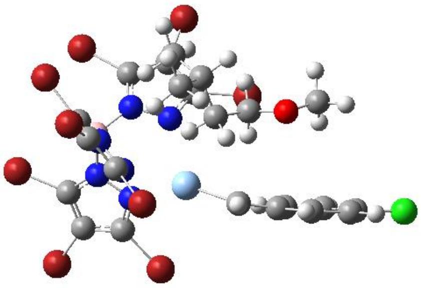

G = -25026.167053 a.u.

(b)

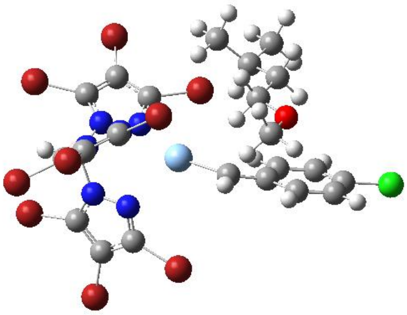

G = -25026.166863 a.u.

(c)

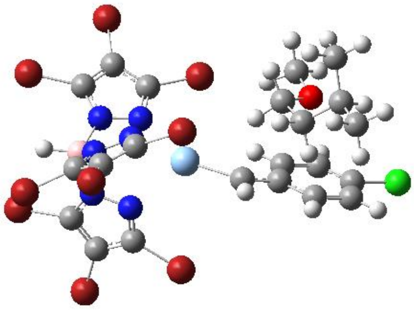

G = -25026.164392 a.u.

(d)

Supplementary Fig. 7 Four possible conformers for 2° α-C–H insertion of methyl isoamyl ether.

2) For 1° α-C–H insertion of methyl isoamyl ether, as shown in Supplementary Fig. 9, we have searched for two relatively stable conformers **a** and **b** with different orientations. Based on their energies, we choosed the more stable conformer **a** as the model for the calculation of 1° α-C–H insertion of ethyl isoamyl ether.

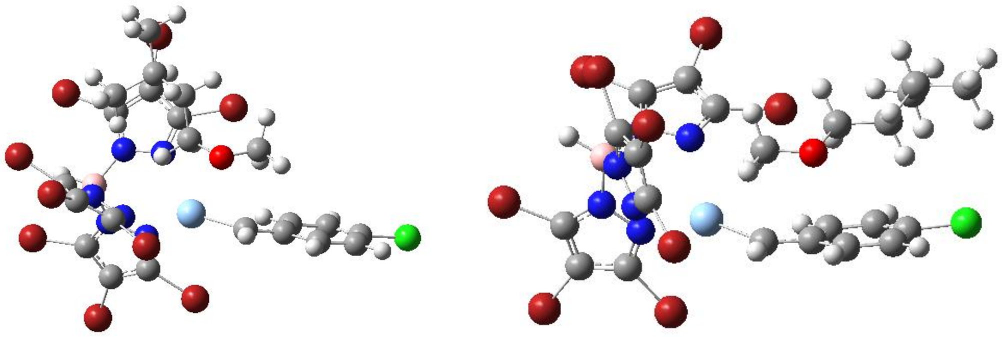

G = -25026.174765 a.u.   G = -25026.166409 a.u.

(a)   (b)

Supplementary Fig. 8 Two relatively stable conformers for 1° α-C–H insertion of methyl isoamyl ether.

3) For alkane 3° C–H insertion of methyl isoamyl ether, as shown in Supplementary Fig. 10, we have searched for two relatively stable conformers **a** and **b** with different orientations. Based on their energies, we choosed the more stable conformer **a** as the model for the calculation of alkane 3° C–H insertion of ethyl isoamyl ether.

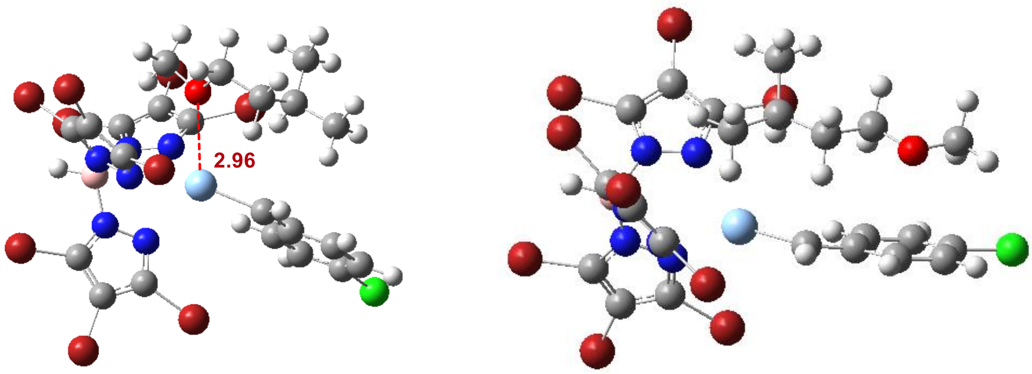

G = -25026.173528 a.u.   G = -25026.170630 a.u.

(a) favored   (b)

Supplementary Fig. 9 Two relatively stable conformers for alkane 3° C–H insertion of methyl isoamyl ether.

DFT calculations at different levels of theory using single-point energy calculations

We have also optimized the calculated geometries using single-point energy calculations at multiple different higher levels of theory, such as M062X, ωB97XD and B3LYP-D3(BJ), where the site selectivity trend and the relative energies for transition states and intermediates are unchanged.

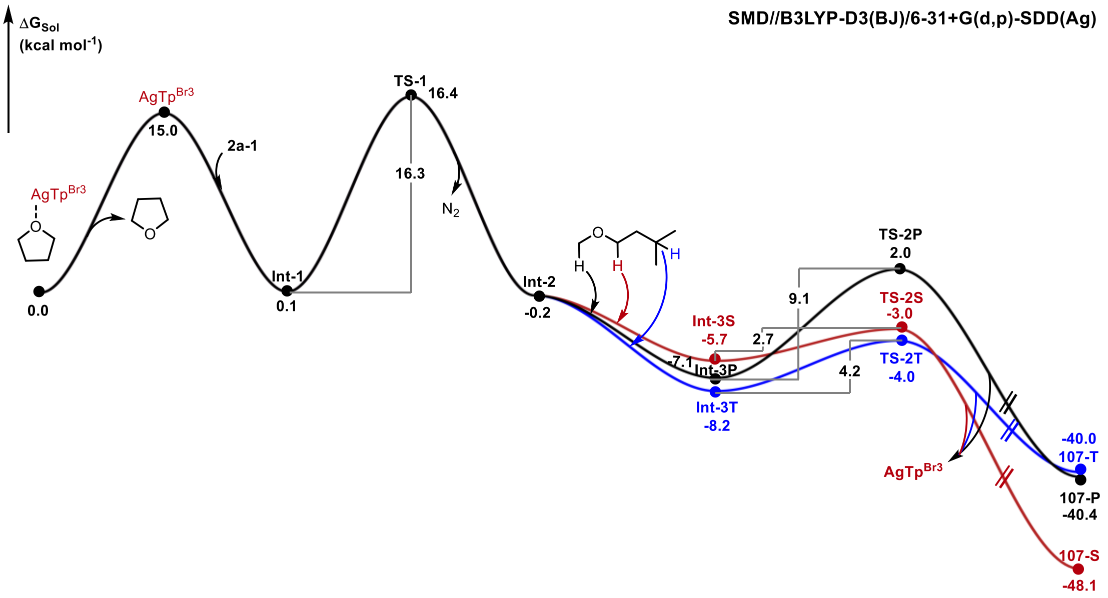

Supplementary Fig. 10 Computed energy profiles for the silver-catalyzed carbene insertion into C–H bonds of methyl isoamyl ether at SMD//B3LYP-D3(BJ)/6-31+G(d,p)-SDD(Ag) level. Energies are given in kcal mol-1 and distances in angstroms.

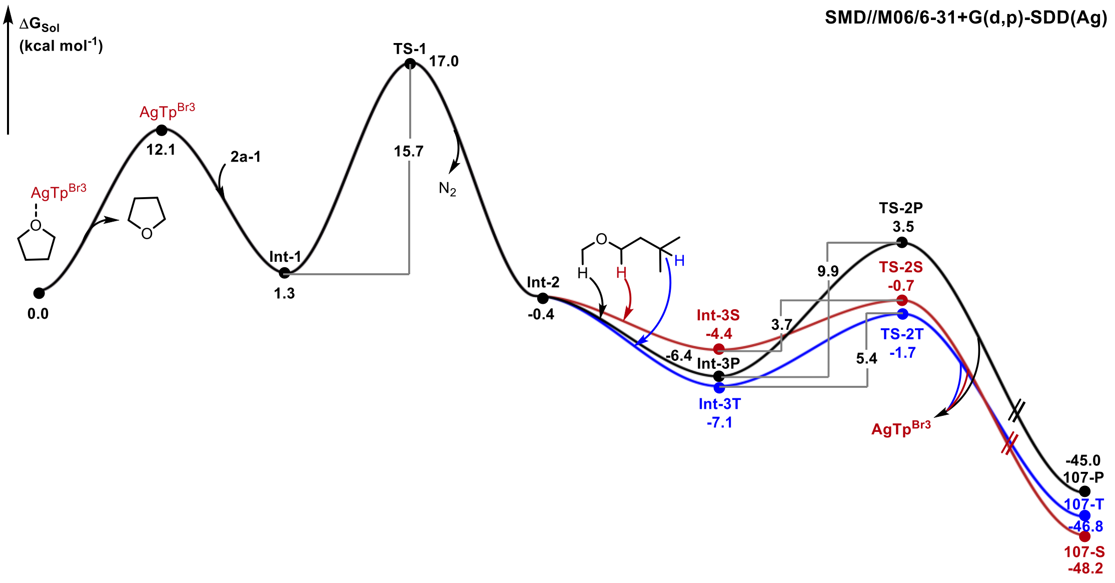

Supplementary Fig. 11 Computed energy profiles for the silver-catalyzed carbene insertion into C–H bonds of methyl isoamyl ether at SMD//M06/6-31+G(d,p)-SDD(Ag) level. Energies are given in kcal mol-1 and distances in angstroms.

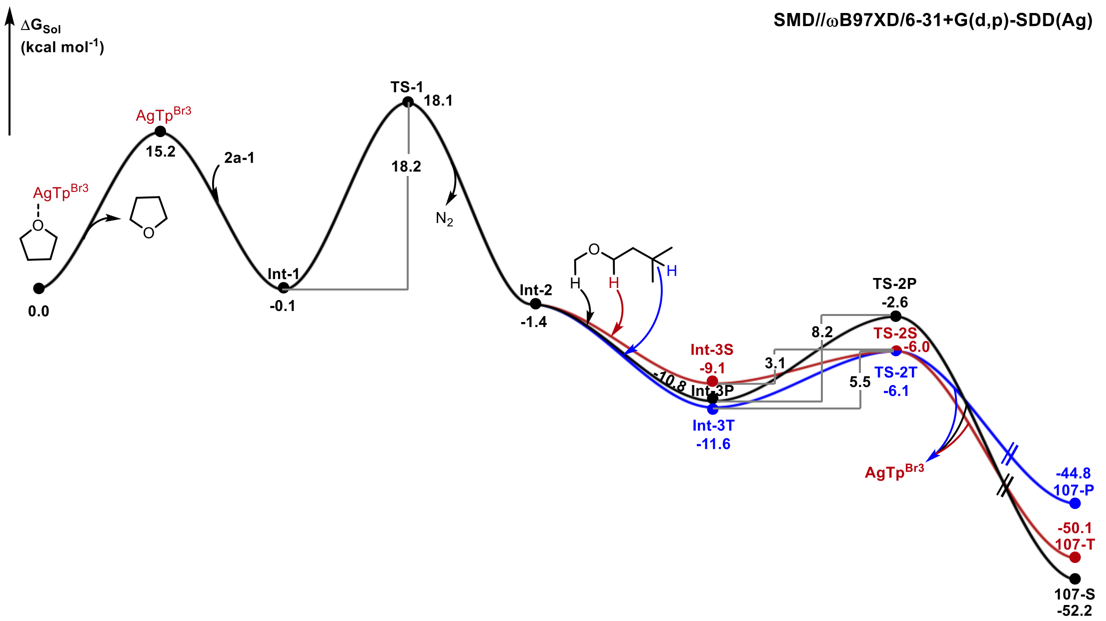

Supplementary Fig. 12 Computed energy profiles for the silver-catalyzed carbene insertion into C–H bonds of methyl isoamyl ether at SMD//ωB97XD/6-31+G(d,p)-SDD(Ag) level. Energies are given in kcal mol-1 and distances in angstroms.

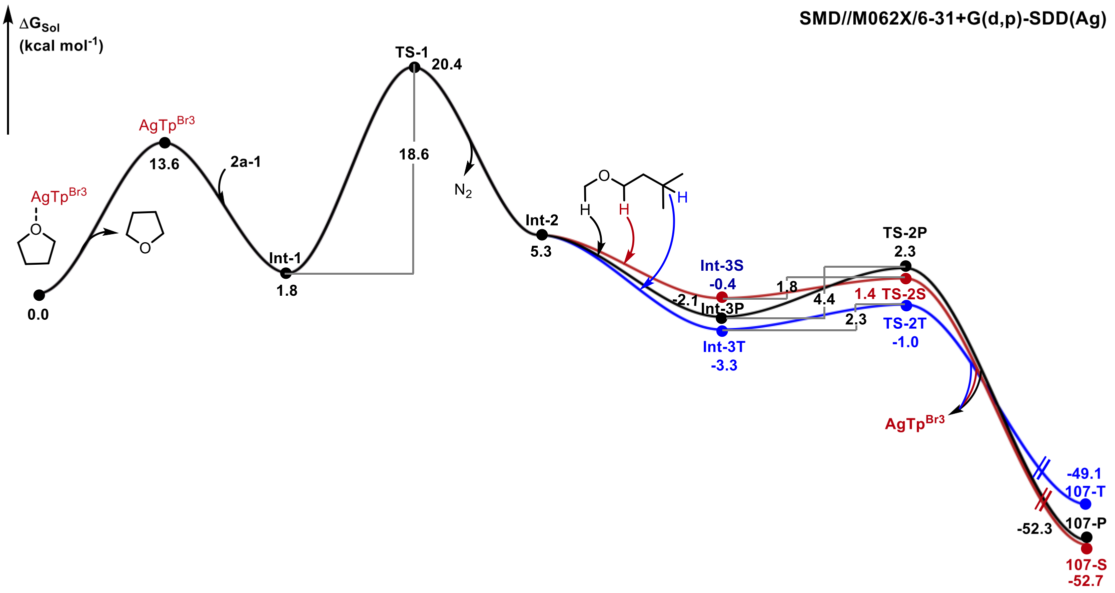

Supplementary Fig. 13 Computed energy profiles for the silver-catalyzed carbene insertion into C–H bonds of methyl isoamyl ether at at SMD//M062X/6-31+G(d,p)-SDD(Ag) level. Energies are given in kcal mol-1 and distances in angstroms.

Cartesian coordinates of the SMD//M06/[6-31G(d)/SDD(Ag)] computed structures

AgTp^{Br3}-THF

Zero-point correction= 0.227738 (Hartree/Particle)
Thermal correction to Energy= 0.262654
Thermal correction to Enthalpy= 0.263598
Thermal correction to Gibbs Free Energy= 0.147436
Sum of electronic and zero-point Energies= -24216.850591
Sum of electronic and thermal Energies= -24216.815675
Sum of electronic and thermal Enthalpies= -24216.814731
Sum of electronic and thermal Free Energies= -24216.930893

Ag	-1.59688600	0.56942900	-1.15095000
N	-1.22419300	0.14263900	1.20592900
N	0.00213400	-0.26370900	1.61695700
N	-0.12980500	-1.32843300	-1.24658600
N	0.97150800	-1.36612400	-0.45549600
N	0.48769500	1.66341000	-0.81128900
N	1.41761500	1.10068200	0.00181500
B	1.21098900	-0.29745300	0.64431600
C	-2.03358000	0.01647600	2.24151200
C	-1.36056100	-0.47984100	3.36406700
C	-0.06239600	-0.64615200	2.91364300
C	-0.04944300	-2.36493400	-2.06284400
C	1.10821500	-3.11581200	-1.82738200
C	1.72753200	-2.43791500	-0.79060100
C	0.95235000	2.85061700	-1.15934500
C	2.20415900	3.09986700	-0.58647700
C	2.45724900	1.95428800	0.14820900
H	2.18633500	-0.58719900	1.25706300
Br	-1.38177800	-2.67558300	-3.32865200
Br	1.67482700	-4.65660800	-2.69356000
Br	3.32949500	-2.88308600	0.04027700
Br	-2.04467900	-0.82923500	5.05416500
Br	-3.83699100	0.46827200	2.09291800
Br	1.38623500	-1.29488600	3.88006000
Br	3.95905800	1.59951300	1.18412500
Br	3.27053000	4.60930300	-0.75817700
Br	-0.04306000	3.96200700	-2.27567900
C	-4.61814900	-0.18386400	-1.98768900
O	-3.63942800	0.87546600	-2.06965200
C	-4.28333600	2.15941100	-1.94313800
C	-5.75274800	1.88225400	-2.17892900
C	-5.90775700	0.49315300	-1.56668200
H	-4.70347500	-0.64621300	-2.98174200
H	-4.25608700	-0.94064000	-1.27895900
H	-4.10021700	2.55604600	-0.93138400

H	-3.82568000	2.83588800	-2.67437400
H	-6.39766100	2.63733900	-1.71677800
H	-5.96913100	1.85285000	-3.25580700
H	-5.96853900	0.56287000	-0.47088800
H	-6.79254900	-0.04455000	-1.92388500

THF

Zero-point correction= 0.116669 (Hartree/Particle)
Thermal correction to Energy= 0.121604
Thermal correction to Enthalpy= 0.122548
Thermal correction to Gibbs Free Energy= 0.088158
Sum of electronic and zero-point Energies= -232.175982
Sum of electronic and thermal Energies= -232.171047
Sum of electronic and thermal Enthalpies= -232.170103
Sum of electronic and thermal Free Energies= -232.204493

C	-1.15693600	-0.41992200	-0.13331600
O	-0.00285800	-1.24211000	-0.00511400
C	1.15300500	-0.42589500	0.13978200
C	0.72715100	0.98201600	-0.23995100
C	-0.72010700	0.98790200	0.23696000
H	-1.52268400	-0.45928600	-1.17424500
H	-1.95098700	-0.81616200	0.51476200
H	1.50353500	-0.46286900	1.18635300
H	1.95400500	-0.82860500	-0.49529500
H	1.35063700	1.75537600	0.22273900
H	0.76854400	1.11379900	-1.33049300
H	-0.75996100	1.12776500	1.32652600
H	-1.33891200	1.76226000	-0.23028600

$AgTp^{Br3}$

Zero-point correction= 0.108903 (Hartree/Particle)
Thermal correction to Energy= 0.136650
Thermal correction to Enthalpy= 0.137594
Thermal correction to Gibbs Free Energy= 0.040222
Sum of electronic and zero-point Energies= -23984.651874
Sum of electronic and thermal Energies= -23984.624126
Sum of electronic and thermal Enthalpies= -23984.623182
Sum of electronic and thermal Free Energies= -23984.720554

Ag	0.01443800	-0.03669900	-2.47352700
N	-0.59534000	1.67739200	-0.94688000

N	-0.44279300	1.39332500	0.37281700
N	-1.16563700	-1.39807600	-0.91279200
N	-0.97653500	-1.10339500	0.39953800
N	1.78939100	-0.36778100	-0.92757800
N	1.45808800	-0.31556300	0.38849600
B	0.01459200	-0.00561200	0.87309100
C	-0.99369300	2.93552900	-1.02487600
C	-1.11370200	3.51340300	0.24340200
C	-0.75196400	2.49043800	1.10264300
C	-2.08171100	-2.34918100	-0.96399900
C	-2.52055000	-2.70666900	0.31524300
C	-1.78717000	-1.88386300	1.15187200
C	3.07833900	-0.65408300	-0.98835700
C	3.63172500	-0.79359800	0.28863200
C	2.55869500	-0.57015400	1.13372400
H	0.01626000	0.00565900	2.05982300
Br	2.58109300	-0.60855500	2.99180800
Br	5.38799700	-1.18284600	0.74350600
Br	3.94008500	-0.83085100	-2.63061900
Br	-0.68810700	2.56810500	2.95804100
Br	-1.63805600	5.24053400	0.67373700
Br	-1.32408600	3.73389500	-2.67627700
Br	-1.87086500	-1.82022200	3.00712100
Br	-3.78613600	-3.98181200	0.78071500
Br	-2.64071900	-3.04239200	-2.60044500

2a-1

Zero-point correction= 0.104979 (Hartree/Particle)
Thermal correction to Energy= 0.113499
Thermal correction to Enthalpy= 0.114443
Thermal correction to Gibbs Free Energy= 0.070668
Sum of electronic and zero-point Energies= -838.998174
Sum of electronic and thermal Energies= -838.989654
Sum of electronic and thermal Enthalpies= -838.988710
Sum of electronic and thermal Free Energies= -839.032485

N	-3.37842800	-0.16415100	0.00004700
C	-2.48158100	0.77116200	-0.00014400
N	-4.15388100	-1.00657000	0.00026400
H	-2.86153000	1.78891200	-0.00028600
C	-1.06392100	0.45711100	-0.00014400
C	-0.14115500	1.51259200	0.00001700
C	-0.58066000	-0.85908800	-0.00017300

C	1.22385800	1.26552800	0.00008000
H	-0.50180900	2.54057200	0.00009800
C	0.78226600	-1.11507700	-0.00011900
H	-1.27705200	-1.69732600	-0.00023700
C	1.67372600	-0.04865500	-0.00001000
H	1.93454600	2.08894400	0.00021500
H	1.15137700	-2.13821300	-0.00016900
Cl	3.40031900	-0.36995800	0.00006800

Int-1

Zero-point correction=	0.214949 (Hartree/Particle)
Thermal correction to Energy=	0.253803
Thermal correction to Enthalpy=	0.254747
Thermal correction to Gibbs Free Energy=	0.130100
Sum of electronic and zero-point Energies=	-24823.662032
Sum of electronic and thermal Energies=	-24823.623178
Sum of electronic and thermal Enthalpies=	-24823.622234
Sum of electronic and thermal Free Energies=	-24823.746881

Ag	-1.10594700	-1.21054800	-0.35456600
N	-0.93803300	1.17943700	-0.34080700
N	0.28512200	1.76250100	-0.24449900
N	0.48455500	-0.90709600	1.43266200
N	1.35932600	0.12908900	1.38157900
N	0.93365600	-0.83029700	-1.64313200
N	1.88385600	-0.03669100	-1.08795400
B	1.55936900	0.94126600	0.07129700
C	-1.80431800	2.14807100	-0.58657700
C	-1.17167300	3.39514000	-0.65501500
C	0.16020700	3.09666700	-0.43083500
C	0.54459300	-1.39153900	2.66185300
C	1.46323600	-0.68816300	3.44782500
C	1.95604500	0.27542400	2.58522100
C	1.54958300	-1.58212500	-2.53819100
C	2.91785700	-1.29676600	-2.60337900
C	3.08250200	-0.30286000	-1.65541700
H	2.47640600	1.68391400	0.21420800
Br	-0.50900300	-2.85008700	3.15058400
Br	1.93144600	-0.97660700	5.22070600
Br	3.22439200	1.57805700	2.96772900
Br	-1.94117200	5.05485400	-0.97047900
Br	-3.61769600	1.77525800	-0.79434800
Br	1.58049700	4.29652300	-0.37355900

Br	4.66867000	0.53961600	-1.17950700
Br	4.20248400	-2.06486000	-3.70145700
Br	0.61257300	-2.84826000	-3.53789000
N	-2.39368700	-3.82549900	0.05068300
C	-2.70539400	-2.80355300	-0.76057700
N	-2.11180600	-4.63924400	0.78294900
H	-2.45569600	-3.05073900	-1.79685100
C	-4.00357200	-2.13478600	-0.49464700
C	-4.73928400	-1.65498800	-1.58243800
C	-4.49238400	-1.94522200	0.80231000
C	-5.94464900	-0.99464500	-1.38246100
H	-4.36018000	-1.79140100	-2.59420500
C	-5.70308400	-1.29886600	1.01168500
H	-3.92036400	-2.29282600	1.66257700
C	-6.41641300	-0.82719600	-0.08554000
H	-6.51600000	-0.61790600	-2.22789800
H	-6.08362000	-1.15203200	2.02025000
Cl	-7.93912000	-0.00234100	0.17320400

TS-1

Zero-point correction= 0.212736 (Hartree/Particle)
Thermal correction to Energy= 0.251708
Thermal correction to Enthalpy= 0.252652
Thermal correction to Gibbs Free Energy= 0.128296
Sum of electronic and zero-point Energies= -24823.639743
Sum of electronic and thermal Energies= -24823.600771
Sum of electronic and thermal Enthalpies= -24823.599827
Sum of electronic and thermal Free Energies= -24823.724183

Ag	-1.17556300	0.49354700	1.02310000
N	-0.82588700	-1.09169500	-0.83134100
N	0.45561400	-1.35913900	-1.18947700
N	0.29007000	1.71313600	-0.38272500
N	1.21154800	1.08152900	-1.15227000
N	1.01744800	-0.49292300	1.57753800
N	1.98594700	-0.53561900	0.62892100
B	1.61818800	-0.38851500	-0.87181600
C	-1.53081000	-2.16979100	-1.13483800
C	-0.72928100	-3.17145000	-1.69568100
C	0.53408000	-2.60758100	-1.69993000
C	0.23788500	2.96546700	-0.80172500
C	1.12919800	3.19205300	-1.85598200
C	1.72864300	1.95846300	-2.04252900

C	1.63314100	-0.57773300	2.74422100
C	3.02096100	-0.68207300	2.59069200
C	3.19587500	-0.64654800	1.21812100
H	2.55933800	-0.66561800	-1.54453900
Br	-0.91096100	4.19108500	0.00736600
Br	1.44707700	4.77106000	-2.77857400
Br	3.03235000	1.53127400	-3.29544300
Br	-1.22780800	-4.85621300	-2.29629800
Br	-3.36429200	-2.24687900	-0.79735800
Br	2.12192100	-3.39477200	-2.26144300
Br	4.80030600	-0.72631300	0.28229100
Br	4.32092600	-0.83035700	3.90756500
Br	0.66784500	-0.53704500	4.34036400
N	-2.84841400	2.39922500	2.80664700
C	-2.96237200	0.68134900	2.19528700
N	-2.63860000	3.48911800	2.78477600
H	-2.94809900	0.24039900	3.20001500
C	-4.28536300	0.62885400	1.57600500
C	-5.34165500	-0.04469500	2.20758100
C	-4.49174000	1.16953800	0.29595000
C	-6.56276900	-0.20846500	1.56946900
H	-5.19553100	-0.45912900	3.20473200
C	-5.71690200	1.05227900	-0.33743300
H	-3.66584500	1.67764400	-0.20464700
C	-6.73529200	0.35197800	0.30735300
H	-7.37780800	-0.74677400	2.04766500
H	-5.87991300	1.47323500	-1.32686100
Cl	-8.27441500	0.16978500	-0.49489600

Int-2

Zero-point correction=	0.212349 (Hartree/Particle)
Thermal correction to Energy=	0.252313
Thermal correction to Enthalpy=	0.253257
Thermal correction to Gibbs Free Energy=	0.126437
Sum of electronic and zero-point Energies=	-24823.664983
Sum of electronic and thermal Energies=	-24823.625020
Sum of electronic and thermal Enthalpies=	-24823.624076
Sum of electronic and thermal Free Energies=	-24823.750895

Ag	-1.37011600	-0.29019400	-0.75323000
N	-0.08846700	1.67641200	-0.78686200
N	1.24348100	1.68170200	-0.53126600
N	-0.05510800	-0.60043600	1.25736000

N	1.12583400	0.05243500	1.38556500
N	0.97398600	-0.92355400	-1.79057900
N	1.93320500	-0.75123900	-0.84596200
B	1.93107400	0.45565300	0.12321200
C	-0.39262600	2.87479300	-1.25990900
C	0.73938900	3.69800800	-1.31748500
C	1.75473500	2.88949400	-0.84288600
C	-0.51639900	-0.78324400	2.48377100
C	0.35019300	-0.25544500	3.44688600
C	1.38197400	0.27666300	2.69241900
C	1.20587500	-2.10327300	-2.33832100
C	2.31668300	-2.73916600	-1.76735700
C	2.73892100	-1.83426800	-0.80783400
H	3.04780000	0.74335900	0.42995000
Br	-2.14314300	-1.66007100	2.77564200
Br	0.17066100	-0.26610500	5.29548300
Br	2.88377200	1.18280300	3.30131900
Br	0.84658400	5.46796700	-1.87243200
Br	-2.13499600	3.29904800	-1.75915700
Br	3.54526800	3.35357400	-0.62973700
Br	4.13852000	-2.03173500	0.40448900
Br	3.01210000	-4.41400200	-2.15636300
Br	0.09590000	-2.77117000	-3.68411400
C	-3.15357800	-1.17012300	-1.34711800
H	-3.14469700	-1.99975400	-2.07341700
C	-4.46248300	-0.90918100	-0.88638400
C	-5.57926800	-1.66459300	-1.34531400
C	-4.68784000	0.12185700	0.06556300
C	-6.85786700	-1.40254900	-0.88646800
H	-5.42209900	-2.46156400	-2.07302900
C	-5.95990900	0.39534800	0.52799600
H	-3.82658300	0.68590800	0.42838100
C	-7.02611100	-0.37047500	0.04026500
H	-7.71423600	-1.97487700	-1.23266100
H	-6.13940400	1.18508200	1.25548800
Cl	-8.62700600	-0.02522300	0.61523400

Methyl isoamyl ether

Zero-point correction= 0.193604 (Hartree/Particle)
Thermal correction to Energy= 0.202670
Thermal correction to Enthalpy= 0.203614
Thermal correction to Gibbs Free Energy= 0.160326
Sum of electronic and zero-point Energies= -311.861432

Sum of electronic and thermal Energies= -311.852365
Sum of electronic and thermal Enthalpies= -311.851421
Sum of electronic and thermal Free Energies= -311.894709

C	3.28562500	0.15255900	0.06325100
H	3.34668300	1.09780700	-0.50549100
H	3.38507700	0.39540100	1.13647600
H	4.12617900	-0.48681100	-0.22921000
O	2.10022600	-0.54065100	-0.20485900
C	0.96504300	0.21512100	0.14274900
H	0.99083200	0.46669700	1.22331800
H	0.97587700	1.17622800	-0.40627800
C	-0.27264400	-0.59073300	-0.18180100
H	-0.34772900	-0.71222900	-1.27585600
H	-0.13527900	-1.60046400	0.23601000
C	-1.57014700	0.01049400	0.35392200
H	-1.49008000	0.06205900	1.45453300
C	-1.80623500	1.42142500	-0.17084900
H	-2.77954700	1.80875200	0.16096900
H	-1.03932700	2.12937000	0.17093400
H	-1.80392900	1.43150500	-1.27221200
C	-2.74625500	-0.88845400	0.00007700
H	-2.60585900	-1.91084600	0.37744100
H	-3.68733700	-0.50327200	0.41623500
H	-2.86968700	-0.95146300	-1.09210300

Int-3S

Zero-point correction= 0.401374 (Hartree/Particle)
Thermal correction to Energy= 0.448172
Thermal correction to Enthalpy= 0.449116
Thermal correction to Gibbs Free Energy= 0.309261
Sum of electronic and zero-point Energies= -25026.077593
Sum of electronic and thermal Energies= -25026.030794
Sum of electronic and thermal Enthalpies= -25026.029850
Sum of electronic and thermal Free Energies= -25026.169705

Ag	0.89632800	0.43591000	-0.68006100
N	-0.15645500	-0.34885400	1.34420600
N	-1.50877000	-0.31903000	1.45372500
N	-1.07097600	1.72564000	-0.63619800
N	-2.28662500	1.16374300	-0.41586800
N	-1.04384700	-0.98176600	-1.84811800
N	-1.88890700	-1.30762800	-0.83922300

B	-2.39957600	-0.26017200	0.18040800
C	0.31881100	-0.26424300	2.57829900
C	-0.70964300	-0.18006000	3.52325400
C	-1.85882400	-0.21088800	2.75201900
C	-1.29240900	2.94216400	-1.10354800
C	-2.66234600	3.21417200	-1.19339600
C	-3.25481200	2.04821900	-0.74042600
C	-0.79441900	-2.10812900	-2.49428400
C	-1.46121000	-3.20011900	-1.92275700
C	-2.14343700	-2.63356400	-0.86059400
H	-3.53128300	-0.49421700	0.47529900
C	2.77042500	0.28889400	-1.55806800
H	2.92602600	-0.47362800	-2.33838200
C	3.95429100	1.01149800	-1.30045900
C	3.98418800	2.00510200	-0.28640400
C	5.15320000	0.73590300	-2.01140500
C	5.15160100	2.66885500	0.02734200
H	3.06060700	2.22616500	0.24992700
C	6.32550200	1.39981800	-1.71576700
H	5.13585400	-0.01755100	-2.79866600
C	6.31019800	2.34638800	-0.68693800
H	5.18502500	3.42130000	0.81141200
H	7.24862600	1.19053500	-2.25134500
Cl	7.79231600	3.14083000	-0.27247700
Br	0.12261000	4.06913600	-1.55241700
Br	-3.49005400	4.77288400	-1.76685400
Br	-5.07282700	1.70895800	-0.56559200
Br	-0.56762700	-0.05453200	5.36913100
Br	2.15404100	-0.23263200	2.91380000
Br	-3.61742900	-0.10426600	3.34299700
Br	-3.23478600	-3.48152200	0.38369700
Br	-1.44455600	-4.98090800	-2.44866200
Br	0.34557300	-2.11525700	-3.97341700
O	3.28738400	-3.02373500	-0.88937200
C	3.76877600	-2.39761100	0.27413500
H	3.23283300	-1.43701700	0.43033600
H	3.54337600	-3.02727000	1.15718100
C	1.92160600	-3.32022700	-0.78581800
H	1.61170800	-3.79204400	-1.72503000
H	1.71655200	-4.01714700	0.04734300
H	1.31659900	-2.40518900	-0.62263800
C	5.25367400	-2.15020500	0.13406900
H	5.42646700	-1.69437900	-0.85610600
H	5.79270500	-3.11289500	0.13409900

C	5.82700300	-1.23441000	1.21407700
H	5.27227400	-0.27726800	1.16801700
C	7.29684400	-0.94713200	0.93985500
H	7.45221100	-0.54325900	-0.07177300
H	7.70598700	-0.22085100	1.65662000
H	7.89293600	-1.86887300	1.02386100
C	5.65919600	-1.81698500	2.61214800
H	4.60531400	-1.96702300	2.88568900
H	6.15904500	-2.79552000	2.68550500
H	6.10592700	-1.15950800	3.37100200

Int-3P

Zero-point correction= 0.399583 (Hartree/Particle)
Thermal correction to Energy= 0.446800
Thermal correction to Enthalpy= 0.447745
Thermal correction to Gibbs Free Energy= 0.305420
Sum of electronic and zero-point Energies= -25026.080602
Sum of electronic and thermal Energies= -25026.033385
Sum of electronic and thermal Enthalpies= -25026.032440
Sum of electronic and thermal Free Energies= -25026.174765

Ag	-1.51006000	0.11325300	0.62511100
N	-0.15943800	-0.63697500	-1.20771300
N	1.07694900	-0.12650900	-1.45049200
N	-0.51076500	2.13317100	-0.17065600
N	0.81617400	2.20859000	-0.46054200
N	0.78984200	0.21523300	1.72539600
N	1.86469400	0.35182100	0.90709200
B	1.70909700	0.94369200	-0.51915600
C	-0.37824000	-1.55995000	-2.12877900
C	0.71506200	-1.69782100	-2.98941600
C	1.62063100	-0.76084600	-2.51790600
C	-0.96021100	3.37793000	-0.13986800
C	0.05956700	4.30754600	-0.39920000
C	1.17529100	3.50808100	-0.59541500
C	1.23405100	-0.38150500	2.82294200
C	2.60285200	-0.66528500	2.74061800
C	2.95870200	-0.17881100	1.49445900
H	2.77954500	1.23452700	-0.94610400
C	-3.25158200	-0.56117700	1.54090300
H	-3.19863000	-1.04570700	2.52924600
C	-4.58120300	-0.53819100	1.05867600
C	-4.87020600	0.02886800	-0.21571800

C	-5.65117800	-1.09745800	1.80089300
C	-6.15023000	0.03258500	-0.71781100
H	-4.04394900	0.45464000	-0.78836200
C	-6.93727200	-1.09756300	1.31345800
H	-5.43439300	-1.54331200	2.77464500
C	-7.16951300	-0.53105300	0.05600800
H	-6.37518700	0.46520600	-1.69180700
H	-7.76434900	-1.52925800	1.87818400
Cl	-8.78599100	-0.53686500	-0.57178100
Br	-2.75023100	3.74179200	0.24148800
Br	-0.05025800	6.15839500	-0.48080500
Br	2.90672700	4.09720500	-0.97215100
Br	0.93863900	-2.89369100	-4.38954600
Br	-1.97289800	-2.52424700	-2.18023200
Br	3.30886100	-0.41340300	-3.20667100
Br	4.63972400	-0.24480200	0.69960700
Br	3.67512000	-1.53681300	3.97874800
Br	0.09263400	-0.75065500	4.24746700
C	1.21988800	-4.31062700	-0.37235100
H	0.64207900	-3.88048800	-1.20787400
C	2.27958900	-3.29004900	0.03586700
H	1.85063700	-2.29777500	0.22111900
H	2.80935600	-3.60604400	0.95283100
H	3.03968200	-3.18014200	-0.75475700
C	1.87346800	-5.59189100	-0.86807600
H	1.12528300	-6.34349600	-1.15900400
H	2.51514200	-5.39954600	-1.74111900
H	2.50892800	-6.03834400	-0.08359000
C	0.24032900	-4.61657800	0.76036300
H	0.75139900	-5.18981800	1.54954400
H	-0.55587600	-5.27181300	0.36078900
C	-0.39198300	-3.37840600	1.37133500
H	-0.71149100	-2.67325600	0.57406100
H	0.34317400	-2.84578100	1.99669800
O	-1.48883600	-3.66090400	2.22075000
C	-2.67987200	-3.88409800	1.52530500
H	-2.95807900	-3.00112200	0.91238200
H	-2.63478500	-4.75599800	0.84519100
H	-3.46959000	-4.06178600	2.26175500

Int-3T

Zero-point correction= 0.400840 (Hartree/Particle)
Thermal correction to Energy= 0.446530

Thermal correction to Enthalpy= 0.447474
Thermal correction to Gibbs Free Energy= 0.309577
Sum of electronic and zero-point Energies= -25026.082265
Sum of electronic and thermal Energies= -25026.036575
Sum of electronic and thermal Enthalpies= -25026.035631
Sum of electronic and thermal Free Energies= -25026.173528

Ag	0.90266900	-0.05000700	-1.12757500
N	0.43257100	-1.04865400	1.01101000
N	-0.56898200	-0.61129400	1.81886000
N	0.44215500	1.94302200	0.88908900
N	-0.88553200	1.82583300	1.13998600
N	-1.43936800	0.31425900	-1.20684900
N	-2.12823800	0.02852600	-0.07100800
B	-1.57580400	0.45401300	1.32632000
C	1.07940300	-1.98458900	1.68862000
C	0.51957600	-2.18521500	2.95297200
C	-0.53513800	-1.28575000	2.99274700
C	0.63268200	3.20705500	0.55185000
C	-0.55886300	3.94486100	0.55870100
C	-1.50384100	3.00538900	0.93107900
C	-2.16950700	-0.14834300	-2.21090800
C	-3.35224300	-0.74713900	-1.75897800
C	-3.27721700	-0.60724200	-0.38311800
H	-2.48403900	0.53190000	2.09660700
C	2.72969600	0.21680600	-2.12030100
H	2.83926700	0.10316900	-3.21239200
C	3.96633300	0.49822800	-1.50226500
C	4.03152900	0.68862900	-0.09592600
C	5.16951300	0.60285100	-2.25477500
C	5.22261600	0.97008200	0.53355900
H	3.10127500	0.61133800	0.47746900
C	6.37196600	0.88078200	-1.63827500
H	5.13011800	0.45585500	-3.33531900
C	6.38181100	1.06228600	-0.24878700
H	5.27635400	1.11829700	1.60932500
H	7.29853500	0.96263300	-2.20615300
Cl	7.88627900	1.41236400	0.53114300
Br	2.33620300	3.84671400	0.13039400
Br	-0.81930800	5.73836300	0.16875700
Br	-3.33982100	3.24938900	1.10046100
Br	1.05056000	-3.37861600	4.27193000
Br	2.54538500	-2.87234800	0.95720800
Br	-1.71295600	-1.01104000	4.40081300
Br	-4.48575900	-1.21073600	0.89126000

Br	-4.68151000	-1.57643500	-2.75412000
Br	-1.61178400	0.03857300	-3.98098500
C	2.39539500	-3.16875000	-2.97926700
H	2.40393800	-2.48415400	-2.11431700
C	1.06548900	-2.97985200	-3.70605500
H	0.96004000	-1.91790700	-3.99003800
H	1.08505900	-3.55144900	-4.65489900
C	2.56844700	-4.58492400	-2.44505000
H	3.56658600	-4.72092600	-2.00182200
H	1.83010900	-4.82707200	-1.66679300
H	2.46118100	-5.32795900	-3.25340700
C	3.55072100	-2.80875000	-3.90051100
H	3.42118000	-1.81633100	-4.35366400
H	4.51181400	-2.80910000	-3.36053200
H	3.63508700	-3.53310100	-4.72238400
C	-0.16121500	-3.41144500	-2.93868900
H	-1.07701800	-3.22194200	-3.53485800
H	-0.13051600	-4.49918200	-2.74379500
O	-0.23685300	-2.71376300	-1.71178800
C	-1.15363300	-3.31155500	-0.82984400
H	-1.22839400	-2.68187300	0.06124100
H	-2.15531100	-3.41470800	-1.28702000
H	-0.80830500	-4.31488100	-0.52630800

TS-2P

Zero-point correction= 0.398507 (Hartree/Particle)
Thermal correction to Energy= 0.444244
Thermal correction to Enthalpy= 0.445188
Thermal correction to Gibbs Free Energy= 0.309279
Sum of electronic and zero-point Energies= -25026.067999
Sum of electronic and thermal Energies= -25026.022262
Sum of electronic and thermal Enthalpies= -25026.021318
Sum of electronic and thermal Free Energies= -25026.157227

Ag	1.58736500	0.16212500	-0.76244700
N	-0.03971200	-0.32752400	1.24735700
N	-1.28671400	0.20498300	1.23133800
N	0.49689100	2.15357200	-0.35428300
N	-0.84480400	2.27075600	-0.17527500
N	-0.62942000	-0.07689400	-1.97129800
N	-1.76000700	0.13658800	-1.25385000
B	-1.76974100	1.03938000	0.01028700
C	0.07219000	-0.97408300	2.39336800

C	-1.10309700	-0.90225600	3.15109600
C	-1.94101600	-0.12828300	2.36741000
C	0.99169400	3.37881700	-0.38346900
C	-0.01291500	4.33871700	-0.22167600
C	-1.16470400	3.58368700	-0.08847500
C	-0.93452000	-0.97509100	-2.89182000
C	-2.27181800	-1.37980900	-2.79880400
C	-2.75791200	-0.64013800	-1.73494500
H	-2.88152000	1.41161800	0.21694500
C	3.21077600	-1.16694700	-1.26290300
H	3.22172500	-1.49721500	-2.31027700
C	4.55887000	-1.08956900	-0.69462100
C	4.75955000	-0.62803700	0.61801200
C	5.68459400	-1.45626000	-1.45024100
C	6.03072300	-0.53844200	1.16235500
H	3.89239000	-0.32867200	1.20956400
C	6.96753900	-1.34833700	-0.93118700
H	5.54666500	-1.82457700	-2.46709700
C	7.12221900	-0.89673900	0.37516500
H	6.18083000	-0.17963000	2.17818700
H	7.83799000	-1.61982300	-1.52464200
Cl	8.73247500	-0.76915000	1.04818900
Br	2.82145500	3.65640800	-0.60254700
Br	0.15678500	6.18638800	-0.18502100
Br	-2.88438200	4.23032300	0.19269100
Br	-1.47628400	-1.70354800	4.78359600
Br	1.66049900	-1.84433300	2.85773700
Br	-3.67547100	0.39997700	2.77621600
Br	-4.47575500	-0.69043400	-1.02770700
Br	-3.17192100	-2.63817700	-3.82487200
Br	0.34915800	-1.58091100	-4.10209000
C	-1.22626100	-4.16765700	1.28589800
H	-0.73495200	-3.47974600	1.99684700
C	-2.40090300	-3.42345700	0.66281100
H	-2.09041900	-2.47078300	0.20752700
H	-2.88664300	-4.02445100	-0.12387300
H	-3.16265100	-3.18998500	1.42108800
C	-1.70332600	-5.38484700	2.06148700
H	-0.86415300	-5.93467100	2.51029500
H	-2.38541300	-5.09363300	2.87231500
H	-2.24566400	-6.07975500	1.40233700
C	-0.17555700	-4.57541700	0.24952700
H	-0.55601300	-5.40500400	-0.36866200
H	0.71665300	-4.95731700	0.77265300

C	0.22740300	-3.42671600	-0.65072300
H	0.37527700	-2.49053600	-0.08515700
H	-0.52756300	-3.23342300	-1.42237400
O	1.43647300	-3.71954100	-1.38427000
C	2.56833700	-3.40899300	-0.76064000
H	2.59558400	-2.10170100	-0.68778600
H	2.60259200	-3.54422800	0.33474900
H	3.45621700	-3.76184400	-1.28783600

TS-2S

Zero-point correction=	0.396159 (Hartree/Particle)
Thermal correction to Energy=	0.442577
Thermal correction to Enthalpy=	0.443521
Thermal correction to Gibbs Free Energy=	0.304043
Sum of electronic and zero-point Energies=	-25026.071520
Sum of electronic and thermal Energies=	-25026.025102
Sum of electronic and thermal Enthalpies=	-25026.024158
Sum of electronic and thermal Free Energies=	-25026.163636

Ag	0.98406500	0.31638400	-0.57712600
N	-0.40293300	-0.74330500	1.36105700
N	-1.71566900	-0.40223300	1.34705700
N	-0.79847800	1.75125600	-0.42340400
N	-2.08782200	1.32651900	-0.43679800
N	-1.04193300	-0.79002100	-2.00288700
N	-1.94027800	-1.12857400	-1.04652100
B	-2.44426400	-0.11395100	0.00838300
C	-0.04741000	-0.76226900	2.63545900
C	-1.11343400	-0.44012700	3.48465300
C	-2.15788000	-0.21031000	2.60736600
C	-0.80878300	3.01822900	-0.80697500
C	-2.10861400	3.45803200	-1.07706100
C	-2.88783900	2.34095600	-0.82803100
C	-0.80444400	-1.89860900	-2.68588900
C	-1.52861500	-2.98920400	-2.18556800
C	-2.23700300	-2.44153800	-1.12981500
H	-3.62471800	-0.21089700	0.16449400
C	2.99036800	-0.21396400	-1.05885900
H	3.14239700	-0.81974200	-1.96535800
C	4.12289200	0.67000000	-0.81996500
C	4.13655500	1.54638200	0.28321800
C	5.21979700	0.69529000	-1.70172600
C	5.20133900	2.39990600	0.51089700

H	3.28260700	1.54426000	0.96355900
C	6.27812800	1.57418700	-1.51190300
H	5.22488900	0.02681900	-2.56311300
C	6.26039400	2.40665300	-0.39818500
H	5.21168700	3.06832800	1.36902100
H	7.11335400	1.60641200	-2.20818600
Cl	7.59851700	3.49651300	-0.12981200
Br	0.77704200	3.98643800	-0.94096800
Br	-2.66544000	5.14055400	-1.62880100
Br	-4.73245800	2.20376600	-0.99233500
Br	-1.13303800	-0.34135900	5.33858500
Br	1.70808800	-1.15079100	3.14736900
Br	-3.89524300	0.31019400	3.01284600
Br	-3.40192100	-3.29046900	0.04475400
Br	-1.52738600	-4.75216500	-2.77000300
Br	0.39878800	-1.89145300	-4.11409000
O	3.00006600	-3.22870300	-0.89352400
C	3.51866400	-2.41077400	0.03811600
H	3.10298300	-1.22688600	-0.19296600
H	3.01004900	-2.47676700	1.01818200
C	1.60372000	-3.47749500	-0.73627700
H	1.30630200	-4.14980100	-1.54556400
H	1.41088300	-3.96129000	0.23296300
H	1.03575000	-2.53624800	-0.79188600
C	5.01176200	-2.36888100	0.09034700
H	5.39876400	-1.85147700	-0.80307800
H	5.38083600	-3.40636900	0.02779600
C	5.52118400	-1.70331900	1.37244800
H	4.89984600	-0.80878500	1.56323600
C	6.96235900	-1.25051500	1.19998500
H	7.06238100	-0.52120900	0.38349400
H	7.34680500	-0.78576800	2.11784600
H	7.61101400	-2.10855200	0.96515100
C	5.39599600	-2.64481100	2.56389000
H	4.38037000	-3.04993400	2.68640800
H	6.07370800	-3.50331400	2.44212900
H	5.66487200	-2.13763300	3.50013200

TS-2T

Zero-point correction= 0.397773 (Hartree/Particle)
Thermal correction to Energy= 0.443312
Thermal correction to Enthalpy= 0.444256
Thermal correction to Gibbs Free Energy= 0.306529

Sum of electronic and zero-point Energies= -25026.074546
Sum of electronic and thermal Energies= -25026.029008
Sum of electronic and thermal Enthalpies= -25026.028063
Sum of electronic and thermal Free Energies= -25026.165791

Ag	1.03388600	-0.52466600	-0.11379500
N	-0.78283700	-0.79043600	1.35222000
N	-1.76750100	0.13839000	1.44210400
N	0.44786900	1.80307400	0.59593700
N	-0.82022400	2.19245000	0.30467500
N	-0.78341000	0.44669000	-1.82375300
N	-1.90764900	0.40749200	-1.06256500
B	-1.97591700	1.16592700	0.29783300
C	-0.81560700	-1.48675300	2.47663600
C	-1.82668900	-1.03465000	3.33112700
C	-2.40487100	0.00635000	2.62530300
C	1.23041700	2.82118800	0.28341800
C	0.49867200	3.90155100	-0.22547000
C	-0.80600000	3.44366000	-0.20232100
C	-1.01749700	-0.34639600	-2.85652100
C	-2.28997300	-0.92693300	-2.79711200
C	-2.81590800	-0.41569200	-1.62454800
H	-3.03164900	1.70898600	0.43798500
C	2.88988500	-1.19764700	-0.98927700
H	2.98862400	-1.03267500	-2.07125700
C	4.13929900	-0.94398600	-0.27707300
C	4.23028400	-1.13924900	1.11354100
C	5.25999300	-0.42495100	-0.94748400
C	5.38496500	-0.82592400	1.81159700
H	3.35858000	-1.51544500	1.65123800
C	6.41258400	-0.07038800	-0.25872700
H	5.21294700	-0.27701400	-2.02668800
C	6.46182000	-0.28239000	1.11415000
H	5.44651500	-0.97111700	2.88798700
H	7.26899200	0.35299800	-0.77904700
Cl	7.91487300	0.14504200	1.99175500
Br	3.07852300	2.70387000	0.51573800
Br	1.12415500	5.55838600	-0.78500500
Br	-2.33091000	4.33824900	-0.77479500
Br	-2.29148800	-1.68774700	5.00568700
Br	0.38496800	-2.88134300	2.77942900
Br	-3.83114200	1.06740600	3.16448900
Br	-4.46614500	-0.78686900	-0.85147600
Br	-3.08406900	-2.12171400	-3.97721000

Br	0.26532800	-0.57410200	-4.19483200
C	2.88882000	-3.56101700	-1.44397900
H	2.55592000	-2.38134500	-0.91716300
C	1.82867100	-3.70646300	-2.51565100
H	1.80417100	-2.79054600	-3.12680100
H	2.16923400	-4.50653800	-3.19471700
C	2.70255900	-4.32688900	-0.16258200
H	3.45819100	-4.03579100	0.58007600
H	1.70366500	-4.17437600	0.26580500
H	2.83659900	-5.40192200	-0.36335300
C	4.28679500	-3.60009100	-1.97825500
H	4.39760500	-3.01099800	-2.89816800
H	5.02429900	-3.25732800	-1.24096600
H	4.53170600	-4.64652900	-2.21962600
C	0.43079300	-4.05552400	-2.06112300
H	-0.21954700	-4.17876500	-2.94754900
H	0.42728500	-5.02785700	-1.53155700
O	-0.08122300	-3.05120100	-1.21998500
C	-1.33077400	-3.41385600	-0.69039500
H	-1.68595900	-2.58013100	-0.07505900
H	-2.06586400	-3.61172900	-1.49111700
H	-1.25327600	-4.32036500	-0.06346400

107-P

Zero-point correction= 0.405260 (Hartree/Particle)
Thermal correction to Energy= 0.450712
Thermal correction to Enthalpy= 0.451657
Thermal correction to Gibbs Free Energy= 0.315628
Sum of electronic and zero-point Energies= -25026.148975
Sum of electronic and thermal Energies= -25026.103522
Sum of electronic and thermal Enthalpies= -25026.102578
Sum of electronic and thermal Free Energies= -25026.238607

Ag	1.11609700	0.97626600	-0.59187500
N	0.25091700	-0.22685500	1.28331200
N	-1.09779300	-0.37831400	1.32805900
N	-0.81904500	2.30885500	-0.20461800
N	-1.97684200	1.61619800	-0.00723200
N	-0.50427900	-0.22282500	-1.88773300
N	-1.57210400	-0.63510200	-1.15768100
B	-2.01458300	0.07608400	0.15148200
C	0.72905300	-0.76386500	2.39414400
C	-0.29154700	-1.28447100	3.19082500

C	-1.44123400	-1.01150900	2.46805300
C	-1.14779100	3.58579600	-0.28193100
C	-2.53045400	3.77205500	-0.12879900
C	-3.01344800	2.49109700	0.04236200
C	-0.36080300	-1.08882400	-2.87516900
C	-1.33150200	-2.09962800	-2.81604200
C	-2.07621900	-1.76735400	-1.70035500
H	-3.13232600	-0.24834900	0.40176600
C	3.97579000	-0.29973800	-1.53020900
H	3.94314700	-0.19520400	-2.62533300
C	5.03468000	0.56127000	-0.91582800
C	4.75899300	1.31040500	0.22792800
C	6.33065400	0.59666100	-1.44126800
C	5.73510000	2.09557800	0.84209600
H	3.74455900	1.29634000	0.63618200
C	7.32230200	1.36628800	-0.84887100
H	6.56610400	0.01814300	-2.33707500
C	7.01243800	2.10750600	0.28712400
H	5.50690900	2.68060100	1.72868100
H	8.32598200	1.39625200	-1.26297900
Cl	8.25774000	3.08507000	1.04081300
Br	0.13852800	4.90471800	-0.55790800
Br	-3.47808900	5.36699000	-0.15274000
Br	-4.78822300	2.00385700	0.31647900
Br	-0.14683600	-2.18380400	4.80407200
Br	2.56412100	-0.78649600	2.72158200
Br	-3.18394300	-1.41807600	2.95880700
Br	-3.51318400	-2.70779900	-0.99508600
Br	-1.53109400	-3.57238700	-3.92410800
Br	1.01248900	-0.91412000	-4.11920200
C	1.14134200	-4.10838500	1.10105500
H	1.31433200	-3.28806200	1.82208100
C	-0.28130900	-3.97150900	0.57619900
H	-0.47842200	-2.95953000	0.18431000
H	-0.47147000	-4.68753700	-0.23753400
H	-1.01973000	-4.16387600	1.36800100
C	1.31564300	-5.42791900	1.83250600
H	2.33850000	-5.54775800	2.22428200
H	0.62396200	-5.51083000	2.68146500
H	1.11872900	-6.27722000	1.15860600
C	2.19458100	-3.97165600	0.00268100
H	2.19083700	-4.87076300	-0.63503000
H	3.19369000	-3.92842400	0.47532300
C	2.02151500	-2.75456900	-0.89064500

H	1.84412200	-1.83590200	-0.28926500
H	1.14584400	-2.88148900	-1.54332700
O	3.13699700	-2.57007800	-1.74485700
C	4.15559700	-1.77679300	-1.18579100
H	2.98879800	0.03022200	-1.14339800
H	4.20877500	-1.89714400	-0.08624000
H	5.11826100	-2.12717400	-1.59032200

107-T

Zero-point correction=	0.404429 (Hartree/Particle)
Thermal correction to Energy=	0.449931
Thermal correction to Enthalpy=	0.450875
Thermal correction to Gibbs Free Energy=	0.314473
Sum of electronic and zero-point Energies=	-25026.163344
Sum of electronic and thermal Energies=	-25026.117841
Sum of electronic and thermal Enthalpies=	-25026.116897
Sum of electronic and thermal Free Energies=	-25026.253300

Ag	0.00834200	1.50412700	0.37095200
N	0.34311600	-0.78483900	1.20670400
N	-0.57261300	-1.66670200	0.72250900
N	-2.11329800	0.78317400	-0.04412600
N	-2.31315700	-0.41818000	-0.64237500
N	0.18288100	0.25196900	-1.99833400
N	-0.03993800	-1.03841300	-1.63845900
B	-1.18919900	-1.47981500	-0.69553800
C	0.74150000	-1.27089100	2.37313900
C	0.09899000	-2.47555100	2.68051400
C	-0.73354100	-2.68324200	1.59535800
C	-3.25590100	1.44589400	-0.11274800
C	-4.24184700	0.68992300	-0.75486100
C	-3.59283900	-0.49232500	-1.06982900
C	1.32986500	0.24285100	-2.66512100
C	1.89914300	-1.03344600	-2.72813700
C	0.97863400	-1.81910100	-2.06193800
H	-1.64605200	-2.51916900	-1.06120900
C	2.92636600	1.82913400	0.34056000
H	2.60252700	1.46265600	-0.64467100
C	1.96933600	2.90444400	0.79306500
C	1.80323400	3.22178000	2.15822200
C	1.22986700	3.66359600	-0.14302500
C	0.97241000	4.25351600	2.57005100
H	2.34714600	2.64512000	2.90518700

C	0.38663200	4.70498400	0.26153800
H	1.36488000	3.47541600	-1.20966800
C	0.27279600	4.98667800	1.61288000
H	0.86022100	4.48601200	3.62694500
H	-0.16935600	5.28075700	-0.47562200
Cl	-0.79445300	6.27195900	2.13334000
Br	-3.39384500	3.16163400	0.59825000
Br	-6.00410000	1.15782800	-1.09727700
Br	-4.32011600	-1.97420800	-1.92042500
Br	0.31320500	-3.54675800	4.18112700
Br	2.03452400	-0.41327100	3.40997000
Br	-1.91145100	-4.09473200	1.32876400
Br	1.04755700	-3.65584600	-1.77697800
Br	3.51588800	-1.54700500	-3.47642400
Br	2.01106000	1.79538500	-3.45528700
C	4.42190600	2.19582700	0.21852000
H	2.84425100	0.96589900	1.01440400
C	5.13742000	1.03797400	-0.50538200
H	4.74890400	0.96850900	-1.53669800
H	6.20396100	1.30276000	-0.59179300
C	5.03663400	2.44272300	1.59376900
H	4.59578200	3.32890700	2.07345300
H	4.89249300	1.59326400	2.27735600
H	6.11847500	2.62424300	1.50917000
C	4.59946400	3.45110200	-0.63151600
H	4.16223600	3.32345100	-1.63327300
H	4.12799300	4.32783600	-0.16390100
H	5.66670900	3.68180300	-0.76346200
C	5.06297700	-0.34965000	0.10212900
H	5.79845200	-1.00431100	-0.40578200
H	5.34301900	-0.34045800	1.17489200
O	3.77226000	-0.89064600	-0.04770400
C	3.69023100	-2.19781200	0.43939900
H	2.64310800	-2.52141400	0.37446500
H	4.30773800	-2.89439900	-0.15718000
H	4.01517900	-2.26533900	1.49402600

107-S

Zero-point correction= 0.403589 (Hartree/Particle)
Thermal correction to Energy= 0.449696
Thermal correction to Enthalpy= 0.450641
Thermal correction to Gibbs Free Energy= 0.312973
Sum of electronic and zero-point Energies= -25026.157579

Sum of electronic and thermal Energies= -25026.111471
Sum of electronic and thermal Enthalpies= -25026.110527
Sum of electronic and thermal Free Energies= -25026.248195

Ag	1.01183600	0.67969700	0.01517300
N	-0.33151800	-0.97493400	1.33425100
N	-1.65497300	-0.91787500	1.02665700
N	-1.01498400	1.78387200	-0.09076800
N	-2.10960600	1.07713100	-0.47304600
N	-0.05192300	-0.55198100	-1.85516800
N	-1.21547700	-1.11686300	-1.44083700
B	-2.13997900	-0.46751200	-0.38004700
C	-0.26653800	-1.40067900	2.58726100
C	-1.53616400	-1.62871500	3.12909200
C	-2.39026400	-1.30160500	2.09135400
C	-1.31853800	3.06529900	-0.22156300
C	-2.62576400	3.23189000	-0.68922100
C	-3.08961500	1.93591100	-0.83380300
C	0.49748900	-1.41865700	-2.69396900
C	-0.28177000	-2.57164700	-2.83930500
C	-1.36219800	-2.33284400	-2.00879100
H	-3.25788200	-0.84066900	-0.55387400
C	3.55090800	-0.94315300	0.29180900
H	3.32232900	-1.31996900	-0.71810100
C	3.36523500	0.55081500	0.35278500
C	3.29253800	1.22108100	1.59626800
C	3.44610700	1.34706100	-0.81160200
C	3.36043100	2.60701700	1.67843400
H	3.22303100	0.63310100	2.51188100
C	3.51281000	2.73870800	-0.74024100
H	3.51349900	0.85807200	-1.78355900
C	3.48953000	3.34799700	0.50696700
H	3.31668000	3.10658200	2.64391400
H	3.58590300	3.33541800	-1.64697100
Cl	3.57919500	5.09193900	0.60759100
Br	-0.07294800	4.38494100	0.19380300
Br	-3.53028500	4.81620700	-1.02998300
Br	-4.77249500	1.41633500	-1.42293600
Br	-1.97254900	-2.23980300	4.82594300
Br	1.36573500	-1.66869200	3.45652800
Br	-4.24759900	-1.34722000	2.10332100
Br	-2.79237900	-3.46643800	-1.65917500
Br	0.03923600	-4.06142300	-3.89737200
Br	2.11131400	-1.04716900	-3.55699200

O	5.08379900	-2.79667300	0.54042400
C	4.98099000	-1.39218400	0.64062600
H	2.84899900	-1.42636600	0.98669500
H	5.18793000	-1.09495200	1.69140300
C	4.36320000	-3.51371100	1.50699200
H	4.73580600	-4.54411500	1.49502400
H	4.51881800	-3.09685300	2.51854600
H	3.28009200	-3.54233100	1.30306800
C	6.03345400	-0.79665000	-0.28857400
H	5.58946800	-0.64583200	-1.28810600
H	6.82250800	-1.55458500	-0.41843800
C	6.68786100	0.49975300	0.20124700
H	5.90515000	1.18438100	0.57231000
C	7.40637300	1.18731000	-0.95032000
H	6.70894500	1.46551500	-1.75369700
H	7.91894500	2.10061600	-0.61816900
H	8.16560600	0.51882500	-1.38529800
C	7.65878500	0.22780300	1.34354300
H	7.18189100	-0.29434800	2.18491700
H	8.49485400	-0.39893000	0.99710400
H	8.08347900	1.16261100	1.73468400

Cartesian coordinates of computed structures with single point energy calculations at SMD// B3LYP-D3(BJ)/6-31+G(d,p)-SDD(Ag) level.

AgTp^{Br3}-THF

Zero-point correction=	0.227110 (Hartree/Particle)
Thermal correction to Energy=	0.256343
Thermal correction to Enthalpy=	0.257287
Thermal correction to Gibbs Free Energy=	0.160335
Sum of electronic and zero-point Energies=	-24219.087593
Sum of electronic and thermal Energies=	-24219.058359
Sum of electronic and thermal Enthalpies=	-24219.057415
Sum of electronic and thermal Free Energies=	-24219.154367

Ag	-1.59688600	0.56942900	-1.15095000
N	-1.22419300	0.14263900	1.20592900
N	0.00213400	-0.26370900	1.61695700
N	-0.12980500	-1.32843300	-1.24658600
N	0.97150800	-1.36612400	-0.45549600
N	0.48769500	1.66341000	-0.81128900
N	1.41761500	1.10068200	0.00181500

B	1.21098900	-0.29745300	0.64431600
C	-2.03358000	0.01647600	2.24151200
C	-1.36056100	-0.47984100	3.36406700
C	-0.06239600	-0.64615200	2.91364300
C	-0.04944300	-2.36493400	-2.06284400
C	1.10821500	-3.11581200	-1.82738200
C	1.72753200	-2.43791500	-0.79060100
C	0.95235000	2.85061700	-1.15934500
C	2.20415900	3.09986700	-0.58647700
C	2.45724900	1.95428800	0.14820900
H	2.18633500	-0.58719900	1.25706300
Br	-1.38177800	-2.67558300	-3.32865200
Br	1.67482700	-4.65660800	-2.69356000
Br	3.32949500	-2.88308600	0.04027700
Br	-2.04467900	-0.82923500	5.05416500
Br	-3.83699100	0.46827200	2.09291800
Br	1.38623500	-1.29488600	3.88006000
Br	3.95905800	1.59951300	1.18412500
Br	3.27053000	4.60930300	-0.75817700
Br	-0.04306000	3.96200700	-2.27567900
C	-4.61814900	-0.18386400	-1.98768900
O	-3.63942800	0.87546600	-2.06965200
C	-4.28333600	2.15941100	-1.94313800
C	-5.75274800	1.88225400	-2.17892900
C	-5.90775700	0.49315300	-1.56668200
H	-4.70347500	-0.64621300	-2.98174200
H	-4.25608700	-0.94064000	-1.27895900
H	-4.10021700	2.55604600	-0.93138400
H	-3.82568000	2.83588800	-2.67437400
H	-6.39766100	2.63733900	-1.71677800
H	-5.96913100	1.85285000	-3.25580700
H	-5.96853900	0.56287000	-0.47088800
H	-6.79254900	-0.04455000	-1.92388500

THF

Zero-point correction=	0.116763 (Hartree/Particle)
Thermal correction to Energy=	0.120802
Thermal correction to Enthalpy=	0.121746
Thermal correction to Gibbs Free Energy=	0.089848
Sum of electronic and zero-point Energies=	-232.375278
Sum of electronic and thermal Energies=	-232.371239
Sum of electronic and thermal Enthalpies=	-232.370295
Sum of electronic and thermal Free Energies=	-232.402193

C	-1.15693500	-0.41992300	-0.13331600
O	-0.00285600	-1.24211000	-0.00511400
C	1.15300600	-0.42589400	0.13978200
C	0.72715000	0.98201700	-0.23995100
C	-0.72010800	0.98790100	0.23696000
H	-1.52268300	-0.45928800	-1.17424500
H	-1.95098600	-0.81616400	0.51476200
H	1.50353600	-0.46286700	1.18635300
H	1.95400600	-0.82860300	-0.49529500
H	1.35063500	1.75537800	0.22273900
H	0.76854300	1.11380000	-1.33049300
H	-0.75996200	1.12776400	1.32652600
H	-1.33891400	1.76225800	-0.23028600

AgTp^{Br3}

Zero-point correction= 0.109291 (Hartree/Particle)
Thermal correction to Energy= 0.134369
Thermal correction to Enthalpy= 0.135313
Thermal correction to Gibbs Free Energy= 0.046514
Sum of electronic and zero-point Energies= -23986.647649
Sum of electronic and thermal Energies= -23986.622571
Sum of electronic and thermal Enthalpies= -23986.621627
Sum of electronic and thermal Free Energies= -23986.710426

Ag	0.02336100	-2.02193500	1.31818900
N	1.12572700	-1.64473400	-0.68036400
N	0.91663700	-0.44055600	-1.27449500
N	-1.82501500	-1.21424200	0.07329300
N	-1.62888800	-0.45153800	-1.04370600
N	0.11592400	0.21455800	1.40179300
N	-0.18270600	1.02622700	0.36697500
B	-0.34804800	0.42127700	-1.02904500
C	2.36867900	-2.02153000	-0.98171900
C	2.97871100	-1.12785400	-1.87477100
C	2.03469800	-0.11203100	-1.97604400
C	-3.12802900	-1.37138800	0.21534200
C	-3.83265900	-0.56300400	-0.67387500
C	-2.82635800	0.06431100	-1.37643900
C	0.77101900	0.96027700	2.25846700
C	0.71952600	2.30789900	1.95854300
C	0.16522800	2.28877600	0.68628500
H	-0.30822700	1.28341900	-1.84393400

Br	-0.11164200	3.61964700	-0.55151700
Br	1.29004300	3.73218100	3.01562100
Br	1.71981700	0.12638600	3.64503800
Br	2.26097700	1.60890100	-2.66147500
Br	4.77812500	-1.03203400	-2.34637800
Br	3.03594600	-3.48907200	-0.08935000
Br	-3.12204800	1.31785400	-2.71597400
Br	-5.66796600	-0.34161100	-0.89241900
Br	-3.73199900	-2.49481300	1.55974300

2a-1

Zero-point correction= 0.104429 (Hartree/Particle)
Thermal correction to Energy= 0.112254
Thermal correction to Enthalpy= 0.113198
Thermal correction to Gibbs Free Energy= 0.070902
Sum of electronic and zero-point Energies= -839.347843
Sum of electronic and thermal Energies= -839.340018
Sum of electronic and thermal Enthalpies= -839.339074
Sum of electronic and thermal Free Energies= -839.381371

N	-3.35468800	-0.17482500	0.13233000
C	-2.50887900	0.74144900	-0.36236800
N	-4.05632400	-0.98532800	0.49100000
H	-2.84869500	1.74977400	-0.10718500
C	-1.06335200	0.43454700	-0.22273400
C	-0.17872400	1.49198200	0.00971400
C	-0.56213900	-0.86560900	-0.34662600
C	1.18630400	1.25908800	0.11652000
H	-0.56133600	2.50773200	0.09837200
C	0.79919400	-1.11045800	-0.22733600
H	-1.23546200	-1.69815100	-0.55059700
C	1.66087800	-0.04245400	0.00088100
H	1.87567200	2.08156600	0.29383600
H	1.18896800	-2.12164500	-0.32297900
Cl	3.37989700	-0.34408600	0.14217200

Int-1

Zero-point correction= 0.214975 (Hartree/Particle)
Thermal correction to Energy= 0.249733
Thermal correction to Enthalpy= 0.250677
Thermal correction to Gibbs Free Energy= 0.138439
Sum of electronic and zero-point Energies= -24826.061570

Sum of electronic and thermal Energies= -24826.026812
Sum of electronic and thermal Enthalpies= -24826.025868
Sum of electronic and thermal Free Energies= -24826.138105

Ag	-1.10594700	-1.21054800	-0.35456600
N	-0.93803300	1.17943700	-0.34080700
N	0.28512200	1.76250100	-0.24449900
N	0.48455500	-0.90709600	1.43266200
N	1.35932600	0.12908900	1.38157900
N	0.93365600	-0.83029700	-1.64313200
N	1.88385600	-0.03669100	-1.08795400
B	1.55936900	0.94126600	0.07129700
C	-1.80431800	2.14807100	-0.58657700
C	-1.17167300	3.39514000	-0.65501500
C	0.16020700	3.09666700	-0.43083500
C	0.54459300	-1.39153900	2.66185300
C	1.46323600	-0.68816300	3.44782500
C	1.95604500	0.27542400	2.58522100
C	1.54958300	-1.58212500	-2.53819100
C	2.91785700	-1.29676600	-2.60337900
C	3.08250200	-0.30286000	-1.65541700
H	2.47640600	1.68391400	0.21420800
Br	-0.50900300	-2.85008700	3.15058400
Br	1.93144600	-0.97660700	5.22070600
Br	3.22439200	1.57805700	2.96772900
Br	-1.94117200	5.05485400	-0.97047900
Br	-3.61769600	1.77525800	-0.79434800
Br	1.58049700	4.29652300	-0.37355900
Br	4.66867000	0.53961600	-1.17950700
Br	4.20248400	-2.06486000	-3.70145700
Br	0.61257300	-2.84826000	-3.53789000
N	-2.39368700	-3.82549900	0.05068300
C	-2.70539400	-2.80355300	-0.76057700
N	-2.11180600	-4.63924400	0.78294900
H	-2.45569600	-3.05073900	-1.79685100
C	-4.00357200	-2.13478600	-0.49464700
C	-4.73928400	-1.65498800	-1.58243800
C	-4.49238400	-1.94522200	0.80231000
C	-5.94464900	-0.99464500	-1.38246100
H	-4.36018000	-1.79140100	-2.59420500
C	-5.70308400	-1.29886600	1.01168500
H	-3.92036400	-2.29282600	1.66257700
C	-6.41641300	-0.82719600	-0.08554000
H	-6.51600000	-0.61790600	-2.22789800

H	-6.08362000	-1.15203200	2.02025000
Cl	-7.93912000	-0.00234100	0.17320400

TS-1

Zero-point correction= 0.212545 (Hartree/Particle)
Thermal correction to Energy= 0.245662
Thermal correction to Enthalpy= 0.246607
Thermal correction to Gibbs Free Energy= 0.141157
Sum of electronic and zero-point Energies= -24826.040695
Sum of electronic and thermal Energies= -24826.007577
Sum of electronic and thermal Enthalpies= -24826.006633
Sum of electronic and thermal Free Energies= -24826.112083

Ag	-1.17556300	0.49354700	1.02310000
N	-0.82588700	-1.09169500	-0.83134100
N	0.45561400	-1.35913900	-1.18947700
N	0.29007000	1.71313600	-0.38272500
N	1.21154800	1.08152900	-1.15227000
N	1.01744800	-0.49292300	1.57753800
N	1.98594700	-0.53561900	0.62892100
B	1.61818800	-0.38851500	-0.87181600
C	-1.53081000	-2.16979100	-1.13483800
C	-0.72928100	-3.17145000	-1.69568100
C	0.53408000	-2.60758100	-1.69993000
C	0.23788500	2.96546700	-0.80172500
C	1.12919800	3.19205300	-1.85598200
C	1.72864300	1.95846300	-2.04252900
C	1.63314100	-0.57773300	2.74422100
C	3.02096100	-0.68207300	2.59069200
C	3.19587500	-0.64654800	1.21812100
H	2.55933800	-0.66561800	-1.54453900
Br	-0.91096100	4.19108500	0.00736600
Br	1.44707700	4.77106000	-2.77857400
Br	3.03235000	1.53127400	-3.29544300
Br	-1.22780800	-4.85621300	-2.29629800
Br	-3.36429200	-2.24687900	-0.79735800
Br	2.12192100	-3.39477200	-2.26144300
Br	4.80030600	-0.72631300	0.28229100
Br	4.32092600	-0.83035700	3.90756500
Br	0.66784500	-0.53704500	4.34036400
N	-2.84841400	2.39922500	2.80664700
C	-2.96237200	0.68134900	2.19528700
N	-2.63860000	3.48911800	2.78477600

H	-2.94809900	0.24039900	3.20001500
C	-4.28536300	0.62885400	1.57600500
C	-5.34165500	-0.04469500	2.20758100
C	-4.49174000	1.16953800	0.29595000
C	-6.56276900	-0.20846500	1.56946900
H	-5.19553100	-0.45912900	3.20473200
C	-5.71690200	1.05227900	-0.33743300
H	-3.66584500	1.67764400	-0.20464700
C	-6.73529200	0.35197800	0.30735300
H	-7.37780800	-0.74677400	2.04766500
H	-5.87991300	1.47323500	-1.32686100
Cl	-8.27441500	0.16978500	-0.49489600

Int-2

Zero-point correction= 0.211948 (Hartree/Particle)
Thermal correction to Energy= 0.247052
Thermal correction to Enthalpy= 0.247997
Thermal correction to Gibbs Free Energy= 0.137442
Sum of electronic and zero-point Energies= -24826.068183
Sum of electronic and thermal Energies= -24826.033078
Sum of electronic and thermal Enthalpies= -24826.032134
Sum of electronic and thermal Free Energies= -24826.142689

Ag	-1.37011600	-0.29019400	-0.75323000
N	-0.08846700	1.67641200	-0.78686200
N	1.24348100	1.68170200	-0.53126600
N	-0.05510800	-0.60043600	1.25736000
N	1.12583400	0.05243500	1.38556500
N	0.97398600	-0.92355400	-1.79057900
N	1.93320500	-0.75123900	-0.84596200
B	1.93107400	0.45565300	0.12321200
C	-0.39262600	2.87479300	-1.25990900
C	0.73938900	3.69800800	-1.31748500
C	1.75473500	2.88949400	-0.84288600
C	-0.51639900	-0.78324400	2.48377100
C	0.35019300	-0.25544500	3.44688600
C	1.38197400	0.27666300	2.69241900
C	1.20587500	-2.10327300	-2.33832100
C	2.31668300	-2.73916600	-1.76735700
C	2.73892100	-1.83426800	-0.80783400
H	3.04780000	0.74335900	0.42995000
Br	-2.14314300	-1.66007100	2.77564200
Br	0.17066100	-0.26610500	5.29548300

Br	2.88377200	1.18280300	3.30131900
Br	0.84658400	5.46796700	-1.87243200
Br	-2.13499600	3.29904800	-1.75915700
Br	3.54526800	3.35357400	-0.62973700
Br	4.13852000	-2.03173500	0.40448900
Br	3.01210000	-4.41400200	-2.15636300
Br	0.09590000	-2.77117000	-3.68411400
C	-3.15357800	-1.17012300	-1.34711800
H	-3.14469700	-1.99975400	-2.07341700
C	-4.46248300	-0.90918100	-0.88638400
C	-5.57926800	-1.66459300	-1.34531400
C	-4.68784000	0.12185700	0.06556300
C	-6.85786700	-1.40254900	-0.88646800
H	-5.42209900	-2.46156400	-2.07302900
C	-5.95990900	0.39534800	0.52799600
H	-3.82658300	0.68590800	0.42838100
C	-7.02611100	-0.37047500	0.04026500
H	-7.71423600	-1.97487700	-1.23266100
H	-6.13940400	1.18508200	1.25548800
Cl	-8.62700600	-0.02522300	0.61523400

Methyl isoamyl ether

Zero-point correction=	0.193513 (Hartree/Particle)
Thermal correction to Energy=	0.202559
Thermal correction to Enthalpy=	0.203503
Thermal correction to Gibbs Free Energy=	0.159993
Sum of electronic and zero-point Energies=	-312.154710
Sum of electronic and thermal Energies=	-312.145664
Sum of electronic and thermal Enthalpies=	-312.144720
Sum of electronic and thermal Free Energies=	-312.188230

C	3.28562500	0.15255900	0.06325100
H	3.34668300	1.09780700	-0.50549100
H	3.38507700	0.39540100	1.13647600
H	4.12617900	-0.48681100	-0.22921000
O	2.10022600	-0.54065100	-0.20485900
C	0.96504300	0.21512100	0.14274900
H	0.99083200	0.46669700	1.22331800
H	0.97587700	1.17622800	-0.40627800
C	-0.27264400	-0.59073300	-0.18180100
H	-0.34772900	-0.71222900	-1.27585600
H	-0.13527900	-1.60046400	0.23601000
C	-1.57014700	0.01049400	0.35392200

H	-1.49008000	0.06205900	1.45453300
C	-1.80623500	1.42142500	-0.17084900
H	-2.77954700	1.80875200	0.16096900
H	-1.03932700	2.12937000	0.17093400
H	-1.80392900	1.43150500	-1.27221200
C	-2.74625500	-0.88845400	0.00007700
H	-2.60585900	-1.91084600	0.37744100
H	-3.68733700	-0.50327200	0.41623500
H	-2.86968700	-0.95146300	-1.09210300

Int-3S

Zero-point correction=	0.400800 (Hartree/Particle)
Thermal correction to Energy=	0.442781
Thermal correction to Enthalpy=	0.443726
Thermal correction to Gibbs Free Energy=	0.318886
Sum of electronic and zero-point Energies=	-25028.707799
Sum of electronic and thermal Energies=	-25028.665817
Sum of electronic and thermal Enthalpies=	-25028.664873
Sum of electronic and thermal Free Energies=	-25028.789713

Ag	0.89632800	0.43591000	-0.68006100
N	-0.15645500	-0.34885400	1.34420600
N	-1.50877000	-0.31903000	1.45372500
N	-1.07097600	1.72564000	-0.63619800
N	-2.28662500	1.16374300	-0.41586800
N	-1.04384700	-0.98176600	-1.84811800
N	-1.88890700	-1.30762800	-0.83922300
B	-2.39957600	-0.26017200	0.18040800
C	0.31881100	-0.26424300	2.57829900
C	-0.70964300	-0.18006000	3.52325400
C	-1.85882400	-0.21088800	2.75201900
C	-1.29240900	2.94216400	-1.10354800
C	-2.66234600	3.21417200	-1.19339600
C	-3.25481200	2.04821900	-0.74042600
C	-0.79441900	-2.10812900	-2.49428400
C	-1.46121000	-3.20011900	-1.92275700
C	-2.14343700	-2.63356400	-0.86059400
H	-3.53128300	-0.49421700	0.47529900
C	2.77042500	0.28889400	-1.55806800
H	2.92602600	-0.47362800	-2.33838200
C	3.95429100	1.01149800	-1.30045900
C	3.98418800	2.00510200	-0.28640400
C	5.15320000	0.73590300	-2.01140500

C	5.15160100	2.66885500	0.02734200
H	3.06060700	2.22616500	0.24992700
C	6.32550200	1.39981700	-1.71576700
H	5.13585400	-0.01755100	-2.79866600
C	6.31019800	2.34638700	-0.68693800
H	5.18502500	3.42130000	0.81141200
H	7.24862600	1.19053400	-2.25134500
Cl	7.79231600	3.14082900	-0.27247700
Br	0.12261000	4.06913600	-1.55241700
Br	-3.49005400	4.77288400	-1.76685400
Br	-5.07282700	1.70895800	-0.56559200
Br	-0.56762700	-0.05453200	5.36913100
Br	2.15404100	-0.23263200	2.91380000
Br	-3.61742900	-0.10426600	3.34299700
Br	-3.23478600	-3.48152200	0.38369700
Br	-1.44455700	-4.98090800	-2.44866200
Br	0.34557300	-2.11525700	-3.97341700
O	3.28738400	-3.02373500	-0.88937200
C	3.76877600	-2.39761100	0.27413500
H	3.23283300	-1.43701700	0.43033600
H	3.54337600	-3.02727000	1.15718100
C	1.92160600	-3.32022700	-0.78581800
H	1.61170800	-3.79204400	-1.72503000
H	1.71655200	-4.01714700	0.04734300
H	1.31659900	-2.40518900	-0.62263800
C	5.25367400	-2.15020500	0.13406900
H	5.42646700	-1.69437900	-0.85610600
H	5.79270500	-3.11289600	0.13409900
C	5.82700300	-1.23441100	1.21407700
H	5.27227400	-0.27726800	1.16801700
C	7.29684400	-0.94713300	0.93985500
H	7.45221100	-0.54326000	-0.07177300
H	7.70598700	-0.22085200	1.65662000
H	7.89293600	-1.86887400	1.02386100
C	5.65919600	-1.81698500	2.61214800
H	4.60531400	-1.96702300	2.88568900
H	6.15904500	-2.79552100	2.68550500
H	6.10592700	-1.15950900	3.37100200

Int-3P

Zero-point correction= 0.399900 (Hartree/Particle)
Thermal correction to Energy= 0.444036
Thermal correction to Enthalpy= 0.444981

Thermal correction to Gibbs Free Energy= 0.310192
Sum of electronic and zero-point Energies= -25028.711356
Sum of electronic and thermal Energies= -25028.667220
Sum of electronic and thermal Enthalpies= -25028.666276
Sum of electronic and thermal Free Energies= -25028.801064

Ag	-1.51006000	0.11325300	0.62511100
N	-0.15943800	-0.63697500	-1.20771300
N	1.07694900	-0.12650900	-1.45049200
N	-0.51076500	2.13317100	-0.17065600
N	0.81617400	2.20859000	-0.46054200
N	0.78984200	0.21523300	1.72539600
N	1.86469400	0.35182100	0.90709200
B	1.70909700	0.94369200	-0.51915600
C	-0.37824000	-1.55995000	-2.12877900
C	0.71506200	-1.69782100	-2.98941600
C	1.62063100	-0.76084600	-2.51790600
C	-0.96021100	3.37793000	-0.13986800
C	0.05956700	4.30754600	-0.39920000
C	1.17529100	3.50808100	-0.59541500
C	1.23405100	-0.38150500	2.82294200
C	2.60285200	-0.66528500	2.74061800
C	2.95870200	-0.17881100	1.49445900
H	2.77954500	1.23452700	-0.94610400
C	-3.25158200	-0.56117700	1.54090300
H	-3.19863000	-1.04570700	2.52924600
C	-4.58120300	-0.53819100	1.05867600
C	-4.87020600	0.02886800	-0.21571800
C	-5.65117800	-1.09745800	1.80089300
C	-6.15023000	0.03258500	-0.71781100
H	-4.04394900	0.45464000	-0.78836200
C	-6.93727200	-1.09756300	1.31345800
H	-5.43439300	-1.54331200	2.77464500
C	-7.16951300	-0.53105300	0.05600800
H	-6.37518700	0.46520600	-1.69180700
H	-7.76434900	-1.52925800	1.87818400
Cl	-8.78599100	-0.53686400	-0.57178100
Br	-2.75023100	3.74179200	0.24148800
Br	-0.05025800	6.15839500	-0.48080500
Br	2.90672700	4.09720500	-0.97215100
Br	0.93863900	-2.89369100	-4.38954600
Br	-1.97289800	-2.52424700	-2.18023200
Br	3.30886100	-0.41340300	-3.20667100
Br	4.63972400	-0.24480200	0.69960700

Br	3.67512000	-1.53681300	3.97874800
Br	0.09263400	-0.75065500	4.24746700
C	1.21988800	-4.31062700	-0.37235100
H	0.64207900	-3.88048800	-1.20787400
C	2.27958900	-3.29004900	0.03586700
H	1.85063700	-2.29777500	0.22111900
H	2.80935600	-3.60604400	0.95283100
H	3.03968200	-3.18014200	-0.75475700
C	1.87346800	-5.59189100	-0.86807600
H	1.12528300	-6.34349600	-1.15900400
H	2.51514200	-5.39954600	-1.74111900
H	2.50892800	-6.03834400	-0.08359000
C	0.24032900	-4.61657800	0.76036300
H	0.75139900	-5.18981800	1.54954400
H	-0.55587600	-5.27181300	0.36078900
C	-0.39198300	-3.37840600	1.37133500
H	-0.71149100	-2.67325600	0.57406100
H	0.34317400	-2.84578100	1.99669800
O	-1.48883600	-3.66090400	2.22075000
C	-2.67987200	-3.88409800	1.52530500
H	-2.95807900	-3.00112200	0.91238200
H	-2.63478500	-4.75599800	0.84519100
H	-3.46959000	-4.06178600	2.26175500

Int-3T

Zero-point correction=	0.400239 (Hartree/Particle)
Thermal correction to Energy=	0.442227
Thermal correction to Enthalpy=	0.443171
Thermal correction to Gibbs Free Energy=	0.316352
Sum of electronic and zero-point Energies=	-25028.712866
Sum of electronic and thermal Energies=	-25028.670878
Sum of electronic and thermal Enthalpies=	-25028.669934
Sum of electronic and thermal Free Energies=	-25028.796753

Ag	0.93448700	-0.01502000	-1.08182500
N	0.42413900	-1.06406100	1.01562000
N	-0.58920500	-0.63173300	1.82027000
N	0.37867300	1.95311300	0.91620800
N	-0.95057800	1.80399300	1.15413100
N	-1.41667900	0.28080800	-1.21251100
N	-2.12889200	-0.01176000	-0.09045600
B	-1.60766200	0.41323900	1.31895600
C	1.06759700	-2.00302500	1.69168300

C	0.49549400	-2.20886600	2.95524500
C	-0.56192400	-1.31446900	2.98953200
C	0.54112500	3.21799500	0.57634200
C	-0.66901600	3.92811700	0.56876500
C	-1.59220900	2.96328600	0.93357600
C	-2.12141900	-0.18267200	-2.23190500
C	-3.31009000	-0.79220300	-1.80915900
C	-3.26545700	-0.65663400	-0.43129200
H	-2.52966000	0.46310600	2.07458700
C	2.76209400	0.31364500	-2.05711500
H	2.88146200	0.22532300	-3.14810400
C	3.99311000	0.60874100	-1.42645100
C	4.05192500	0.76985900	-0.01766900
C	5.19646600	0.74667800	-2.17322700
C	5.23750000	1.04904500	0.62357600
H	3.12392000	0.66579600	0.54975300
C	6.39376700	1.02269500	-1.54980100
H	5.15744200	0.62196700	-3.26055400
C	6.39770500	1.16897100	-0.15389000
H	5.29109600	1.17032100	1.70133700
H	7.32300300	1.12630100	-2.11168900
Cl	7.90323600	1.50676300	0.63353900
Br	2.23392300	3.90275900	0.17794600
Br	-0.96645800	5.71433200	0.16764100
Br	-3.43498900	3.16136100	1.08420000
Br	1.02236500	-3.40727400	4.26884200
Br	2.53268000	-2.88789700	0.96742400
Br	-1.74837100	-1.04751200	4.39037400
Br	-4.50355200	-1.26788100	0.81321200
Br	-4.61208700	-1.62368400	-2.83730000
Br	-1.51800200	0.00867900	-3.98587000
C	2.56675200	-3.05931800	-3.00046800
H	2.53814600	-2.39163800	-2.12069300
C	1.23597000	-2.92013600	-3.73319300
H	1.09006600	-1.85870900	-4.00643300
H	1.29193500	-3.47870300	-4.69058300
C	2.80306100	-4.47507000	-2.49320100
H	3.80586900	-4.57548800	-2.03917800
H	2.07239100	-4.77175400	-1.72912500
H	2.74324100	-5.20828000	-3.31833400
C	3.71429500	-2.62583300	-3.90464300
H	3.54314800	-1.62823800	-4.33798700
H	4.67109100	-2.59712400	-3.35861400
H	3.83073200	-3.32694200	-4.74014600

C	0.02067200	-3.41035700	-2.98447400
H	-0.89315900	-3.25355100	-3.59956600
H	0.09354300	-4.49336600	-2.79170400
O	-0.11332200	-2.71913600	-1.75924300
C	-1.06287800	-3.32620500	-0.92246200
H	-1.17918900	-2.70017600	-0.03331500
H	-2.04464600	-3.42925200	-1.42166400
H	-0.72373900	-4.33063000	-0.61104400

TS-2P

Zero-point correction= 0.398851 (Hartree/Particle)
Thermal correction to Energy= 0.441429
Thermal correction to Enthalpy= 0.442373
Thermal correction to Gibbs Free Energy= 0.314190
Sum of electronic and zero-point Energies= -25028.701900
Sum of electronic and thermal Energies= -25028.659323
Sum of electronic and thermal Enthalpies= -25028.658379
Sum of electronic and thermal Free Energies= -25028.786561

Ag	1.58736500	0.16212500	-0.76244700
N	-0.03971200	-0.32752400	1.24735700
N	-1.28671400	0.20498300	1.23133800
N	0.49689000	2.15357200	-0.35428300
N	-0.84480500	2.27075600	-0.17527500
N	-0.62942000	-0.07689400	-1.97129800
N	-1.76000700	0.13658800	-1.25385000
B	-1.76974100	1.03938000	0.01028700
C	0.07219000	-0.97408300	2.39336800
C	-1.10309700	-0.90225600	3.15109600
C	-1.94101600	-0.12828300	2.36741000
C	0.99169300	3.37881700	-0.38346900
C	-0.01291600	4.33871700	-0.22167600
C	-1.16470500	3.58368700	-0.08847500
C	-0.93452000	-0.97509100	-2.89182000
C	-2.27181800	-1.37981000	-2.79880400
C	-2.75791200	-0.64013900	-1.73494500
H	-2.88152000	1.41161700	0.21694500
C	3.21077600	-1.16694600	-1.26290300
H	3.22172500	-1.49721400	-2.31027700
C	4.55887000	-1.08956800	-0.69462100
C	4.75955000	-0.62803600	0.61801200
C	5.68459400	-1.45625900	-1.45024100
C	6.03072300	-0.53844100	1.16235500

H	3.89239000	-0.32867100	1.20956400
C	6.96753900	-1.34833500	-0.93118700
H	5.54666500	-1.82457600	-2.46709700
C	7.12221900	-0.89673700	0.37516500
H	6.18083000	-0.17962900	2.17818700
H	7.83799000	-1.61982100	-1.52464200
Cl	8.73247500	-0.76914800	1.04818900
Br	2.82145400	3.65640900	-0.60254700
Br	0.15678400	6.18638800	-0.18502100
Br	-2.88438300	4.23032200	0.19269100
Br	-1.47628400	-1.70354800	4.78359600
Br	1.66049900	-1.84433300	2.85773700
Br	-3.67547100	0.39997600	2.77621600
Br	-4.47575500	-0.69043500	-1.02770700
Br	-3.17192000	-2.63817800	-3.82487200
Br	0.34915800	-1.58091100	-4.10209000
C	-1.22626000	-4.16765700	1.28589800
H	-0.73495100	-3.47974600	1.99684700
C	-2.40090200	-3.42345800	0.66281100
H	-2.09041800	-2.47078300	0.20752700
H	-2.88664200	-4.02445200	-0.12387300
H	-3.16265000	-3.18998600	1.42108800
C	-1.70332500	-5.38484700	2.06148700
H	-0.86415200	-5.93467100	2.51029500
H	-2.38541200	-5.09363400	2.87231500
H	-2.24566300	-6.07975600	1.40233700
C	-0.17555600	-4.57541700	0.24952700
H	-0.55601200	-5.40500400	-0.36866200
H	0.71665400	-4.95731700	0.77265300
C	0.22740400	-3.42671600	-0.65072300
H	0.37527800	-2.49053600	-0.08515700
H	-0.52756200	-3.23342300	-1.42237400
O	1.43647400	-3.71954100	-1.38427000
C	2.56833800	-3.40899200	-0.76064000
H	2.59558400	-2.10170000	-0.68778600
H	2.60259300	-3.54422700	0.33474900
H	3.45621800	-3.76184300	-1.28783600

TS-2S

Zero-point correction= 0.396809 (Hartree/Particle)
Thermal correction to Energy= 0.438042
Thermal correction to Enthalpy= 0.438986
Thermal correction to Gibbs Free Energy= 0.315307

Sum of electronic and zero-point Energies= -25028.703960
Sum of electronic and thermal Energies= -25028.662727
Sum of electronic and thermal Enthalpies= -25028.661783
Sum of electronic and thermal Free Energies= -25028.785462

Ag	0.98406500	0.31638400	-0.57712600
N	-0.40293300	-0.74330500	1.36105700
N	-1.71566900	-0.40223300	1.34705700
N	-0.79847800	1.75125600	-0.42340400
N	-2.08782200	1.32651900	-0.43679800
N	-1.04193300	-0.79002100	-2.00288700
N	-1.94027800	-1.12857400	-1.04652100
B	-2.44426400	-0.11395100	0.00838300
C	-0.04741000	-0.76226900	2.63545900
C	-1.11343400	-0.44012700	3.48465300
C	-2.15788000	-0.21031000	2.60736600
C	-0.80878300	3.01822900	-0.80697500
C	-2.10861400	3.45803200	-1.07706100
C	-2.88783900	2.34095600	-0.82803100
C	-0.80444400	-1.89860900	-2.68588900
C	-1.52861500	-2.98920400	-2.18556800
C	-2.23700300	-2.44153800	-1.12981500
H	-3.62471800	-0.21089700	0.16449400
C	2.99036800	-0.21396400	-1.05885900
H	3.14239700	-0.81974200	-1.96535800
C	4.12289200	0.67000000	-0.81996500
C	4.13655500	1.54638200	0.28321800
C	5.21979700	0.69529000	-1.70172600
C	5.20133900	2.39990600	0.51089700
H	3.28260700	1.54426000	0.96355900
C	6.27812800	1.57418700	-1.51190300
H	5.22488900	0.02681900	-2.56311300
C	6.26039400	2.40665300	-0.39818500
H	5.21168700	3.06832800	1.36902100
H	7.11335400	1.60641200	-2.20818600
Cl	7.59851700	3.49651300	-0.12981200
Br	0.77704200	3.98643800	-0.94096800
Br	-2.66544000	5.14055400	-1.62880100
Br	-4.73245800	2.20376600	-0.99233500
Br	-1.13303800	-0.34135900	5.33858500
Br	1.70808800	-1.15079100	3.14736900
Br	-3.89524300	0.31019400	3.01284600
Br	-3.40192100	-3.29046900	0.04475400
Br	-1.52738600	-4.75216500	-2.77000300

Br	0.39878800	-1.89145300	-4.11409000
O	3.00006600	-3.22870300	-0.89352400
C	3.51866400	-2.41077400	0.03811600
H	3.10298300	-1.22688600	-0.19296600
H	3.01004900	-2.47676700	1.01818200
C	1.60372000	-3.47749500	-0.73627700
H	1.30630200	-4.14980100	-1.54556400
H	1.41088300	-3.96129000	0.23296300
H	1.03575000	-2.53624800	-0.79188600
C	5.01176200	-2.36888100	0.09034700
H	5.39876400	-1.85147700	-0.80307800
H	5.38083600	-3.40636900	0.02779600
C	5.52118400	-1.70331900	1.37244800
H	4.89984600	-0.80878500	1.56323600
C	6.96235900	-1.25051500	1.19998500
H	7.06238100	-0.52120900	0.38349400
H	7.34680500	-0.78576800	2.11784600
H	7.61101400	-2.10855200	0.96515100
C	5.39599600	-2.64481100	2.56389000
H	4.38037000	-3.04993400	2.68640800
H	6.07370800	-3.50331400	2.44212900
H	5.66487200	-2.13763300	3.50013200

TS-2T

Zero-point correction= 0.397411 (Hartree/Particle)
Thermal correction to Energy= 0.439139
Thermal correction to Enthalpy= 0.440083
Thermal correction to Gibbs Free Energy= 0.315768
Sum of electronic and zero-point Energies= -25028.708389
Sum of electronic and thermal Energies= -25028.666661
Sum of electronic and thermal Enthalpies= -25028.665717
Sum of electronic and thermal Free Energies= -25028.790032

Ag	1.03388600	-0.52466600	-0.11379500
N	-0.78283700	-0.79043600	1.35222000
N	-1.76750100	0.13839000	1.44210400
N	0.44786900	1.80307400	0.59593700
N	-0.82022400	2.19245000	0.30467500
N	-0.78341000	0.44669000	-1.82375300
N	-1.90764900	0.40749200	-1.06256500
B	-1.97591700	1.16592700	0.29783300
C	-0.81560700	-1.48675300	2.47663600
C	-1.82668900	-1.03465000	3.33112700

C	-2.40487100	0.00635000	2.62530300
C	1.23041700	2.82118800	0.28341800
C	0.49867200	3.90155100	-0.22547000
C	-0.80600000	3.44366000	-0.20232100
C	-1.01749700	-0.34639600	-2.85652100
C	-2.28997300	-0.92693300	-2.79711200
C	-2.81590800	-0.41569200	-1.62454800
H	-3.03164900	1.70898600	0.43798500
C	2.88988500	-1.19764700	-0.98927700
H	2.98862400	-1.03267500	-2.07125700
C	4.13929900	-0.94398600	-0.27707300
C	4.23028400	-1.13924900	1.11354100
C	5.25999300	-0.42495100	-0.94748400
C	5.38496500	-0.82592400	1.81159700
H	3.35858000	-1.51544500	1.65123800
C	6.41258400	-0.07038800	-0.25872700
H	5.21294700	-0.27701400	-2.02668800
C	6.46182000	-0.28239000	1.11415000
H	5.44651500	-0.97111700	2.88798700
H	7.26899200	0.35299800	-0.77904700
Cl	7.91487300	0.14504200	1.99175500
Br	3.07852300	2.70387000	0.51573800
Br	1.12415500	5.55838600	-0.78500500
Br	-2.33091000	4.33824900	-0.77479500
Br	-2.29148800	-1.68774700	5.00568700
Br	0.38496800	-2.88134300	2.77942900
Br	-3.83114200	1.06740600	3.16448900
Br	-4.46614500	-0.78686900	-0.85147600
Br	-3.08406900	-2.12171400	-3.97721000
Br	0.26532800	-0.57410200	-4.19483200
C	2.88882000	-3.56101700	-1.44397900
H	2.55592000	-2.38134500	-0.91716300
C	1.82867100	-3.70646300	-2.51565100
H	1.80417100	-2.79054600	-3.12680100
H	2.16923400	-4.50653800	-3.19471700
C	2.70255900	-4.32688900	-0.16258200
H	3.45819100	-4.03579100	0.58007600
H	1.70366500	-4.17437600	0.26580500
H	2.83659900	-5.40192200	-0.36335300
C	4.28679500	-3.60009100	-1.97825500
H	4.39760500	-3.01099800	-2.89816800
H	5.02429900	-3.25732800	-1.24096600
H	4.53170600	-4.64652900	-2.21962600
C	0.43079300	-4.05552400	-2.06112300

H	-0.21954700	-4.17876500	-2.94754900
H	0.42728500	-5.02785700	-1.53155700
O	-0.08122300	-3.05120100	-1.21998500
C	-1.33077400	-3.41385600	-0.69039500
H	-1.68595900	-2.58013100	-0.07505900
H	-2.06586400	-3.61172900	-1.49111700
H	-1.25327600	-4.32036500	-0.06346400

107-P

Zero-point correction= 0.404588 (Hartree/Particle)
Thermal correction to Energy= 0.446333
Thermal correction to Enthalpy= 0.447277
Thermal correction to Gibbs Free Energy= 0.322101
Sum of electronic and zero-point Energies= -25028.779266
Sum of electronic and thermal Energies= -25028.737521
Sum of electronic and thermal Enthalpies= -25028.736577
Sum of electronic and thermal Free Energies= -25028.861753

Ag	1.11609700	0.97626600	-0.59187500
N	0.25091700	-0.22685500	1.28331200
N	-1.09779300	-0.37831400	1.32805900
N	-0.81904500	2.30885500	-0.20461800
N	-1.97684200	1.61619800	-0.00723200
N	-0.50427900	-0.22282500	-1.88773300
N	-1.57210400	-0.63510200	-1.15768100
B	-2.01458300	0.07608400	0.15148200
C	0.72905300	-0.76386500	2.39414400
C	-0.29154700	-1.28447100	3.19082500
C	-1.44123400	-1.01150900	2.46805300
C	-1.14779100	3.58579600	-0.28193100
C	-2.53045400	3.77205500	-0.12879900
C	-3.01344800	2.49109700	0.04236200
C	-0.36080300	-1.08882400	-2.87516900
C	-1.33150200	-2.09962800	-2.81604200
C	-2.07621900	-1.76735400	-1.70035500
H	-3.13232600	-0.24834900	0.40176600
C	3.97579000	-0.29973800	-1.53020900
H	3.94314700	-0.19520400	-2.62533300
C	5.03468000	0.56127000	-0.91582800
C	4.75899300	1.31040500	0.22792800
C	6.33065400	0.59666100	-1.44126800
C	5.73510000	2.09557800	0.84209600
H	3.74455900	1.29634000	0.63618200

C	7.32230200	1.36628800	-0.84887100
H	6.56610400	0.01814300	-2.33707500
C	7.01243800	2.10750600	0.28712400
H	5.50690900	2.68060100	1.72868100
H	8.32598200	1.39625200	-1.26297900
Cl	8.25774000	3.08507000	1.04081300
Br	0.13852800	4.90471800	-0.55790800
Br	-3.47808900	5.36699000	-0.15274000
Br	-4.78822300	2.00385700	0.31647900
Br	-0.14683600	-2.18380400	4.80407200
Br	2.56412100	-0.78649600	2.72158200
Br	-3.18394300	-1.41807600	2.95880700
Br	-3.51318400	-2.70779900	-0.99508600
Br	-1.53109400	-3.57238700	-3.92410800
Br	1.01248900	-0.91412000	-4.11920200
C	1.14134200	-4.10838500	1.10105500
H	1.31433200	-3.28806200	1.82208100
C	-0.28130900	-3.97150900	0.57619900
H	-0.47842200	-2.95953000	0.18431000
H	-0.47147000	-4.68753700	-0.23753400
H	-1.01973000	-4.16387600	1.36800100
C	1.31564300	-5.42791900	1.83250600
H	2.33850000	-5.54775800	2.22428200
H	0.62396200	-5.51083000	2.68146500
H	1.11872900	-6.27722000	1.15860600
C	2.19458100	-3.97165600	0.00268100
H	2.19083700	-4.87076300	-0.63503000
H	3.19369000	-3.92842400	0.47532300
C	2.02151500	-2.75456900	-0.89064500
H	1.84412200	-1.83590200	-0.28926500
H	1.14584400	-2.88148900	-1.54332700
O	3.13699700	-2.57007800	-1.74485700
C	4.15559700	-1.77679300	-1.18579100
H	2.98879800	0.03022200	-1.14339800
H	4.20877500	-1.89714400	-0.08624000
H	5.11826100	-2.12717400	-1.59032200

107-T

Zero-point correction= 0.403886 (Hartree/Particle)
Thermal correction to Energy= 0.444829
Thermal correction to Enthalpy= 0.445773
Thermal correction to Gibbs Free Energy= 0.323987
Sum of electronic and zero-point Energies= -25028.788492

Sum of electronic and thermal Energies= -25028.747549
Sum of electronic and thermal Enthalpies= -25028.746605
Sum of electronic and thermal Free Energies= -25028.868391

Ag	0.00834200	1.50412700	0.37095200
N	0.34311600	-0.78483900	1.20670400
N	-0.57261300	-1.66670200	0.72250900
N	-2.11329800	0.78317400	-0.04412600
N	-2.31315700	-0.41818000	-0.64237500
N	0.18288100	0.25196900	-1.99833400
N	-0.03993800	-1.03841300	-1.63845900
B	-1.18919900	-1.47981500	-0.69553800
C	0.74150000	-1.27089100	2.37313900
C	0.09899000	-2.47555100	2.68051400
C	-0.73354100	-2.68324200	1.59535800
C	-3.25590100	1.44589400	-0.11274800
C	-4.24184700	0.68992400	-0.75486100
C	-3.59283900	-0.49232500	-1.06982900
C	1.32986500	0.24285100	-2.66512100
C	1.89914300	-1.03344600	-2.72813700
C	0.97863400	-1.81910100	-2.06193800
H	-1.64605200	-2.51916900	-1.06120900
C	2.92636600	1.82913400	0.34056000
H	2.60252700	1.46265600	-0.64467100
C	1.96933600	2.90444400	0.79306500
C	1.80323400	3.22178000	2.15822200
C	1.22986800	3.66359600	-0.14302500
C	0.97241100	4.25351600	2.57005100
H	2.34714600	2.64512000	2.90518700
C	0.38663300	4.70498400	0.26153800
H	1.36488000	3.47541600	-1.20966800
C	0.27279700	4.98667800	1.61288000
H	0.86022200	4.48601200	3.62694500
H	-0.16935500	5.28075700	-0.47562200
Cl	-0.79445200	6.27195900	2.13334000
Br	-3.39384500	3.16163400	0.59825000
Br	-6.00410000	1.15782900	-1.09727700
Br	-4.32011600	-1.97420700	-1.92042500
Br	0.31320500	-3.54675800	4.18112700
Br	2.03452400	-0.41327100	3.40997000
Br	-1.91145200	-4.09473200	1.32876400
Br	1.04755700	-3.65584600	-1.77697800
Br	3.51588800	-1.54700600	-3.47642400
Br	2.01106000	1.79538500	-3.45528700

C	4.42190600	2.19582600	0.21852000
H	2.84425100	0.96589900	1.01440400
C	5.13742000	1.03797300	-0.50538200
H	4.74890400	0.96850800	-1.53669800
H	6.20396100	1.30275900	-0.59179300
C	5.03663400	2.44272200	1.59376900
H	4.59578200	3.32890600	2.07345300
H	4.89249300	1.59326300	2.27735600
H	6.11847500	2.62424200	1.50917000
C	4.59946400	3.45110100	-0.63151600
H	4.16223600	3.32345000	-1.63327300
H	4.12799400	4.32783500	-0.16390100
H	5.66671000	3.68180200	-0.76346200
C	5.06297700	-0.34965100	0.10212900
H	5.79845200	-1.00431200	-0.40578200
H	5.34301900	-0.34045900	1.17489200
O	3.77226000	-0.89064700	-0.04770400
C	3.69023100	-2.19781300	0.43939900
H	2.64310800	-2.52141400	0.37446500
H	4.30773800	-2.89440000	-0.15718000
H	4.01517900	-2.26534000	1.49402600

107-S

Zero-point correction= 0.403694 (Hartree/Particle)
Thermal correction to Energy= 0.446723
Thermal correction to Enthalpy= 0.447668
Thermal correction to Gibbs Free Energy= 0.315129
Sum of electronic and zero-point Energies= -25028.784450
Sum of electronic and thermal Energies= -25028.741421
Sum of electronic and thermal Enthalpies= -25028.740477
Sum of electronic and thermal Free Energies= -25028.873015

Ag	1.01183600	0.67969700	0.01517300
N	-0.33151800	-0.97493400	1.33425100
N	-1.65497300	-0.91787500	1.02665700
N	-1.01498400	1.78387200	-0.09076800
N	-2.10960600	1.07713100	-0.47304600
N	-0.05192300	-0.55198100	-1.85516800
N	-1.21547700	-1.11686300	-1.44083700
B	-2.13997900	-0.46751200	-0.38004700
C	-0.26653800	-1.40067900	2.58726100
C	-1.53616400	-1.62871500	3.12909200
C	-2.39026400	-1.30160500	2.09135400

C	-1.31853800	3.06529900	-0.22156300
C	-2.62576400	3.23189000	-0.68922100
C	-3.08961500	1.93591100	-0.83380300
C	0.49748900	-1.41865700	-2.69396900
C	-0.28177000	-2.57164700	-2.83930500
C	-1.36219800	-2.33284400	-2.00879100
H	-3.25788200	-0.84066900	-0.55387400
C	3.55090800	-0.94315300	0.29180900
H	3.32232900	-1.31996900	-0.71810100
C	3.36523500	0.55081500	0.35278500
C	3.29253800	1.22108100	1.59626800
C	3.44610700	1.34706100	-0.81160200
C	3.36043100	2.60701700	1.67843400
H	3.22303100	0.63310100	2.51188100
C	3.51281000	2.73870800	-0.74024100
H	3.51349900	0.85807200	-1.78355900
C	3.48953000	3.34799700	0.50696700
H	3.31668000	3.10658200	2.64391400
H	3.58590300	3.33541800	-1.64697100
Cl	3.57919500	5.09193900	0.60759100
Br	-0.07294800	4.38494100	0.19380300
Br	-3.53028500	4.81620700	-1.02998300
Br	-4.77249500	1.41633500	-1.42293600
Br	-1.97254900	-2.23980300	4.82594300
Br	1.36573500	-1.66869200	3.45652800
Br	-4.24759900	-1.34722000	2.10332100
Br	-2.79237900	-3.46643800	-1.65917500
Br	0.03923600	-4.06142300	-3.89737200
Br	2.11131400	-1.04716900	-3.55699200
O	5.08379900	-2.79667300	0.54042400
C	4.98099000	-1.39218400	0.64062600
H	2.84899900	-1.42636600	0.98669500
H	5.18793000	-1.09495200	1.69140300
C	4.36320000	-3.51371100	1.50699200
H	4.73580600	-4.54411500	1.49502400
H	4.51881800	-3.09685300	2.51854600
H	3.28009200	-3.54233100	1.30306800
C	6.03345400	-0.79665000	-0.28857400
H	5.58946800	-0.64583200	-1.28810600
H	6.82250800	-1.55458500	-0.41843800
C	6.68786100	0.49975300	0.20124700
H	5.90515000	1.18438100	0.57231000
C	7.40637300	1.18731000	-0.95032000
H	6.70894500	1.46551500	-1.75369700

H	7.91894500	2.10061600	-0.61816900
H	8.16560600	0.51882500	-1.38529800
C	7.65878500	0.22780300	1.34354300
H	7.18189100	-0.29434800	2.18491700
H	8.49485400	-0.39893000	0.99710400
H	8.08347900	1.16261100	1.73468400

Cartesian coordinates of computed structures with single point energy calculations at SMD// M06/6-31+G(d,p)-SDD(Ag) level.

AgTp^{Br3}-THF

Zero-point correction=	0.226688 (Hartree/Particle)
Thermal correction to Energy=	0.260764
Thermal correction to Enthalpy=	0.261708
Thermal correction to Gibbs Free Energy=	0.149169
Sum of electronic and zero-point Energies=	-24216.922226
Sum of electronic and thermal Energies=	-24216.888150
Sum of electronic and thermal Enthalpies=	-24216.887206
Sum of electronic and thermal Free Energies=	-24216.999745

Ag	-1.59688600	0.56942900	-1.15095000
N	-1.22419300	0.14263900	1.20592900
N	0.00213400	-0.26370900	1.61695700
N	-0.12980500	-1.32843300	-1.24658600
N	0.97150800	-1.36612400	-0.45549600
N	0.48769500	1.66341000	-0.81128900
N	1.41761500	1.10068200	0.00181500
B	1.21098900	-0.29745300	0.64431600
C	-2.03358000	0.01647600	2.24151200
C	-1.36056100	-0.47984100	3.36406700
C	-0.06239600	-0.64615200	2.91364300
C	-0.04944300	-2.36493400	-2.06284400
C	1.10821500	-3.11581200	-1.82738200
C	1.72753200	-2.43791500	-0.79060100
C	0.95235000	2.85061700	-1.15934500
C	2.20415900	3.09986700	-0.58647700
C	2.45724900	1.95428800	0.14820900
H	2.18633500	-0.58719900	1.25706300
Br	-1.38177800	-2.67558300	-3.32865200
Br	1.67482700	-4.65660800	-2.69356000
Br	3.32949500	-2.88308600	0.04027700
Br	-2.04467900	-0.82923500	5.05416500

Br	-3.83699100	0.46827200	2.09291800
Br	1.38623500	-1.29488600	3.88006000
Br	3.95905800	1.59951300	1.18412500
Br	3.27053000	4.60930300	-0.75817700
Br	-0.04306000	3.96200700	-2.27567900
C	-4.61814900	-0.18386400	-1.98768900
O	-3.63942800	0.87546600	-2.06965200
C	-4.28333600	2.15941100	-1.94313800
C	-5.75274800	1.88225400	-2.17892900
C	-5.90775700	0.49315300	-1.56668200
H	-4.70347500	-0.64621300	-2.98174200
H	-4.25608700	-0.94064000	-1.27895900
H	-4.10021700	2.55604600	-0.93138400
H	-3.82568000	2.83588800	-2.67437400
H	-6.39766100	2.63733900	-1.71677800
H	-5.96913100	1.85285000	-3.25580700
H	-5.96853900	0.56287000	-0.47088800
H	-6.79254900	-0.04455000	-1.92388500

THF

Zero-point correction= 0.115900 (Hartree/Particle)
Thermal correction to Energy= 0.120884
Thermal correction to Enthalpy= 0.121829
Thermal correction to Gibbs Free Energy= 0.086818
Sum of electronic and zero-point Energies= -232.194695
Sum of electronic and thermal Energies= -232.189710
Sum of electronic and thermal Enthalpies= -232.188766
Sum of electronic and thermal Free Energies= -232.223777

C	-1.15693500	-0.41992300	-0.13331600
O	-0.00285600	-1.24211000	-0.00511400
C	1.15300600	-0.42589400	0.13978200
C	0.72715000	0.98201700	-0.23995100
C	-0.72010800	0.98790100	0.23696000
H	-1.52268300	-0.45928800	-1.17424500
H	-1.95098600	-0.81616400	0.51476200
H	1.50353600	-0.46286700	1.18635300
H	1.95400600	-0.82860300	-0.49529500
H	1.35063500	1.75537800	0.22273900
H	0.76854300	1.11380000	-1.33049300
H	-0.75996200	1.12776400	1.32652600
H	-1.33891400	1.76225800	-0.23028600

AgTp^{Br3}

Zero-point correction=	0.108641 (Hartree/Particle)
Thermal correction to Energy=	0.134628
Thermal correction to Enthalpy=	0.135573
Thermal correction to Gibbs Free Energy=	0.043436
Sum of electronic and zero-point Energies=	-23984.669593
Sum of electronic and thermal Energies=	-23984.643605
Sum of electronic and thermal Enthalpies=	-23984.642661
Sum of electronic and thermal Free Energies=	-23984.734798

Ag	0.02336100	-2.02193500	1.31818900
N	1.12572700	-1.64473400	-0.68036400
N	0.91663700	-0.44055600	-1.27449500
N	-1.82501500	-1.21424200	0.07329300
N	-1.62888800	-0.45153800	-1.04370600
N	0.11592400	0.21455800	1.40179300
N	-0.18270600	1.02622700	0.36697500
B	-0.34804800	0.42127700	-1.02904500
C	2.36867900	-2.02153000	-0.98171900
C	2.97871100	-1.12785400	-1.87477100
C	2.03469800	-0.11203100	-1.97604400
C	-3.12802900	-1.37138800	0.21534200
C	-3.83265900	-0.56300400	-0.67387500
C	-2.82635800	0.06431100	-1.37643900
C	0.77101900	0.96027700	2.25846700
C	0.71952600	2.30789900	1.95854300
C	0.16522800	2.28877600	0.68628500
H	-0.30822700	1.28341900	-1.84393400
Br	-0.11164200	3.61964700	-0.55151700
Br	1.29004300	3.73218100	3.01562100
Br	1.71981700	0.12638600	3.64503800
Br	2.26097700	1.60890100	-2.66147500
Br	4.77812500	-1.03203400	-2.34637800
Br	3.03594600	-3.48907200	-0.08935000
Br	-3.12204800	1.31785400	-2.71597400
Br	-5.66796600	-0.34161100	-0.89241900
Br	-3.73199900	-2.49481300	1.55974300

2a-1

Zero-point correction=	0.103690 (Hartree/Particle)
Thermal correction to Energy=	0.112405
Thermal correction to Enthalpy=	0.113349

Thermal correction to Gibbs Free Energy= 0.068595
Sum of electronic and zero-point Energies= -839.010225
Sum of electronic and thermal Energies= -839.001510
Sum of electronic and thermal Enthalpies= -839.000566
Sum of electronic and thermal Free Energies= -839.045320

N	-3.35468800	-0.17482500	0.13233000
C	-2.50887900	0.74144900	-0.36236800
N	-4.05632400	-0.98532800	0.49100000
H	-2.84869500	1.74977400	-0.10718500
C	-1.06335200	0.43454700	-0.22273400
C	-0.17872400	1.49198200	0.00971400
C	-0.56213900	-0.86560900	-0.34662600
C	1.18630400	1.25908800	0.11652000
H	-0.56133600	2.50773200	0.09837200
C	0.79919400	-1.11045800	-0.22733600
H	-1.23546200	-1.69815100	-0.55059700
C	1.66087800	-0.04245400	0.00088100
H	1.87567200	2.08156600	0.29383600
H	1.18896800	-2.12164500	-0.32297900
Cl	3.37989700	-0.34408600	0.14217200

Int-1

Zero-point correction= 0.214368 (Hartree/Particle)
Thermal correction to Energy= 0.253294
Thermal correction to Enthalpy= 0.254238
Thermal correction to Gibbs Free Energy= 0.128979
Sum of electronic and zero-point Energies= -24823.738213
Sum of electronic and thermal Energies= -24823.699287
Sum of electronic and thermal Enthalpies= -24823.698343
Sum of electronic and thermal Free Energies= -24823.823602

Ag	-1.10594700	-1.21054800	-0.35456600
N	-0.93803300	1.17943700	-0.34080700
N	0.28512200	1.76250100	-0.24449900
N	0.48455500	-0.90709600	1.43266200
N	1.35932600	0.12908900	1.38157900
N	0.93365600	-0.83029700	-1.64313200
N	1.88385600	-0.03669100	-1.08795400
B	1.55936900	0.94126600	0.07129700
C	-1.80431800	2.14807100	-0.58657700
C	-1.17167300	3.39514000	-0.65501500
C	0.16020700	3.09666700	-0.43083500

C	0.54459300	-1.39153900	2.66185300
C	1.46323600	-0.68816300	3.44782500
C	1.95604500	0.27542400	2.58522100
C	1.54958300	-1.58212500	-2.53819100
C	2.91785700	-1.29676600	-2.60337900
C	3.08250200	-0.30286000	-1.65541700
H	2.47640600	1.68391400	0.21420800
Br	-0.50900300	-2.85008700	3.15058400
Br	1.93144600	-0.97660700	5.22070600
Br	3.22439200	1.57805700	2.96772900
Br	-1.94117200	5.05485400	-0.97047900
Br	-3.61769600	1.77525800	-0.79434800
Br	1.58049700	4.29652300	-0.37355900
Br	4.66867000	0.53961600	-1.17950700
Br	4.20248400	-2.06486000	-3.70145700
Br	0.61257300	-2.84826000	-3.53789000
N	-2.39368700	-3.82549900	0.05068300
C	-2.70539400	-2.80355300	-0.76057700
N	-2.11180600	-4.63924400	0.78294900
H	-2.45569600	-3.05073900	-1.79685100
C	-4.00357200	-2.13478600	-0.49464700
C	-4.73928400	-1.65498800	-1.58243800
C	-4.49238400	-1.94522200	0.80231000
C	-5.94464900	-0.99464500	-1.38246100
H	-4.36018000	-1.79140100	-2.59420500
C	-5.70308400	-1.29886600	1.01168500
H	-3.92036400	-2.29282600	1.66257700
C	-6.41641300	-0.82719600	-0.08554000
H	-6.51600000	-0.61790600	-2.22789800
H	-6.08362000	-1.15203200	2.02025000
Cl	-7.93912000	-0.00234100	0.17320400

TS-1

Zero-point correction= 0.212026 (Hartree/Particle)
Thermal correction to Energy= 0.250198
Thermal correction to Enthalpy= 0.251143
Thermal correction to Gibbs Free Energy= 0.129189
Sum of electronic and zero-point Energies= -24823.715755
Sum of electronic and thermal Energies= -24823.677583
Sum of electronic and thermal Enthalpies= -24823.676638
Sum of electronic and thermal Free Energies= -24823.798592

Ag	-1.17556300	0.49354700	1.02310000

N	-0.82588700	-1.09169500	-0.83134100
N	0.45561400	-1.35913900	-1.18947700
N	0.29007000	1.71313600	-0.38272500
N	1.21154800	1.08152900	-1.15227000
N	1.01744800	-0.49292300	1.57753800
N	1.98594700	-0.53561900	0.62892100
B	1.61818800	-0.38851500	-0.87181600
C	-1.53081000	-2.16979100	-1.13483800
C	-0.72928100	-3.17145000	-1.69568100
C	0.53408000	-2.60758100	-1.69993000
C	0.23788500	2.96546700	-0.80172500
C	1.12919800	3.19205300	-1.85598200
C	1.72864300	1.95846300	-2.04252900
C	1.63314100	-0.57773300	2.74422100
C	3.02096100	-0.68207300	2.59069200
C	3.19587500	-0.64654800	1.21812100
H	2.55933800	-0.66561800	-1.54453900
Br	-0.91096100	4.19108500	0.00736600
Br	1.44707700	4.77106000	-2.77857400
Br	3.03235000	1.53127400	-3.29544300
Br	-1.22780800	-4.85621300	-2.29629800
Br	-3.36429200	-2.24687900	-0.79735800
Br	2.12192100	-3.39477200	-2.26144300
Br	4.80030600	-0.72631300	0.28229100
Br	4.32092600	-0.83035700	3.90756500
Br	0.66784500	-0.53704500	4.34036400
N	-2.84841400	2.39922500	2.80664700
C	-2.96237200	0.68134900	2.19528700
N	-2.63860000	3.48911800	2.78477600
H	-2.94809900	0.24039900	3.20001500
C	-4.28536300	0.62885400	1.57600500
C	-5.34165500	-0.04469500	2.20758100
C	-4.49174000	1.16953800	0.29595000
C	-6.56276900	-0.20846500	1.56946900
H	-5.19553100	-0.45912900	3.20473200
C	-5.71690200	1.05227900	-0.33743300
H	-3.66584500	1.67764400	-0.20464700
C	-6.73529200	0.35197800	0.30735300
H	-7.37780800	-0.74677400	2.04766500
H	-5.87991300	1.47323500	-1.32686100
Cl	-8.27441500	0.16978500	-0.49489600

Int-2

Zero-point correction= 0.211808 (Hartree/Particle)
Thermal correction to Energy= 0.251876
Thermal correction to Enthalpy= 0.252820
Thermal correction to Gibbs Free Energy= 0.123605
Sum of electronic and zero-point Energies= -24823.742723
Sum of electronic and thermal Energies= -24823.702655
Sum of electronic and thermal Enthalpies= -24823.701711
Sum of electronic and thermal Free Energies= -24823.830926

Ag	-1.37011600	-0.29019400	-0.75323000
N	-0.08846700	1.67641200	-0.78686200
N	1.24348100	1.68170200	-0.53126600
N	-0.05510800	-0.60043600	1.25736000
N	1.12583400	0.05243500	1.38556500
N	0.97398600	-0.92355400	-1.79057900
N	1.93320500	-0.75123900	-0.84596200
B	1.93107400	0.45565300	0.12321200
C	-0.39262600	2.87479300	-1.25990900
C	0.73938900	3.69800800	-1.31748500
C	1.75473500	2.88949400	-0.84288600
C	-0.51639900	-0.78324400	2.48377100
C	0.35019300	-0.25544500	3.44688600
C	1.38197400	0.27666300	2.69241900
C	1.20587500	-2.10327300	-2.33832100
C	2.31668300	-2.73916600	-1.76735700
C	2.73892100	-1.83426800	-0.80783400
H	3.04780000	0.74335900	0.42995000
Br	-2.14314300	-1.66007100	2.77564200
Br	0.17066100	-0.26610500	5.29548300
Br	2.88377200	1.18280300	3.30131900
Br	0.84658400	5.46796700	-1.87243200
Br	-2.13499600	3.29904800	-1.75915700
Br	3.54526800	3.35357400	-0.62973700
Br	4.13852000	-2.03173500	0.40448900
Br	3.01210000	-4.41400200	-2.15636300
Br	0.09590000	-2.77117000	-3.68411400
C	-3.15357800	-1.17012300	-1.34711800
H	-3.14469700	-1.99975400	-2.07341700
C	-4.46248300	-0.90918100	-0.88638400
C	-5.57926800	-1.66459300	-1.34531400
C	-4.68784000	0.12185700	0.06556300
C	-6.85786700	-1.40254900	-0.88646800
H	-5.42209900	-2.46156400	-2.07302900
C	-5.95990900	0.39534800	0.52799600

H	-3.82658300	0.68590800	0.42838100
C	-7.02611100	-0.37047500	0.04026500
H	-7.71423600	-1.97487700	-1.23266100
H	-6.13940400	1.18508200	1.25548800
Cl	-8.62700600	-0.02522300	0.61523400

Methyl isoamyl ether

Zero-point correction= 0.192233 (Hartree/Particle)
Thermal correction to Energy= 0.201247
Thermal correction to Enthalpy= 0.202191
Thermal correction to Gibbs Free Energy= 0.159086
Sum of electronic and zero-point Energies= -311.889424
Sum of electronic and thermal Energies= -311.880410
Sum of electronic and thermal Enthalpies= -311.879466
Sum of electronic and thermal Free Energies= -311.922571

C	3.28562500	0.15255900	0.06325100
H	3.34668300	1.09780700	-0.50549100
H	3.38507700	0.39540100	1.13647600
H	4.12617900	-0.48681100	-0.22921000
O	2.10022600	-0.54065100	-0.20485900
C	0.96504300	0.21512100	0.14274900
H	0.99083200	0.46669700	1.22331800
H	0.97587700	1.17622800	-0.40627800
C	-0.27264400	-0.59073300	-0.18180100
H	-0.34772900	-0.71222900	-1.27585600
H	-0.13527900	-1.60046400	0.23601000
C	-1.57014700	0.01049400	0.35392200
H	-1.49008000	0.06205900	1.45453300
C	-1.80623500	1.42142500	-0.17084900
H	-2.77954700	1.80875200	0.16096900
H	-1.03932700	2.12937000	0.17093400
H	-1.80392900	1.43150500	-1.27221200
C	-2.74625500	-0.88845400	0.00007700
H	-2.60585900	-1.91084600	0.37744100
H	-3.68733700	-0.50327200	0.41623500
H	-2.86968700	-0.95146300	-1.09210300

Int-3S

Zero-point correction= 0.399345 (Hartree/Particle)
Thermal correction to Energy= 0.446217
Thermal correction to Enthalpy= 0.447162

Thermal correction to Gibbs Free Energy= 0.306427
Sum of electronic and zero-point Energies= -25026.180968
Sum of electronic and thermal Energies= -25026.134095
Sum of electronic and thermal Enthalpies= -25026.133151
Sum of electronic and thermal Free Energies= -25026.273886

Ag	0.89632800	0.43591000	-0.68006100
N	-0.15645500	-0.34885400	1.34420600
N	-1.50877000	-0.31903000	1.45372500
N	-1.07097600	1.72564000	-0.63619800
N	-2.28662500	1.16374300	-0.41586800
N	-1.04384700	-0.98176600	-1.84811800
N	-1.88890700	-1.30762800	-0.83922300
B	-2.39957600	-0.26017200	0.18040800
C	0.31881100	-0.26424300	2.57829900
C	-0.70964300	-0.18006000	3.52325400
C	-1.85882400	-0.21088800	2.75201900
C	-1.29240900	2.94216400	-1.10354800
C	-2.66234600	3.21417200	-1.19339600
C	-3.25481200	2.04821900	-0.74042600
C	-0.79441900	-2.10812900	-2.49428400
C	-1.46121000	-3.20011900	-1.92275700
C	-2.14343700	-2.63356400	-0.86059400
H	-3.53128300	-0.49421700	0.47529900
C	2.77042500	0.28889400	-1.55806800
H	2.92602600	-0.47362800	-2.33838200
C	3.95429100	1.01149800	-1.30045900
C	3.98418800	2.00510200	-0.28640400
C	5.15320000	0.73590300	-2.01140500
C	5.15160100	2.66885500	0.02734200
H	3.06060700	2.22616500	0.24992700
C	6.32550200	1.39981700	-1.71576700
H	5.13585400	-0.01755100	-2.79866600
C	6.31019800	2.34638700	-0.68693800
H	5.18502500	3.42130000	0.81141200
H	7.24862600	1.19053400	-2.25134500
Cl	7.79231600	3.14082900	-0.27247700
Br	0.12261000	4.06913600	-1.55241700
Br	-3.49005400	4.77288400	-1.76685400
Br	-5.07282700	1.70895800	-0.56559200
Br	-0.56762700	-0.05453200	5.36913100
Br	2.15404100	-0.23263200	2.91380000
Br	-3.61742900	-0.10426600	3.34299700
Br	-3.23478600	-3.48152200	0.38369700

Br	-1.44455700	-4.98090800	-2.44866200
Br	0.34557300	-2.11525700	-3.97341700
O	3.28738400	-3.02373500	-0.88937200
C	3.76877600	-2.39761100	0.27413500
H	3.23283300	-1.43701700	0.43033600
H	3.54337600	-3.02727000	1.15718100
C	1.92160600	-3.32022700	-0.78581800
H	1.61170800	-3.79204400	-1.72503000
H	1.71655200	-4.01714700	0.04734300
H	1.31659900	-2.40518900	-0.62263800
C	5.25367400	-2.15020500	0.13406900
H	5.42646700	-1.69437900	-0.85610600
H	5.79270500	-3.11289600	0.13409900
C	5.82700300	-1.23441100	1.21407700
H	5.27227400	-0.27726800	1.16801700
C	7.29684400	-0.94713300	0.93985500
H	7.45221100	-0.54326000	-0.07177300
H	7.70598700	-0.22085200	1.65662000
H	7.89293600	-1.86887400	1.02386100
C	5.65919600	-1.81698500	2.61214800
H	4.60531400	-1.96702300	2.88568900
H	6.15904500	-2.79552100	2.68550500
H	6.10592700	-1.15950900	3.37100200

Int-3P

Zero-point correction= 0.397920 (Hartree/Particle)
Thermal correction to Energy= 0.445065
Thermal correction to Enthalpy= 0.446009
Thermal correction to Gibbs Free Energy= 0.304072
Sum of electronic and zero-point Energies= -25026.186447
Sum of electronic and thermal Energies= -25026.139301
Sum of electronic and thermal Enthalpies= -25026.138357
Sum of electronic and thermal Free Energies= -25026.280294

Ag	-1.51006000	0.11325300	0.62511100
N	-0.15943800	-0.63697500	-1.20771300
N	1.07694900	-0.12650900	-1.45049200
N	-0.51076500	2.13317100	-0.17065600
N	0.81617400	2.20859000	-0.46054200
N	0.78984200	0.21523300	1.72539600
N	1.86469400	0.35182100	0.90709200
B	1.70909700	0.94369200	-0.51915600
C	-0.37824000	-1.55995000	-2.12877900

C	0.71506200	-1.69782100	-2.98941600
C	1.62063100	-0.76084600	-2.51790600
C	-0.96021100	3.37793000	-0.13986800
C	0.05956700	4.30754600	-0.39920000
C	1.17529100	3.50808100	-0.59541500
C	1.23405100	-0.38150500	2.82294200
C	2.60285200	-0.66528500	2.74061800
C	2.95870200	-0.17881100	1.49445900
H	2.77954500	1.23452700	-0.94610400
C	-3.25158200	-0.56117700	1.54090300
H	-3.19863000	-1.04570700	2.52924600
C	-4.58120300	-0.53819100	1.05867600
C	-4.87020600	0.02886800	-0.21571800
C	-5.65117800	-1.09745800	1.80089300
C	-6.15023000	0.03258500	-0.71781100
H	-4.04394900	0.45464000	-0.78836200
C	-6.93727200	-1.09756300	1.31345800
H	-5.43439300	-1.54331200	2.77464500
C	-7.16951300	-0.53105300	0.05600800
H	-6.37518700	0.46520600	-1.69180700
H	-7.76434900	-1.52925800	1.87818400
Cl	-8.78599100	-0.53686400	-0.57178100
Br	-2.75023100	3.74179200	0.24148800
Br	-0.05025800	6.15839500	-0.48080500
Br	2.90672700	4.09720500	-0.97215100
Br	0.93863900	-2.89369100	-4.38954600
Br	-1.97289800	-2.52424700	-2.18023200
Br	3.30886100	-0.41340300	-3.20667100
Br	4.63972400	-0.24480200	0.69960700
Br	3.67512000	-1.53681300	3.97874800
Br	0.09263400	-0.75065500	4.24746700
C	1.21988800	-4.31062700	-0.37235100
H	0.64207900	-3.88048800	-1.20787400
C	2.27958900	-3.29004900	0.03586700
H	1.85063700	-2.29777500	0.22111900
H	2.80935600	-3.60604400	0.95283100
H	3.03968200	-3.18014200	-0.75475700
C	1.87346800	-5.59189100	-0.86807600
H	1.12528300	-6.34349600	-1.15900400
H	2.51514200	-5.39954600	-1.74111900
H	2.50892800	-6.03834400	-0.08359000
C	0.24032900	-4.61657800	0.76036300
H	0.75139900	-5.18981800	1.54954400
H	-0.55587600	-5.27181300	0.36078900

C	-0.39198300	-3.37840600	1.37133500
H	-0.71149100	-2.67325600	0.57406100
H	0.34317400	-2.84578100	1.99669800
O	-1.48883600	-3.66090400	2.22075000
C	-2.67987200	-3.88409800	1.52530500
H	-2.95807900	-3.00112200	0.91238200
H	-2.63478500	-4.75599800	0.84519100
H	-3.46959000	-4.06178600	2.26175500

Int-3T

Zero-point correction=	0.399045 (Hartree/Particle)
Thermal correction to Energy=	0.443756
Thermal correction to Enthalpy=	0.444700
Thermal correction to Gibbs Free Energy=	0.310890
Sum of electronic and zero-point Energies=	-25026.187277
Sum of electronic and thermal Energies=	-25026.142566
Sum of electronic and thermal Enthalpies=	-25026.141622
Sum of electronic and thermal Free Energies=	-25026.275432

Ag	0.90266900	-0.05000700	-1.12757500
N	0.43257100	-1.04865400	1.01101000
N	-0.56898200	-0.61129400	1.81886000
N	0.44215500	1.94302200	0.88908900
N	-0.88553200	1.82583300	1.13998600
N	-1.43936800	0.31425900	-1.20684900
N	-2.12823800	0.02852600	-0.07100800
B	-1.57580400	0.45401300	1.32632000
C	1.07940300	-1.98458900	1.68862000
C	0.51957600	-2.18521500	2.95297200
C	-0.53513800	-1.28575000	2.99274700
C	0.63268200	3.20705500	0.55185000
C	-0.55886300	3.94486100	0.55870100
C	-1.50384100	3.00538900	0.93107900
C	-2.16950700	-0.14834300	-2.21090800
C	-3.35224300	-0.74713900	-1.75897800
C	-3.27721700	-0.60724200	-0.38311800
H	-2.48403900	0.53190000	2.09660700
C	2.72969600	0.21680600	-2.12030100
H	2.83926700	0.10316900	-3.21239200
C	3.96633300	0.49822800	-1.50226500
C	4.03152900	0.68862900	-0.09592600
C	5.16951300	0.60285100	-2.25477500
C	5.22261600	0.97008200	0.53355900

H	3.10127500	0.61133800	0.47746900
C	6.37196600	0.88078200	-1.63827500
H	5.13011800	0.45585500	-3.33531900
C	6.38181100	1.06228600	-0.24878700
H	5.27635400	1.11829700	1.60932500
H	7.29853500	0.96263300	-2.20615300
Cl	7.88627900	1.41236400	0.53114300
Br	2.33620300	3.84671400	0.13039400
Br	-0.81930800	5.73836300	0.16875700
Br	-3.33982100	3.24938900	1.10046100
Br	1.05056000	-3.37861600	4.27193000
Br	2.54538500	-2.87234800	0.95720800
Br	-1.71295600	-1.01104000	4.40081300
Br	-4.48575900	-1.21073600	0.89126000
Br	-4.68151000	-1.57643500	-2.75412000
Br	-1.61178400	0.03857300	-3.98098500
C	2.39539500	-3.16875000	-2.97926700
H	2.40393800	-2.48415400	-2.11431700
C	1.06548900	-2.97985200	-3.70605500
H	0.96004000	-1.91790700	-3.99003800
H	1.08505900	-3.55144900	-4.65489900
C	2.56844700	-4.58492400	-2.44505000
H	3.56658600	-4.72092600	-2.00182200
H	1.83010900	-4.82707200	-1.66679300
H	2.46118100	-5.32795900	-3.25340700
C	3.55072100	-2.80875000	-3.90051100
H	3.42118000	-1.81633100	-4.35366400
H	4.51181400	-2.80910000	-3.36053200
H	3.63508700	-3.53310100	-4.72238400
C	-0.16121500	-3.41144500	-2.93868900
H	-1.07701800	-3.22194200	-3.53485800
H	-0.13051600	-4.49918200	-2.74379500
O	-0.23685300	-2.71376300	-1.71178800
C	-1.15363300	-3.31155500	-0.82984400
H	-1.22839400	-2.68187300	0.06124100
H	-2.15531100	-3.41470800	-1.28702000
H	-0.80830500	-4.31488100	-0.52630800

TS-2P

Zero-point correction= 0.396447 (Hartree/Particle)
Thermal correction to Energy= 0.442276
Thermal correction to Enthalpy= 0.443220
Thermal correction to Gibbs Free Energy= 0.306948

Sum of electronic and zero-point Energies= -25026.174950
Sum of electronic and thermal Energies= -25026.129122
Sum of electronic and thermal Enthalpies= -25026.128177
Sum of electronic and thermal Free Energies= -25026.264449

Ag	1.58736500	0.16212500	-0.76244700
N	-0.03971200	-0.32752400	1.24735700
N	-1.28671400	0.20498300	1.23133800
N	0.49689000	2.15357200	-0.35428300
N	-0.84480500	2.27075600	-0.17527500
N	-0.62942000	-0.07689400	-1.97129800
N	-1.76000700	0.13658800	-1.25385000
B	-1.76974100	1.03938000	0.01028700
C	0.07219000	-0.97408300	2.39336800
C	-1.10309700	-0.90225600	3.15109600
C	-1.94101600	-0.12828300	2.36741000
C	0.99169300	3.37881700	-0.38346900
C	-0.01291600	4.33871700	-0.22167600
C	-1.16470500	3.58368700	-0.08847500
C	-0.93452000	-0.97509100	-2.89182000
C	-2.27181800	-1.37981000	-2.79880400
C	-2.75791200	-0.64013900	-1.73494500
H	-2.88152000	1.41161700	0.21694500
C	3.21077600	-1.16694600	-1.26290300
H	3.22172500	-1.49721400	-2.31027700
C	4.55887000	-1.08956800	-0.69462100
C	4.75955000	-0.62803600	0.61801200
C	5.68459400	-1.45625900	-1.45024100
C	6.03072300	-0.53844100	1.16235500
H	3.89239000	-0.32867100	1.20956400
C	6.96753900	-1.34833500	-0.93118700
H	5.54666500	-1.82457600	-2.46709700
C	7.12221900	-0.89673700	0.37516500
H	6.18083000	-0.17962900	2.17818700
H	7.83799000	-1.61982100	-1.52464200
Cl	8.73247500	-0.76914800	1.04818900
Br	2.82145400	3.65640900	-0.60254700
Br	0.15678400	6.18638800	-0.18502100
Br	-2.88438300	4.23032200	0.19269100
Br	-1.47628400	-1.70354800	4.78359600
Br	1.66049900	-1.84433300	2.85773700
Br	-3.67547100	0.39997600	2.77621600
Br	-4.47575500	-0.69043500	-1.02770700
Br	-3.17192000	-2.63817800	-3.82487200

Br	0.34915800	-1.58091100	-4.10209000
C	-1.22626000	-4.16765700	1.28589800
H	-0.73495100	-3.47974600	1.99684700
C	-2.40090200	-3.42345800	0.66281100
H	-2.09041800	-2.47078300	0.20752700
H	-2.88664200	-4.02445200	-0.12387300
H	-3.16265000	-3.18998600	1.42108800
C	-1.70332500	-5.38484700	2.06148700
H	-0.86415200	-5.93467100	2.51029500
H	-2.38541200	-5.09363400	2.87231500
H	-2.24566300	-6.07975600	1.40233700
C	-0.17555600	-4.57541700	0.24952700
H	-0.55601200	-5.40500400	-0.36866200
H	0.71665400	-4.95731700	0.77265300
C	0.22740400	-3.42671600	-0.65072300
H	0.37527800	-2.49053600	-0.08515700
H	-0.52756200	-3.23342300	-1.42237400
O	1.43647400	-3.71954100	-1.38427000
C	2.56833800	-3.40899200	-0.76064000
H	2.59558400	-2.10170000	-0.68778600
H	2.60259300	-3.54422700	0.33474900
H	3.45621800	-3.76184300	-1.28783600

TS-2S

Zero-point correction= 0.394176 (Hartree/Particle)
Thermal correction to Energy= 0.440530
Thermal correction to Enthalpy= 0.441474
Thermal correction to Gibbs Free Energy= 0.301402
Sum of electronic and zero-point Energies= -25026.175220
Sum of electronic and thermal Energies= -25026.128866
Sum of electronic and thermal Enthalpies= -25026.127922
Sum of electronic and thermal Free Energies= -25026.267994

Ag	0.99906700	0.31560700	-0.62364500
N	-0.40363800	-0.66116600	1.37802400
N	-1.72340800	-0.36340700	1.31560600
N	-0.79697700	1.71944000	-0.55350900
N	-2.08259600	1.27869500	-0.55022000
N	-0.99459000	-0.89933800	-2.01171000
N	-1.89206500	-1.20173200	-1.04353000
B	-2.42747300	-0.14582200	-0.05176000
C	-0.07897600	-0.61099700	2.65558000
C	-1.17280900	-0.29003700	3.46901000

C	-2.20275200	-0.13039700	2.55670100
C	-0.82386700	2.98677300	-0.93858100
C	-2.13453800	3.41075700	-1.19220300
C	-2.89696100	2.28659400	-0.93735000
C	-0.73936500	-2.03707200	-2.64146300
C	-1.45566900	-3.11265800	-2.09643700
C	-2.17680600	-2.52415500	-1.06735100
H	-3.60882000	-0.25209100	0.08903800
C	3.01562500	-0.24998800	-1.03797000
H	3.18395000	-0.91443900	-1.89648400
C	4.14783100	0.63775600	-0.82540400
C	4.14046600	1.58194000	0.22074300
C	5.26554000	0.60290900	-1.68261700
C	5.21035400	2.44305800	0.41737200
H	3.27302000	1.62426500	0.88417900
C	6.32423500	1.48775900	-1.52496900
H	5.28193000	-0.11892500	-2.50057200
C	6.28571300	2.38820800	-0.46691000
H	5.20444400	3.16480500	1.23219500
H	7.17294900	1.46971000	-2.20556900
Cl	7.62652800	3.48432400	-0.24061500
Br	0.75035800	3.96760700	-1.09746400
Br	-2.71991900	5.09340500	-1.72417900
Br	-4.74347000	2.13232400	-1.09858300
Br	-1.24634400	-0.11244400	5.31653400
Br	1.67752000	-0.91708200	3.22712500
Br	-3.96380100	0.34982000	2.90631900
Br	-3.33432600	-3.32986400	0.14119500
Br	-1.43258900	-4.89695800	-2.59446400
Br	0.47652700	-2.08656500	-4.05379600
O	3.02262700	-3.25767600	-0.67660500
C	3.51264100	-2.37398500	0.21647900
H	3.10048300	-1.21535700	-0.10147800
H	2.97733800	-2.38547800	1.18620100
C	1.62168800	-3.48360900	-0.55762300
H	1.35004700	-4.20993500	-1.33184300
H	1.38045300	-3.89481700	0.43598400
H	1.06212700	-2.54230400	-0.70807800
C	5.00440500	-2.32598000	0.30542400
H	5.41337400	-1.86146200	-0.60734700
H	5.37769900	-3.36606600	0.30923400
C	5.48224200	-1.59054500	1.55507800
H	4.85633400	-0.68559100	1.67689200
C	6.92662900	-1.14200400	1.38745000

H	7.04583500	-0.45523100	0.53915100
H	7.29063600	-0.62748200	2.29000100
H	7.58015600	-2.01221600	1.21549600
C	5.32973600	-2.45916600	2.79555300
H	4.31611400	-2.86324600	2.91677200
H	6.01580900	-3.31981100	2.74179700
H	5.57762200	-1.89774100	3.70816800

TS-2T

Zero-point correction= 0.395614 (Hartree/Particle)
Thermal correction to Energy= 0.440357
Thermal correction to Enthalpy= 0.441301
Thermal correction to Gibbs Free Energy= 0.309300
Sum of electronic and zero-point Energies= -25026.180517
Sum of electronic and thermal Energies= -25026.135774
Sum of electronic and thermal Enthalpies= -25026.134830
Sum of electronic and thermal Free Energies= -25026.266832

Ag	1.03388600	-0.52466600	-0.11379500
N	-0.78283700	-0.79043600	1.35222000
N	-1.76750100	0.13839000	1.44210400
N	0.44786900	1.80307400	0.59593700
N	-0.82022400	2.19245000	0.30467500
N	-0.78341000	0.44669000	-1.82375300
N	-1.90764900	0.40749200	-1.06256500
B	-1.97591700	1.16592700	0.29783300
C	-0.81560700	-1.48675300	2.47663600
C	-1.82668900	-1.03465000	3.33112700
C	-2.40487100	0.00635000	2.62530300
C	1.23041700	2.82118800	0.28341800
C	0.49867200	3.90155100	-0.22547000
C	-0.80600000	3.44366000	-0.20232100
C	-1.01749700	-0.34639600	-2.85652100
C	-2.28997300	-0.92693300	-2.79711200
C	-2.81590800	-0.41569200	-1.62454800
H	-3.03164900	1.70898600	0.43798500
C	2.88988500	-1.19764700	-0.98927700
H	2.98862400	-1.03267500	-2.07125700
C	4.13929900	-0.94398600	-0.27707300
C	4.23028400	-1.13924900	1.11354100
C	5.25999300	-0.42495100	-0.94748400
C	5.38496500	-0.82592400	1.81159700
H	3.35858000	-1.51544500	1.65123800

C	6.41258400	-0.07038800	-0.25872700
H	5.21294700	-0.27701400	-2.02668800
C	6.46182000	-0.28239000	1.11415000
H	5.44651500	-0.97111700	2.88798700
H	7.26899200	0.35299800	-0.77904700
Cl	7.91487300	0.14504200	1.99175500
Br	3.07852300	2.70387000	0.51573800
Br	1.12415500	5.55838600	-0.78500500
Br	-2.33091000	4.33824900	-0.77479500
Br	-2.29148800	-1.68774700	5.00568700
Br	0.38496800	-2.88134300	2.77942900
Br	-3.83114200	1.06740600	3.16448900
Br	-4.46614500	-0.78686900	-0.85147600
Br	-3.08406900	-2.12171400	-3.97721000
Br	0.26532800	-0.57410200	-4.19483200
C	2.88882000	-3.56101700	-1.44397900
H	2.55592000	-2.38134500	-0.91716300
C	1.82867100	-3.70646300	-2.51565100
H	1.80417100	-2.79054600	-3.12680100
H	2.16923400	-4.50653800	-3.19471700
C	2.70255900	-4.32688900	-0.16258200
H	3.45819100	-4.03579100	0.58007600
H	1.70366500	-4.17437600	0.26580500
H	2.83659900	-5.40192200	-0.36335300
C	4.28679500	-3.60009100	-1.97825500
H	4.39760500	-3.01099800	-2.89816800
H	5.02429900	-3.25732800	-1.24096600
H	4.53170600	-4.64652900	-2.21962600
C	0.43079300	-4.05552400	-2.06112300
H	-0.21954700	-4.17876500	-2.94754900
H	0.42728500	-5.02785700	-1.53155700
O	-0.08122300	-3.05120100	-1.21998500
C	-1.33077400	-3.41385600	-0.69039500
H	-1.68595900	-2.58013100	-0.07505900
H	-2.06586400	-3.61172900	-1.49111700
H	-1.25327600	-4.32036500	-0.06346400

107-P

Zero-point correction= 0.403156 (Hartree/Particle)
Thermal correction to Energy= 0.447848
Thermal correction to Enthalpy= 0.448792
Thermal correction to Gibbs Free Energy= 0.315522
Sum of electronic and zero-point Energies= -25026.255064

Sum of electronic and thermal Energies= -25026.210372
Sum of electronic and thermal Enthalpies= -25026.209428
Sum of electronic and thermal Free Energies= -25026.342698

Ag	1.11609700	0.97626600	-0.59187500
N	0.25091700	-0.22685500	1.28331200
N	-1.09779300	-0.37831400	1.32805900
N	-0.81904500	2.30885500	-0.20461800
N	-1.97684200	1.61619800	-0.00723200
N	-0.50427900	-0.22282500	-1.88773300
N	-1.57210400	-0.63510200	-1.15768100
B	-2.01458300	0.07608400	0.15148200
C	0.72905300	-0.76386500	2.39414400
C	-0.29154700	-1.28447100	3.19082500
C	-1.44123400	-1.01150900	2.46805300
C	-1.14779100	3.58579600	-0.28193100
C	-2.53045400	3.77205500	-0.12879900
C	-3.01344800	2.49109700	0.04236200
C	-0.36080300	-1.08882400	-2.87516900
C	-1.33150200	-2.09962800	-2.81604200
C	-2.07621900	-1.76735400	-1.70035500
H	-3.13232600	-0.24834900	0.40176600
C	3.97579000	-0.29973800	-1.53020900
H	3.94314700	-0.19520400	-2.62533300
C	5.03468000	0.56127000	-0.91582800
C	4.75899300	1.31040500	0.22792800
C	6.33065400	0.59666100	-1.44126800
C	5.73510000	2.09557800	0.84209600
H	3.74455900	1.29634000	0.63618200
C	7.32230200	1.36628800	-0.84887100
H	6.56610400	0.01814300	-2.33707500
C	7.01243800	2.10750600	0.28712400
H	5.50690900	2.68060100	1.72868100
H	8.32598200	1.39625200	-1.26297900
Cl	8.25774000	3.08507000	1.04081300
Br	0.13852800	4.90471800	-0.55790800
Br	-3.47808900	5.36699000	-0.15274000
Br	-4.78822300	2.00385700	0.31647900
Br	-0.14683600	-2.18380400	4.80407200
Br	2.56412100	-0.78649600	2.72158200
Br	-3.18394300	-1.41807600	2.95880700
Br	-3.51318400	-2.70779900	-0.99508600
Br	-1.53109400	-3.57238700	-3.92410800
Br	1.01248900	-0.91412000	-4.11920200

C	1.14134200	-4.10838500	1.10105500
H	1.31433200	-3.28806200	1.82208100
C	-0.28130900	-3.97150900	0.57619900
H	-0.47842200	-2.95953000	0.18431000
H	-0.47147000	-4.68753700	-0.23753400
H	-1.01973000	-4.16387600	1.36800100
C	1.31564300	-5.42791900	1.83250600
H	2.33850000	-5.54775800	2.22428200
H	0.62396200	-5.51083000	2.68146500
H	1.11872900	-6.27722000	1.15860600
C	2.19458100	-3.97165600	0.00268100
H	2.19083700	-4.87076300	-0.63503000
H	3.19369000	-3.92842400	0.47532300
C	2.02151500	-2.75456900	-0.89064500
H	1.84412200	-1.83590200	-0.28926500
H	1.14584400	-2.88148900	-1.54332700
O	3.13699700	-2.57007800	-1.74485700
C	4.15559700	-1.77679300	-1.18579100
H	2.98879800	0.03022200	-1.14339800
H	4.20877500	-1.89714400	-0.08624000
H	5.11826100	-2.12717400	-1.59032200

107-T

Zero-point correction=	0.402249 (Hartree/Particle)
Thermal correction to Energy=	0.446947
Thermal correction to Enthalpy=	0.447891
Thermal correction to Gibbs Free Energy=	0.315004
Sum of electronic and zero-point Energies=	-25026.267065
Sum of electronic and thermal Energies=	-25026.222367
Sum of electronic and thermal Enthalpies=	-25026.221423
Sum of electronic and thermal Free Energies=	-25026.354310

Ag	0.00834200	1.50412700	0.37095200
N	0.34311600	-0.78483900	1.20670400
N	-0.57261300	-1.66670200	0.72250900
N	-2.11329800	0.78317400	-0.04412600
N	-2.31315700	-0.41818000	-0.64237500
N	0.18288100	0.25196900	-1.99833400
N	-0.03993800	-1.03841300	-1.63845900
B	-1.18919900	-1.47981500	-0.69553800
C	0.74150000	-1.27089100	2.37313900
C	0.09899000	-2.47555100	2.68051400
C	-0.73354100	-2.68324200	1.59535800

C	-3.25590100	1.44589400	-0.11274800
C	-4.24184700	0.68992400	-0.75486100
C	-3.59283900	-0.49232500	-1.06982900
C	1.32986500	0.24285100	-2.66512100
C	1.89914300	-1.03344600	-2.72813700
C	0.97863400	-1.81910100	-2.06193800
H	-1.64605200	-2.51916900	-1.06120900
C	2.92636600	1.82913400	0.34056000
H	2.60252700	1.46265600	-0.64467100
C	1.96933600	2.90444400	0.79306500
C	1.80323400	3.22178000	2.15822200
C	1.22986800	3.66359600	-0.14302500
C	0.97241100	4.25351600	2.57005100
H	2.34714600	2.64512000	2.90518700
C	0.38663300	4.70498400	0.26153800
H	1.36488000	3.47541600	-1.20966800
C	0.27279700	4.98667800	1.61288000
H	0.86022200	4.48601200	3.62694500
H	-0.16935500	5.28075700	-0.47562200
Cl	-0.79445200	6.27195900	2.13334000
Br	-3.39384500	3.16163400	0.59825000
Br	-6.00410000	1.15782900	-1.09727700
Br	-4.32011600	-1.97420700	-1.92042500
Br	0.31320500	-3.54675800	4.18112700
Br	2.03452400	-0.41327100	3.40997000
Br	-1.91145200	-4.09473200	1.32876400
Br	1.04755700	-3.65584600	-1.77697800
Br	3.51588800	-1.54700600	-3.47642400
Br	2.01106000	1.79538500	-3.45528700
C	4.42190600	2.19582600	0.21852000
H	2.84425100	0.96589900	1.01440400
C	5.13742000	1.03797300	-0.50538200
H	4.74890400	0.96850800	-1.53669800
H	6.20396100	1.30275900	-0.59179300
C	5.03663400	2.44272200	1.59376900
H	4.59578200	3.32890600	2.07345300
H	4.89249300	1.59326300	2.27735600
H	6.11847500	2.62424200	1.50917000
C	4.59946400	3.45110100	-0.63151600
H	4.16223600	3.32345000	-1.63327300
H	4.12799400	4.32783500	-0.16390100
H	5.66671000	3.68180200	-0.76346200
C	5.06297700	-0.34965100	0.10212900
H	5.79845200	-1.00431200	-0.40578200

H	5.34301900	-0.34045900	1.17489200
O	3.77226000	-0.89064700	-0.04770400
C	3.69023100	-2.19781300	0.43939900
H	2.64310800	-2.52141400	0.37446500
H	4.30773800	-2.89440000	-0.15718000
H	4.01517900	-2.26534000	1.49402600

107-S

Zero-point correction=	0.401579 (Hartree/Particle)
Thermal correction to Energy=	0.447754
Thermal correction to Enthalpy=	0.448698
Thermal correction to Gibbs Free Energy=	0.310277
Sum of electronic and zero-point Energies=	-25026.259939
Sum of electronic and thermal Energies=	-25026.213765
Sum of electronic and thermal Enthalpies=	-25026.212821
Sum of electronic and thermal Free Energies=	-25026.351241

Ag	1.01183600	0.67969700	0.01517300
N	-0.33151800	-0.97493400	1.33425100
N	-1.65497300	-0.91787500	1.02665700
N	-1.01498400	1.78387200	-0.09076800
N	-2.10960600	1.07713100	-0.47304600
N	-0.05192300	-0.55198100	-1.85516800
N	-1.21547700	-1.11686300	-1.44083700
B	-2.13997900	-0.46751200	-0.38004700
C	-0.26653800	-1.40067900	2.58726100
C	-1.53616400	-1.62871500	3.12909200
C	-2.39026400	-1.30160500	2.09135400
C	-1.31853800	3.06529900	-0.22156300
C	-2.62576400	3.23189000	-0.68922100
C	-3.08961500	1.93591100	-0.83380300
C	0.49748900	-1.41865700	-2.69396900
C	-0.28177000	-2.57164700	-2.83930500
C	-1.36219800	-2.33284400	-2.00879100
H	-3.25788200	-0.84066900	-0.55387400
C	3.55090800	-0.94315300	0.29180900
H	3.32232900	-1.31996900	-0.71810100
C	3.36523500	0.55081500	0.35278500
C	3.29253800	1.22108100	1.59626800
C	3.44610700	1.34706100	-0.81160200
C	3.36043100	2.60701700	1.67843400
H	3.22303100	0.63310100	2.51188100
C	3.51281000	2.73870800	-0.74024100

H	3.51349900	0.85807200	-1.78355900
C	3.48953000	3.34799700	0.50696700
H	3.31668000	3.10658200	2.64391400
H	3.58590300	3.33541800	-1.64697100
Cl	3.57919500	5.09193900	0.60759100
Br	-0.07294800	4.38494100	0.19380300
Br	-3.53028500	4.81620700	-1.02998300
Br	-4.77249500	1.41633500	-1.42293600
Br	-1.97254900	-2.23980300	4.82594300
Br	1.36573500	-1.66869200	3.45652800
Br	-4.24759900	-1.34722000	2.10332100
Br	-2.79237900	-3.46643800	-1.65917500
Br	0.03923600	-4.06142300	-3.89737200
Br	2.11131400	-1.04716900	-3.55699200
O	5.08379900	-2.79667300	0.54042400
C	4.98099000	-1.39218400	0.64062600
H	2.84899900	-1.42636600	0.98669500
H	5.18793000	-1.09495200	1.69140300
C	4.36320000	-3.51371100	1.50699200
H	4.73580600	-4.54411500	1.49502400
H	4.51881800	-3.09685300	2.51854600
H	3.28009200	-3.54233100	1.30306800
C	6.03345400	-0.79665000	-0.28857400
H	5.58946800	-0.64583200	-1.28810600
H	6.82250800	-1.55458500	-0.41843800
C	6.68786100	0.49975300	0.20124700
H	5.90515000	1.18438100	0.57231000
C	7.40637300	1.18731000	-0.95032000
H	6.70894500	1.46551500	-1.75369700
H	7.91894500	2.10061600	-0.61816900
H	8.16560600	0.51882500	-1.38529800
C	7.65878500	0.22780300	1.34354300
H	7.18189100	-0.29434800	2.18491700
H	8.49485400	-0.39893000	0.99710400
H	8.08347900	1.16261100	1.73468400

Cartesian coordinates of computed structures with single point energy calculations at SMD// ωB97XD/6-31+G(d,p)-SDD(Ag) level.

AgTp^{Br3}-THF

Zero-point correction=	0.229707 (Hartree/Particle)
Thermal correction to Energy=	0.262895

Thermal correction to Enthalpy= 0.263839
Thermal correction to Gibbs Free Energy= 0.152992
Sum of electronic and zero-point Energies= -24218.899781
Sum of electronic and thermal Energies= -24218.866593
Sum of electronic and thermal Enthalpies= -24218.865649
Sum of electronic and thermal Free Energies= -24218.976496

Ag	-1.59688600	0.56942900	-1.15095000
N	-1.22419300	0.14263900	1.20592900
N	0.00213400	-0.26370900	1.61695700
N	-0.12980500	-1.32843300	-1.24658600
N	0.97150800	-1.36612400	-0.45549600
N	0.48769500	1.66341000	-0.81128900
N	1.41761500	1.10068200	0.00181500
B	1.21098900	-0.29745300	0.64431600
C	-2.03358000	0.01647600	2.24151200
C	-1.36056100	-0.47984100	3.36406700
C	-0.06239600	-0.64615200	2.91364300
C	-0.04944300	-2.36493400	-2.06284400
C	1.10821500	-3.11581200	-1.82738200
C	1.72753200	-2.43791500	-0.79060100
C	0.95235000	2.85061700	-1.15934500
C	2.20415900	3.09986700	-0.58647700
C	2.45724900	1.95428800	0.14820900
H	2.18633500	-0.58719900	1.25706300
Br	-1.38177800	-2.67558300	-3.32865200
Br	1.67482700	-4.65660800	-2.69356000
Br	3.32949500	-2.88308600	0.04027700
Br	-2.04467900	-0.82923500	5.05416500
Br	-3.83699100	0.46827200	2.09291800
Br	1.38623500	-1.29488600	3.88006000
Br	3.95905800	1.59951300	1.18412500
Br	3.27053000	4.60930300	-0.75817700
Br	-0.04306000	3.96200700	-2.27567900
C	-4.61814900	-0.18386400	-1.98768900
O	-3.63942800	0.87546600	-2.06965200
C	-4.28333600	2.15941100	-1.94313800
C	-5.75274800	1.88225400	-2.17892900
C	-5.90775700	0.49315300	-1.56668200
H	-4.70347500	-0.64621300	-2.98174200
H	-4.25608700	-0.94064000	-1.27895900
H	-4.10021700	2.55604600	-0.93138400
H	-3.82568000	2.83588800	-2.67437400
H	-6.39766100	2.63733900	-1.71677800

H	-5.96913100	1.85285000	-3.25580700
H	-5.96853900	0.56287000	-0.47088800
H	-6.79254900	-0.04455000	-1.92388500

THF

Zero-point correction= 0.117649 (Hartree/Particle)
Thermal correction to Energy= 0.122519
Thermal correction to Enthalpy= 0.123464
Thermal correction to Gibbs Free Energy= 0.089166
Sum of electronic and zero-point Energies= -232.285547
Sum of electronic and thermal Energies= -232.280677
Sum of electronic and thermal Enthalpies= -232.279733
Sum of electronic and thermal Free Energies= -232.314031

C	-1.15693500	-0.41992300	-0.13331600
O	-0.00285600	-1.24211000	-0.00511400
C	1.15300600	-0.42589400	0.13978200
C	0.72715000	0.98201700	-0.23995100
C	-0.72010800	0.98790100	0.23696000
H	-1.52268300	-0.45928800	-1.17424500
H	-1.95098600	-0.81616400	0.51476200
H	1.50353600	-0.46286700	1.18635300
H	1.95400600	-0.82860300	-0.49529500
H	1.35063500	1.75537800	0.22273900
H	0.76854300	1.11380000	-1.33049300
H	-0.75996200	1.12776400	1.32652600
H	-1.33891400	1.76225800	-0.23028600

AgTp^{Br3}

Zero-point correction= 0.111011 (Hartree/Particle)
Thermal correction to Energy= 0.137449
Thermal correction to Enthalpy= 0.138393
Thermal correction to Gibbs Free Energy= 0.044693
Sum of electronic and zero-point Energies= -23986.547467
Sum of electronic and thermal Energies= -23986.521030
Sum of electronic and thermal Enthalpies= -23986.520085
Sum of electronic and thermal Free Energies= -23986.613786

Ag	0.02336100	-2.02193500	1.31818900
N	1.12572700	-1.64473400	-0.68036400
N	0.91663700	-0.44055600	-1.27449500
N	-1.82501500	-1.21424200	0.07329300

N	-1.62888800	-0.45153800	-1.04370600
N	0.11592400	0.21455800	1.40179300
N	-0.18270600	1.02622700	0.36697500
B	-0.34804800	0.42127700	-1.02904500
C	2.36867900	-2.02153000	-0.98171900
C	2.97871100	-1.12785400	-1.87477100
C	2.03469800	-0.11203100	-1.97604400
C	-3.12802900	-1.37138800	0.21534200
C	-3.83265900	-0.56300400	-0.67387500
C	-2.82635800	0.06431100	-1.37643900
C	0.77101900	0.96027700	2.25846700
C	0.71952600	2.30789900	1.95854300
C	0.16522800	2.28877600	0.68628500
H	-0.30822700	1.28341900	-1.84393400
Br	-0.11164200	3.61964700	-0.55151700
Br	1.29004300	3.73218100	3.01562100
Br	1.71981700	0.12638600	3.64503800
Br	2.26097700	1.60890100	-2.66147500
Br	4.77812500	-1.03203400	-2.34637800
Br	3.03594600	-3.48907200	-0.08935000
Br	-3.12204800	1.31785400	-2.71597400
Br	-5.66796600	-0.34161100	-0.89241900
Br	-3.73199900	-2.49481300	1.55974300

2a-1

Zero-point correction= 0.105583 (Hartree/Particle)
Thermal correction to Energy= 0.113996
Thermal correction to Enthalpy= 0.114940
Thermal correction to Gibbs Free Energy= 0.071312
Sum of electronic and zero-point Energies= -839.158344
Sum of electronic and thermal Energies= -839.149931
Sum of electronic and thermal Enthalpies= -839.148987
Sum of electronic and thermal Free Energies= -839.192615

N	-3.35468800	-0.17482500	0.13233000
C	-2.50887900	0.74144900	-0.36236800
N	-4.05632400	-0.98532800	0.49100000
H	-2.84869500	1.74977400	-0.10718500
C	-1.06335200	0.43454700	-0.22273400
C	-0.17872400	1.49198200	0.00971400
C	-0.56213900	-0.86560900	-0.34662600
C	1.18630400	1.25908800	0.11652000
H	-0.56133600	2.50773200	0.09837200

C	0.79919400	-1.11045800	-0.22733600
H	-1.23546200	-1.69815100	-0.55059700
C	1.66087800	-0.04245400	0.00088100
H	1.87567200	2.08156600	0.29383600
H	1.18896800	-2.12164500	-0.32297900
Cl	3.37989700	-0.34408600	0.14217200

Int-1

Zero-point correction=	0.217986 (Hartree/Particle)
Thermal correction to Energy=	0.255556
Thermal correction to Enthalpy=	0.256500
Thermal correction to Gibbs Free Energy=	0.133290
Sum of electronic and zero-point Energies=	-24825.774533
Sum of electronic and thermal Energies=	-24825.736963
Sum of electronic and thermal Enthalpies=	-24825.736019
Sum of electronic and thermal Free Energies=	-24825.859229

Ag	-1.10594700	-1.21054800	-0.35456600
N	-0.93803300	1.17943700	-0.34080700
N	0.28512200	1.76250100	-0.24449900
N	0.48455500	-0.90709600	1.43266200
N	1.35932600	0.12908900	1.38157900
N	0.93365600	-0.83029700	-1.64313200
N	1.88385600	-0.03669100	-1.08795400
B	1.55936900	0.94126600	0.07129700
C	-1.80431800	2.14807100	-0.58657700
C	-1.17167300	3.39514000	-0.65501500
C	0.16020700	3.09666700	-0.43083500
C	0.54459300	-1.39153900	2.66185300
C	1.46323600	-0.68816300	3.44782500
C	1.95604500	0.27542400	2.58522100
C	1.54958300	-1.58212500	-2.53819100
C	2.91785700	-1.29676600	-2.60337900
C	3.08250200	-0.30286000	-1.65541700
H	2.47640600	1.68391400	0.21420800
Br	-0.50900300	-2.85008700	3.15058400
Br	1.93144600	-0.97660700	5.22070600
Br	3.22439200	1.57805700	2.96772900
Br	-1.94117200	5.05485400	-0.97047900
Br	-3.61769600	1.77525800	-0.79434800
Br	1.58049700	4.29652300	-0.37355900
Br	4.66867000	0.53961600	-1.17950700
Br	4.20248400	-2.06486000	-3.70145700

Br	0.61257300	-2.84826000	-3.53789000
N	-2.39368700	-3.82549900	0.05068300
C	-2.70539400	-2.80355300	-0.76057700
N	-2.11180600	-4.63924400	0.78294900
H	-2.45569600	-3.05073900	-1.79685100
C	-4.00357200	-2.13478600	-0.49464700
C	-4.73928400	-1.65498800	-1.58243800
C	-4.49238400	-1.94522200	0.80231000
C	-5.94464900	-0.99464500	-1.38246100
H	-4.36018000	-1.79140100	-2.59420500
C	-5.70308400	-1.29886600	1.01168500
H	-3.92036400	-2.29282600	1.66257700
C	-6.41641300	-0.82719600	-0.08554000
H	-6.51600000	-0.61790600	-2.22789800
H	-6.08362000	-1.15203200	2.02025000
Cl	-7.93912000	-0.00234100	0.17320400

TS-1

Zero-point correction= 0.214502 (Hartree/Particle)
Thermal correction to Energy= 0.251121
Thermal correction to Enthalpy= 0.252065
Thermal correction to Gibbs Free Energy= 0.134662
Sum of electronic and zero-point Energies= -24825.750493
Sum of electronic and thermal Energies= -24825.713873
Sum of electronic and thermal Enthalpies= -24825.712929
Sum of electronic and thermal Free Energies= -24825.830332

Ag	-1.11673200	0.38839000	1.04105600
N	-0.86629000	-0.89157100	-1.09403000
N	0.44085100	-1.24056300	-1.34680900
N	0.36578100	1.73508200	-0.19323900
N	1.21074800	1.17685300	-1.09895300
N	1.04422900	-0.77762700	1.42845900
N	2.01525500	-0.61110800	0.48234400
B	1.62011600	-0.31891600	-0.98188100
C	-1.58733500	-1.95377900	-1.43506600
C	-0.78401600	-3.02079600	-1.90260300
C	0.51116900	-2.51473800	-1.80781700
C	0.27758700	3.01743500	-0.49350900
C	1.07837200	3.33750700	-1.59974700
C	1.65178900	2.12846600	-1.95027100
C	1.67156300	-0.94131000	2.58401400
C	3.06670900	-0.89406000	2.42801000

C	3.23626000	-0.67301900	1.06585300
H	2.53792200	-0.52630900	-1.71186100
Br	-0.79269000	4.15164900	0.53499600
Br	1.31089300	4.99962600	-2.39937800
Br	2.83965700	1.81736200	-3.35673300
Br	-1.29315400	-4.70069600	-2.49776700
Br	-3.45770400	-1.92406800	-1.24969100
Br	2.13253400	-3.41518500	-2.18681100
Br	4.84337500	-0.48452500	0.12259100
Br	4.39098900	-1.06369200	3.72083800
Br	0.70552600	-1.19352500	4.16269600
N	-2.73261100	2.07053400	3.07413400
C	-2.87092200	0.44445100	2.27512800
N	-2.52149400	3.16672900	3.16117900
H	-2.82880200	-0.11872000	3.22510200
C	-4.20484200	0.46221900	1.69993800
C	-5.25027500	-0.27168500	2.28114600
C	-4.45380300	1.16702300	0.49948900
C	-6.49299400	-0.35236800	1.66698000
H	-5.07661900	-0.80690900	3.21339600
C	-5.70023800	1.13144100	-0.09600300
H	-3.63650400	1.72494700	0.03909300
C	-6.69776400	0.36066000	0.48441200
H	-7.28990600	-0.94845400	2.10128000
H	-5.89081200	1.67335000	-1.02965600
Cl	-8.27130500	0.28448700	-0.27792800

Int-2

Zero-point correction= 0.214832 (Hartree/Particle)
Thermal correction to Energy= 0.253659
Thermal correction to Enthalpy= 0.254603
Thermal correction to Gibbs Free Energy= 0.128780
Sum of electronic and zero-point Energies= -24825.778740
Sum of electronic and thermal Energies= -24825.739913
Sum of electronic and thermal Enthalpies= -24825.738969
Sum of electronic and thermal Free Energies= -24825.864791

Ag	-1.37011600	-0.29019400	-0.75323000
N	-0.08846700	1.67641200	-0.78686200
N	1.24348100	1.68170200	-0.53126600
N	-0.05510800	-0.60043600	1.25736000
N	1.12583400	0.05243500	1.38556500
N	0.97398600	-0.92355400	-1.79057900

N	1.93320500	-0.75123900	-0.84596200
B	1.93107400	0.45565300	0.12321200
C	-0.39262600	2.87479300	-1.25990900
C	0.73938900	3.69800800	-1.31748500
C	1.75473500	2.88949400	-0.84288600
C	-0.51639900	-0.78324400	2.48377100
C	0.35019300	-0.25544500	3.44688600
C	1.38197400	0.27666300	2.69241900
C	1.20587500	-2.10327300	-2.33832100
C	2.31668300	-2.73916600	-1.76735700
C	2.73892100	-1.83426800	-0.80783400
H	3.04780000	0.74335900	0.42995000
Br	-2.14314300	-1.66007100	2.77564200
Br	0.17066100	-0.26610500	5.29548300
Br	2.88377200	1.18280300	3.30131900
Br	0.84658400	5.46796700	-1.87243200
Br	-2.13499600	3.29904800	-1.75915700
Br	3.54526800	3.35357400	-0.62973700
Br	4.13852000	-2.03173500	0.40448900
Br	3.01210000	-4.41400200	-2.15636300
Br	0.09590000	-2.77117000	-3.68411400
C	-3.15357800	-1.17012300	-1.34711800
H	-3.14469700	-1.99975400	-2.07341700
C	-4.46248300	-0.90918100	-0.88638400
C	-5.57926800	-1.66459300	-1.34531400
C	-4.68784000	0.12185700	0.06556300
C	-6.85786700	-1.40254900	-0.88646800
H	-5.42209900	-2.46156400	-2.07302900
C	-5.95990900	0.39534800	0.52799600
H	-3.82658300	0.68590800	0.42838100
C	-7.02611100	-0.37047500	0.04026500
H	-7.71423600	-1.97487700	-1.23266100
H	-6.13940400	1.18508200	1.25548800
Cl	-8.62700600	-0.02522300	0.61523400

Methyl isoamyl ether

Zero-point correction=	0.195118 (Hartree/Particle)
Thermal correction to Energy=	0.203908
Thermal correction to Enthalpy=	0.204852
Thermal correction to Gibbs Free Energy=	0.162190
Sum of electronic and zero-point Energies=	-312.028993
Sum of electronic and thermal Energies=	-312.020203
Sum of electronic and thermal Enthalpies=	-312.019259

Sum of electronic and thermal Free Energies= -312.061921

C	3.28562500	0.15255900	0.06325100
H	3.34668300	1.09780700	-0.50549100
H	3.38507700	0.39540100	1.13647600
H	4.12617900	-0.48681100	-0.22921000
O	2.10022600	-0.54065100	-0.20485900
C	0.96504300	0.21512100	0.14274900
H	0.99083200	0.46669700	1.22331800
H	0.97587700	1.17622800	-0.40627800
C	-0.27264400	-0.59073300	-0.18180100
H	-0.34772900	-0.71222900	-1.27585600
H	-0.13527900	-1.60046400	0.23601000
C	-1.57014700	0.01049400	0.35392200
H	-1.49008000	0.06205900	1.45453300
C	-1.80623500	1.42142500	-0.17084900
H	-2.77954700	1.80875200	0.16096900
H	-1.03932700	2.12937000	0.17093400
H	-1.80392900	1.43150500	-1.27221200
C	-2.74625500	-0.88845400	0.00007700
H	-2.60585900	-1.91084600	0.37744100
H	-3.68733700	-0.50327200	0.41623500
H	-2.86968700	-0.95146300	-1.09210300

Int-3S

Zero-point correction= 0.404869 (Hartree/Particle)
Thermal correction to Energy= 0.450618
Thermal correction to Enthalpy= 0.451563
Thermal correction to Gibbs Free Energy= 0.312741
Sum of electronic and zero-point Energies= -25028.338264
Sum of electronic and thermal Energies= -25028.292515
Sum of electronic and thermal Enthalpies= -25028.291570
Sum of electronic and thermal Free Energies= -25028.430392

Ag	0.89632800	0.43591000	-0.68006100
N	-0.15645500	-0.34885400	1.34420600
N	-1.50877000	-0.31903000	1.45372500
N	-1.07097600	1.72564000	-0.63619800
N	-2.28662500	1.16374300	-0.41586800
N	-1.04384700	-0.98176600	-1.84811800
N	-1.88890700	-1.30762800	-0.83922300
B	-2.39957600	-0.26017200	0.18040800
C	0.31881100	-0.26424300	2.57829900

C	-0.70964300	-0.18006000	3.52325400
C	-1.85882400	-0.21088800	2.75201900
C	-1.29240900	2.94216400	-1.10354800
C	-2.66234600	3.21417200	-1.19339600
C	-3.25481200	2.04821900	-0.74042600
C	-0.79441900	-2.10812900	-2.49428400
C	-1.46121000	-3.20011900	-1.92275700
C	-2.14343700	-2.63356400	-0.86059400
H	-3.53128300	-0.49421700	0.47529900
C	2.77042500	0.28889400	-1.55806800
H	2.92602600	-0.47362800	-2.33838200
C	3.95429100	1.01149800	-1.30045900
C	3.98418800	2.00510200	-0.28640400
C	5.15320000	0.73590300	-2.01140500
C	5.15160100	2.66885500	0.02734200
H	3.06060700	2.22616500	0.24992700
C	6.32550200	1.39981700	-1.71576700
H	5.13585400	-0.01755100	-2.79866600
C	6.31019800	2.34638700	-0.68693800
H	5.18502500	3.42130000	0.81141200
H	7.24862600	1.19053400	-2.25134500
Cl	7.79231600	3.14082900	-0.27247700
Br	0.12261000	4.06913600	-1.55241700
Br	-3.49005400	4.77288400	-1.76685400
Br	-5.07282700	1.70895800	-0.56559200
Br	-0.56762700	-0.05453200	5.36913100
Br	2.15404100	-0.23263200	2.91380000
Br	-3.61742900	-0.10426600	3.34299700
Br	-3.23478600	-3.48152200	0.38369700
Br	-1.44455700	-4.98090800	-2.44866200
Br	0.34557300	-2.11525700	-3.97341700
O	3.28738400	-3.02373500	-0.88937200
C	3.76877600	-2.39761100	0.27413500
H	3.23283300	-1.43701700	0.43033600
H	3.54337600	-3.02727000	1.15718100
C	1.92160600	-3.32022700	-0.78581800
H	1.61170800	-3.79204400	-1.72503000
H	1.71655200	-4.01714700	0.04734300
H	1.31659900	-2.40518900	-0.62263800
C	5.25367400	-2.15020500	0.13406900
H	5.42646700	-1.69437900	-0.85610600
H	5.79270500	-3.11289600	0.13409900
C	5.82700300	-1.23441100	1.21407700
H	5.27227400	-0.27726800	1.16801700

C	7.29684400	-0.94713300	0.93985500
H	7.45221100	-0.54326000	-0.07177300
H	7.70598700	-0.22085200	1.65662000
H	7.89293600	-1.86887400	1.02386100
C	5.65919600	-1.81698500	2.61214800
H	4.60531400	-1.96702300	2.88568900
H	6.15904500	-2.79552100	2.68550500
H	6.10592700	-1.15950900	3.37100200

Int-3P

Zero-point correction=	0.402930 (Hartree/Particle)
Thermal correction to Energy=	0.449177
Thermal correction to Enthalpy=	0.450121
Thermal correction to Gibbs Free Energy=	0.308506
Sum of electronic and zero-point Energies=	-25028.343417
Sum of electronic and thermal Energies=	-25028.297171
Sum of electronic and thermal Enthalpies=	-25028.296227
Sum of electronic and thermal Free Energies=	-25028.437841

Ag	-1.52885100	0.15469600	0.55661000
N	-0.12953300	-0.61771800	-1.22478500
N	1.11729600	-0.11633400	-1.43674900
N	-0.47061400	2.15603700	-0.19378900
N	0.86193000	2.21646600	-0.43867200
N	0.75564200	0.22689600	1.73988900
N	1.84674800	0.33456800	0.94726300
B	1.73992100	0.93587900	-0.47796500
C	-0.33754300	-1.53295200	-2.15485800
C	0.77466500	-1.67977600	-2.99081600
C	1.67775200	-0.75164100	-2.49241600
C	-0.90512400	3.40611300	-0.16926400
C	0.13526900	4.32424100	-0.38434600
C	1.24424900	3.51200200	-0.54943300
C	1.15186400	-0.39601000	2.84160600
C	2.51293500	-0.73122000	2.78269800
C	2.91269900	-0.24264300	1.55138000
H	2.82650600	1.21564800	-0.87831000
C	-3.27097100	-0.50358700	1.47667800
H	-3.21224000	-0.96455600	2.47879300
C	-4.60204900	-0.49156300	1.00473600
C	-4.90069400	0.03653500	-0.28757000
C	-5.66809200	-1.02213100	1.77443700
C	-6.18717900	0.01884800	-0.77871700

H	-4.07831500	0.44057600	-0.87787300
C	-6.96099500	-1.03867200	1.30283900
H	-5.44476200	-1.44138000	2.76523900
C	-7.20318200	-0.51689100	0.02349300
H	-6.41899100	0.41994100	-1.76501800
H	-7.78418200	-1.45358200	1.88514800
Cl	-8.82397600	-0.54427800	-0.59462800
Br	-2.70183700	3.79025800	0.15591000
Br	0.05464500	6.17663600	-0.45239200
Br	2.99396200	4.07645800	-0.86570100
Br	1.01445700	-2.87076100	-4.39323200
Br	-1.94261400	-2.47708500	-2.24623500
Br	3.38252000	-0.42144300	-3.14288300
Br	4.60680900	-0.36014500	0.78717500
Br	3.52592300	-1.65735000	4.02982000
Br	-0.02989400	-0.73856800	4.23760700
C	1.17370200	-4.32745800	-0.39025200
H	0.61173300	-3.88562000	-1.22895500
C	2.25335900	-3.33240500	0.02348000
H	1.84833400	-2.33109800	0.20669400
H	2.76709800	-3.65998400	0.94836700
H	3.02419100	-3.24254400	-0.75750200
C	1.79544700	-5.62370200	-0.88157900
H	1.03348300	-6.35931300	-1.18069600
H	2.45043300	-5.44949400	-1.74668600
H	2.41573800	-6.08729000	-0.09295800
C	0.17467000	-4.60751100	0.73261700
H	0.66892500	-5.19138500	1.52794200
H	-0.63219500	-5.24560200	0.32953200
C	-0.43347900	-3.35518300	1.33922600
H	-0.72935600	-2.64177700	0.53519000
H	0.30656400	-2.84018900	1.97123500
O	-1.54920000	-3.61370300	2.17169400
C	-2.73490600	-3.81332400	1.46303600
H	-2.98572700	-2.92368400	0.84517900
H	-2.69808400	-4.68691000	0.78373000
H	-3.53524600	-3.97529800	2.18889400

Int-3T

Ag	0.89752000	-0.10245600	-1.13916200
N	0.40363500	-1.04487600	1.01644900
N	-0.55725700	-0.53887700	1.82674600
N	0.53782100	1.94940500	0.77983900

N	-0.77951200	1.89473100	1.09954900
N	-1.41730200	0.37852700	-1.21768400
N	-2.10327400	0.12628300	-0.06704800
B	-1.52461800	0.55773800	1.32394100
C	1.00888500	-2.00262700	1.70629600
C	0.46840000	-2.14311300	2.98119800
C	-0.53630700	-1.18938300	3.01436900
C	0.77710400	3.19683100	0.41312300
C	-0.37077200	3.99547500	0.47854400
C	-1.34930800	3.10550400	0.90277500
C	-2.17052600	-0.09395600	-2.20209000
C	-3.35309900	-0.67307400	-1.72209100
C	-3.26345300	-0.49724400	-0.35069200
H	-2.41852900	0.69013600	2.09360800
C	2.73990500	0.03529700	-2.12965300
H	2.85997200	-0.10706600	-3.22069500
C	3.98629900	0.28779200	-1.50334200
C	4.03911900	0.52639200	-0.10228000
C	5.19336400	0.33529400	-2.25317600
C	5.23088300	0.80129000	0.52573900
H	3.10390100	0.50080800	0.46429800
C	6.39616100	0.60591700	-1.63820400
H	5.16293200	0.15620200	-3.32989000
C	6.40307300	0.83720600	-0.25323300
H	5.28562200	0.98046300	1.59734700
H	7.32838600	0.64631700	-2.19953400
Cl	7.91337400	1.18067200	0.52959300
Br	2.48860500	3.73341900	-0.11257700
Br	-0.55993600	5.79048700	0.08333700
Br	-3.15900600	3.45213800	1.16909800
Br	0.95558300	-3.33965400	4.31917800
Br	2.41517100	-2.98687400	0.96655500
Br	-1.67160700	-0.81555900	4.44017200
Br	-4.46061500	-1.05271100	0.95170800
Br	-4.70111100	-1.50698500	-2.68205700
Br	-1.62820700	0.05185600	-3.98300900
C	2.05076400	-3.33363400	-2.99901100
H	2.12974700	-2.63330100	-2.14954600
C	0.68045400	-3.12062500	-3.65219700
H	0.59623800	-2.06049600	-3.95739500
H	0.62335900	-3.71040800	-4.57819100
C	2.20086800	-4.74564500	-2.43699500
H	3.21818900	-4.90615800	-2.03384100
H	1.49376700	-4.94042000	-1.61496800

H	2.03166700	-5.49917900	-3.21752700
C	3.15727200	-3.04145100	-3.99144800
H	3.02312300	-2.06285200	-4.48349700
H	4.14489900	-3.05308000	-3.50424400
H	3.17927900	-3.80473800	-4.78784500
C	-0.51229700	-3.48387600	-2.80104100
H	-1.45657500	-3.26601000	-3.34935900
H	-0.51515900	-4.56824000	-2.59329300
O	-0.49234200	-2.76992500	-1.57900900
C	-1.36210500	-3.33121900	-0.63339700
H	-1.36860700	-2.69142400	0.25462500
H	-2.39341500	-3.41930300	-1.02509400
H	-1.01537000	-4.33387400	-0.33335400

TS-2P

Zero-point correction= 0.402622 (Hartree/Particle)
Thermal correction to Energy= 0.448059
Thermal correction to Enthalpy= 0.449004
Thermal correction to Gibbs Free Energy= 0.311174
Sum of electronic and zero-point Energies= -25028.333318
Sum of electronic and thermal Energies= -25028.287880
Sum of electronic and thermal Enthalpies= -25028.286936
Sum of electronic and thermal Free Energies= -25028.424766

Ag	1.58736500	0.16212500	-0.76244700
N	-0.03971200	-0.32752400	1.24735700
N	-1.28671400	0.20498300	1.23133800
N	0.49689000	2.15357200	-0.35428300
N	-0.84480500	2.27075600	-0.17527500
N	-0.62942000	-0.07689400	-1.97129800
N	-1.76000700	0.13658800	-1.25385000
B	-1.76974100	1.03938000	0.01028700
C	0.07219000	-0.97408300	2.39336800
C	-1.10309700	-0.90225600	3.15109600
C	-1.94101600	-0.12828300	2.36741000
C	0.99169300	3.37881700	-0.38346900
C	-0.01291600	4.33871700	-0.22167600
C	-1.16470500	3.58368700	-0.08847500
C	-0.93452000	-0.97509100	-2.89182000
C	-2.27181800	-1.37981000	-2.79880400
C	-2.75791200	-0.64013900	-1.73494500
H	-2.88152000	1.41161700	0.21694500
C	3.21077600	-1.16694600	-1.26290300

H	3.22172500	-1.49721400	-2.31027700
C	4.55887000	-1.08956800	-0.69462100
C	4.75955000	-0.62803600	0.61801200
C	5.68459400	-1.45625900	-1.45024100
C	6.03072300	-0.53844100	1.16235500
H	3.89239000	-0.32867100	1.20956400
C	6.96753900	-1.34833500	-0.93118700
H	5.54666500	-1.82457600	-2.46709700
C	7.12221900	-0.89673700	0.37516500
H	6.18083000	-0.17962900	2.17818700
H	7.83799000	-1.61982100	-1.52464200
Cl	8.73247500	-0.76914800	1.04818900
Br	2.82145400	3.65640900	-0.60254700
Br	0.15678400	6.18638800	-0.18502100
Br	-2.88438300	4.23032200	0.19269100
Br	-1.47628400	-1.70354800	4.78359600
Br	1.66049900	-1.84433300	2.85773700
Br	-3.67547100	0.39997600	2.77621600
Br	-4.47575500	-0.69043500	-1.02770700
Br	-3.17192000	-2.63817800	-3.82487200
Br	0.34915800	-1.58091100	-4.10209000
C	-1.22626000	-4.16765700	1.28589800
H	-0.73495100	-3.47974600	1.99684700
C	-2.40090200	-3.42345800	0.66281100
H	-2.09041800	-2.47078300	0.20752700
H	-2.88664200	-4.02445200	-0.12387300
H	-3.16265000	-3.18998600	1.42108800
C	-1.70332500	-5.38484700	2.06148700
H	-0.86415200	-5.93467100	2.51029500
H	-2.38541200	-5.09363400	2.87231500
H	-2.24566300	-6.07975600	1.40233700
C	-0.17555600	-4.57541700	0.24952700
H	-0.55601200	-5.40500400	-0.36866200
H	0.71665400	-4.95731700	0.77265300
C	0.22740400	-3.42671600	-0.65072300
H	0.37527800	-2.49053600	-0.08515700
H	-0.52756200	-3.23342300	-1.42237400
O	1.43647400	-3.71954100	-1.38427000
C	2.56833800	-3.40899200	-0.76064000
H	2.59558400	-2.10170000	-0.68778600
H	2.60259300	-3.54422700	0.33474900
H	3.45621800	-3.76184300	-1.28783600

TS-2S

Zero-point correction= 0.401065 (Hartree/Particle)
Thermal correction to Energy= 0.446065
Thermal correction to Enthalpy= 0.447010
Thermal correction to Gibbs Free Energy= 0.308755
Sum of electronic and zero-point Energies= -25028.333061
Sum of electronic and thermal Energies= -25028.288060
Sum of electronic and thermal Enthalpies= -25028.287116
Sum of electronic and thermal Free Energies= -25028.425370

Ag	0.98406500	0.31638400	-0.57712600
N	-0.40293300	-0.74330500	1.36105700
N	-1.71566900	-0.40223300	1.34705700
N	-0.79847800	1.75125600	-0.42340400
N	-2.08782200	1.32651900	-0.43679800
N	-1.04193300	-0.79002100	-2.00288700
N	-1.94027800	-1.12857400	-1.04652100
B	-2.44426400	-0.11395100	0.00838300
C	-0.04741000	-0.76226900	2.63545900
C	-1.11343400	-0.44012700	3.48465300
C	-2.15788000	-0.21031000	2.60736600
C	-0.80878300	3.01822900	-0.80697500
C	-2.10861400	3.45803200	-1.07706100
C	-2.88783900	2.34095600	-0.82803100
C	-0.80444400	-1.89860900	-2.68588900
C	-1.52861500	-2.98920400	-2.18556800
C	-2.23700300	-2.44153800	-1.12981500
H	-3.62471800	-0.21089700	0.16449400
C	2.99036800	-0.21396400	-1.05885900
H	3.14239700	-0.81974200	-1.96535800
C	4.12289200	0.67000000	-0.81996500
C	4.13655500	1.54638200	0.28321800
C	5.21979700	0.69529000	-1.70172600
C	5.20133900	2.39990600	0.51089700
H	3.28260700	1.54426000	0.96355900
C	6.27812800	1.57418700	-1.51190300
H	5.22488900	0.02681900	-2.56311300
C	6.26039400	2.40665300	-0.39818500
H	5.21168700	3.06832800	1.36902100
H	7.11335400	1.60641200	-2.20818600
Cl	7.59851700	3.49651300	-0.12981200
Br	0.77704200	3.98643800	-0.94096800
Br	-2.66544000	5.14055400	-1.62880100
Br	-4.73245800	2.20376600	-0.99233500

Br	-1.13303800	-0.34135900	5.33858500
Br	1.70808800	-1.15079100	3.14736900
Br	-3.89524300	0.31019400	3.01284600
Br	-3.40192100	-3.29046900	0.04475400
Br	-1.52738600	-4.75216500	-2.77000300
Br	0.39878800	-1.89145300	-4.11409000
O	3.00006600	-3.22870300	-0.89352400
C	3.51866400	-2.41077400	0.03811600
H	3.10298300	-1.22688600	-0.19296600
H	3.01004900	-2.47676700	1.01818200
C	1.60372000	-3.47749500	-0.73627700
H	1.30630200	-4.14980100	-1.54556400
H	1.41088300	-3.96129000	0.23296300
H	1.03575000	-2.53624800	-0.79188600
C	5.01176200	-2.36888100	0.09034700
H	5.39876400	-1.85147700	-0.80307800
H	5.38083600	-3.40636900	0.02779600
C	5.52118400	-1.70331900	1.37244800
H	4.89984600	-0.80878500	1.56323600
C	6.96235900	-1.25051500	1.19998500
H	7.06238100	-0.52120900	0.38349400
H	7.34680500	-0.78576800	2.11784600
H	7.61101400	-2.10855200	0.96515100
C	5.39599600	-2.64481100	2.56389000
H	4.38037000	-3.04993400	2.68640800
H	6.07370800	-3.50331400	2.44212900
H	5.66487200	-2.13763300	3.50013200

TS-2T

Zero-point correction= 0.401634 (Hartree/Particle)
Thermal correction to Energy= 0.447254
Thermal correction to Enthalpy= 0.448199
Thermal correction to Gibbs Free Energy= 0.309154
Sum of electronic and zero-point Energies= -25028.337546
Sum of electronic and thermal Energies= -25028.291925
Sum of electronic and thermal Enthalpies= -25028.290981
Sum of electronic and thermal Free Energies= -25028.430026

Ag	1.03388600	-0.52466600	-0.11379500
N	-0.78283700	-0.79043600	1.35222000
N	-1.76750100	0.13839000	1.44210400
N	0.44786900	1.80307400	0.59593700
N	-0.82022400	2.19245000	0.30467500

N	-0.78341000	0.44669000	-1.82375300
N	-1.90764900	0.40749200	-1.06256500
B	-1.97591700	1.16592700	0.29783300
C	-0.81560700	-1.48675300	2.47663600
C	-1.82668900	-1.03465000	3.33112700
C	-2.40487100	0.00635000	2.62530300
C	1.23041700	2.82118800	0.28341800
C	0.49867200	3.90155100	-0.22547000
C	-0.80600000	3.44366000	-0.20232100
C	-1.01749700	-0.34639600	-2.85652100
C	-2.28997300	-0.92693300	-2.79711200
C	-2.81590800	-0.41569200	-1.62454800
H	-3.03164900	1.70898600	0.43798500
C	2.88988500	-1.19764700	-0.98927700
H	2.98862400	-1.03267500	-2.07125700
C	4.13929900	-0.94398600	-0.27707300
C	4.23028400	-1.13924900	1.11354100
C	5.25999300	-0.42495100	-0.94748400
C	5.38496500	-0.82592400	1.81159700
H	3.35858000	-1.51544500	1.65123800
C	6.41258400	-0.07038800	-0.25872700
H	5.21294700	-0.27701400	-2.02668800
C	6.46182000	-0.28239000	1.11415000
H	5.44651500	-0.97111700	2.88798700
H	7.26899200	0.35299800	-0.77904700
Cl	7.91487300	0.14504200	1.99175500
Br	3.07852300	2.70387000	0.51573800
Br	1.12415500	5.55838600	-0.78500500
Br	-2.33091000	4.33824900	-0.77479500
Br	-2.29148800	-1.68774700	5.00568700
Br	0.38496800	-2.88134300	2.77942900
Br	-3.83114200	1.06740600	3.16448900
Br	-4.46614500	-0.78686900	-0.85147600
Br	-3.08406900	-2.12171400	-3.97721000
Br	0.26532800	-0.57410200	-4.19483200
C	2.88882000	-3.56101700	-1.44397900
H	2.55592000	-2.38134500	-0.91716300
C	1.82867100	-3.70646300	-2.51565100
H	1.80417100	-2.79054600	-3.12680100
H	2.16923400	-4.50653800	-3.19471700
C	2.70255900	-4.32688900	-0.16258200
H	3.45819100	-4.03579100	0.58007600
H	1.70366500	-4.17437600	0.26580500
H	2.83659900	-5.40192200	-0.36335300

C	4.28679500	-3.60009100	-1.97825500
H	4.39760500	-3.01099800	-2.89816800
H	5.02429900	-3.25732800	-1.24096600
H	4.53170600	-4.64652900	-2.21962600
C	0.43079300	-4.05552400	-2.06112300
H	-0.21954700	-4.17876500	-2.94754900
H	0.42728500	-5.02785700	-1.53155700
O	-0.08122300	-3.05120100	-1.21998500
C	-1.33077400	-3.41385600	-0.69039500
H	-1.68595900	-2.58013100	-0.07505900
H	-2.06586400	-3.61172900	-1.49111700
H	-1.25327600	-4.32036500	-0.06346400

107-P

Zero-point correction= 0.407840 (Hartree/Particle)
Thermal correction to Energy= 0.453566
Thermal correction to Enthalpy= 0.454510
Thermal correction to Gibbs Free Energy= 0.313877
Sum of electronic and zero-point Energies= -25028.421399
Sum of electronic and thermal Energies= -25028.375673
Sum of electronic and thermal Enthalpies= -25028.374729
Sum of electronic and thermal Free Energies= -25028.515362

Ag	1.11609700	0.97626600	-0.59187500
N	0.25091700	-0.22685500	1.28331200
N	-1.09779300	-0.37831400	1.32805900
N	-0.81904500	2.30885500	-0.20461800
N	-1.97684200	1.61619800	-0.00723200
N	-0.50427900	-0.22282500	-1.88773300
N	-1.57210400	-0.63510200	-1.15768100
B	-2.01458300	0.07608400	0.15148200
C	0.72905300	-0.76386500	2.39414400
C	-0.29154700	-1.28447100	3.19082500
C	-1.44123400	-1.01150900	2.46805300
C	-1.14779100	3.58579600	-0.28193100
C	-2.53045400	3.77205500	-0.12879900
C	-3.01344800	2.49109700	0.04236200
C	-0.36080300	-1.08882400	-2.87516900
C	-1.33150200	-2.09962800	-2.81604200
C	-2.07621900	-1.76735400	-1.70035500
H	-3.13232600	-0.24834900	0.40176600
C	3.97579000	-0.29973800	-1.53020900
H	3.94314700	-0.19520400	-2.62533300

C	5.03468000	0.56127000	-0.91582800
C	4.75899300	1.31040500	0.22792800
C	6.33065400	0.59666100	-1.44126800
C	5.73510000	2.09557800	0.84209600
H	3.74455900	1.29634000	0.63618200
C	7.32230200	1.36628800	-0.84887100
H	6.56610400	0.01814300	-2.33707500
C	7.01243800	2.10750600	0.28712400
H	5.50690900	2.68060100	1.72868100
H	8.32598200	1.39625200	-1.26297900
Cl	8.25774000	3.08507000	1.04081300
Br	0.13852800	4.90471800	-0.55790800
Br	-3.47808900	5.36699000	-0.15274000
Br	-4.78822300	2.00385700	0.31647900
Br	-0.14683600	-2.18380400	4.80407200
Br	2.56412100	-0.78649600	2.72158200
Br	-3.18394300	-1.41807600	2.95880700
Br	-3.51318400	-2.70779900	-0.99508600
Br	-1.53109400	-3.57238700	-3.92410800
Br	1.01248900	-0.91412000	-4.11920200
C	1.14134200	-4.10838500	1.10105500
H	1.31433200	-3.28806200	1.82208100
C	-0.28130900	-3.97150900	0.57619900
H	-0.47842200	-2.95953000	0.18431000
H	-0.47147000	-4.68753700	-0.23753400
H	-1.01973000	-4.16387600	1.36800100
C	1.31564300	-5.42791900	1.83250600
H	2.33850000	-5.54775800	2.22428200
H	0.62396200	-5.51083000	2.68146500
H	1.11872900	-6.27722000	1.15860600
C	2.19458100	-3.97165600	0.00268100
H	2.19083700	-4.87076300	-0.63503000
H	3.19369000	-3.92842400	0.47532300
C	2.02151500	-2.75456900	-0.89064500
H	1.84412200	-1.83590200	-0.28926500
H	1.14584400	-2.88148900	-1.54332700
O	3.13699700	-2.57007800	-1.74485700
C	4.15559700	-1.77679300	-1.18579100
H	2.98879800	0.03022200	-1.14339800
H	4.20877500	-1.89714400	-0.08624000
H	5.11826100	-2.12717400	-1.5903220

107-T

Zero-point correction= 0.407790 (Hartree/Particle)
Thermal correction to Energy= 0.451576
Thermal correction to Enthalpy= 0.452520
Thermal correction to Gibbs Free Energy= 0.320657
Sum of electronic and zero-point Energies= -25028.427927
Sum of electronic and thermal Energies= -25028.384141
Sum of electronic and thermal Enthalpies= -25028.383197
Sum of electronic and thermal Free Energies= -25028.515059

Ag	0.00834200	1.50412700	0.37095200
N	0.34311600	-0.78483900	1.20670400
N	-0.57261300	-1.66670200	0.72250900
N	-2.11329800	0.78317400	-0.04412600
N	-2.31315700	-0.41818000	-0.64237500
N	0.18288100	0.25196900	-1.99833400
N	-0.03993800	-1.03841300	-1.63845900
B	-1.18919900	-1.47981500	-0.69553800
C	0.74150000	-1.27089100	2.37313900
C	0.09899000	-2.47555100	2.68051400
C	-0.73354100	-2.68324200	1.59535800
C	-3.25590100	1.44589400	-0.11274800
C	-4.24184700	0.68992400	-0.75486100
C	-3.59283900	-0.49232500	-1.06982900
C	1.32986500	0.24285100	-2.66512100
C	1.89914300	-1.03344600	-2.72813700
C	0.97863400	-1.81910100	-2.06193800
H	-1.64605200	-2.51916900	-1.06120900
C	2.92636600	1.82913400	0.34056000
H	2.60252700	1.46265600	-0.64467100
C	1.96933600	2.90444400	0.79306500
C	1.80323400	3.22178000	2.15822200
C	1.22986800	3.66359600	-0.14302500
C	0.97241100	4.25351600	2.57005100
H	2.34714600	2.64512000	2.90518700
C	0.38663300	4.70498400	0.26153800
H	1.36488000	3.47541600	-1.20966800
C	0.27279700	4.98667800	1.61288000
H	0.86022200	4.48601200	3.62694500
H	-0.16935500	5.28075700	-0.47562200
Cl	-0.79445200	6.27195900	2.13334000
Br	-3.39384500	3.16163400	0.59825000
Br	-6.00410000	1.15782900	-1.09727700
Br	-4.32011600	-1.97420700	-1.92042500
Br	0.31320500	-3.54675800	4.18112700

Br	2.03452400	-0.41327100	3.40997000
Br	-1.91145200	-4.09473200	1.32876400
Br	1.04755700	-3.65584600	-1.77697800
Br	3.51588800	-1.54700600	-3.47642400
Br	2.01106000	1.79538500	-3.45528700
C	4.42190600	2.19582600	0.21852000
H	2.84425100	0.96589900	1.01440400
C	5.13742000	1.03797300	-0.50538200
H	4.74890400	0.96850800	-1.53669800
H	6.20396100	1.30275900	-0.59179300
C	5.03663400	2.44272200	1.59376900
H	4.59578200	3.32890600	2.07345300
H	4.89249300	1.59326300	2.27735600
H	6.11847500	2.62424200	1.50917000
C	4.59946400	3.45110100	-0.63151600
H	4.16223600	3.32345000	-1.63327300
H	4.12799400	4.32783500	-0.16390100
H	5.66671000	3.68180200	-0.76346200
C	5.06297700	-0.34965100	0.10212900
H	5.79845200	-1.00431200	-0.40578200
H	5.34301900	-0.34045900	1.17489200
O	3.77226000	-0.89064700	-0.04770400
C	3.69023100	-2.19781300	0.43939900
H	2.64310800	-2.52141400	0.37446500
H	4.30773800	-2.89440000	-0.15718000
H	4.01517900	-2.26534000	1.49402600

107-S

Zero-point correction= 0.407862 (Hartree/Particle)
Thermal correction to Energy= 0.452753
Thermal correction to Enthalpy= 0.453697
Thermal correction to Gibbs Free Energy= 0.318062
Sum of electronic and zero-point Energies= -25028.423215
Sum of electronic and thermal Energies= -25028.378324
Sum of electronic and thermal Enthalpies= -25028.377380
Sum of electronic and thermal Free Energies= -25028.513015

Ag	1.01183600	0.67969700	0.01517300
N	-0.33151800	-0.97493400	1.33425100
N	-1.65497300	-0.91787500	1.02665700
N	-1.01498400	1.78387200	-0.09076800
N	-2.10960600	1.07713100	-0.47304600
N	-0.05192300	-0.55198100	-1.85516800

N	-1.21547700	-1.11686300	-1.44083700
B	-2.13997900	-0.46751200	-0.38004700
C	-0.26653800	-1.40067900	2.58726100
C	-1.53616400	-1.62871500	3.12909200
C	-2.39026400	-1.30160500	2.09135400
C	-1.31853800	3.06529900	-0.22156300
C	-2.62576400	3.23189000	-0.68922100
C	-3.08961500	1.93591100	-0.83380300
C	0.49748900	-1.41865700	-2.69396900
C	-0.28177000	-2.57164700	-2.83930500
C	-1.36219800	-2.33284400	-2.00879100
H	-3.25788200	-0.84066900	-0.55387400
C	3.55090800	-0.94315300	0.29180900
H	3.32232900	-1.31996900	-0.71810100
C	3.36523500	0.55081500	0.35278500
C	3.29253800	1.22108100	1.59626800
C	3.44610700	1.34706100	-0.81160200
C	3.36043100	2.60701700	1.67843400
H	3.22303100	0.63310100	2.51188100
C	3.51281000	2.73870800	-0.74024100
H	3.51349900	0.85807200	-1.78355900
C	3.48953000	3.34799700	0.50696700
H	3.31668000	3.10658200	2.64391400
H	3.58590300	3.33541800	-1.64697100
Cl	3.57919500	5.09193900	0.60759100
Br	-0.07294800	4.38494100	0.19380300
Br	-3.53028500	4.81620700	-1.02998300
Br	-4.77249500	1.41633500	-1.42293600
Br	-1.97254900	-2.23980300	4.82594300
Br	1.36573500	-1.66869200	3.45652800
Br	-4.24759900	-1.34722000	2.10332100
Br	-2.79237900	-3.46643800	-1.65917500
Br	0.03923600	-4.06142300	-3.89737200
Br	2.11131400	-1.04716900	-3.55699200
O	5.08379900	-2.79667300	0.54042400
C	4.98099000	-1.39218400	0.64062600
H	2.84899900	-1.42636600	0.98669500
H	5.18793000	-1.09495200	1.69140300
C	4.36320000	-3.51371100	1.50699200
H	4.73580600	-4.54411500	1.49502400
H	4.51881800	-3.09685300	2.51854600
H	3.28009200	-3.54233100	1.30306800
C	6.03345400	-0.79665000	-0.28857400
H	5.58946800	-0.64583200	-1.28810600

H	6.82250800	-1.55458500	-0.41843800
C	6.68786100	0.49975300	0.20124700
H	5.90515000	1.18438100	0.57231000
C	7.40637300	1.18731000	-0.95032000
H	6.70894500	1.46551500	-1.75369700
H	7.91894500	2.10061600	-0.61816900
H	8.16560600	0.51882500	-1.38529800
C	7.65878500	0.22780300	1.34354300
H	7.18189100	-0.29434800	2.18491700
H	8.49485400	-0.39893000	0.99710400
H	8.08347900	1.16261100	1.73468400

Cartesian coordinates of computed structures with single point energy calculations at SMD// M062X/6-31+G(d,p)-SDD(Ag) level.

AgTp^{Br3}-THF

Zero-point correction=	0.229977 (Hartree/Particle)
Thermal correction to Energy=	0.261378
Thermal correction to Enthalpy=	0.262322
Thermal correction to Gibbs Free Energy=	0.157636
Sum of electronic and zero-point Energies=	-24219.147617
Sum of electronic and thermal Energies=	-24219.116216
Sum of electronic and thermal Enthalpies=	-24219.115271
Sum of electronic and thermal Free Energies=	-24219.219957

Ag	-1.59688600	0.56942900	-1.15095000
N	-1.22419300	0.14263900	1.20592900
N	0.00213400	-0.26370900	1.61695700
N	-0.12980500	-1.32843300	-1.24658600
N	0.97150800	-1.36612400	-0.45549600
N	0.48769500	1.66341000	-0.81128900
N	1.41761500	1.10068200	0.00181500
B	1.21098900	-0.29745300	0.64431600
C	-2.03358000	0.01647600	2.24151200
C	-1.36056100	-0.47984100	3.36406700
C	-0.06239600	-0.64615200	2.91364300
C	-0.04944300	-2.36493400	-2.06284400
C	1.10821500	-3.11581200	-1.82738200
C	1.72753200	-2.43791500	-0.79060100
C	0.95235000	2.85061700	-1.15934500
C	2.20415900	3.09986700	-0.58647700
C	2.45724900	1.95428800	0.14820900

H	2.18633500	-0.58719900	1.25706300
Br	-1.38177800	-2.67558300	-3.32865200
Br	1.67482700	-4.65660800	-2.69356000
Br	3.32949500	-2.88308600	0.04027700
Br	-2.04467900	-0.82923500	5.05416500
Br	-3.83699100	0.46827200	2.09291800
Br	1.38623500	-1.29488600	3.88006000
Br	3.95905800	1.59951300	1.18412500
Br	3.27053000	4.60930300	-0.75817700
Br	-0.04306000	3.96200700	-2.27567900
C	-4.61814900	-0.18386400	-1.98768900
O	-3.63942800	0.87546600	-2.06965200
C	-4.28333600	2.15941100	-1.94313800
C	-5.75274800	1.88225400	-2.17892900
C	-5.90775700	0.49315300	-1.56668200
H	-4.70347500	-0.64621300	-2.98174200
H	-4.25608700	-0.94064000	-1.27895900
H	-4.10021700	2.55604600	-0.93138400
H	-3.82568000	2.83588800	-2.67437400
H	-6.39766100	2.63733900	-1.71677800
H	-5.96913100	1.85285000	-3.25580700
H	-5.96853900	0.56287000	-0.47088800
H	-6.79254900	-0.04455000	-1.92388500

THF

Zero-point correction= 0.117795 (Hartree/Particle)
Thermal correction to Energy= 0.122584
Thermal correction to Enthalpy= 0.123528
Thermal correction to Gibbs Free Energy= 0.089826
Sum of electronic and zero-point Energies= -232.241762
Sum of electronic and thermal Energies= -232.236973
Sum of electronic and thermal Enthalpies= -232.236029
Sum of electronic and thermal Free Energies= -232.269731

C	-1.15693500	-0.41992300	-0.13331600
O	-0.00285600	-1.24211000	-0.00511400
C	1.15300600	-0.42589400	0.13978200
C	0.72715000	0.98201700	-0.23995100
C	-0.72010800	0.98790100	0.23696000
H	-1.52268300	-0.45928800	-1.17424500
H	-1.95098600	-0.81616400	0.51476200
H	1.50353600	-0.46286700	1.18635300
H	1.95400600	-0.82860300	-0.49529500

H	1.35063500	1.75537800	0.22273900
H	0.76854300	1.11380000	-1.33049300
H	-0.75996200	1.12776400	1.32652600
H	-1.33891400	1.76225800	-0.23028600

AgTp^{Br3}

Zero-point correction=	0.111621 (Hartree/Particle)
Thermal correction to Energy=	0.136970
Thermal correction to Enthalpy=	0.137915
Thermal correction to Gibbs Free Energy=	0.048835
Sum of electronic and zero-point Energies=	-23986.840950
Sum of electronic and thermal Energies=	-23986.815600
Sum of electronic and thermal Enthalpies=	-23986.814656
Sum of electronic and thermal Free Energies=	-23986.903736

Ag	0.02336100	-2.02193500	1.31818900
N	1.12572700	-1.64473400	-0.68036400
N	0.91663700	-0.44055600	-1.27449500
N	-1.82501500	-1.21424200	0.07329300
N	-1.62888800	-0.45153800	-1.04370600
N	0.11592400	0.21455800	1.40179300
N	-0.18270600	1.02622700	0.36697500
B	-0.34804800	0.42127700	-1.02904500
C	2.36867900	-2.02153000	-0.98171900
C	2.97871100	-1.12785400	-1.87477100
C	2.03469800	-0.11203100	-1.97604400
C	-3.12802900	-1.37138800	0.21534200
C	-3.83265900	-0.56300400	-0.67387500
C	-2.82635800	0.06431100	-1.37643900
C	0.77101900	0.96027700	2.25846700
C	0.71952600	2.30789900	1.95854300
C	0.16522800	2.28877600	0.68628500
H	-0.30822700	1.28341900	-1.84393400
Br	-0.11164200	3.61964700	-0.55151700
Br	1.29004300	3.73218100	3.01562100
Br	1.71981700	0.12638600	3.64503800
Br	2.26097700	1.60890100	-2.66147500
Br	4.77812500	-1.03203400	-2.34637800
Br	3.03594600	-3.48907200	-0.08935000
Br	-3.12204800	1.31785400	-2.71597400
Br	-5.66796600	-0.34161100	-0.89241900
Br	-3.73199900	-2.49481300	1.55974300

2a-1

Zero-point correction= 0.105529 (Hartree/Particle)
Thermal correction to Energy= 0.113911
Thermal correction to Enthalpy= 0.114856
Thermal correction to Gibbs Free Energy= 0.071404
Sum of electronic and zero-point Energies= -839.119477
Sum of electronic and thermal Energies= -839.111095
Sum of electronic and thermal Enthalpies= -839.110151
Sum of electronic and thermal Free Energies= -839.153602

N	-3.35468800	-0.17482500	0.13233000
C	-2.50887900	0.74144900	-0.36236800
N	-4.05632400	-0.98532800	0.49100000
H	-2.84869500	1.74977400	-0.10718500
C	-1.06335200	0.43454700	-0.22273400
C	-0.17872400	1.49198200	0.00971400
C	-0.56213900	-0.86560900	-0.34662600
C	1.18630400	1.25908800	0.11652000
H	-0.56133600	2.50773200	0.09837200
C	0.79919400	-1.11045800	-0.22733600
H	-1.23546200	-1.69815100	-0.55059700
C	1.66087800	-0.04245400	0.00088100
H	1.87567200	2.08156600	0.29383600
H	1.18896800	-2.12164500	-0.32297900
Cl	3.37989700	-0.34408600	0.14217200

Int-1

Zero-point correction= 0.218376 (Hartree/Particle)
Thermal correction to Energy= 0.255915
Thermal correction to Enthalpy= 0.256859
Thermal correction to Gibbs Free Energy= 0.135145
Sum of electronic and zero-point Energies= -24826.024247
Sum of electronic and thermal Energies= -24825.986708
Sum of electronic and thermal Enthalpies= -24825.985764
Sum of electronic and thermal Free Energies= -24826.107478

Ag	-1.10594700	-1.21054800	-0.35456600
N	-0.93803300	1.17943700	-0.34080700
N	0.28512200	1.76250100	-0.24449900
N	0.48455500	-0.90709600	1.43266200
N	1.35932600	0.12908900	1.38157900
N	0.93365600	-0.83029700	-1.64313200

N	1.88385600	-0.03669100	-1.08795400
B	1.55936900	0.94126600	0.07129700
C	-1.80431800	2.14807100	-0.58657700
C	-1.17167300	3.39514000	-0.65501500
C	0.16020700	3.09666700	-0.43083500
C	0.54459300	-1.39153900	2.66185300
C	1.46323600	-0.68816300	3.44782500
C	1.95604500	0.27542400	2.58522100
C	1.54958300	-1.58212500	-2.53819100
C	2.91785700	-1.29676600	-2.60337900
C	3.08250200	-0.30286000	-1.65541700
H	2.47640600	1.68391400	0.21420800
Br	-0.50900300	-2.85008700	3.15058400
Br	1.93144600	-0.97660700	5.22070600
Br	3.22439200	1.57805700	2.96772900
Br	-1.94117200	5.05485400	-0.97047900
Br	-3.61769600	1.77525800	-0.79434800
Br	1.58049700	4.29652300	-0.37355900
Br	4.66867000	0.53961600	-1.17950700
Br	4.20248400	-2.06486000	-3.70145700
Br	0.61257300	-2.84826000	-3.53789000
N	-2.39368700	-3.82549900	0.05068300
C	-2.70539400	-2.80355300	-0.76057700
N	-2.11180600	-4.63924400	0.78294900
H	-2.45569600	-3.05073900	-1.79685100
C	-4.00357200	-2.13478600	-0.49464700
C	-4.73928400	-1.65498800	-1.58243800
C	-4.49238400	-1.94522200	0.80231000
C	-5.94464900	-0.99464500	-1.38246100
H	-4.36018000	-1.79140100	-2.59420500
C	-5.70308400	-1.29886600	1.01168500
H	-3.92036400	-2.29282600	1.66257700
C	-6.41641300	-0.82719600	-0.08554000
H	-6.51600000	-0.61790600	-2.22789800
H	-6.08362000	-1.15203200	2.02025000
Cl	-7.93912000	-0.00234100	0.17320400

TS-1

Zero-point correction= 0.216398 (Hartree/Particle)
Thermal correction to Energy= 0.253072
Thermal correction to Enthalpy= 0.254016
Thermal correction to Gibbs Free Energy= 0.136330
Sum of electronic and zero-point Energies= -24825.997786

Sum of electronic and thermal Energies= -24825.961111
Sum of electronic and thermal Enthalpies= -24825.960167
Sum of electronic and thermal Free Energies= -24826.077853

Ag	-1.17556300	0.49354700	1.02310000
N	-0.82588700	-1.09169500	-0.83134100
N	0.45561400	-1.35913900	-1.18947700
N	0.29007000	1.71313600	-0.38272500
N	1.21154800	1.08152900	-1.15227000
N	1.01744800	-0.49292300	1.57753800
N	1.98594700	-0.53561900	0.62892100
B	1.61818800	-0.38851500	-0.87181600
C	-1.53081000	-2.16979100	-1.13483800
C	-0.72928100	-3.17145000	-1.69568100
C	0.53408000	-2.60758100	-1.69993000
C	0.23788500	2.96546700	-0.80172500
C	1.12919800	3.19205300	-1.85598200
C	1.72864300	1.95846300	-2.04252900
C	1.63314100	-0.57773300	2.74422100
C	3.02096100	-0.68207300	2.59069200
C	3.19587500	-0.64654800	1.21812100
H	2.55933800	-0.66561800	-1.54453900
Br	-0.91096100	4.19108500	0.00736600
Br	1.44707700	4.77106000	-2.77857400
Br	3.03235000	1.53127400	-3.29544300
Br	-1.22780800	-4.85621300	-2.29629800
Br	-3.36429200	-2.24687900	-0.79735800
Br	2.12192100	-3.39477200	-2.26144300
Br	4.80030600	-0.72631300	0.28229100
Br	4.32092600	-0.83035700	3.90756500
Br	0.66784500	-0.53704500	4.34036400
N	-2.84841400	2.39922500	2.80664700
C	-2.96237200	0.68134900	2.19528700
N	-2.63860000	3.48911800	2.78477600
H	-2.94809900	0.24039900	3.20001500
C	-4.28536300	0.62885400	1.57600500
C	-5.34165500	-0.04469500	2.20758100
C	-4.49174000	1.16953800	0.29595000
C	-6.56276900	-0.20846500	1.56946900
H	-5.19553100	-0.45912900	3.20473200
C	-5.71690200	1.05227900	-0.33743300
H	-3.66584500	1.67764400	-0.20464700
C	-6.73529200	0.35197800	0.30735300
H	-7.37780800	-0.74677400	2.04766500

H	-5.87991300	1.47323500	-1.32686100
Cl	-8.27441500	0.16978500	-0.49489600

Int-2

Zero-point correction= 0.215029 (Hartree/Particle)
Thermal correction to Energy= 0.253936
Thermal correction to Enthalpy= 0.254881
Thermal correction to Gibbs Free Energy= 0.129507
Sum of electronic and zero-point Energies= -24826.022326
Sum of electronic and thermal Energies= -24825.983419
Sum of electronic and thermal Enthalpies= -24825.982475
Sum of electronic and thermal Free Energies= -24826.107849

Ag	-1.37011600	-0.29019400	-0.75323000
N	-0.08846700	1.67641200	-0.78686200
N	1.24348100	1.68170200	-0.53126600
N	-0.05510800	-0.60043600	1.25736000
N	1.12583400	0.05243500	1.38556500
N	0.97398600	-0.92355400	-1.79057900
N	1.93320500	-0.75123900	-0.84596200
B	1.93107400	0.45565300	0.12321200
C	-0.39262600	2.87479300	-1.25990900
C	0.73938900	3.69800800	-1.31748500
C	1.75473500	2.88949400	-0.84288600
C	-0.51639900	-0.78324400	2.48377100
C	0.35019300	-0.25544500	3.44688600
C	1.38197400	0.27666300	2.69241900
C	1.20587500	-2.10327300	-2.33832100
C	2.31668300	-2.73916600	-1.76735700
C	2.73892100	-1.83426800	-0.80783400
H	3.04780000	0.74335900	0.42995000
Br	-2.14314300	-1.66007100	2.77564200
Br	0.17066100	-0.26610500	5.29548300
Br	2.88377200	1.18280300	3.30131900
Br	0.84658400	5.46796700	-1.87243200
Br	-2.13499600	3.29904800	-1.75915700
Br	3.54526800	3.35357400	-0.62973700
Br	4.13852000	-2.03173500	0.40448900
Br	3.01210000	-4.41400200	-2.15636300
Br	0.09590000	-2.77117000	-3.68411400
C	-3.15357800	-1.17012300	-1.34711800
H	-3.14469700	-1.99975400	-2.07341700
C	-4.46248300	-0.90918100	-0.88638400

C	-5.57926800	-1.66459300	-1.34531400
C	-4.68784000	0.12185700	0.06556300
C	-6.85786700	-1.40254900	-0.88646800
H	-5.42209900	-2.46156400	-2.07302900
C	-5.95990900	0.39534800	0.52799600
H	-3.82658300	0.68590800	0.42838100
C	-7.02611100	-0.37047500	0.04026500
H	-7.71423600	-1.97487700	-1.23266100
H	-6.13940400	1.18508200	1.25548800
Cl	-8.62700600	-0.02522300	0.61523400

Methyl isoamyl ether

Zero-point correction= 0.195003 (Hartree/Particle)
Thermal correction to Energy= 0.203887
Thermal correction to Enthalpy= 0.204831
Thermal correction to Gibbs Free Energy= 0.161919
Sum of electronic and zero-point Energies= -311.955615
Sum of electronic and thermal Energies= -311.946732
Sum of electronic and thermal Enthalpies= -311.945788
Sum of electronic and thermal Free Energies= -311.988700

C	3.28562500	0.15255900	0.06325100
H	3.34668300	1.09780700	-0.50549100
H	3.38507700	0.39540100	1.13647600
H	4.12617900	-0.48681100	-0.22921000
O	2.10022600	-0.54065100	-0.20485900
C	0.96504300	0.21512100	0.14274900
H	0.99083200	0.46669700	1.22331800
H	0.97587700	1.17622800	-0.40627800
C	-0.27264400	-0.59073300	-0.18180100
H	-0.34772900	-0.71222900	-1.27585600
H	-0.13527900	-1.60046400	0.23601000
C	-1.57014700	0.01049400	0.35392200
H	-1.49008000	0.06205900	1.45453300
C	-1.80623500	1.42142500	-0.17084900
H	-2.77954700	1.80875200	0.16096900
H	-1.03932700	2.12937000	0.17093400
H	-1.80392900	1.43150500	-1.27221200
C	-2.74625500	-0.88845400	0.00007700
H	-2.60585900	-1.91084600	0.37744100
H	-3.68733700	-0.50327200	0.41623500
H	-2.86968700	-0.95146300	-1.09210300

Int-3S

Zero-point correction= 0.404067 (Hartree/Particle)
Thermal correction to Energy= 0.449205
Thermal correction to Enthalpy= 0.450149
Thermal correction to Gibbs Free Energy= 0.313375
Sum of electronic and zero-point Energies= -25028.500838
Sum of electronic and thermal Energies= -25028.455700
Sum of electronic and thermal Enthalpies= -25028.454755
Sum of electronic and thermal Free Energies= -25028.591530

Ag	0.89632800	0.43591000	-0.68006100
N	-0.15645500	-0.34885400	1.34420600
N	-1.50877000	-0.31903000	1.45372500
N	-1.07097600	1.72564000	-0.63619800
N	-2.28662500	1.16374300	-0.41586800
N	-1.04384700	-0.98176600	-1.84811800
N	-1.88890700	-1.30762800	-0.83922300
B	-2.39957600	-0.26017200	0.18040800
C	0.31881100	-0.26424300	2.57829900
C	-0.70964300	-0.18006000	3.52325400
C	-1.85882400	-0.21088800	2.75201900
C	-1.29240900	2.94216400	-1.10354800
C	-2.66234600	3.21417200	-1.19339600
C	-3.25481200	2.04821900	-0.74042600
C	-0.79441900	-2.10812900	-2.49428400
C	-1.46121000	-3.20011900	-1.92275700
C	-2.14343700	-2.63356400	-0.86059400
H	-3.53128300	-0.49421700	0.47529900
C	2.77042500	0.28889400	-1.55806800
H	2.92602600	-0.47362800	-2.33838200
C	3.95429100	1.01149800	-1.30045900
C	3.98418800	2.00510200	-0.28640400
C	5.15320000	0.73590300	-2.01140500
C	5.15160100	2.66885500	0.02734200
H	3.06060700	2.22616500	0.24992700
C	6.32550200	1.39981700	-1.71576700
H	5.13585400	-0.01755100	-2.79866600
C	6.31019800	2.34638700	-0.68693800
H	5.18502500	3.42130000	0.81141200
H	7.24862600	1.19053400	-2.25134500
Cl	7.79231600	3.14082900	-0.27247700
Br	0.12261000	4.06913600	-1.55241700
Br	-3.49005400	4.77288400	-1.76685400

Br	-5.07282700	1.70895800	-0.56559200
Br	-0.56762700	-0.05453200	5.36913100
Br	2.15404100	-0.23263200	2.91380000
Br	-3.61742900	-0.10426600	3.34299700
Br	-3.23478600	-3.48152200	0.38369700
Br	-1.44455700	-4.98090800	-2.44866200
Br	0.34557300	-2.11525700	-3.97341700
O	3.28738400	-3.02373500	-0.88937200
C	3.76877600	-2.39761100	0.27413500
H	3.23283300	-1.43701700	0.43033600
H	3.54337600	-3.02727000	1.15718100
C	1.92160600	-3.32022700	-0.78581800
H	1.61170800	-3.79204400	-1.72503000
H	1.71655200	-4.01714700	0.04734300
H	1.31659900	-2.40518900	-0.62263800
C	5.25367400	-2.15020500	0.13406900
H	5.42646700	-1.69437900	-0.85610600
H	5.79270500	-3.11289600	0.13409900
C	5.82700300	-1.23441100	1.21407700
H	5.27227400	-0.27726800	1.16801700
C	7.29684400	-0.94713300	0.93985500
H	7.45221100	-0.54326000	-0.07177300
H	7.70598700	-0.22085200	1.65662000
H	7.89293600	-1.86887400	1.02386100
C	5.65919600	-1.81698500	2.61214800
H	4.60531400	-1.96702300	2.88568900
H	6.15904500	-2.79552100	2.68550500
H	6.10592700	-1.15950900	3.37100200

Int-3P

Zero-point correction=	0.403870 (Hartree/Particle)
Thermal correction to Energy=	0.450681
Thermal correction to Enthalpy=	0.451625
Thermal correction to Gibbs Free Energy=	0.308550
Sum of electronic and zero-point Energies=	-25028.503419
Sum of electronic and thermal Energies=	-25028.456608
Sum of electronic and thermal Enthalpies=	-25028.455664
Sum of electronic and thermal Free Energies=	-25028.598739

Ag	-1.47433700	0.10798900	0.77302700
N	-0.20683800	-0.65369600	-1.14079200
N	0.99354800	-0.11141900	-1.46631500
N	-0.54468300	2.12955600	-0.10054300

N	0.76915600	2.21788800	-0.45902200
N	0.87172200	0.24676100	1.73758500
N	1.89345700	0.36576600	0.85125200
B	1.67034200	0.96120400	-0.55557700
C	-0.47112500	-1.57549400	-2.04599500
C	0.56907600	-1.68058600	-2.98618500
C	1.48195800	-0.71717600	-2.57475300
C	-1.00356800	3.37014800	-0.04828400
C	-0.00301800	4.30979500	-0.36152800
C	1.11126400	3.52103300	-0.61200800
C	1.38147200	-0.34974600	2.81486300
C	2.73750700	-0.65098300	2.64111800
C	3.02241500	-0.16704700	1.37526200
H	2.71092000	1.26306100	-1.04032000
C	-3.21818300	-0.61518000	1.65142600
H	-3.18505000	-1.15317800	2.60727900
C	-4.53806300	-0.58174900	1.13784700
C	-4.81902700	0.06773100	-0.10467700
C	-5.61850100	-1.24309000	1.79817100
C	-6.07983100	0.07249500	-0.64042800
H	-3.98589200	0.56286200	-0.62676800
C	-6.89507000	-1.25230700	1.26761600
H	-5.42207400	-1.75093900	2.74348600
C	-7.10840600	-0.59287800	0.04958300
H	-6.29476800	0.57698600	-1.59531900
H	-7.71927300	-1.75325100	1.78143100
Cl	-8.71073300	-0.60011400	-0.61934900
Br	-2.77070800	3.72671700	0.42012200
Br	-0.13236900	6.15991000	-0.44177800
Br	2.81085700	4.12694000	-1.08985500
Br	0.72067400	-2.85963300	-4.40713300
Br	-2.03598500	-2.58375200	-1.99306200
Br	3.10831800	-0.32235100	-3.37289400
Br	4.64159600	-0.26528200	0.45945500
Br	3.88153800	-1.53707300	3.80451200
Br	0.32741900	-0.71393900	4.29815100
C	1.25302600	-4.30757400	-0.41206900
H	0.64092200	-3.87960700	-1.22395200
C	2.33747000	-3.28882400	-0.06012600
H	1.90691000	-2.29107600	0.14478400
H	2.90349600	-3.59337100	0.83540400
H	3.05649200	-3.17938100	-0.88756200
C	1.88872100	-5.59124200	-0.94630100
H	1.12717700	-6.33855400	-1.20187500

H	2.48591700	-5.39016100	-1.84136300
H	2.56667900	-6.03473800	-0.18798700
C	0.33907700	-4.61613900	0.76420500
H	0.88374500	-5.19759200	1.52165700
H	-0.47523200	-5.27712400	0.40217500
C	-0.27063100	-3.38578300	1.41261100
H	-0.63523600	-2.68078100	0.64082900
H	0.50173300	-2.84862900	1.99169100
O	-1.31165900	-3.68099800	2.33016100
C	-2.53434000	-3.93248700	1.70867000
H	-2.86296200	-3.06997500	1.10058300
H	-2.51637200	-4.81163600	1.03427800
H	-3.27401500	-4.10972400	2.49242400

Int-3T

Zero-point correction= 0.403083 (Hartree/Particle)
Thermal correction to Energy= 0.447606
Thermal correction to Enthalpy= 0.448551
Thermal correction to Gibbs Free Energy= 0.311620
Sum of electronic and zero-point Energies= -25028.508024
Sum of electronic and thermal Energies= -25028.463500
Sum of electronic and thermal Enthalpies= -25028.462556
Sum of electronic and thermal Free Energies= -25028.599487

Ag	0.99605400	-0.05965700	-1.03415700
N	0.38203000	-1.03213900	1.07866800
N	-0.68068300	-0.59321900	1.81210200
N	0.31519600	1.96338700	0.82272200
N	-1.01376000	1.82858500	1.06797500
N	-1.36503700	0.21916900	-1.27329200
N	-2.13485900	-0.02912000	-0.17994100
B	-1.67737300	0.44343800	1.24246600
C	0.99619000	-1.93499500	1.81058800
C	0.37628500	-2.11075500	3.04879500
C	-0.70095200	-1.23913800	3.00199600
C	0.48050100	3.22375700	0.47401600
C	-0.72145000	3.94131300	0.46146200
C	-1.65283400	2.99727100	0.84807300
C	-2.01972200	-0.27599600	-2.31154300
C	-3.23930900	-0.85627000	-1.91889300
C	-3.25928600	-0.67303200	-0.55614000
H	-2.62539100	0.50498500	1.95310700
C	2.83812300	0.21026700	-1.98884100

H	2.97831100	0.03327000	-3.07125400
C	4.04495000	0.55810600	-1.35507400
C	4.07118300	0.83383700	0.03844900
C	5.26502100	0.66550700	-2.08776300
C	5.24167000	1.19102600	0.68156900
H	3.13325800	0.75633000	0.58571300
C	6.44787300	1.00918000	-1.45668700
H	5.26619700	0.45199100	-3.15498800
C	6.41968200	1.26439700	-0.07238400
H	5.25528300	1.39603600	1.74497900
H	7.38196900	1.08128200	-1.99849700
Cl	7.89449700	1.70153600	0.71539200
Br	2.18189500	3.88597900	0.03493400
Br	-1.01881800	5.72831800	0.05353300
Br	-3.49620100	3.20897400	1.01678800
Br	0.84738500	-3.25874000	4.43141400
Br	2.52022900	-2.82018100	1.18824200
Br	-1.95731200	-0.95289100	4.34012400
Br	-4.55707200	-1.24969200	0.65613800
Br	-4.49327900	-1.71409700	-2.98098000
Br	-1.32483100	-0.16424600	-4.04087300
C	2.76213800	-3.20752200	-2.72294800
H	2.65840300	-2.50420500	-1.87848800
C	1.50112500	-3.07188300	-3.58998800
H	1.40453100	-2.02974600	-3.91392600
H	1.63417000	-3.67324900	-4.50932600
C	2.91241800	-4.60627300	-2.13720500
H	3.86719300	-4.70492700	-1.60107700
H	2.11200700	-4.85090300	-1.41371900
H	2.88617600	-5.37008200	-2.93034100
C	3.99145700	-2.84619000	-3.52995600
H	3.90031800	-1.85738300	-4.01178900
H	4.90103300	-2.81798900	-2.90225100
H	4.16700800	-3.57653500	-4.33690600
C	0.21037100	-3.51025800	-2.93850800
H	-0.63485500	-3.36923100	-3.63312300
H	0.24657300	-4.58150600	-2.68623900
O	-0.00628200	-2.75837600	-1.75856500
C	-1.02139800	-3.31229500	-0.95520200
H	-1.20771900	-2.62618400	-0.11851600
H	-1.96165600	-3.46326400	-1.51872200
H	-0.70093500	-4.28788800	-0.54146500

TS-2P

Zero-point correction= 0.402437 (Hartree/Particle)
Thermal correction to Energy= 0.448063
Thermal correction to Enthalpy= 0.449007
Thermal correction to Gibbs Free Energy= 0.309655
Sum of electronic and zero-point Energies= -25028.498923
Sum of electronic and thermal Energies= -25028.453296
Sum of electronic and thermal Enthalpies= -25028.452352
Sum of electronic and thermal Free Energies= -25028.591704

Ag	1.58736500	0.16212500	-0.76244700
N	-0.03971200	-0.32752400	1.24735700
N	-1.28671400	0.20498300	1.23133800
N	0.49689000	2.15357200	-0.35428300
N	-0.84480500	2.27075600	-0.17527500
N	-0.62942000	-0.07689400	-1.97129800
N	-1.76000700	0.13658800	-1.25385000
B	-1.76974100	1.03938000	0.01028700
C	0.07219000	-0.97408300	2.39336800
C	-1.10309700	-0.90225600	3.15109600
C	-1.94101600	-0.12828300	2.36741000
C	0.99169300	3.37881700	-0.38346900
C	-0.01291600	4.33871700	-0.22167600
C	-1.16470500	3.58368700	-0.08847500
C	-0.93452000	-0.97509100	-2.89182000
C	-2.27181800	-1.37981000	-2.79880400
C	-2.75791200	-0.64013900	-1.73494500
H	-2.88152000	1.41161700	0.21694500
C	3.21077600	-1.16694600	-1.26290300
H	3.22172500	-1.49721400	-2.31027700
C	4.55887000	-1.08956800	-0.69462100
C	4.75955000	-0.62803600	0.61801200
C	5.68459400	-1.45625900	-1.45024100
C	6.03072300	-0.53844100	1.16235500
H	3.89239000	-0.32867100	1.20956400
C	6.96753900	-1.34833500	-0.93118700
H	5.54666500	-1.82457600	-2.46709700
C	7.12221900	-0.89673700	0.37516500
H	6.18083000	-0.17962900	2.17818700
H	7.83799000	-1.61982100	-1.52464200
Cl	8.73247500	-0.76914800	1.04818900
Br	2.82145400	3.65640900	-0.60254700
Br	0.15678400	6.18638800	-0.18502100
Br	-2.88438300	4.23032200	0.19269100

Br	-1.47628400	-1.70354800	4.78359600
Br	1.66049900	-1.84433300	2.85773700
Br	-3.67547100	0.39997600	2.77621600
Br	-4.47575500	-0.69043500	-1.02770700
Br	-3.17192000	-2.63817800	-3.82487200
Br	0.34915800	-1.58091100	-4.10209000
C	-1.22626000	-4.16765700	1.28589800
H	-0.73495100	-3.47974600	1.99684700
C	-2.40090200	-3.42345800	0.66281100
H	-2.09041800	-2.47078300	0.20752700
H	-2.88664200	-4.02445200	-0.12387300
H	-3.16265000	-3.18998600	1.42108800
C	-1.70332500	-5.38484700	2.06148700
H	-0.86415200	-5.93467100	2.51029500
H	-2.38541200	-5.09363400	2.87231500
H	-2.24566300	-6.07975600	1.40233700
C	-0.17555600	-4.57541700	0.24952700
H	-0.55601200	-5.40500400	-0.36866200
H	0.71665400	-4.95731700	0.77265300
C	0.22740400	-3.42671600	-0.65072300
H	0.37527800	-2.49053600	-0.08515700
H	-0.52756200	-3.23342300	-1.42237400
O	1.43647400	-3.71954100	-1.38427000
C	2.56833800	-3.40899200	-0.76064000
H	2.59558400	-2.10170000	-0.68778600
H	2.60259300	-3.54422700	0.33474900
H	3.45621800	-3.76184300	-1.28783600

TS-2S

Zero-point correction= 0.400289 (Hartree/Particle)
Thermal correction to Energy= 0.444590
Thermal correction to Enthalpy= 0.445534
Thermal correction to Gibbs Free Energy= 0.310776
Sum of electronic and zero-point Energies= -25028.499160
Sum of electronic and thermal Energies= -25028.454859
Sum of electronic and thermal Enthalpies= -25028.453915
Sum of electronic and thermal Free Energies= -25028.588673

Ag	0.98406500	0.31638400	-0.57712600
N	-0.40293300	-0.74330500	1.36105700
N	-1.71566900	-0.40223300	1.34705700
N	-0.79847800	1.75125600	-0.42340400
N	-2.08782200	1.32651900	-0.43679800

N	-1.04193300	-0.79002100	-2.00288700
N	-1.94027800	-1.12857400	-1.04652100
B	-2.44426400	-0.11395100	0.00838300
C	-0.04741000	-0.76226900	2.63545900
C	-1.11343400	-0.44012700	3.48465300
C	-2.15788000	-0.21031000	2.60736600
C	-0.80878300	3.01822900	-0.80697500
C	-2.10861400	3.45803200	-1.07706100
C	-2.88783900	2.34095600	-0.82803100
C	-0.80444400	-1.89860900	-2.68588900
C	-1.52861500	-2.98920400	-2.18556800
C	-2.23700300	-2.44153800	-1.12981500
H	-3.62471800	-0.21089700	0.16449400
C	2.99036800	-0.21396400	-1.05885900
H	3.14239700	-0.81974200	-1.96535800
C	4.12289200	0.67000000	-0.81996500
C	4.13655500	1.54638200	0.28321800
C	5.21979700	0.69529000	-1.70172600
C	5.20133900	2.39990600	0.51089700
H	3.28260700	1.54426000	0.96355900
C	6.27812800	1.57418700	-1.51190300
H	5.22488900	0.02681900	-2.56311300
C	6.26039400	2.40665300	-0.39818500
H	5.21168700	3.06832800	1.36902100
H	7.11335400	1.60641200	-2.20818600
Cl	7.59851700	3.49651300	-0.12981200
Br	0.77704200	3.98643800	-0.94096800
Br	-2.66544000	5.14055400	-1.62880100
Br	-4.73245800	2.20376600	-0.99233500
Br	-1.13303800	-0.34135900	5.33858500
Br	1.70808800	-1.15079100	3.14736900
Br	-3.89524300	0.31019400	3.01284600
Br	-3.40192100	-3.29046900	0.04475400
Br	-1.52738600	-4.75216500	-2.77000300
Br	0.39878800	-1.89145300	-4.11409000
O	3.00006600	-3.22870300	-0.89352400
C	3.51866400	-2.41077400	0.03811600
H	3.10298300	-1.22688600	-0.19296600
H	3.01004900	-2.47676700	1.01818200
C	1.60372000	-3.47749500	-0.73627700
H	1.30630200	-4.14980100	-1.54556400
H	1.41088300	-3.96129000	0.23296300
H	1.03575000	-2.53624800	-0.79188600
C	5.01176200	-2.36888100	0.09034700

H	5.39876400	-1.85147700	-0.80307800
H	5.38083600	-3.40636900	0.02779600
C	5.52118400	-1.70331900	1.37244800
H	4.89984600	-0.80878500	1.56323600
C	6.96235900	-1.25051500	1.19998500
H	7.06238100	-0.52120900	0.38349400
H	7.34680500	-0.78576800	2.11784600
H	7.61101400	-2.10855200	0.96515100
C	5.39599600	-2.64481100	2.56389000
H	4.38037000	-3.04993400	2.68640800
H	6.07370800	-3.50331400	2.44212900
H	5.66487200	-2.13763300	3.50013200

TS-2T

Zero-point correction= 0.401050 (Hartree/Particle)
Thermal correction to Energy= 0.445870
Thermal correction to Enthalpy= 0.446814
Thermal correction to Gibbs Free Energy= 0.311809
Sum of electronic and zero-point Energies= -25028.506624
Sum of electronic and thermal Energies= -25028.461804
Sum of electronic and thermal Enthalpies= -25028.460860
Sum of electronic and thermal Free Energies= -25028.595865

Ag	1.03388600	-0.52466600	-0.11379500
N	-0.78283700	-0.79043600	1.35222000
N	-1.76750100	0.13839000	1.44210400
N	0.44786900	1.80307400	0.59593700
N	-0.82022400	2.19245000	0.30467500
N	-0.78341000	0.44669000	-1.82375300
N	-1.90764900	0.40749200	-1.06256500
B	-1.97591700	1.16592700	0.29783300
C	-0.81560700	-1.48675300	2.47663600
C	-1.82668900	-1.03465000	3.33112700
C	-2.40487100	0.00635000	2.62530300
C	1.23041700	2.82118800	0.28341800
C	0.49867200	3.90155100	-0.22547000
C	-0.80600000	3.44366000	-0.20232100
C	-1.01749700	-0.34639600	-2.85652100
C	-2.28997300	-0.92693300	-2.79711200
C	-2.81590800	-0.41569200	-1.62454800
H	-3.03164900	1.70898600	0.43798500
C	2.88988500	-1.19764700	-0.98927700
H	2.98862400	-1.03267500	-2.07125700

C	4.13929900	-0.94398600	-0.27707300
C	4.23028400	-1.13924900	1.11354100
C	5.25999300	-0.42495100	-0.94748400
C	5.38496500	-0.82592400	1.81159700
H	3.35858000	-1.51544500	1.65123800
C	6.41258400	-0.07038800	-0.25872700
H	5.21294700	-0.27701400	-2.02668800
C	6.46182000	-0.28239000	1.11415000
H	5.44651500	-0.97111700	2.88798700
H	7.26899200	0.35299800	-0.77904700
Cl	7.91487300	0.14504200	1.99175500
Br	3.07852300	2.70387000	0.51573800
Br	1.12415500	5.55838600	-0.78500500
Br	-2.33091000	4.33824900	-0.77479500
Br	-2.29148800	-1.68774700	5.00568700
Br	0.38496800	-2.88134300	2.77942900
Br	-3.83114200	1.06740600	3.16448900
Br	-4.46614500	-0.78686900	-0.85147600
Br	-3.08406900	-2.12171400	-3.97721000
Br	0.26532800	-0.57410200	-4.19483200
C	2.88882000	-3.56101700	-1.44397900
H	2.55592000	-2.38134500	-0.91716300
C	1.82867100	-3.70646300	-2.51565100
H	1.80417100	-2.79054600	-3.12680100
H	2.16923400	-4.50653800	-3.19471700
C	2.70255900	-4.32688900	-0.16258200
H	3.45819100	-4.03579100	0.58007600
H	1.70366500	-4.17437600	0.26580500
H	2.83659900	-5.40192200	-0.36335300
C	4.28679500	-3.60009100	-1.97825500
H	4.39760500	-3.01099800	-2.89816800
H	5.02429900	-3.25732800	-1.24096600
H	4.53170600	-4.64652900	-2.21962600
C	0.43079300	-4.05552400	-2.06112300
H	-0.21954700	-4.17876500	-2.94754900
H	0.42728500	-5.02785700	-1.53155700
O	-0.08122300	-3.05120100	-1.21998500
C	-1.33077400	-3.41385600	-0.69039500
H	-1.68595900	-2.58013100	-0.07505900
H	-2.06586400	-3.61172900	-1.49111700
H	-1.25327600	-4.32036500	-0.06346400

107-P

Zero-point correction= 0.408256 (Hartree/Particle)
Thermal correction to Energy= 0.452970
Thermal correction to Enthalpy= 0.453914
Thermal correction to Gibbs Free Energy= 0.318385
Sum of electronic and zero-point Energies= -25028.595333
Sum of electronic and thermal Energies= -25028.550619
Sum of electronic and thermal Enthalpies= -25028.549675
Sum of electronic and thermal Free Energies= -25028.685204

Ag	1.11609700	0.97626600	-0.59187500
N	0.25091700	-0.22685500	1.28331200
N	-1.09779300	-0.37831400	1.32805900
N	-0.81904500	2.30885500	-0.20461800
N	-1.97684200	1.61619800	-0.00723200
N	-0.50427900	-0.22282500	-1.88773300
N	-1.57210400	-0.63510200	-1.15768100
B	-2.01458300	0.07608400	0.15148200
C	0.72905300	-0.76386500	2.39414400
C	-0.29154700	-1.28447100	3.19082500
C	-1.44123400	-1.01150900	2.46805300
C	-1.14779100	3.58579600	-0.28193100
C	-2.53045400	3.77205500	-0.12879900
C	-3.01344800	2.49109700	0.04236200
C	-0.36080300	-1.08882400	-2.87516900
C	-1.33150200	-2.09962800	-2.81604200
C	-2.07621900	-1.76735400	-1.70035500
H	-3.13232600	-0.24834900	0.40176600
C	3.97579000	-0.29973800	-1.53020900
H	3.94314700	-0.19520400	-2.62533300
C	5.03468000	0.56127000	-0.91582800
C	4.75899300	1.31040500	0.22792800
C	6.33065400	0.59666100	-1.44126800
C	5.73510000	2.09557800	0.84209600
H	3.74455900	1.29634000	0.63618200
C	7.32230200	1.36628800	-0.84887100
H	6.56610400	0.01814300	-2.33707500
C	7.01243800	2.10750600	0.28712400
H	5.50690900	2.68060100	1.72868100
H	8.32598200	1.39625200	-1.26297900
Cl	8.25774000	3.08507000	1.04081300
Br	0.13852800	4.90471800	-0.55790800
Br	-3.47808900	5.36699000	-0.15274000
Br	-4.78822300	2.00385700	0.31647900
Br	-0.14683600	-2.18380400	4.80407200

Br	2.56412100	-0.78649600	2.72158200
Br	-3.18394300	-1.41807600	2.95880700
Br	-3.51318400	-2.70779900	-0.99508600
Br	-1.53109400	-3.57238700	-3.92410800
Br	1.01248900	-0.91412000	-4.11920200
C	1.14134200	-4.10838500	1.10105500
H	1.31433200	-3.28806200	1.82208100
C	-0.28130900	-3.97150900	0.57619900
H	-0.47842200	-2.95953000	0.18431000
H	-0.47147000	-4.68753700	-0.23753400
H	-1.01973000	-4.16387600	1.36800100
C	1.31564300	-5.42791900	1.83250600
H	2.33850000	-5.54775800	2.22428200
H	0.62396200	-5.51083000	2.68146500
H	1.11872900	-6.27722000	1.15860600
C	2.19458100	-3.97165600	0.00268100
H	2.19083700	-4.87076300	-0.63503000
H	3.19369000	-3.92842400	0.47532300
C	2.02151500	-2.75456900	-0.89064500
H	1.84412200	-1.83590200	-0.28926500
H	1.14584400	-2.88148900	-1.54332700
O	3.13699700	-2.57007800	-1.74485700
C	4.15559700	-1.77679300	-1.18579100
H	2.98879800	0.03022200	-1.14339800
H	4.20877500	-1.89714400	-0.08624000
H	5.11826100	-2.12717400	-1.59032200

107-T

Zero-point correction=	0.407096 (Hartree/Particle)
Thermal correction to Energy=	0.450319
Thermal correction to Enthalpy=	0.451264
Thermal correction to Gibbs Free Energy=	0.321198
Sum of electronic and zero-point Energies=	-25028.604062
Sum of electronic and thermal Energies=	-25028.560838
Sum of electronic and thermal Enthalpies=	-25028.559894
Sum of electronic and thermal Free Energies=	-25028.689960

Ag	0.00834200	1.50412700	0.37095200
N	0.34311600	-0.78483900	1.20670400
N	-0.57261300	-1.66670200	0.72250900
N	-2.11329800	0.78317400	-0.04412600
N	-2.31315700	-0.41818000	-0.64237500
N	0.18288100	0.25196900	-1.99833400

N	-0.03993800	-1.03841300	-1.63845900
B	-1.18919900	-1.47981500	-0.69553800
C	0.74150000	-1.27089100	2.37313900
C	0.09899000	-2.47555100	2.68051400
C	-0.73354100	-2.68324200	1.59535800
C	-3.25590100	1.44589400	-0.11274800
C	-4.24184700	0.68992400	-0.75486100
C	-3.59283900	-0.49232500	-1.06982900
C	1.32986500	0.24285100	-2.66512100
C	1.89914300	-1.03344600	-2.72813700
C	0.97863400	-1.81910100	-2.06193800
H	-1.64605200	-2.51916900	-1.06120900
C	2.92636600	1.82913400	0.34056000
H	2.60252700	1.46265600	-0.64467100
C	1.96933600	2.90444400	0.79306500
C	1.80323400	3.22178000	2.15822200
C	1.22986800	3.66359600	-0.14302500
C	0.97241100	4.25351600	2.57005100
H	2.34714600	2.64512000	2.90518700
C	0.38663300	4.70498400	0.26153800
H	1.36488000	3.47541600	-1.20966800
C	0.27279700	4.98667800	1.61288000
H	0.86022200	4.48601200	3.62694500
H	-0.16935500	5.28075700	-0.47562200
Cl	-0.79445200	6.27195900	2.13334000
Br	-3.39384500	3.16163400	0.59825000
Br	-6.00410000	1.15782900	-1.09727700
Br	-4.32011600	-1.97420700	-1.92042500
Br	0.31320500	-3.54675800	4.18112700
Br	2.03452400	-0.41327100	3.40997000
Br	-1.91145200	-4.09473200	1.32876400
Br	1.04755700	-3.65584600	-1.77697800
Br	3.51588800	-1.54700600	-3.47642400
Br	2.01106000	1.79538500	-3.45528700
C	4.42190600	2.19582600	0.21852000
H	2.84425100	0.96589900	1.01440400
C	5.13742000	1.03797300	-0.50538200
H	4.74890400	0.96850800	-1.53669800
H	6.20396100	1.30275900	-0.59179300
C	5.03663400	2.44272200	1.59376900
H	4.59578200	3.32890600	2.07345300
H	4.89249300	1.59326300	2.27735600
H	6.11847500	2.62424200	1.50917000
C	4.59946400	3.45110100	-0.63151600

H	4.16223600	3.32345000	-1.63327300
H	4.12799400	4.32783500	-0.16390100
H	5.66671000	3.68180200	-0.76346200
C	5.06297700	-0.34965100	0.10212900
H	5.79845200	-1.00431200	-0.40578200
H	5.34301900	-0.34045900	1.17489200
O	3.77226000	-0.89064700	-0.04770400
C	3.69023100	-2.19781300	0.43939900
H	2.64310800	-2.52141400	0.37446500
H	4.30773800	-2.89440000	-0.15718000
H	4.01517900	-2.26534000	1.49402600

107-S

Zero-point correction=	0.407978 (Hartree/Particle)
Thermal correction to Energy=	0.452785
Thermal correction to Enthalpy=	0.453730
Thermal correction to Gibbs Free Energy=	0.318583
Sum of electronic and zero-point Energies=	-25028.597518
Sum of electronic and thermal Energies=	-25028.552711
Sum of electronic and thermal Enthalpies=	-25028.551767
Sum of electronic and thermal Free Energies=	-25028.686914

Ag	1.01183600	0.67969700	0.01517300
N	-0.33151800	-0.97493400	1.33425100
N	-1.65497300	-0.91787500	1.02665700
N	-1.01498400	1.78387200	-0.09076800
N	-2.10960600	1.07713100	-0.47304600
N	-0.05192300	-0.55198100	-1.85516800
N	-1.21547700	-1.11686300	-1.44083700
B	-2.13997900	-0.46751200	-0.38004700
C	-0.26653800	-1.40067900	2.58726100
C	-1.53616400	-1.62871500	3.12909200
C	-2.39026400	-1.30160500	2.09135400
C	-1.31853800	3.06529900	-0.22156300
C	-2.62576400	3.23189000	-0.68922100
C	-3.08961500	1.93591100	-0.83380300
C	0.49748900	-1.41865700	-2.69396900
C	-0.28177000	-2.57164700	-2.83930500
C	-1.36219800	-2.33284400	-2.00879100
H	-3.25788200	-0.84066900	-0.55387400
C	3.55090800	-0.94315300	0.29180900
H	3.32232900	-1.31996900	-0.71810100
C	3.36523500	0.55081500	0.35278500

C	3.29253800	1.22108100	1.59626800
C	3.44610700	1.34706100	-0.81160200
C	3.36043100	2.60701700	1.67843400
H	3.22303100	0.63310100	2.51188100
C	3.51281000	2.73870800	-0.74024100
H	3.51349900	0.85807200	-1.78355900
C	3.48953000	3.34799700	0.50696700
H	3.31668000	3.10658200	2.64391400
H	3.58590300	3.33541800	-1.64697100
Cl	3.57919500	5.09193900	0.60759100
Br	-0.07294800	4.38494100	0.19380300
Br	-3.53028500	4.81620700	-1.02998300
Br	-4.77249500	1.41633500	-1.42293600
Br	-1.97254900	-2.23980300	4.82594300
Br	1.36573500	-1.66869200	3.45652800
Br	-4.24759900	-1.34722000	2.10332100
Br	-2.79237900	-3.46643800	-1.65917500
Br	0.03923600	-4.06142300	-3.89737200
Br	2.11131400	-1.04716900	-3.55699200
O	5.08379900	-2.79667300	0.54042400
C	4.98099000	-1.39218400	0.64062600
H	2.84899900	-1.42636600	0.98669500
H	5.18793000	-1.09495200	1.69140300
C	4.36320000	-3.51371100	1.50699200
H	4.73580600	-4.54411500	1.49502400
H	4.51881800	-3.09685300	2.51854600
H	3.28009200	-3.54233100	1.30306800
C	6.03345400	-0.79665000	-0.28857400
H	5.58946800	-0.64583200	-1.28810600
H	6.82250800	-1.55458500	-0.41843800
C	6.68786100	0.49975300	0.20124700
H	5.90515000	1.18438100	0.57231000
C	7.40637300	1.18731000	-0.95032000
H	6.70894500	1.46551500	-1.75369700
H	7.91894500	2.10061600	-0.61816900
H	8.16560600	0.51882500	-1.38529800
C	7.65878500	0.22780300	1.34354300
H	7.18189100	-0.29434800	2.18491700
H	8.49485400	-0.39893000	0.99710400
H	8.08347900	1.16261100	1.73468400

8. NMR Spectra of Products

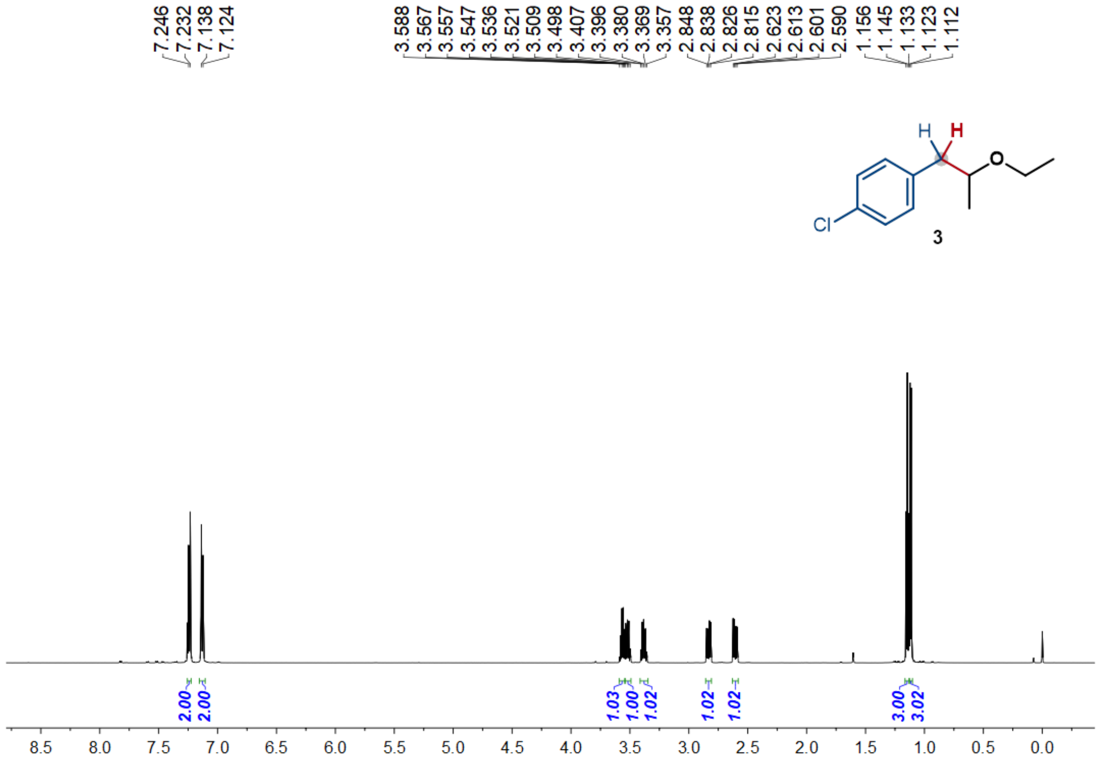

Supplementary Fig. 14 ^{1}H NMR (600 MHz, $CDCl_3$) spectrum of compound **3**

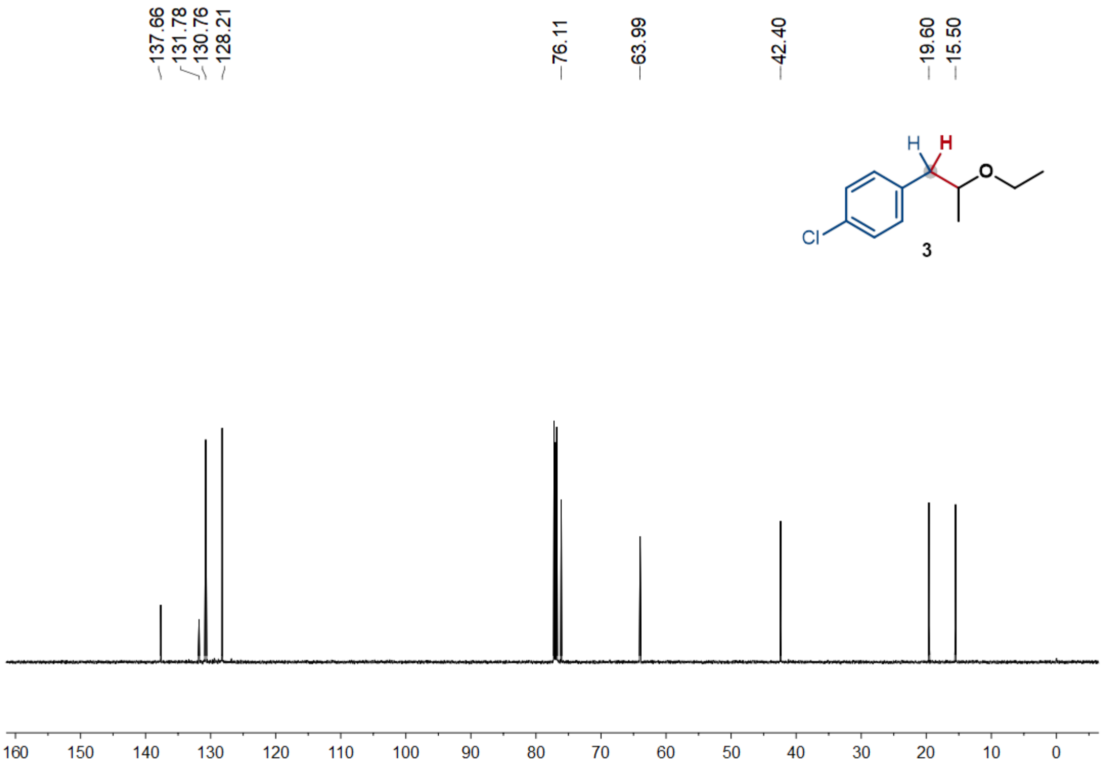

Supplementary Fig. 15 ^{13}C NMR (151 MHz, $CDCl_3$) spectrum of compound **3**

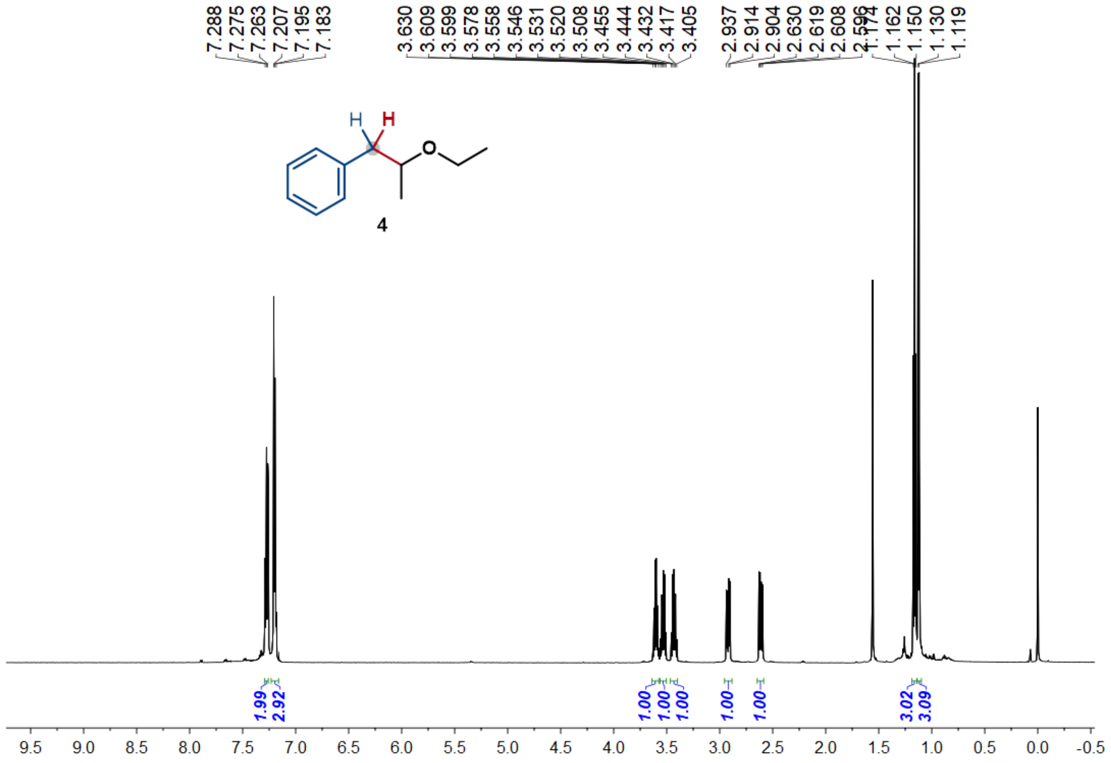

Supplementary Fig. 16 ^{1}H NMR (600 MHz, $CDCl_3$) spectrum of compound **4**

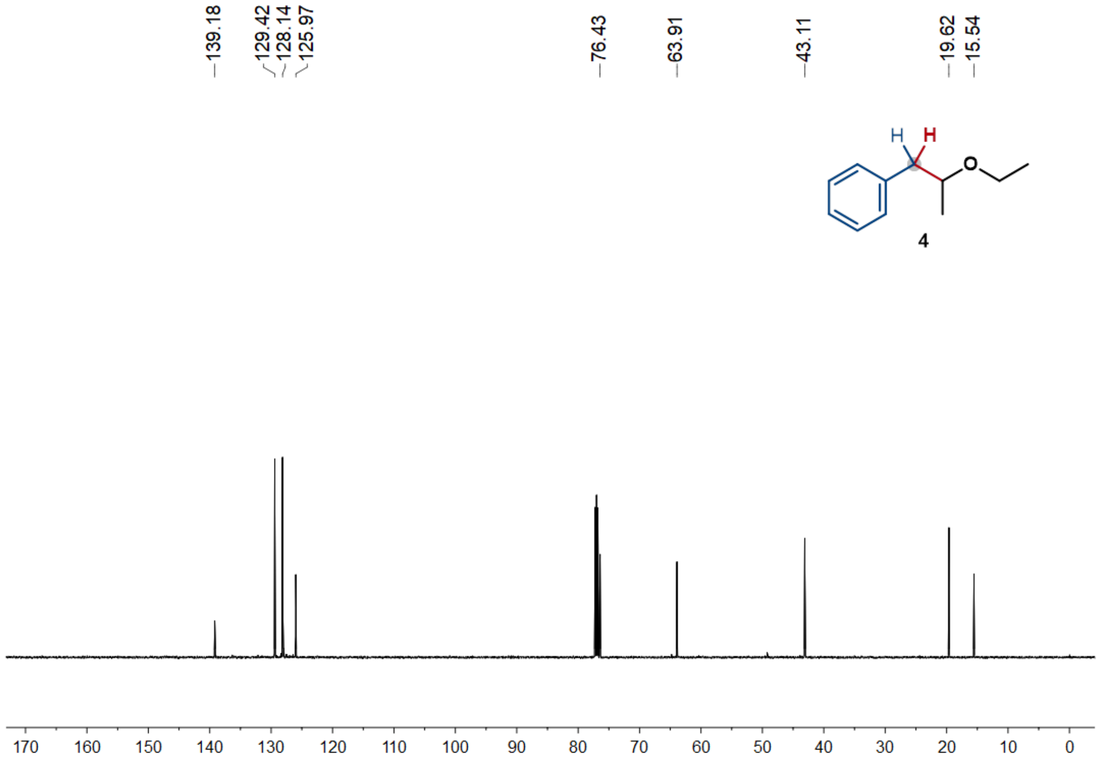

Supplementary Fig. 17 ^{13}**C NMR** (151 MHz, $CDCl_3$) spectrum of compound **4**

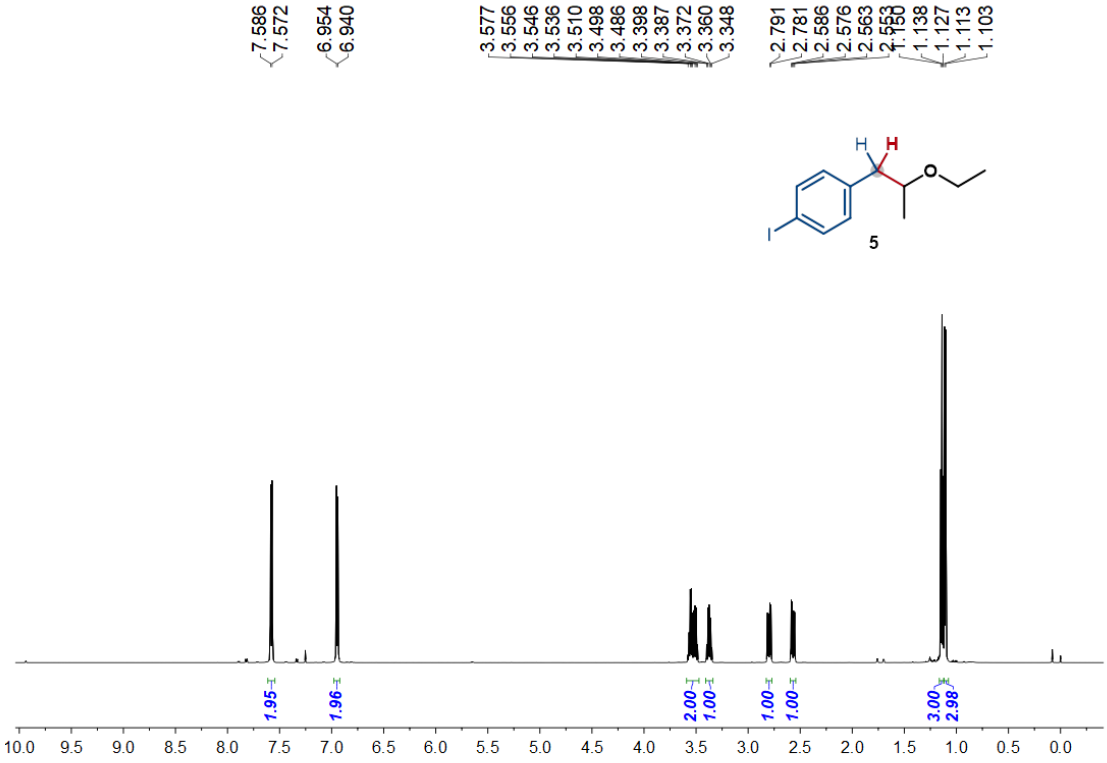

Supplementary Fig. 18 ^{1}H NMR (600 MHz, $CDCl_3$) spectrum of compound **5**

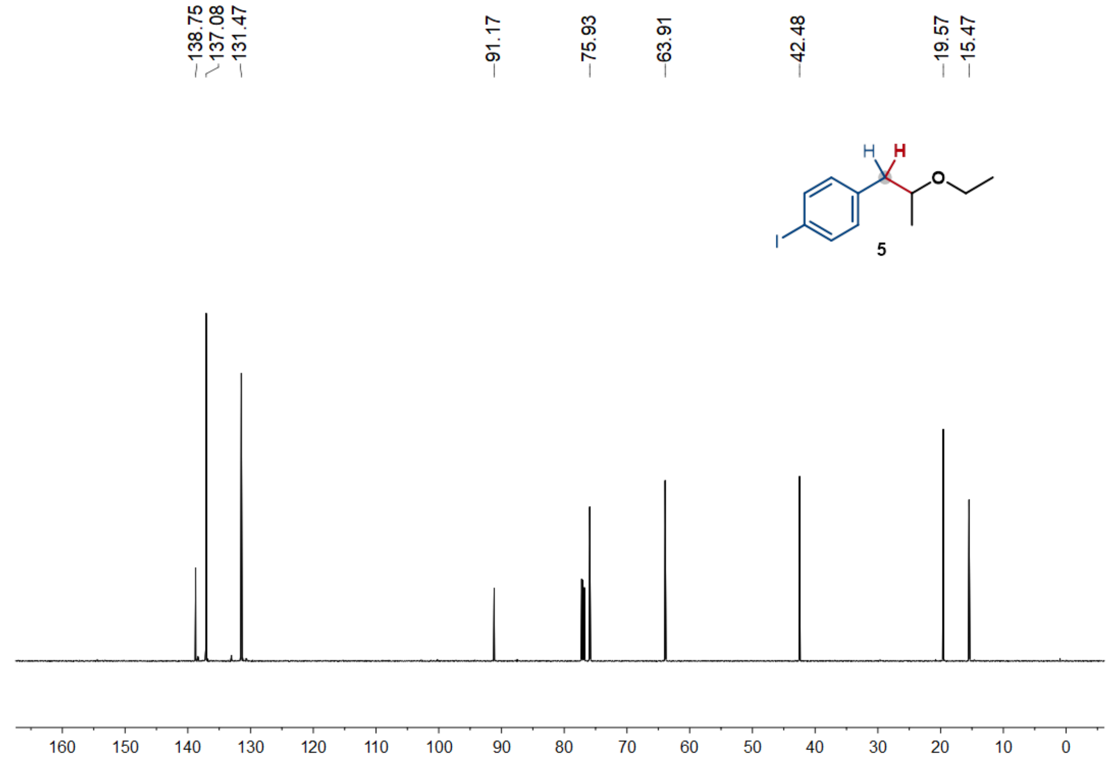

Supplementary Fig. 19 ^{13}C NMR (151 MHz, $CDCl_3$) spectrum of compound **5**

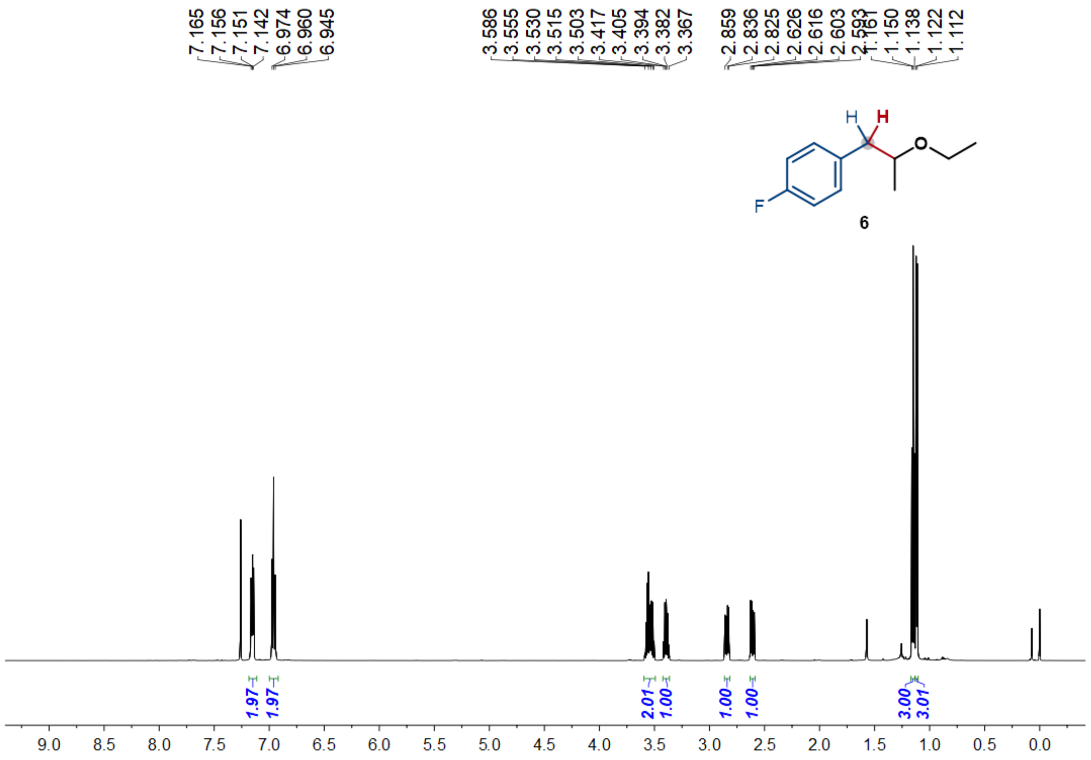

Supplementary Fig. 20 ^{1}**H NMR** (600 MHz, $CDCl_3$) spectrum of compound **6**

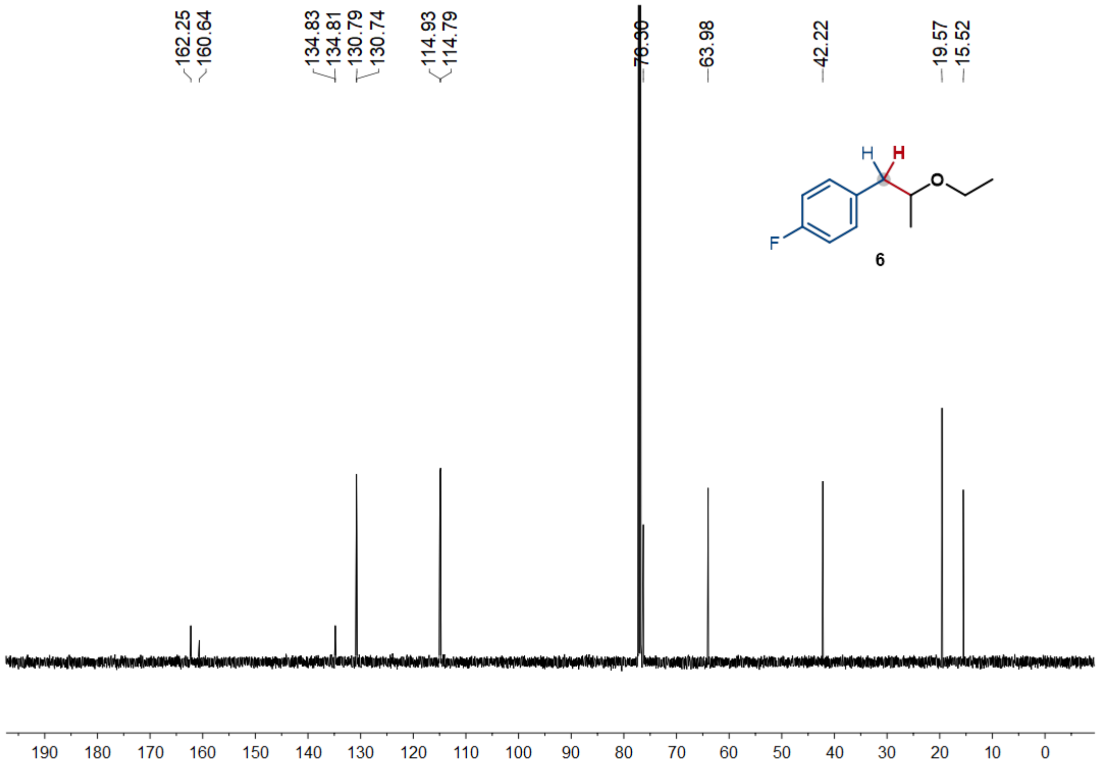

Supplementary Fig. 21 ^{13}**C NMR** (151 MHz, $CDCl_3$) spectrum of compound **6**

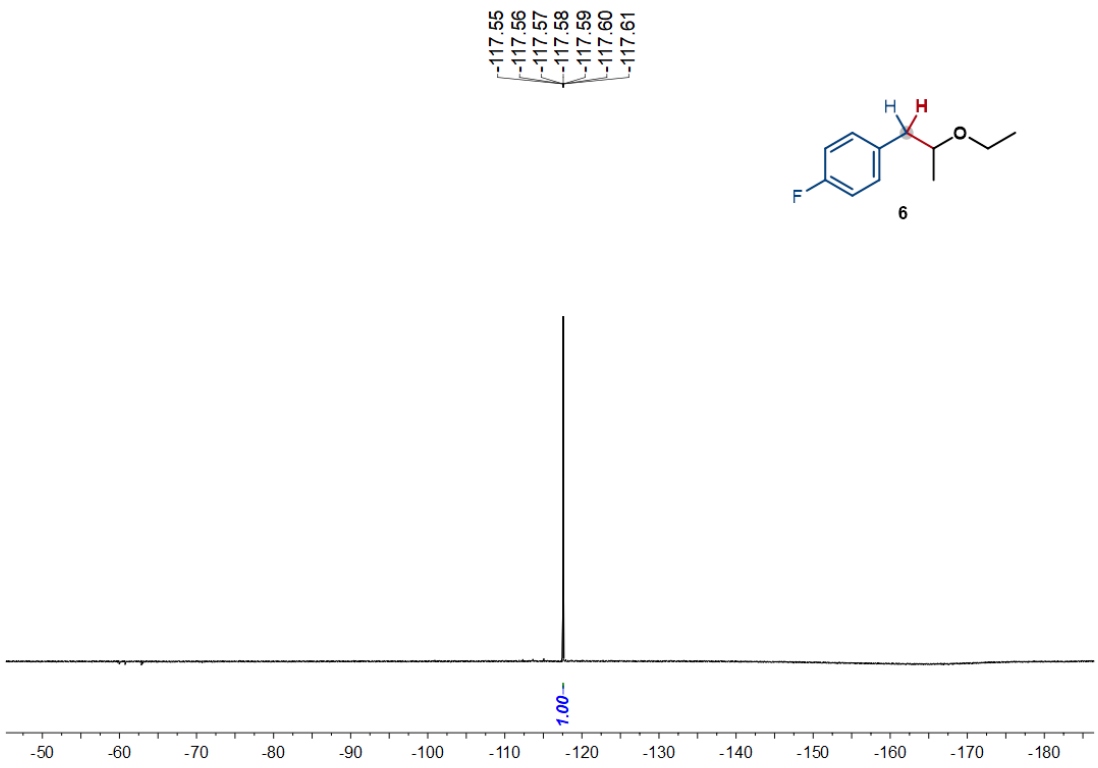

Supplementary Fig. 22 ^{19}**F NMR** (471 MHz, $CDCl_3$) spectrum of compound **6**

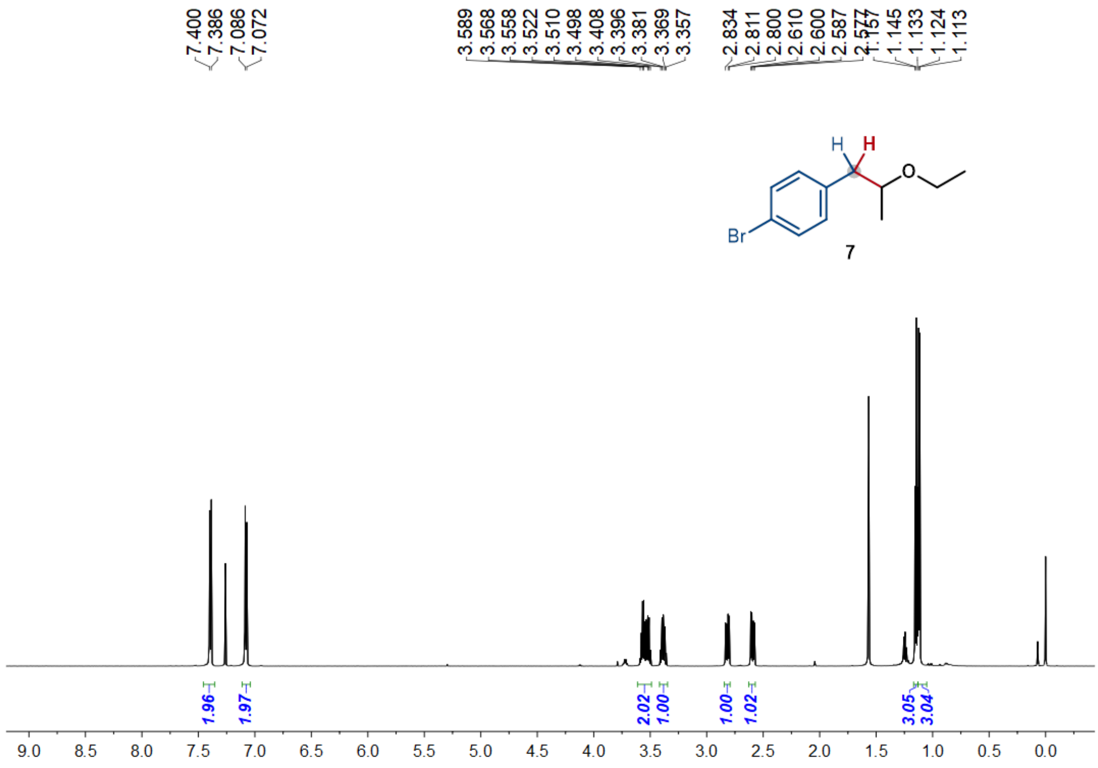

Supplementary Fig. 23 ^{1}**H NMR** (600 MHz, $CDCl_3$) spectrum of compound **7**

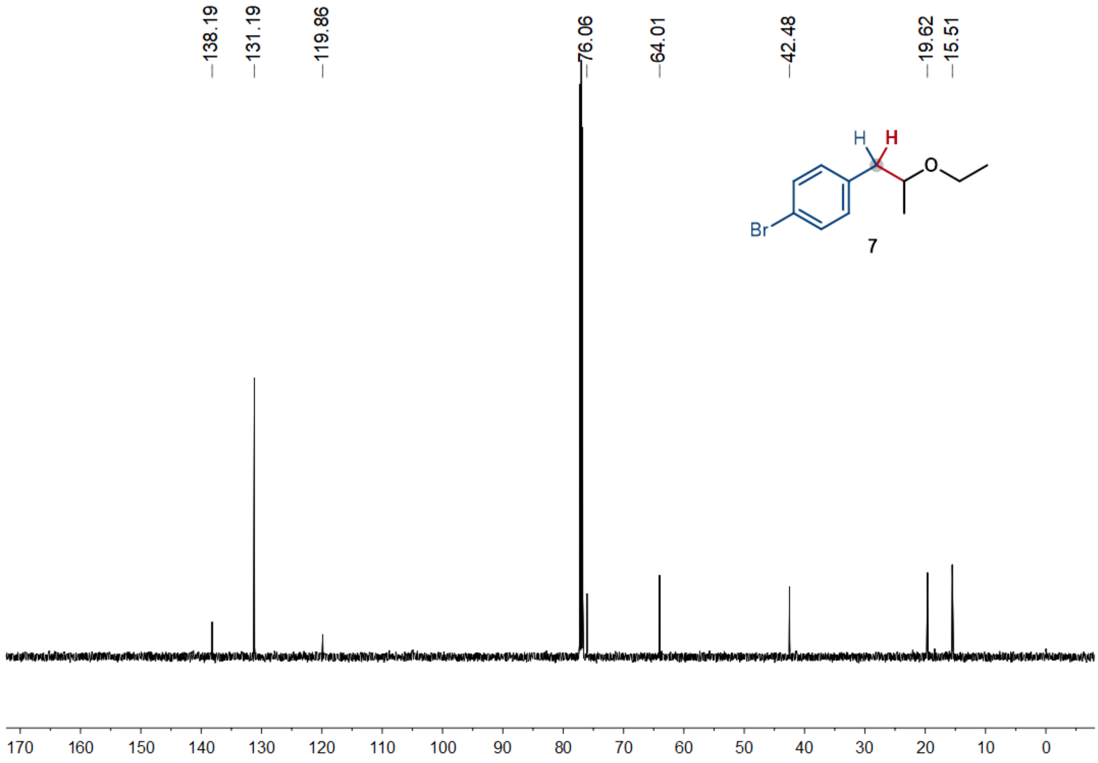

Supplementary Fig. 24 ^{13}C NMR (151 MHz, $CDCl_3$) spectrum of compound **7**

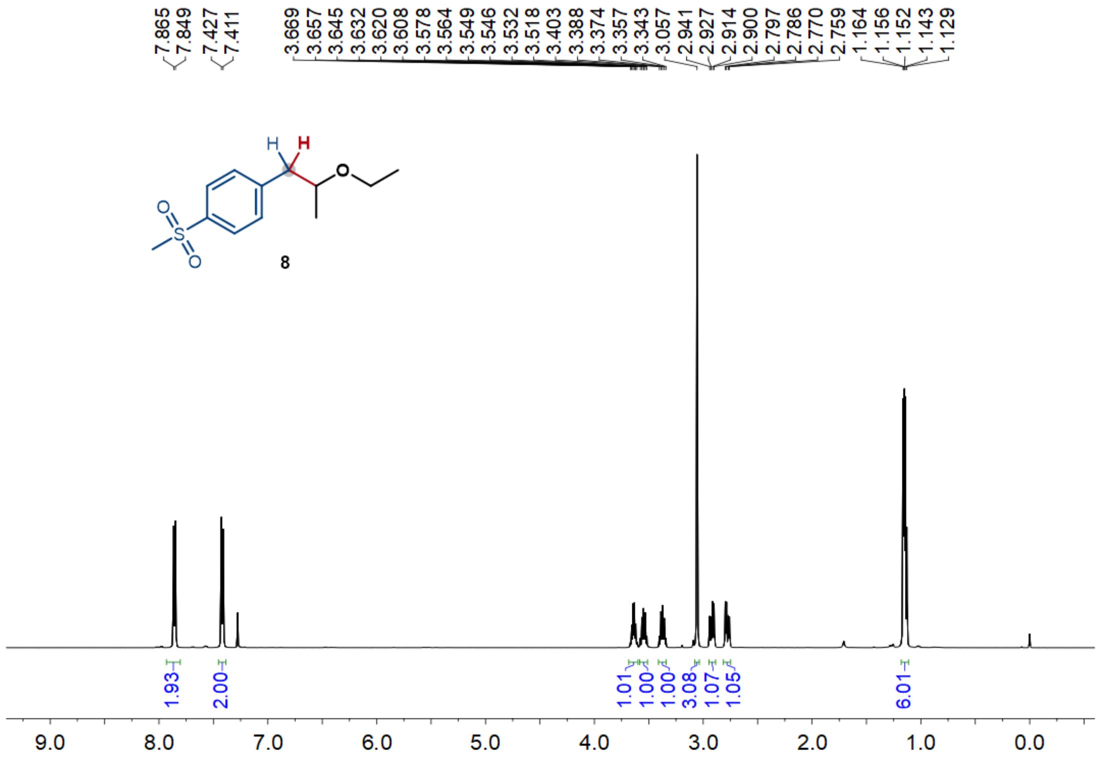

Supplementary Fig. 25 ^{1}H NMR (500 MHz, $CDCl_3$) spectrum of compound **8**

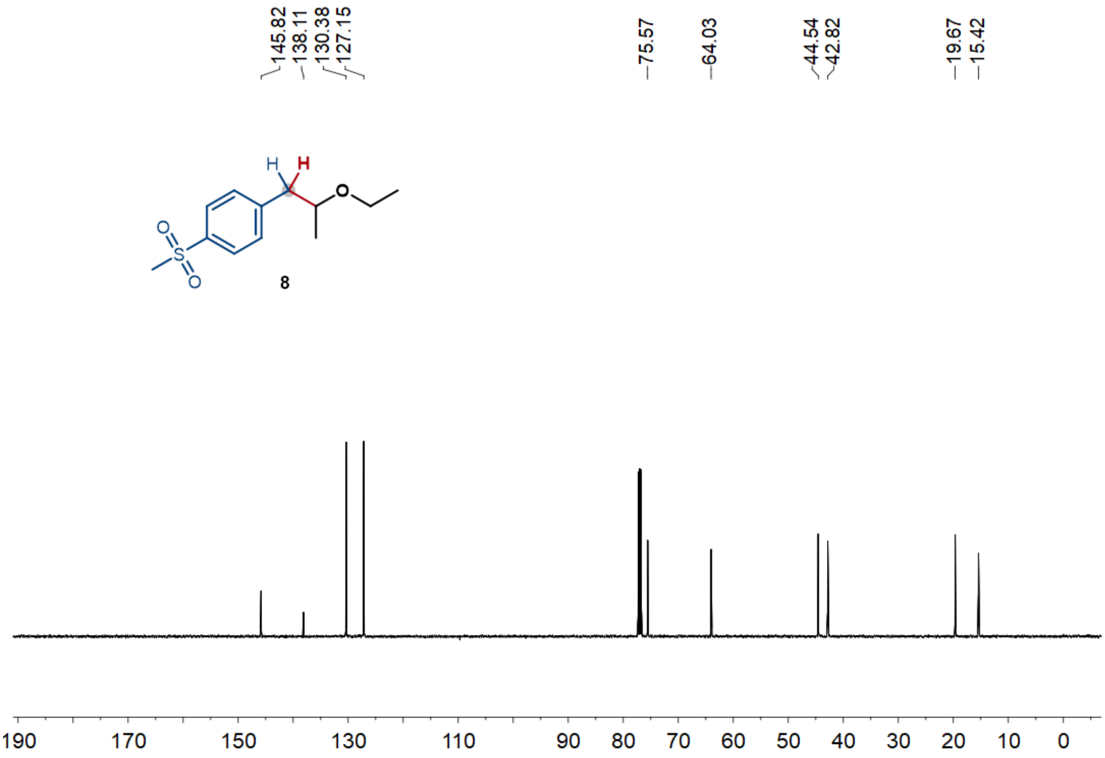

Supplementary Fig. 26 ^{13}C NMR (126 MHz, $CDCl_3$) spectrum of compound **8**

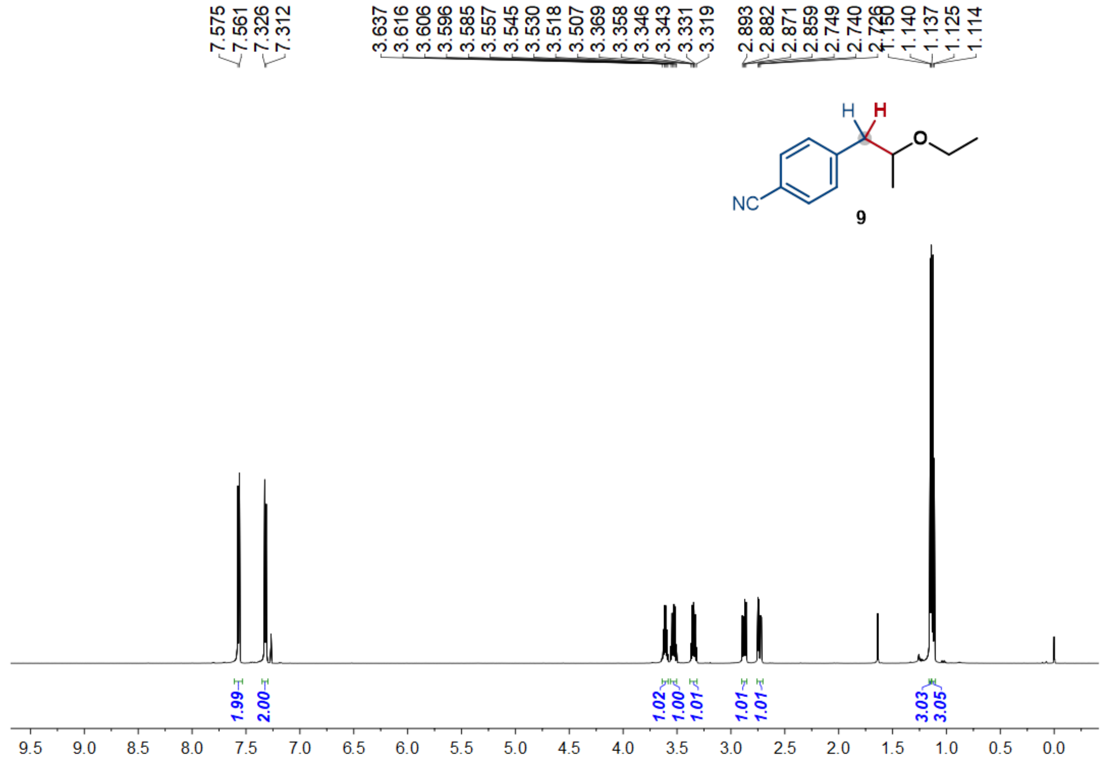

Supplementary Fig. 27 ^{1}H NMR (600 MHz, $CDCl_3$) spectrum of compound **9**

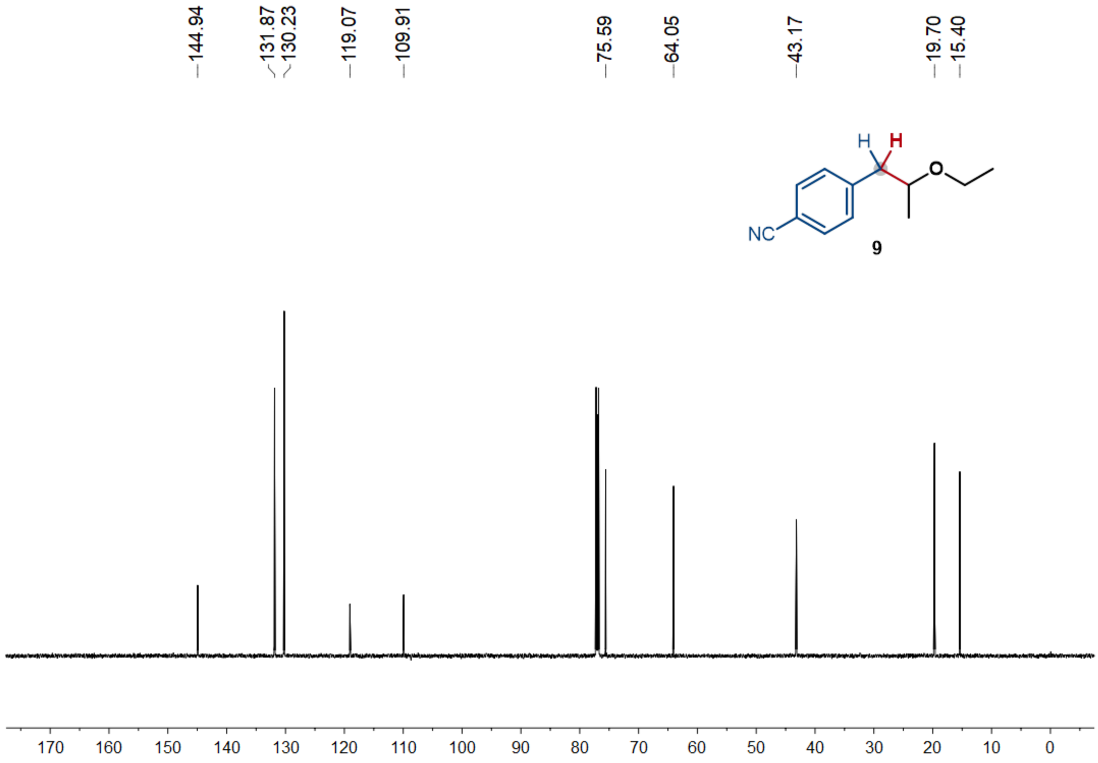

Supplementary Fig. 28 ^{13}**C NMR** (151 MHz, $CDCl_3$) spectrum of compound **9**

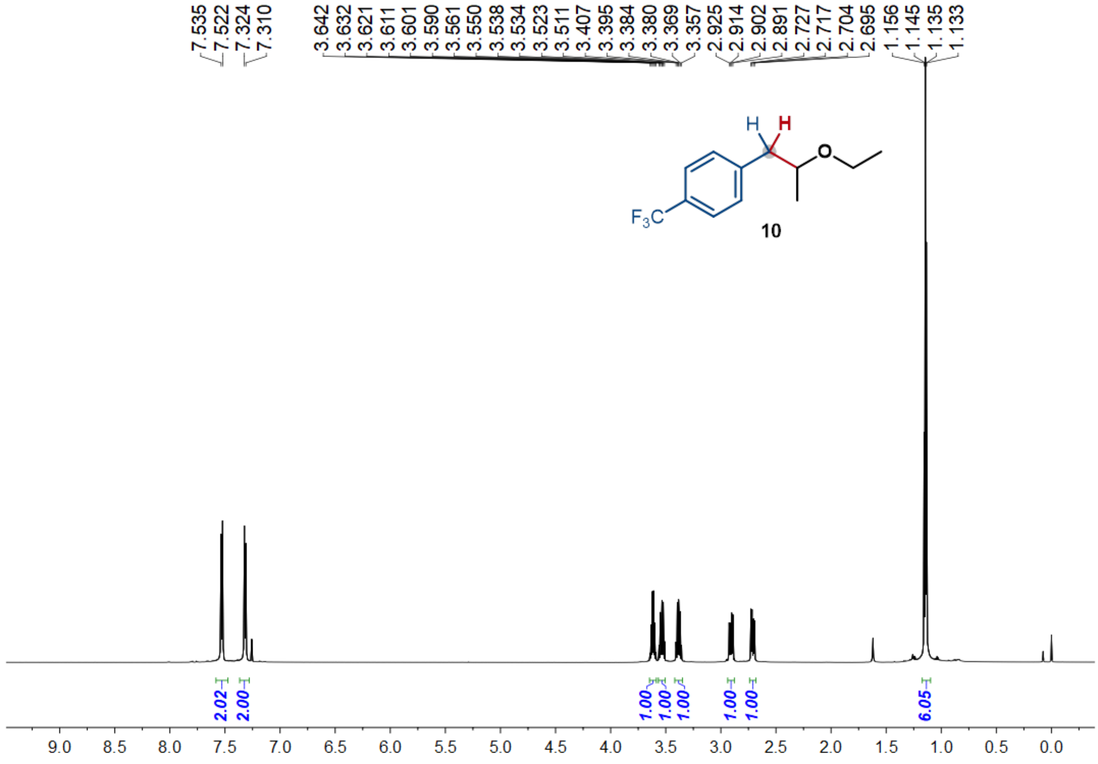

Supplementary Fig. 29 ^{1}**H NMR** (600 MHz, $CDCl_3$) spectrum of compound **10**

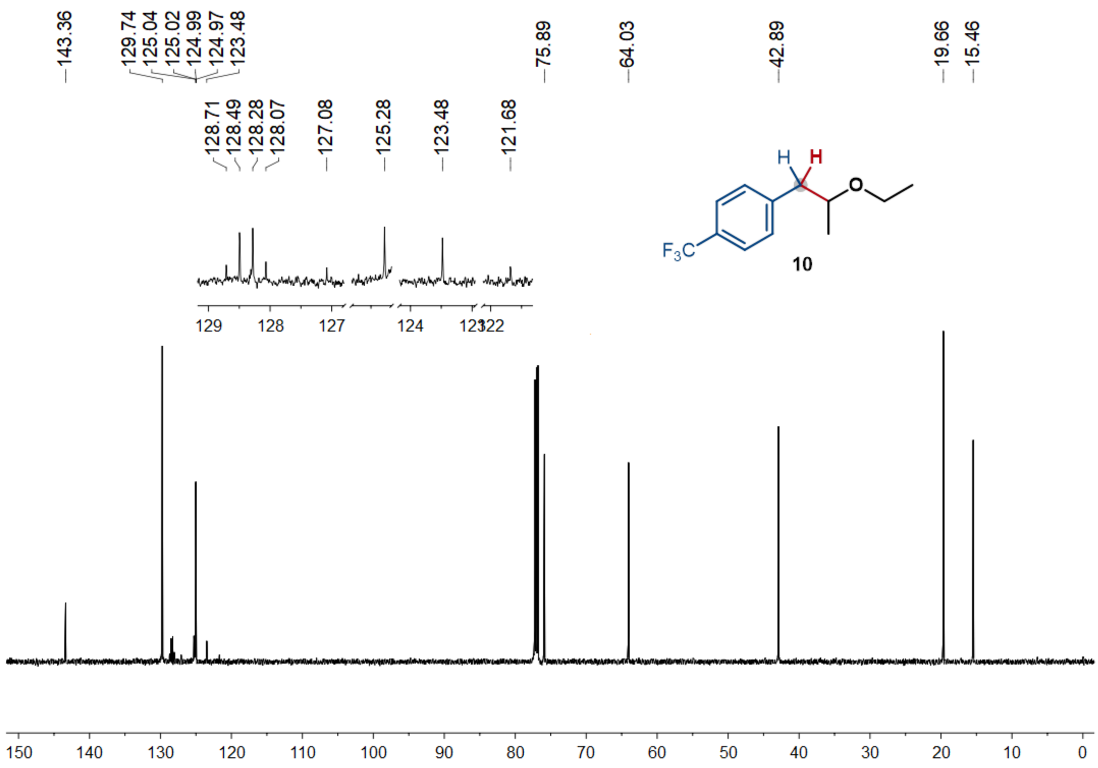

Supplementary Fig. 30 ^{13}**C NMR** (151 MHz, $CDCl_3$) spectrum of compound **10**

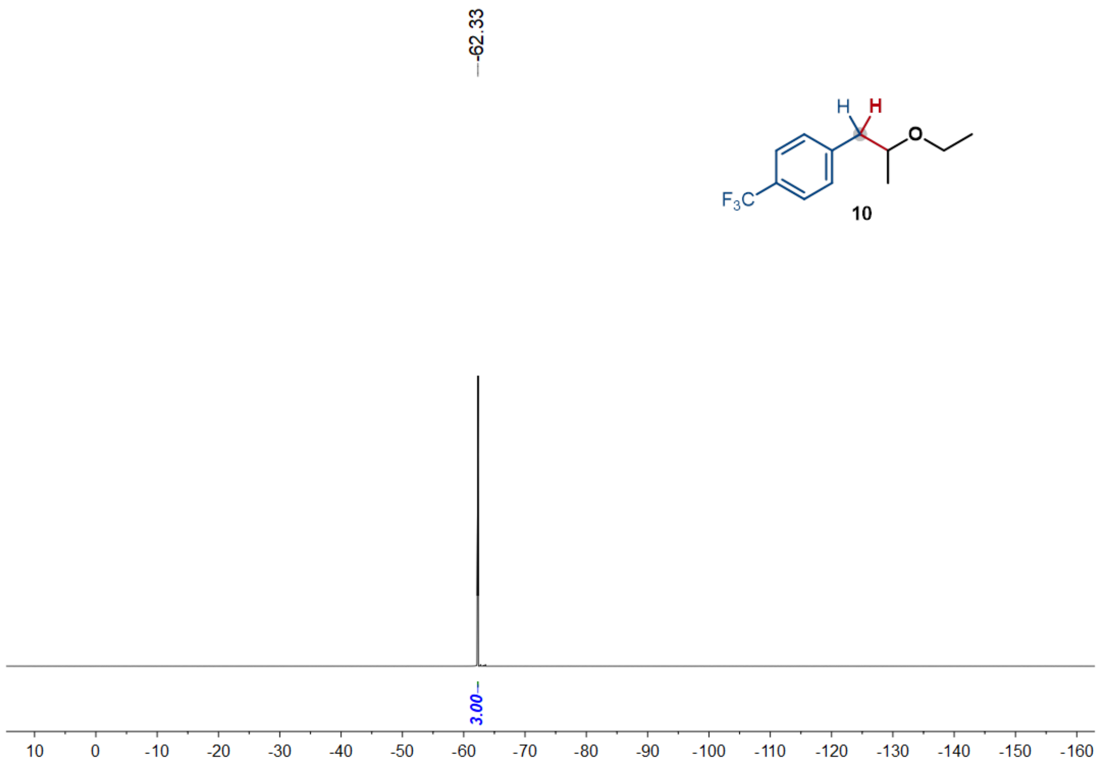

Supplementary Fig. 31 ^{19}**F NMR** (471 MHz, $CDCl_3$) spectrum of compound

10

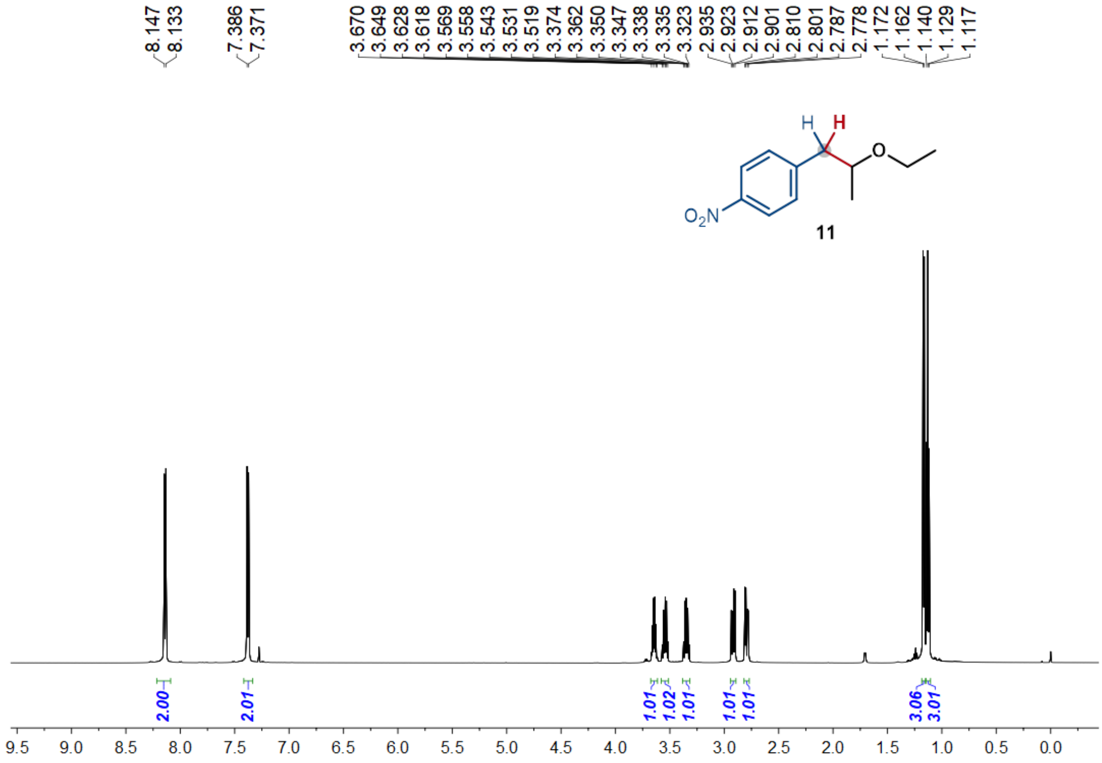

Supplementary Fig. 32 ^{1}H NMR (600 MHz, $CDCl_3$) spectrum of compound **11**

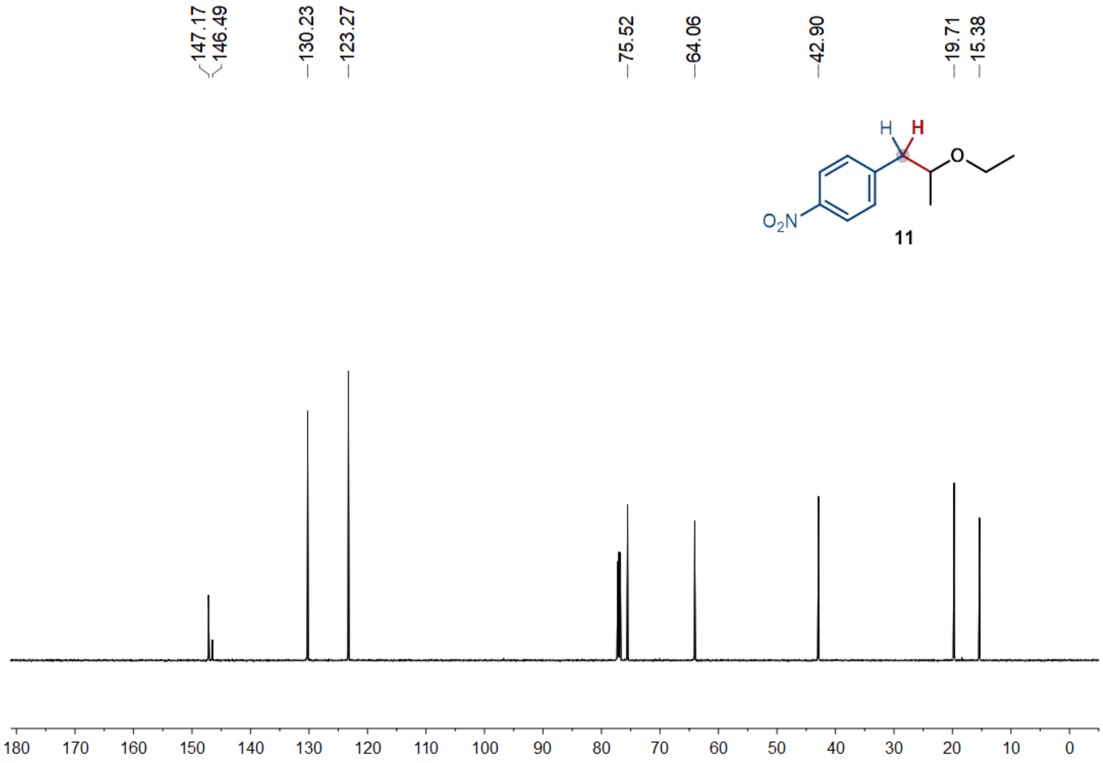

Supplementary Fig. 33 ^{13}C NMR (151 MHz, $CDCl_3$) spectrum of compound **11**

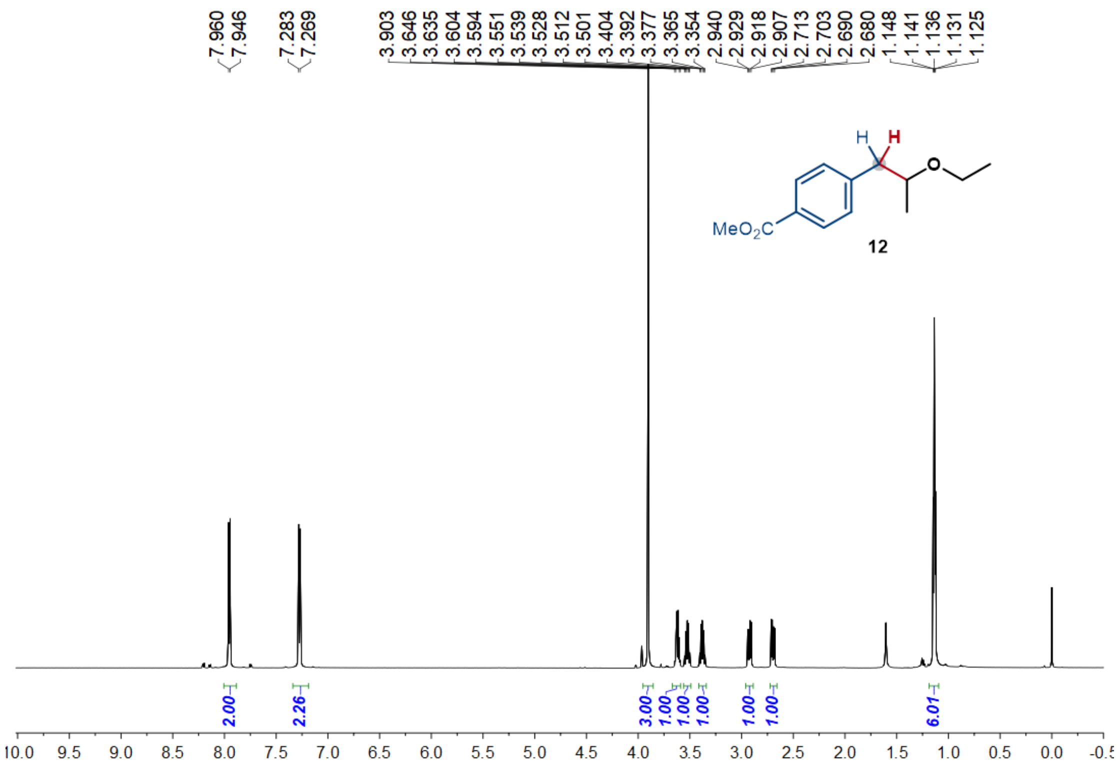

Supplementary Fig. 34 ^{1}**H NMR** (600 MHz, $CDCl_3$) spectrum of compound **12**

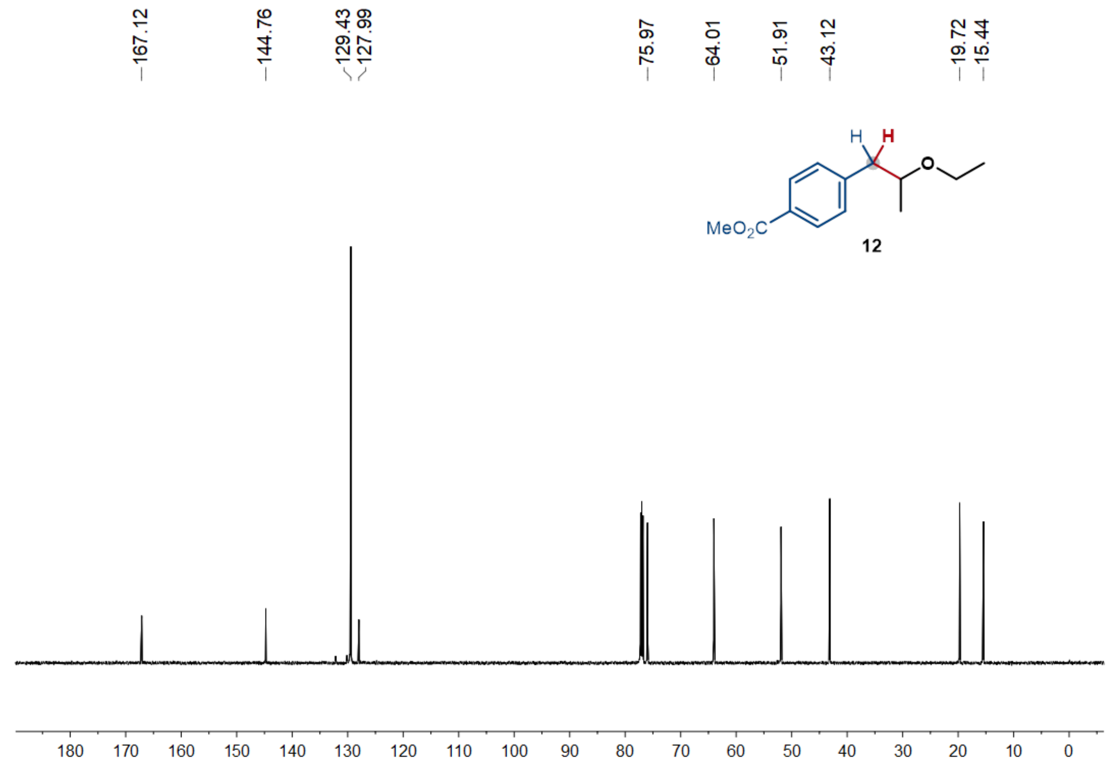

Supplementary Fig. 35 ^{13}**C NMR** (151 MHz, $CDCl_3$) spectrum of compound **12**

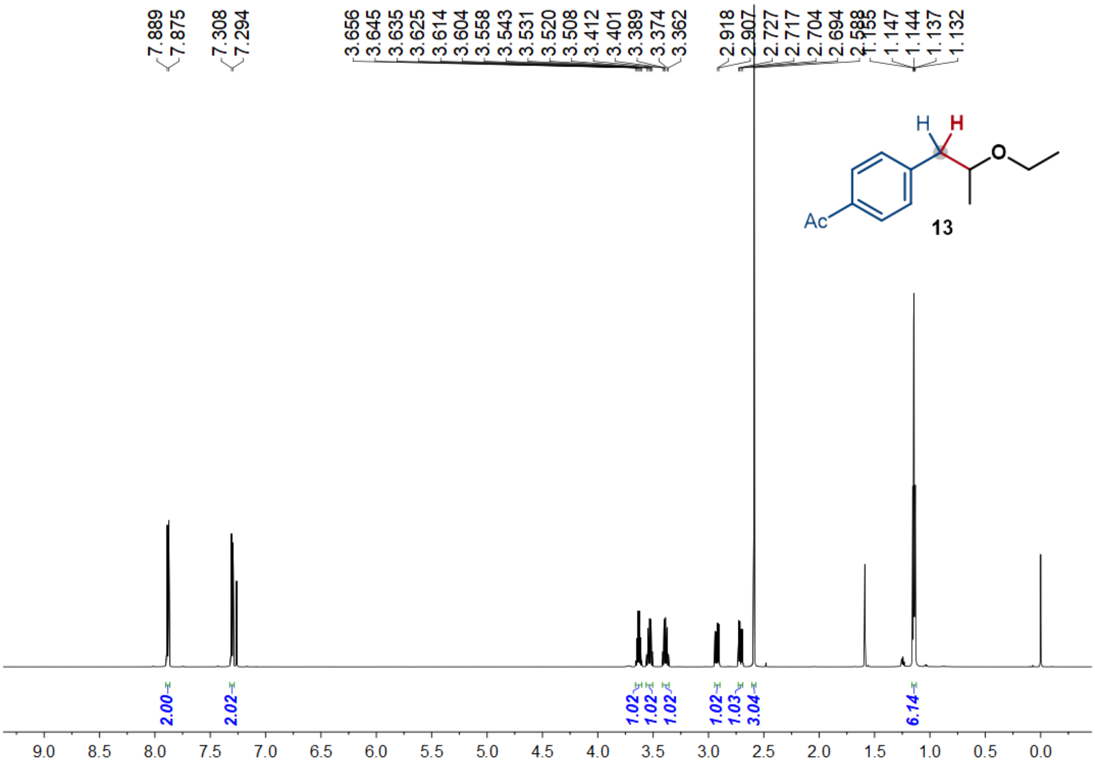

Supplementary Fig. 36 ^{1}**H NMR** (600 MHz, $CDCl_3$) spectrum of compound **13**

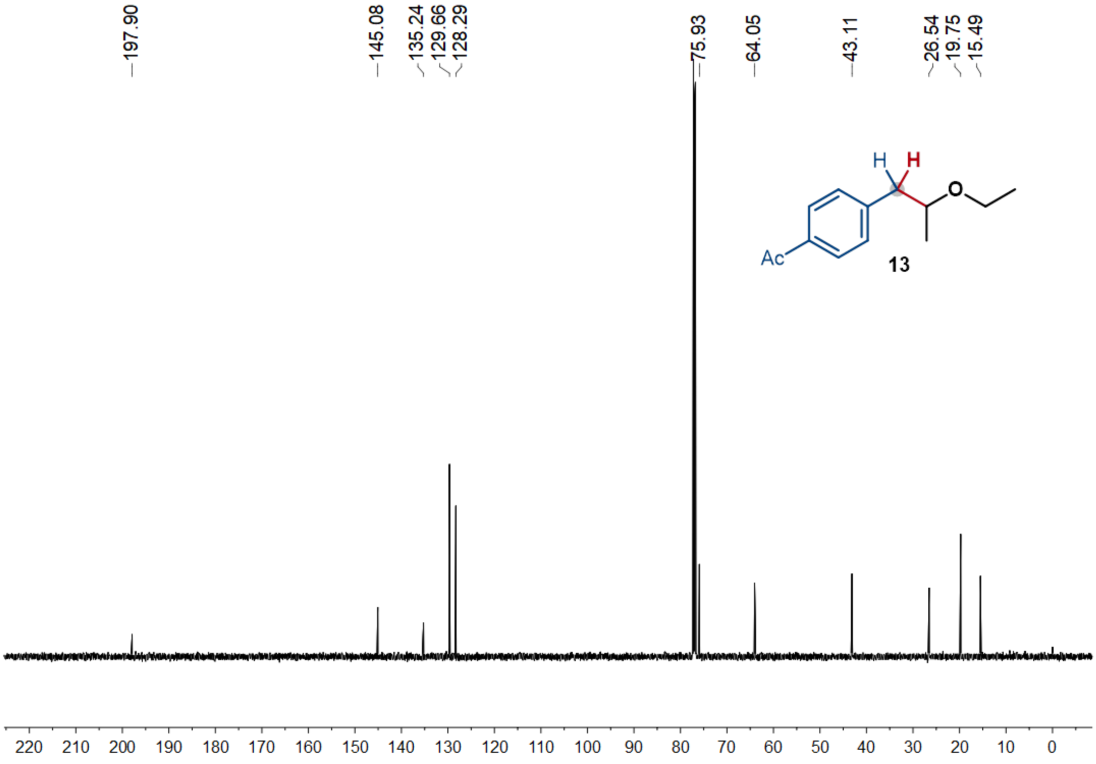

Supplementary Fig. 37 ^{13}**C NMR** (151 MHz, $CDCl_3$) spectrum of compound **13**

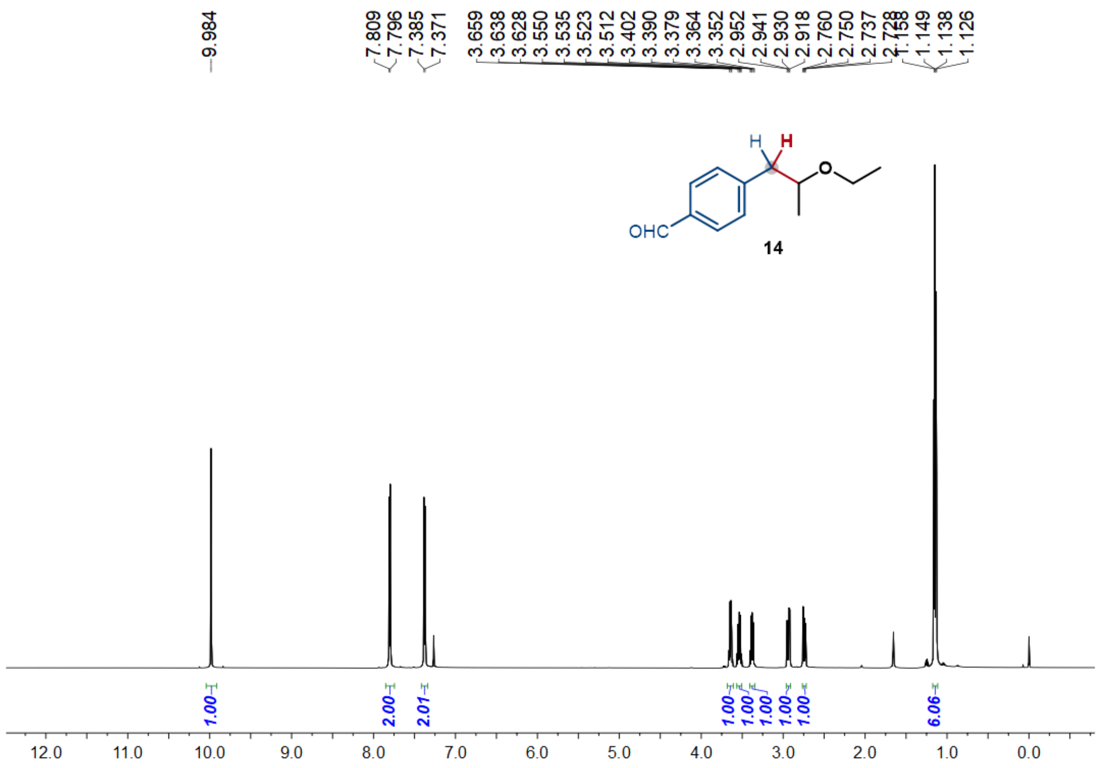

Supplementary Fig. 38 ^{1}**H NMR** (600 MHz, $CDCl_3$) spectrum of compound **14**

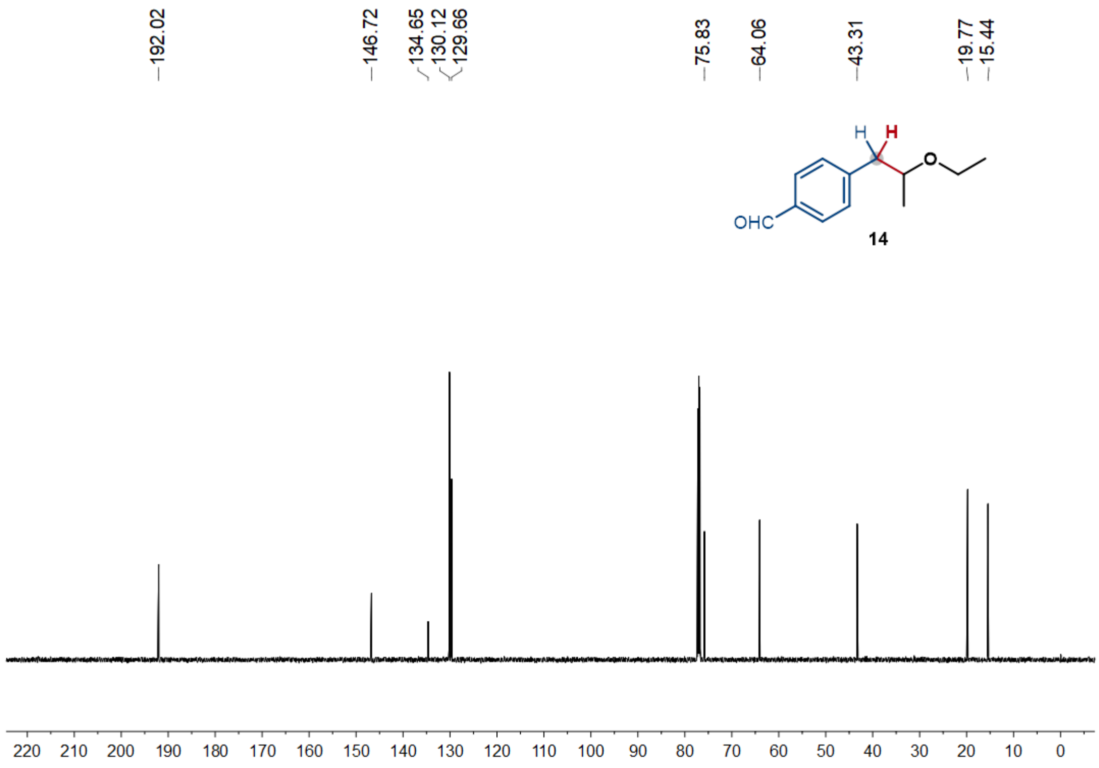

Supplementary Fig. 39 ^{13}**C NMR** (151 MHz, $CDCl_3$) spectrum of compound **14**

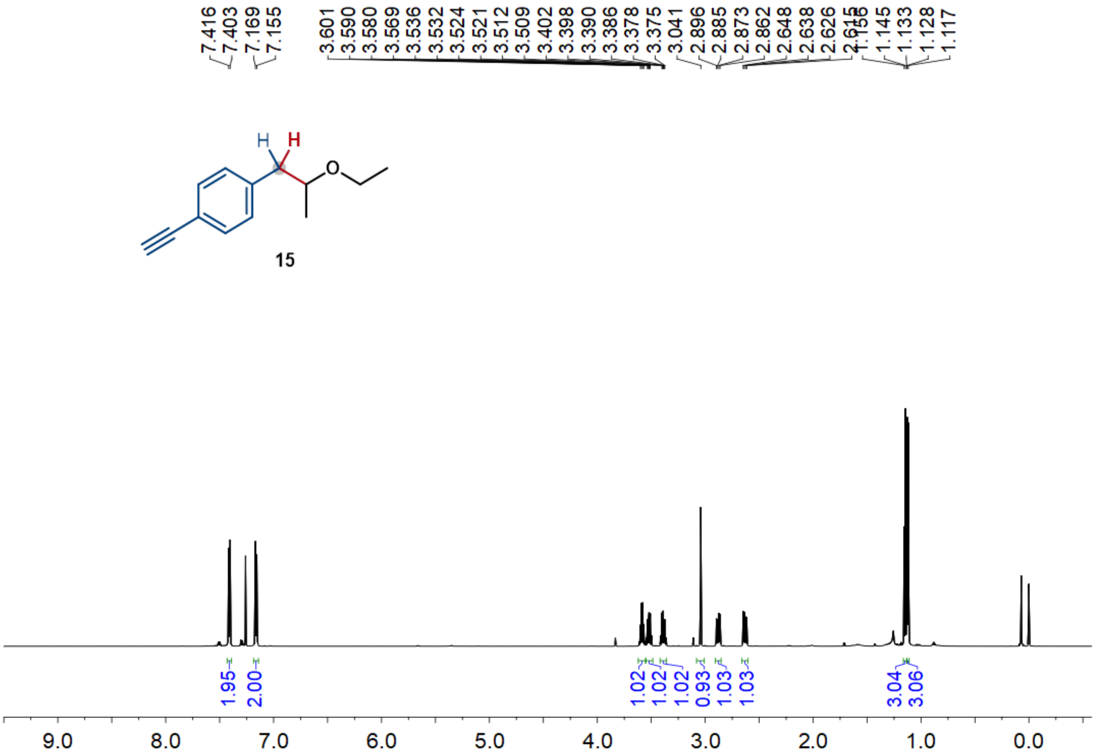

Supplementary Fig. 40 ^{1}H NMR (600 MHz, $CDCl_3$) spectrum of compound **15**

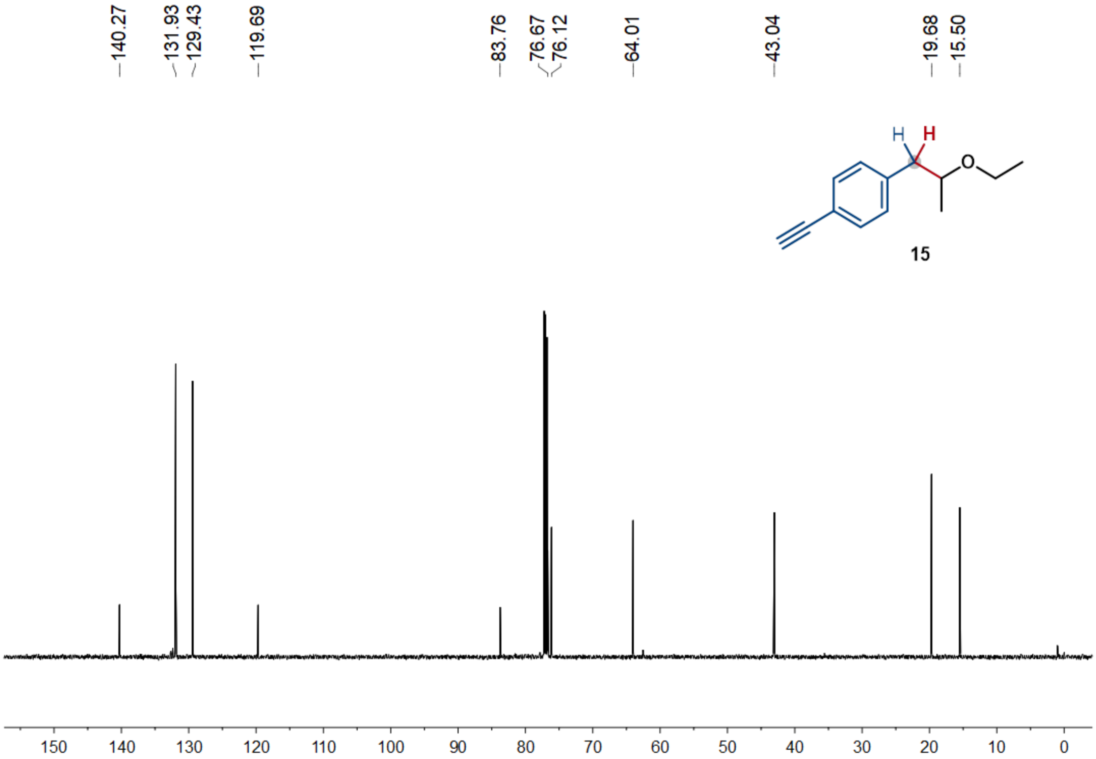

Supplementary Fig. 41 ^{13}C NMR (151 MHz, $CDCl_3$) spectrum of compound **15**

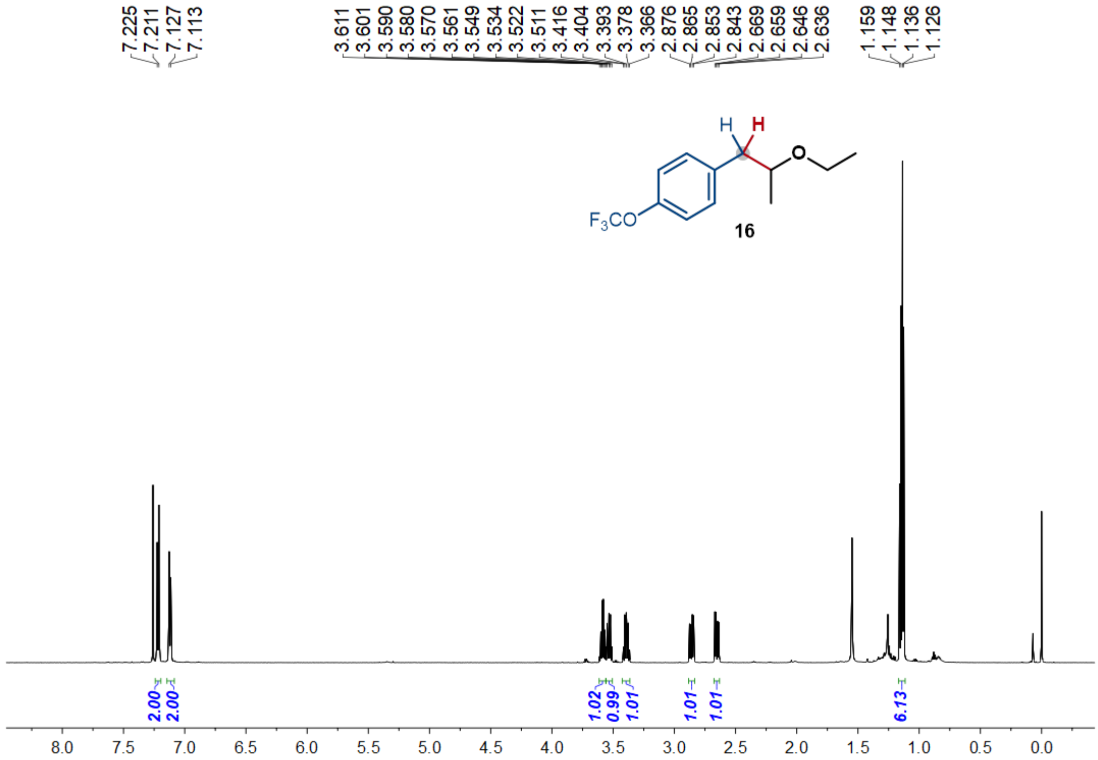

Supplementary Fig. 42 ^{1}H NMR (600 MHz, $CDCl_3$) spectrum of compound **16**

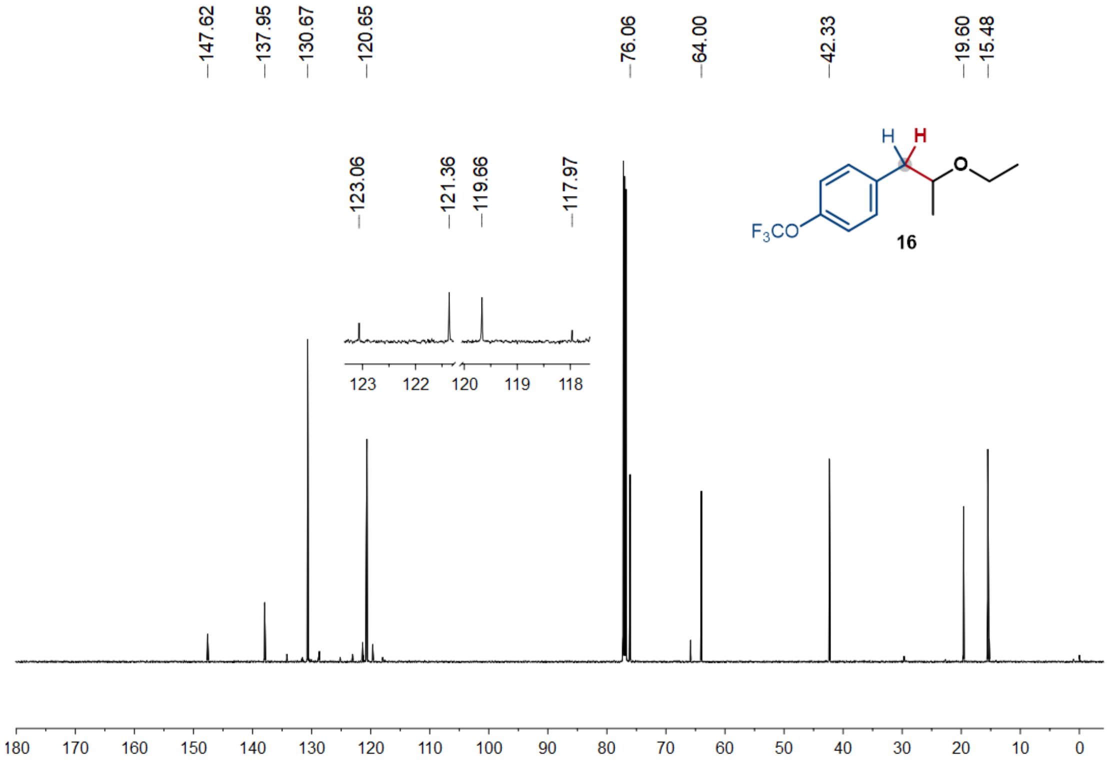

Supplementary Fig. 43 ^{13}C NMR (151 MHz, $CDCl_3$) spectrum of compound **16**

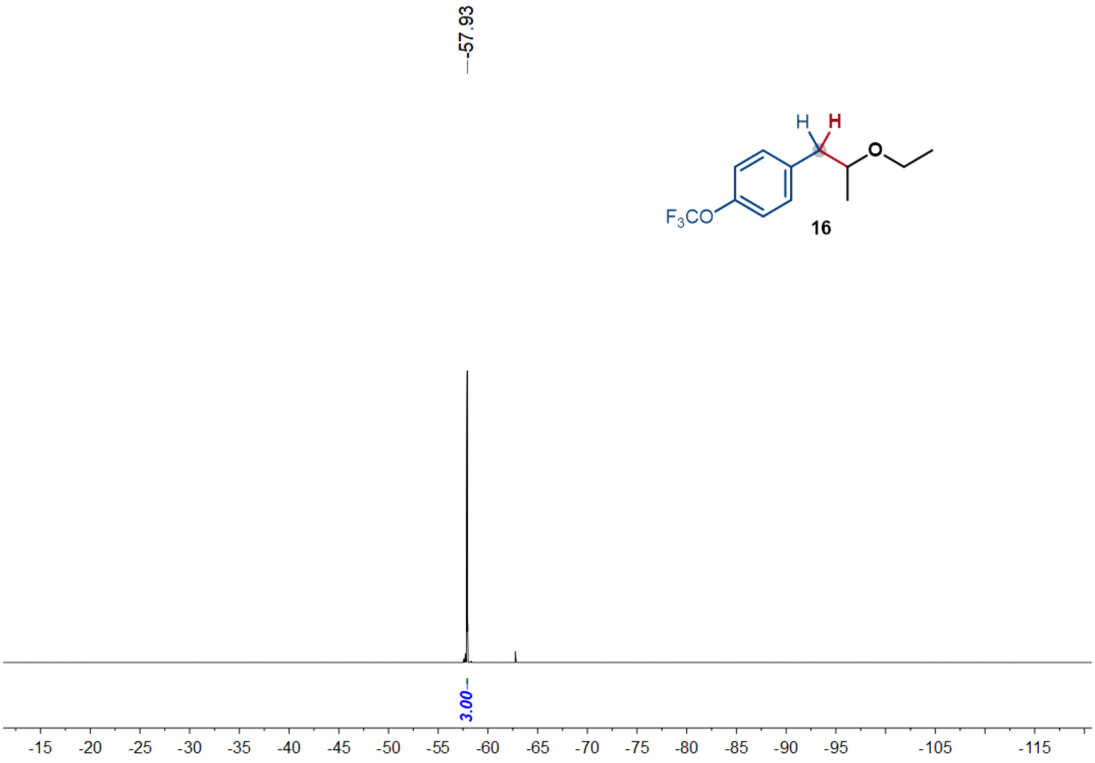

Supplementary Fig. 44 ^{19}F NMR (471 MHz, $CDCl_3$) spectrum of compound **16**

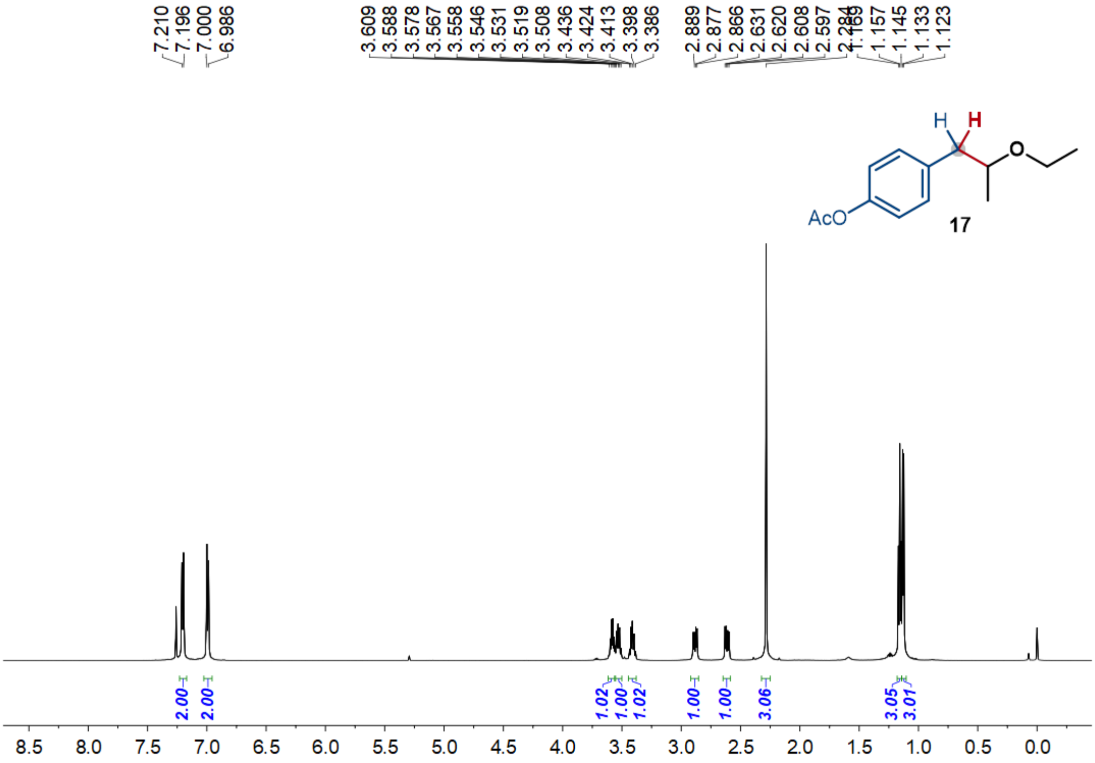

Supplementary Fig. 45 ^{1}H NMR (600 MHz, $CDCl_3$) spectrum of compound **17**

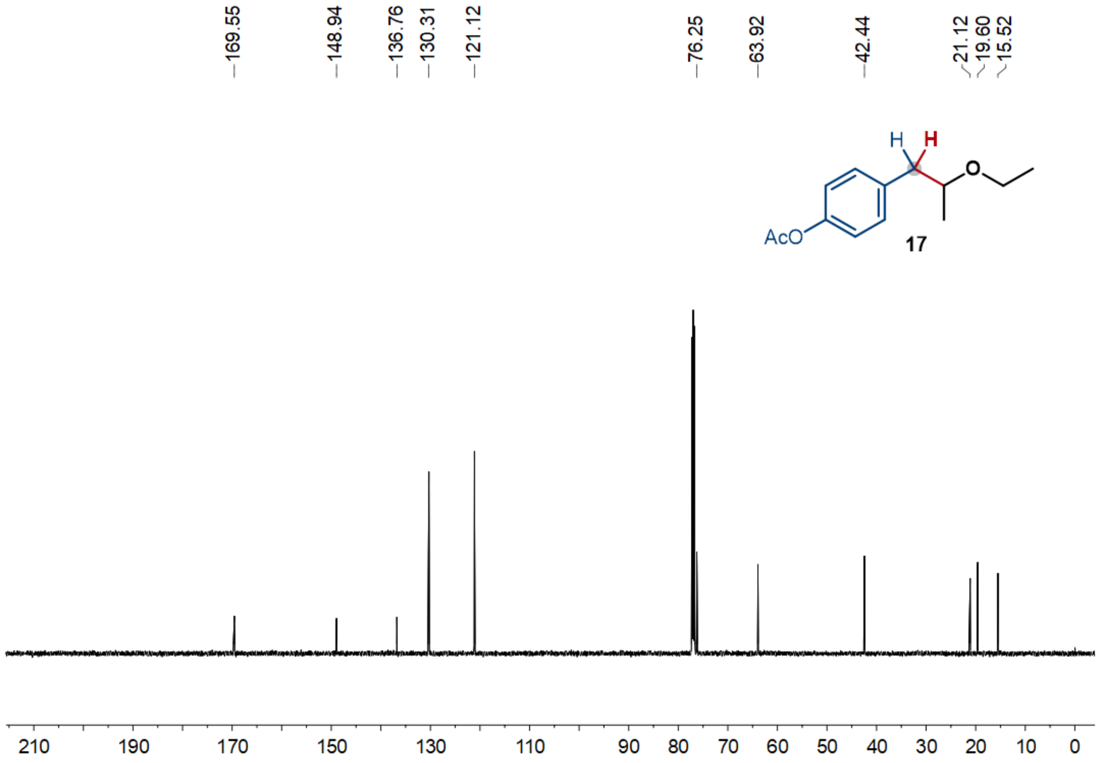

Supplementary Fig. 46 ^{13}C NMR (151 MHz, $CDCl_3$) spectrum of compound **17**

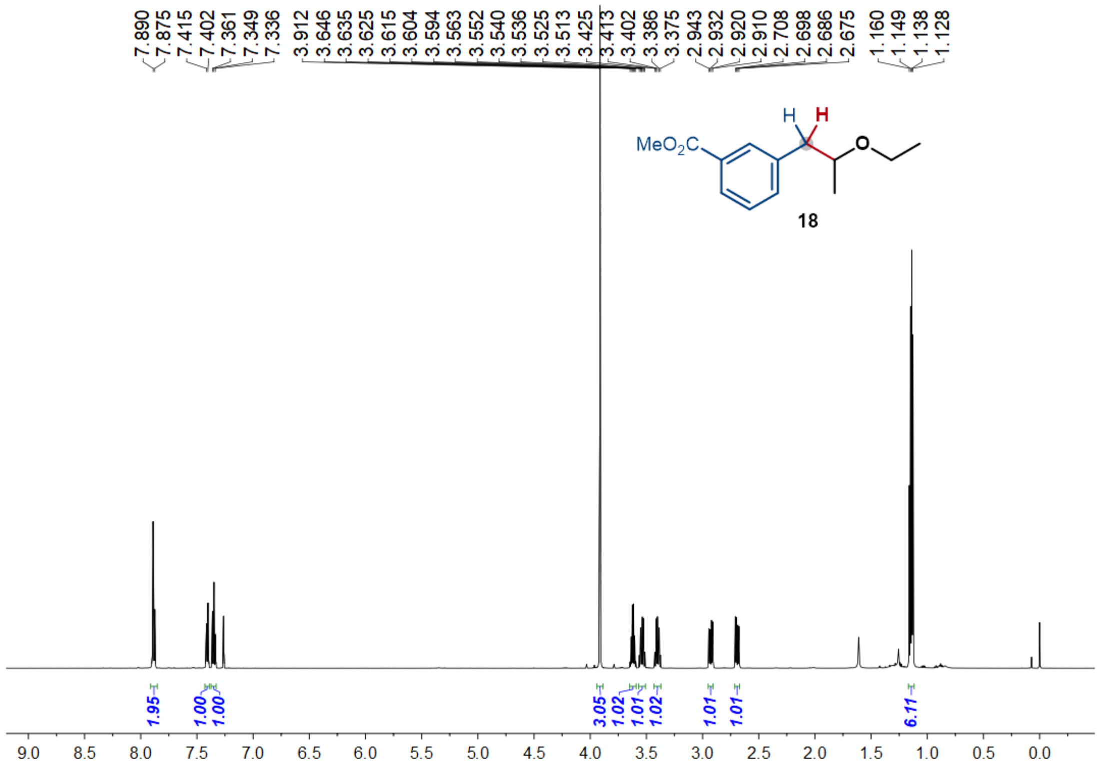

Supplementary Fig. 47 ^{1}H NMR (600 MHz, $CDCl_3$) spectrum of compound **18**

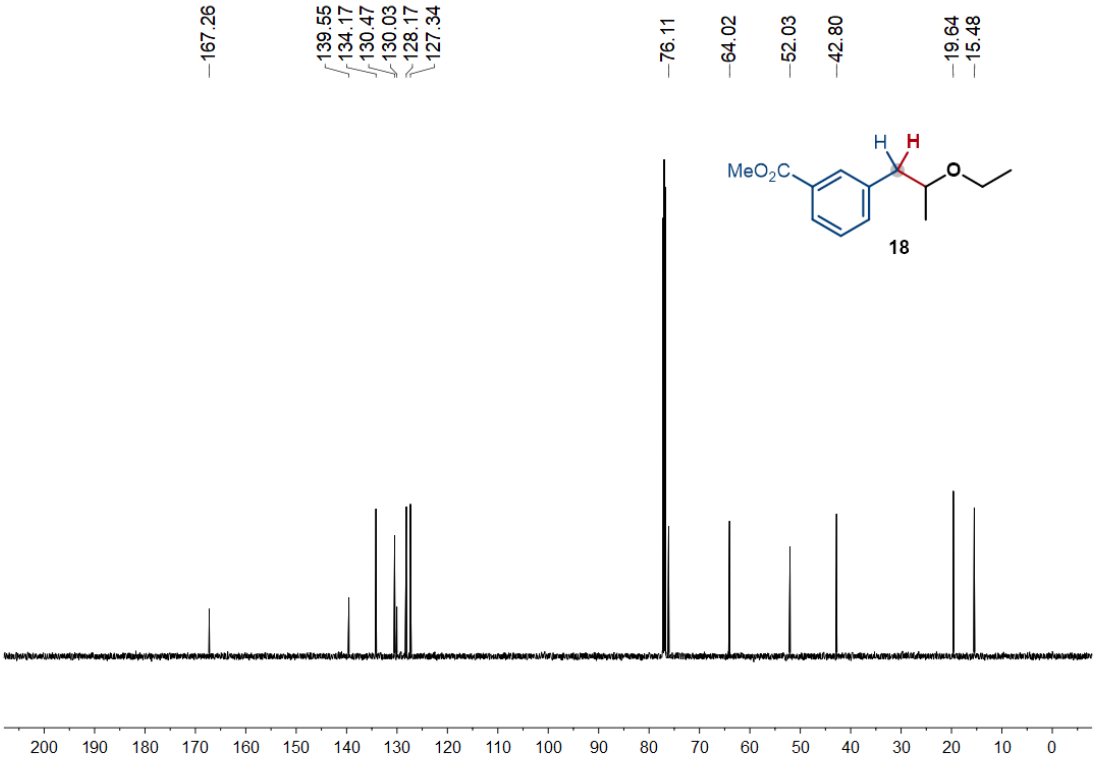

Supplementary Fig. 48 ^{13}C NMR (151 MHz, $CDCl_3$) spectrum of compound **18**

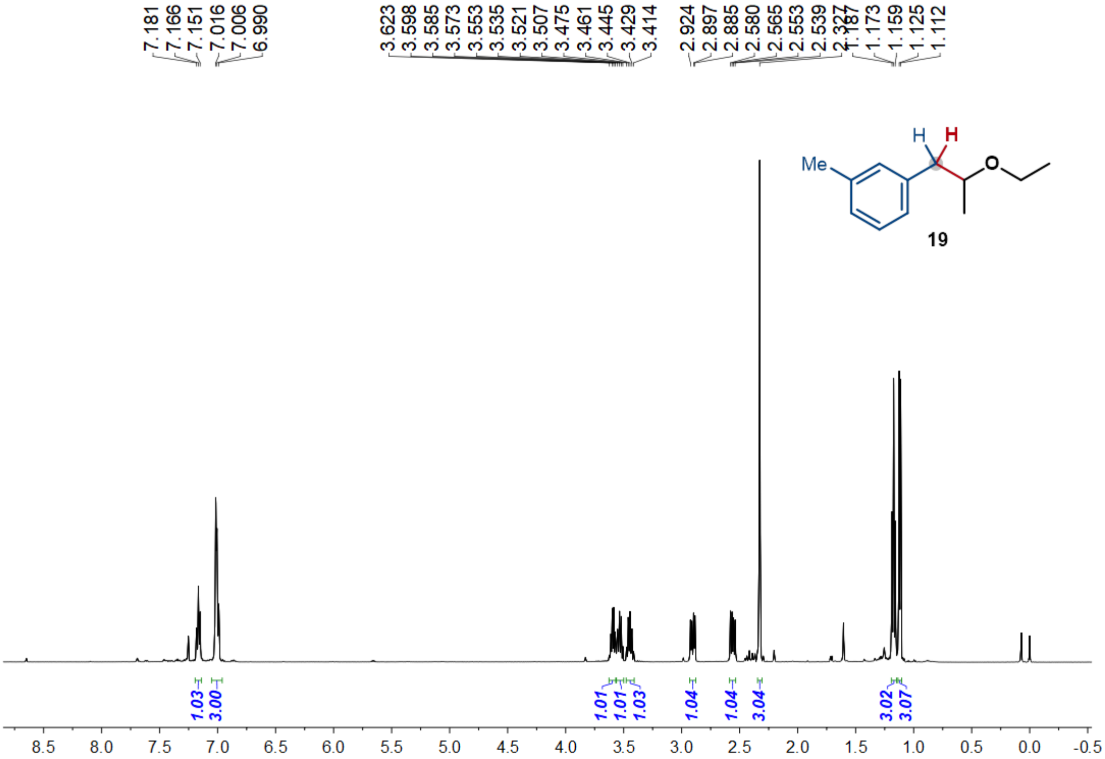

Supplementary Fig. 49 ^{1}H NMR (500 MHz, $CDCl_3$) spectrum of compound **19**

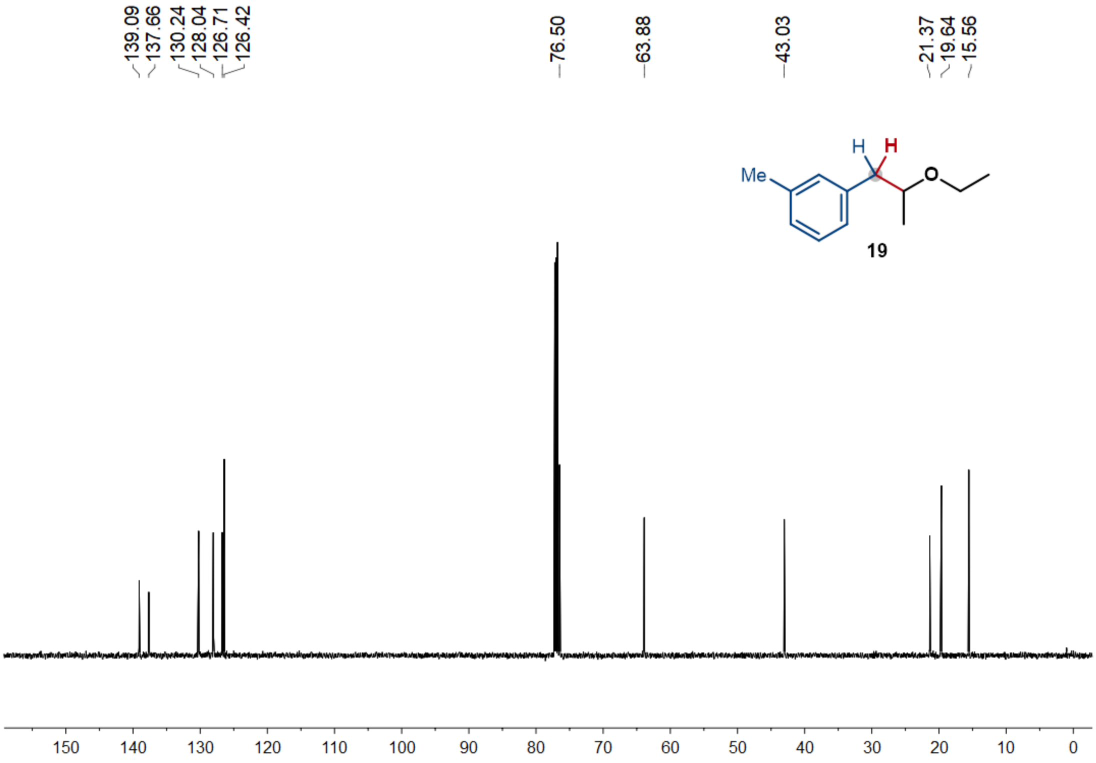

Supplementary Fig. 50 ^{13}C NMR (151 MHz, $CDCl_3$) spectrum of compound **19**

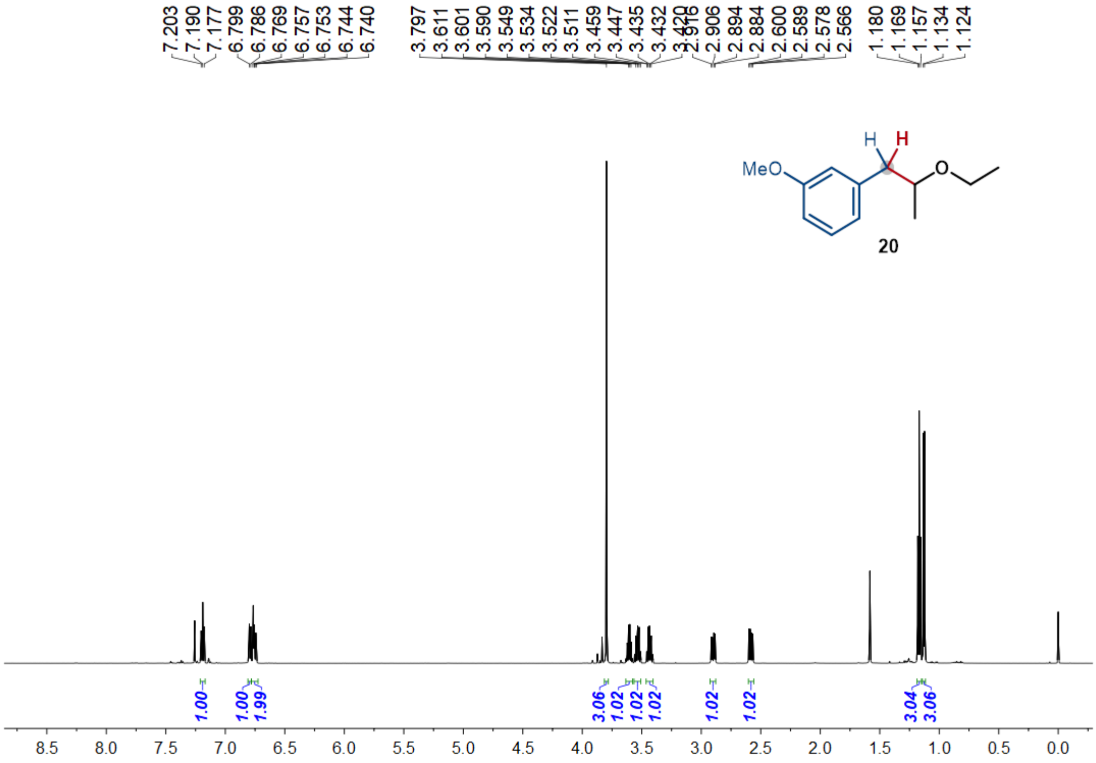

Supplementary Fig. 51 ^{1}H NMR (600 MHz, $CDCl_3$) spectrum of compound **20**

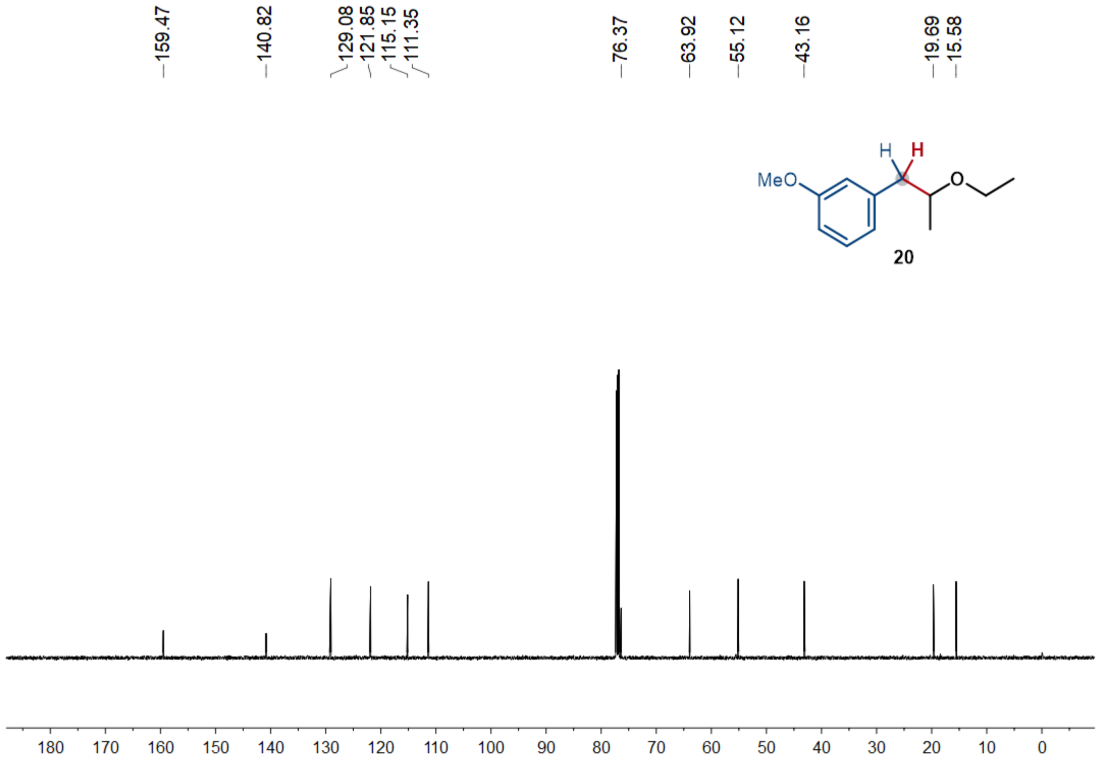

Supplementary Fig. 52 ^{13}**C NMR** (151 MHz, $CDCl_3$) spectrum of compound **20**

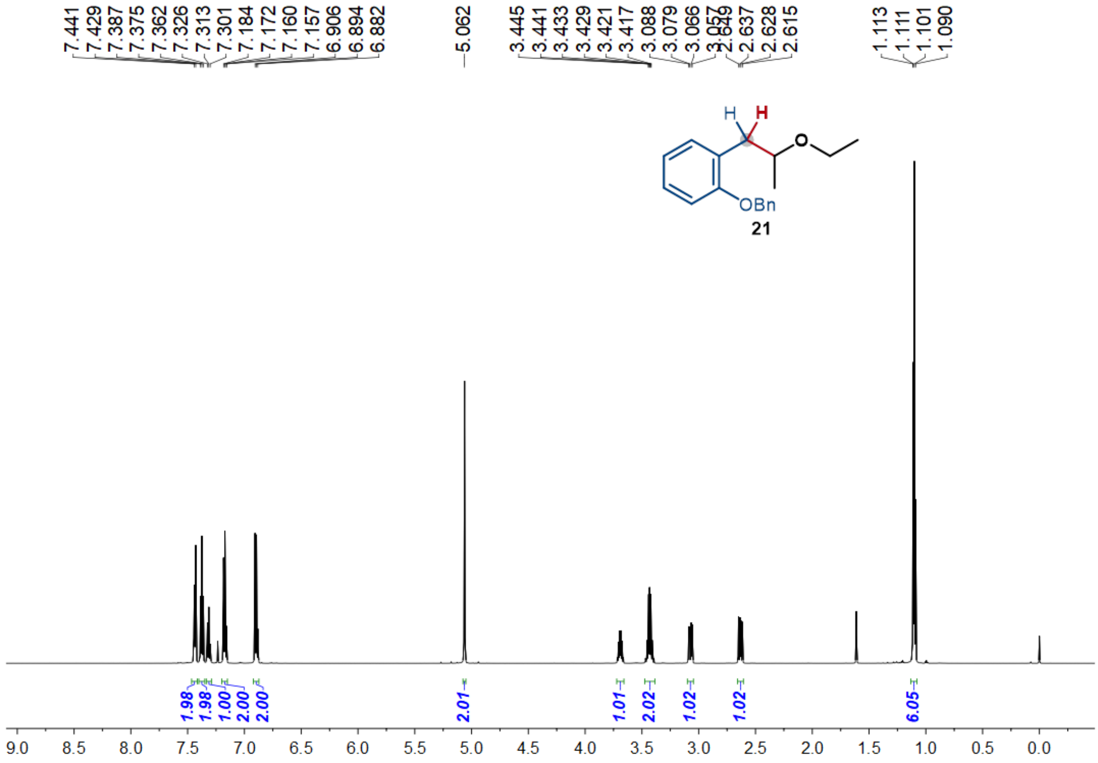

Supplementary Fig. 53 ^{1}**H NMR** (600 MHz, $CDCl_3$) spectrum of compound **21**

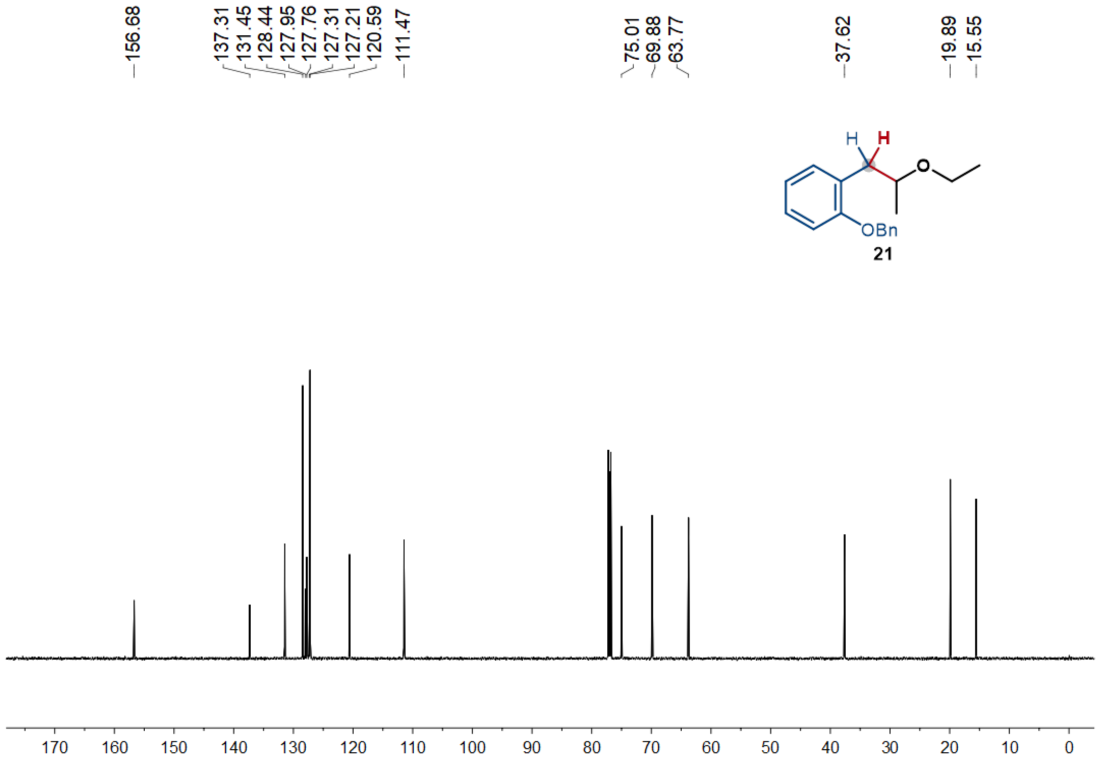

Supplementary Fig. 54 ^{13}C NMR (151 MHz, $CDCl_3$) spectrum of compound **21**

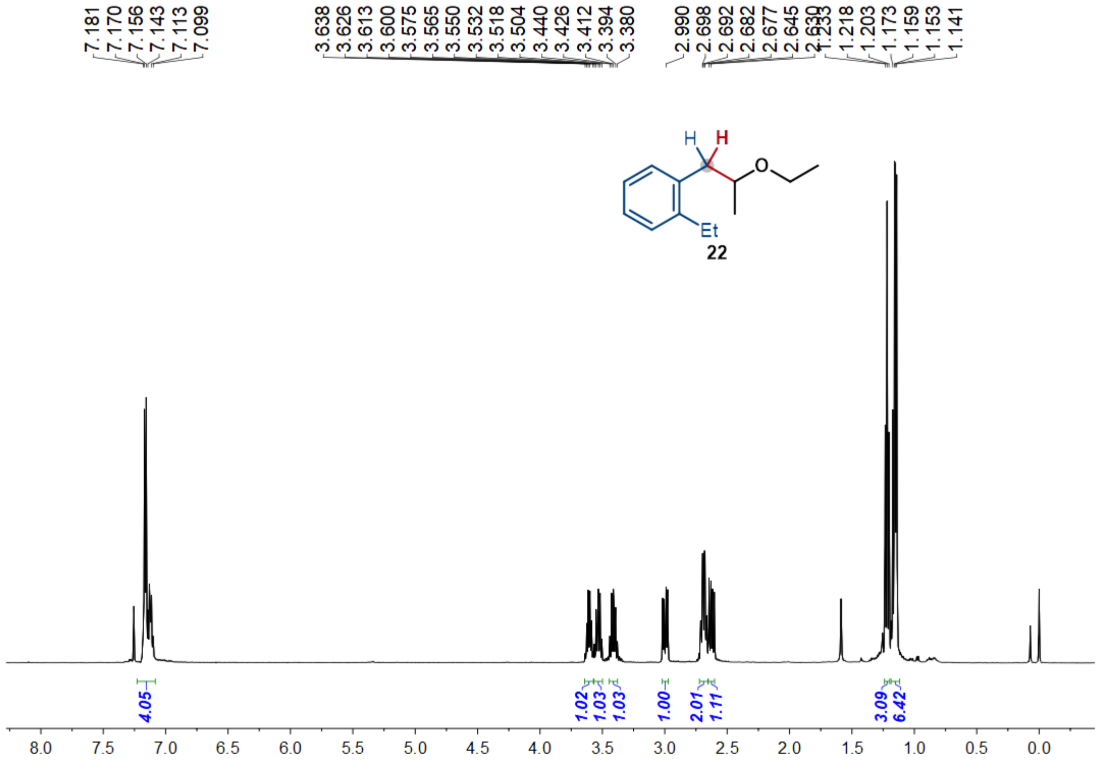

Supplementary Fig. 55 ^{1}H NMR (500 MHz, $CDCl_3$) spectrum of compound **22**

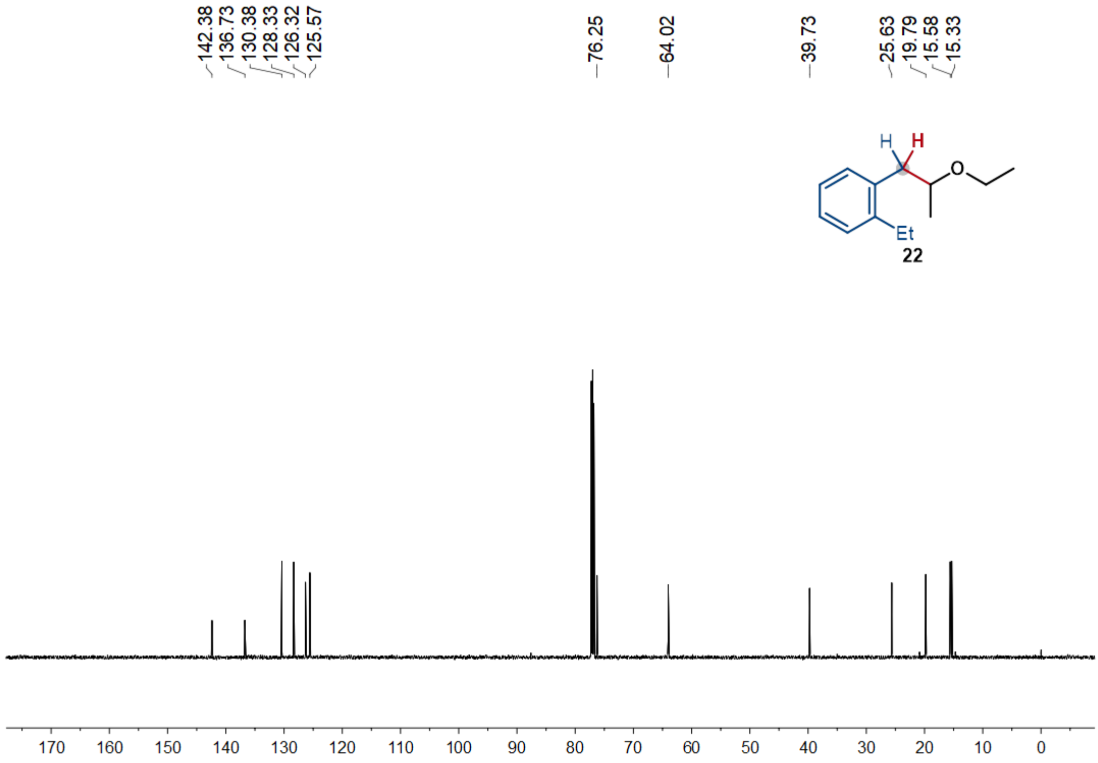

Supplementary Fig. 56 ^{13}C NMR (151 MHz, $CDCl_3$) spectrum of compound **22**

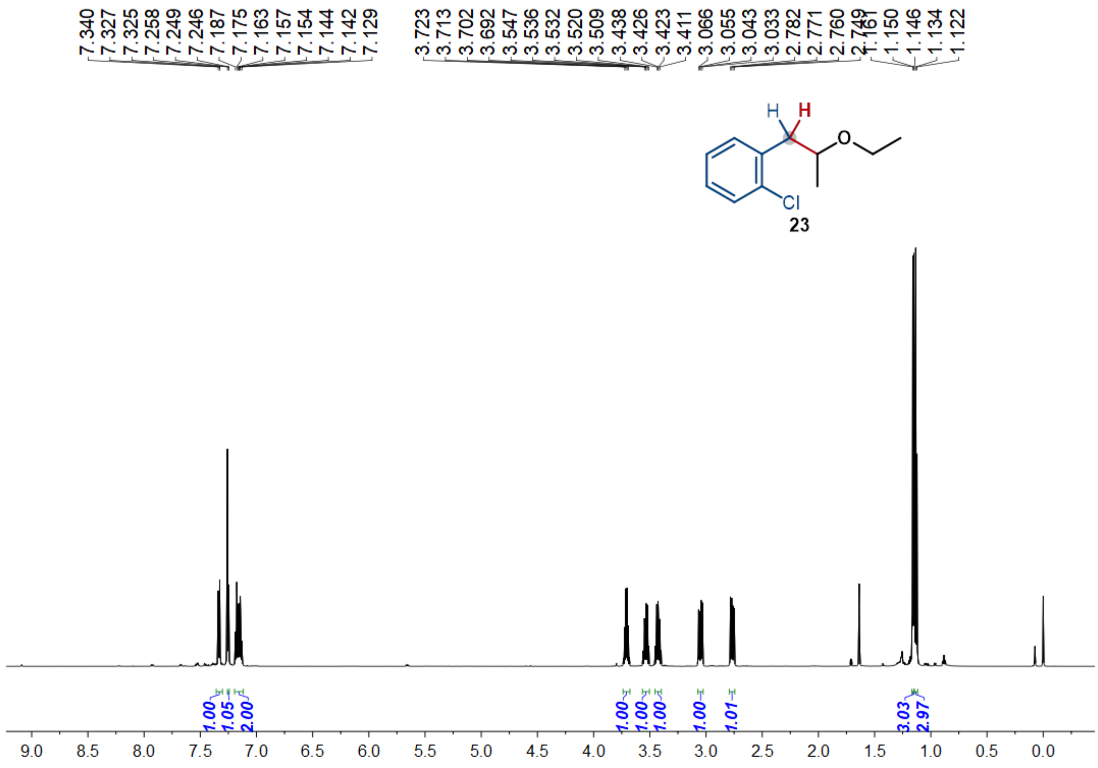

Supplementary Fig. 57 ^{1}H NMR (600 MHz, $CDCl_3$) spectrum of compound **23**

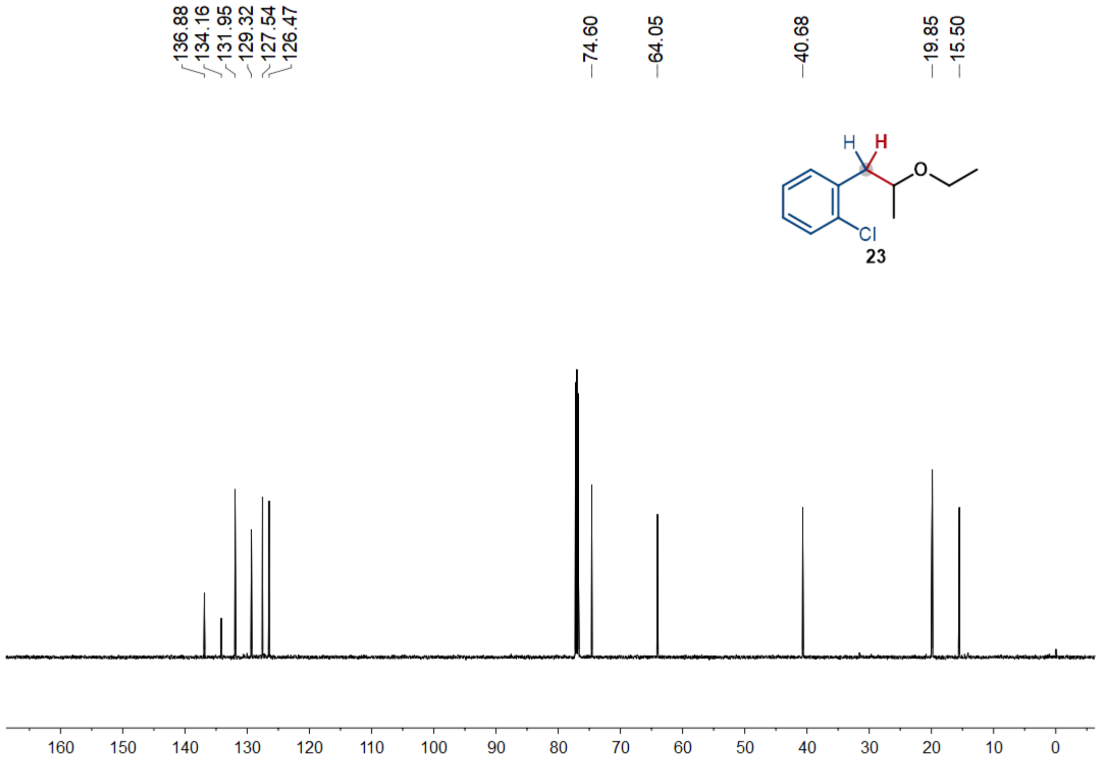

Supplementary Fig. 58 ^{13}C NMR (151 MHz, $CDCl_3$) spectrum of compound **23**

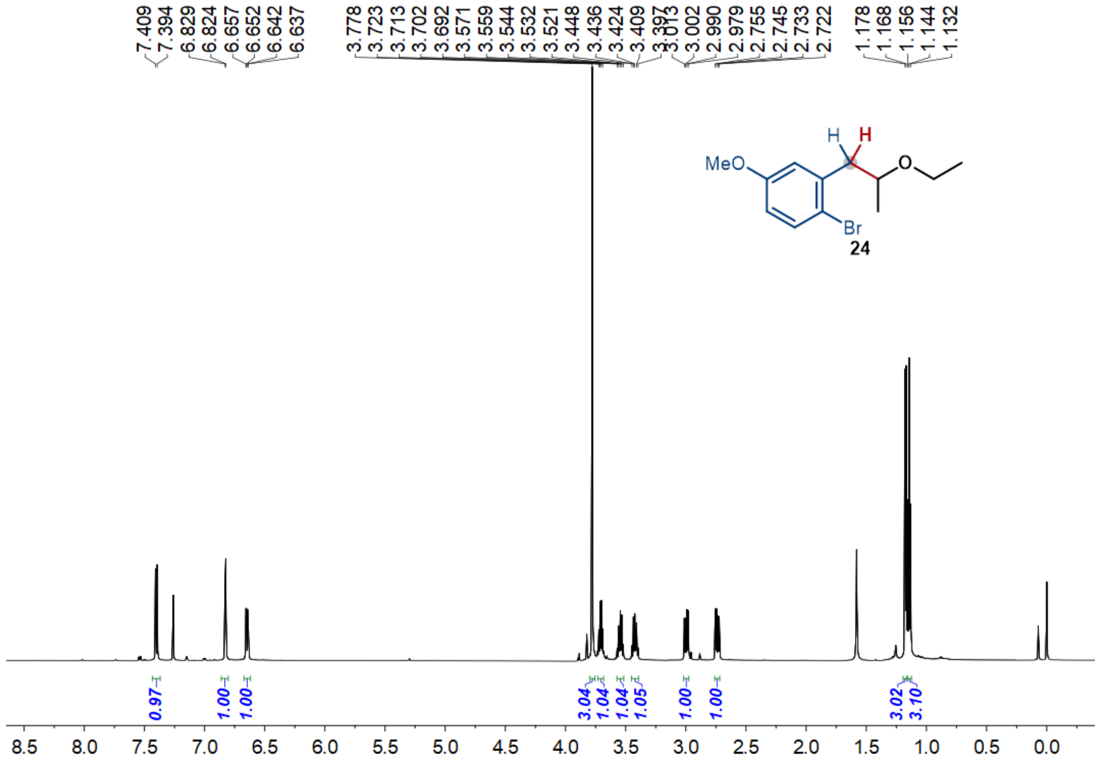

Supplementary Fig. 59 ^{1}H NMR (600 MHz, $CDCl_3$) spectrum of compound **24**

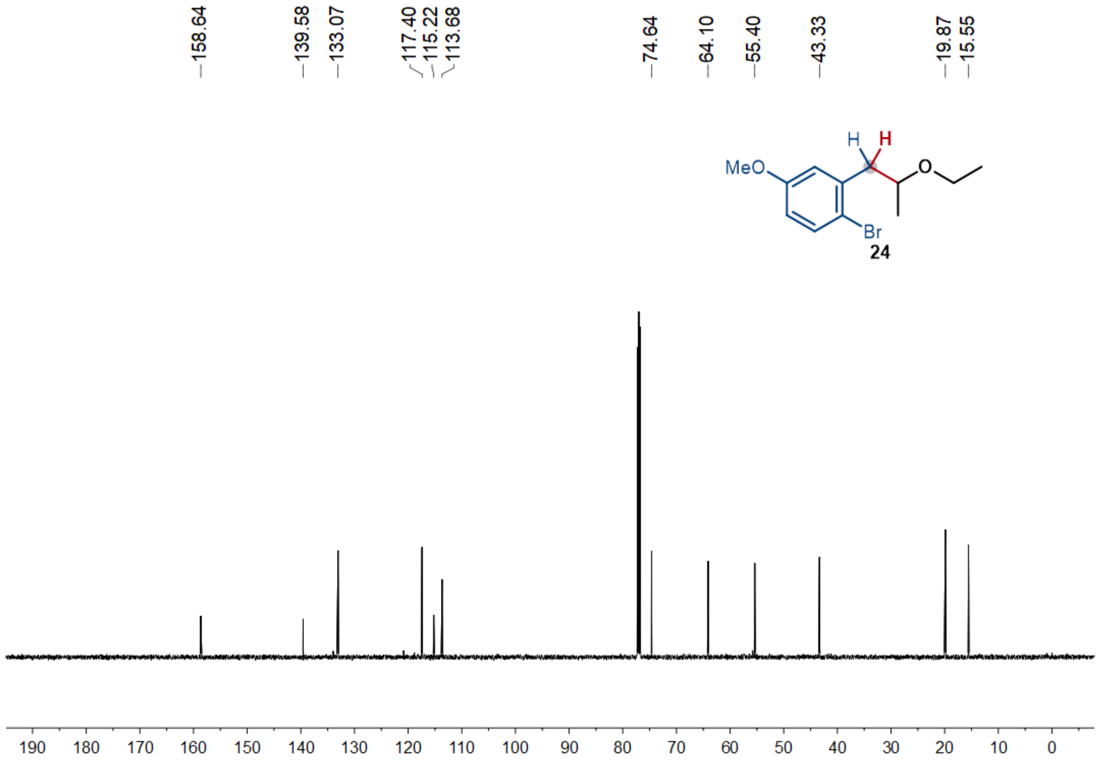

Supplementary Fig. 60 ^{13}**C NMR** (151 MHz, $CDCl_3$) spectrum of compound **24**

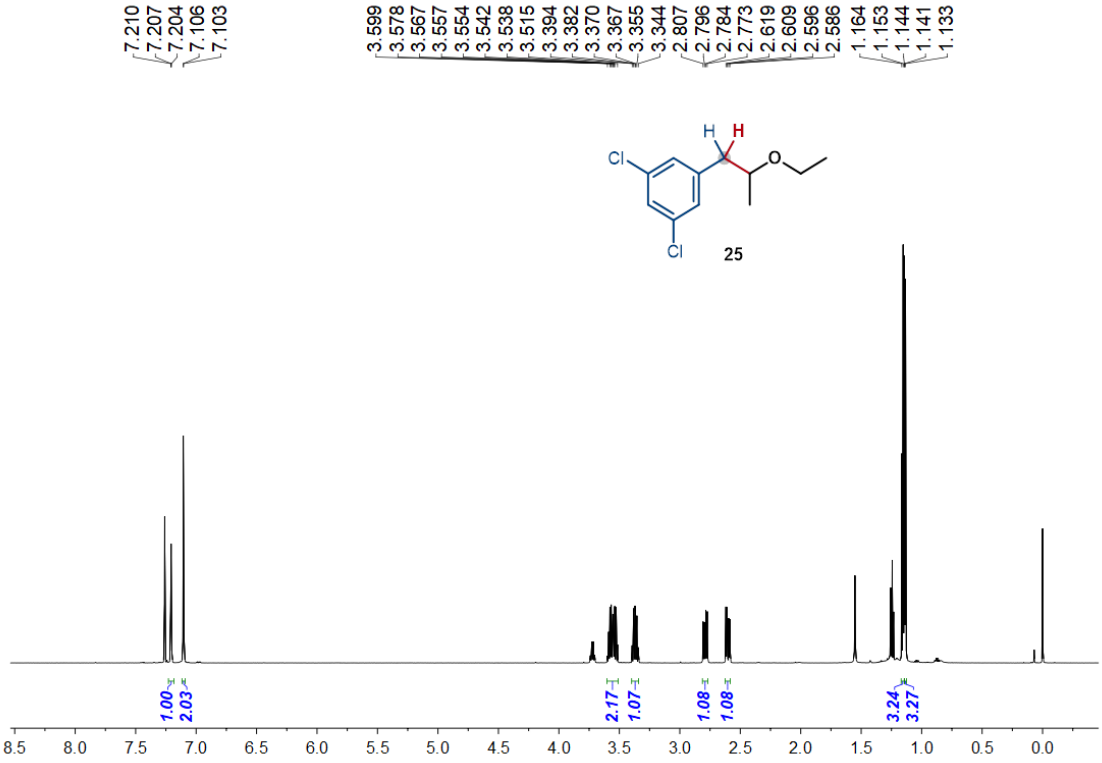

Supplementary Fig. 61 ^{1}**H NMR** (600 MHz, $CDCl_3$) spectrum of compound **25**

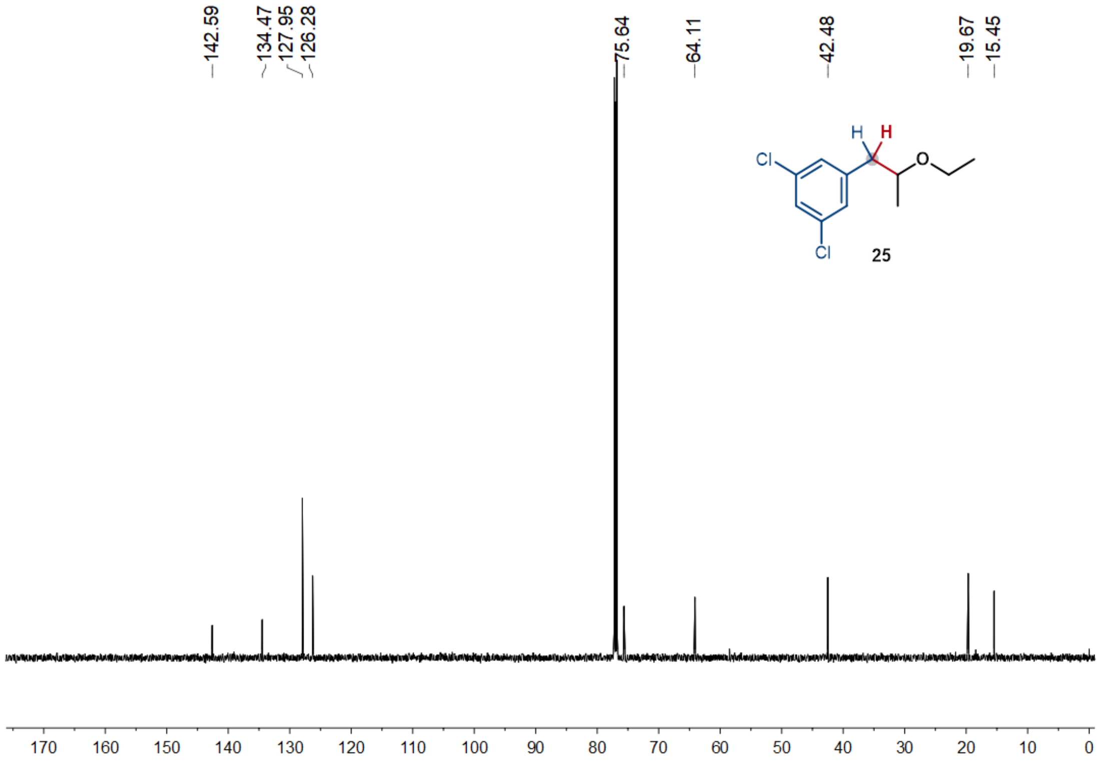

Supplementary Fig. 62 ^{13}**C NMR** (151 MHz, $CDCl_3$) spectrum of compound **25**

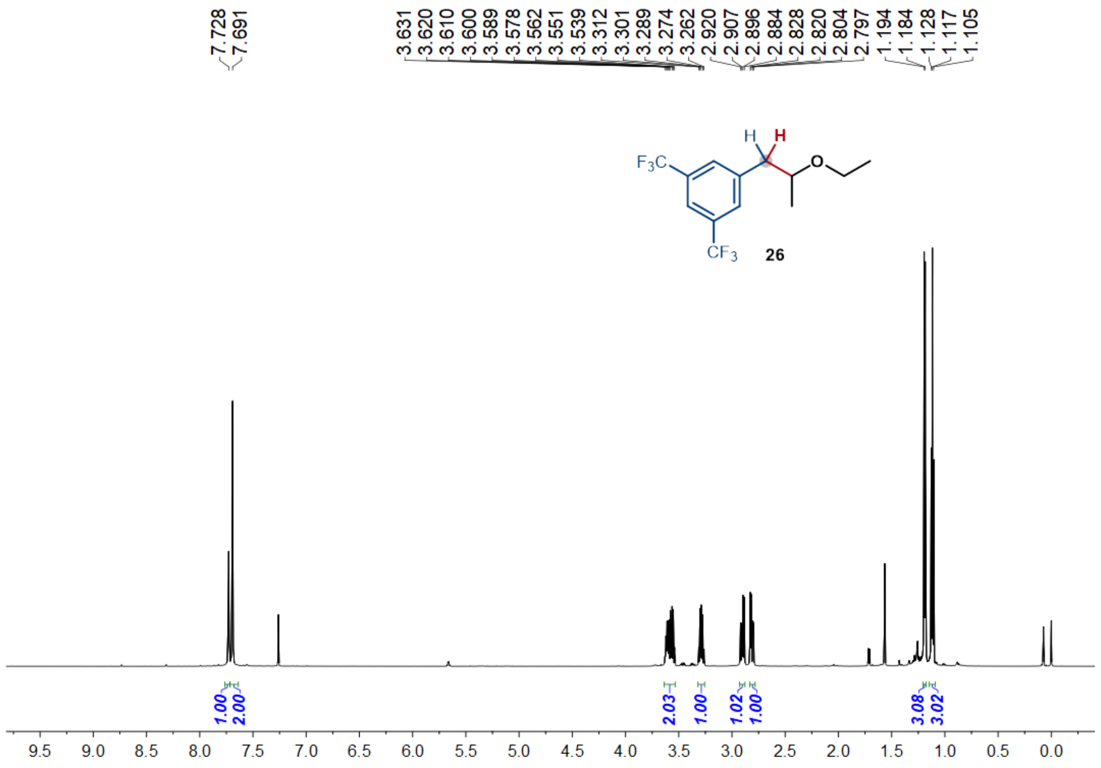

Supplementary Fig. 63 ^{1}**H NMR** (600 MHz, $CDCl_3$) spectrum of compound **26**

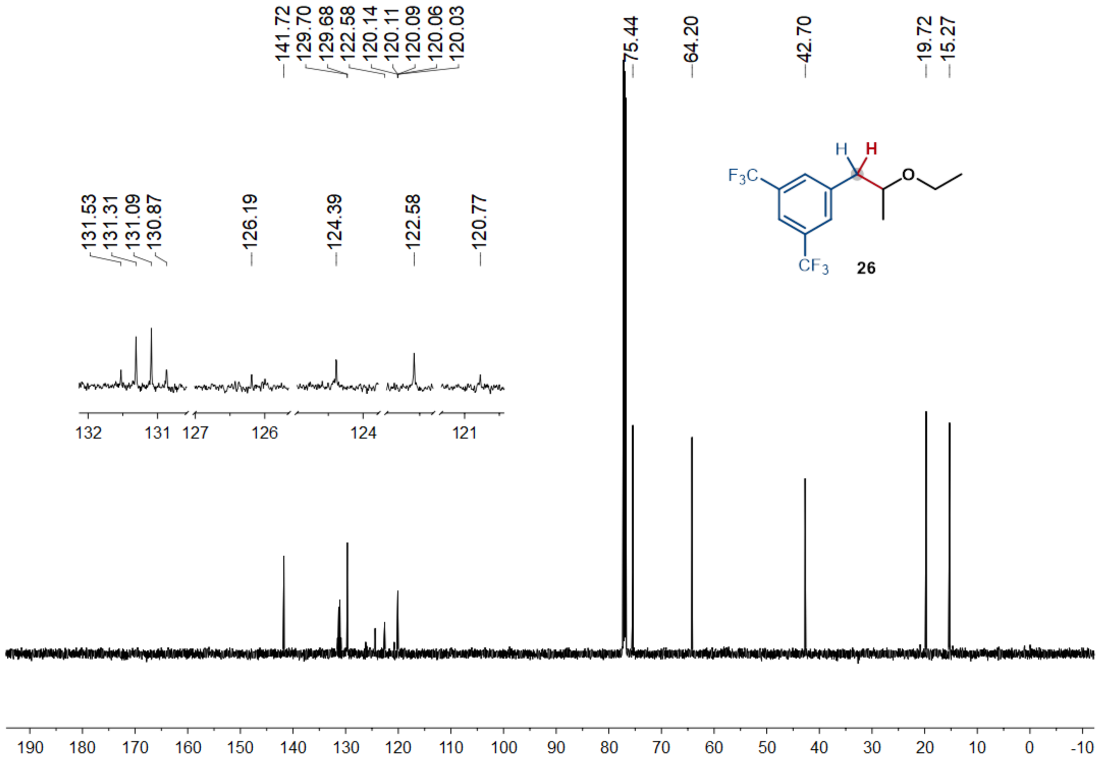

Supplementary Fig. 64 ^{13}**C NMR** (151 MHz, $CDCl_3$) spectrum of compound **26**

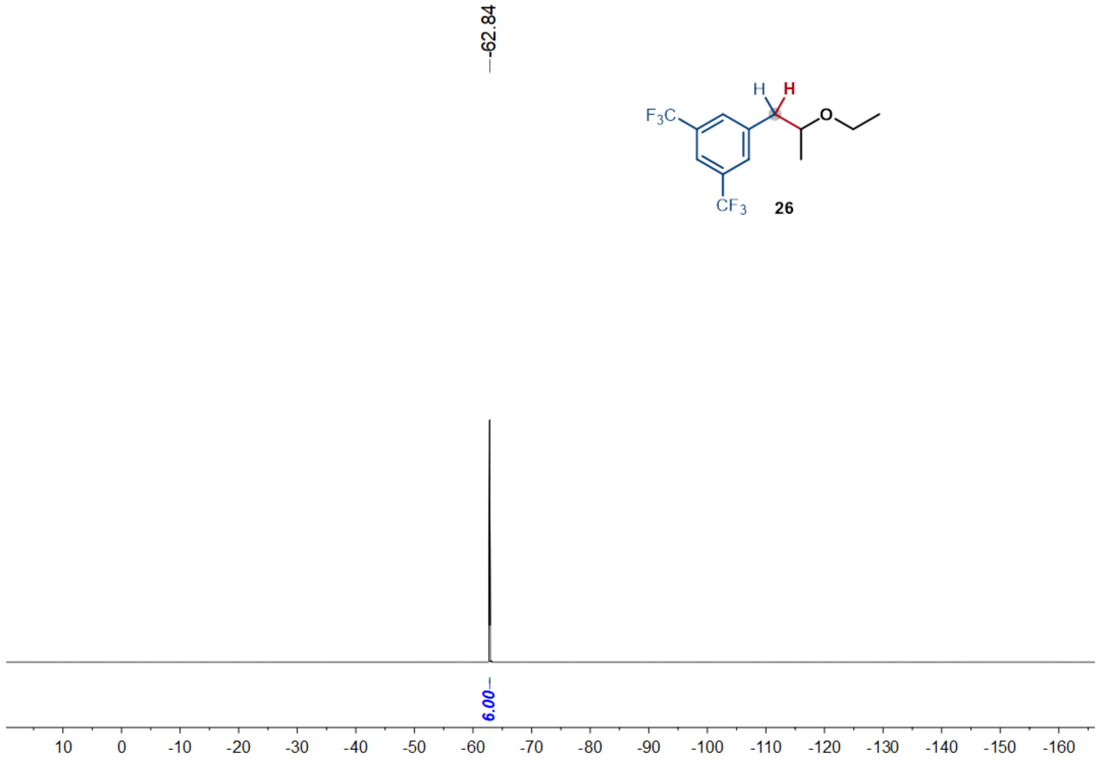

Supplementary Fig. 65 ^{19}**F NMR** (471 MHz, $CDCl_3$) spectrum of compound **26**

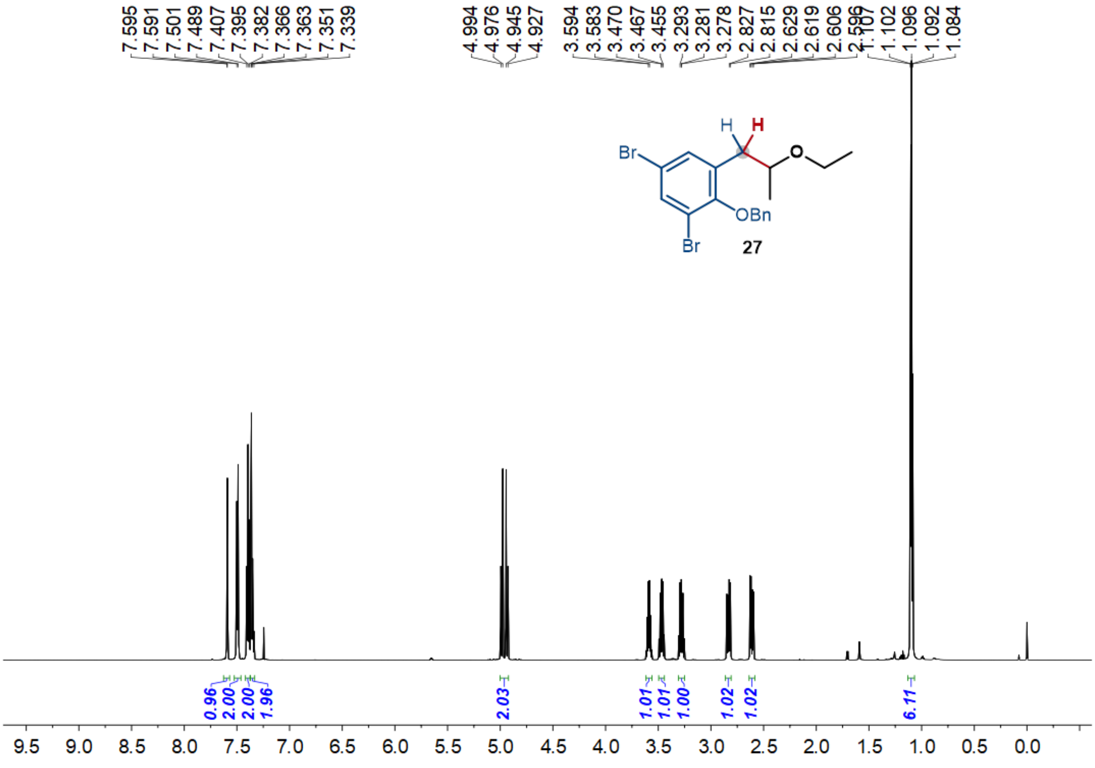

Supplementary Fig. 66 ^{1}**H NMR** (600 MHz, $CDCl_3$) spectrum of compound **27**

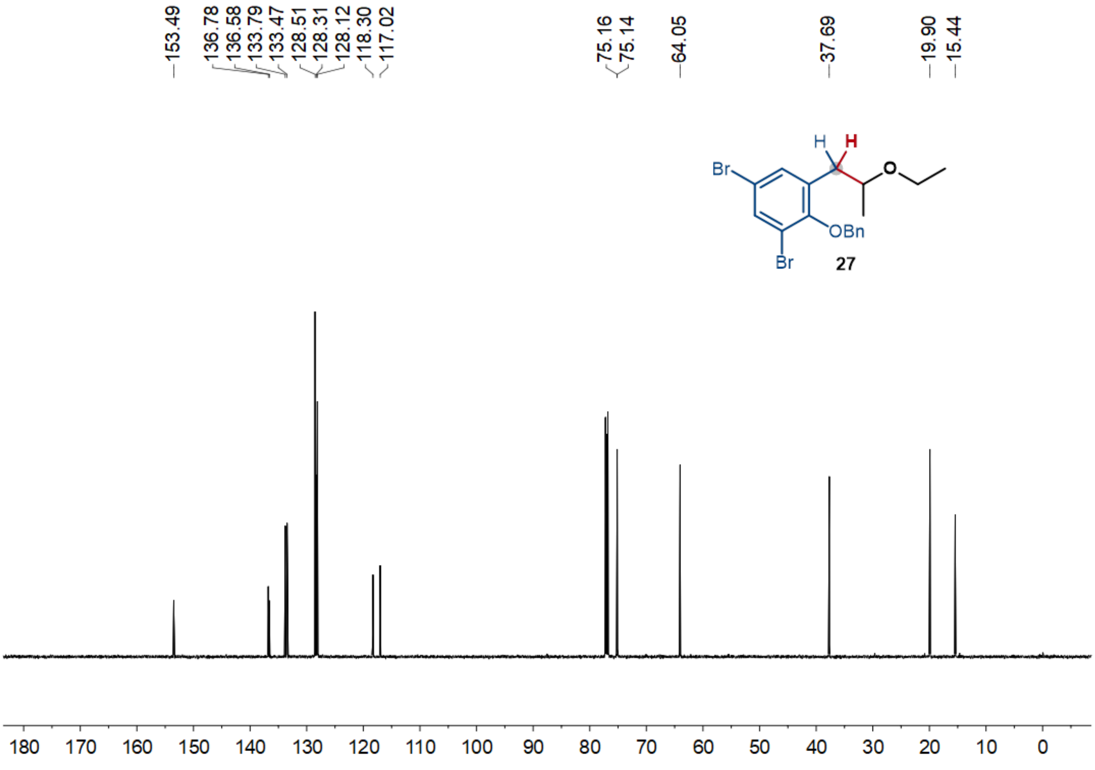

Supplementary Fig. 67 ^{1}**H NMR** (600 MHz, $CDCl_3$) spectrum of compound **27**

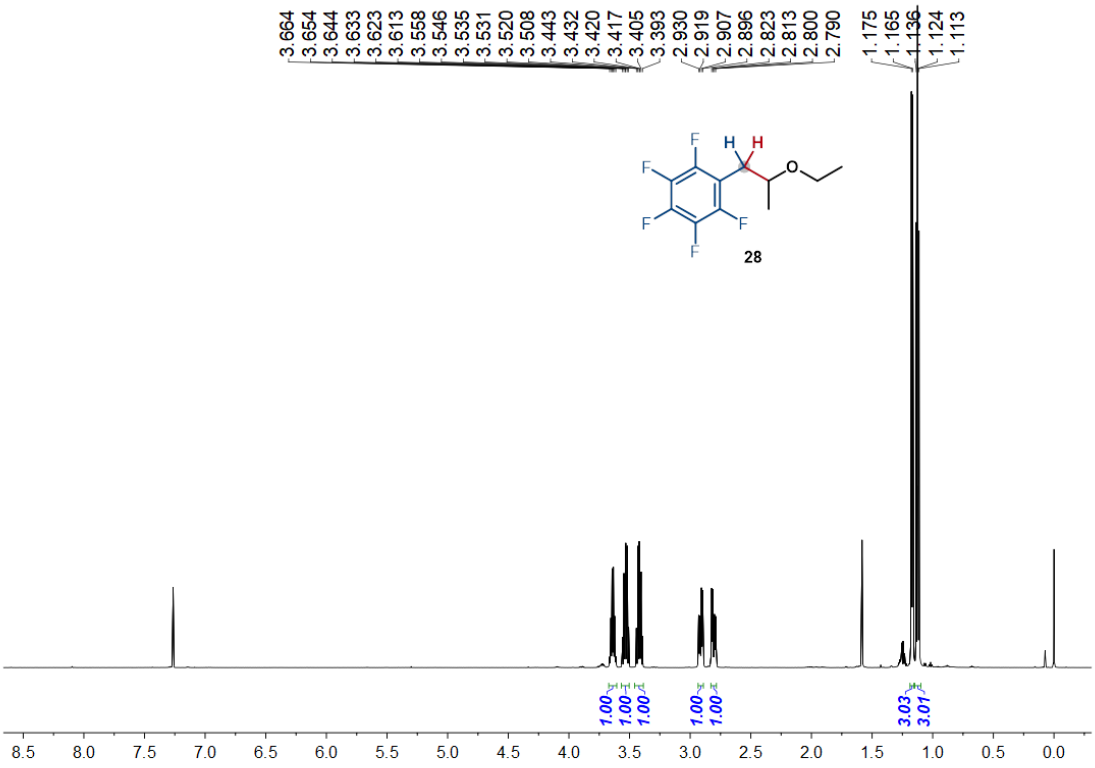

Supplementary Fig. 68 ^{1}H NMR (600 MHz, $CDCl_3$) spectrum of compound **28**

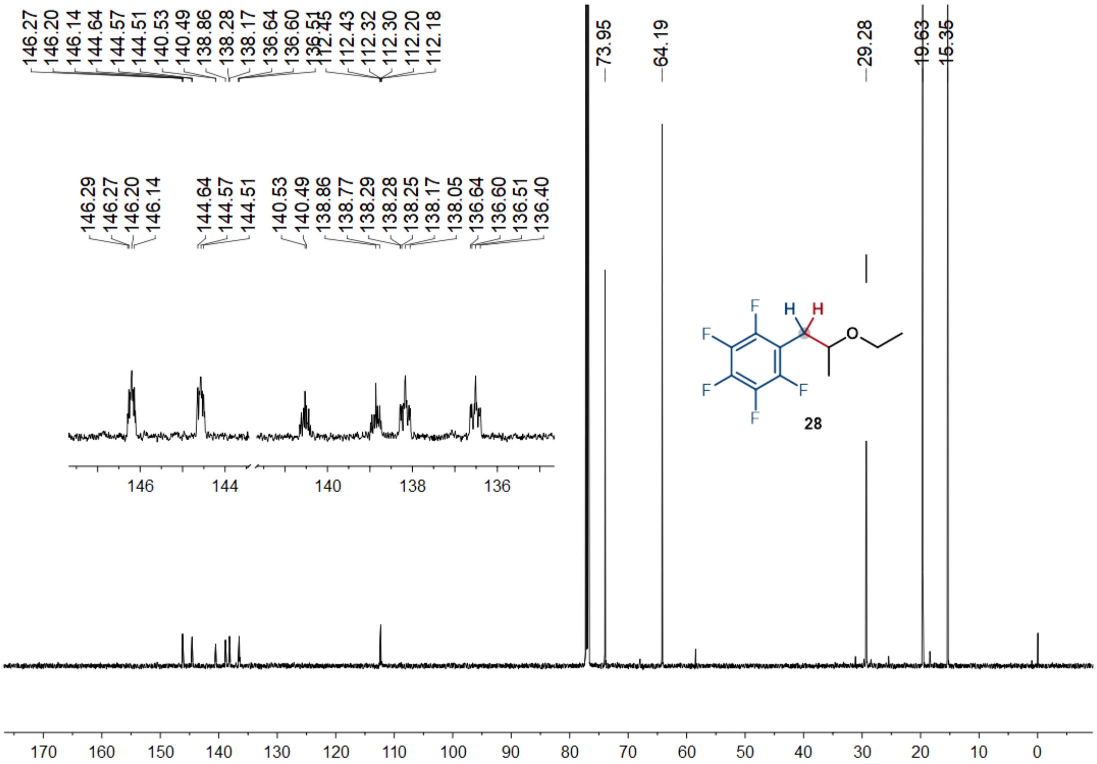

Supplementary Fig. 69 ^{13}C NMR (151 MHz, $CDCl_3$) spectrum of compound **28**

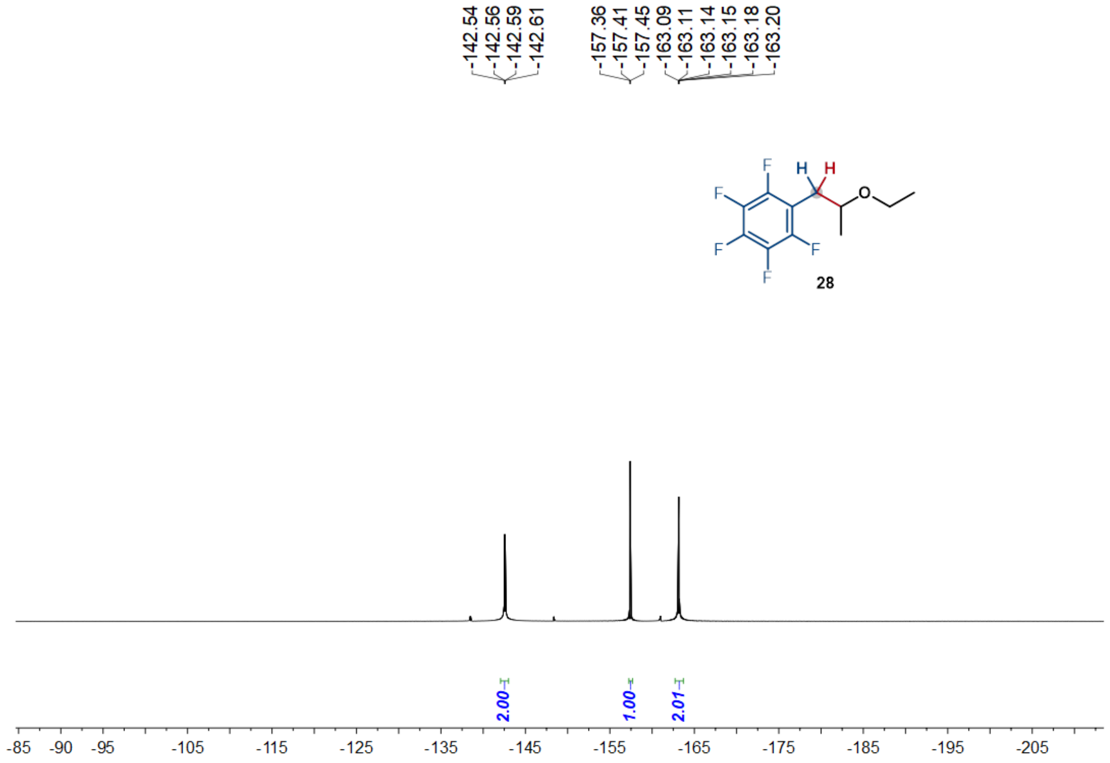

Supplementary Fig. 70 ^{19}F NMR (471 MHz, $CDCl_3$) spectrum of compound **28**

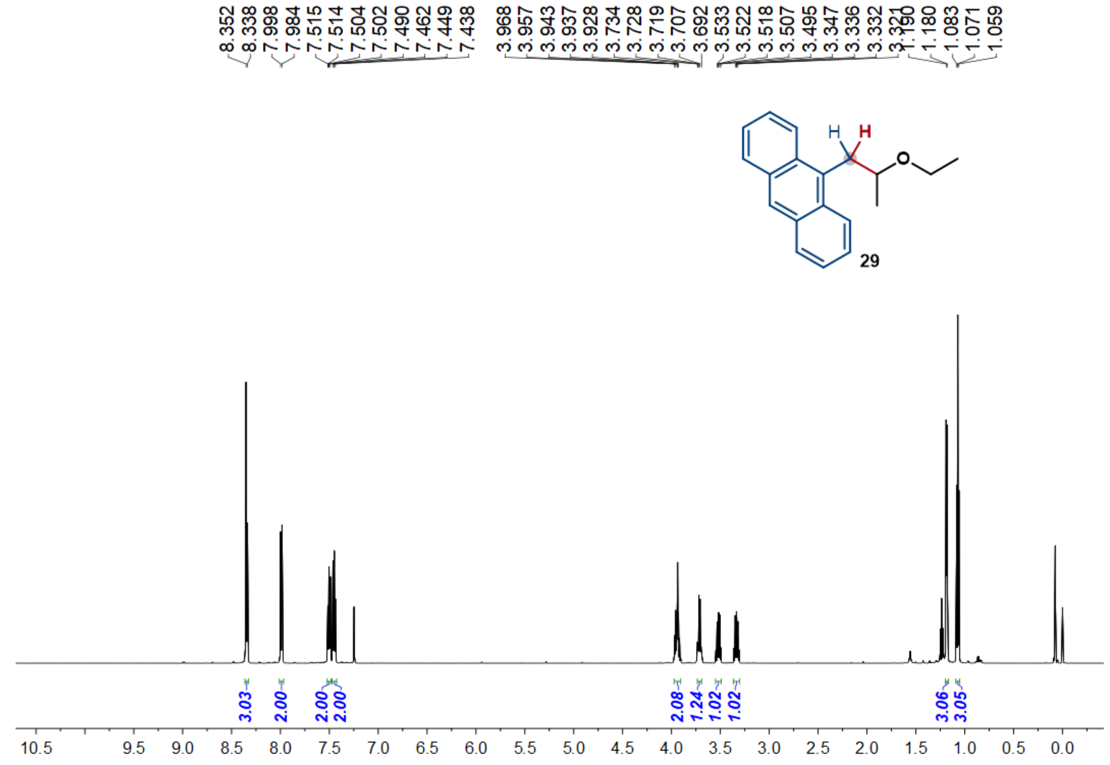

Supplementary Fig. 71 ^{1}H NMR (600 MHz, $CDCl_3$) spectrum of compound **29**

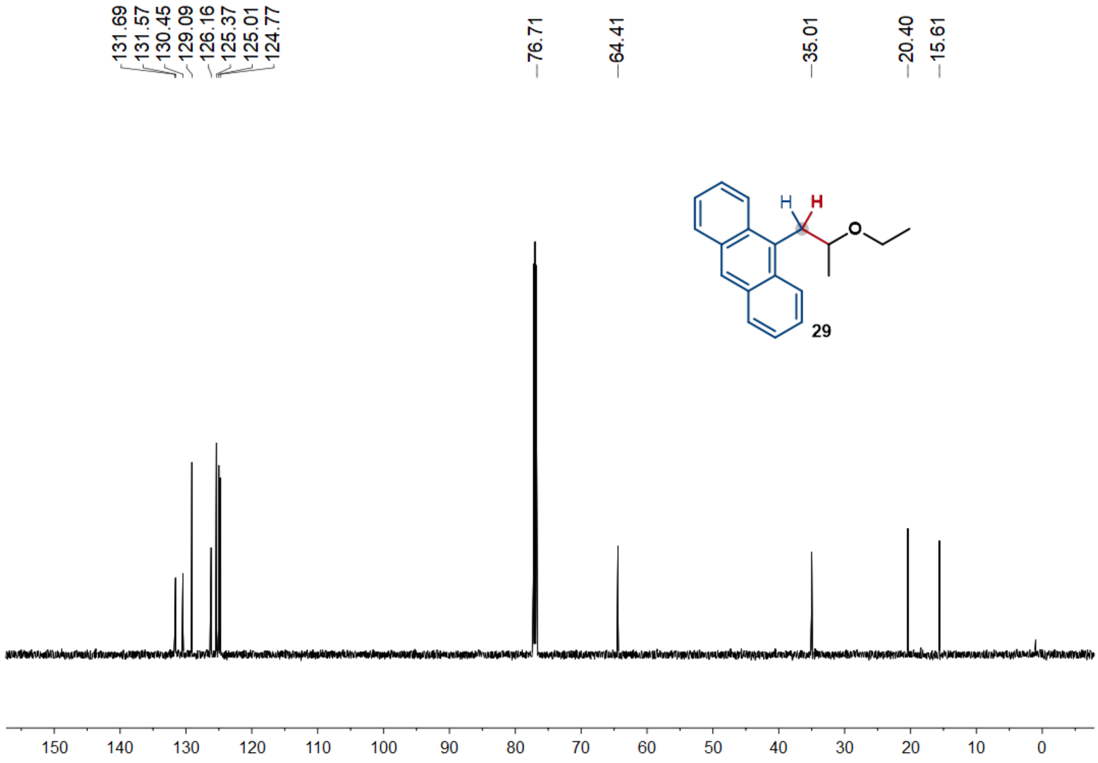

Supplementary Fig. 72 ^{13}C NMR (151 MHz, $CDCl_3$) spectrum of compound **29**

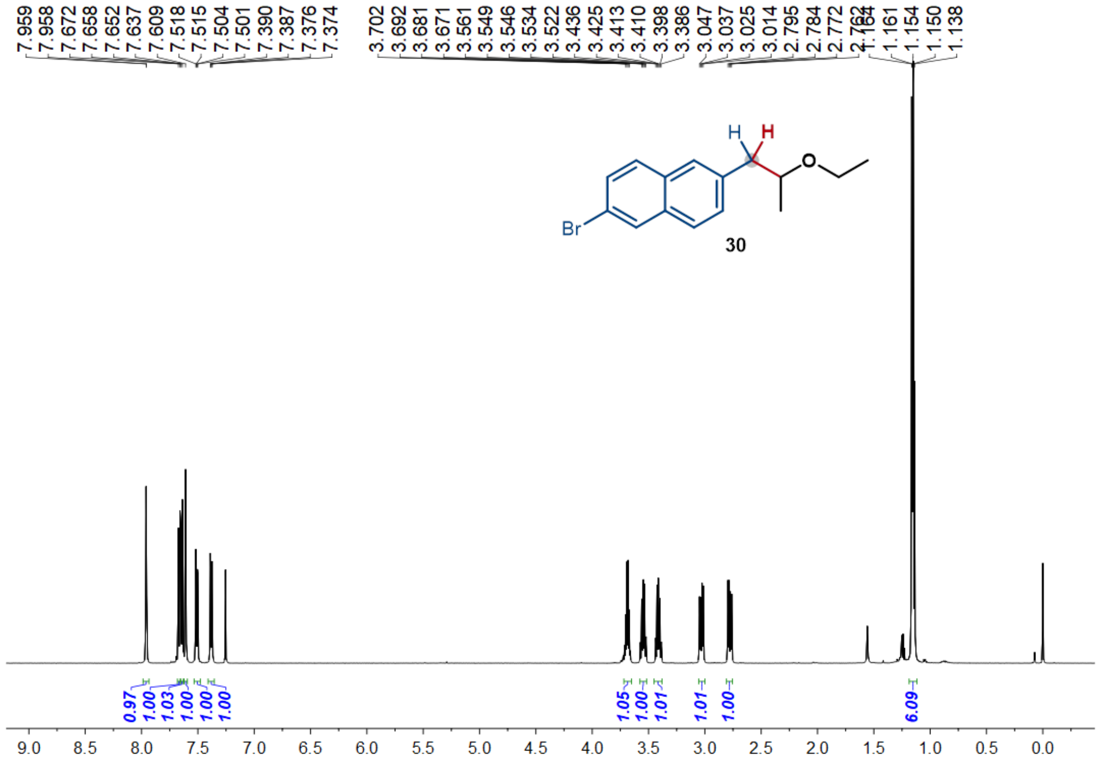

Supplementary Fig. 73 ^{1}H NMR (600 MHz, $CDCl_3$) spectrum of compound **30**

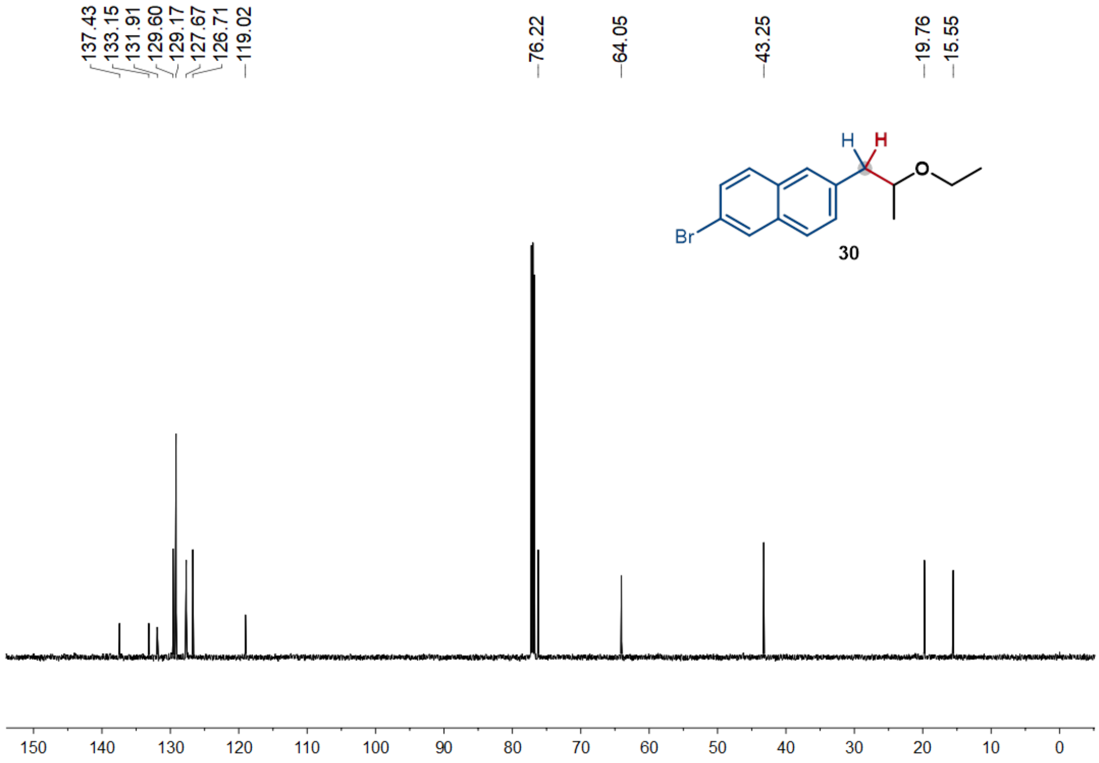

Supplementary Fig. 74 ^{13}**C NMR** (151 MHz, $CDCl_3$) spectrum of compound **30**

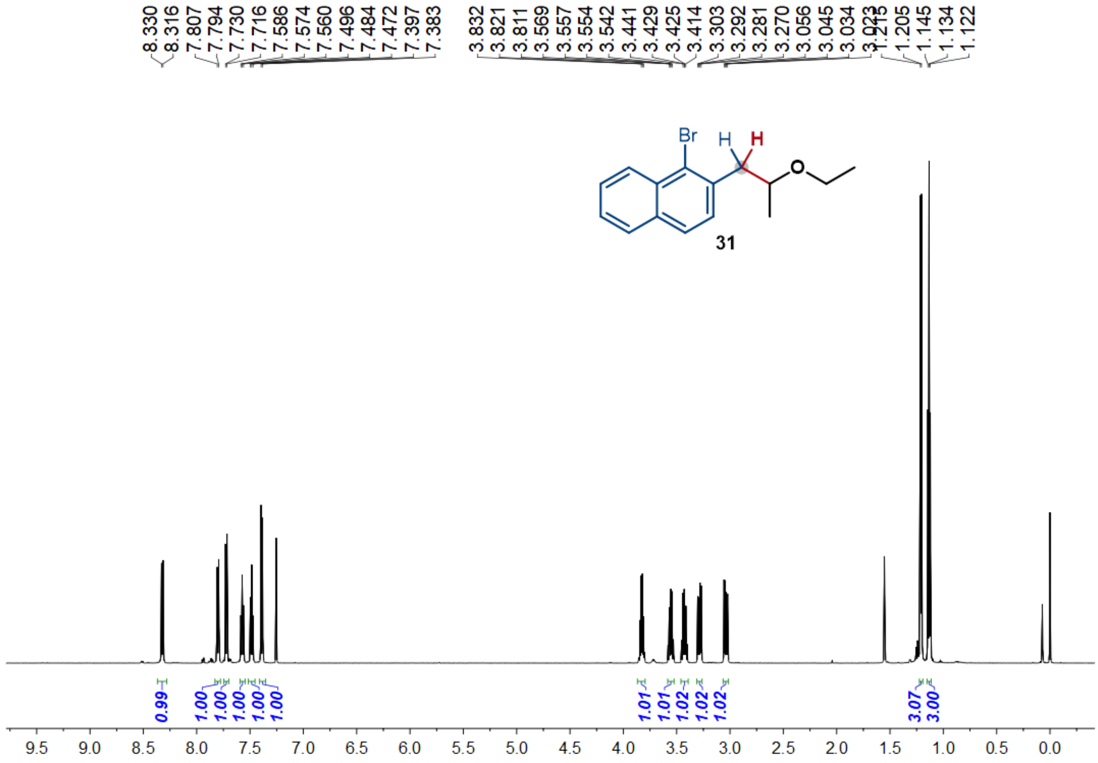

Supplementary Fig. 75 ^{1}**H NMR** (600 MHz, $CDCl_3$) spectrum of compound **31**

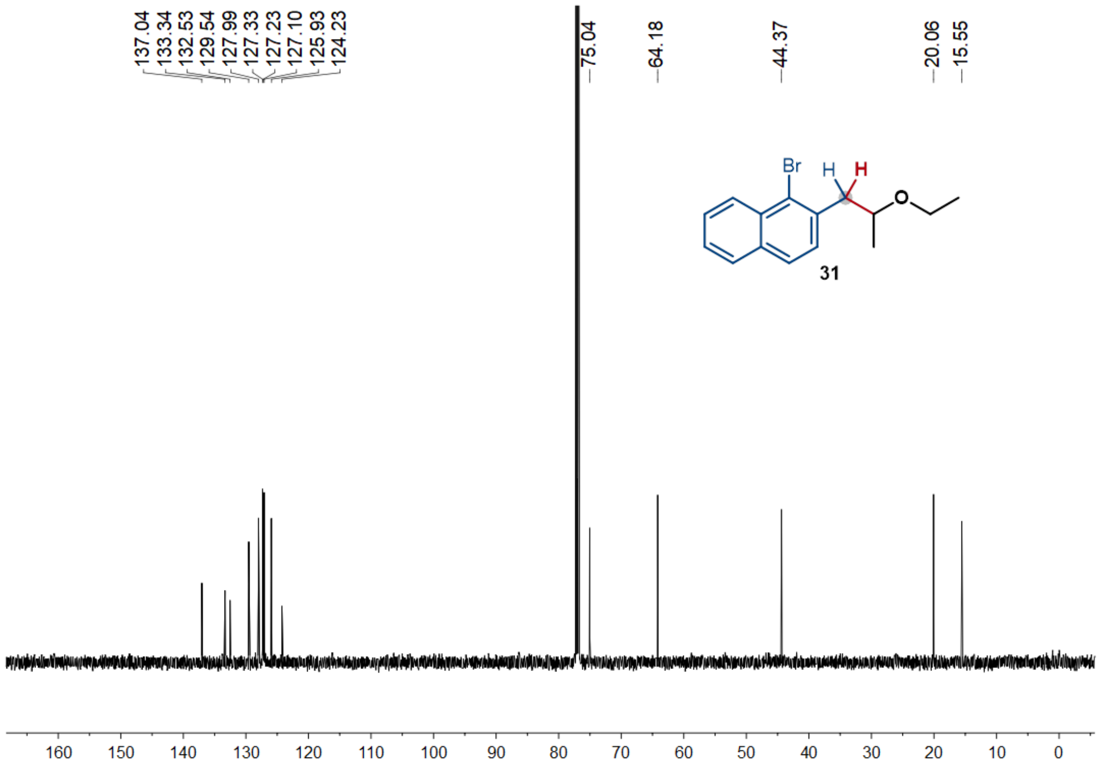

Supplementary Fig. 76 ^{13}**C NMR** (151 MHz, $CDCl_3$) spectrum of compound **31**

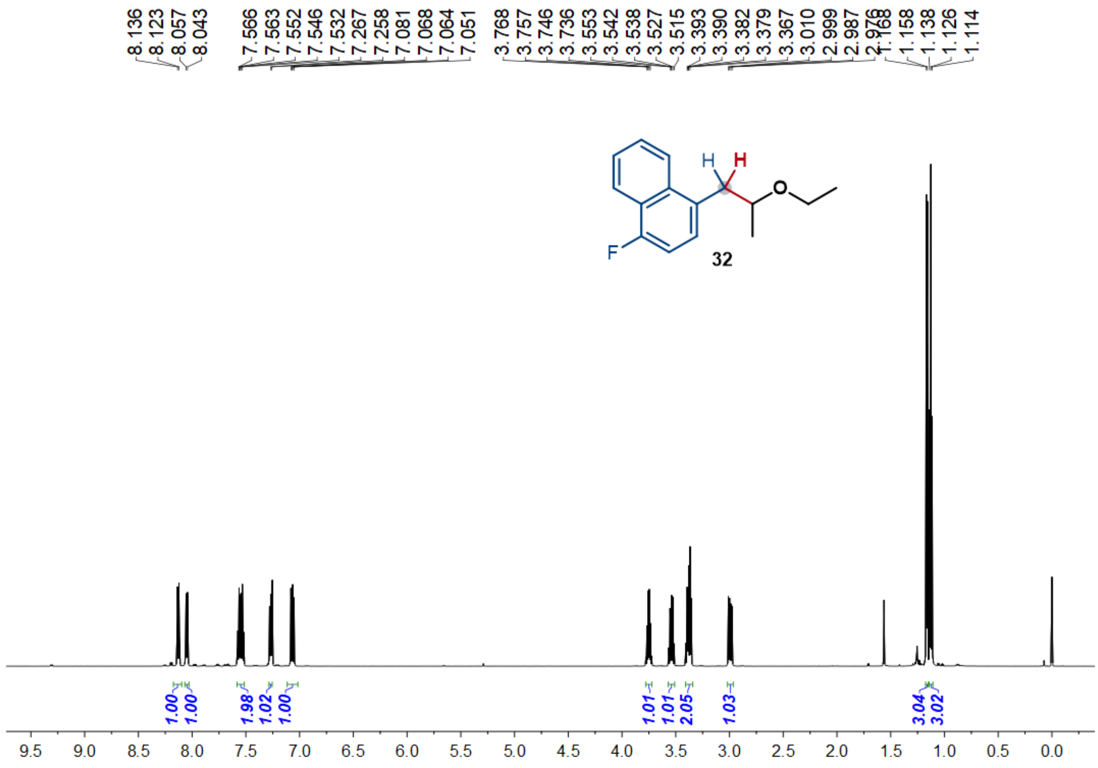

Supplementary Fig. 77 ^{1}**H NMR** (600 MHz, $CDCl_3$) spectrum of compound **32**

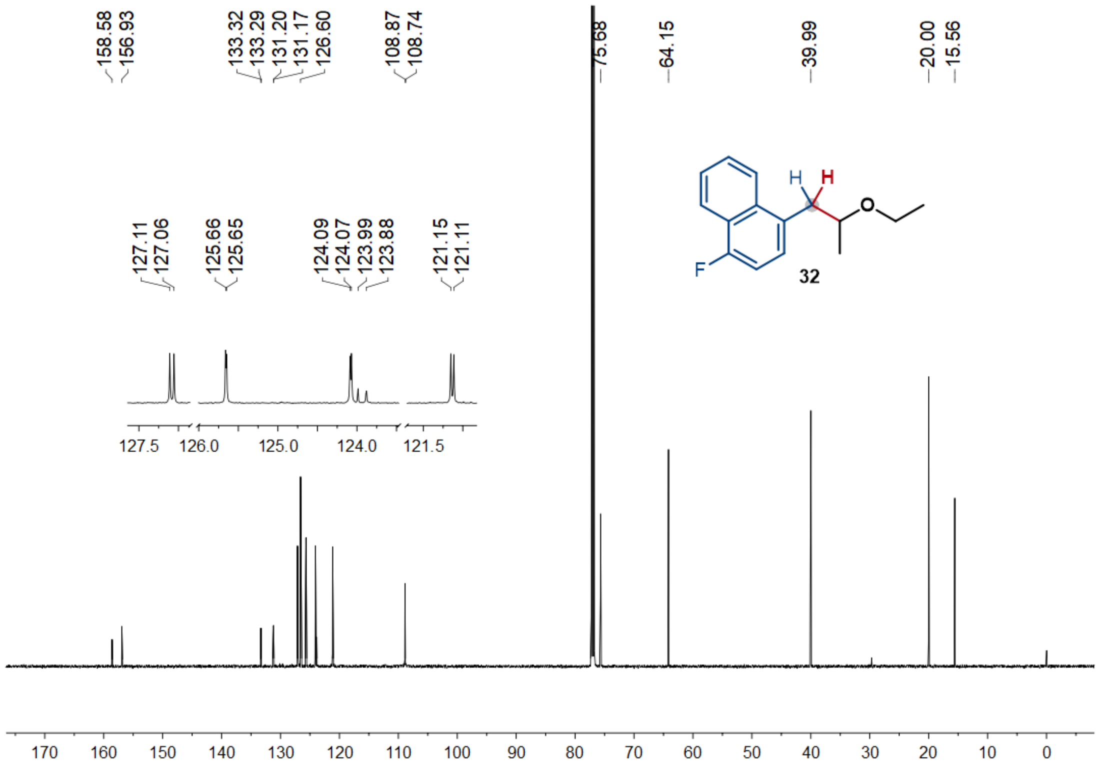

Supplementary Fig. 78 ^{13}**C NMR** (151 MHz, $CDCl_3$) spectrum of compound **32**

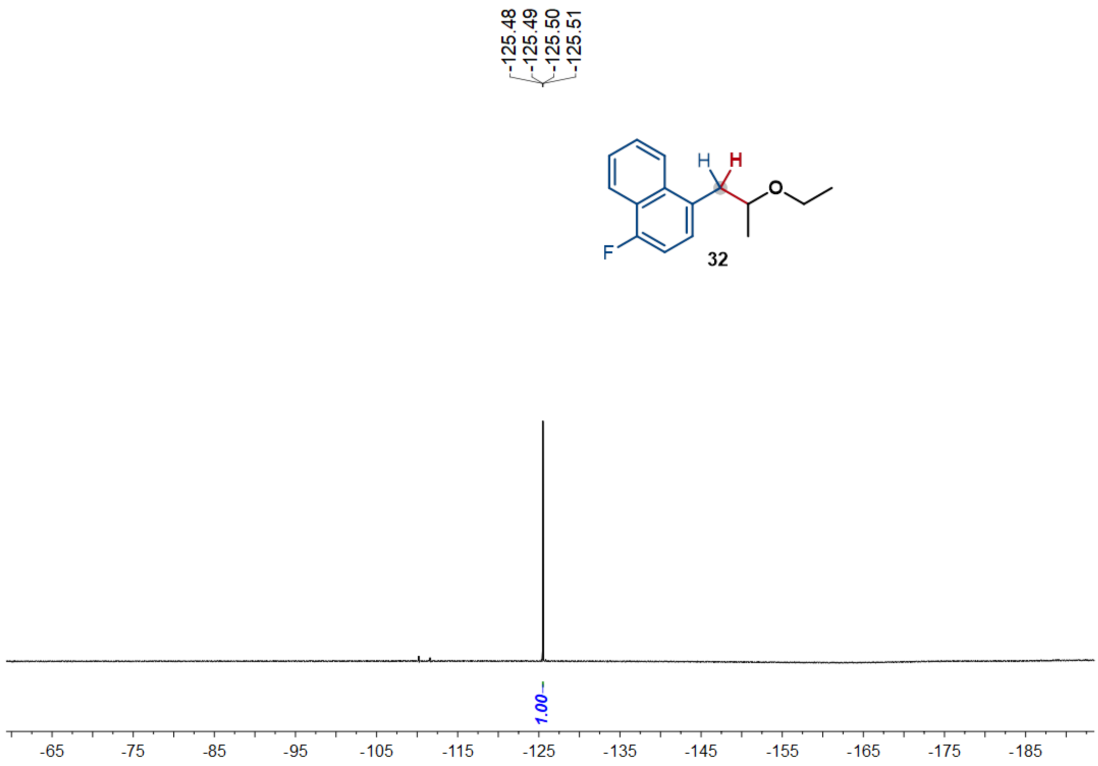

Supplementary Fig. 79 ^{19}**F NMR** (471 MHz, $CDCl_3$) spectrum of compound **32**

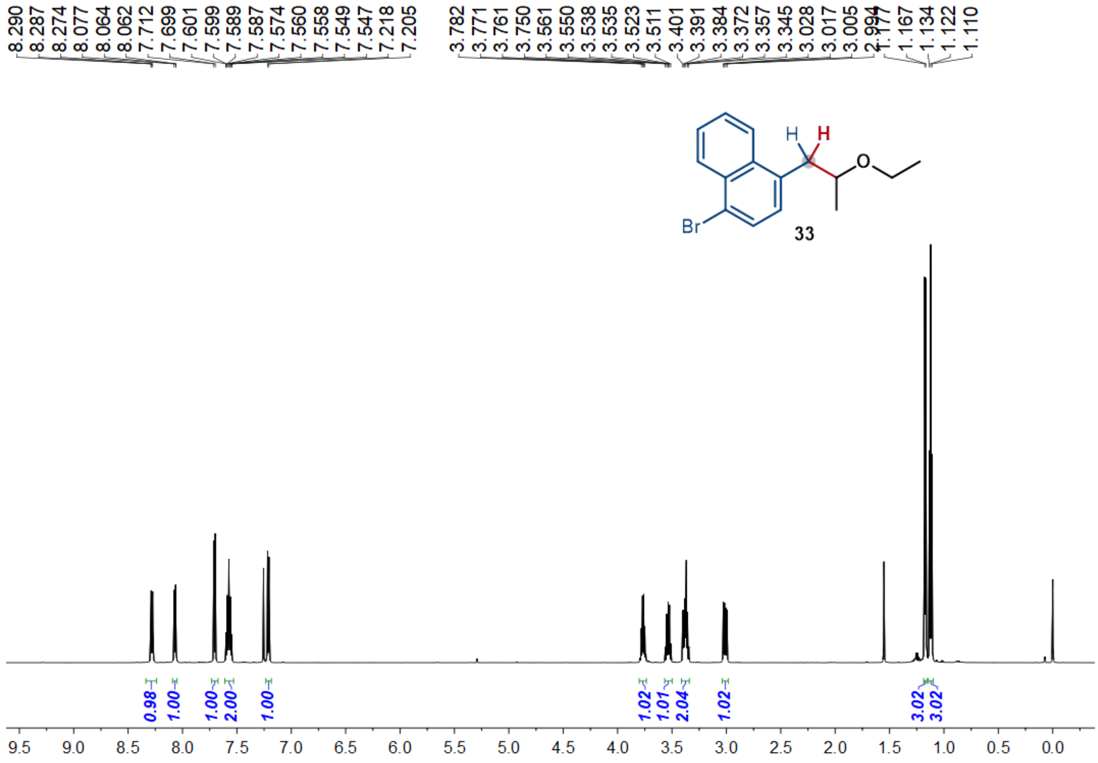

Supplementary Fig. 80 ^{1}**H NMR** (600 MHz, $CDCl_3$) spectrum of compound **33**

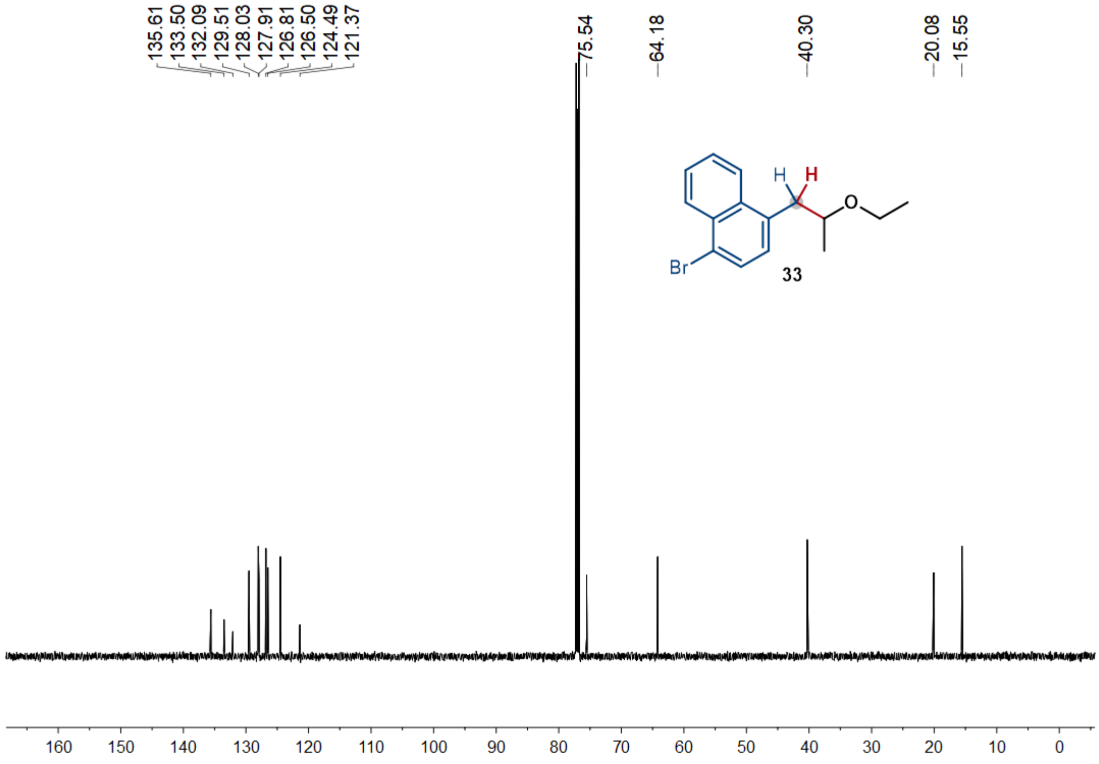

Supplementary Fig. 81 ^{13}**C NMR** (151 MHz, $CDCl_3$) spectrum of compound **33**

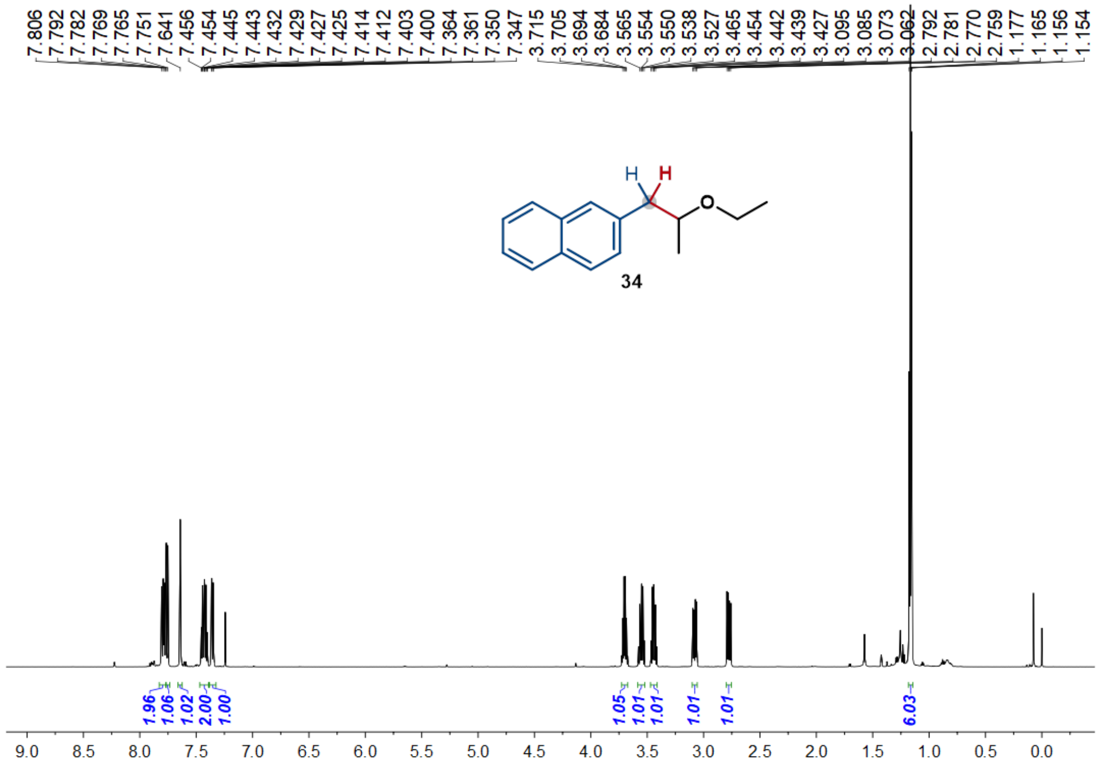

Supplementary Fig. 82 ^{1}H NMR (600 MHz, $CDCl_3$) spectrum of compound **34**

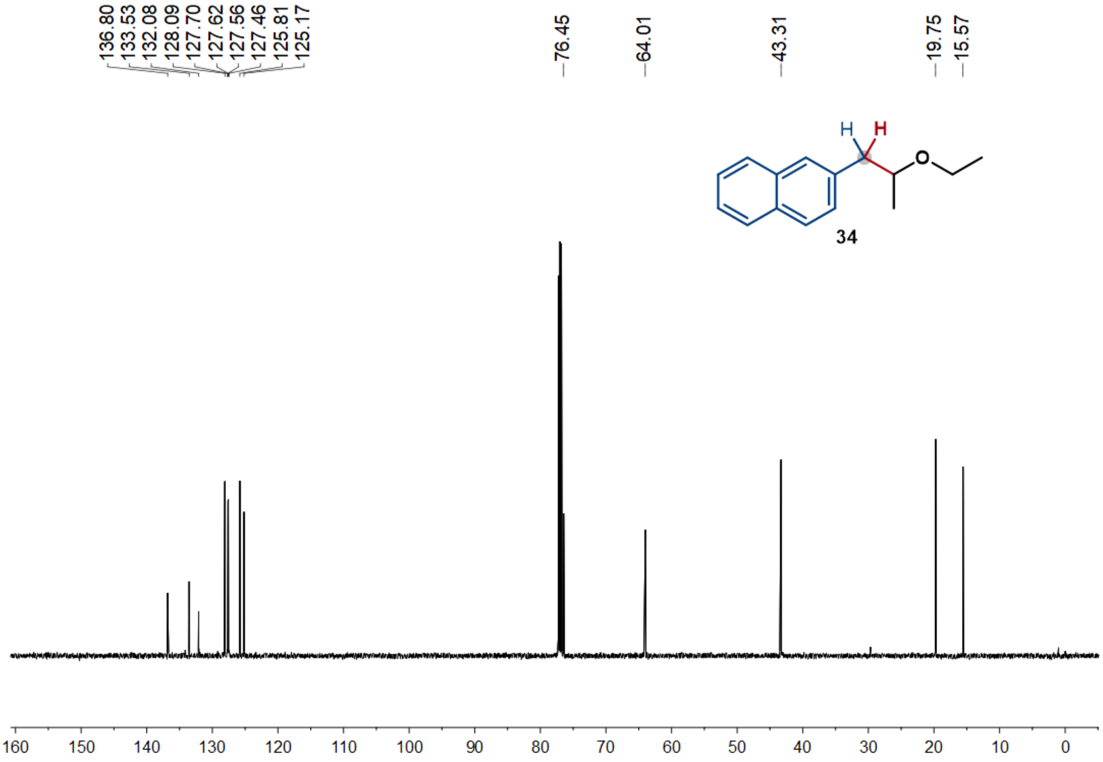

Supplementary Fig. 83 ^{13}C NMR (151 MHz, $CDCl_3$) spectrum of compound **34**

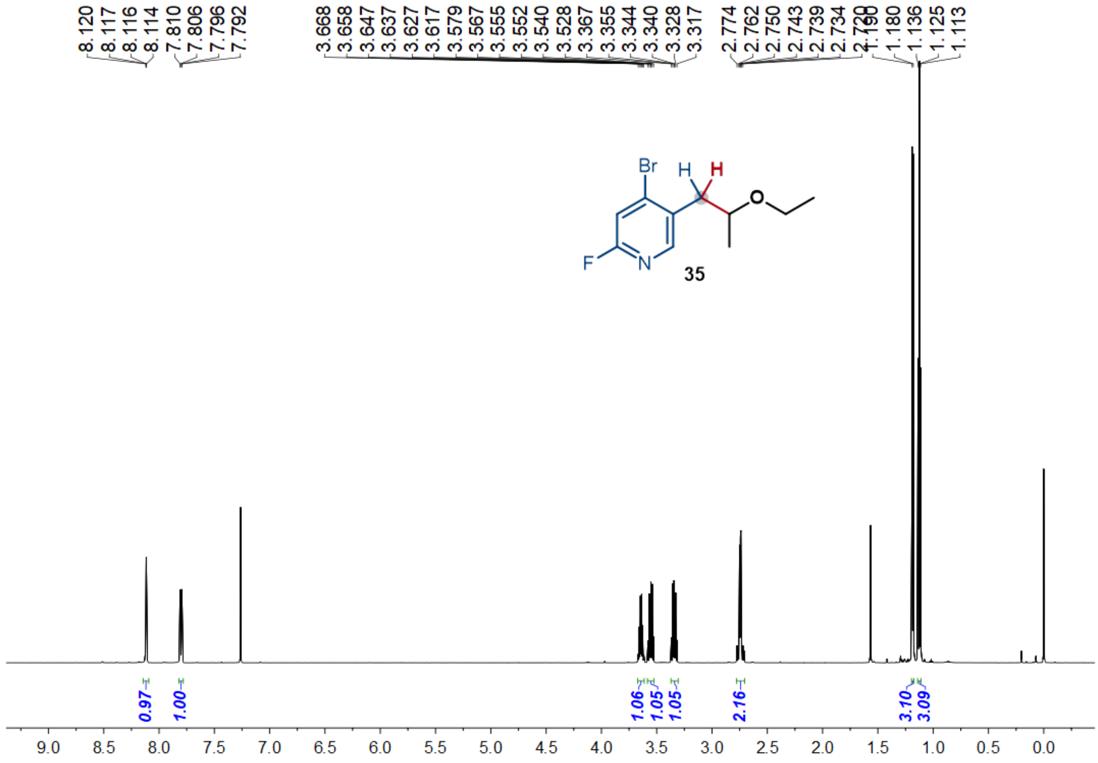

Supplementary Fig. 84 ^{1}H NMR (600 MHz, $CDCl_3$) spectrum of compound **35**

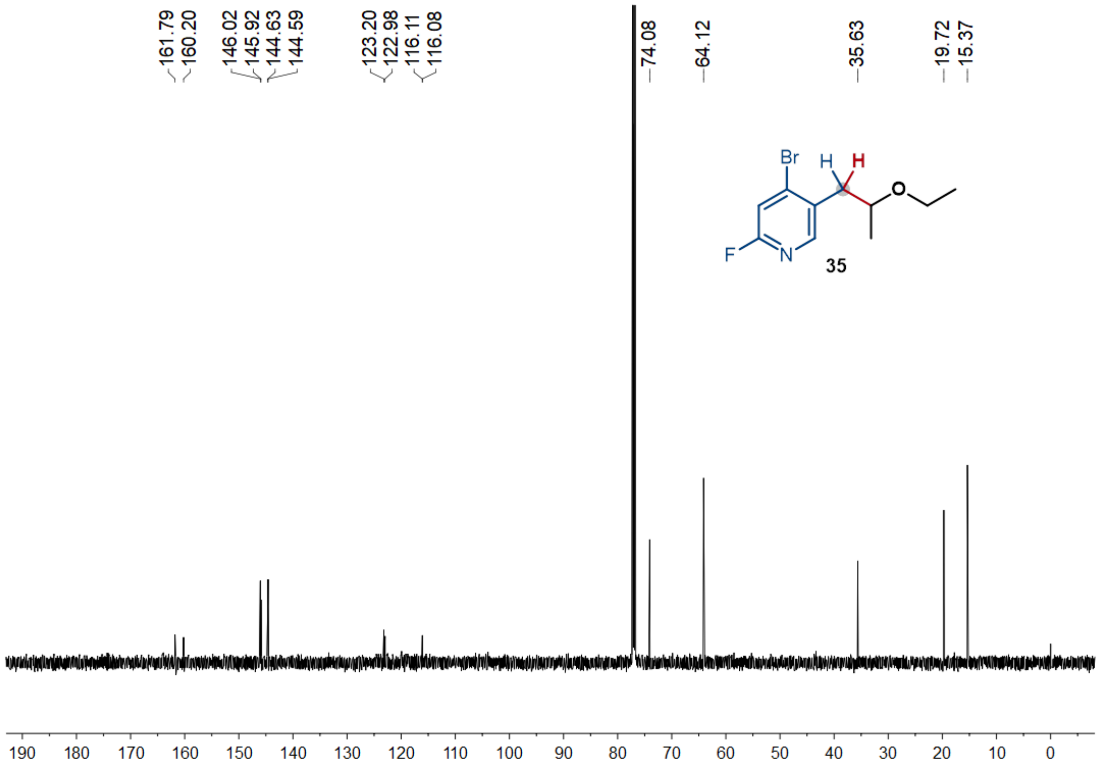

Supplementary Fig. 85 ^{13}C NMR (151 MHz, $CDCl_3$) spectrum of compound **35**

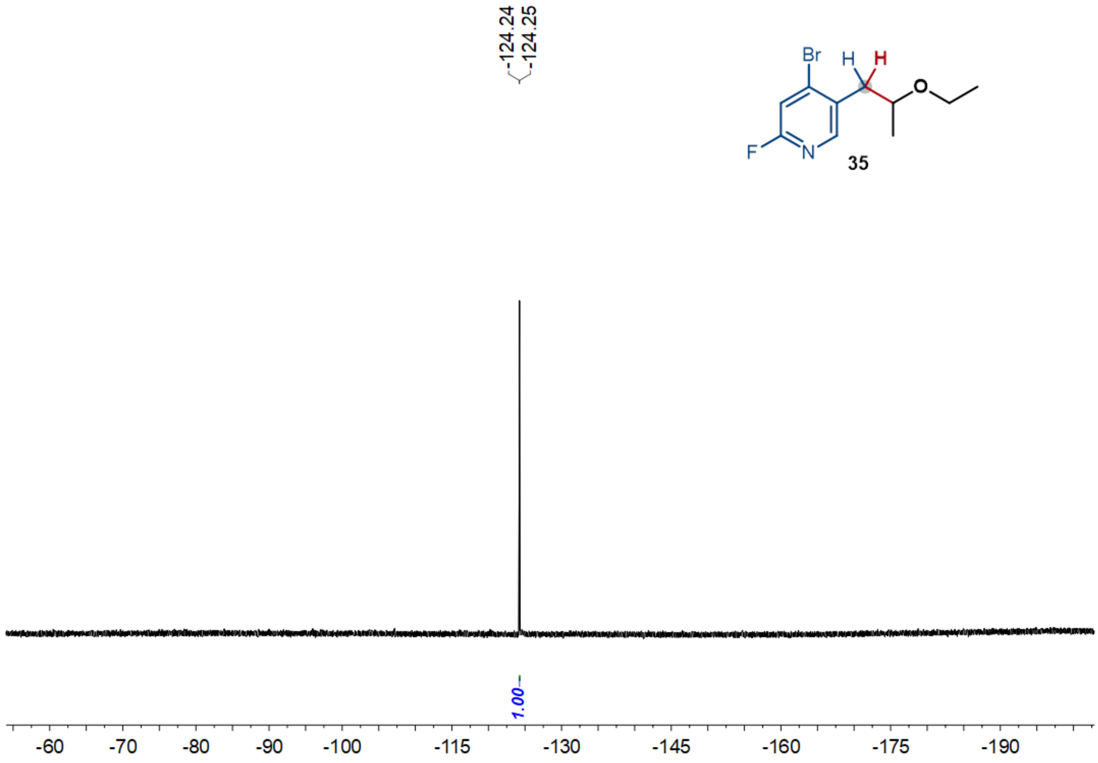

Supplementary Fig. 86 ^{19}**F NMR** (471 MHz, $CDCl_3$) spectrum of compound **35**

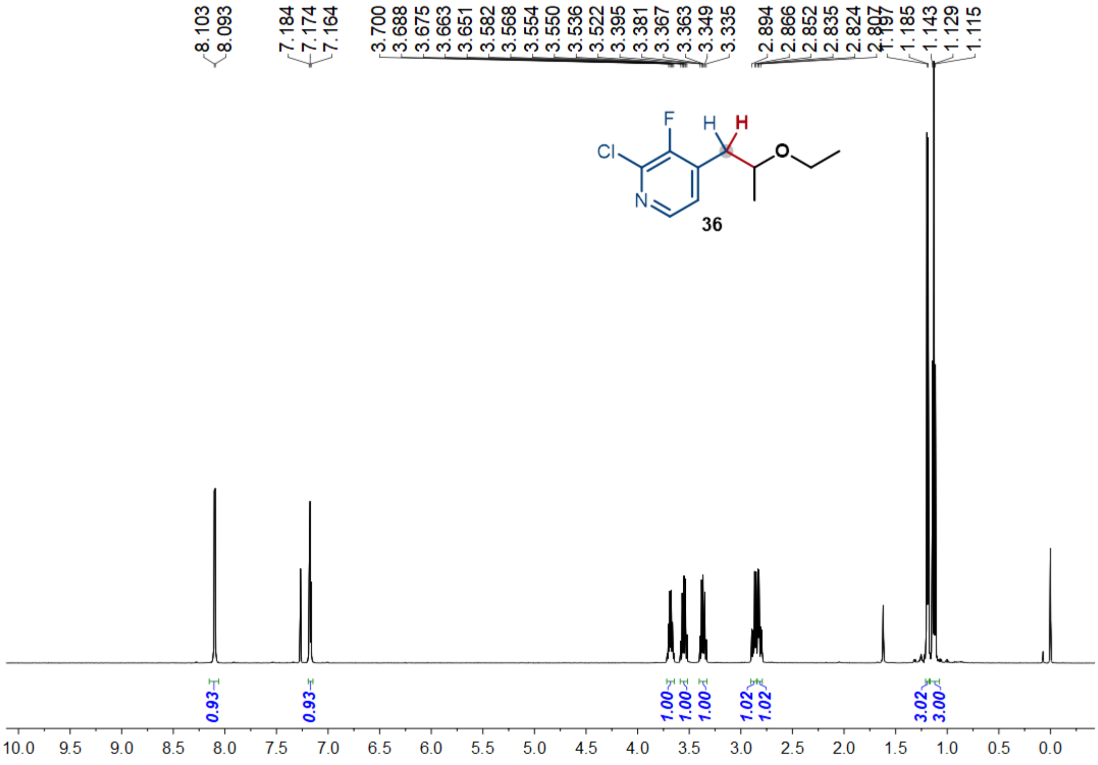

Supplementary Fig. 87 ^{1}**H NMR** (500 MHz, $CDCl_3$) spectrum of compound **36**

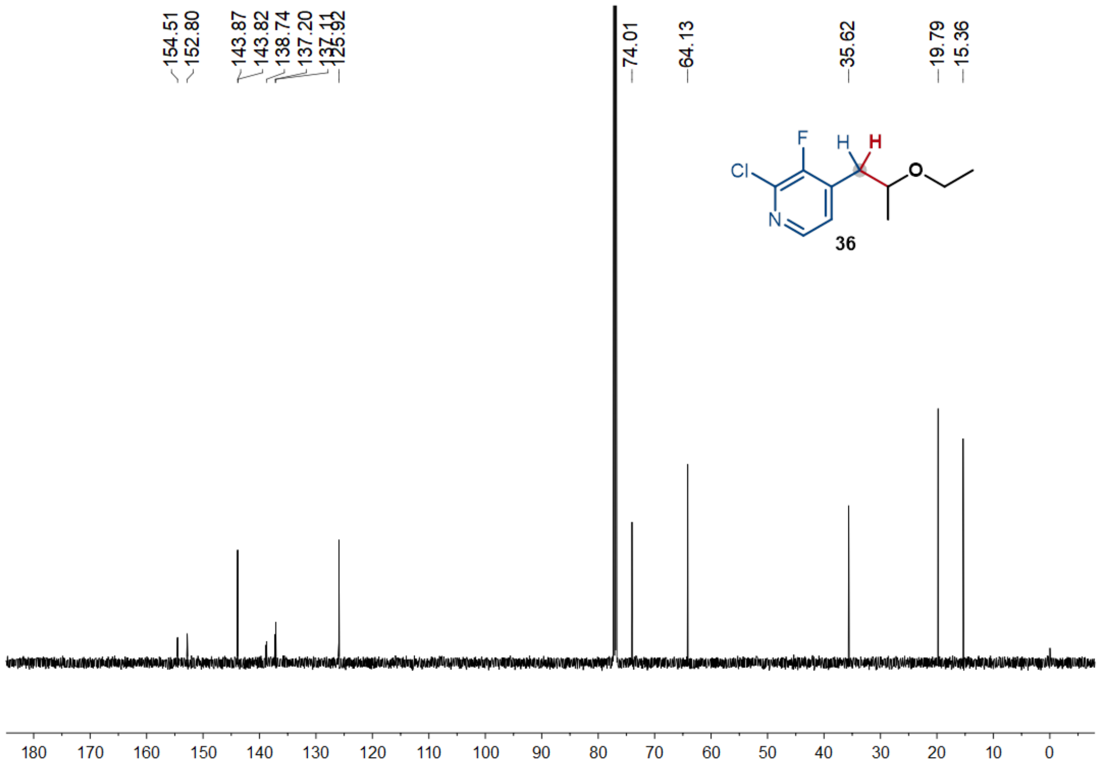

Supplementary Fig. 88 ^{13}C NMR (151 MHz, $CDCl_3$) spectrum of compound **36**

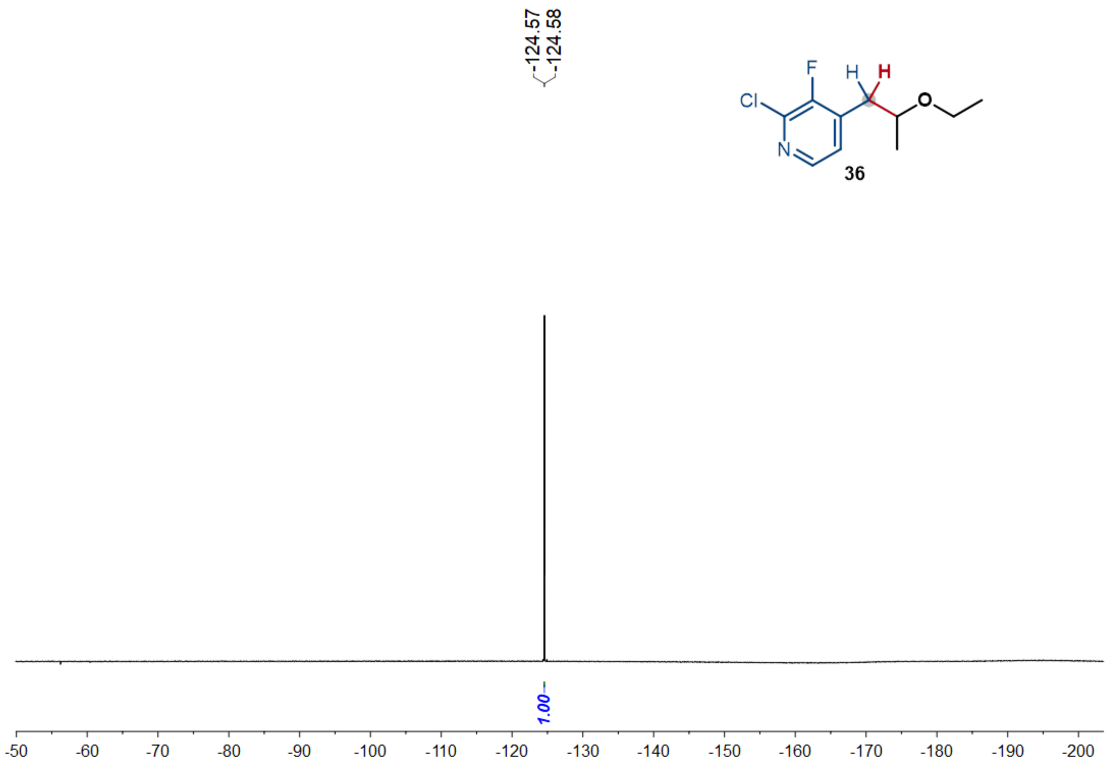

Supplementary Fig. 89 ^{19}F NMR (471 MHz, $CDCl_3$) spectrum of compound **36**

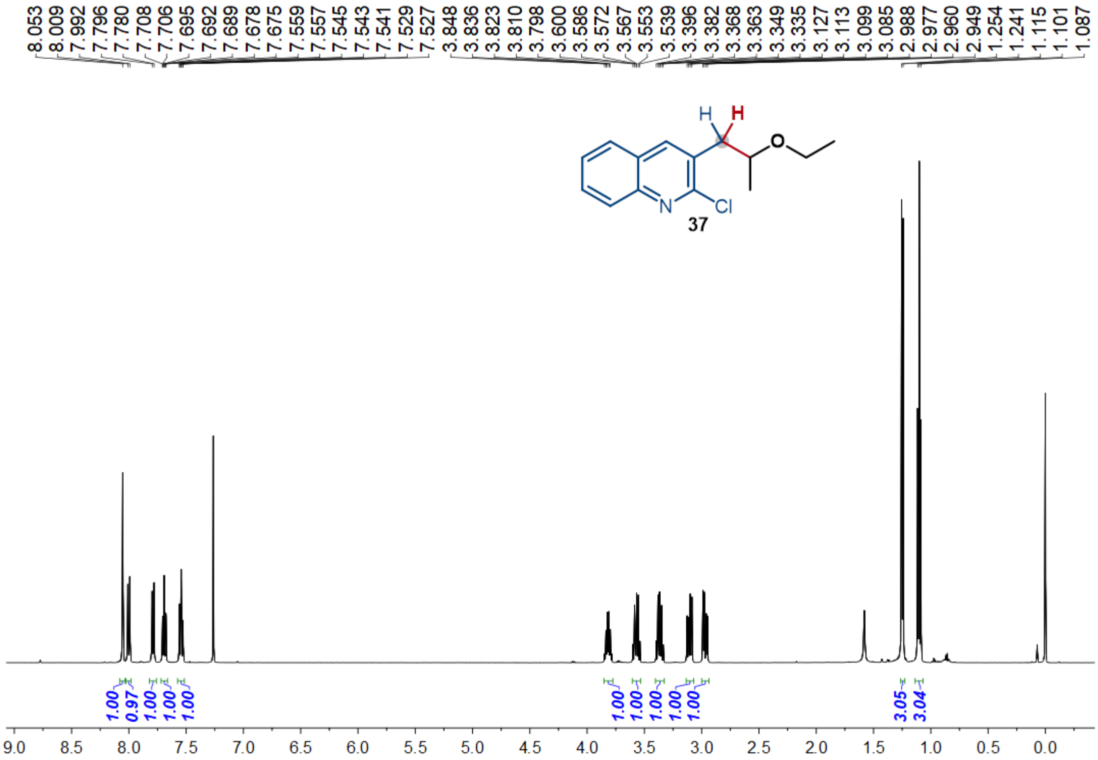

Supplementary Fig. 90 ^{1}**H NMR** (500 MHz, $CDCl_3$) spectrum of compound **37**

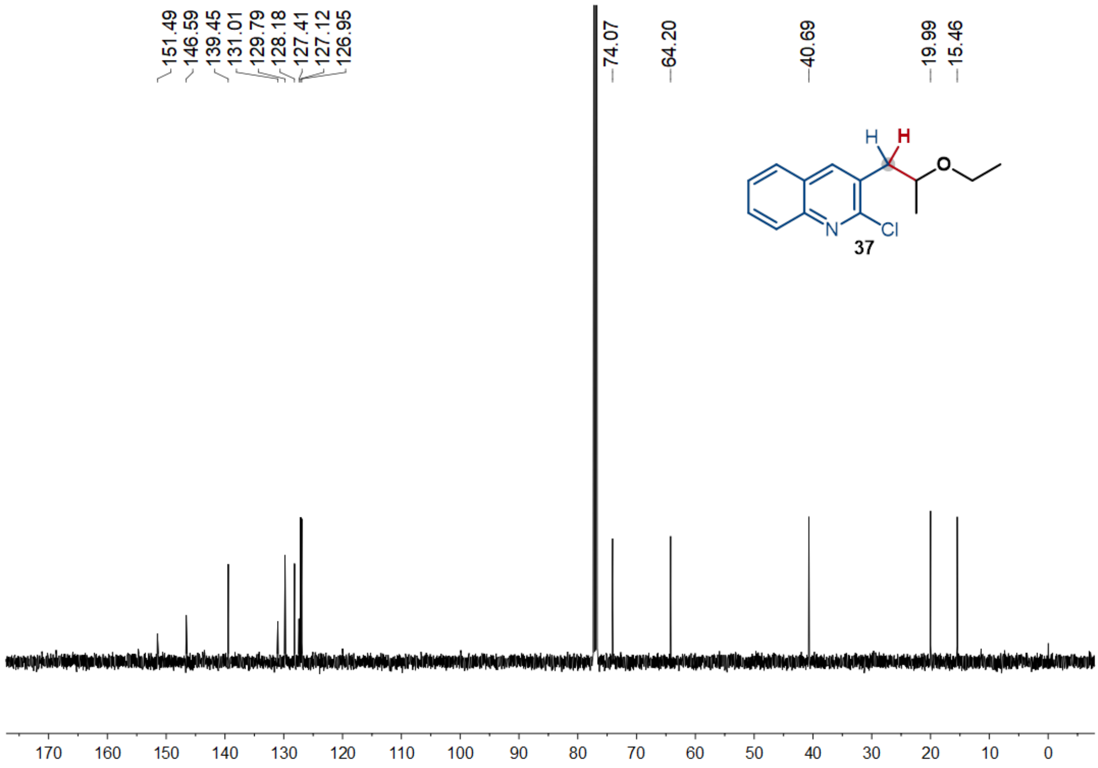

Supplementary Fig. 91 ^{13}**C NMR** (126 MHz, $CDCl_3$) spectrum of compound **37**

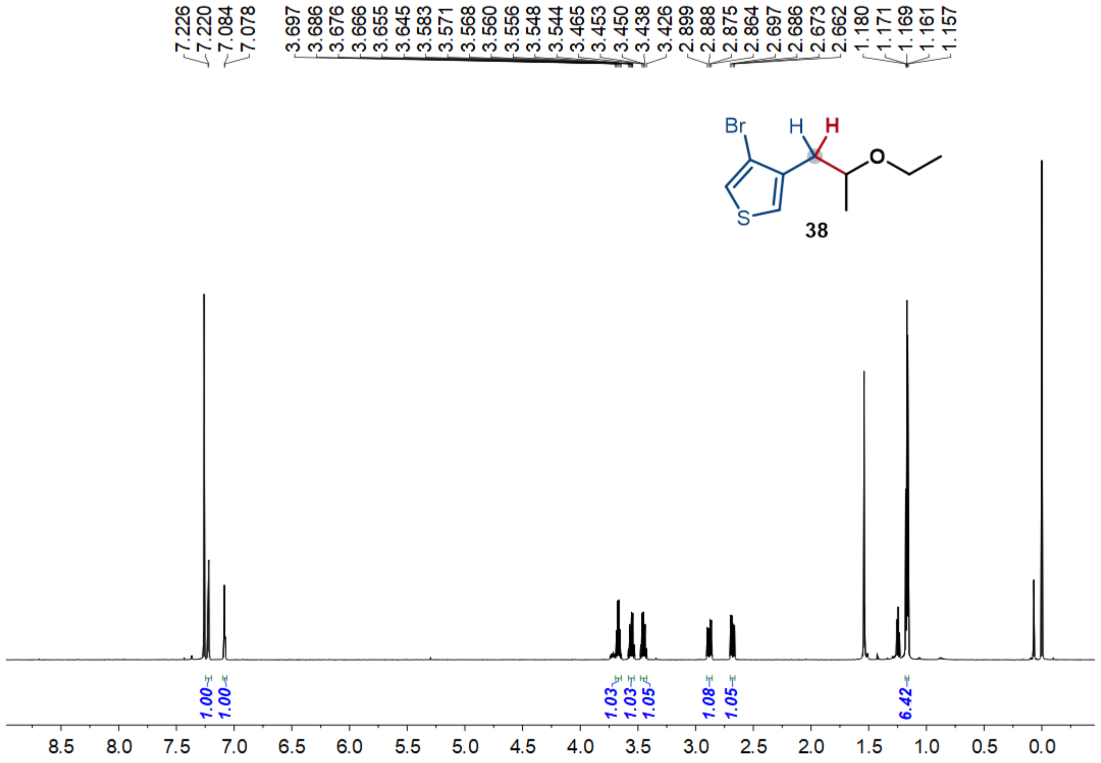

Supplementary Fig. 92 ^{1}**H NMR** (600 MHz, $CDCl_3$) spectrum of compound **38**

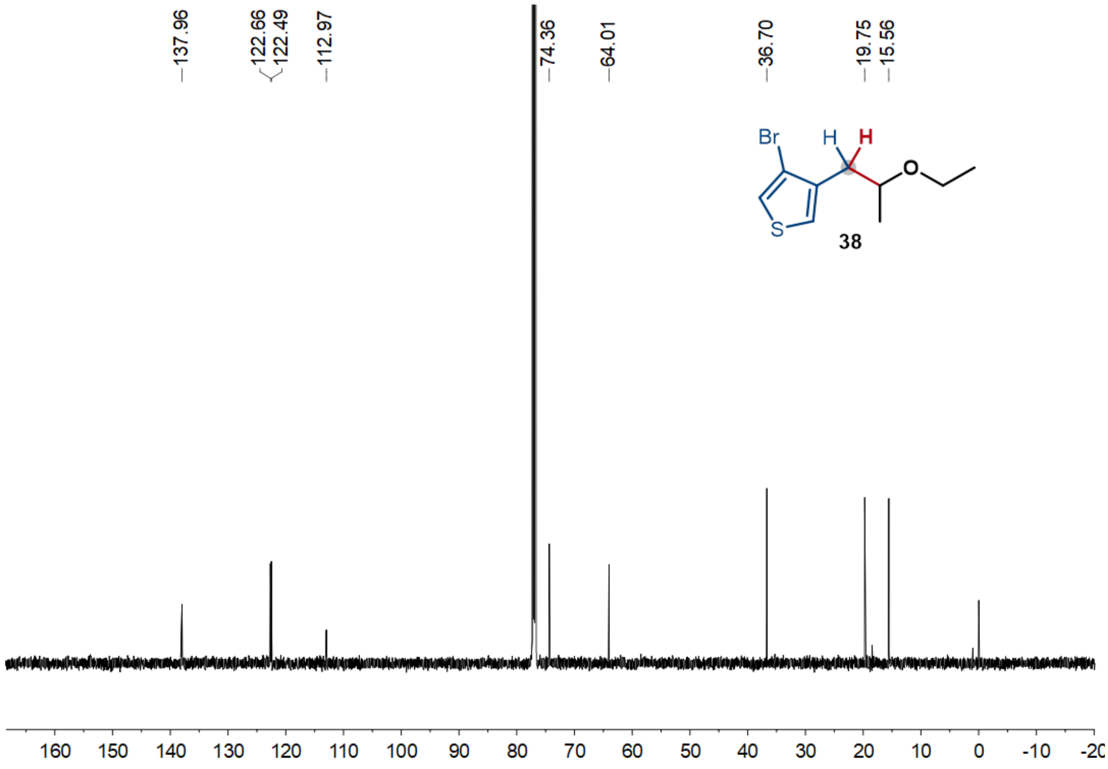

Supplementary Fig. 93 ^{13}**C NMR** (151 MHz, $CDCl_3$) spectrum of compound **38**

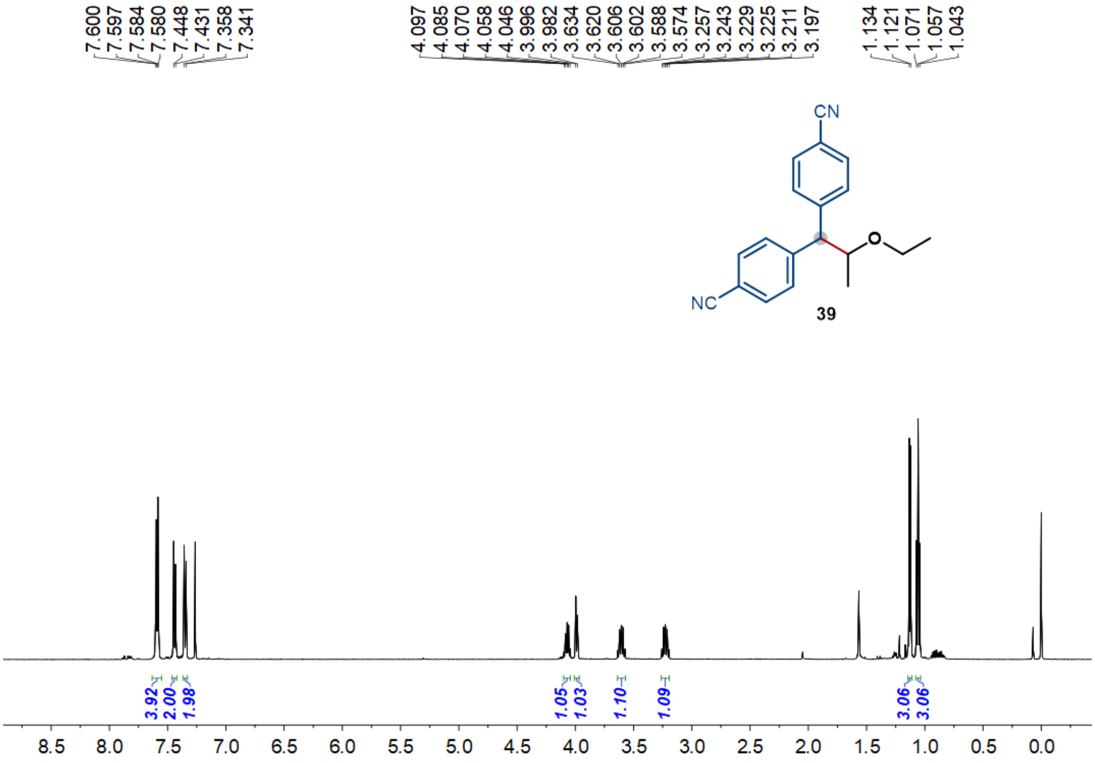

Supplementary Fig. 94 ^{1}**H NMR** (500 MHz, $CDCl_3$) spectrum of compound **39**

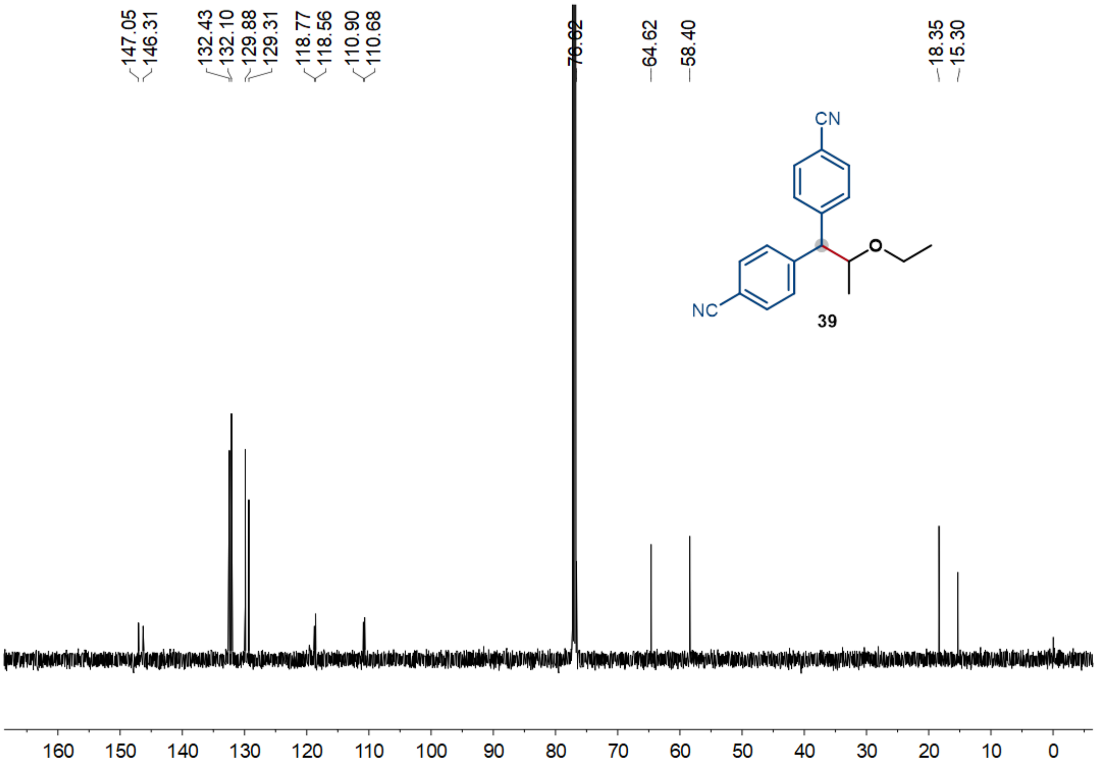

Supplementary Fig. 95 ^{13}**C NMR** (151 MHz, $CDCl_3$) spectrum of compound **39**

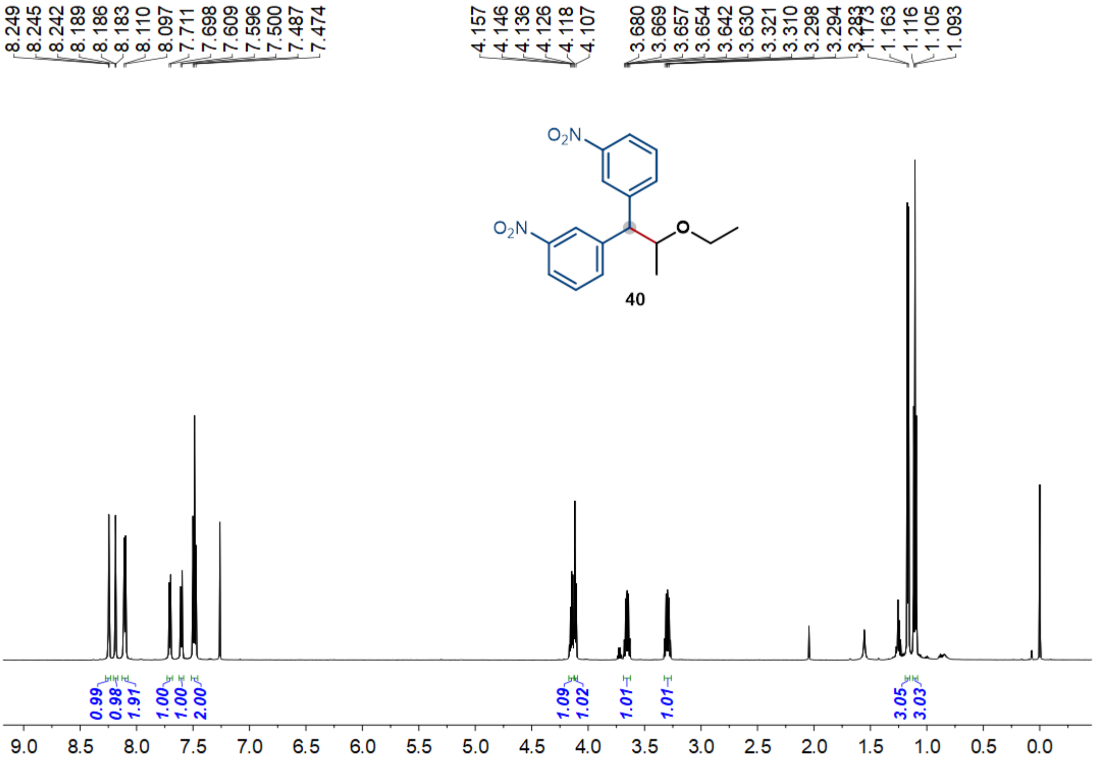

Supplementary Fig. 96 ^{1}H NMR (600 MHz, $CDCl_3$) spectrum of compound **40**

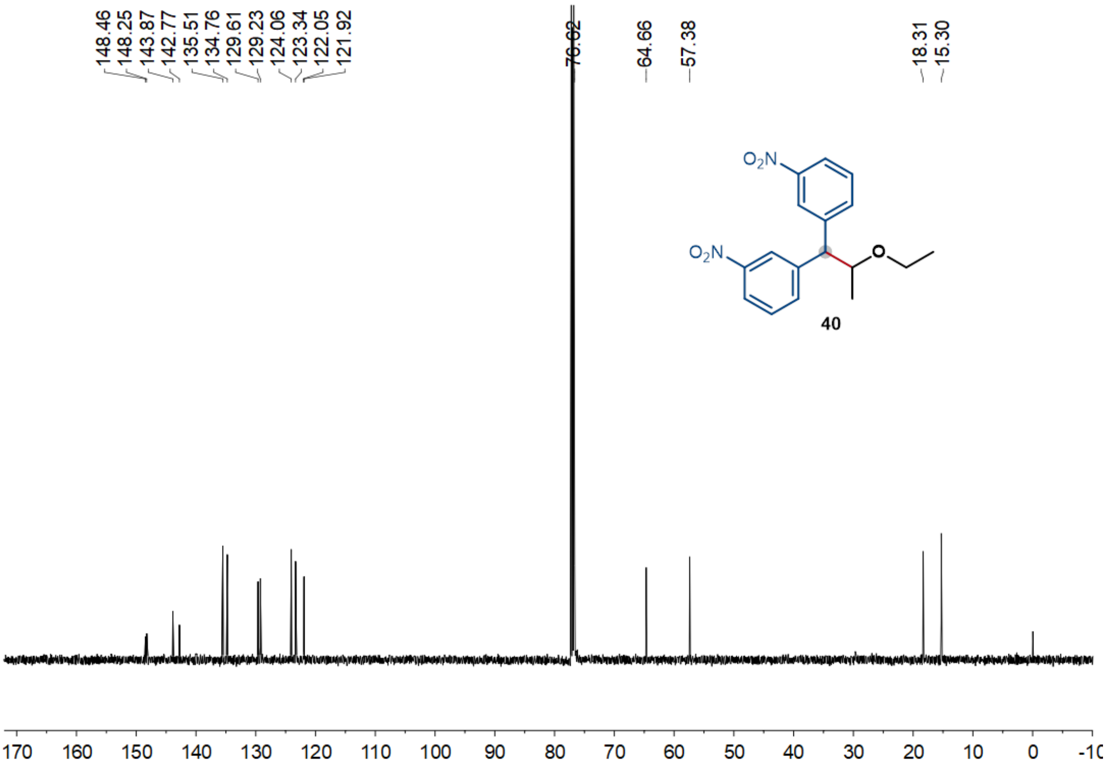

Supplementary Fig. 97 ^{13}C NMR (151 MHz, $CDCl_3$) spectrum of compound **40**

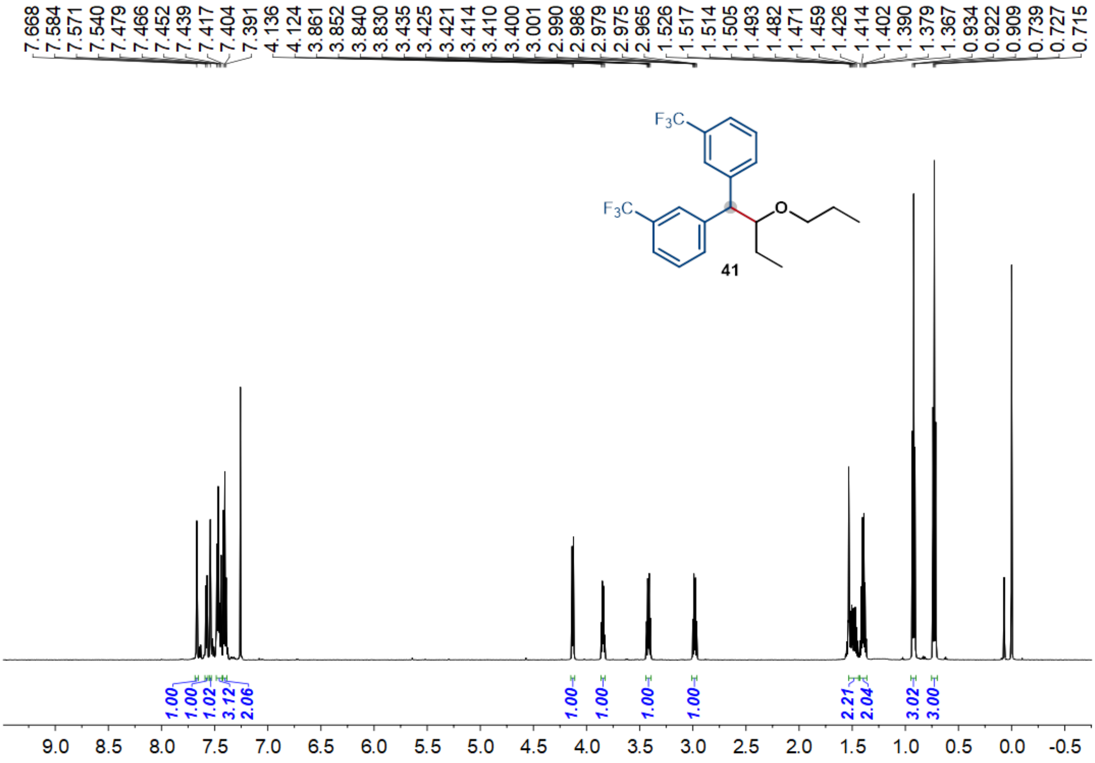

Supplementary Fig. 98 ^{1}H NMR (600 MHz, $CDCl_3$) spectrum of compound **41**

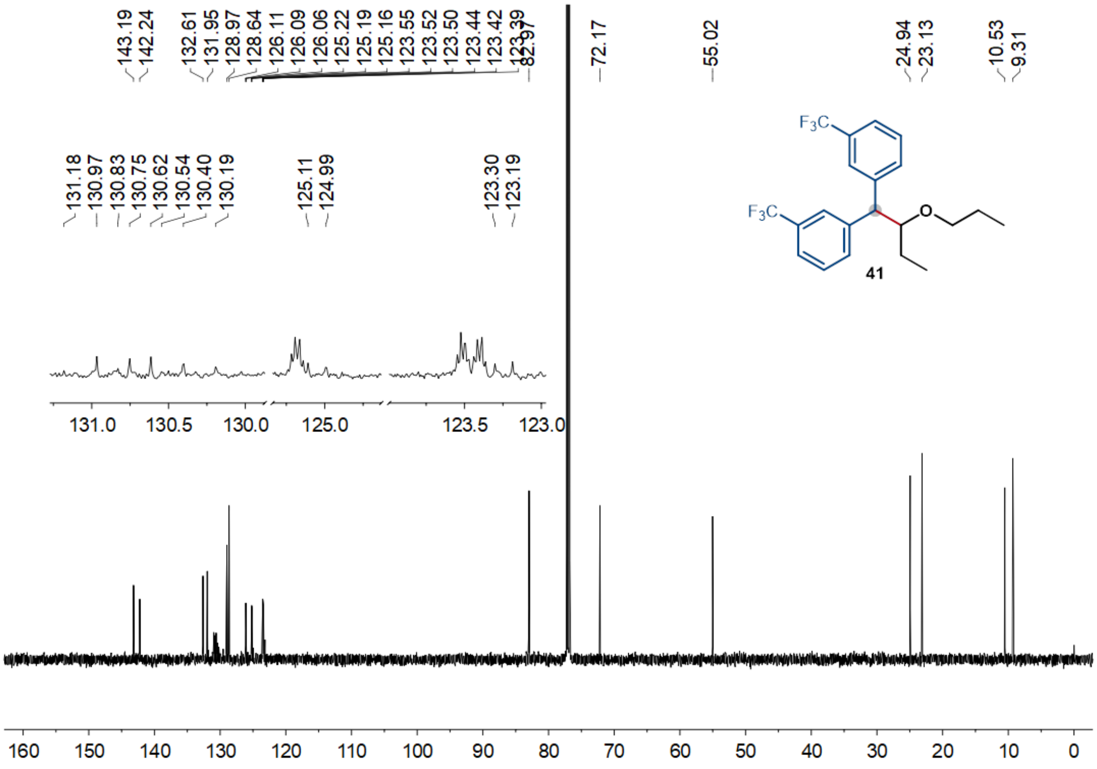

Supplementary Fig. 99 ^{13}C NMR (151 MHz, $CDCl_3$) spectrum of compound **41**

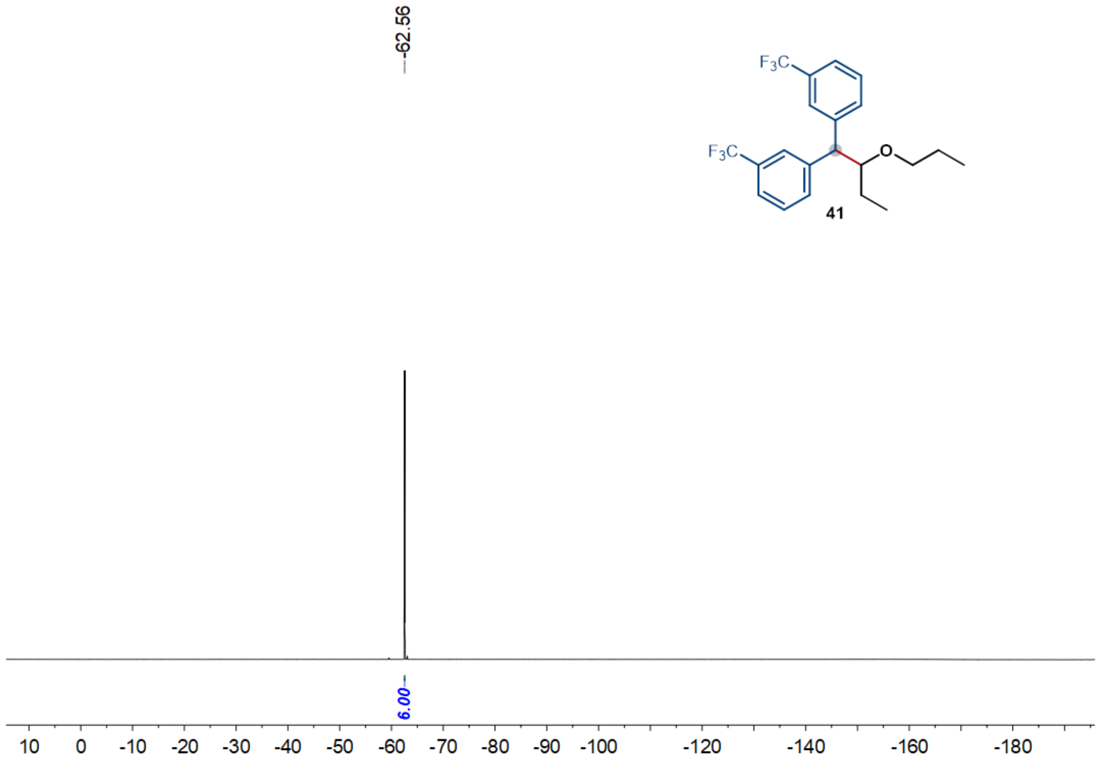

Supplementary Fig. 100 ^{19}F NMR (471 MHz, $CDCl_3$) spectrum of compound **41**

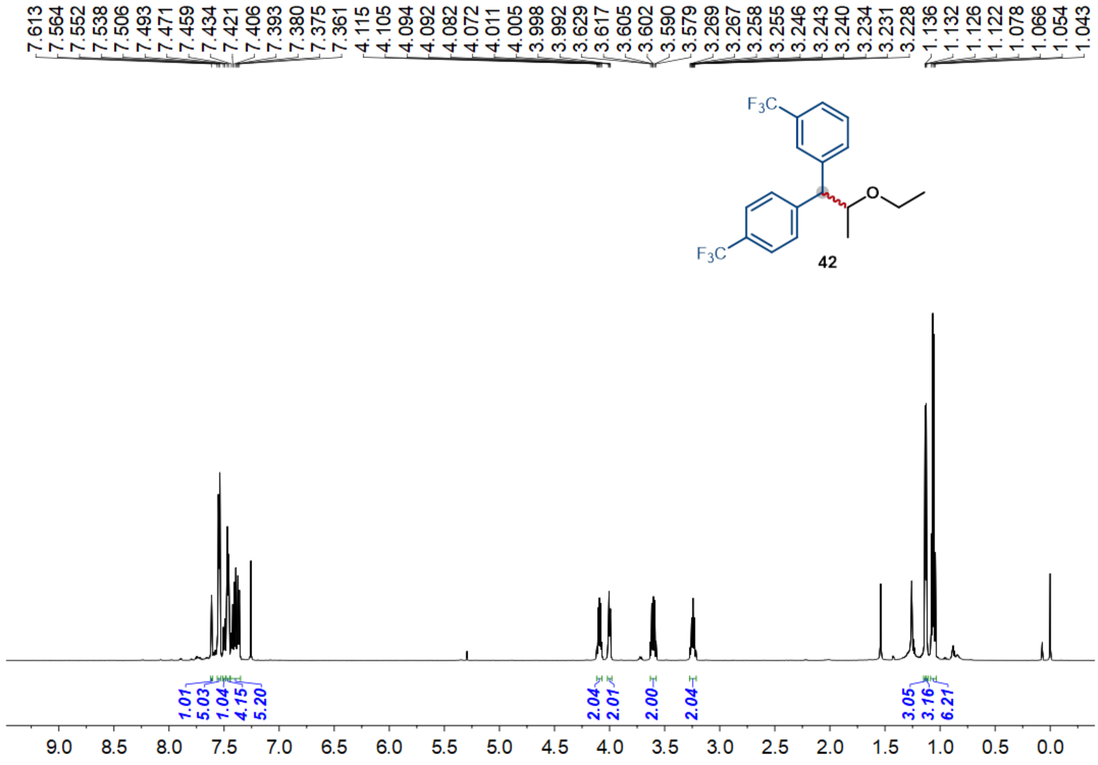

Supplementary Fig. 101 ^{1}H NMR (600 MHz, $CDCl_3$) spectrum of compound **42**

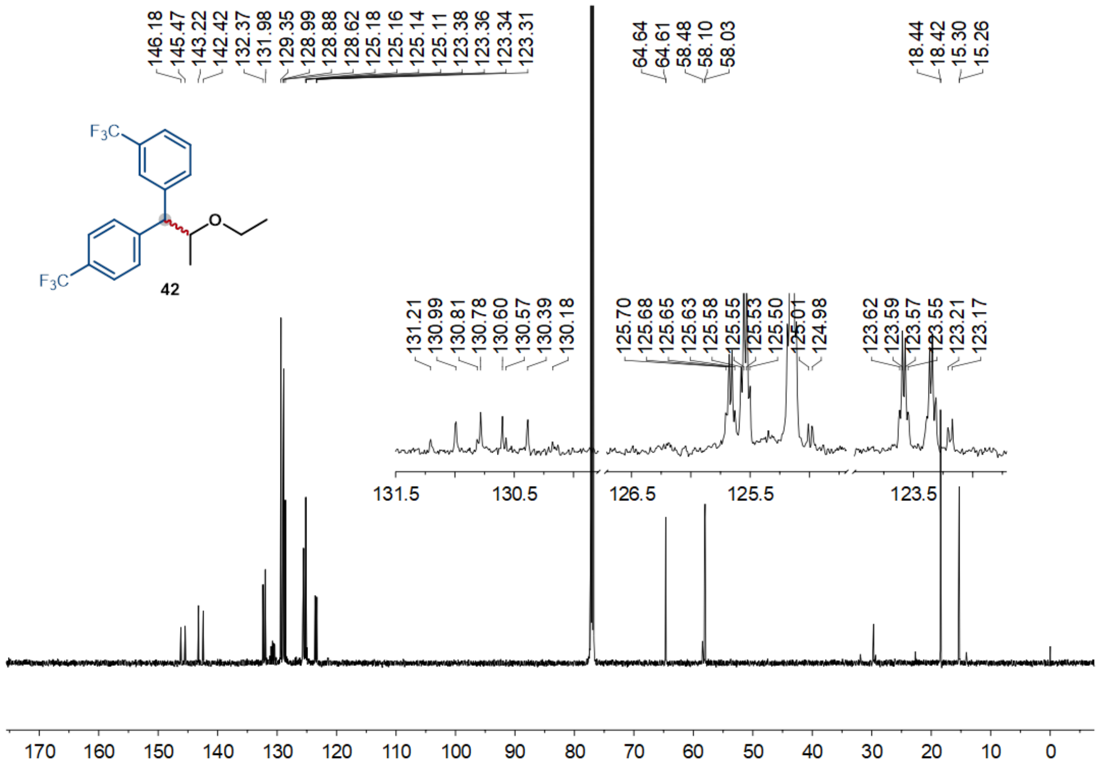

Supplementary Fig. 102 ^{13}**C NMR** (151 MHz, $CDCl_3$) spectrum of compound **42**

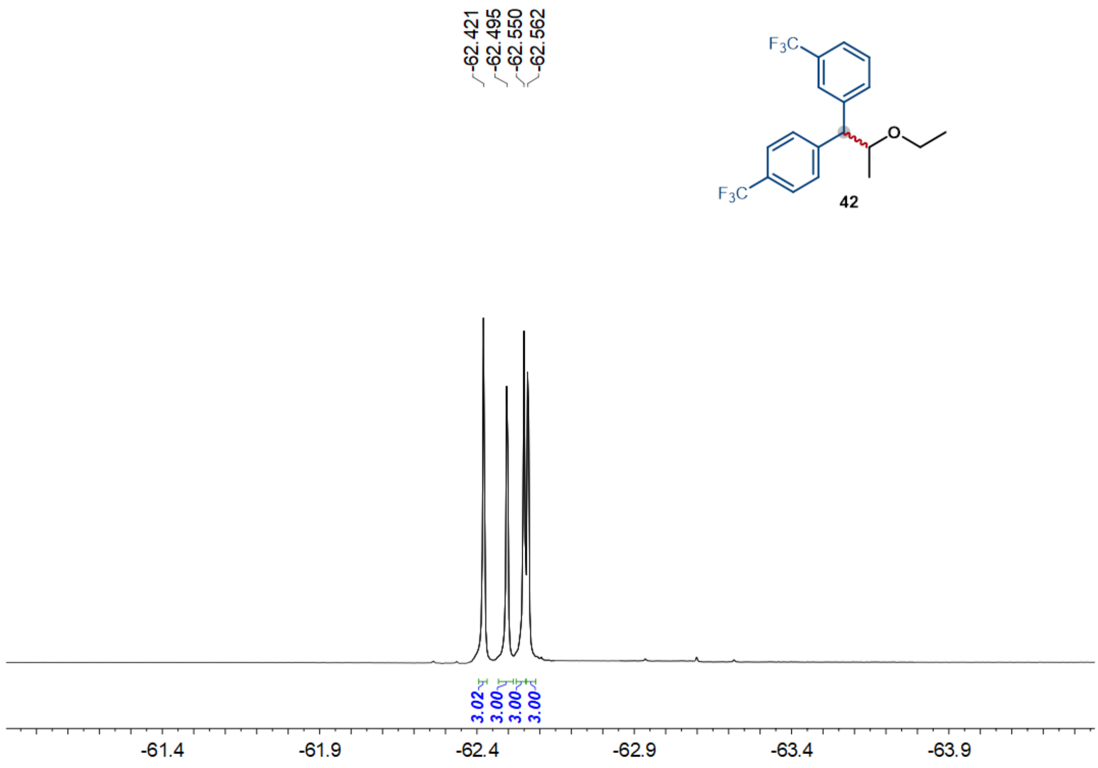

Supplementary Fig. 103 ^{19}**F NMR** (471 MHz, $CDCl_3$) spectrum of compound **42**

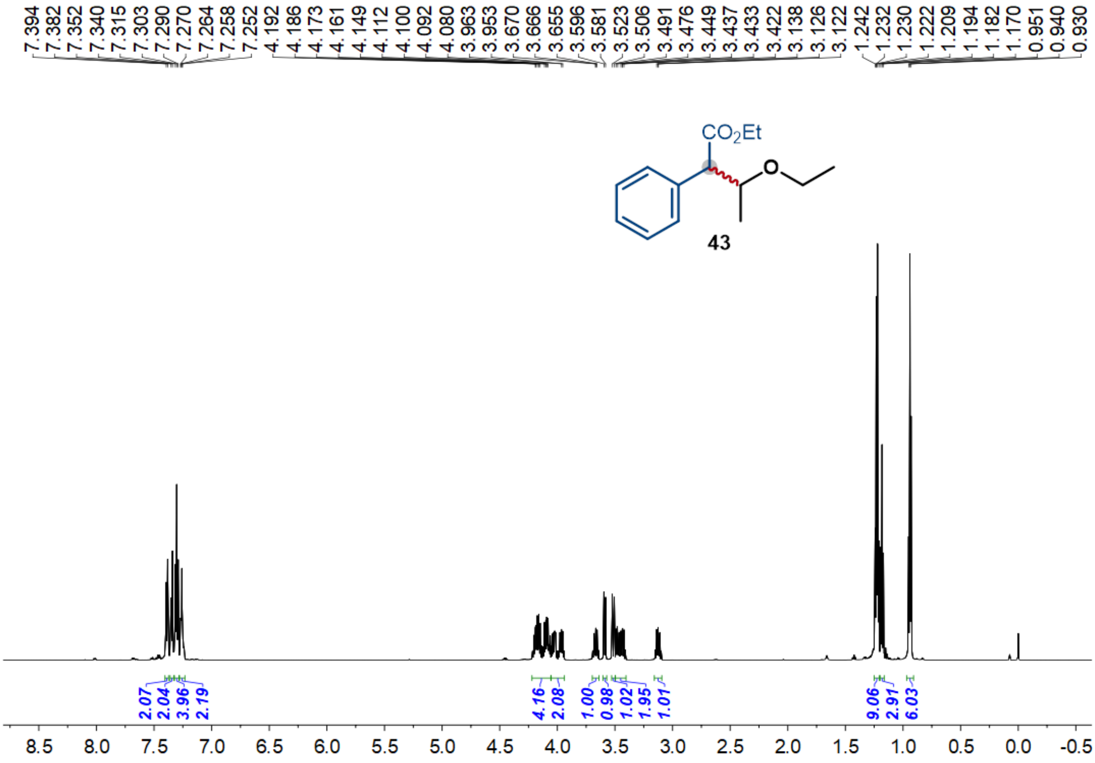

Supplementary Fig. 104 **^{1}H NMR** (600 MHz, $CDCl_3$) spectrum of compound **43**

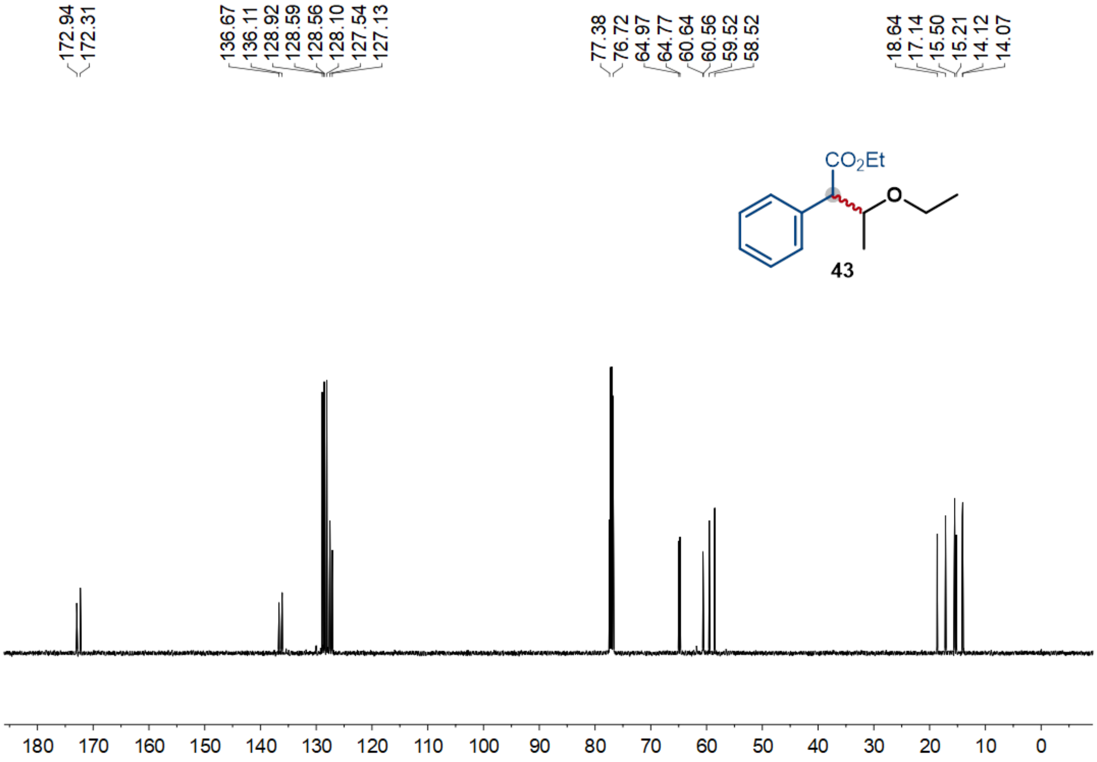

Supplementary Fig. 105 **^{13}C NMR** (151 MHz, $CDCl_3$) spectrum of compound **43**

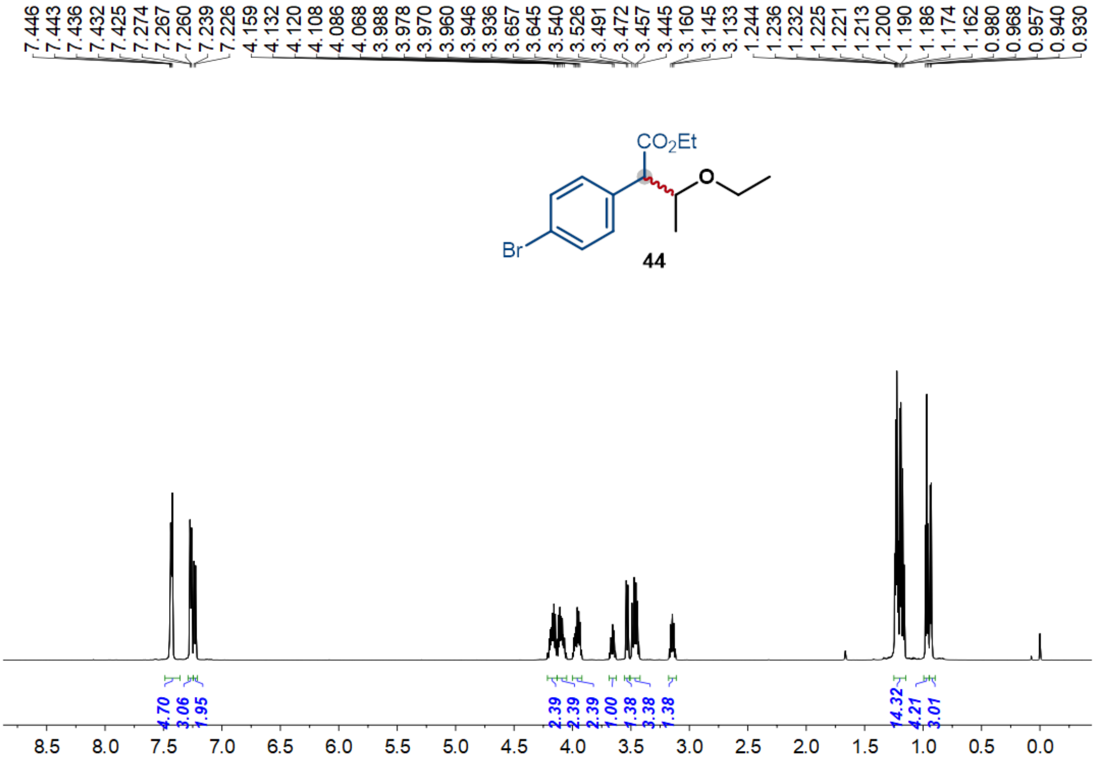

Supplementary Fig. 106 ^{1}H NMR (600 MHz, $CDCl_3$) spectrum of compound **44**

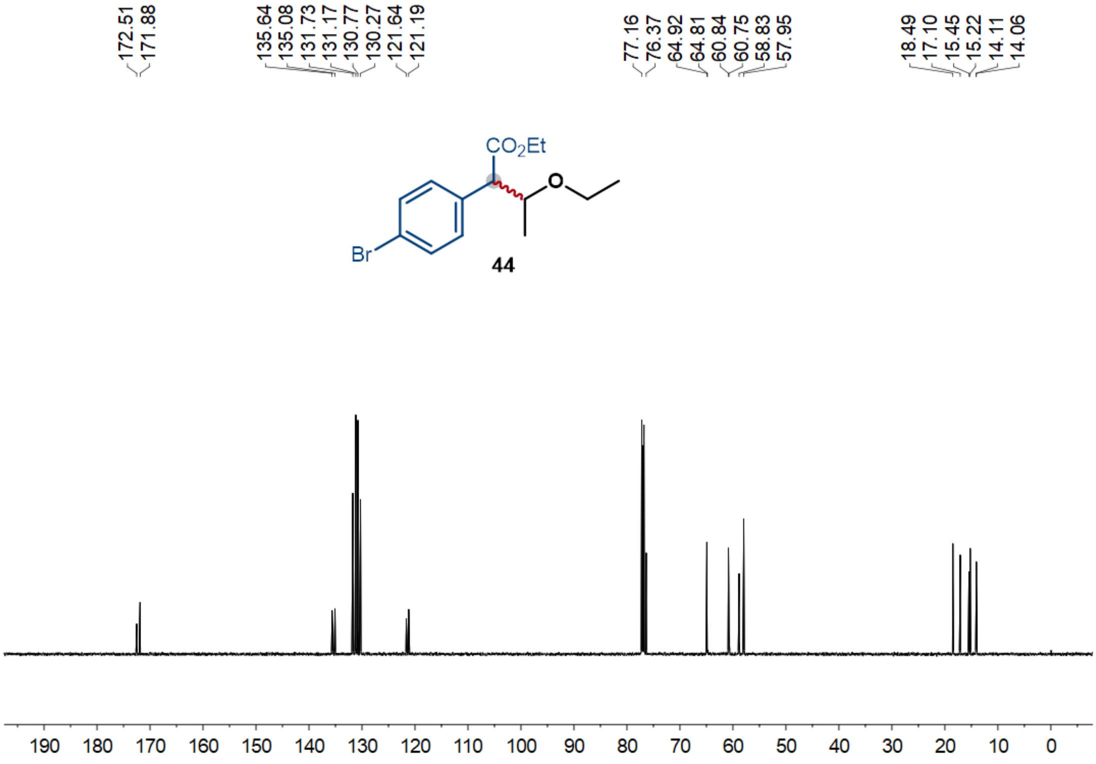

Supplementary Fig. 107 ^{13}C NMR (151 MHz, $CDCl_3$) spectrum of compound **44**

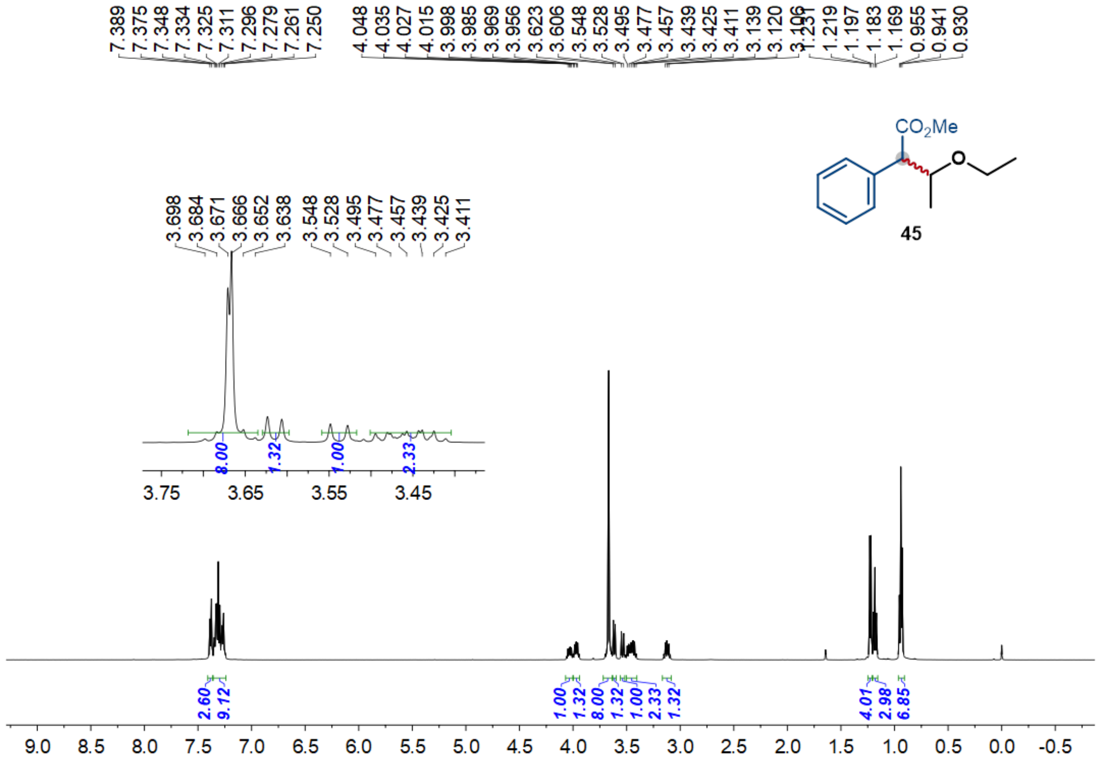

Supplementary Fig. 108 ^{1}**H NMR** (500 MHz, $CDCl_3$) spectrum of compound

45

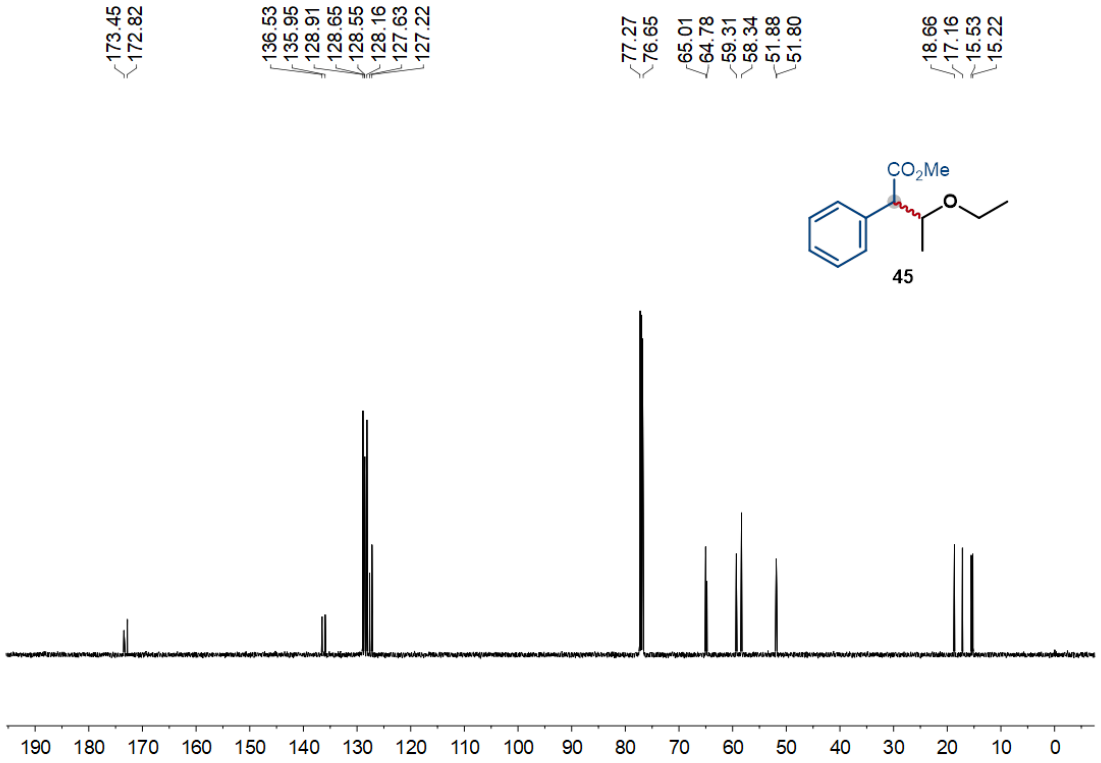

Supplementary Fig. 109 ^{13}**C NMR** (151 MHz, $CDCl_3$) spectrum of compound **45**

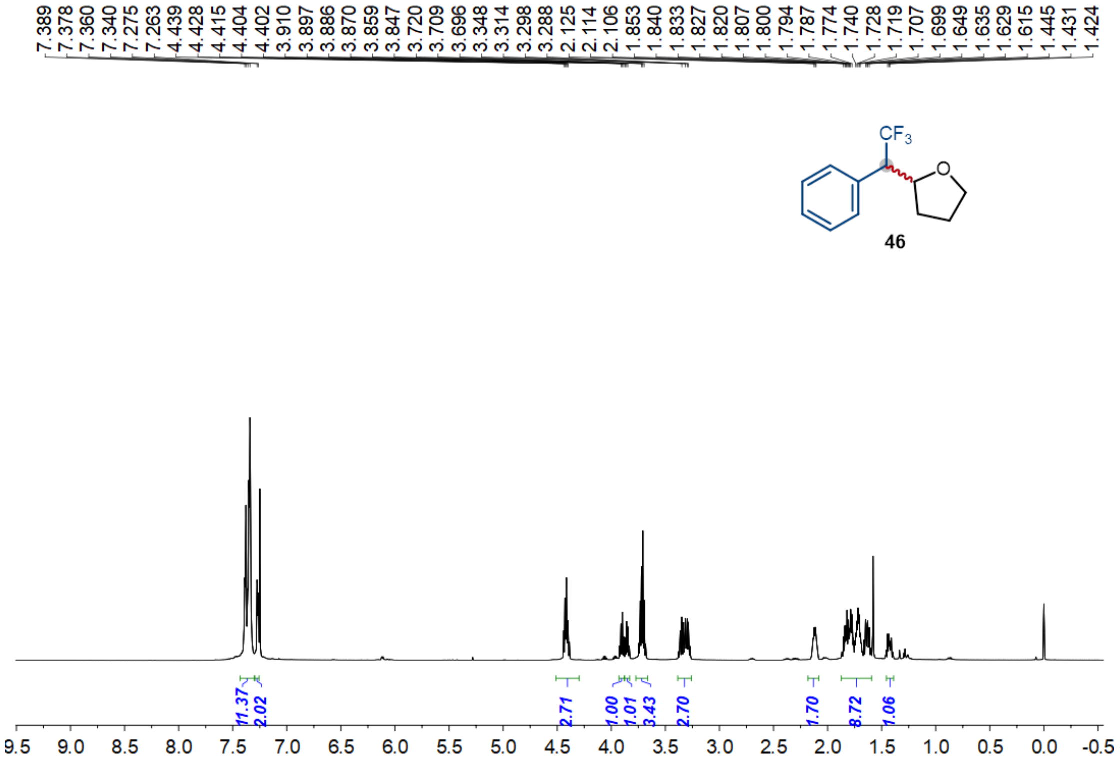

Supplementary Fig. 110 ^{1}H NMR (600 MHz, $CDCl_3$) spectrum of compound **46**

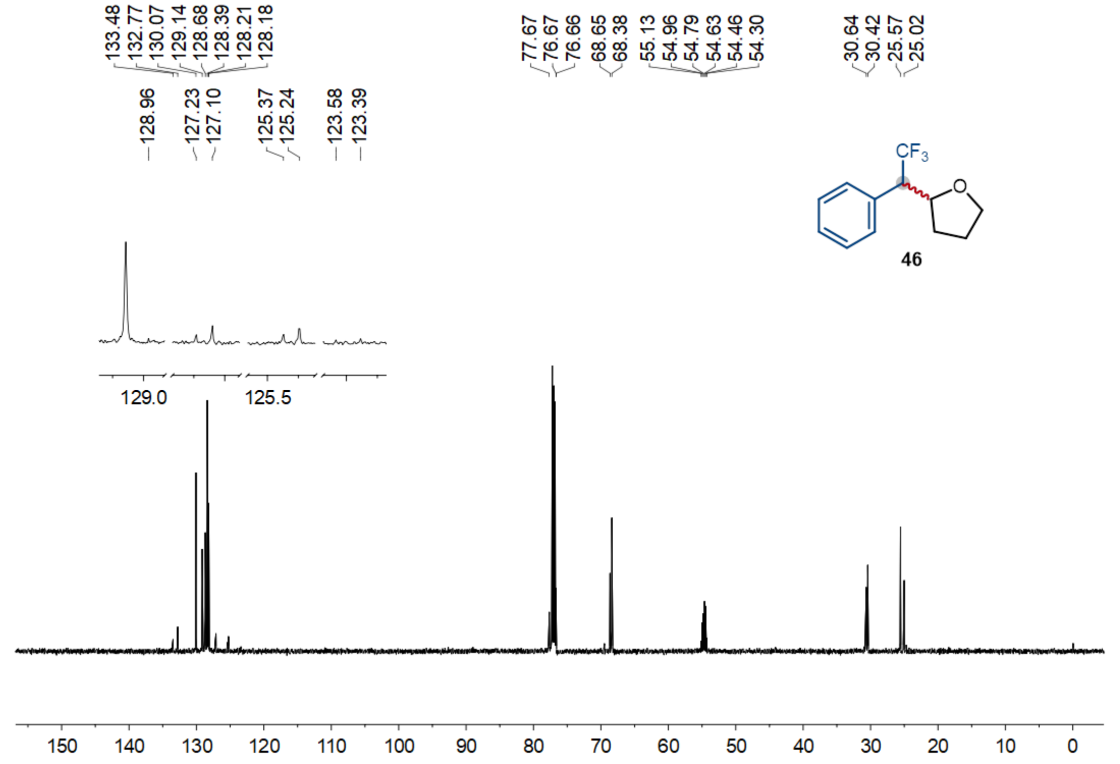

Supplementary Fig. 111 ^{13}C NMR (151 MHz, $CDCl_3$) spectrum of compound **46**

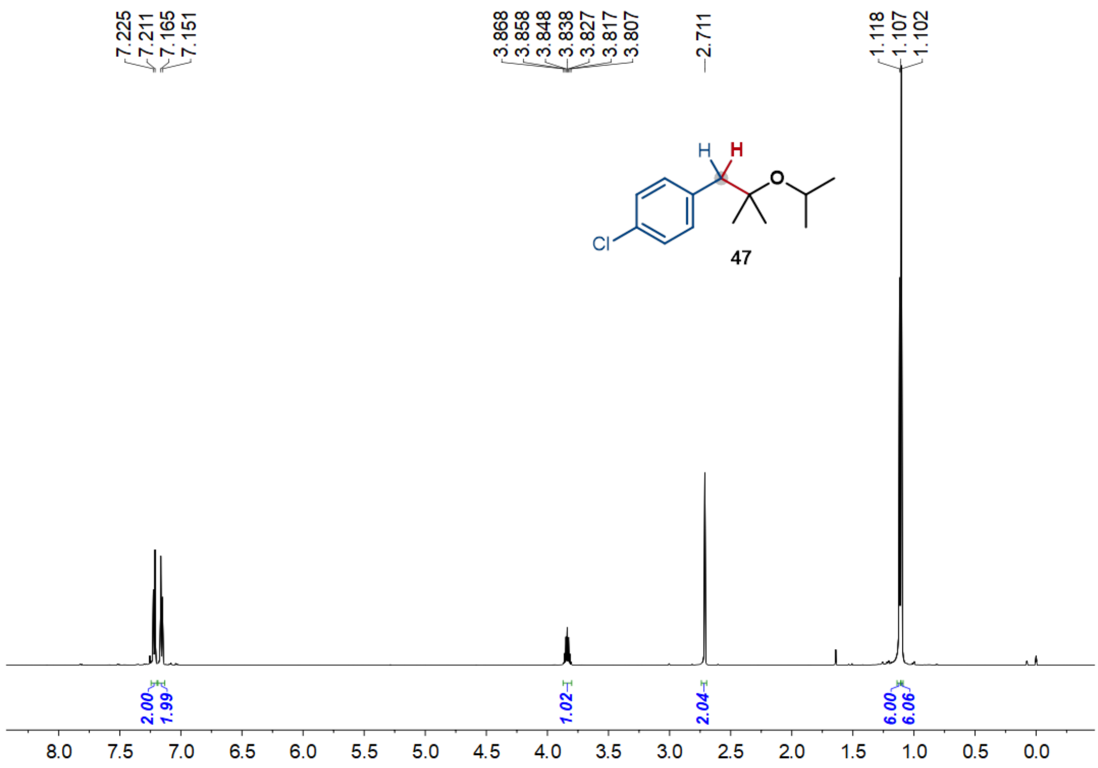

Supplementary Fig. 112 ^{1}H NMR (600 MHz, $CDCl_3$) spectrum of compound **47**

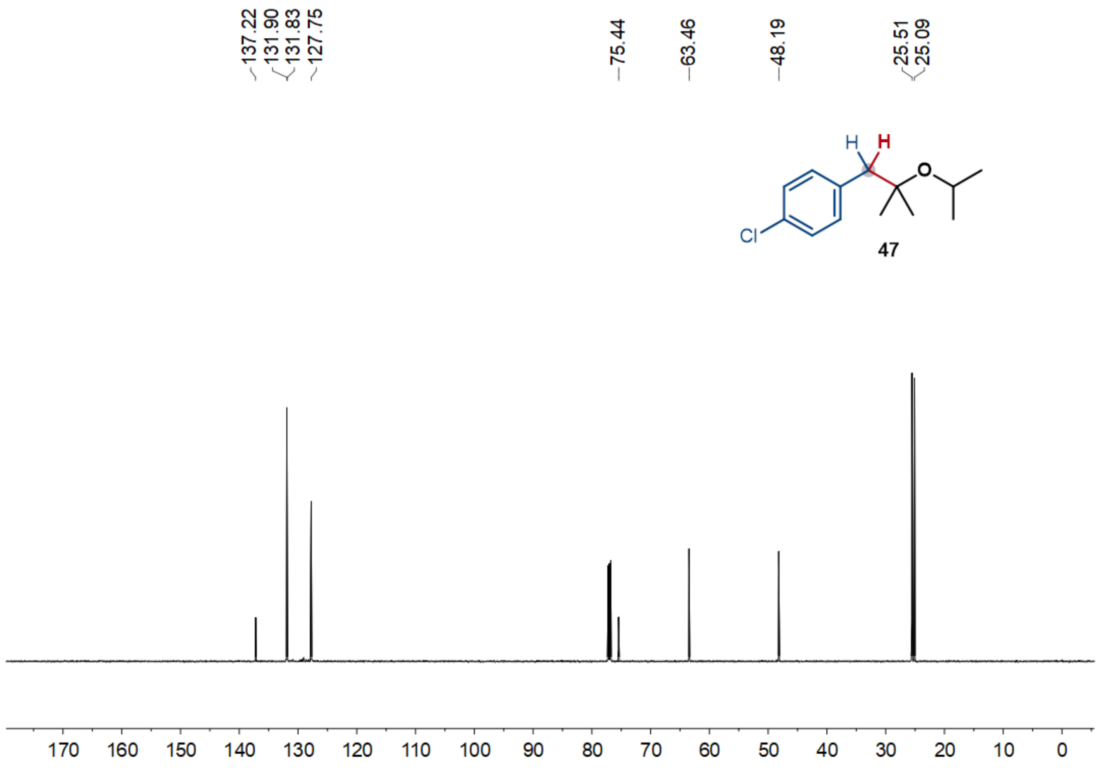

Supplementary Fig. 113 ^{13}C NMR (151 MHz, $CDCl_3$) spectrum of compound **47**

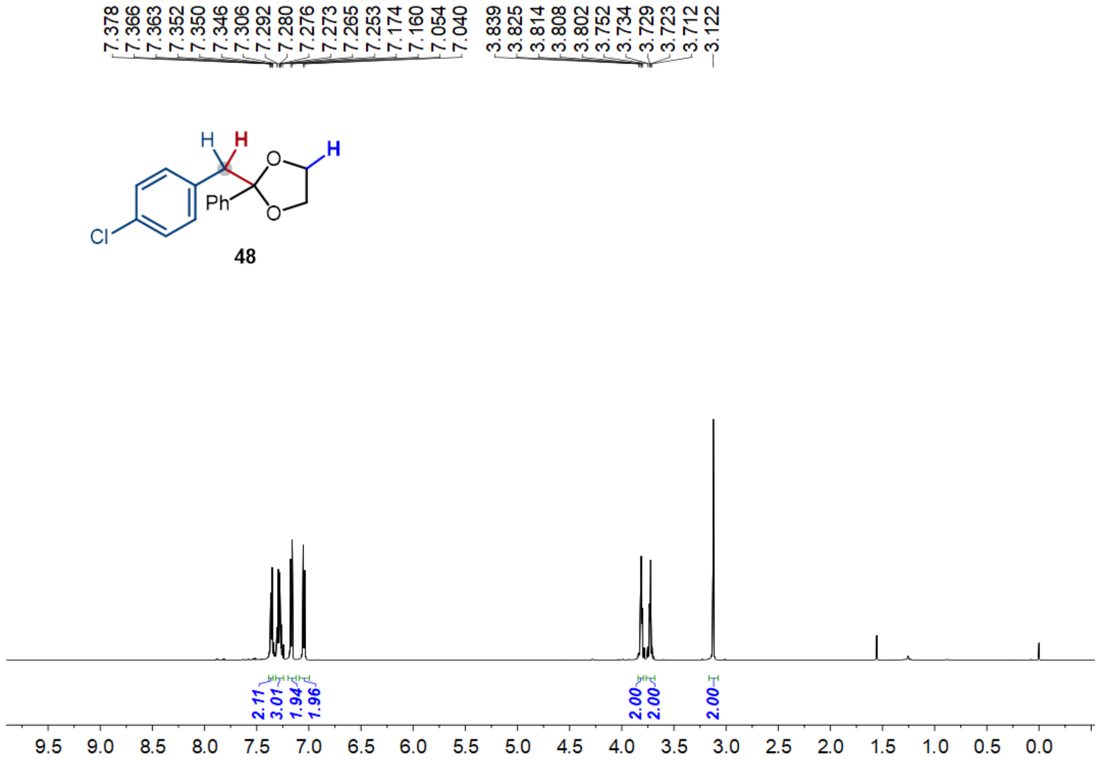

Supplementary Fig. 114 ^{1}H NMR (600 MHz, $CDCl_3$) spectrum of compound **48**

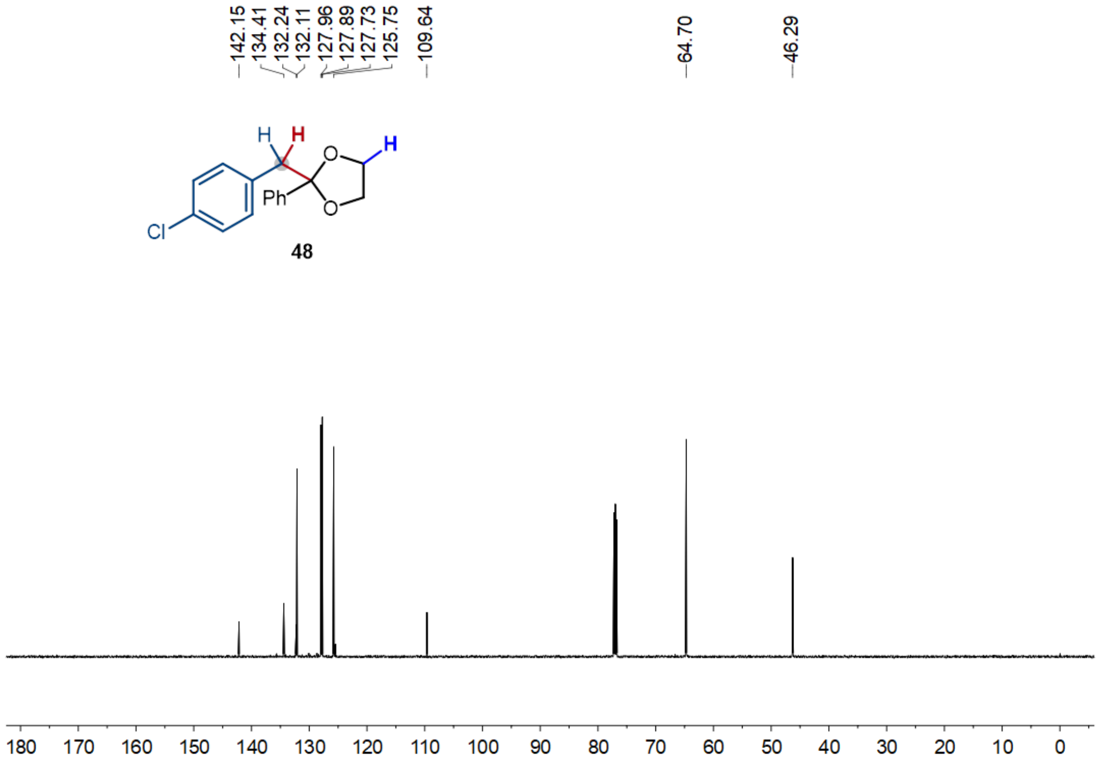

Supplementary Fig. 115 ^{13}C NMR (151 MHz, $CDCl_3$) spectrum of compound **48**

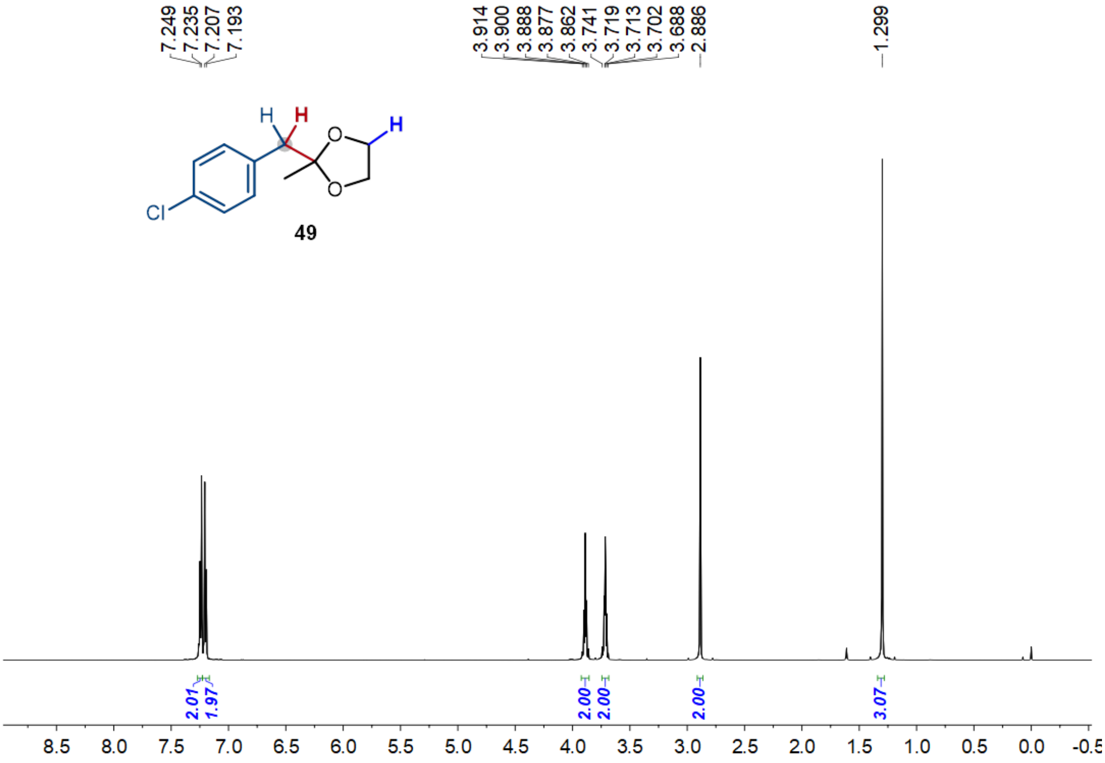

Supplementary Fig. 116 ^{1}**H NMR** (600 MHz, $CDCl_3$) spectrum of compound **49**

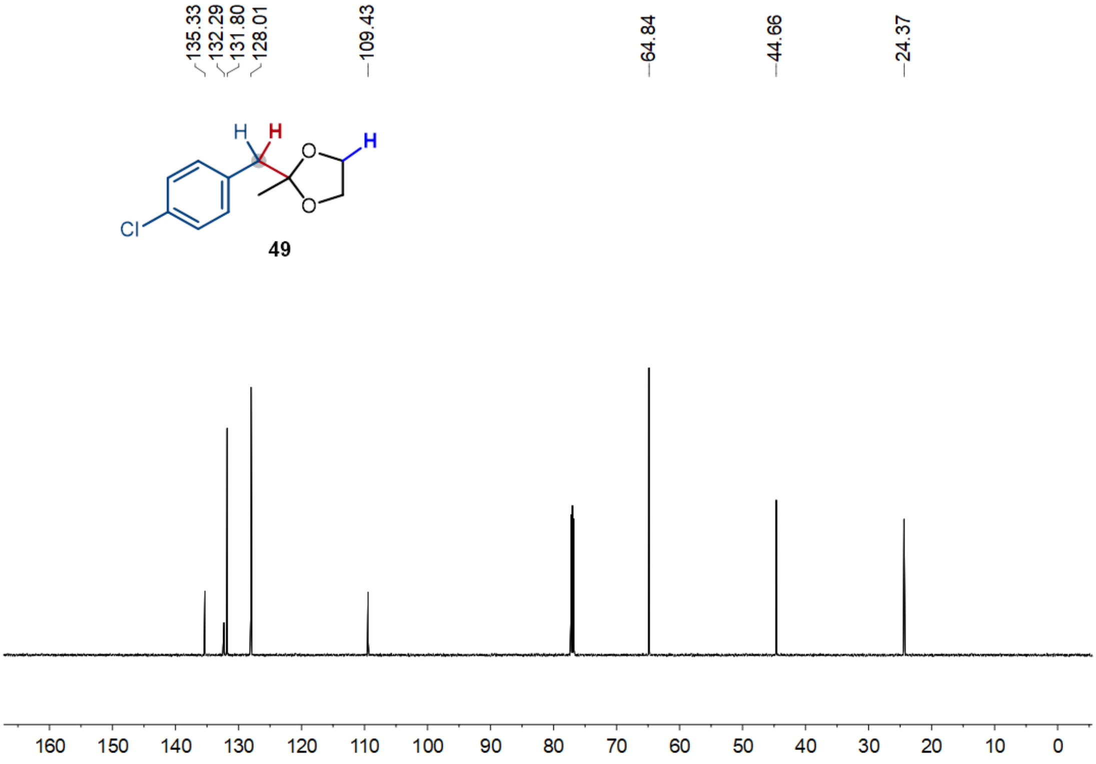

Supplementary Fig. 117 ^{13}**C NMR** (151 MHz, $CDCl_3$) spectrum of compound **49**

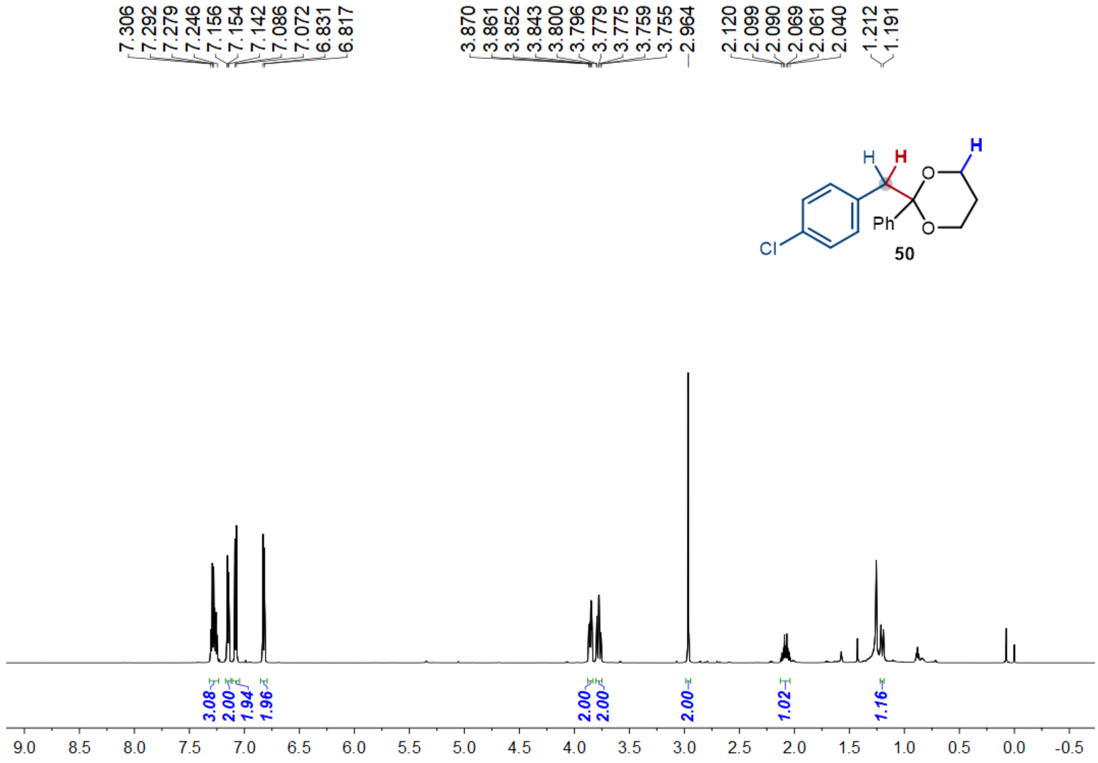

Supplementary Fig. 118 ^{1}**H NMR** (600 MHz, $CDCl_3$) spectrum of compound **50**

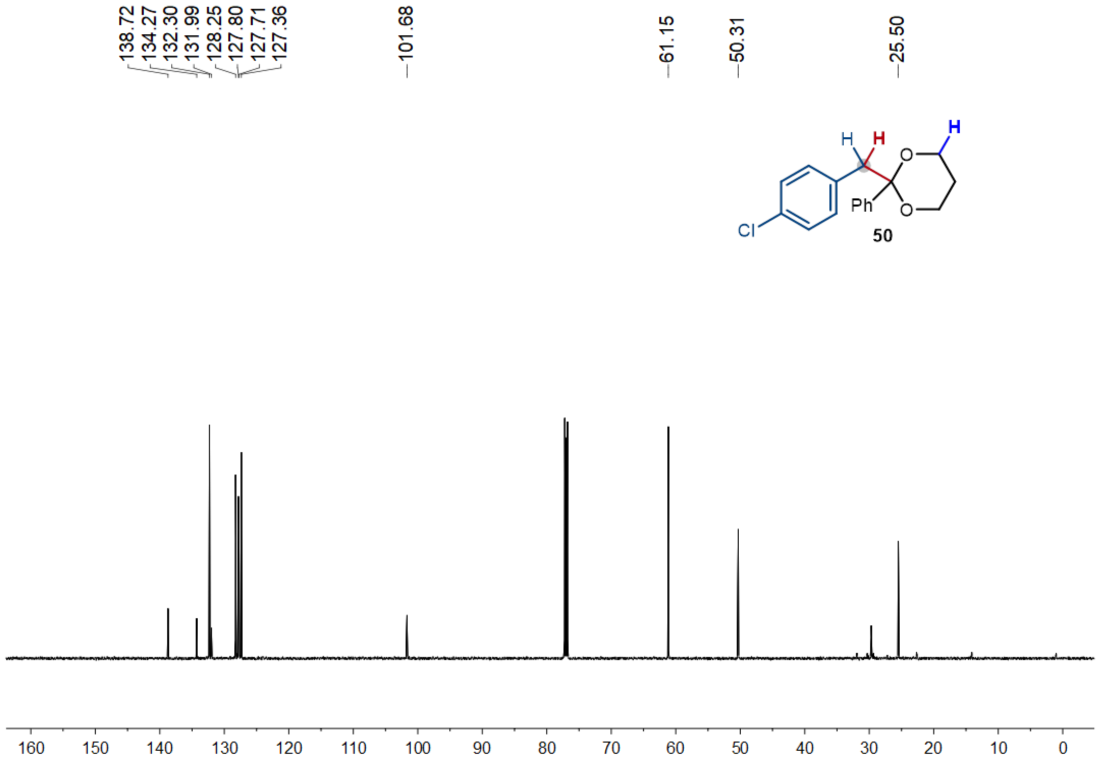

Supplementary Fig. 119 ^{13}**C NMR** (151 MHz, $CDCl_3$) spectrum of compound **50**

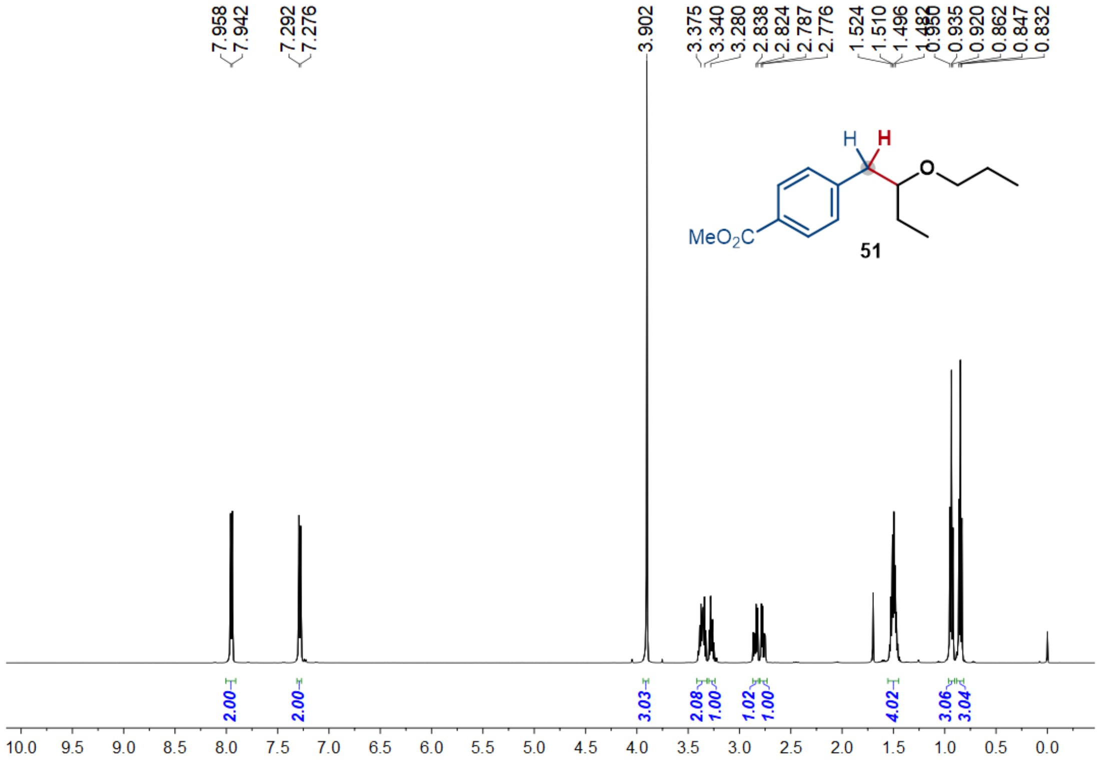

Supplementary Fig. 120 ^{1}H NMR (500 MHz, $CDCl_3$) spectrum of compound **51**

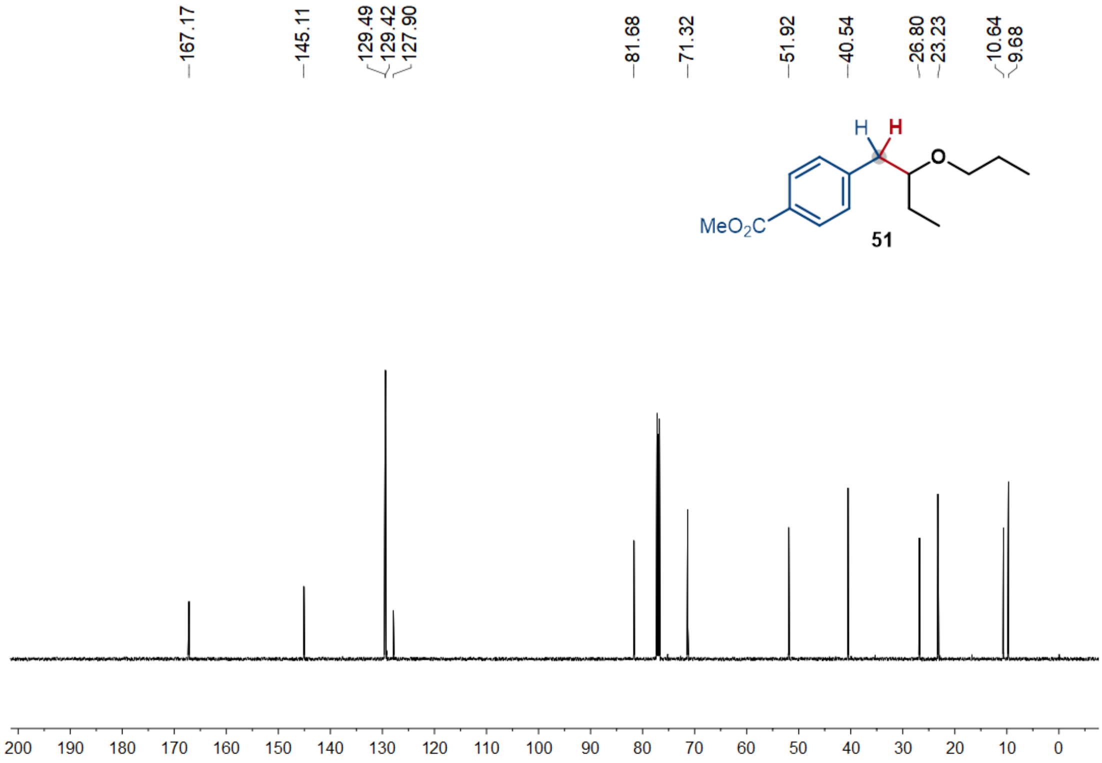

Supplementary Fig. 121 ^{13}C NMR (151 MHz, $CDCl_3$) spectrum of compound **51**

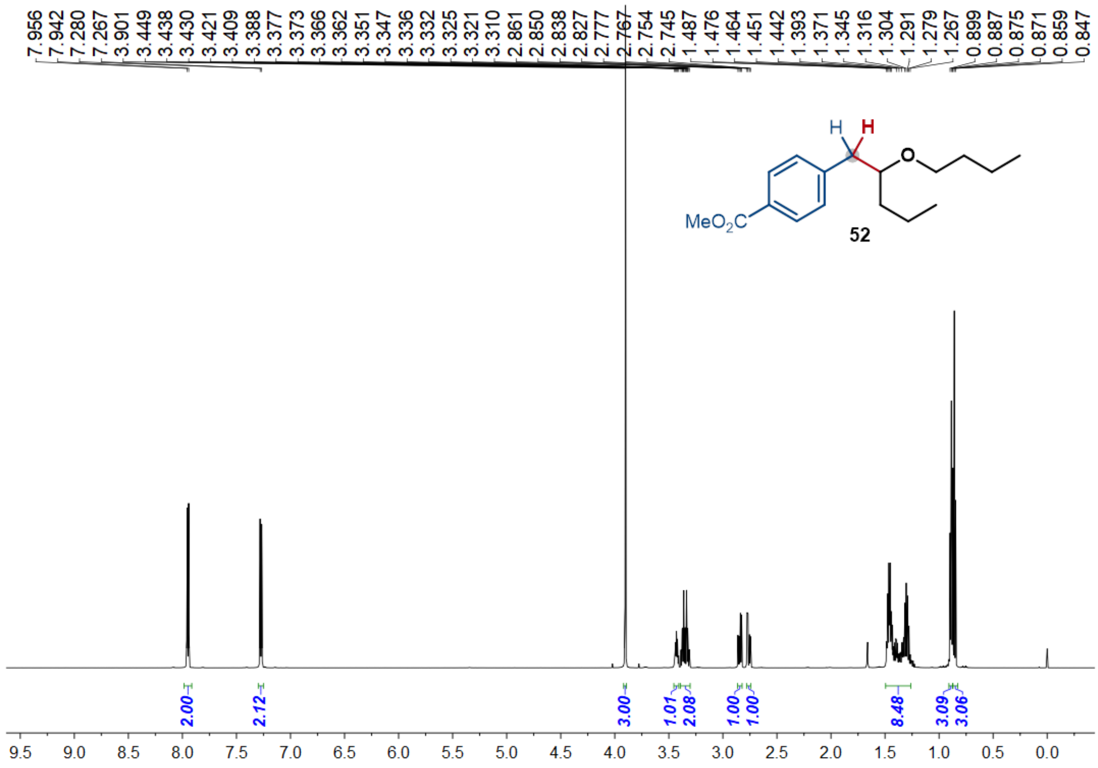

Supplementary Fig. 122 **^{1}H NMR** (600 MHz, $CDCl_3$) spectrum of compound **52**

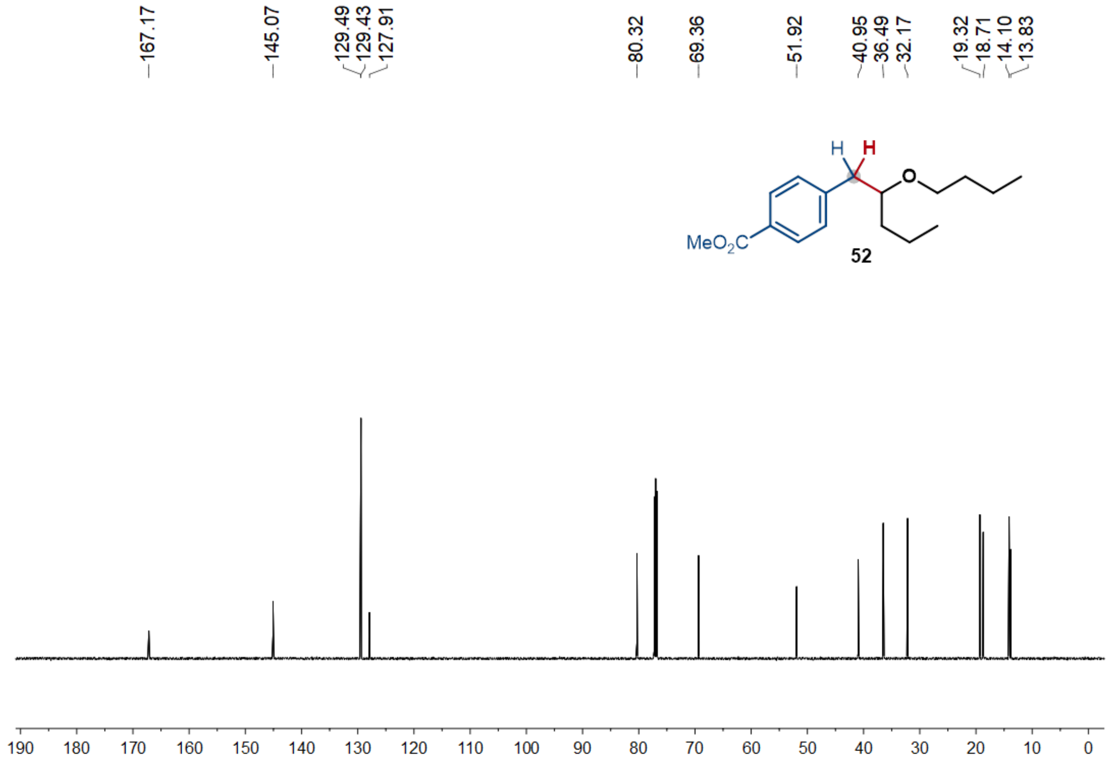

Supplementary Fig. 123 **^{13}C NMR** (151 MHz, $CDCl_3$) spectrum of compound **52**

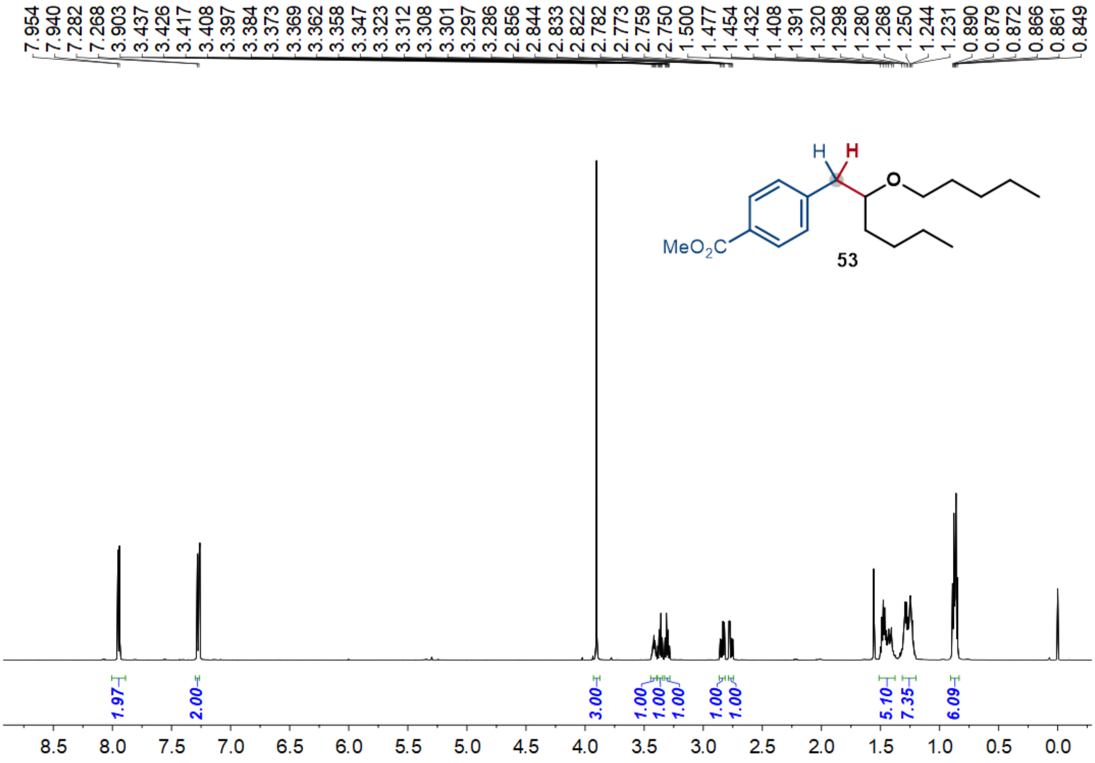

Supplementary Fig. 124 ^{1}H NMR (600 MHz, $CDCl_3$) spectrum of compound **53**

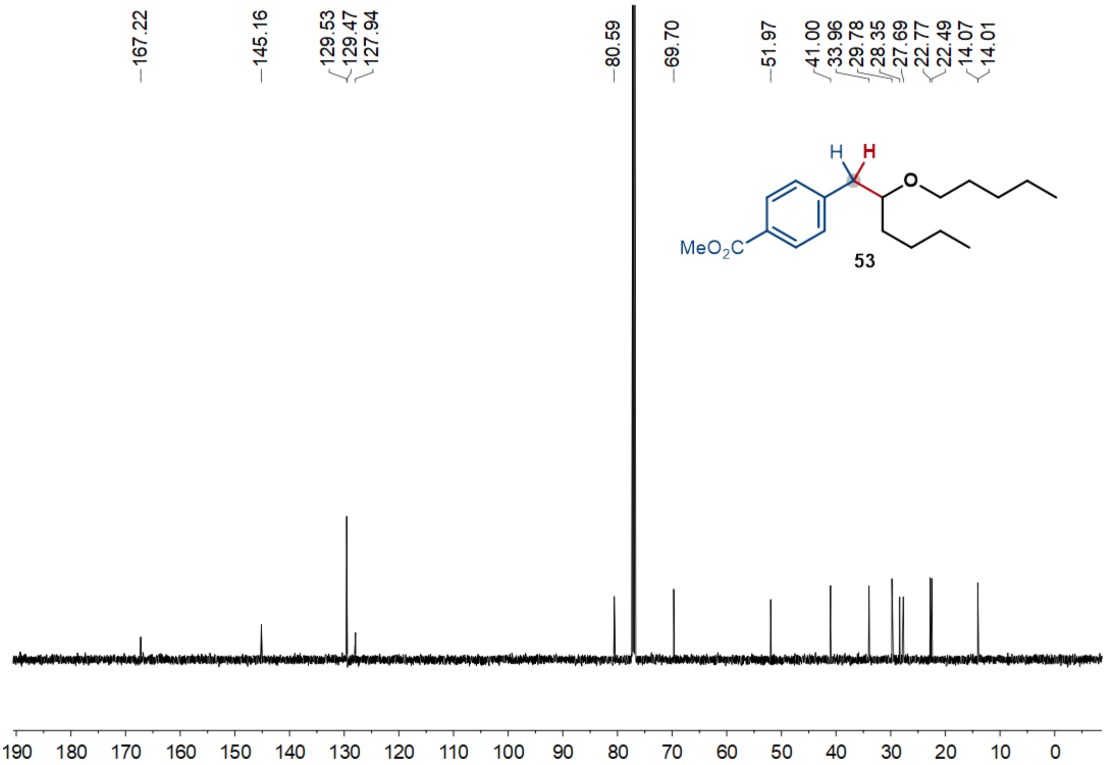

Supplementary Fig. 125 ^{13}C NMR (151 MHz, $CDCl_3$) spectrum of compound **53**

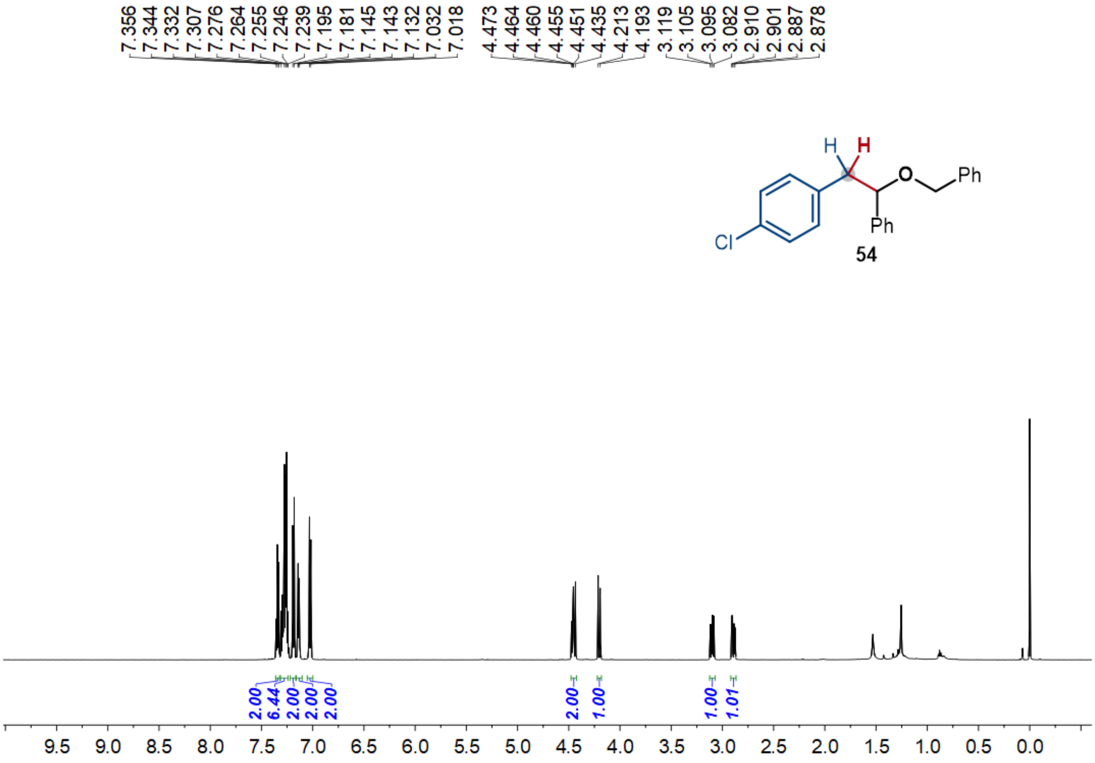

Supplementary Fig. 126 ^{1}H NMR (600 MHz, $CDCl_3$) spectrum of compound **54**

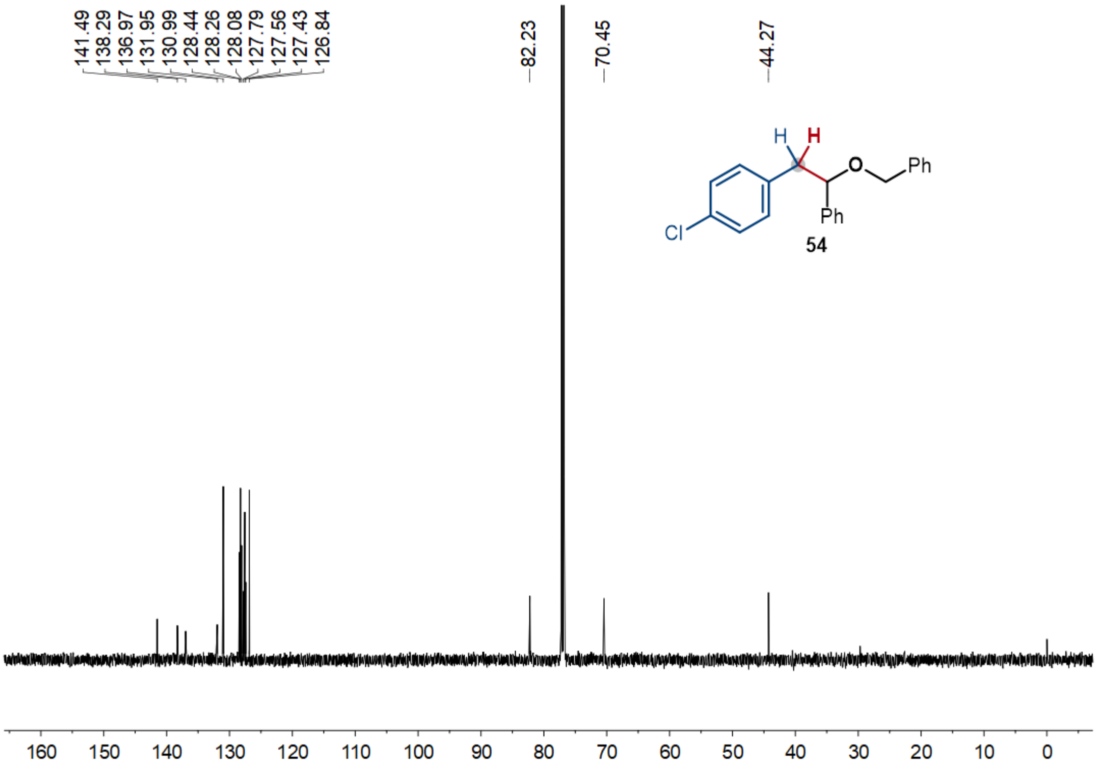

Supplementary Fig. 127 ^{13}C NMR (151 MHz, $CDCl_3$) spectrum of compound **54**

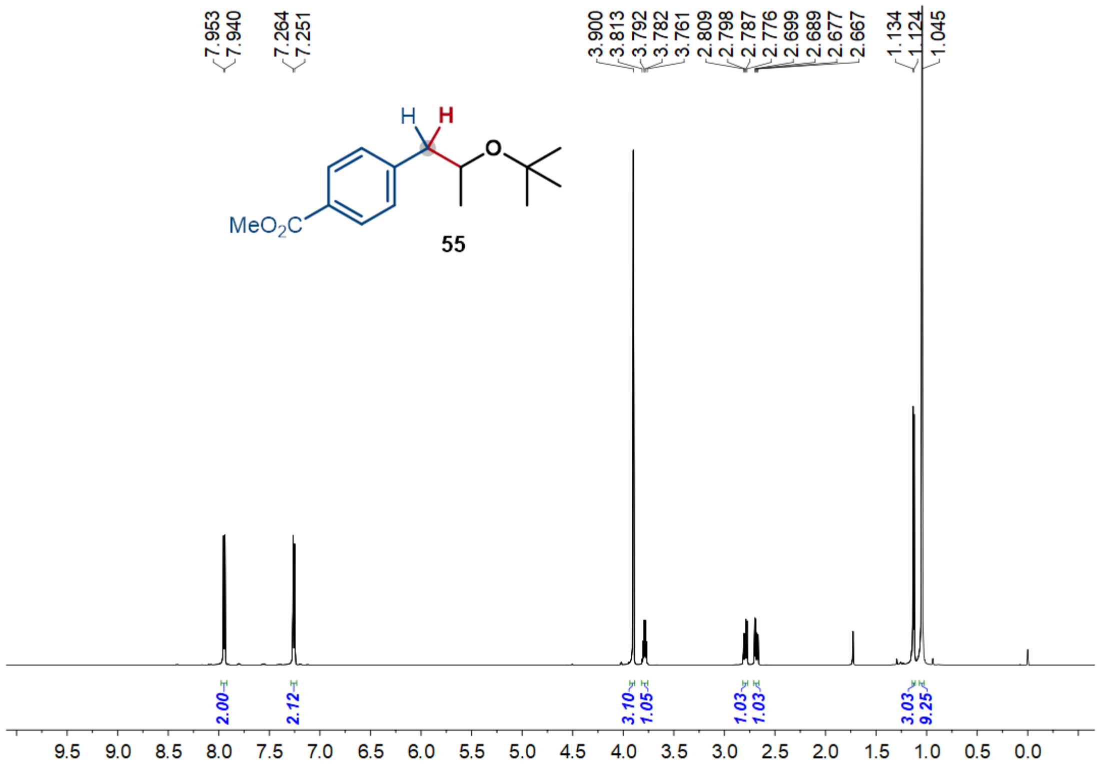

Supplementary Fig. 128 ^{1}**H NMR** (600 MHz, $CDCl_3$) spectrum of compound **55**

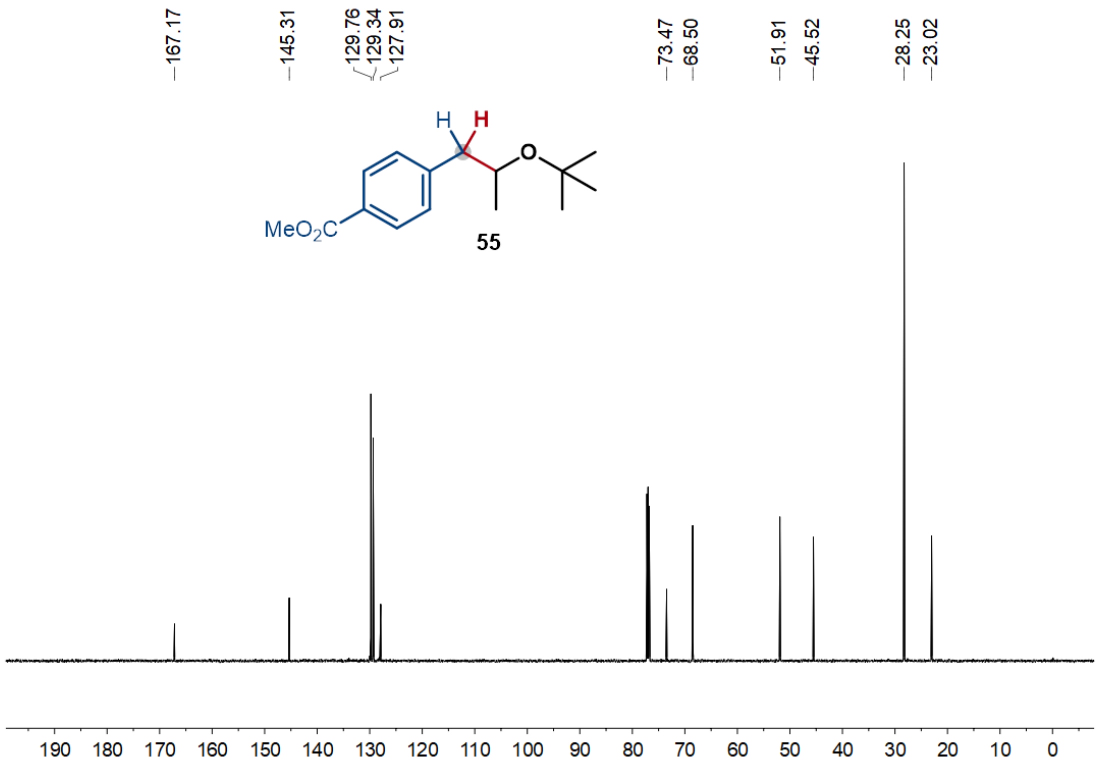

Supplementary Fig. 129 ^{13}**C NMR** (151 MHz, $CDCl_3$) spectrum of compound **55**

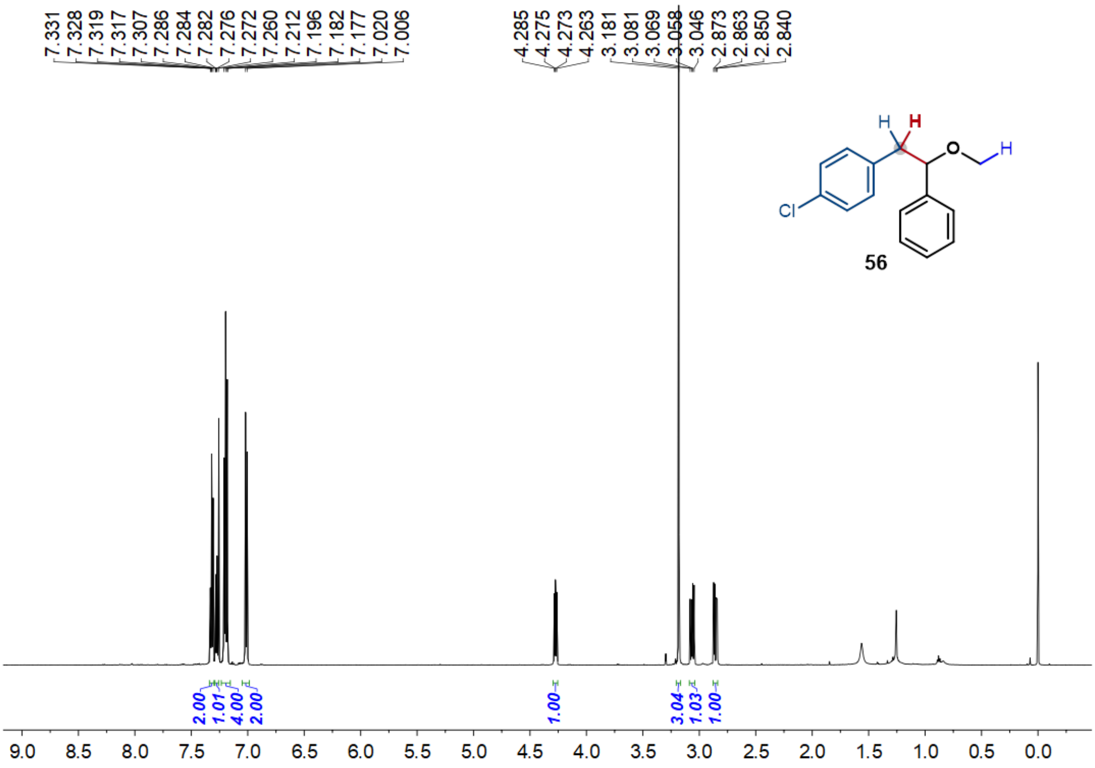

Supplementary Fig. 130 ^{1}H NMR (600 MHz, $CDCl_3$) spectrum of compound **56**

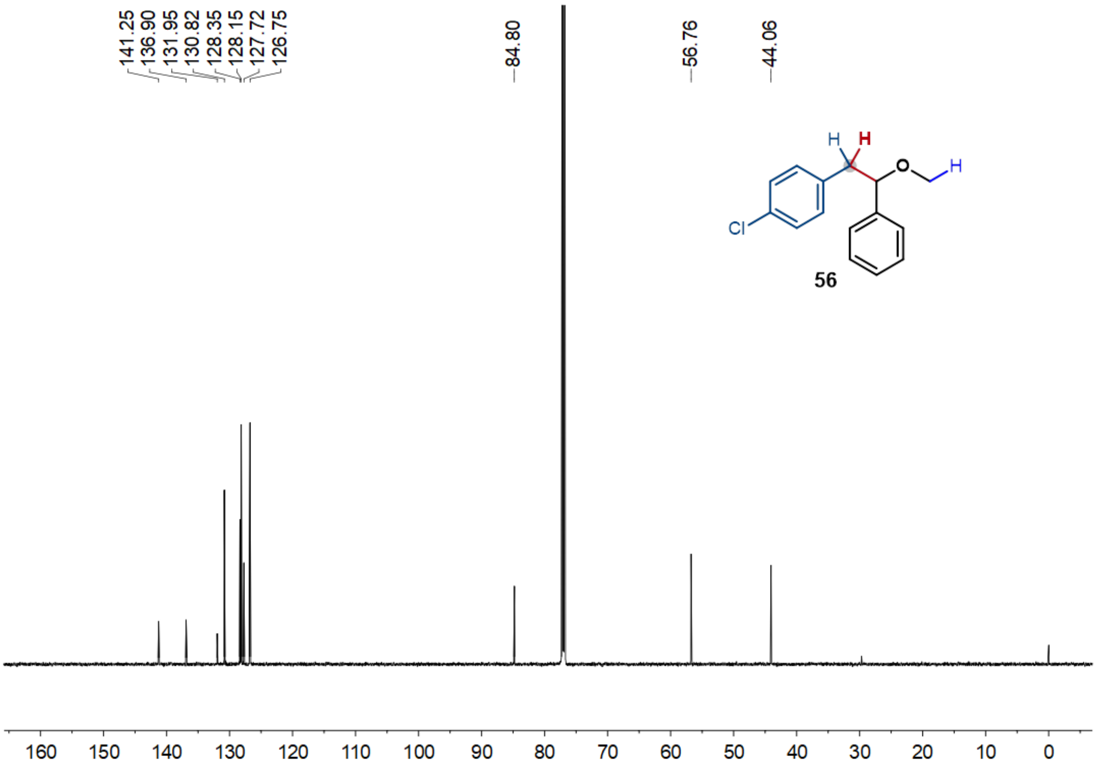

Supplementary Fig. 131 ^{13}C NMR (151 MHz, $CDCl_3$) spectrum of compound **56**

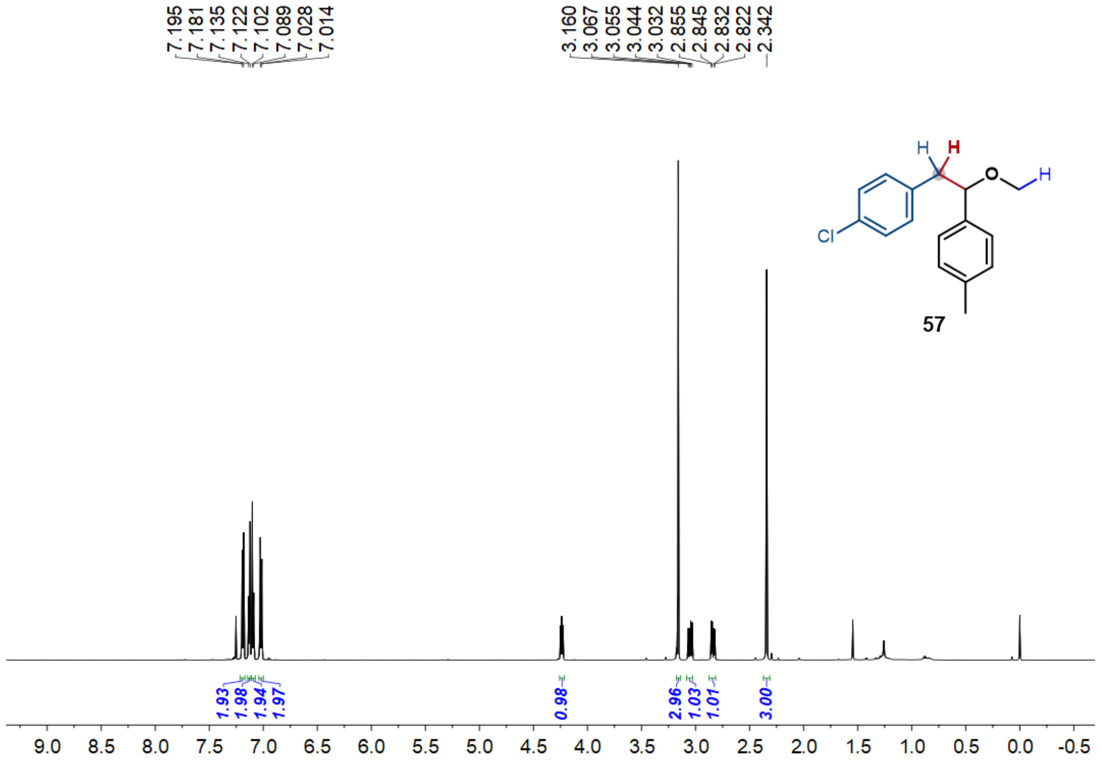

Supplementary Fig. 132 ^{1}H NMR (600 MHz, $CDCl_3$) spectrum of compound **57**

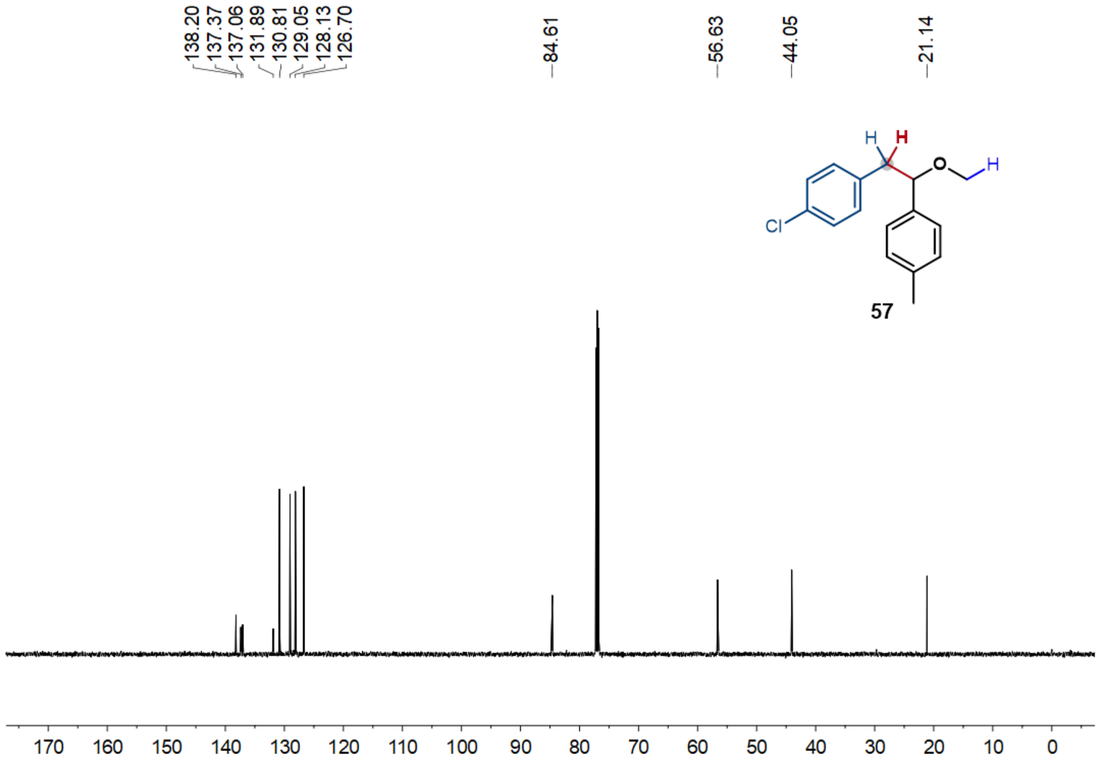

Supplementary Fig. 133 ^{13}C NMR (151 MHz, $CDCl_3$) spectrum of compound **57**

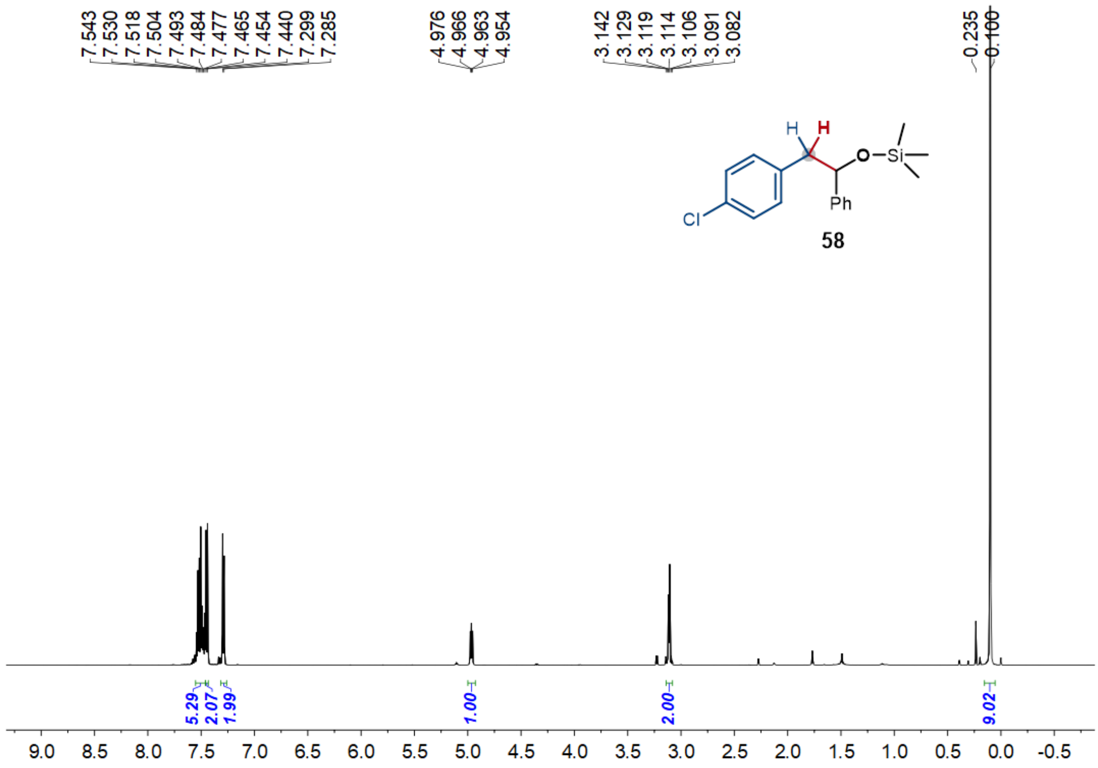

Supplementary Fig. 134 ^{1}H NMR (600 MHz, $CDCl_3$) spectrum of compound **58**

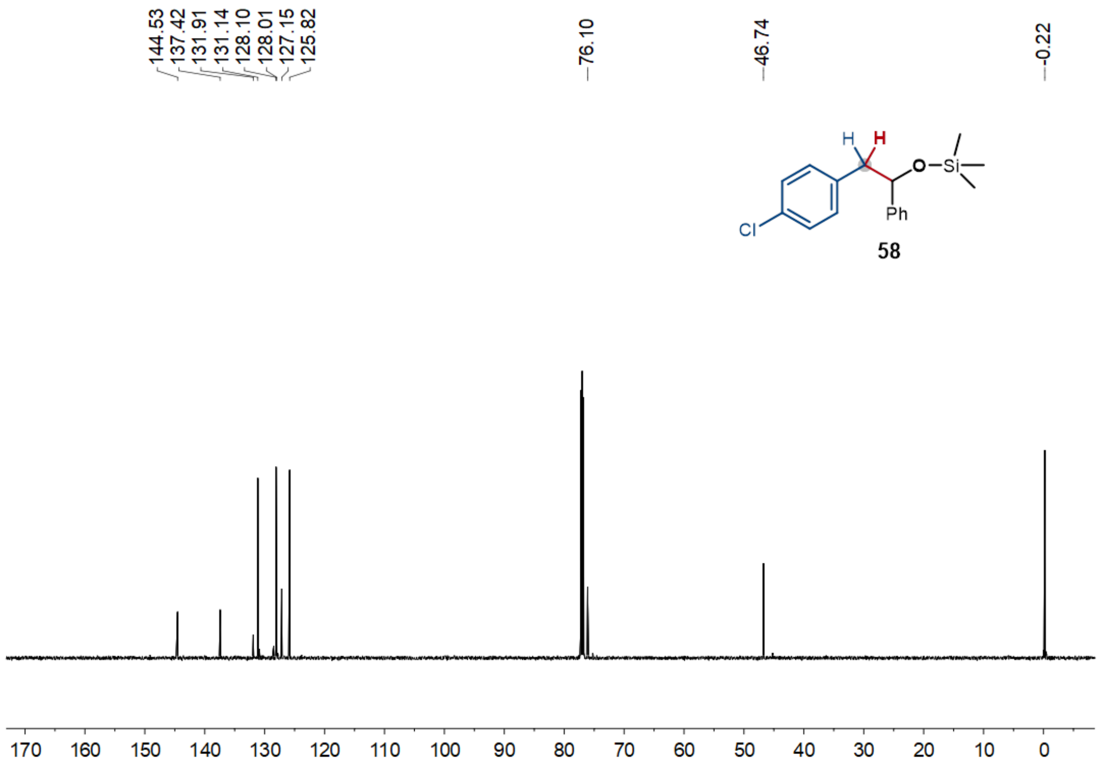

Supplementary Fig. 135 ^{13}C NMR (151 MHz, $CDCl_3$) spectrum of compound **58**

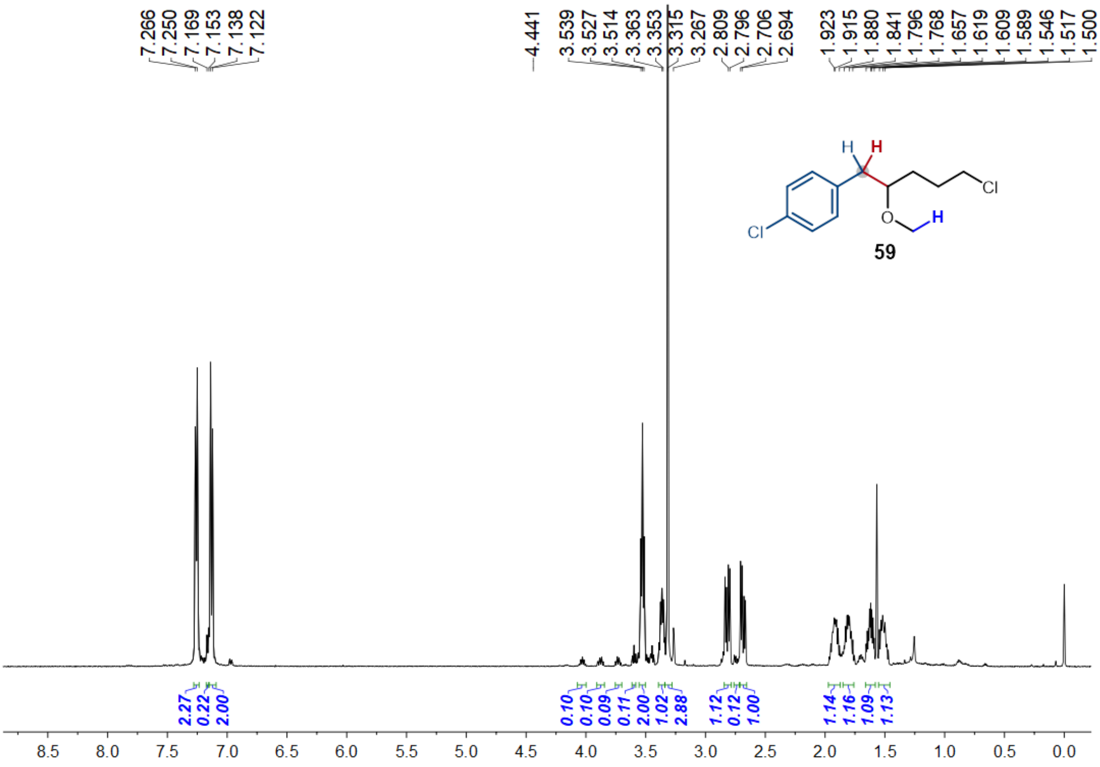

Supplementary Fig. 136 ^{1}H NMR (500 MHz, $CDCl_3$) spectrum of compound **59**

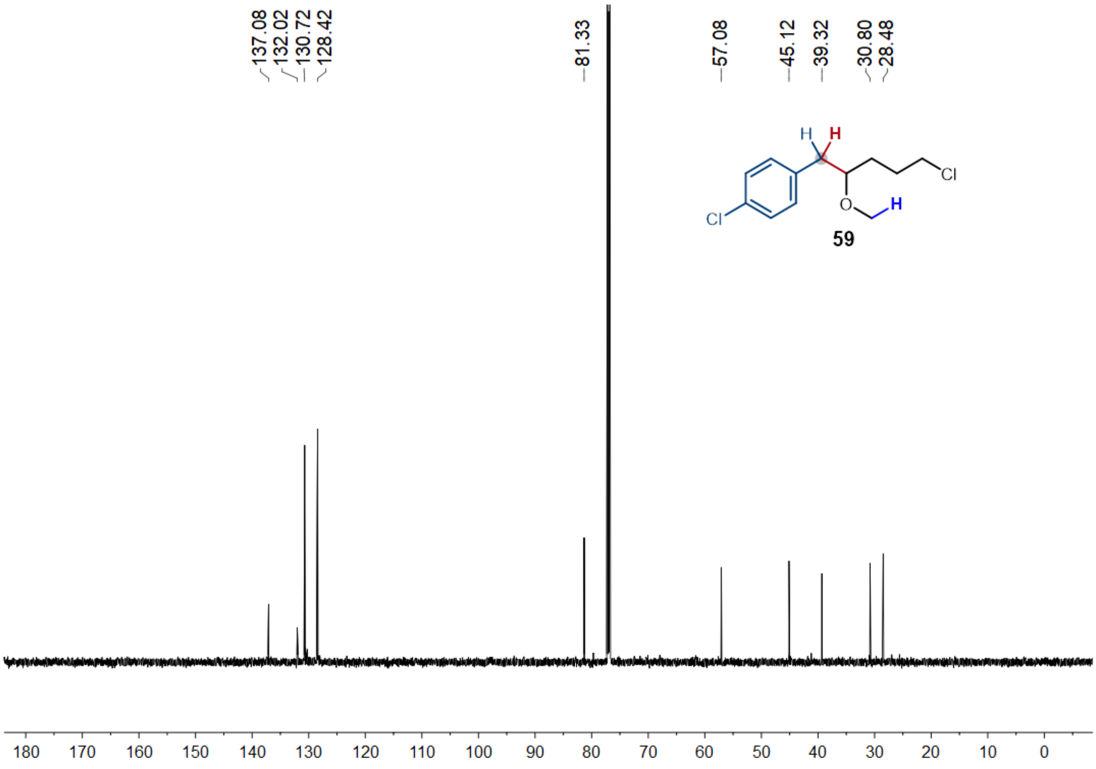

Supplementary Fig. 137 ^{13}C NMR (151 MHz, $CDCl_3$) spectrum of compound **59**

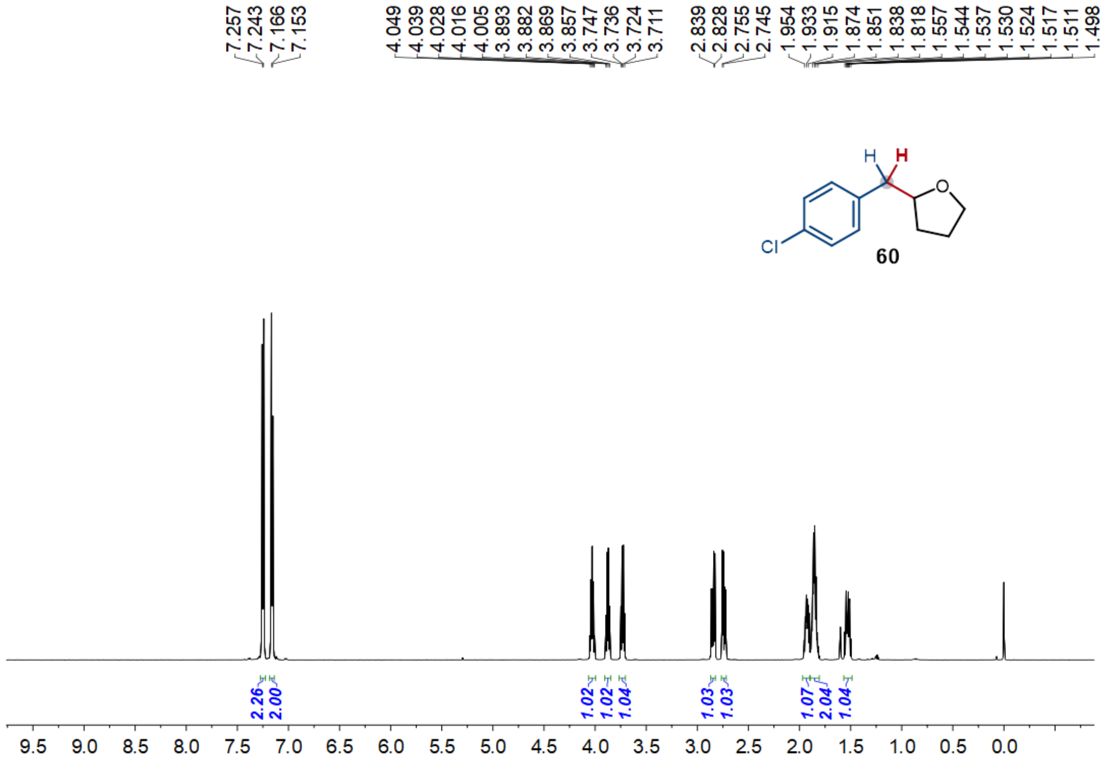

Supplementary Fig. 138 ^{1}H NMR (600 MHz, $CDCl_3$) spectrum of compound **60**

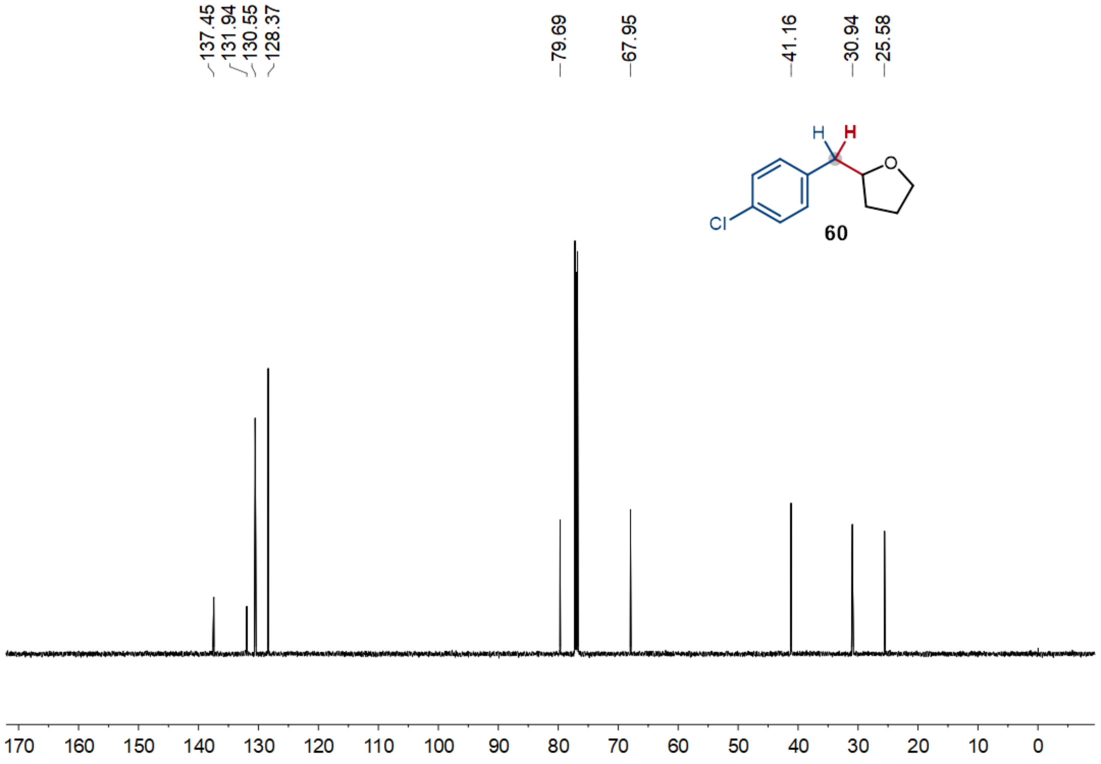

Supplementary Fig. 139 ^{13}C NMR (151 MHz, $CDCl_3$) spectrum of compound **60**

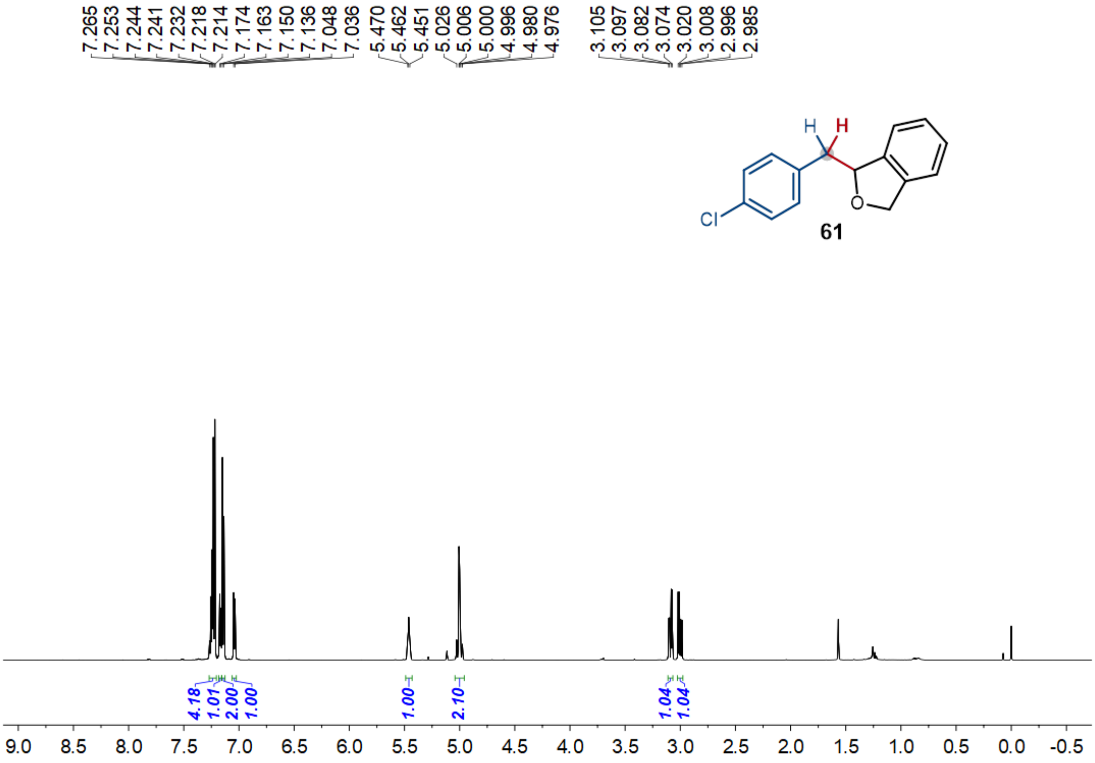

Supplementary Fig. 140 ^{1}**H NMR** (600 MHz, $CDCl_3$) spectrum of compound **61**

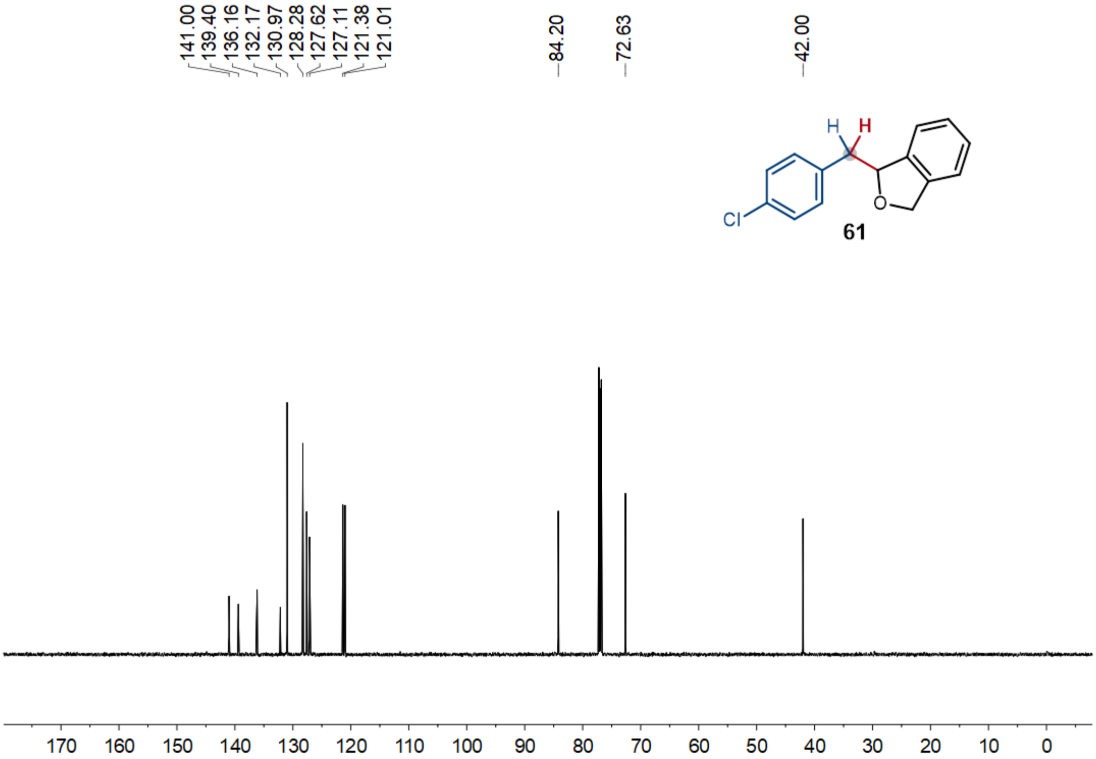

Supplementary Fig. 141 ^{13}**C NMR** (151 MHz, $CDCl_3$) spectrum of compound **61**

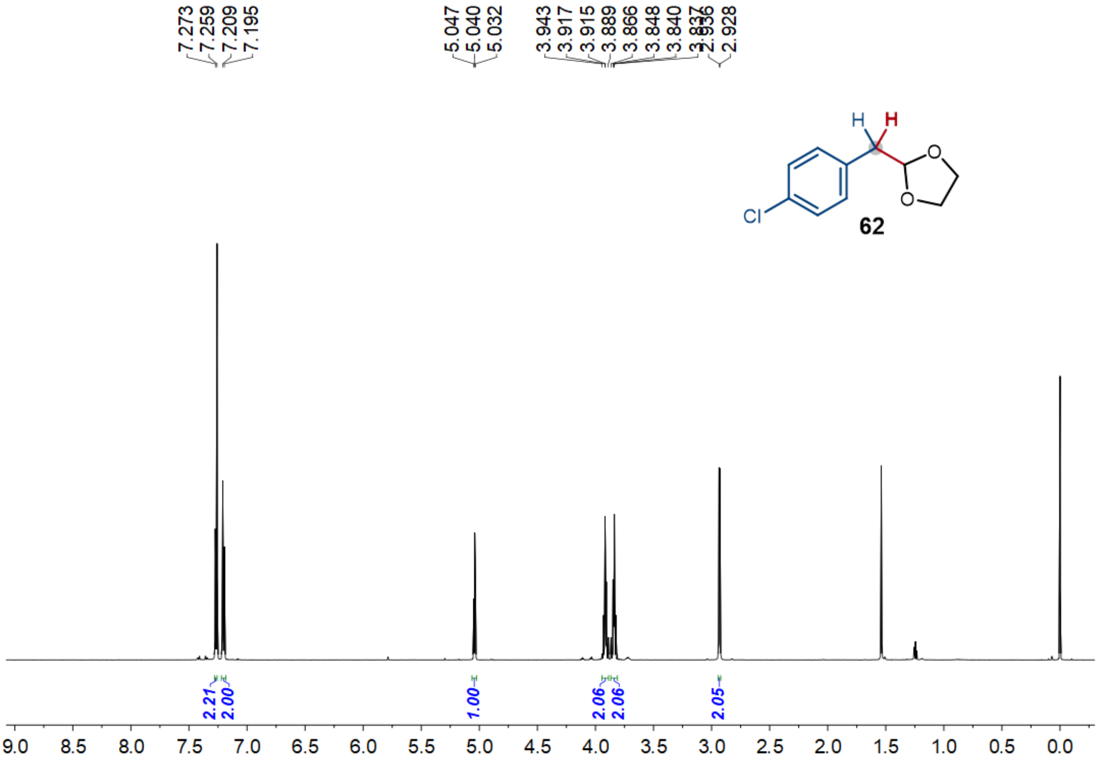

Supplementary Fig. 142 ^{1}H NMR (600 MHz, $CDCl_3$) spectrum of compound **62**

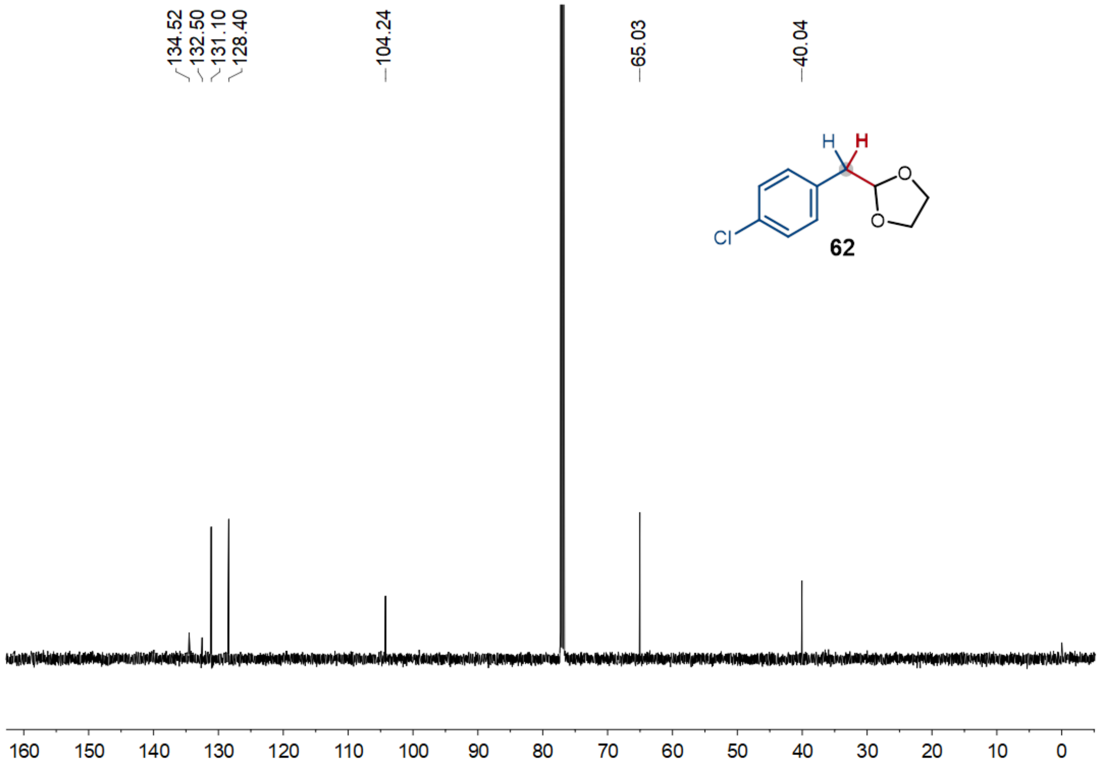

Supplementary Fig. 143 ^{13}C NMR (151 MHz, $CDCl_3$) spectrum of compound **62**

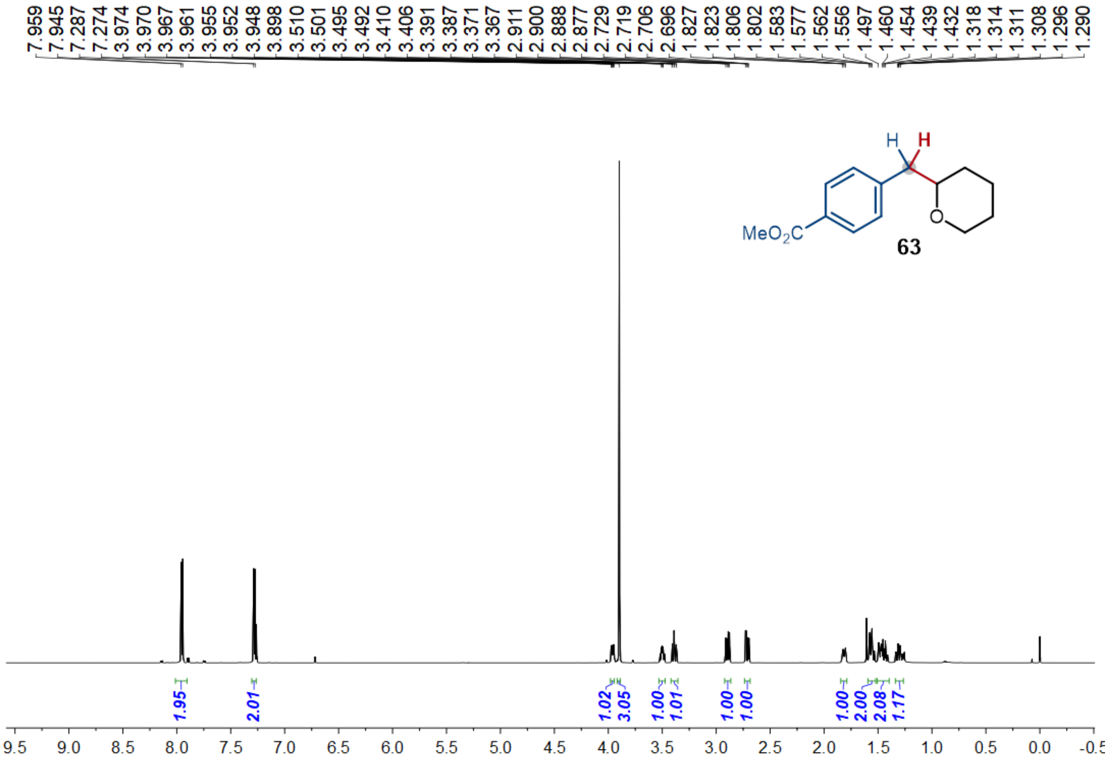

Supplementary Fig. 144 ^{1}H NMR (600 MHz, $CDCl_3$) spectrum of compound **63**

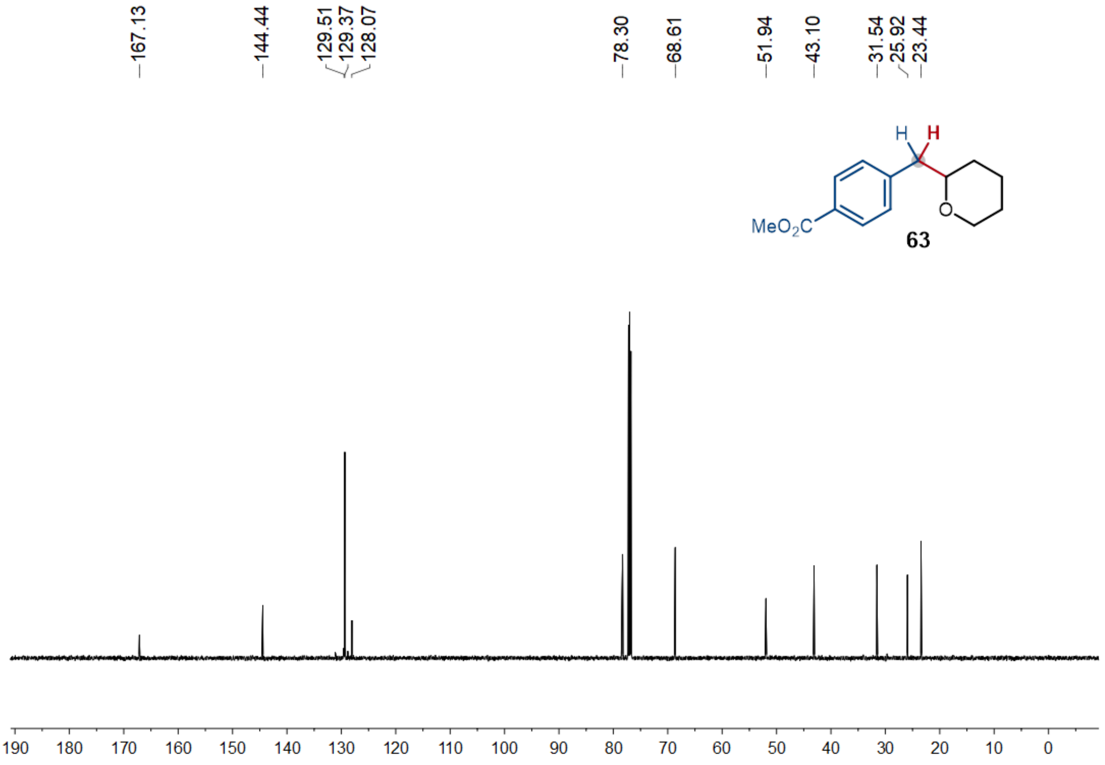

Supplementary Fig. 145 ^{13}C NMR (151 MHz, $CDCl_3$) spectrum of compound **63**

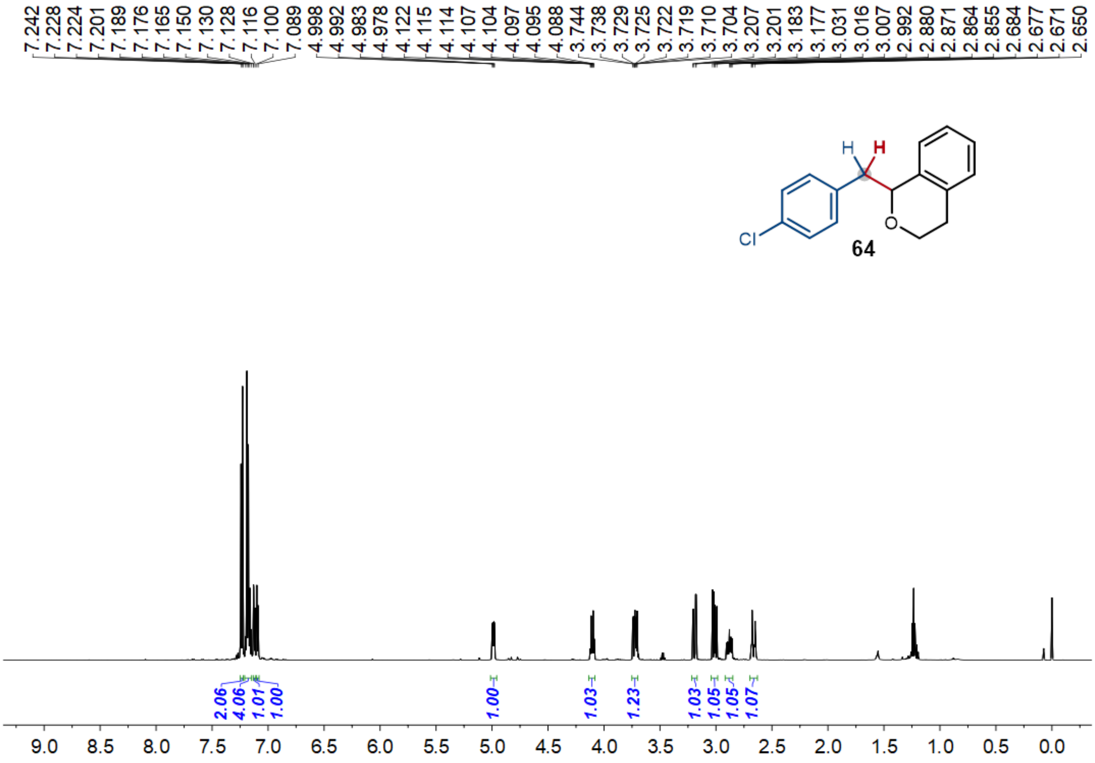

Supplementary Fig. 146 ^{1}H NMR (600 MHz, $CDCl_3$) spectrum of compound **64**

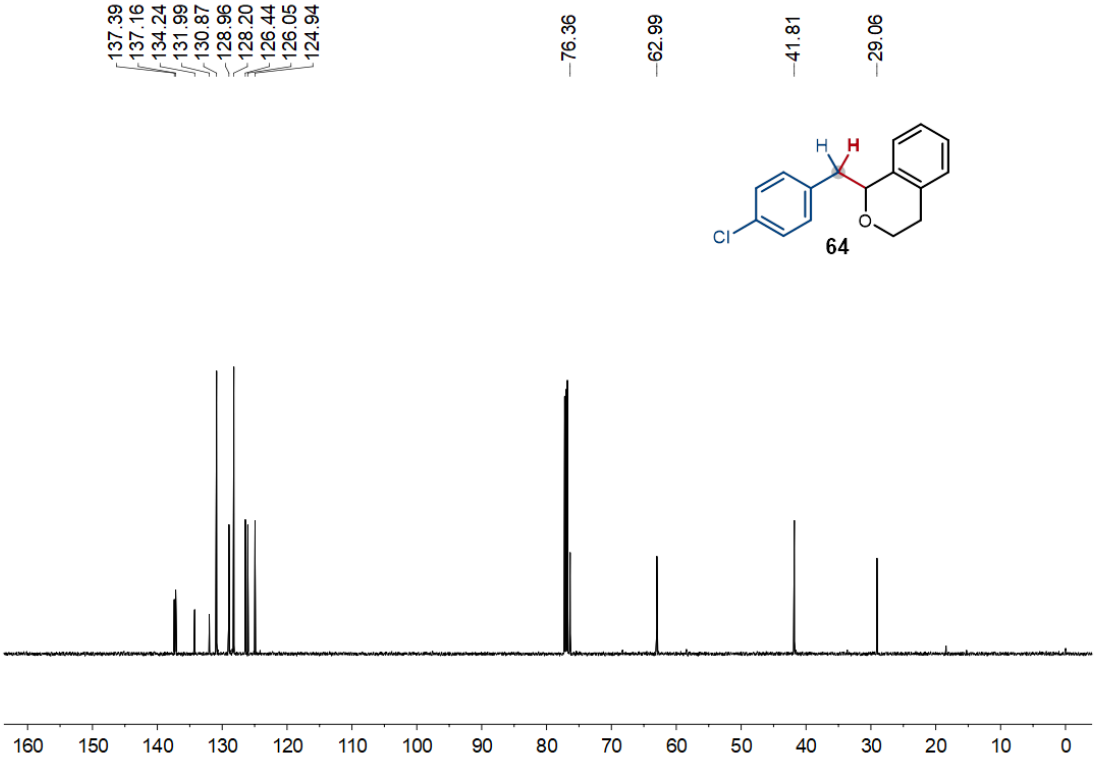

Supplementary Fig. 147 ^{13}C NMR (151 MHz, $CDCl_3$) spectrum of compound **64**

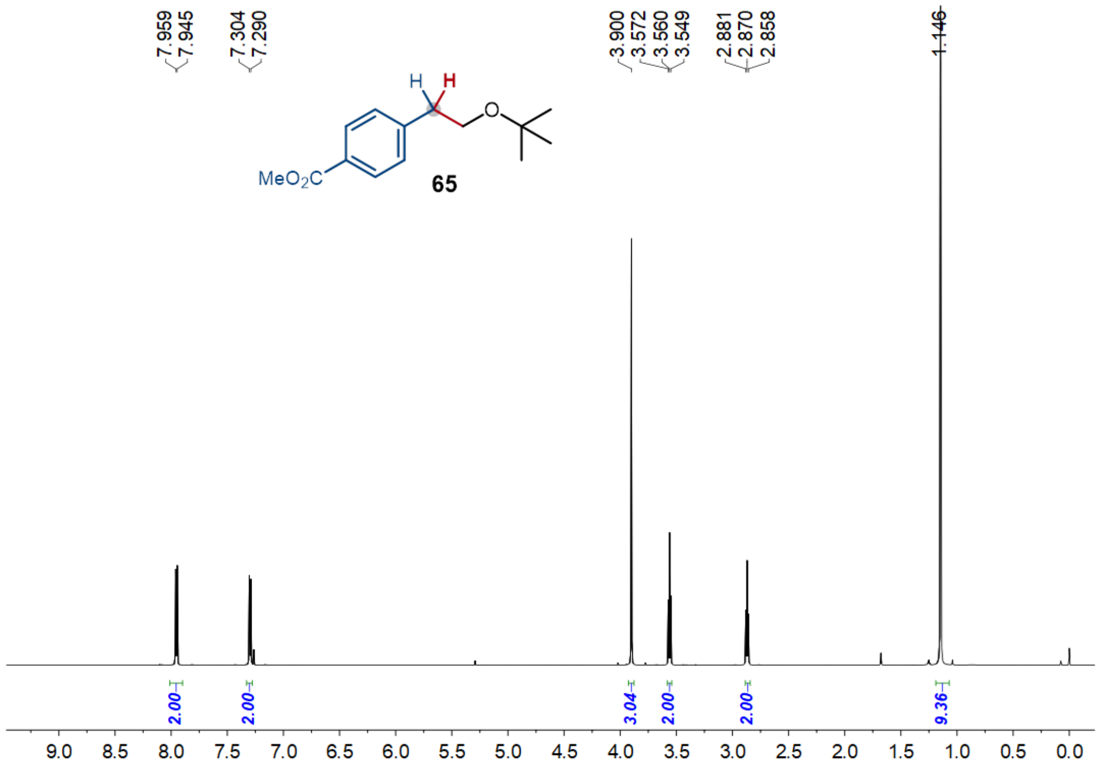

Supplementary Fig. 148 ^{1}H NMR (600 MHz, $CDCl_3$) spectrum of compound **65**

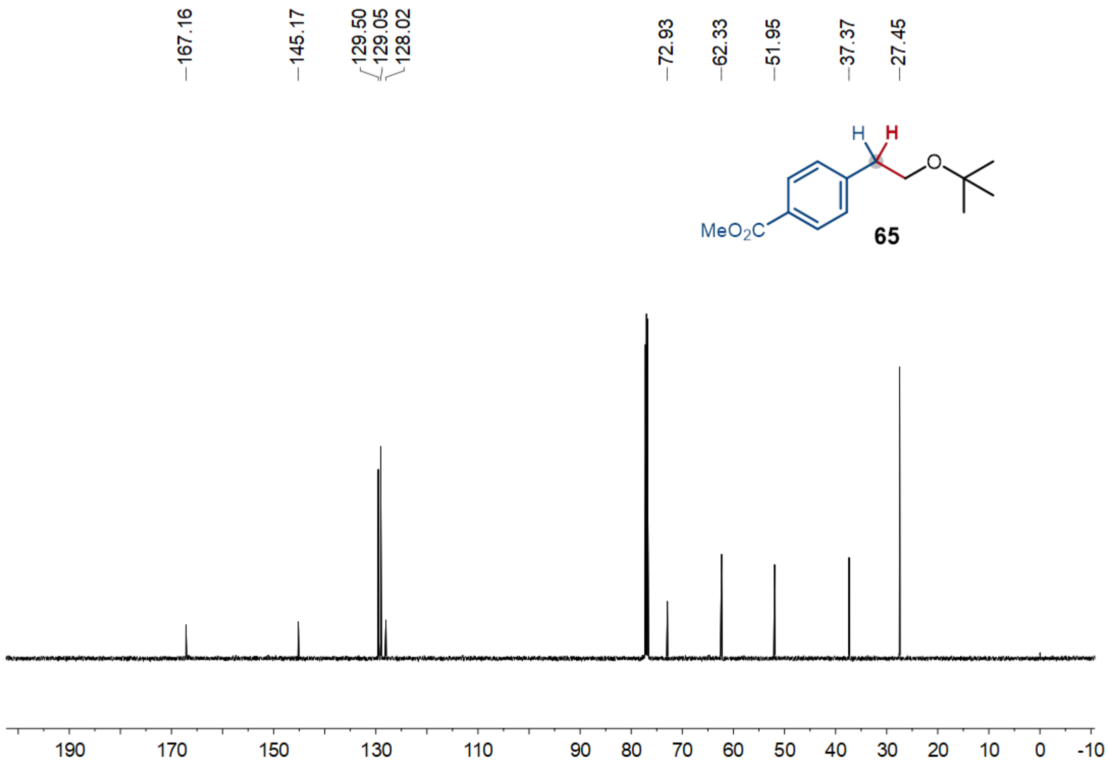

Supplementary Fig. 149 ^{13}C NMR (151 MHz, $CDCl_3$) spectrum of compound **65**

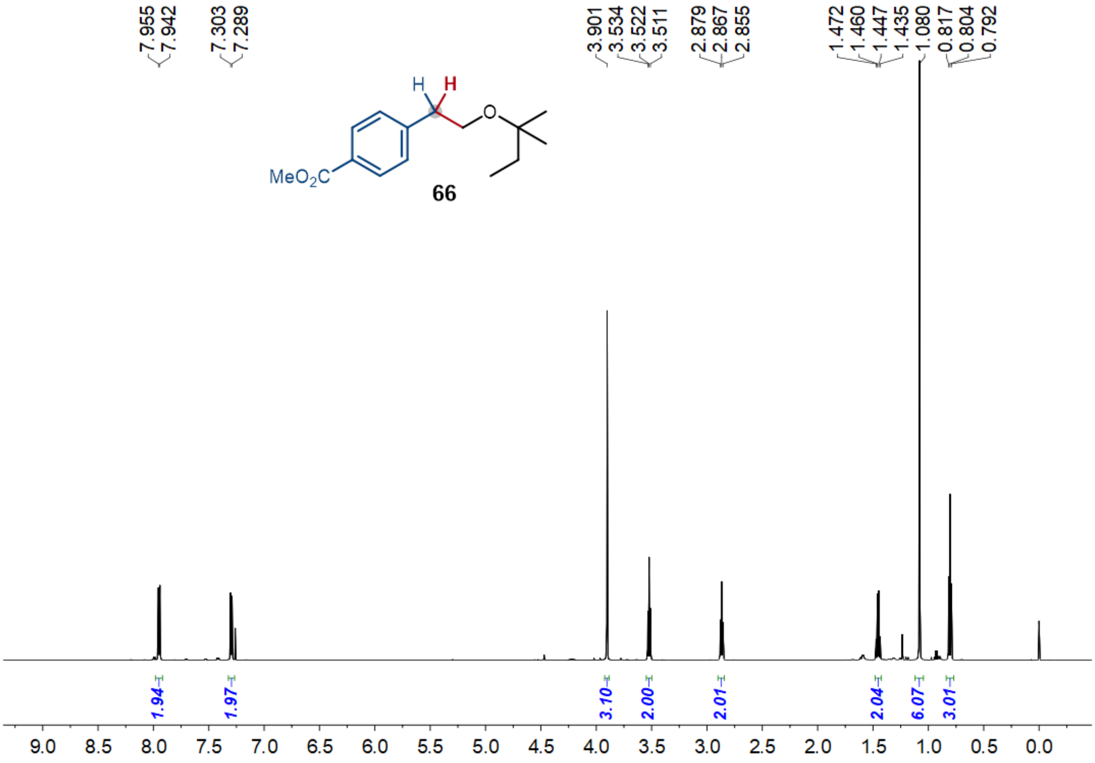

Supplementary Fig. 150 ^{1}H NMR (600 MHz, $CDCl_3$) spectrum of compound **66**

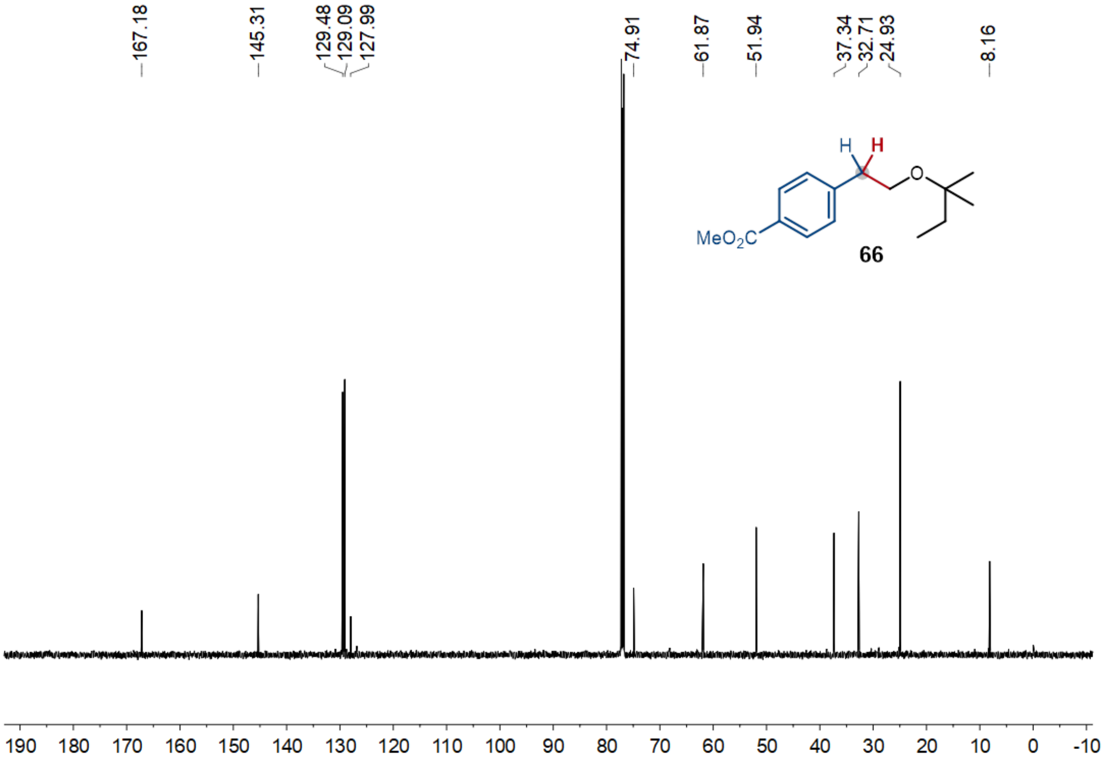

Supplementary Fig. 151 ^{13}C NMR (151 MHz, $CDCl_3$) spectrum of compound **66**

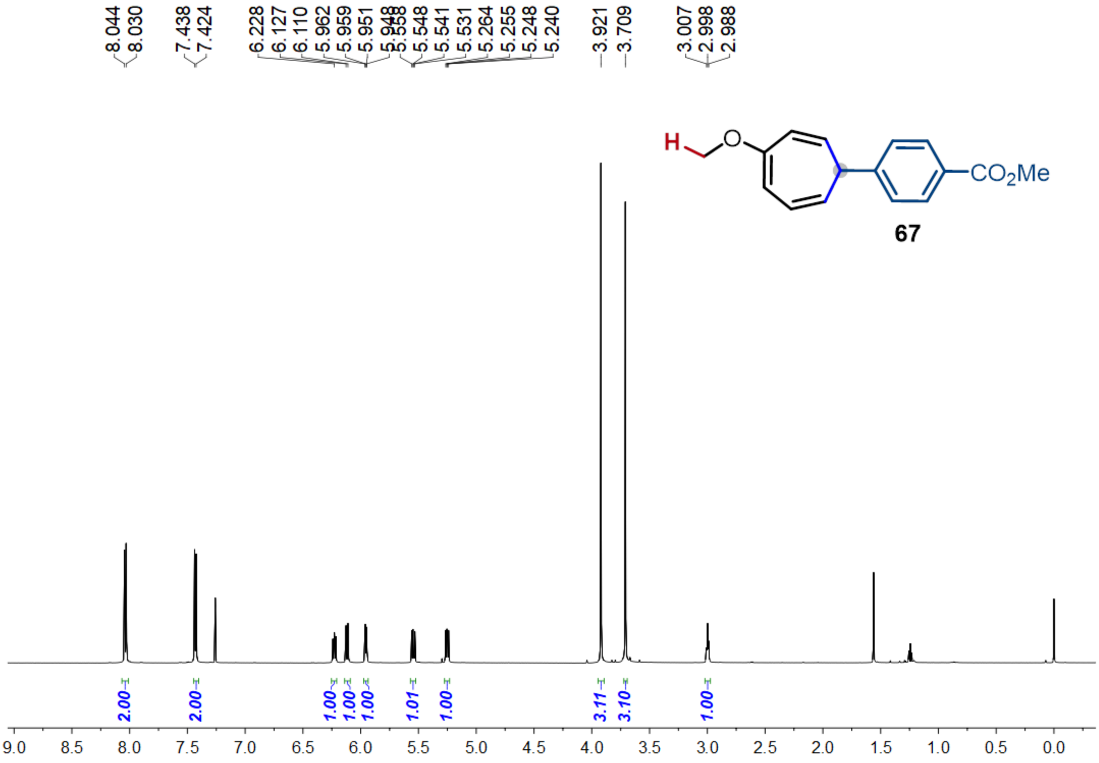

Supplementary Fig. 152 ^{1}**H NMR** (600 MHz, $CDCl_3$) spectrum of compound **67**

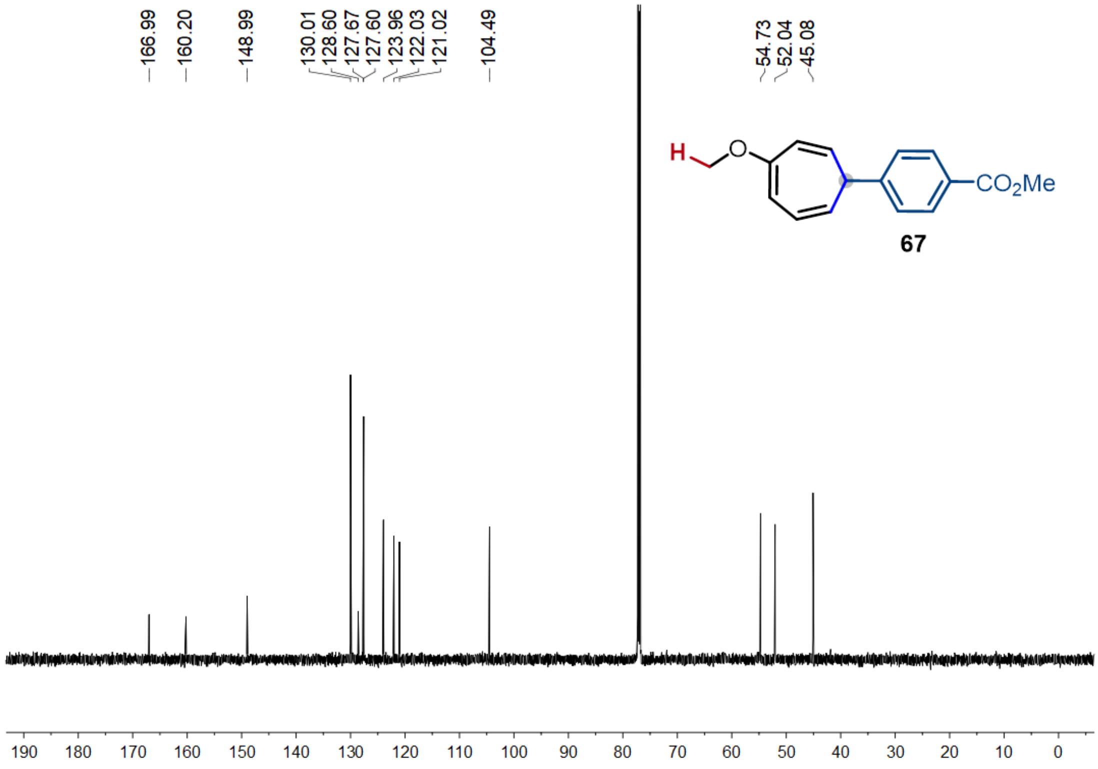

Supplementary Fig. 153 ^{13}**C NMR** (151 MHz, $CDCl_3$) spectrum of compound **67**

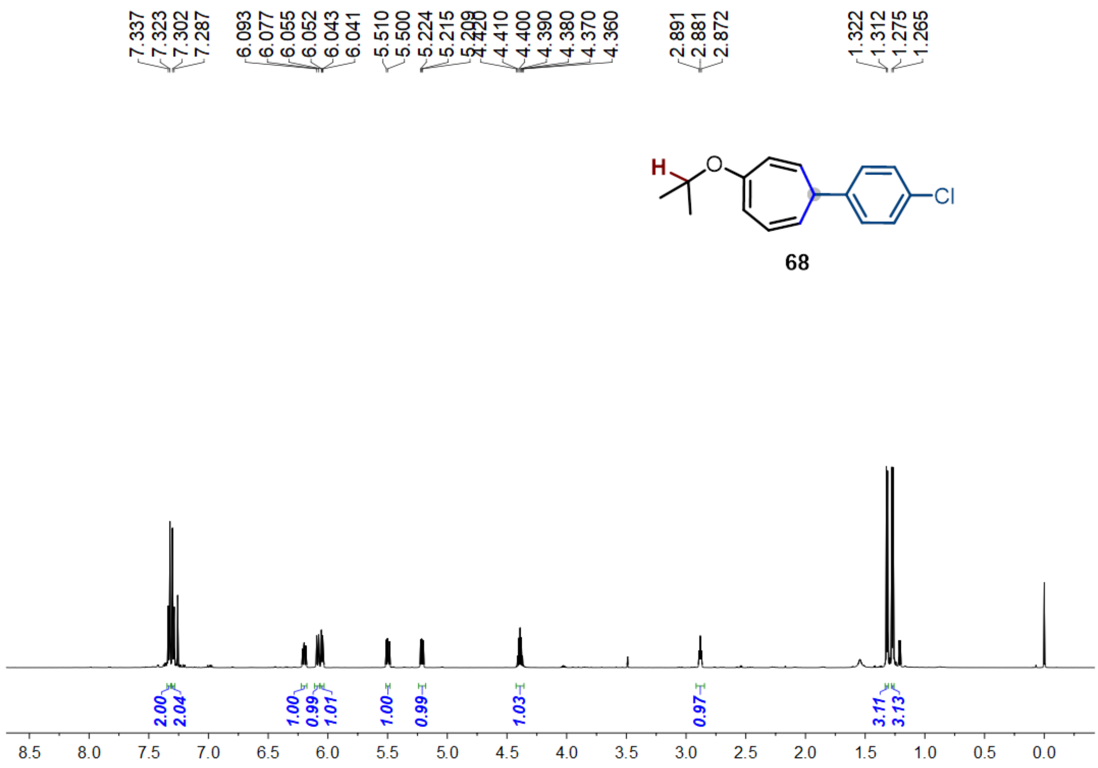

Supplementary Fig. 154 **^{1}H NMR** (600 MHz, $CDCl_3$) spectrum of compound **68**

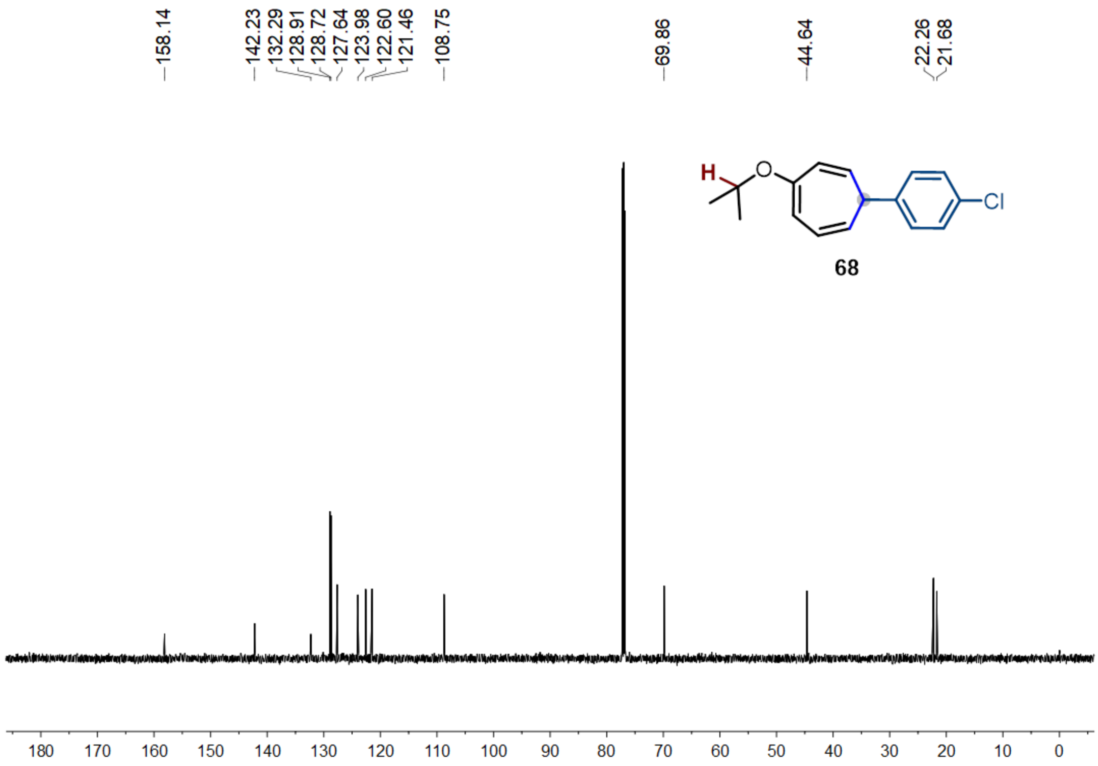

Supplementary Fig. 155 **^{13}C NMR** (151 MHz, $CDCl_3$) spectrum of compound **68**

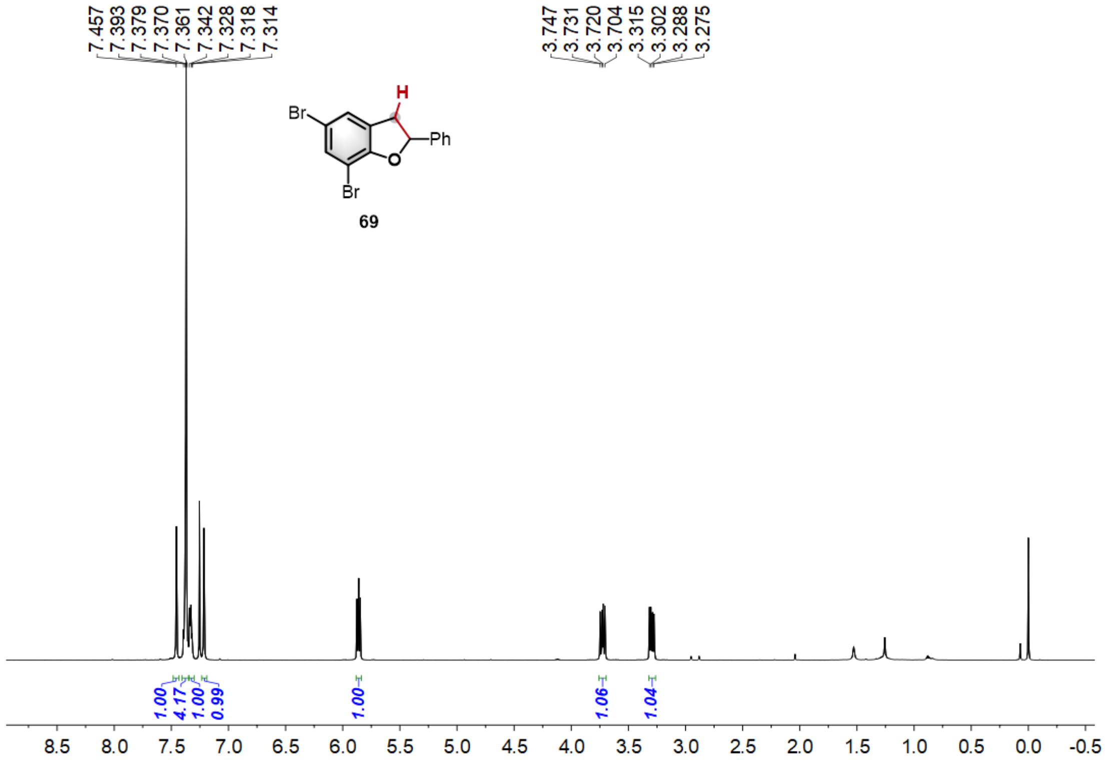

Supplementary Fig. 156 ^{1}H NMR (600 MHz, $CDCl_3$) spectrum of compound **69**

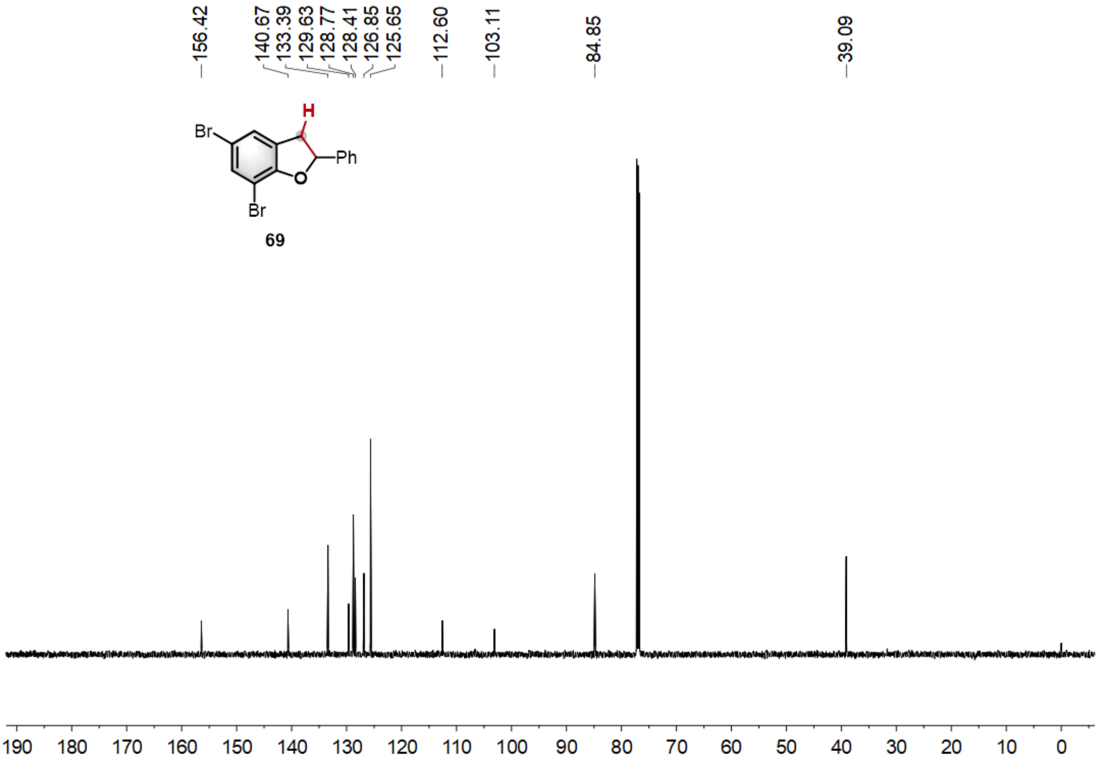

Supplementary Fig. 157 ^{13}C NMR (151 MHz, $CDCl_3$) spectrum of compound **69**

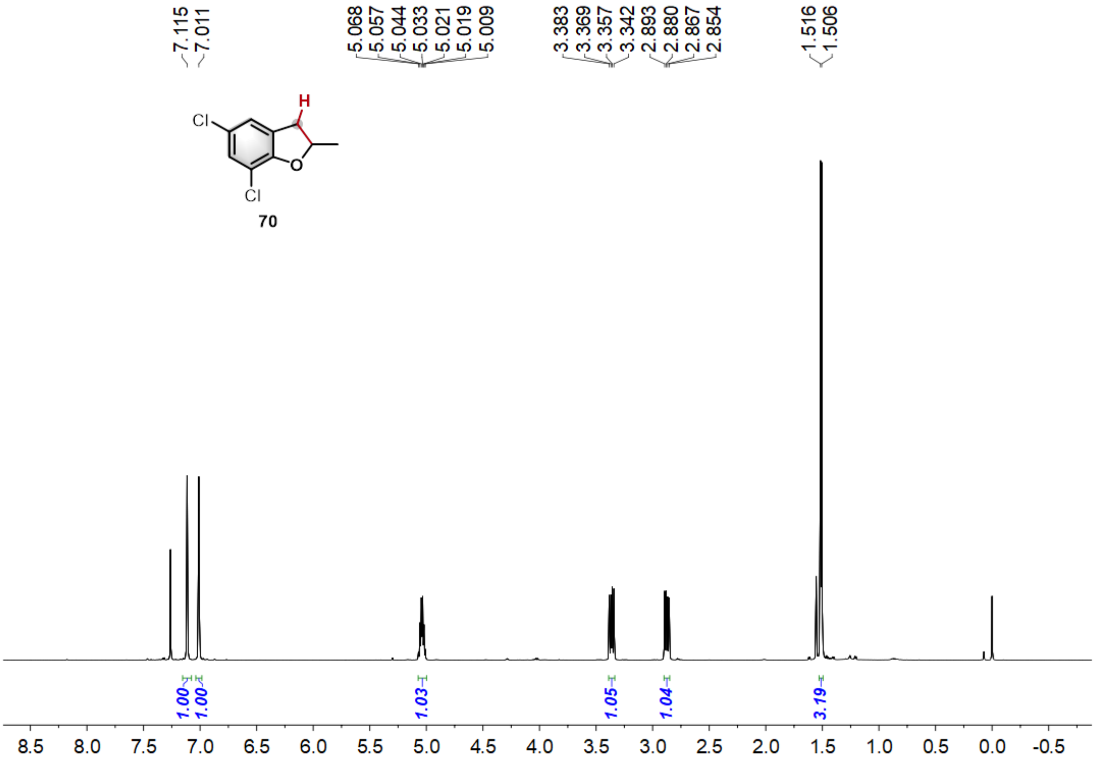

Supplementary Fig. 158 ^{1}H NMR (600 MHz, $CDCl_3$) spectrum of compound **70**

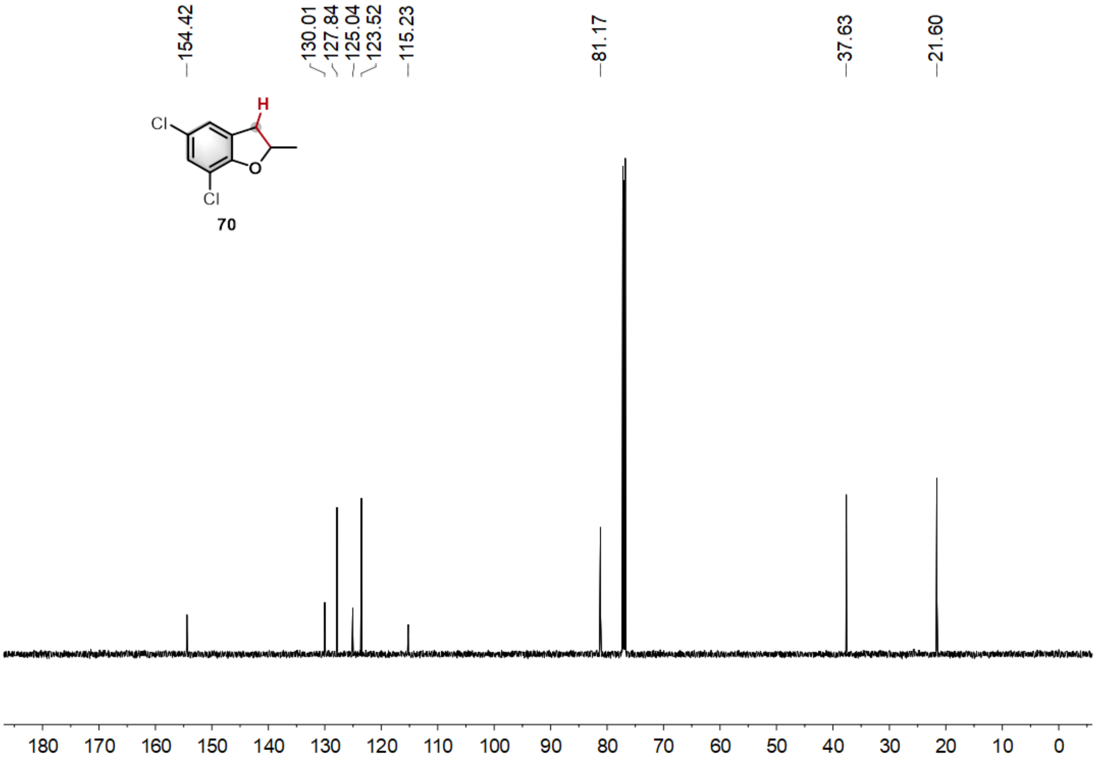

Supplementary Fig. 159 ^{13}C NMR (151 MHz, $CDCl_3$) spectrum of compound **70**

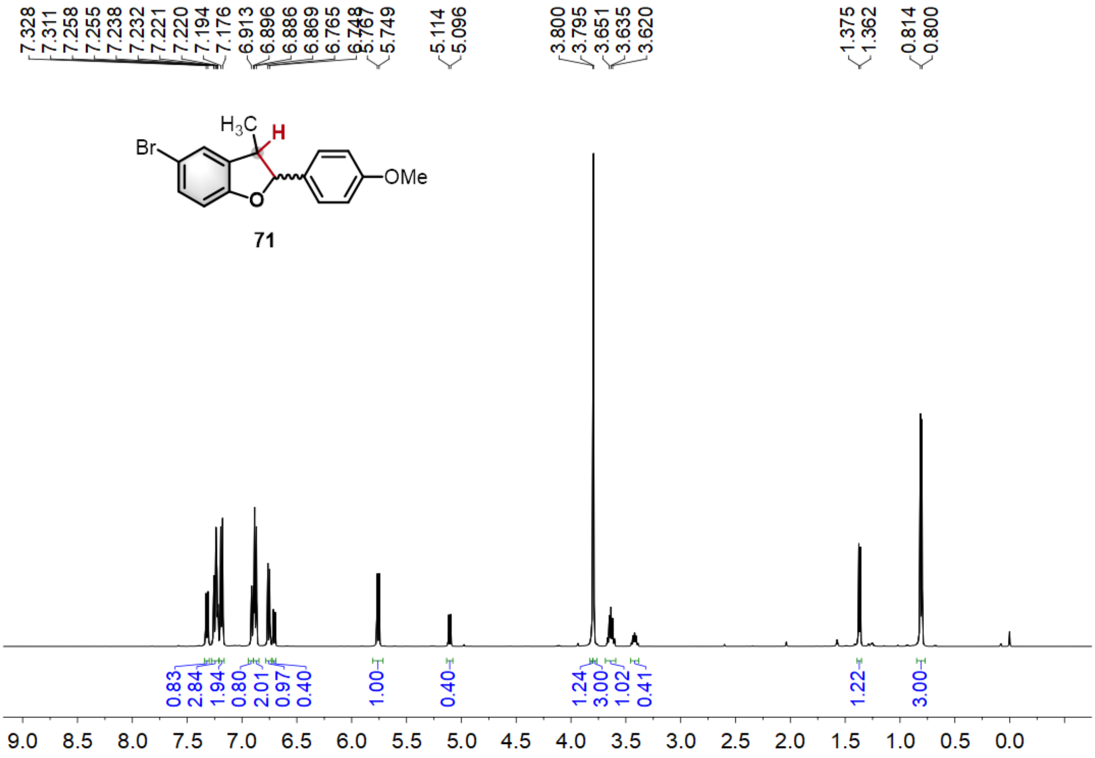

Supplementary Fig. 160 ^{1}**H NMR** (500 MHz, $CDCl_3$) spectrum of compound **71**

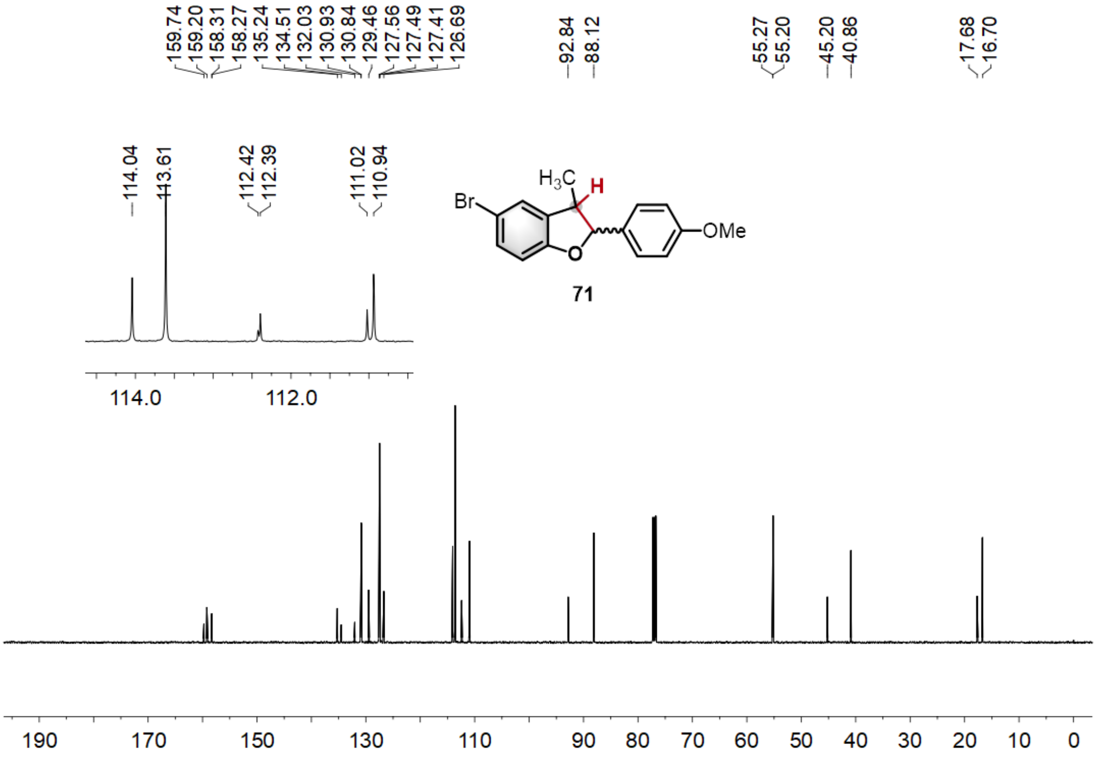

Supplementary Fig. 161 ^{13}**C NMR** (151 MHz, $CDCl_3$) spectrum of compound **71**

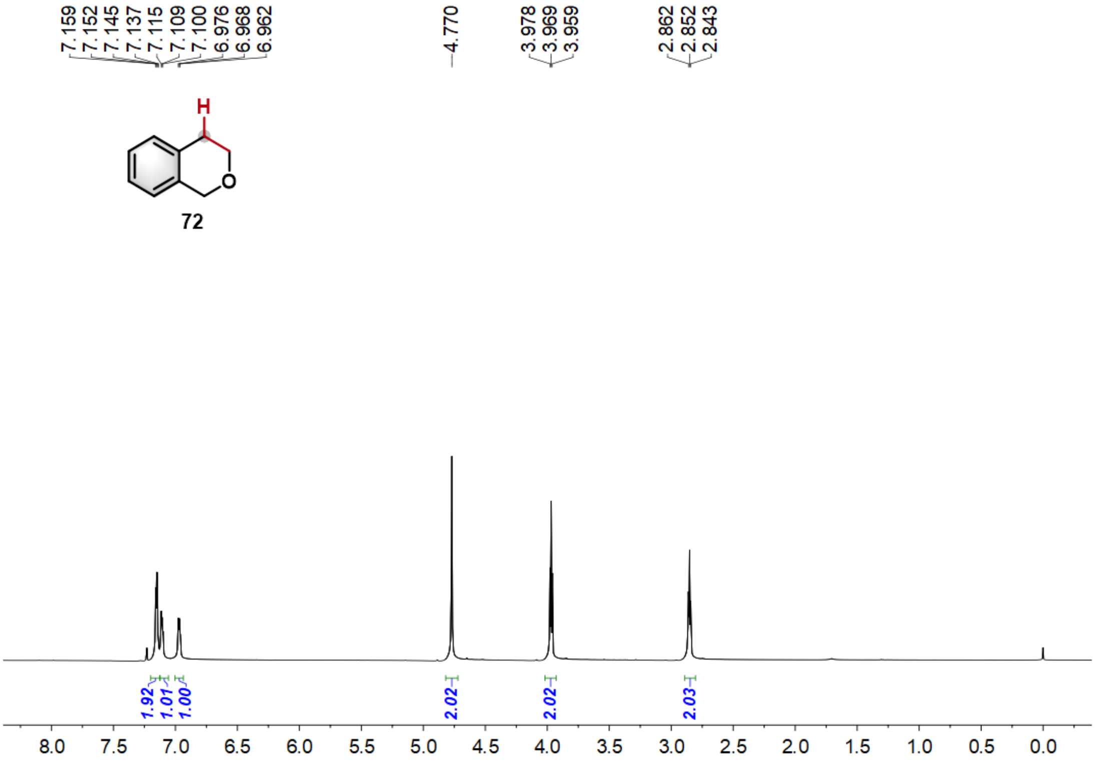

Supplementary Fig. 162 ^{1}H NMR (600 MHz, $CDCl_3$) spectrum of compound **72**

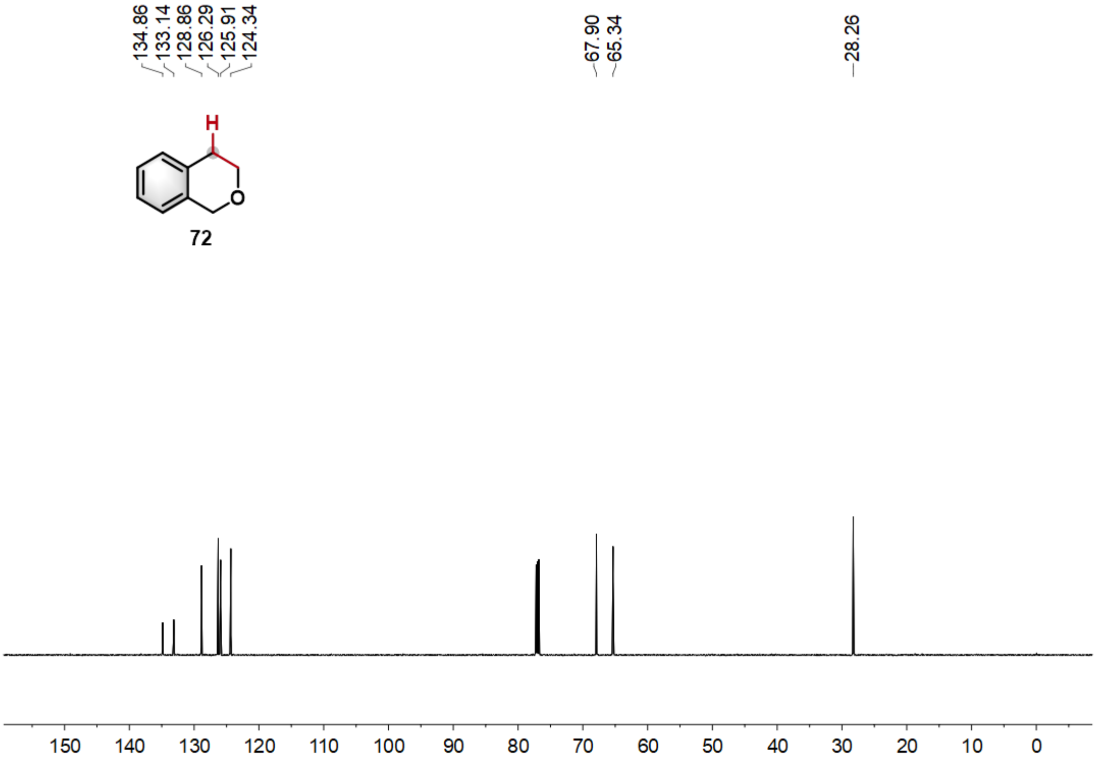

Supplementary Fig. 163 ^{13}C NMR (151 MHz, $CDCl_3$) spectrum of compound **72**

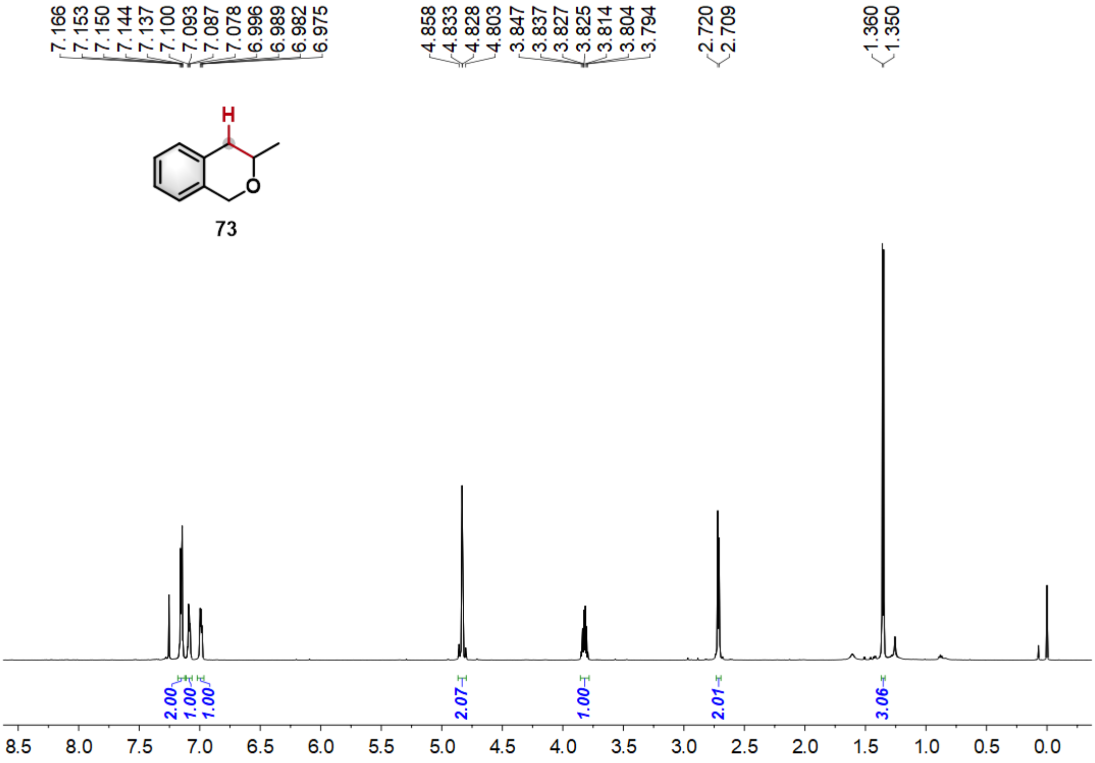

Supplementary Fig. 164 ^{1}**H NMR** (600 MHz, $CDCl_3$) spectrum of compound **73**

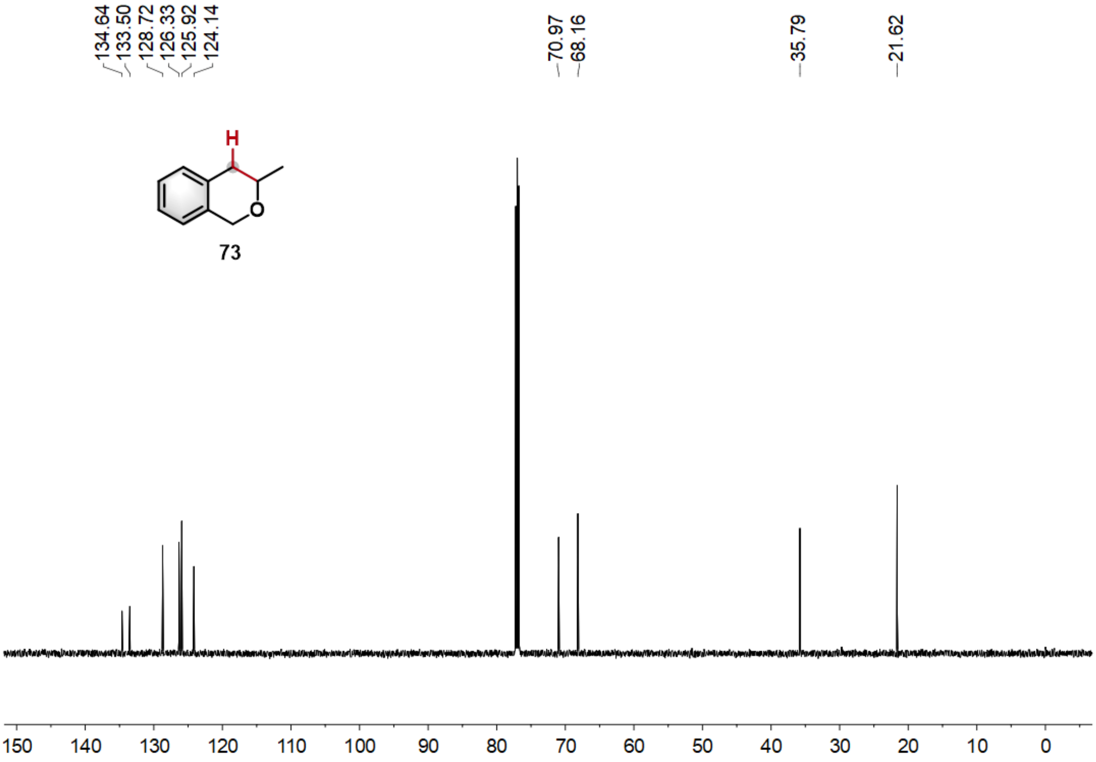

Supplementary Fig. 165 ^{13}**C NMR** (151 MHz, $CDCl_3$) spectrum of compound **73**

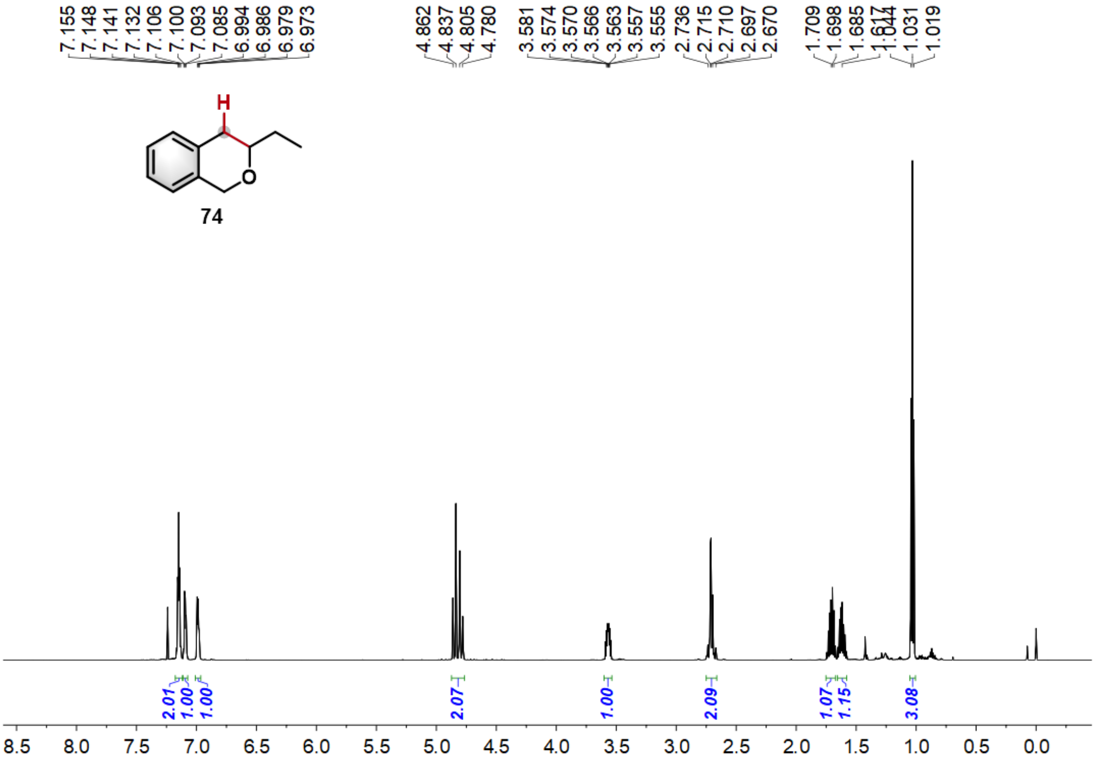

Supplementary Fig. 166 ^{1}H NMR (600 MHz, $CDCl_3$) spectrum of compound **74**

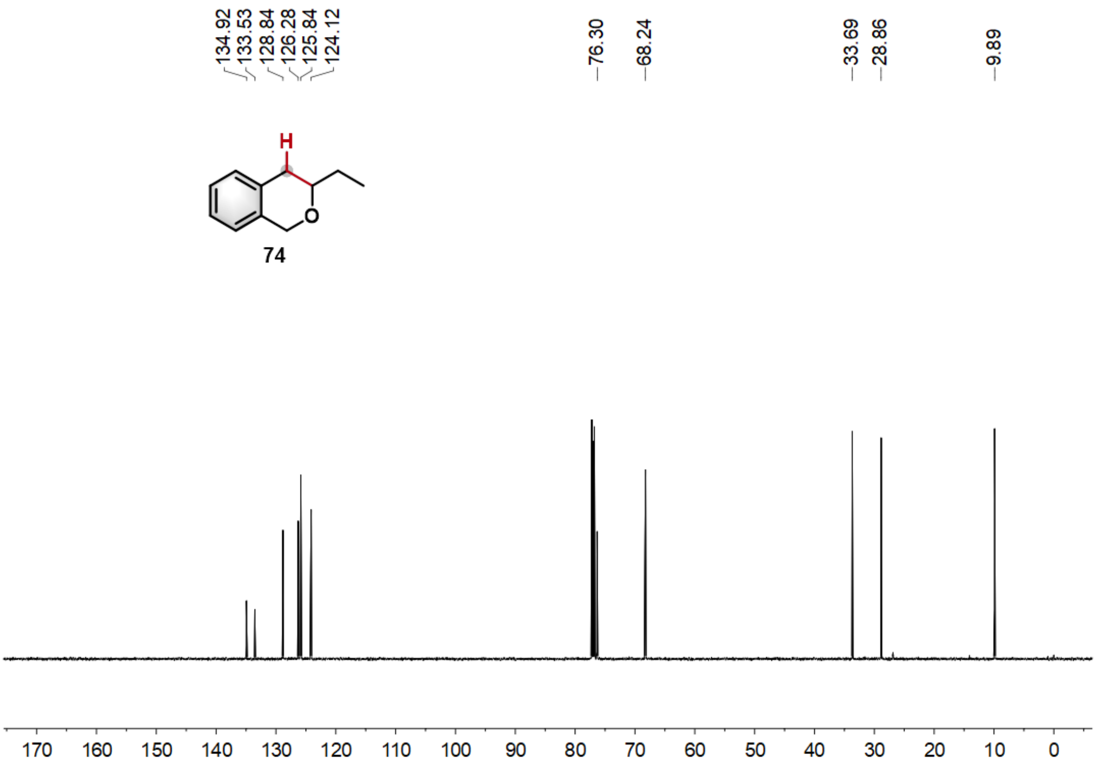

Supplementary Fig. 167 ^{13}C NMR (151 MHz, $CDCl_3$) spectrum of compound **74**

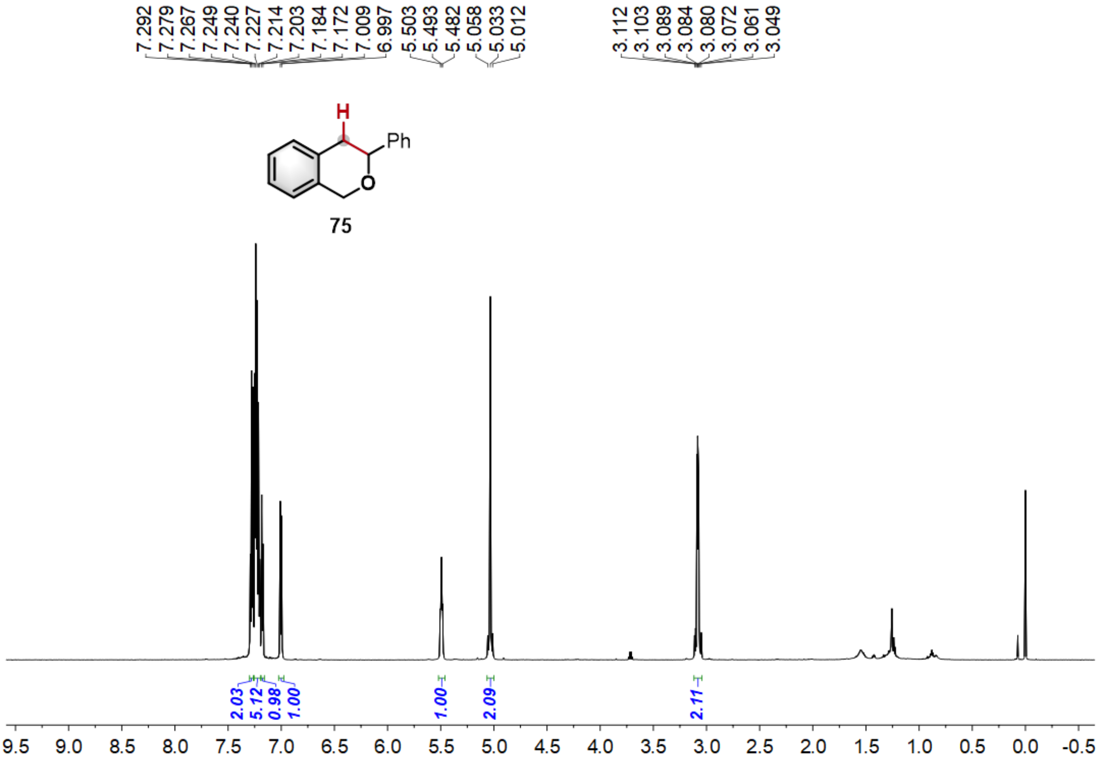

Supplementary Fig. 168 ^{1}**H NMR** (600 MHz, $CDCl_3$) spectrum of compound **75**

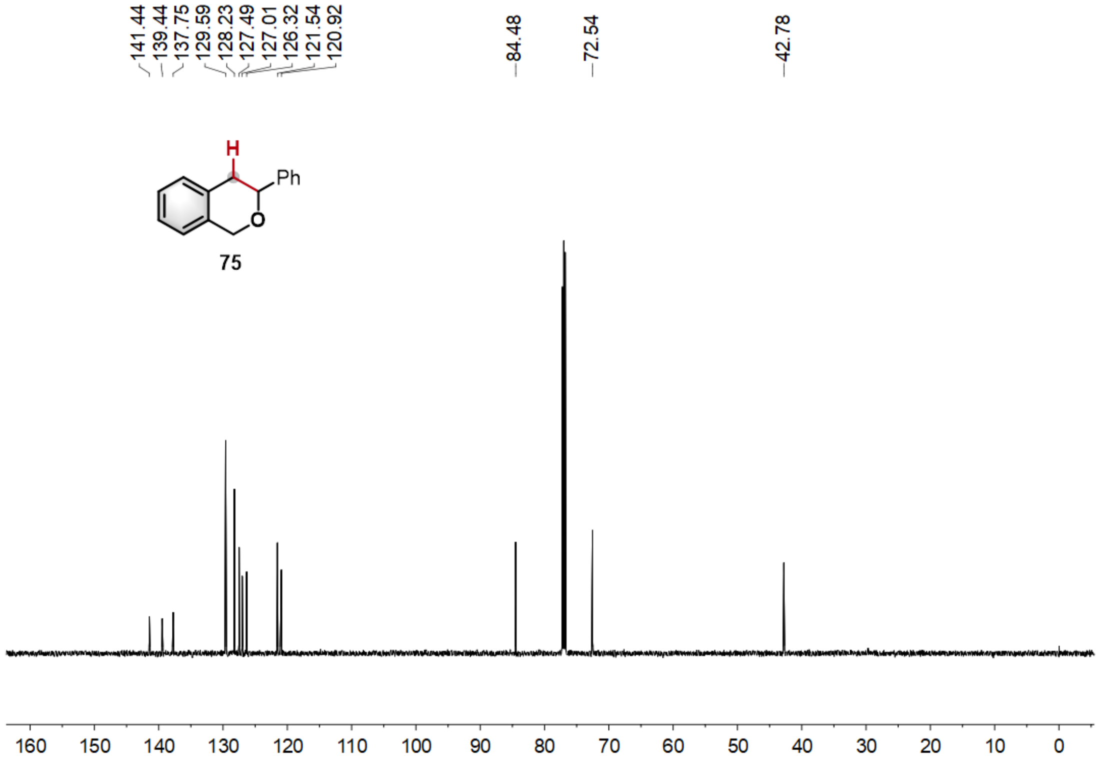

Supplementary Fig. 169 ^{13}**C NMR** (151 MHz, $CDCl_3$) spectrum of compound **75**

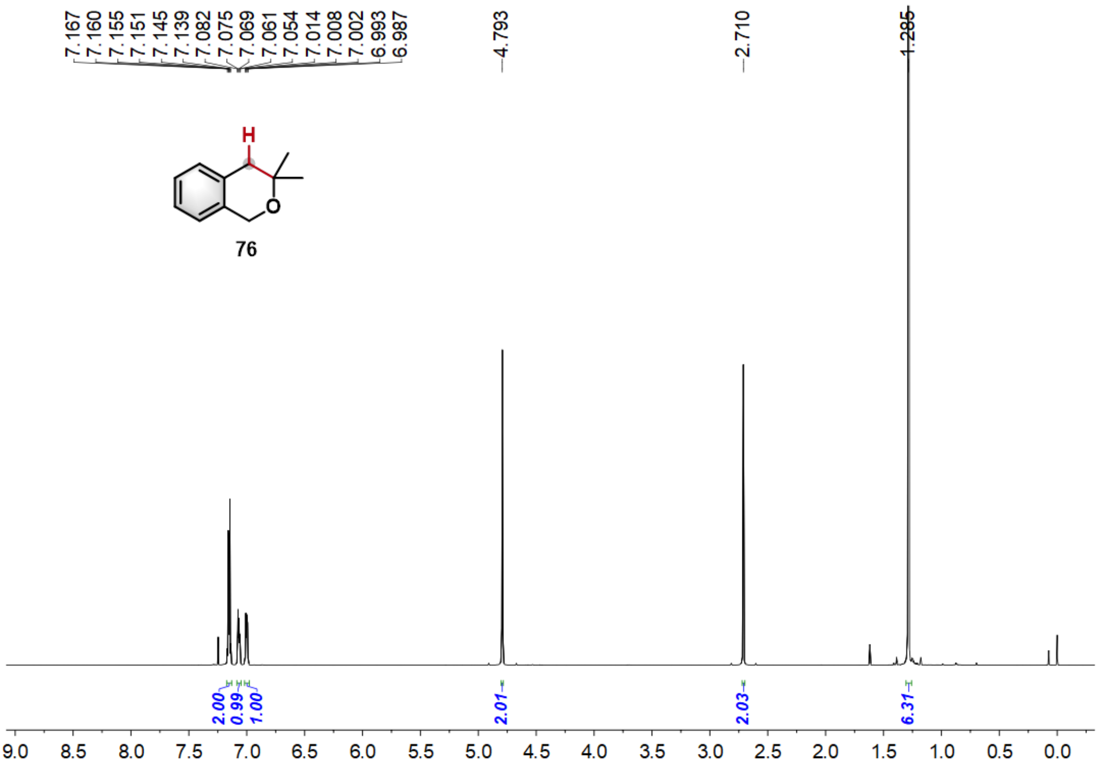

Supplementary Fig. 170 ^{1}**H NMR** (600 MHz, $CDCl_3$) spectrum of compound **76**

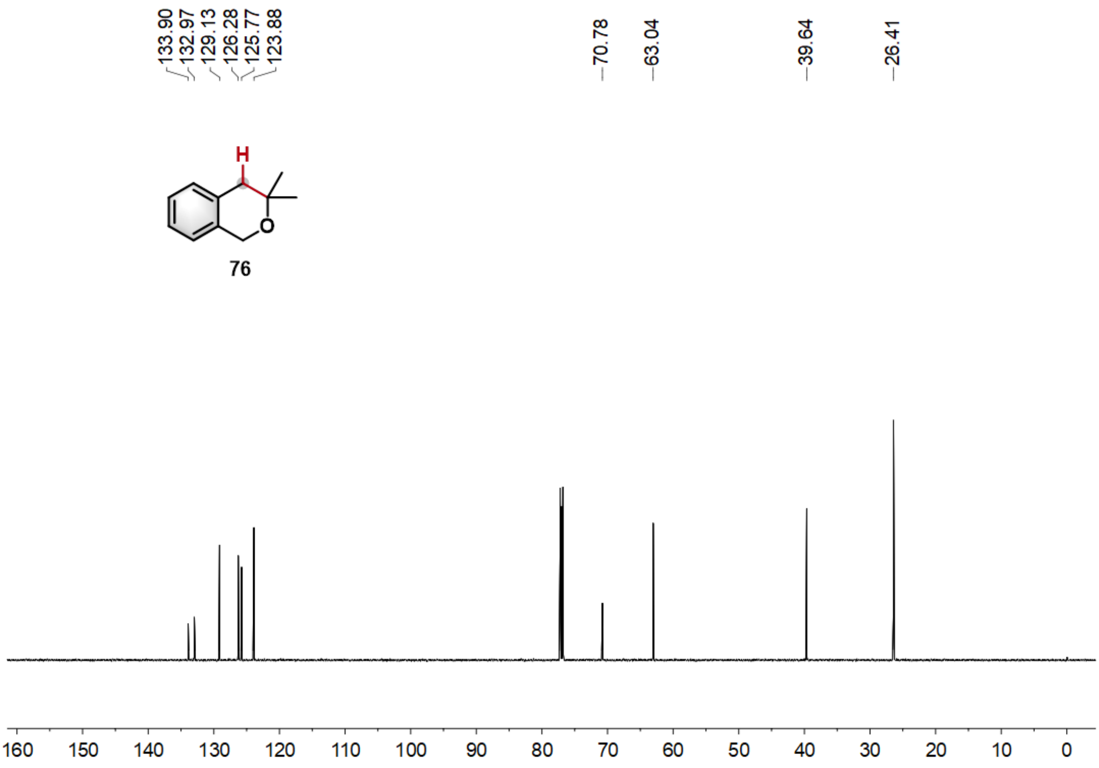

Supplementary Fig. 171 ^{13}**C NMR** (151 MHz, $CDCl_3$) spectrum of compound **76**

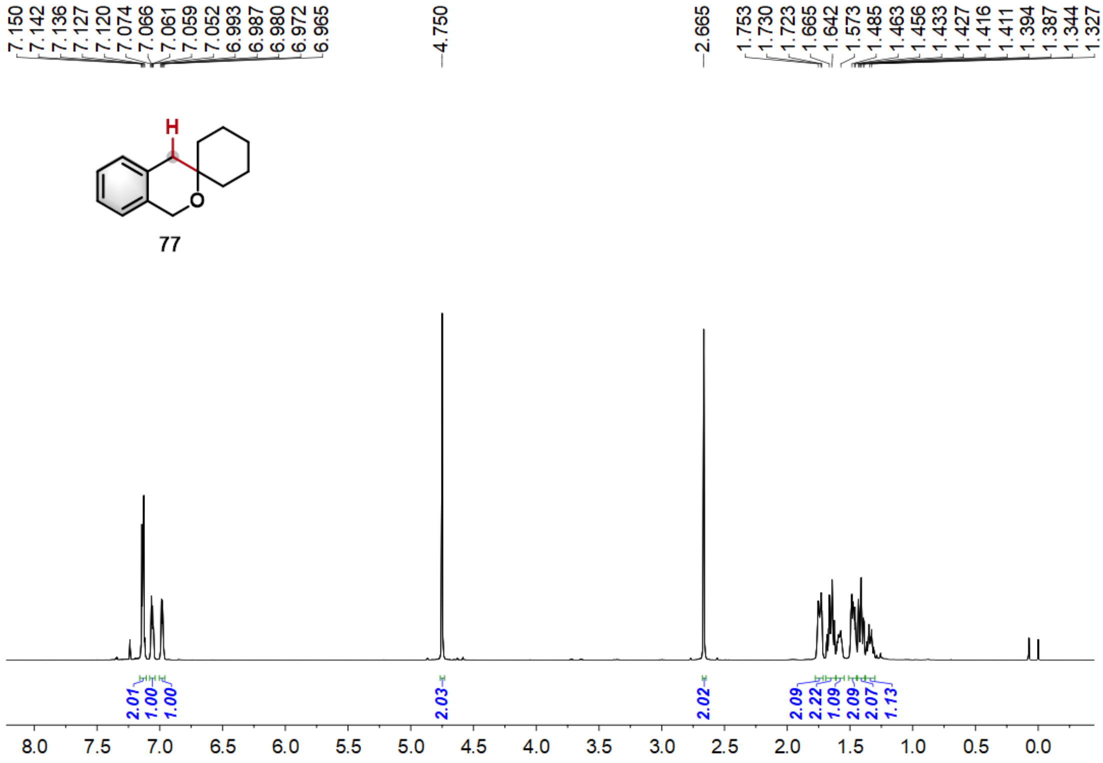

Supplementary Fig. 172 ^{1}H NMR (600 MHz, $CDCl_3$) spectrum of compound **77**

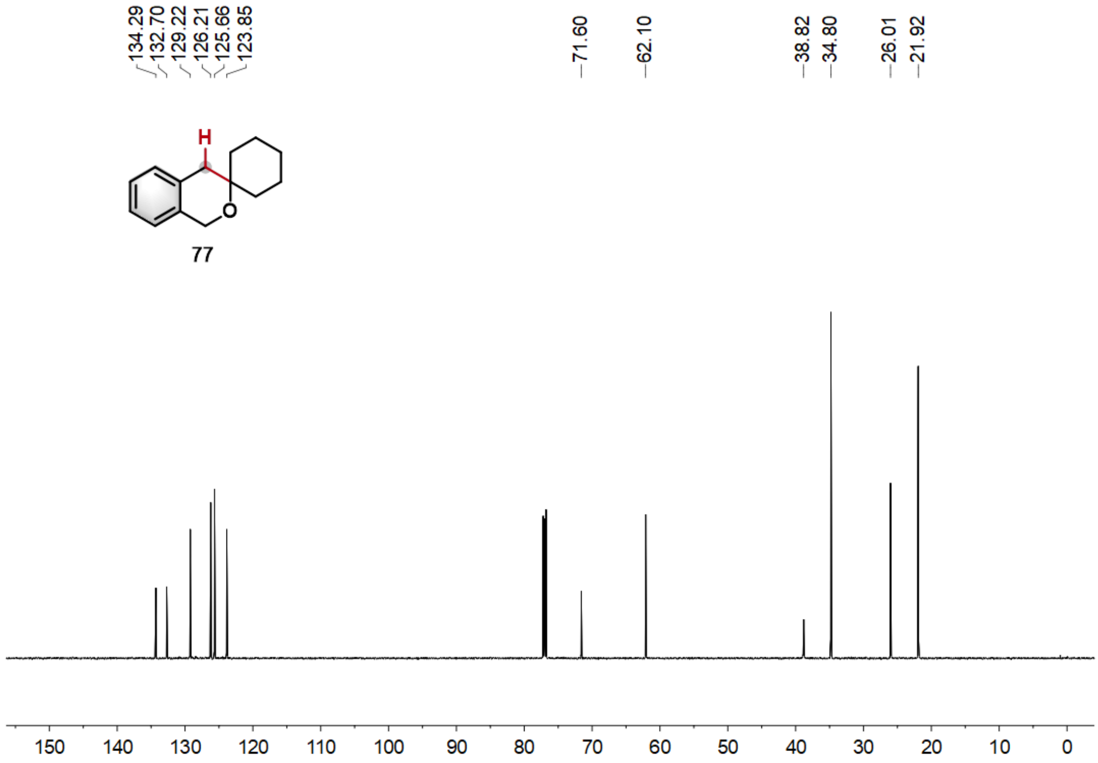

Supplementary Fig. 173 ^{13}C NMR (151 MHz, $CDCl_3$) spectrum of compound **77**

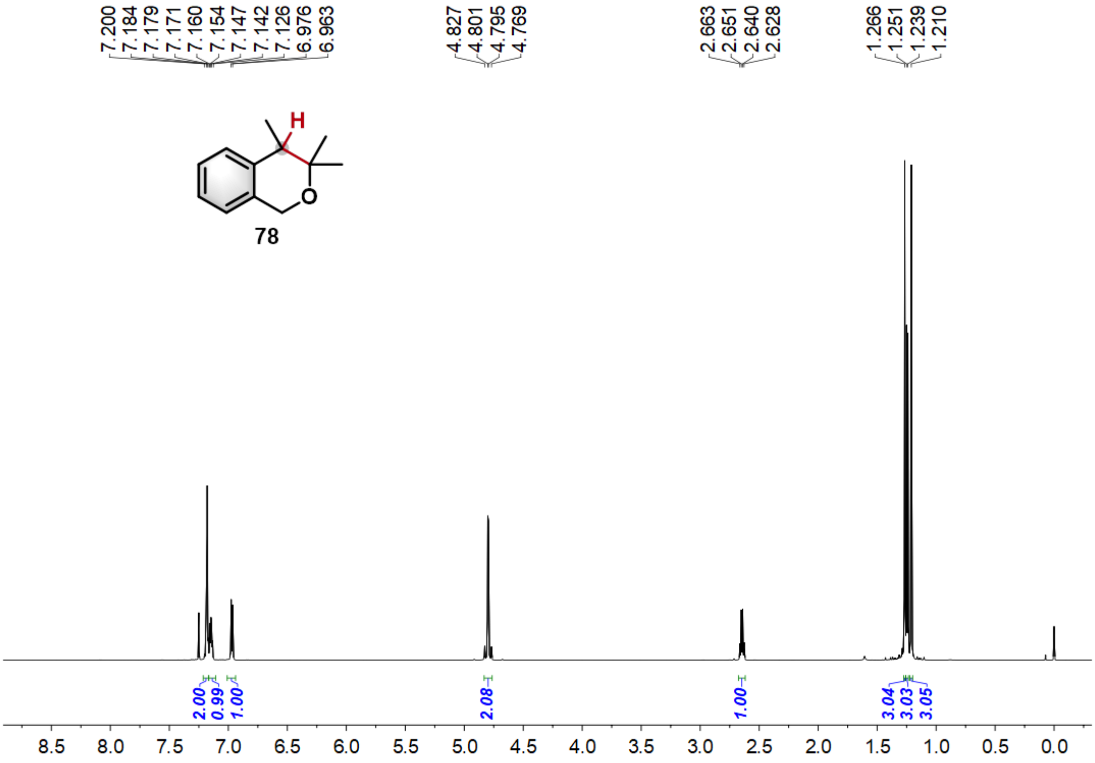

Supplementary Fig. 174 ^{1}H NMR (600 MHz, $CDCl_3$) spectrum of compound **78**

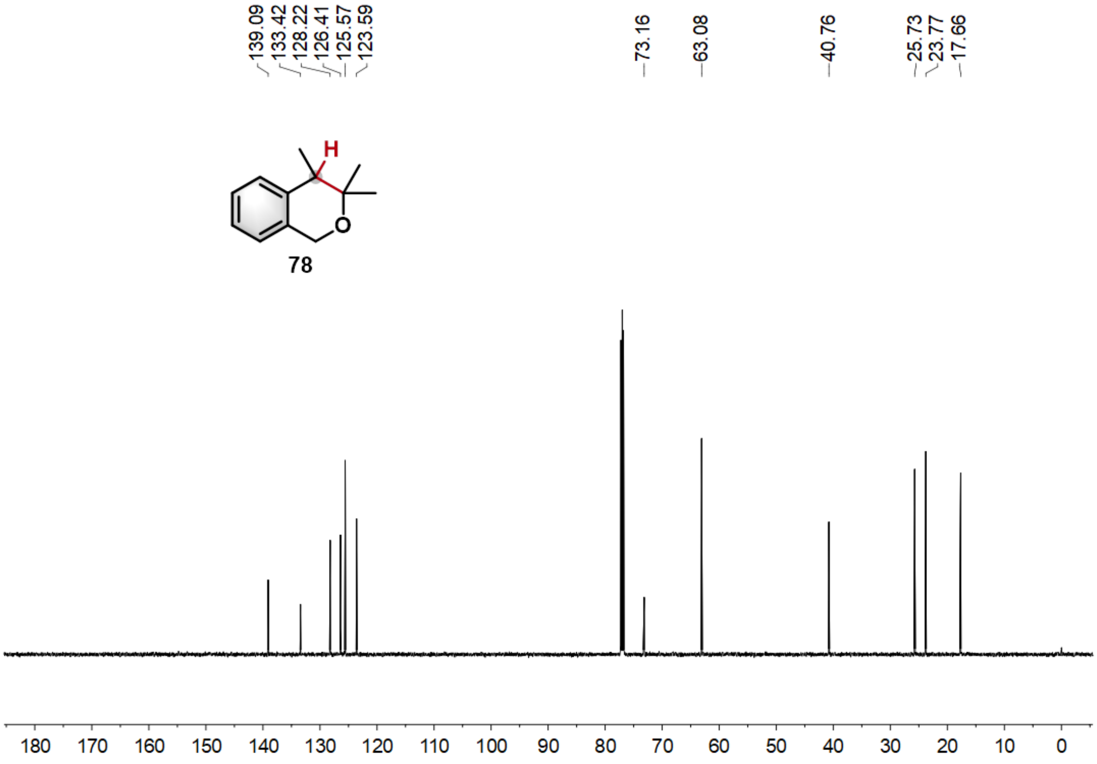

Supplementary Fig. 175 ^{13}C NMR (151 MHz, $CDCl_3$) spectrum of compound **78**

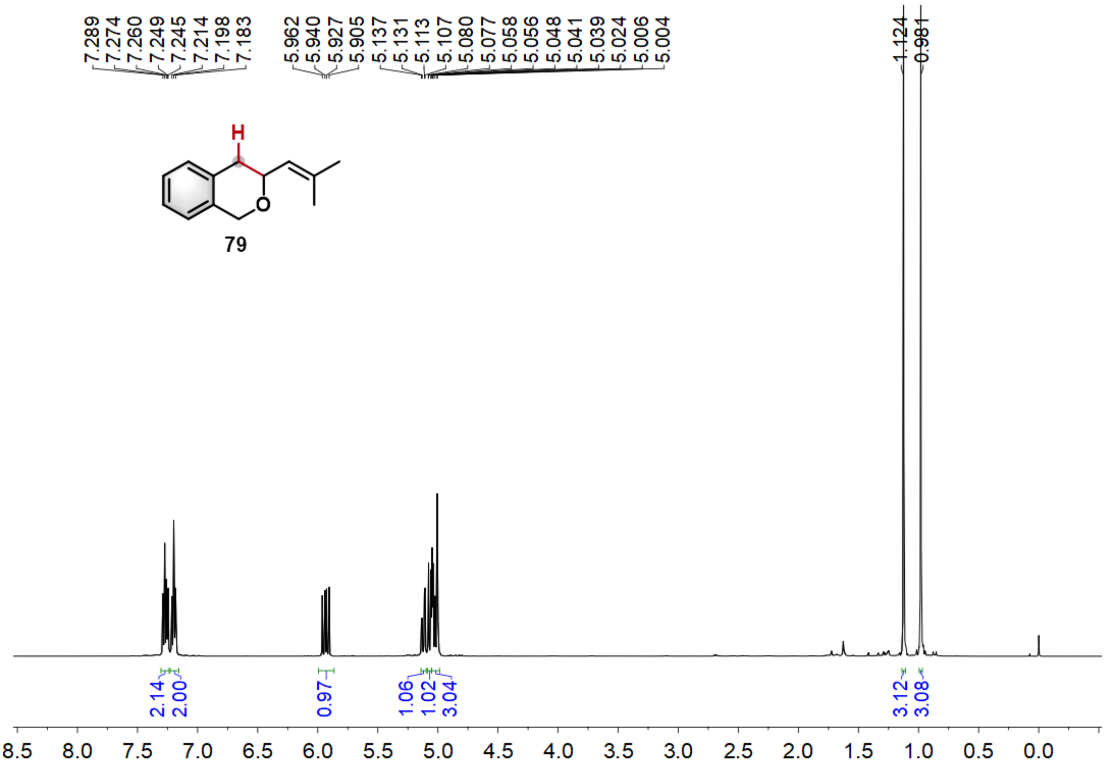

Supplementary Fig. 176 ^{1}H NMR (500 MHz, $CDCl_3$) spectrum of compound **79**

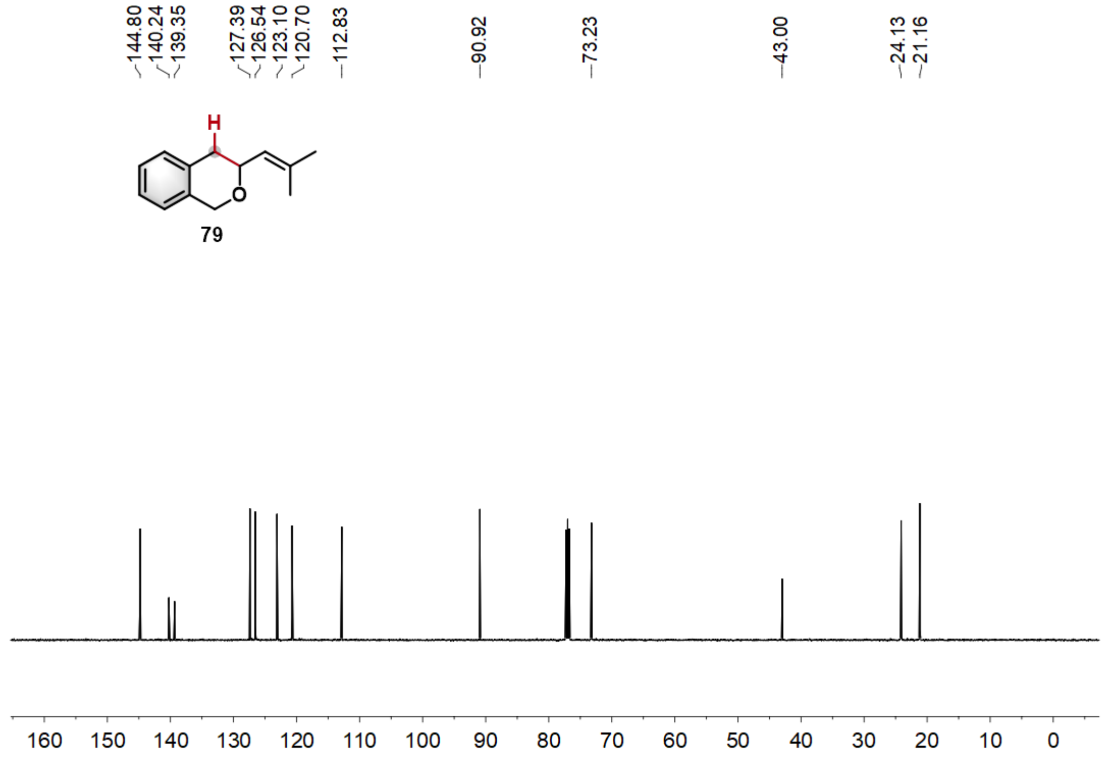

Supplementary Fig. 177 ^{13}C NMR (151 MHz, $CDCl_3$) spectrum of compound **79**

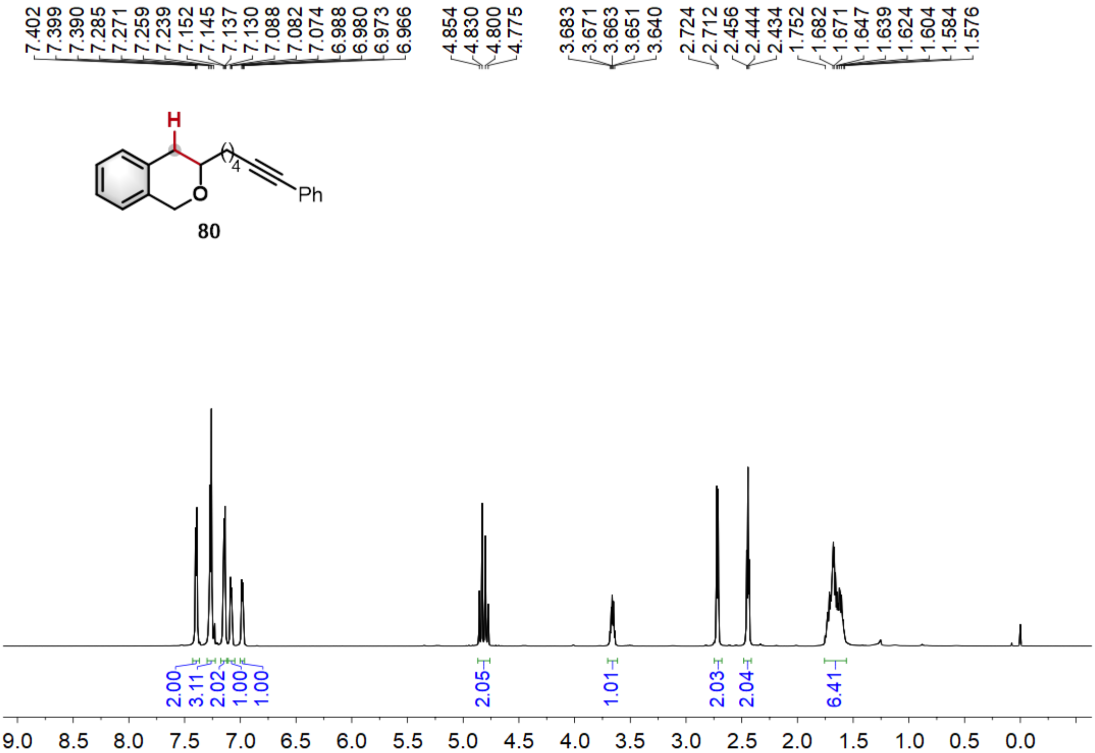

Supplementary Fig. 178 ^{1}**H NMR** (600 MHz, $CDCl_3$) spectrum of compound **80**

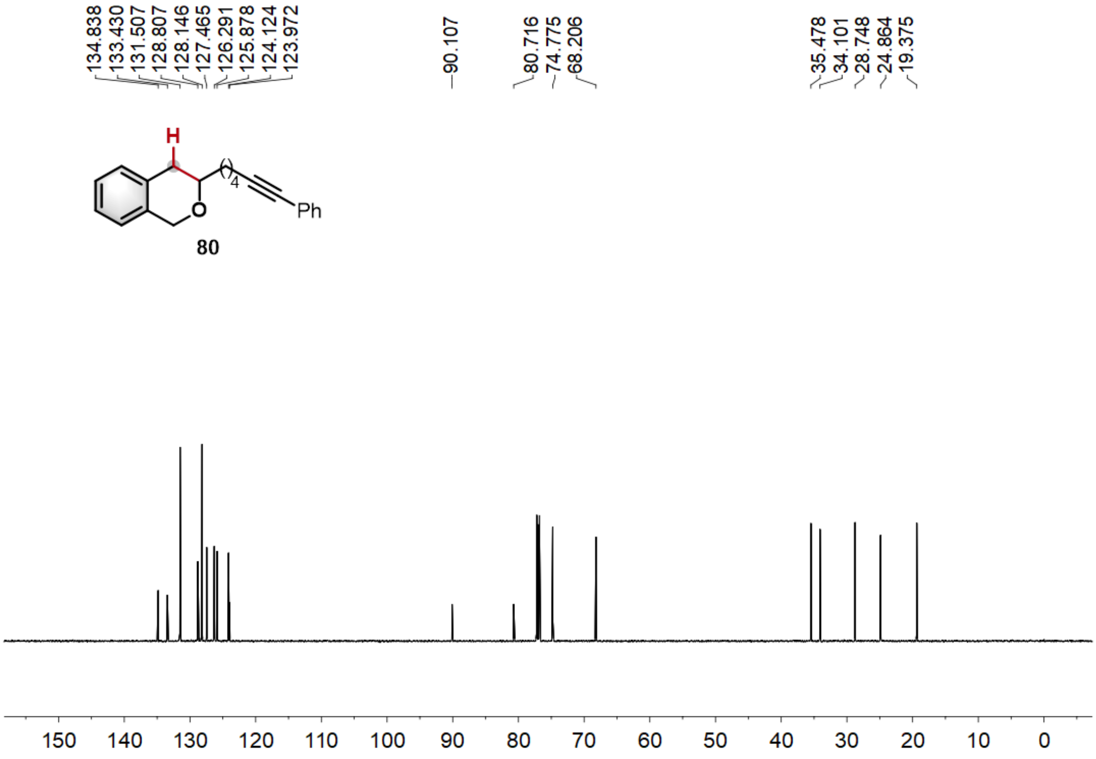

Supplementary Fig. 179 ^{13}**C NMR** (151 MHz, $CDCl_3$) spectrum of compound **80**

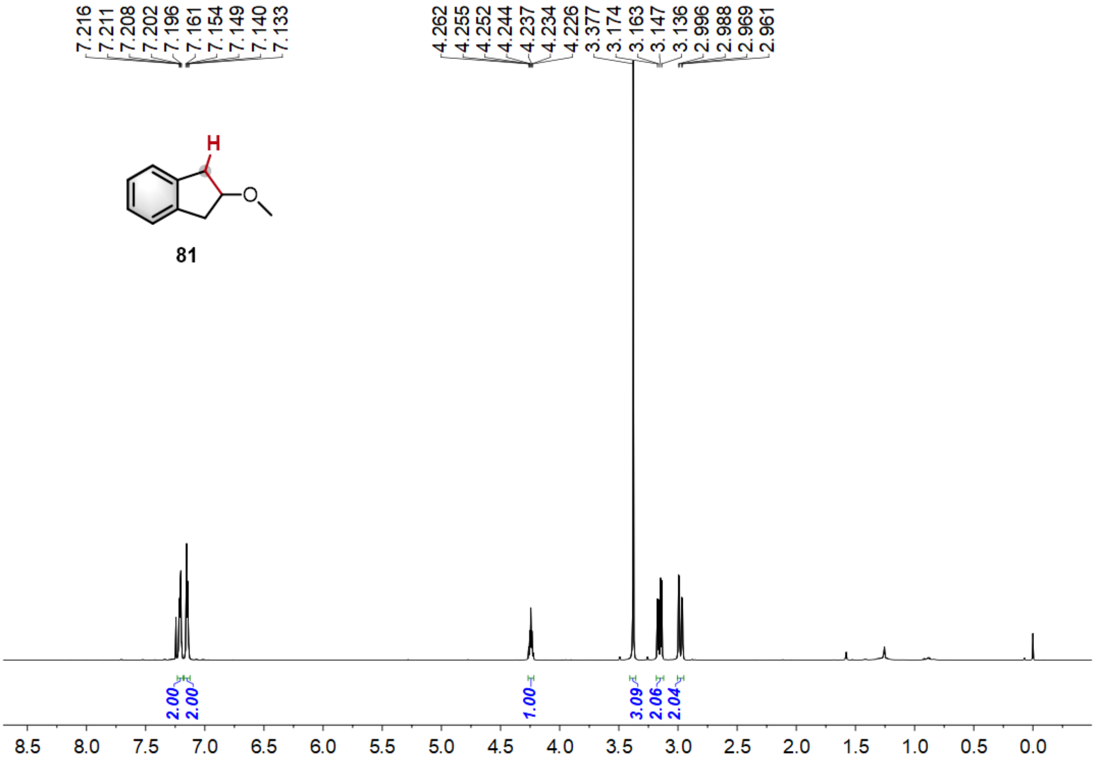

Supplementary Fig. 180 ^{1}H NMR (600 MHz, $CDCl_3$) spectrum of compound **81**

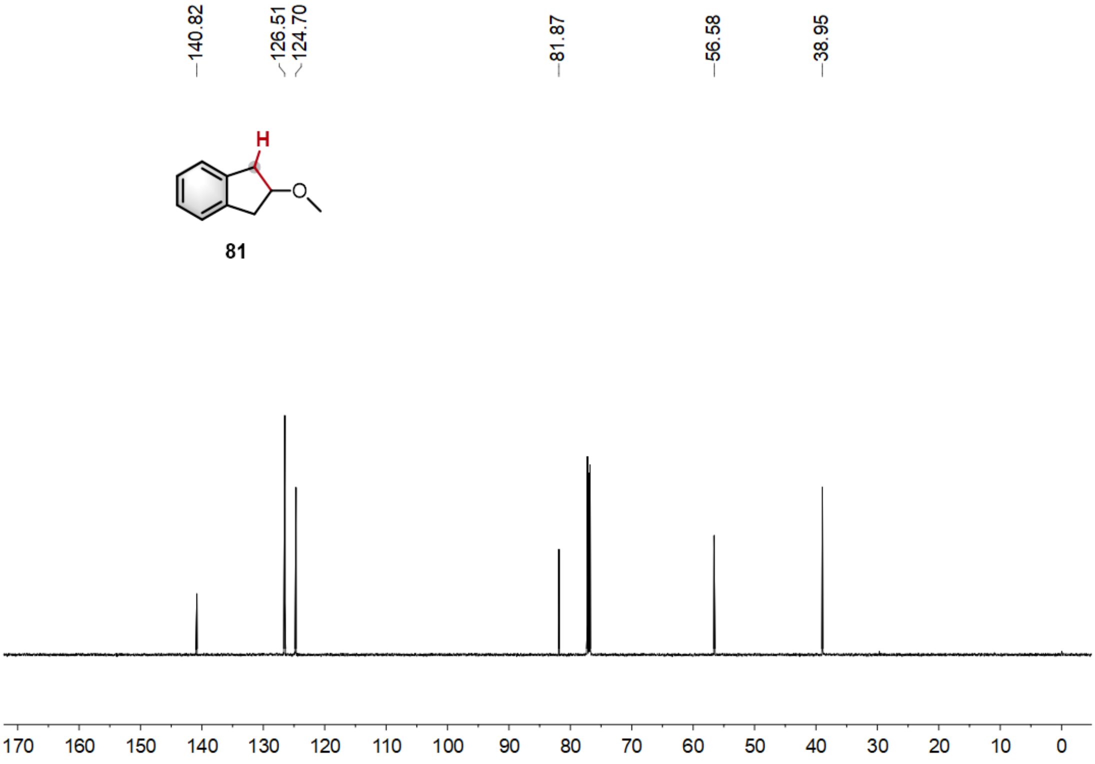

Supplementary Fig. 181 ^{13}C NMR (151 MHz, $CDCl_3$) spectrum of compound **81**

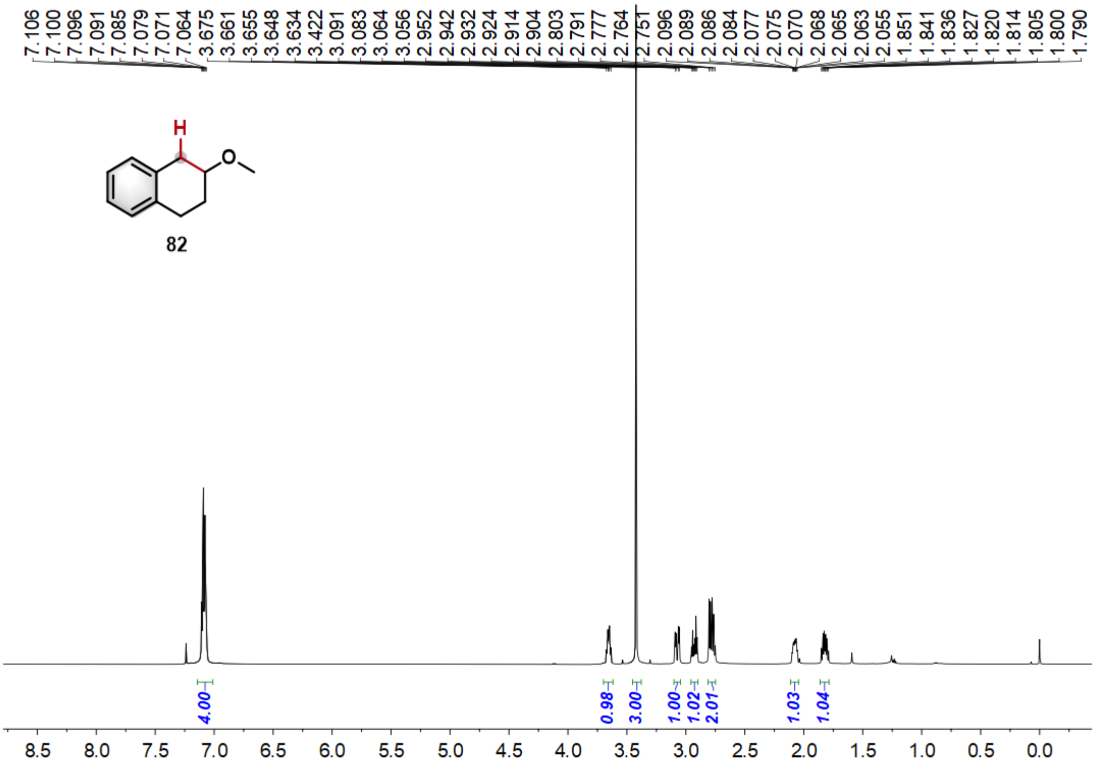

Supplementary Fig. 182 ^{1}**H NMR** (600 MHz, $CDCl_3$) spectrum of compound **82**

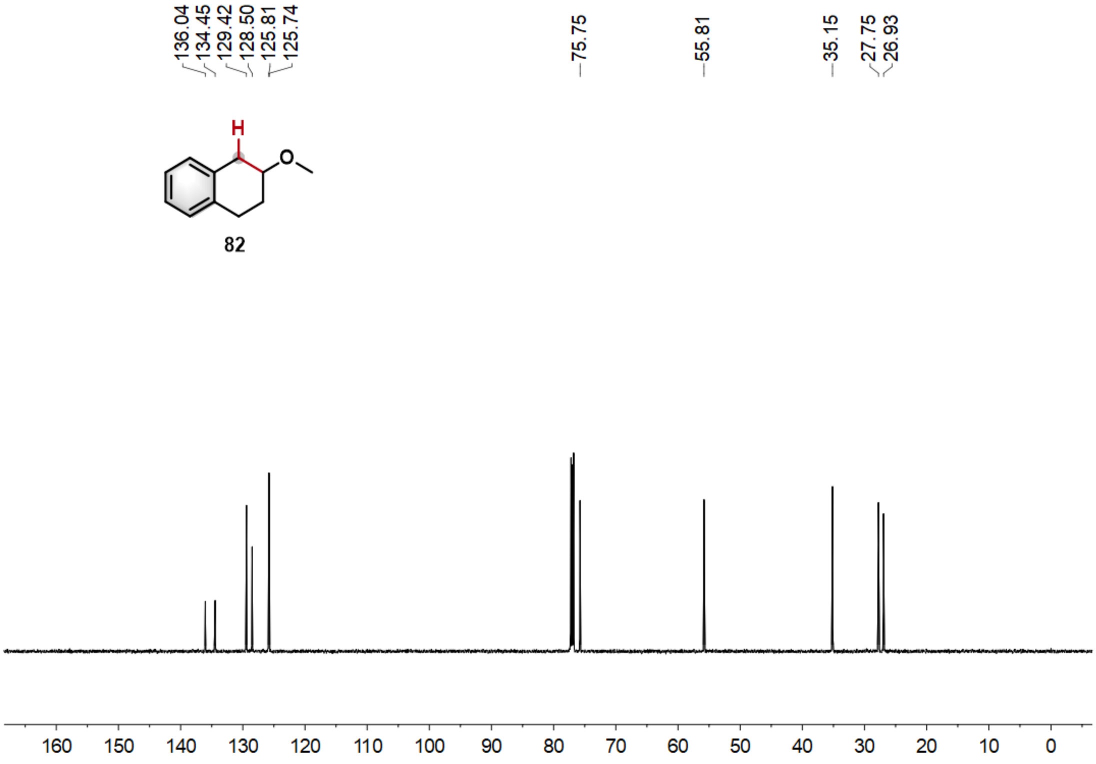

Supplementary Fig. 183 ^{13}**C NMR** (151 MHz, $CDCl_3$) spectrum of compound **82**

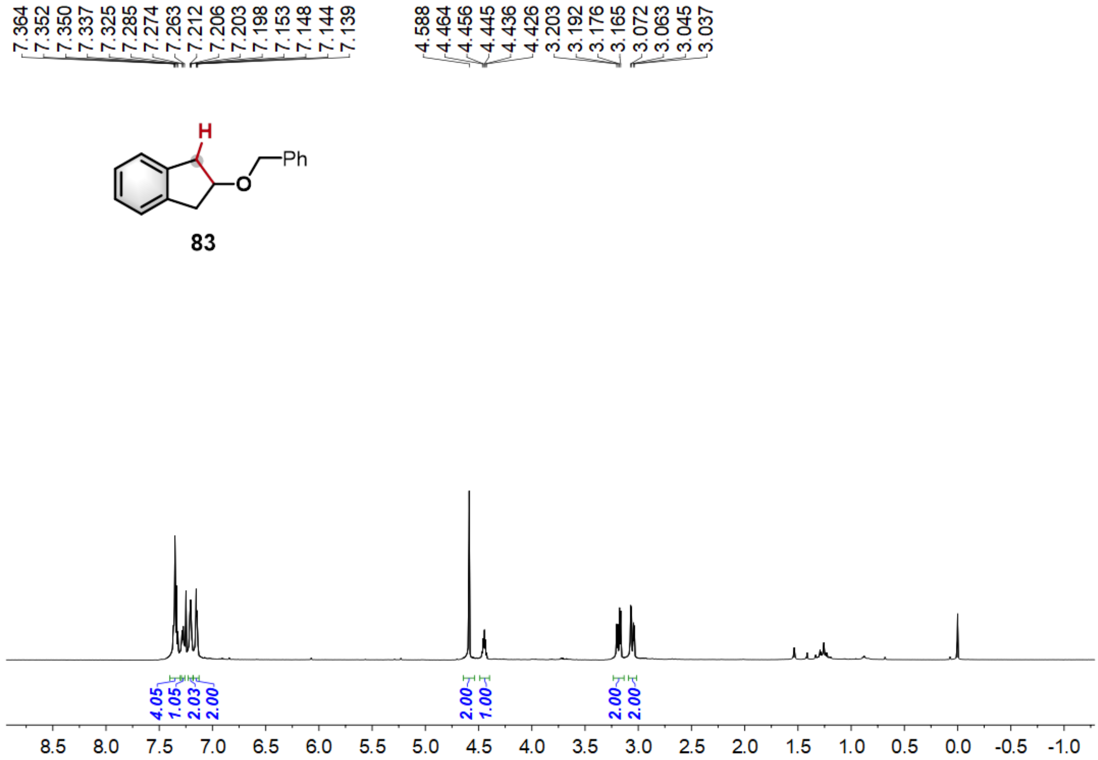

Supplementary Fig. 184 ^{1}H NMR (600 MHz, $CDCl_3$) spectrum of compound **83**

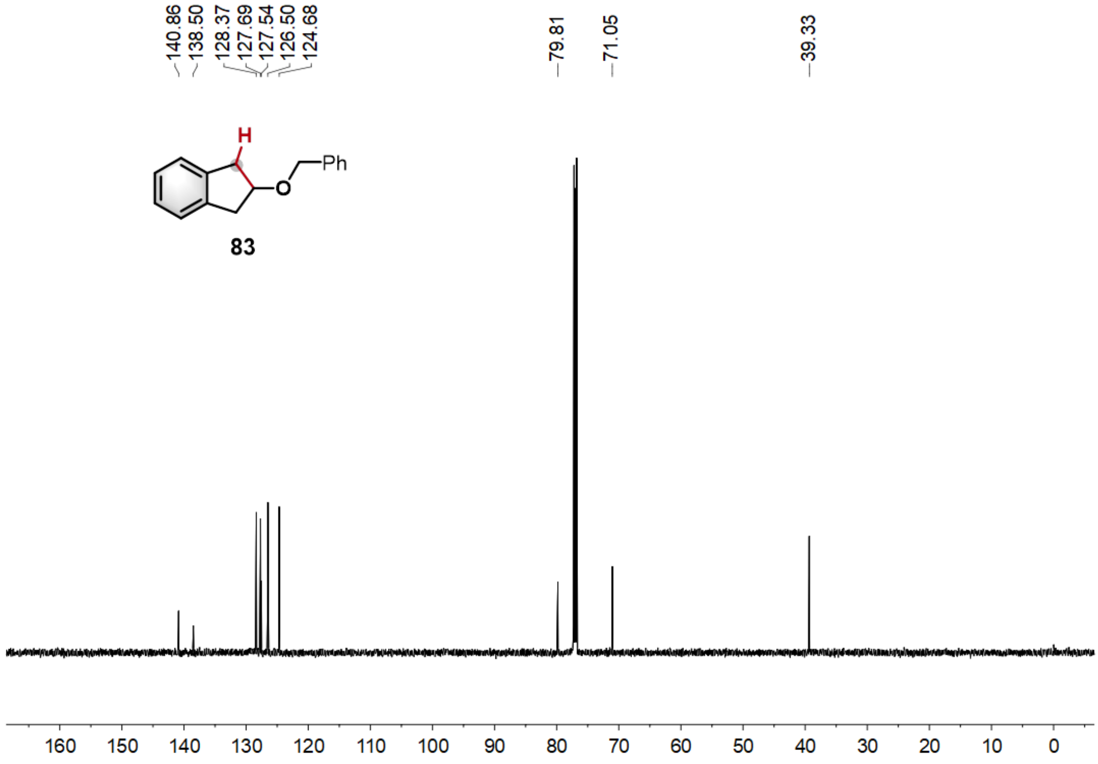

Supplementary Fig. 185 ^{13}C NMR (151 MHz, $CDCl_3$) spectrum of compound

83

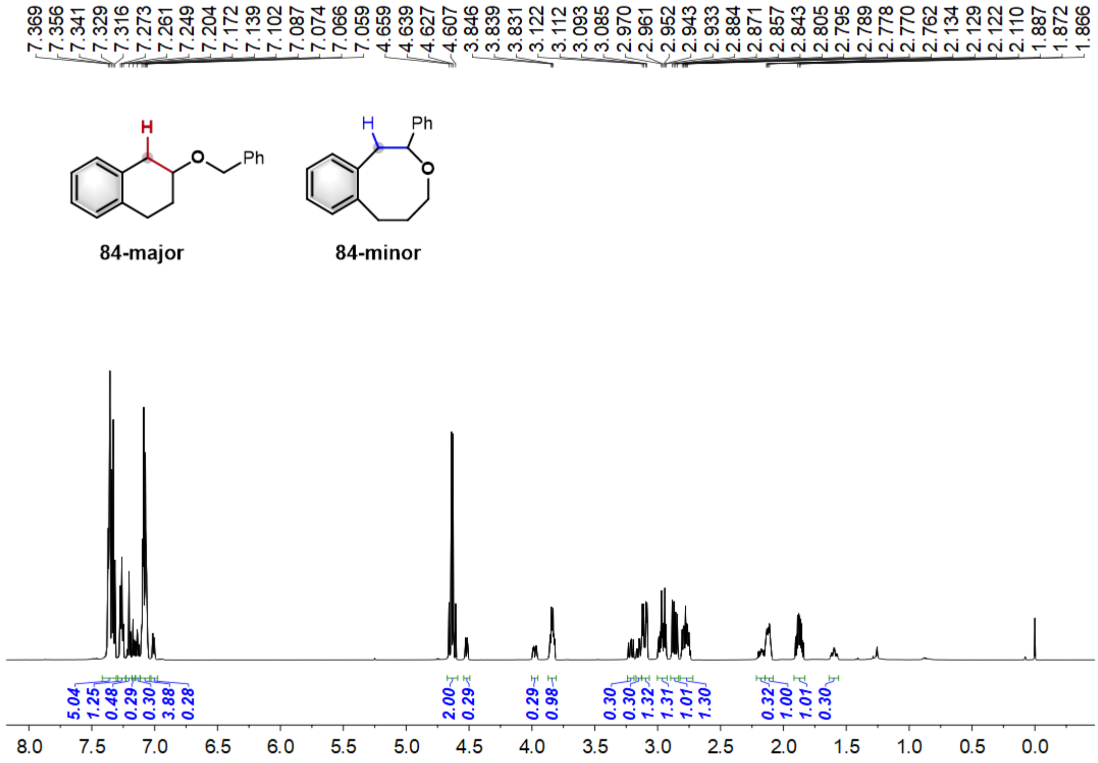

Supplementary Fig. 186 ^{1}**H NMR** (600 MHz, $CDCl_3$) spectrum of compound **84**

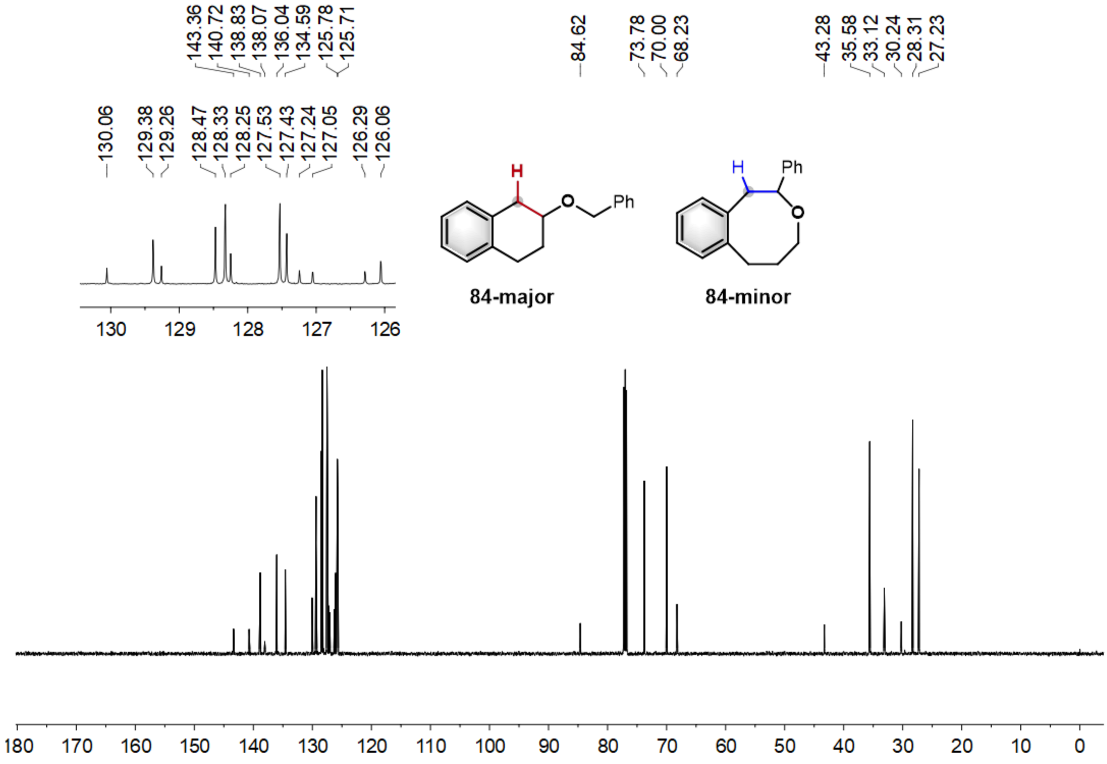

Supplementary Fig. 187 ^{13}**C NMR** (151 MHz, $CDCl_3$) spectrum of compound

84

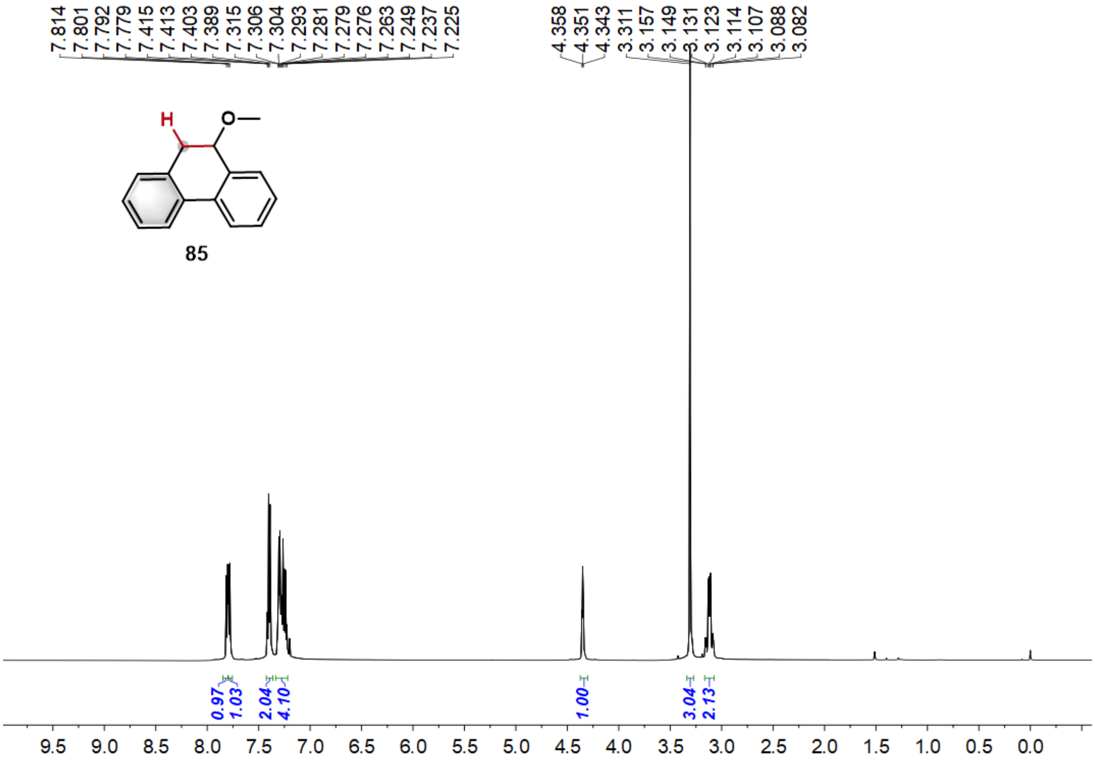

Supplementary Fig. 188 ^{1}**H NMR** (600 MHz, $CDCl_3$) spectrum of compound **85**

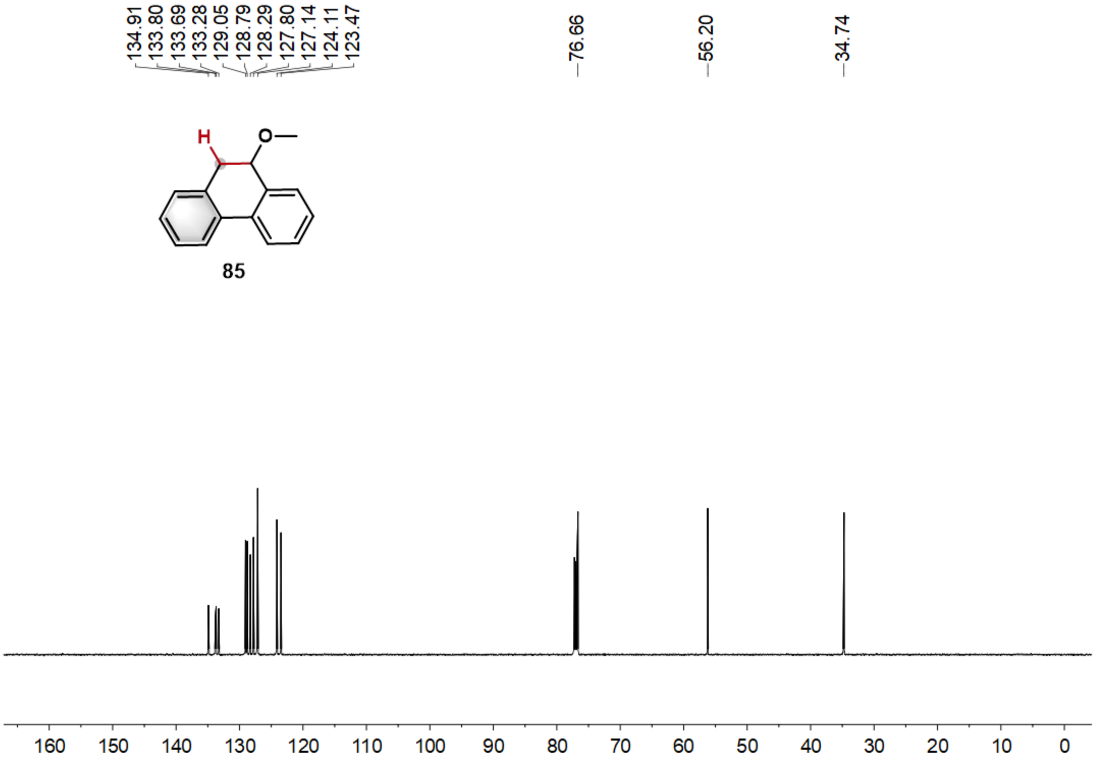

Supplementary Fig. 189 ^{13}**C NMR** (151 MHz, $CDCl_3$) spectrum of compound

85

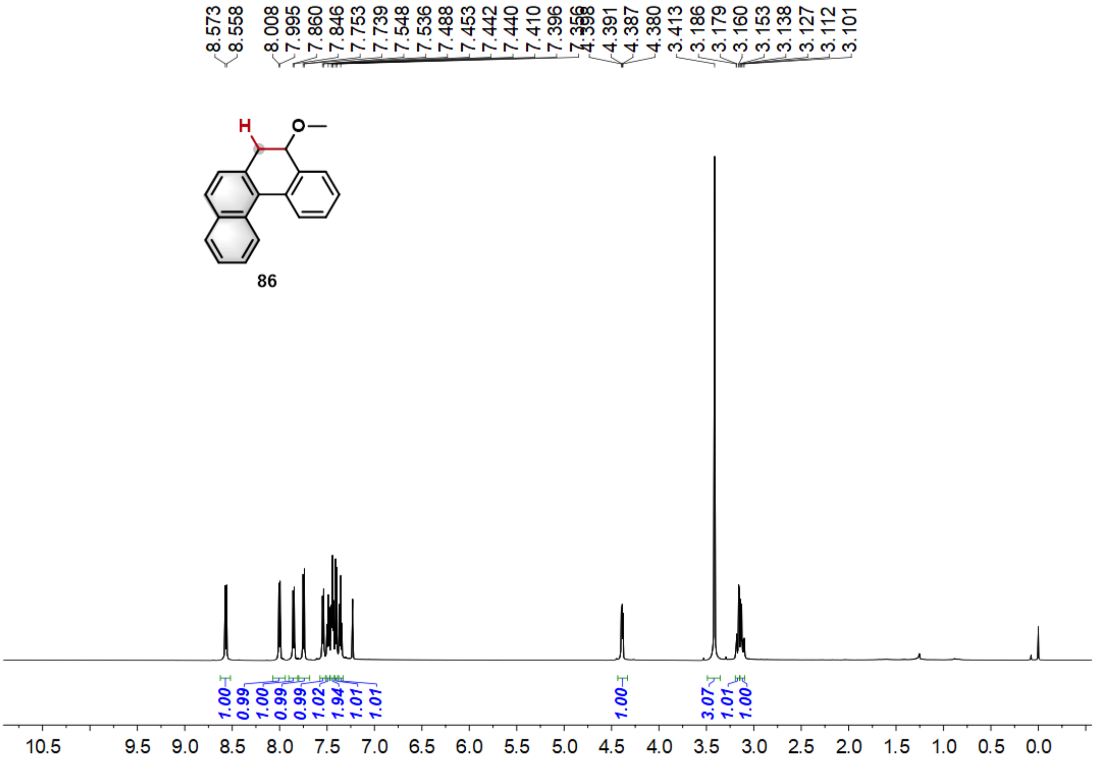

Supplementary Fig. 190 ^{1}H NMR (600 MHz, $CDCl_3$) spectrum of compound **86**

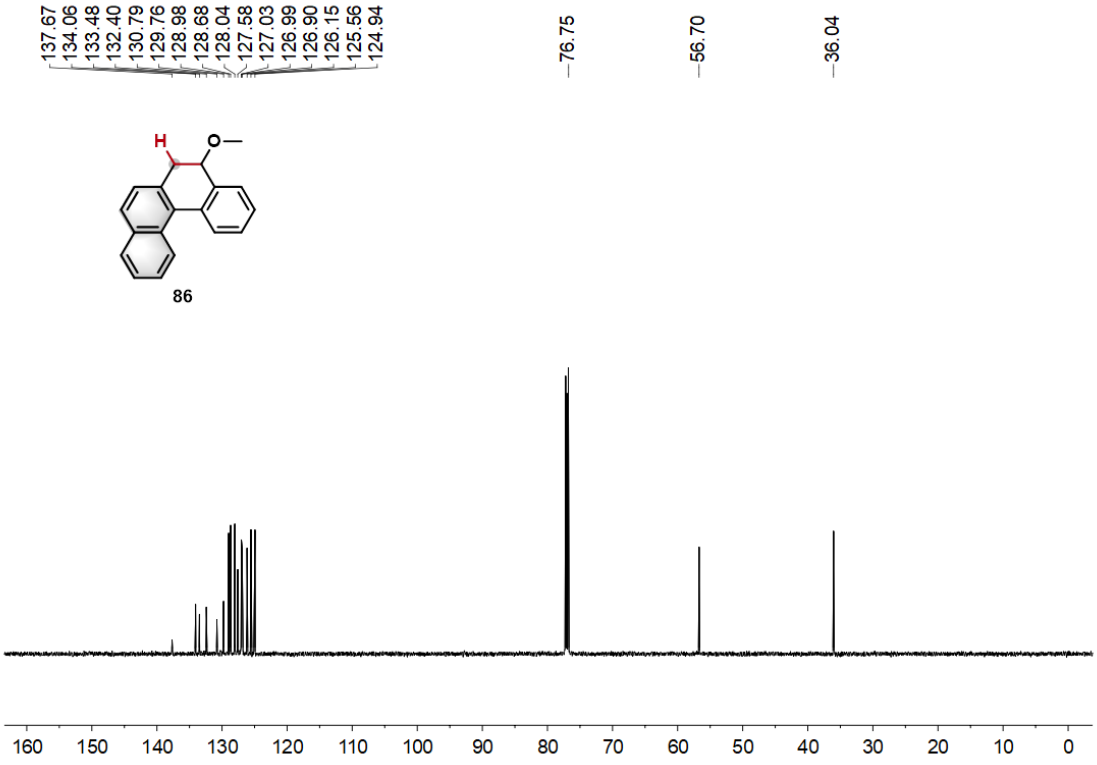

Supplementary Fig. 191 ^{13}C NMR (151 MHz, $CDCl_3$) spectrum of compound

86

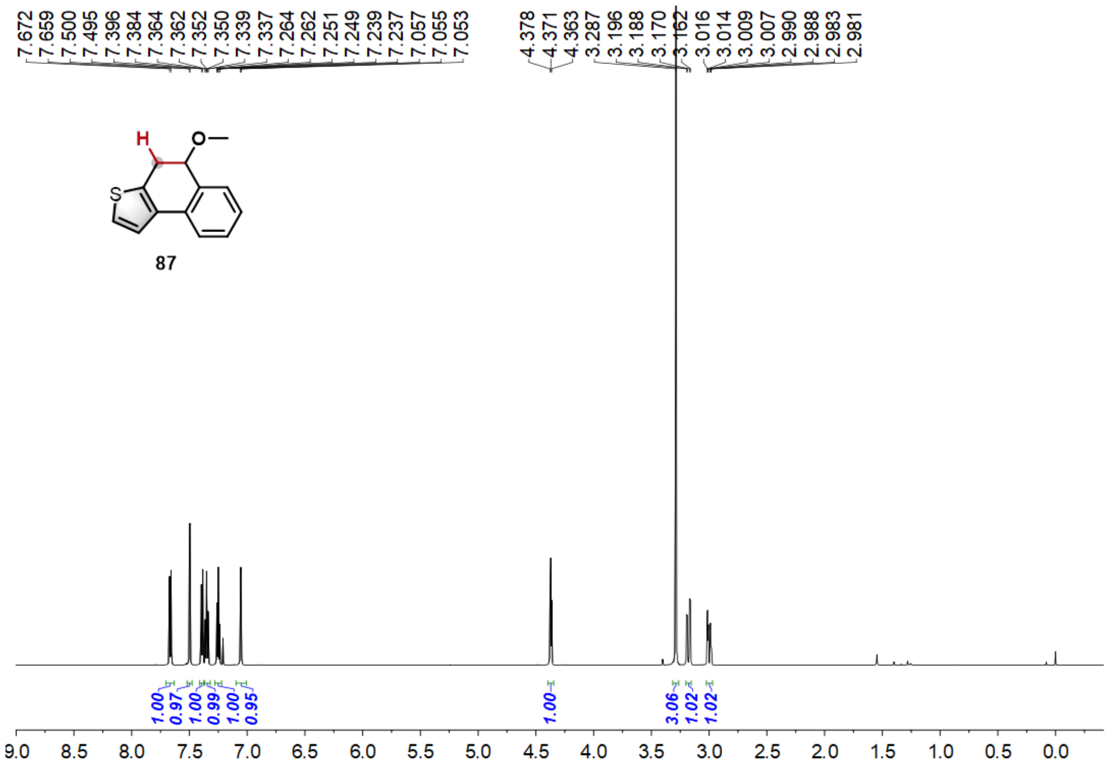

Supplementary Fig. 192 ^{1}H NMR (600 MHz, $CDCl_3$) spectrum of compound **87**

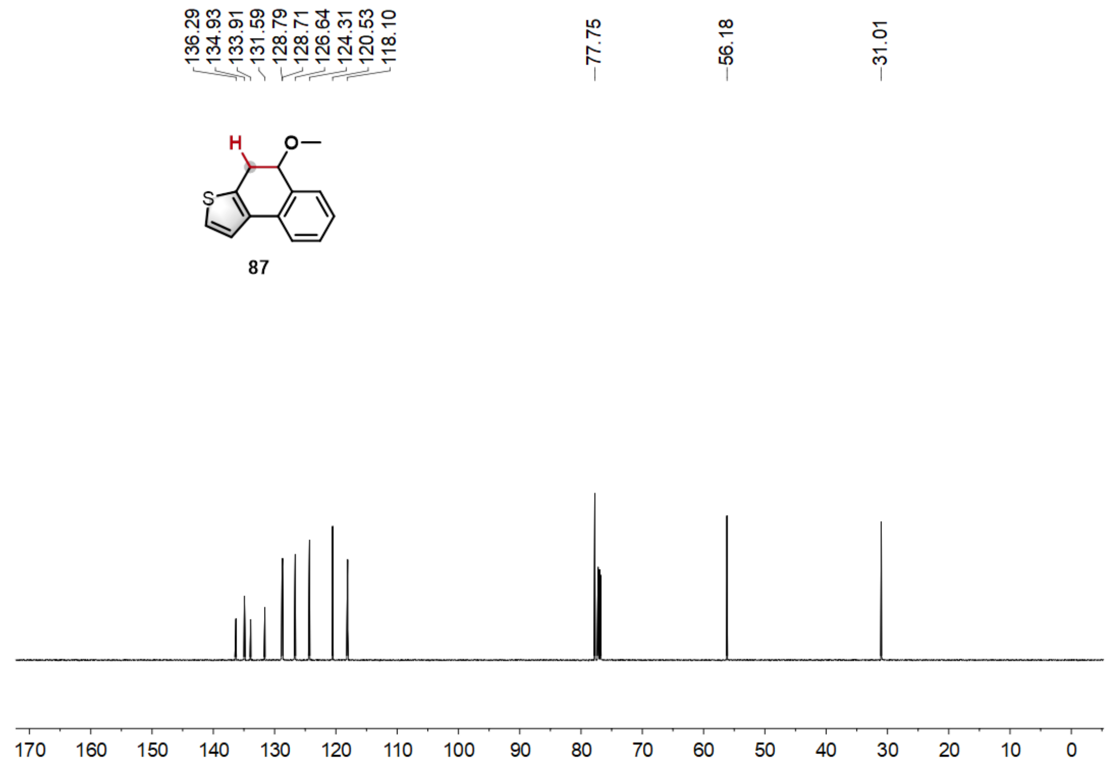

Supplementary Fig. 193 ^{13}C NMR (151 MHz, $CDCl_3$) spectrum of compound

87

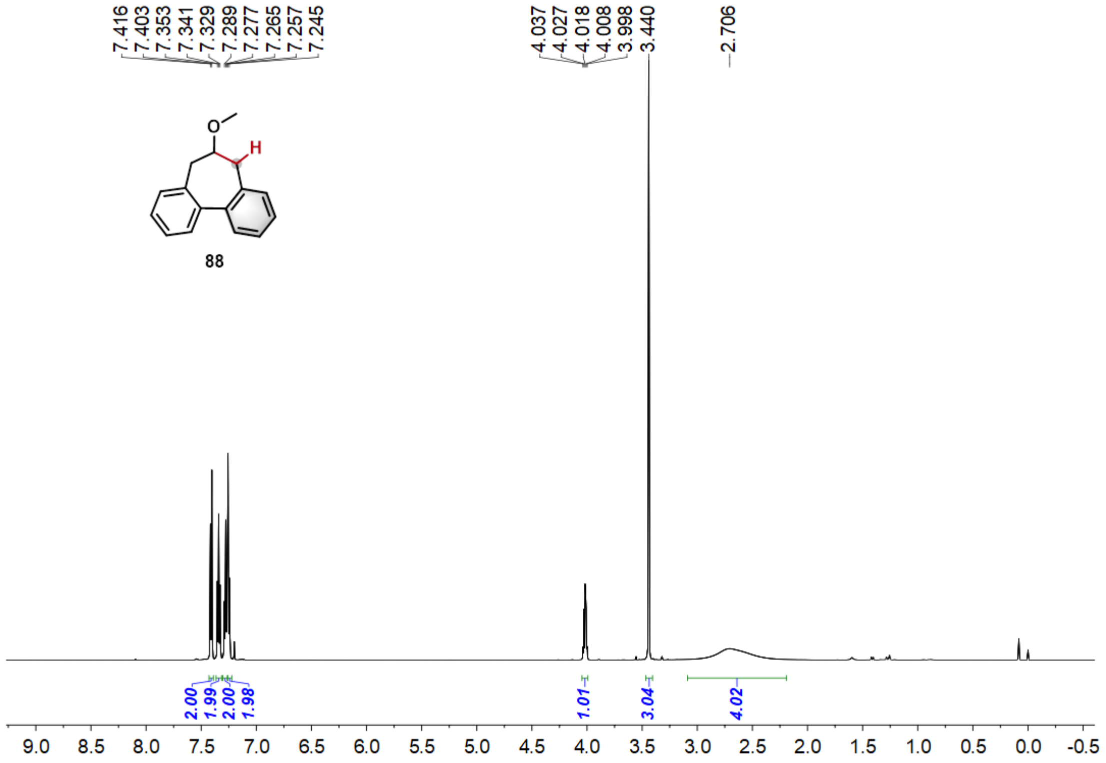

Supplementary Fig. 194 ^{1}H NMR (600 MHz, $CDCl_3$) spectrum of compound **88**

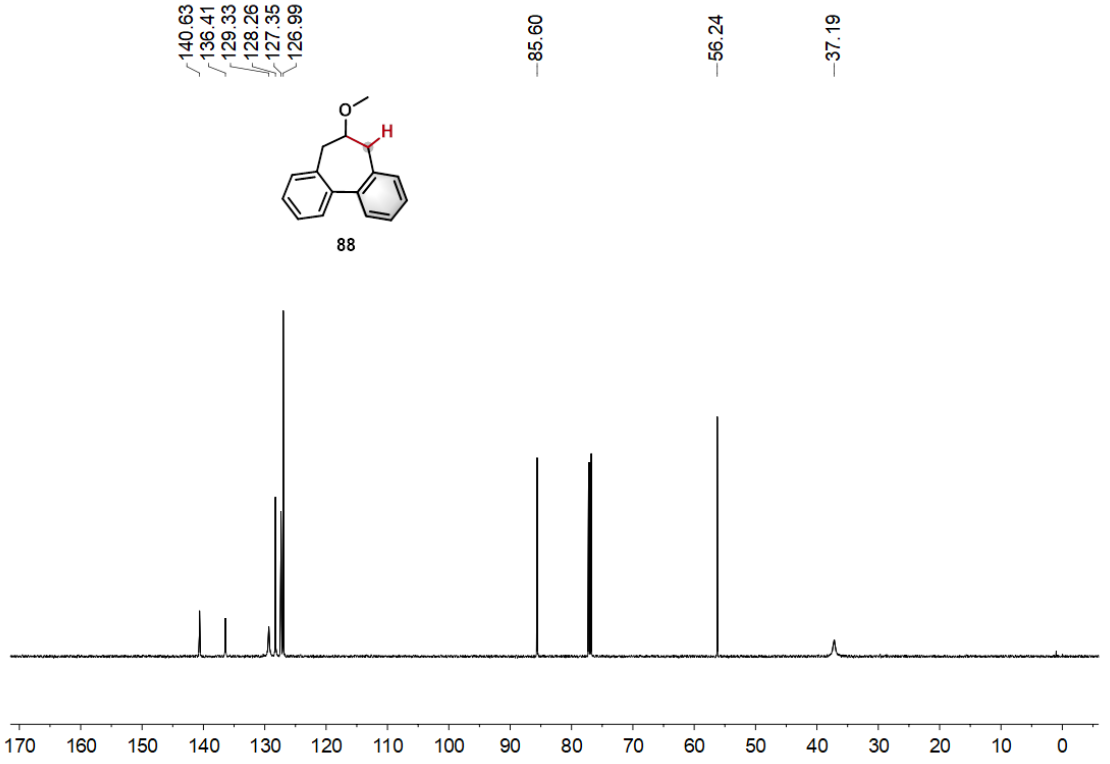

Supplementary Fig. 195 ^{13}C NMR (151 MHz, $CDCl_3$) spectrum of compound

88

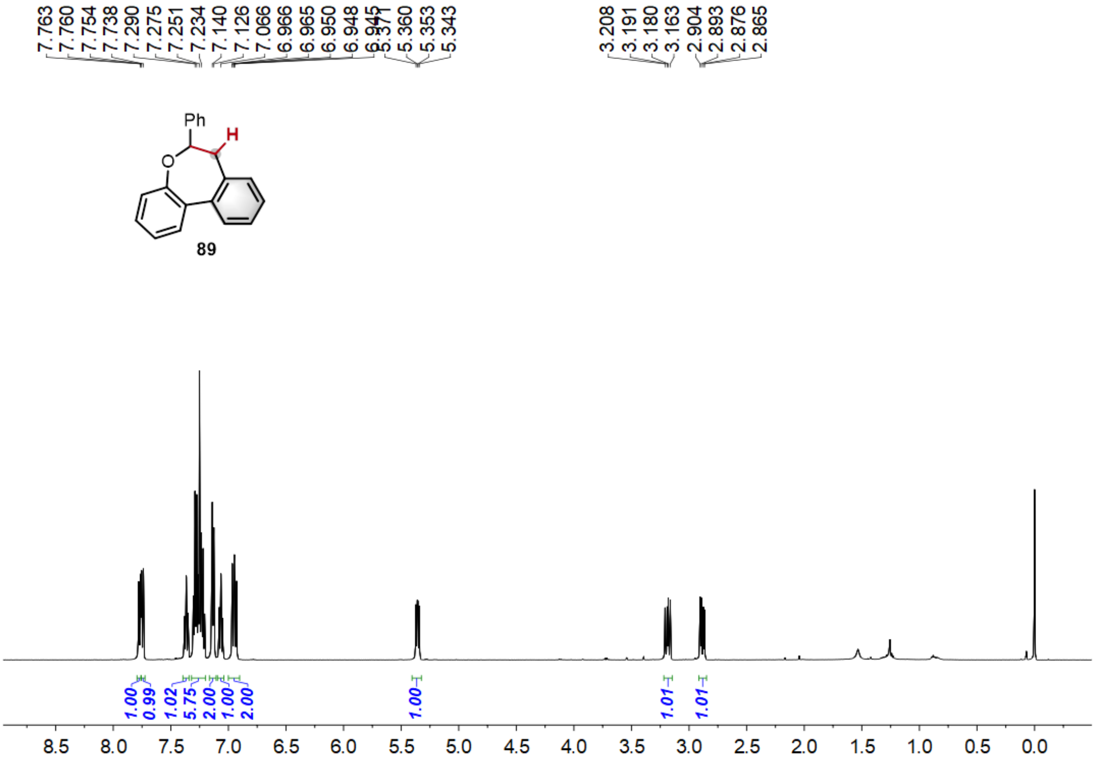

Supplementary Fig. 196 ^{1}H NMR (500 MHz, $CDCl_3$) spectrum of compound **89**

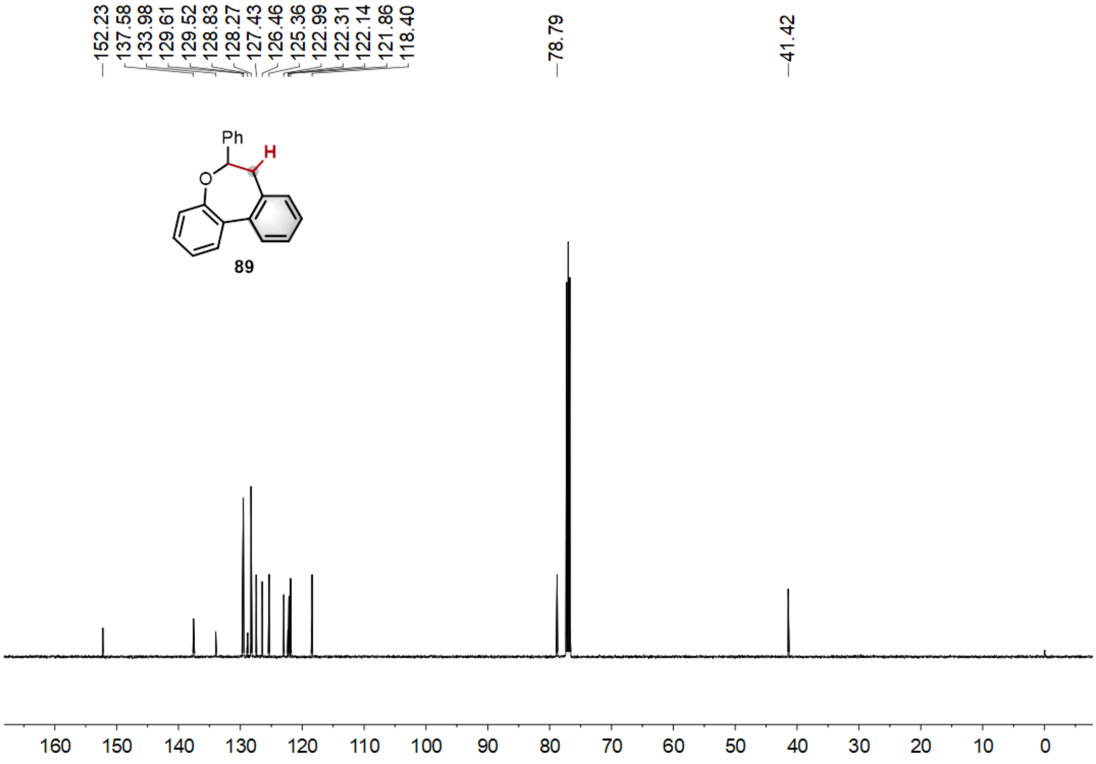

Supplementary Fig. 197 ^{13}C NMR (151 MHz, $CDCl_3$) spectrum of compound

89

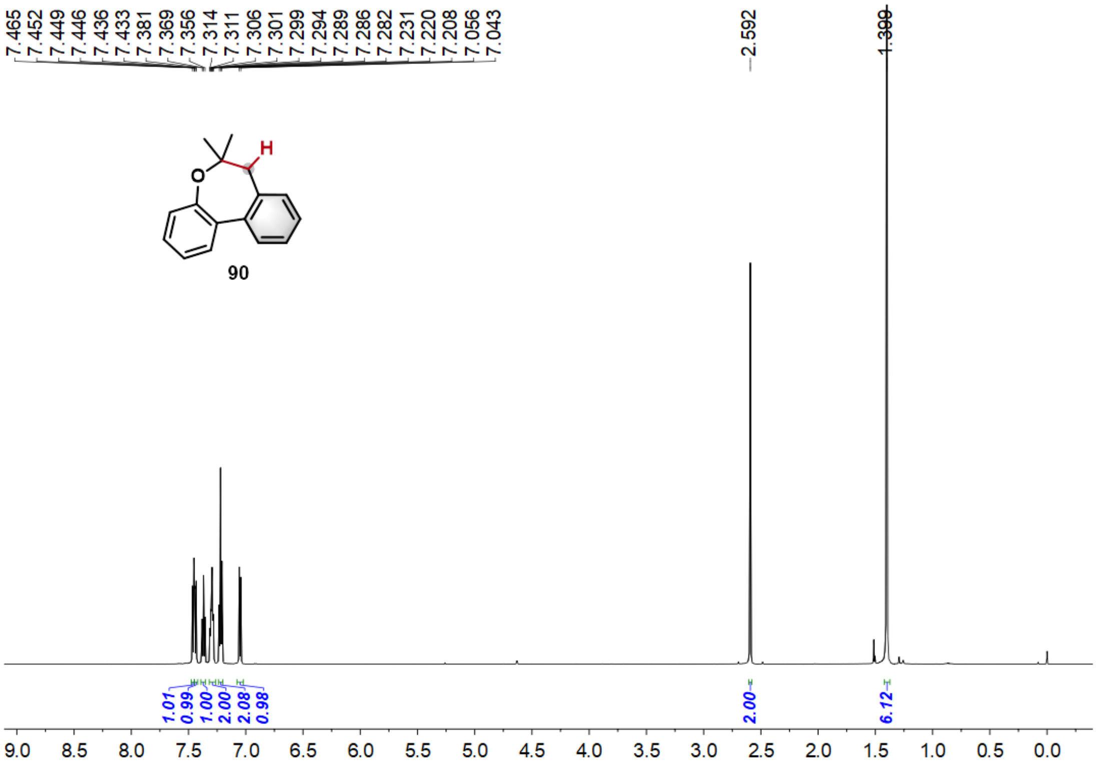

Supplementary Fig. 198 ^{1}H NMR (600 MHz, $CDCl_3$) spectrum of compound **90**

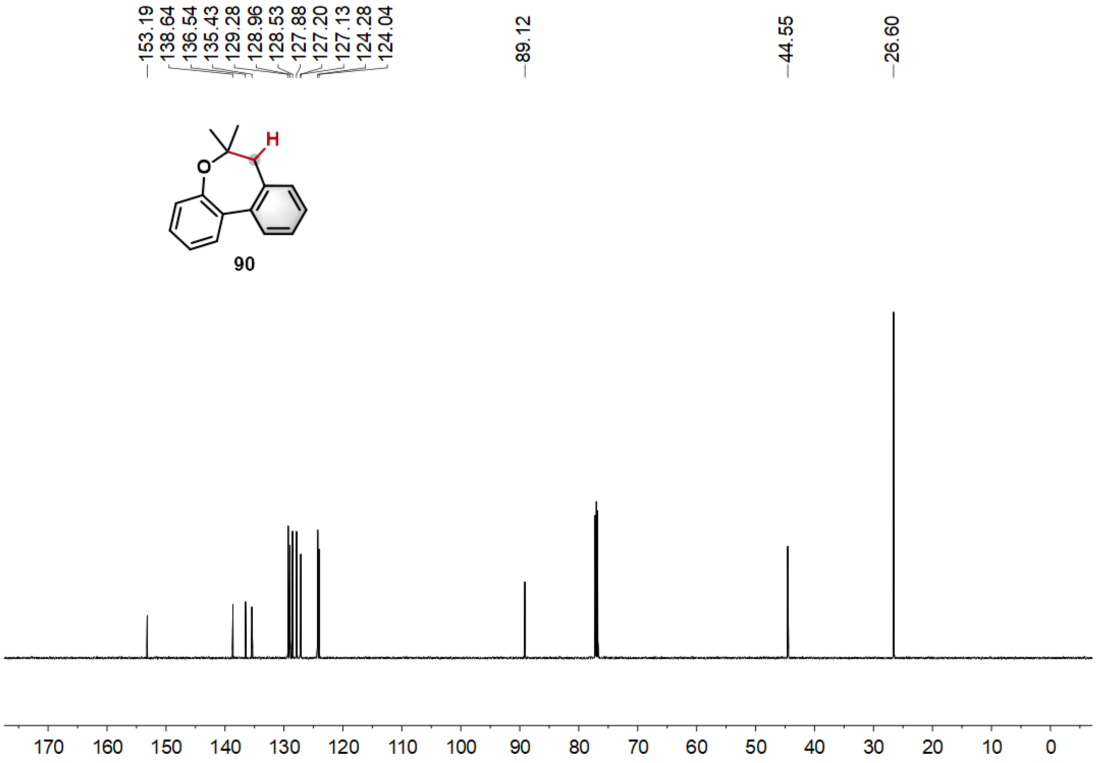

Supplementary Fig. 199 ^{13}C NMR (151 MHz, $CDCl_3$) spectrum of compound

90

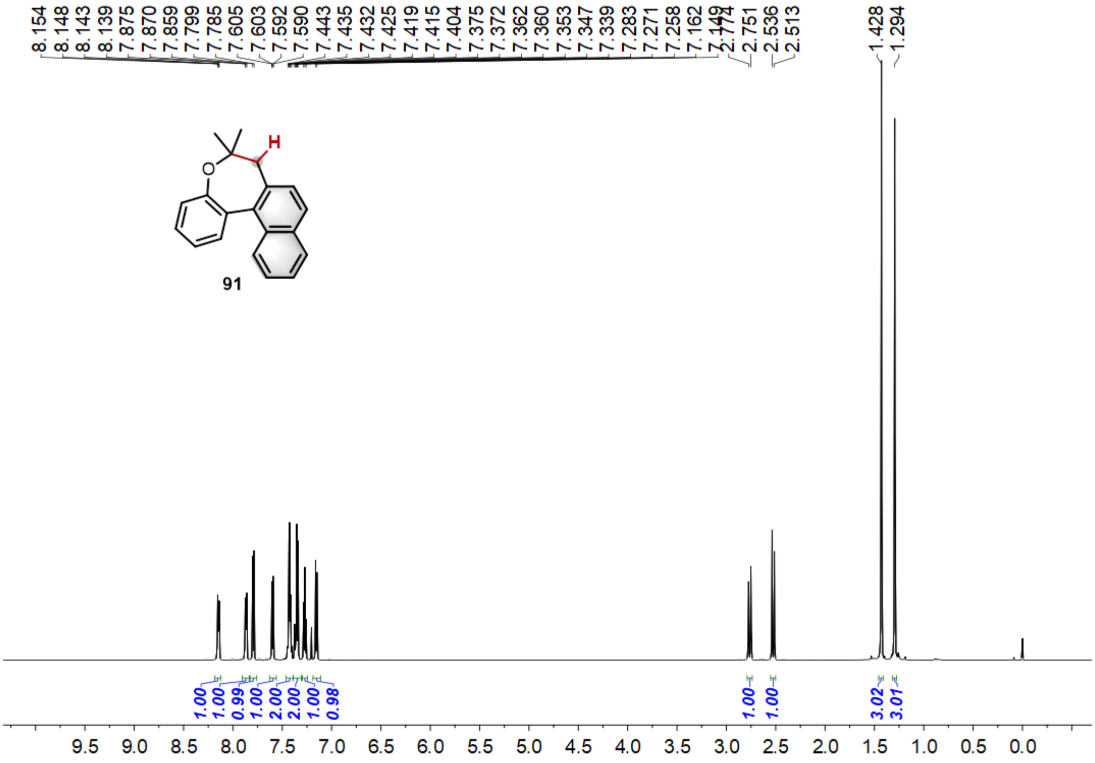

Supplementary Fig. 200 ^{1}H NMR (600 MHz, $CDCl_3$) spectrum of compound **91**

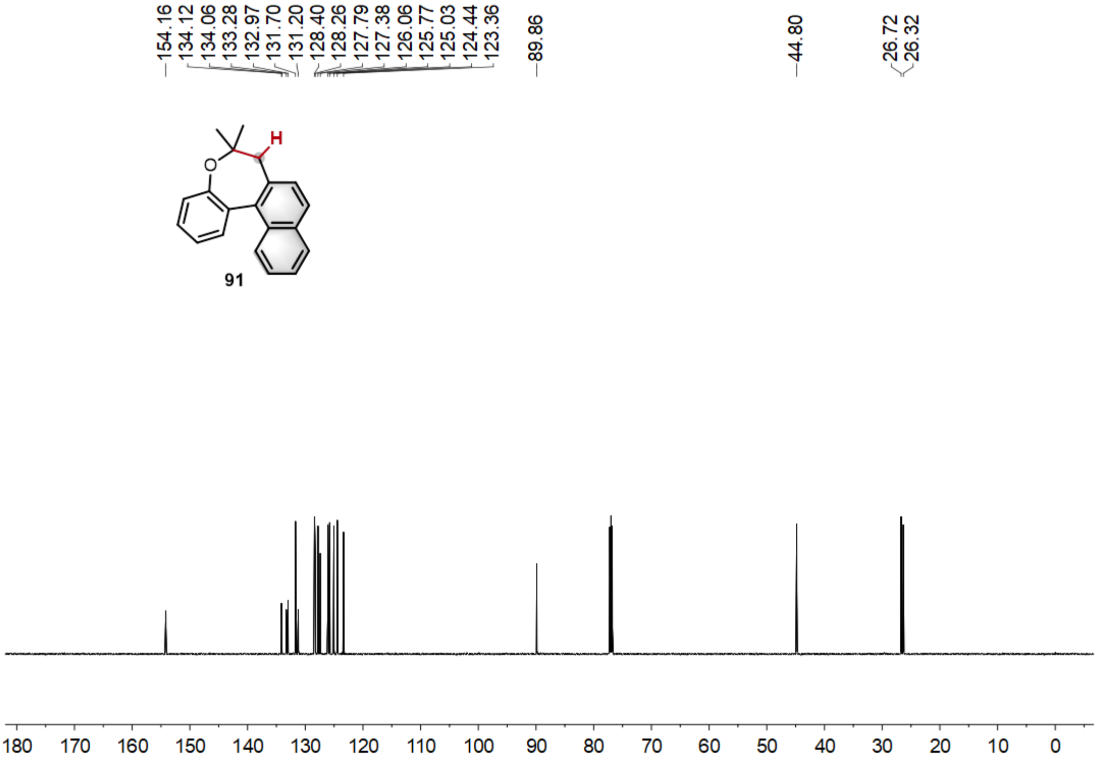

Supplementary Fig. 201 ^{13}C NMR (151 MHz, $CDCl_3$) spectrum of compound

91

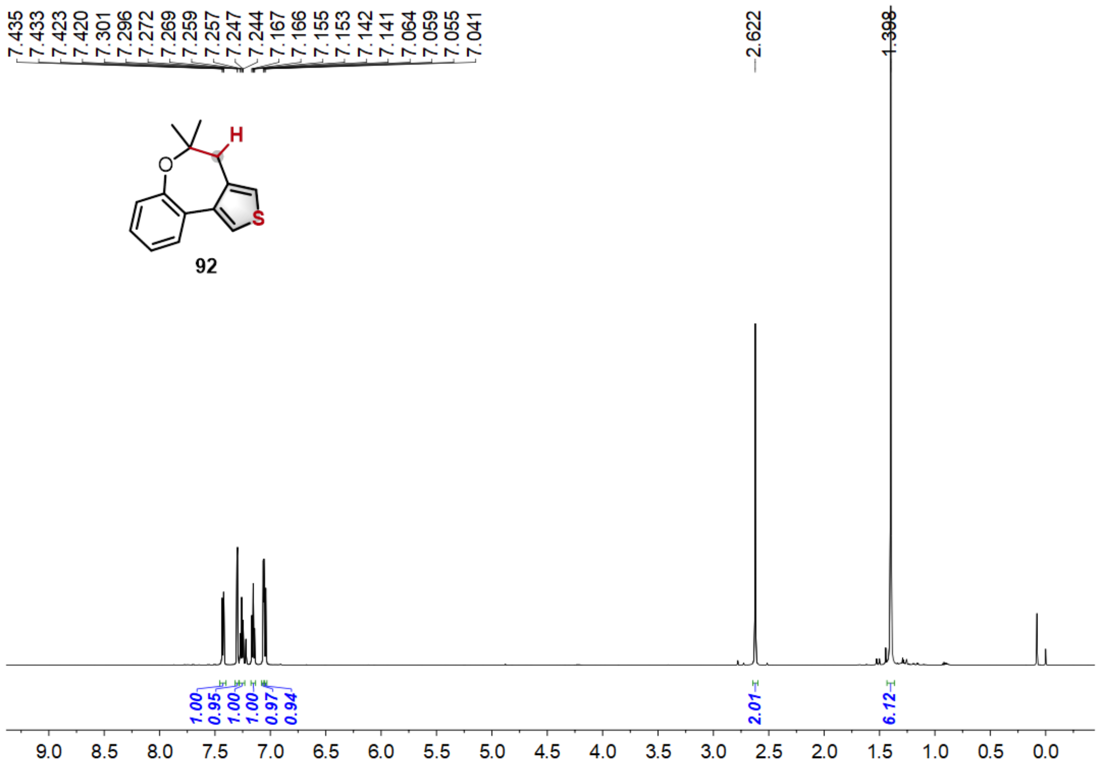

Supplementary Fig. 202 ^{1}H NMR (600 MHz, $CDCl_3$) spectrum of compound **92**

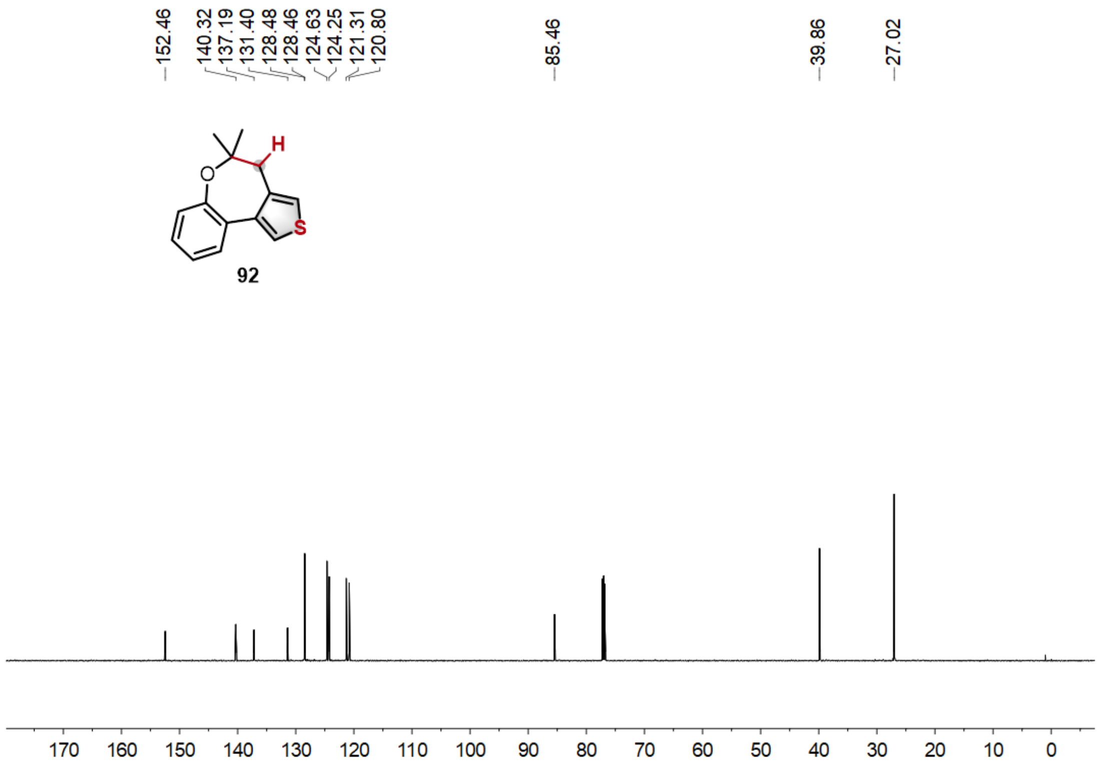

Supplementary Fig. 203 ^{13}C NMR (151 MHz, $CDCl_3$) spectrum of compound **92**

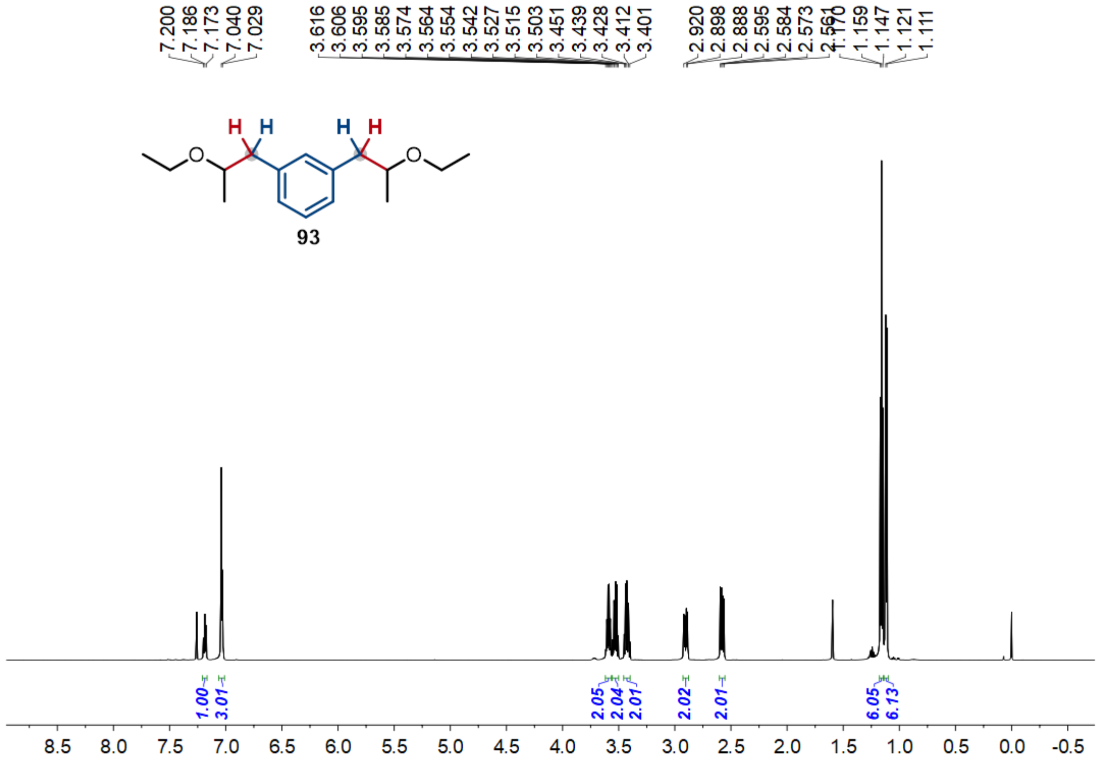

Supplementary Fig. 204 ^{1}H NMR (600 MHz, $CDCl_3$) spectrum of compound **93**

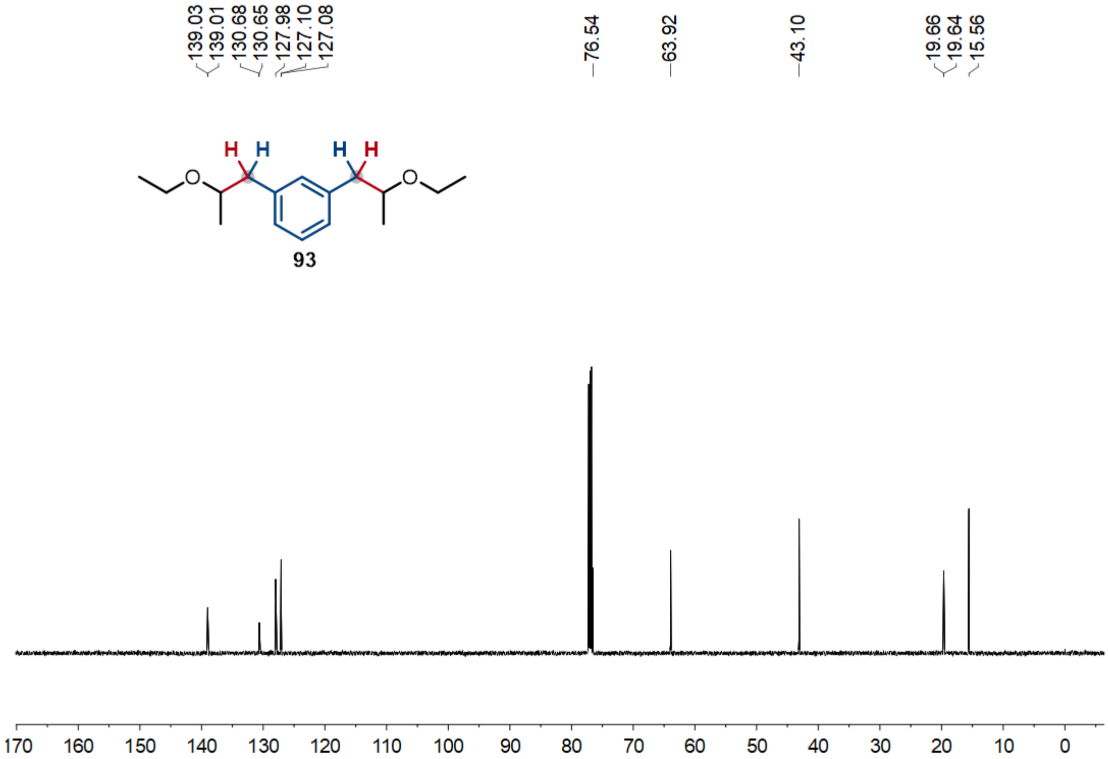

Supplementary Fig. 205 ^{13}C NMR (151 MHz, $CDCl_3$) spectrum of compound

93

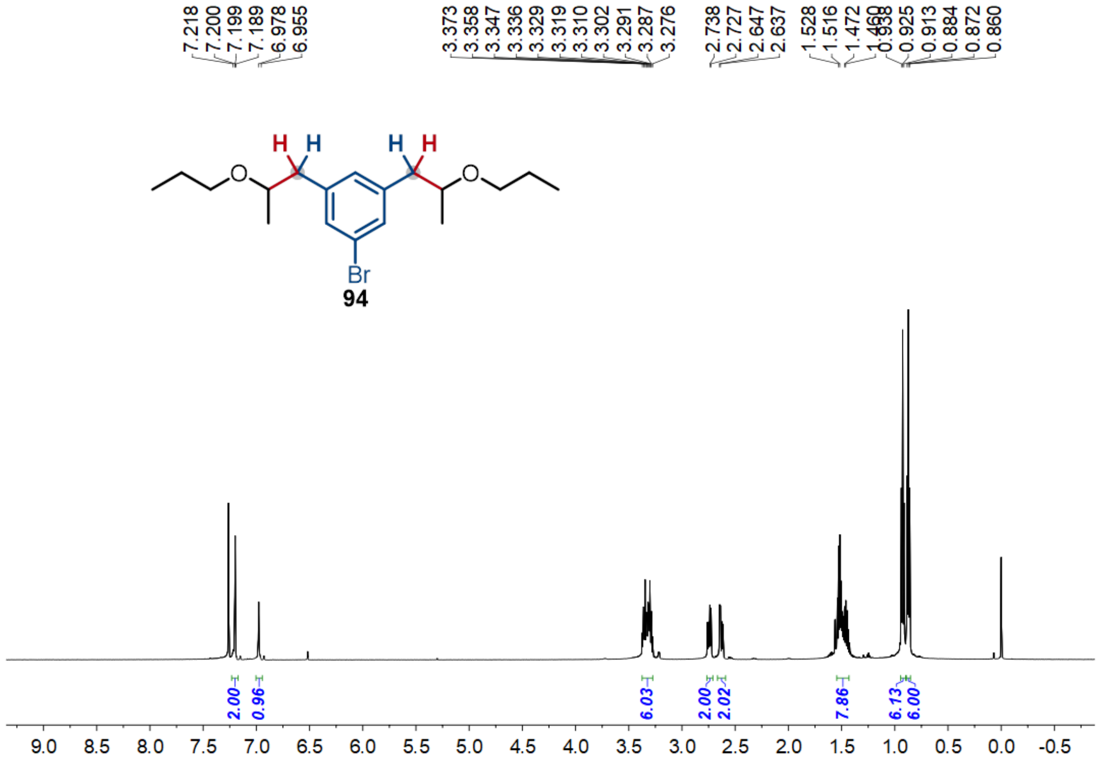

Supplementary Fig. 206 ^{1}H NMR (600 MHz, $CDCl_3$) spectrum of compound **94**

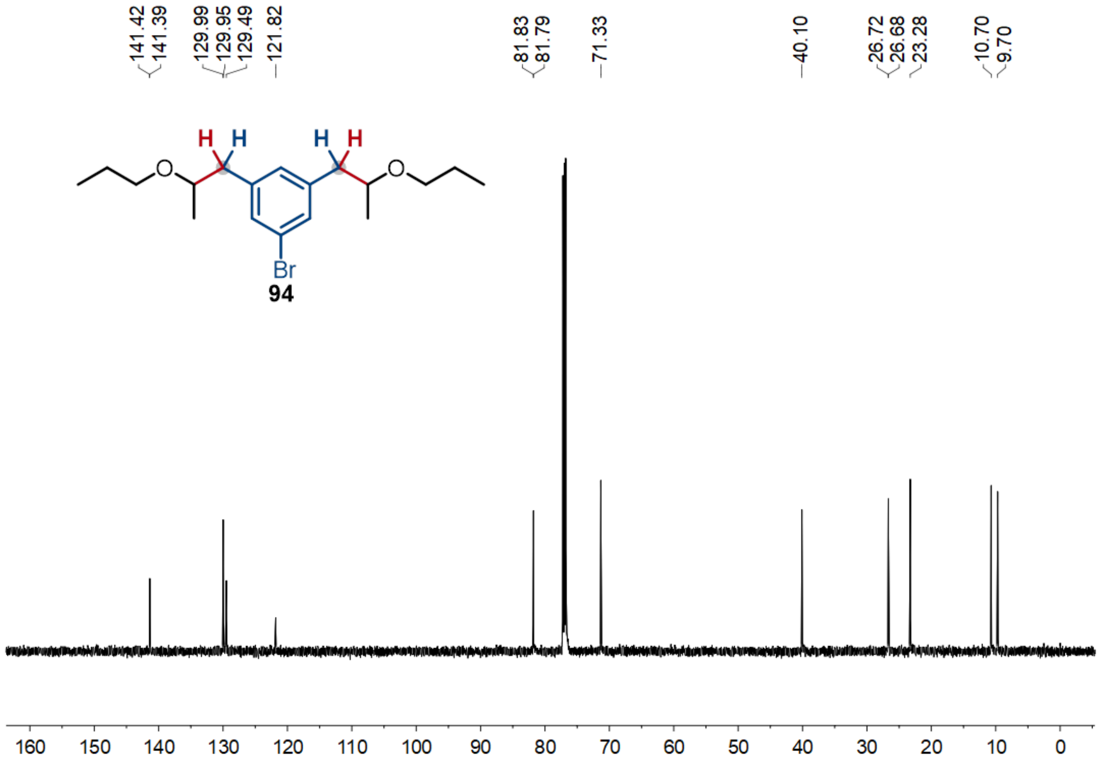

Supplementary Fig. 207 ^{13}C NMR (151 MHz, $CDCl_3$) spectrum of compound

94

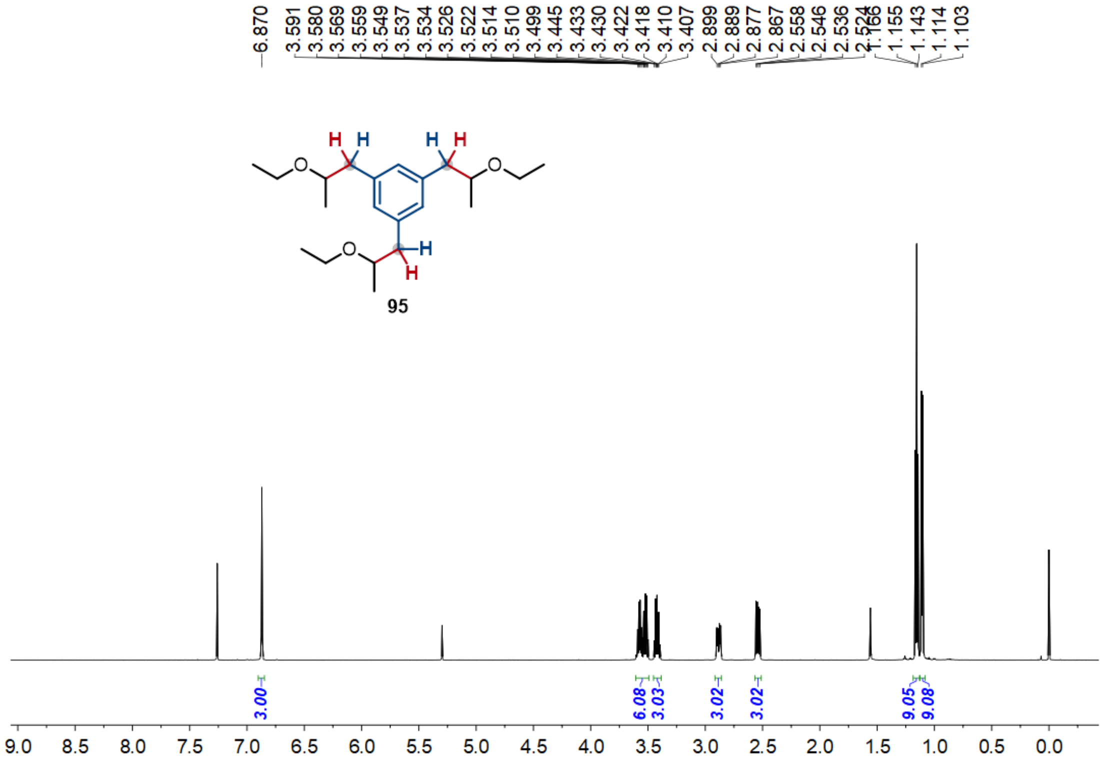

Supplementary Fig. 208 ^{1}H NMR (600 MHz, $CDCl_3$) spectrum of compound **95**

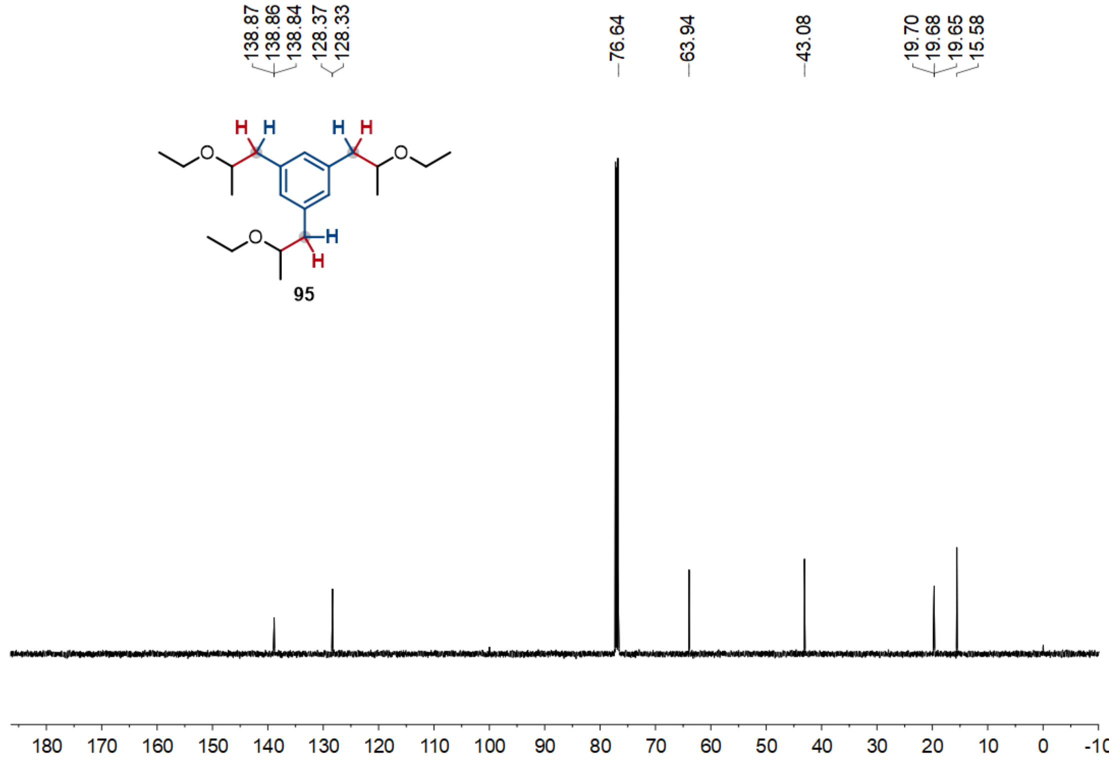

Supplementary Fig. 209 ^{13}C NMR (151 MHz, $CDCl_3$) spectrum of compound

95

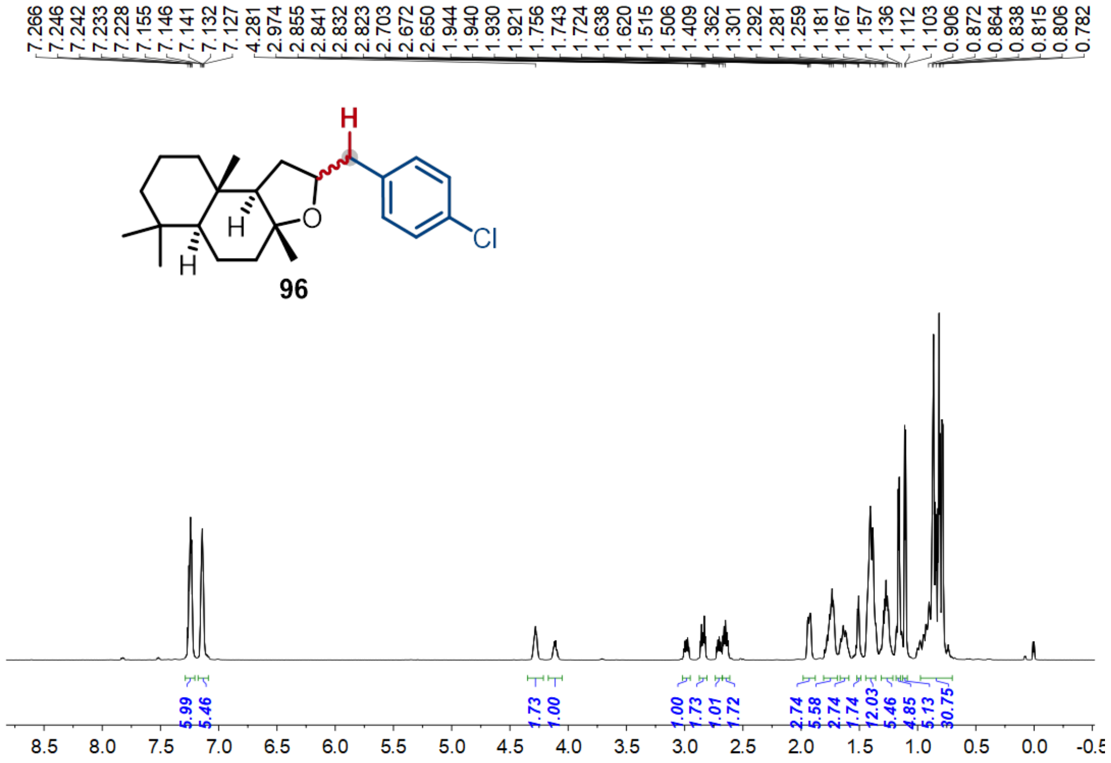

Supplementary Fig. 210 ^{1}H NMR (600 MHz, $CDCl_3$) spectrum of compound **96**

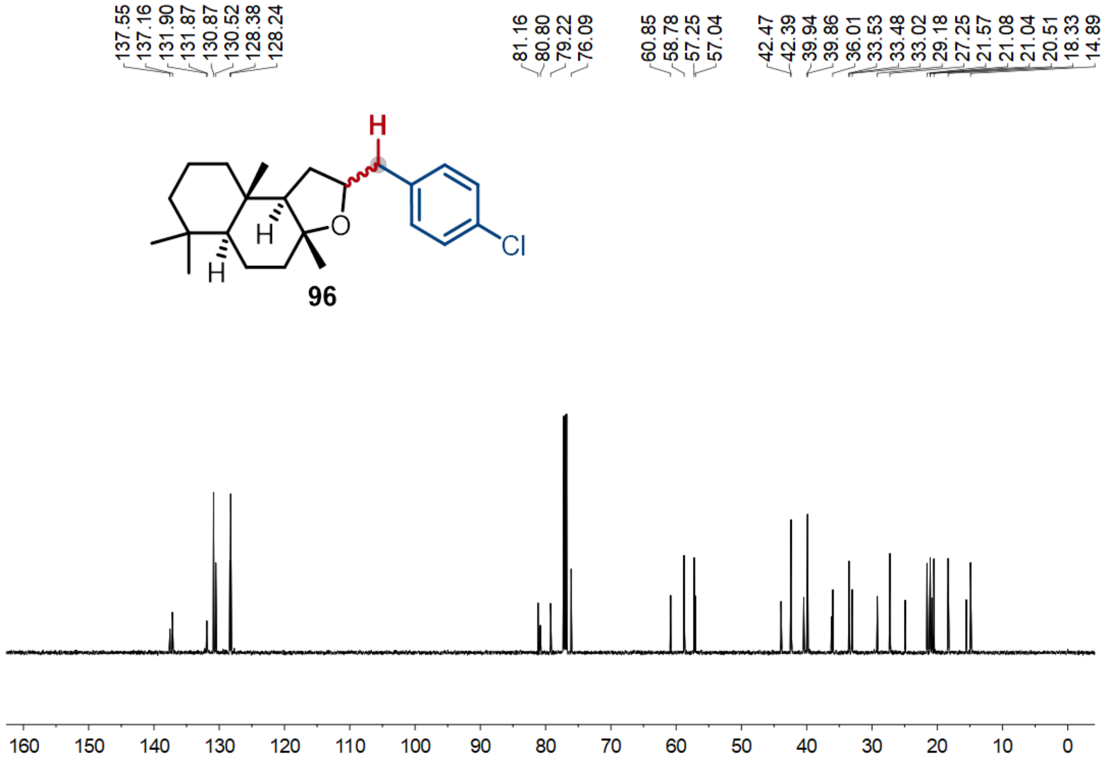

Supplementary Fig. 211 ^{13}**C NMR** (151 MHz, $CDCl_3$) spectrum of compound

96

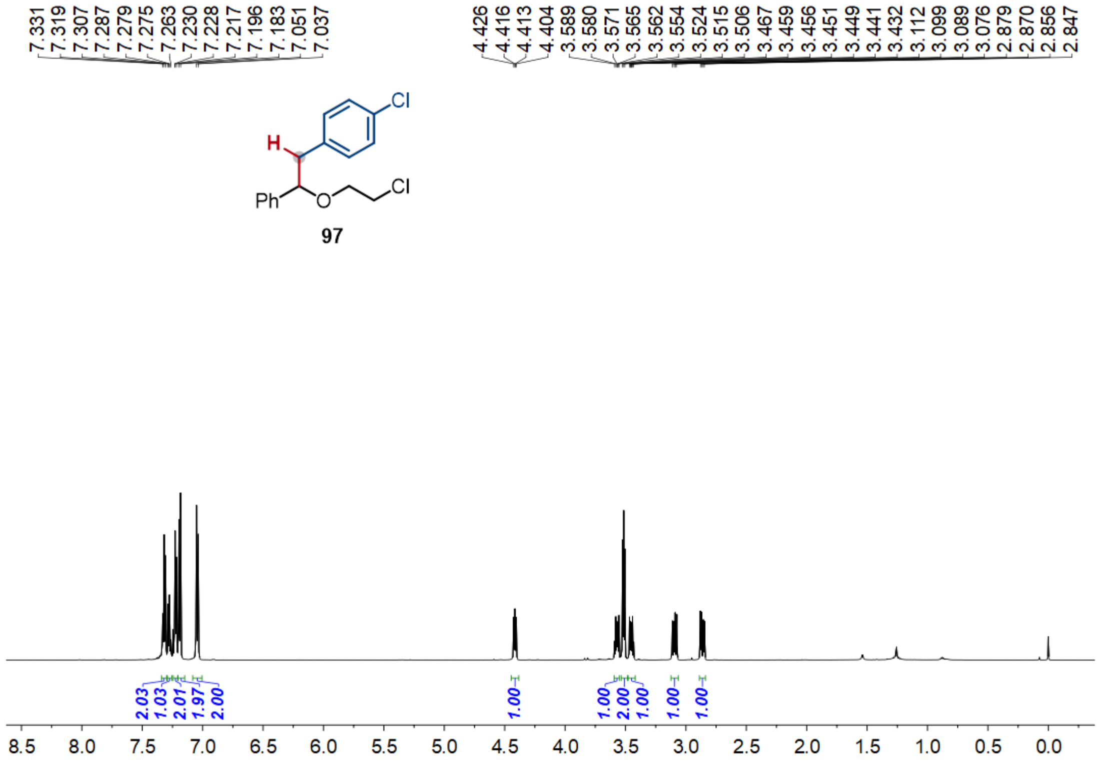

Supplementary Fig. 212 ^{1}H NMR (600 MHz, $CDCl_3$) spectrum of compound **97**

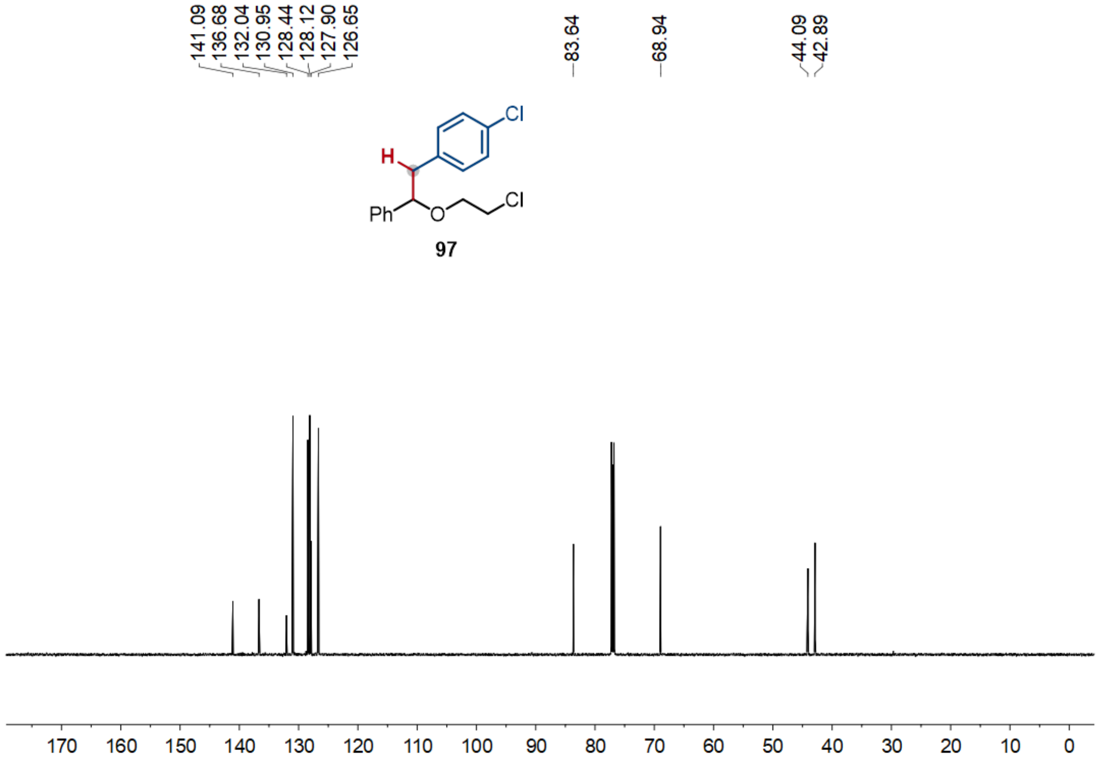

Supplementary Fig. 213 ^{13}C NMR (151 MHz, $CDCl_3$) spectrum of compound

97

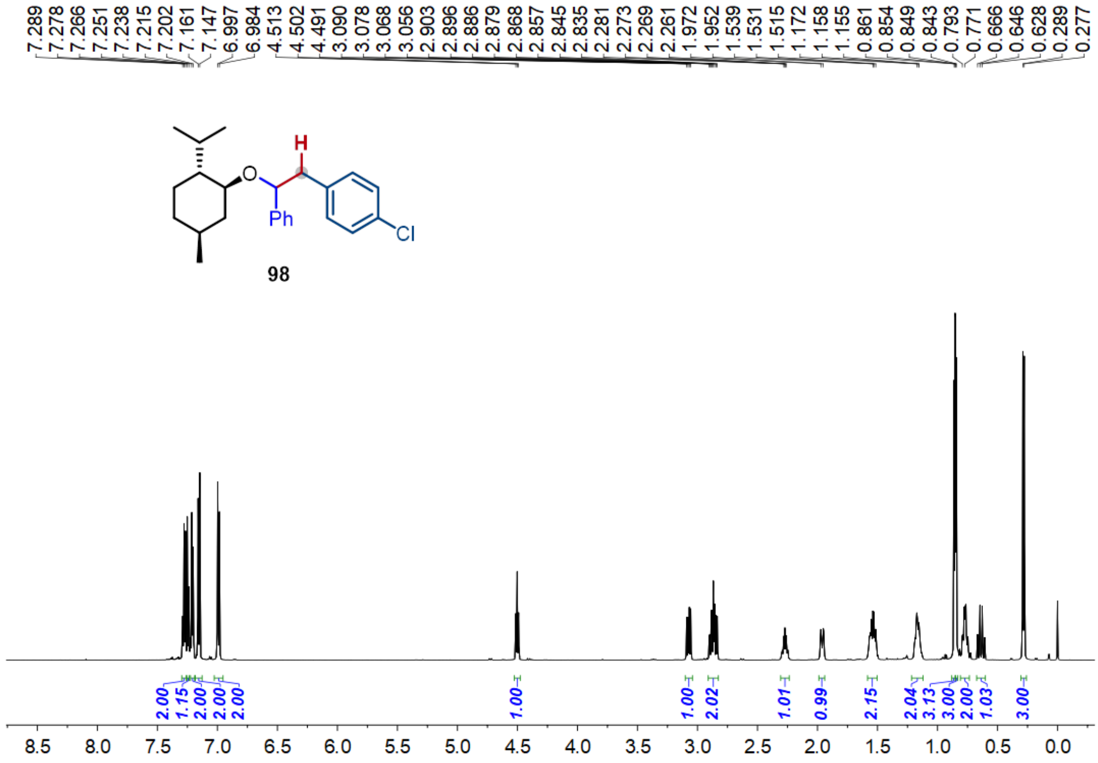

Supplementary Fig. 214 ^{1}H NMR (600 MHz, $CDCl_3$) spectrum of compound **98**

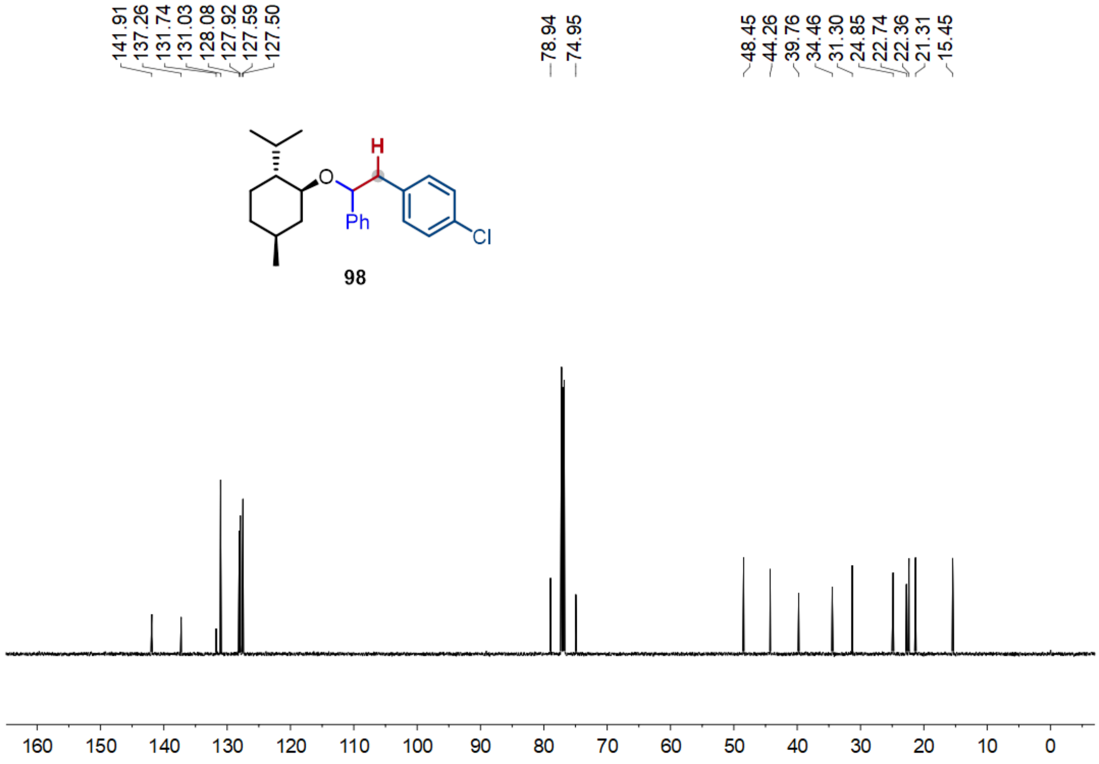

Supplementary Fig. 215 ^{13}C NMR (151 MHz, $CDCl_3$) spectrum of compound

98

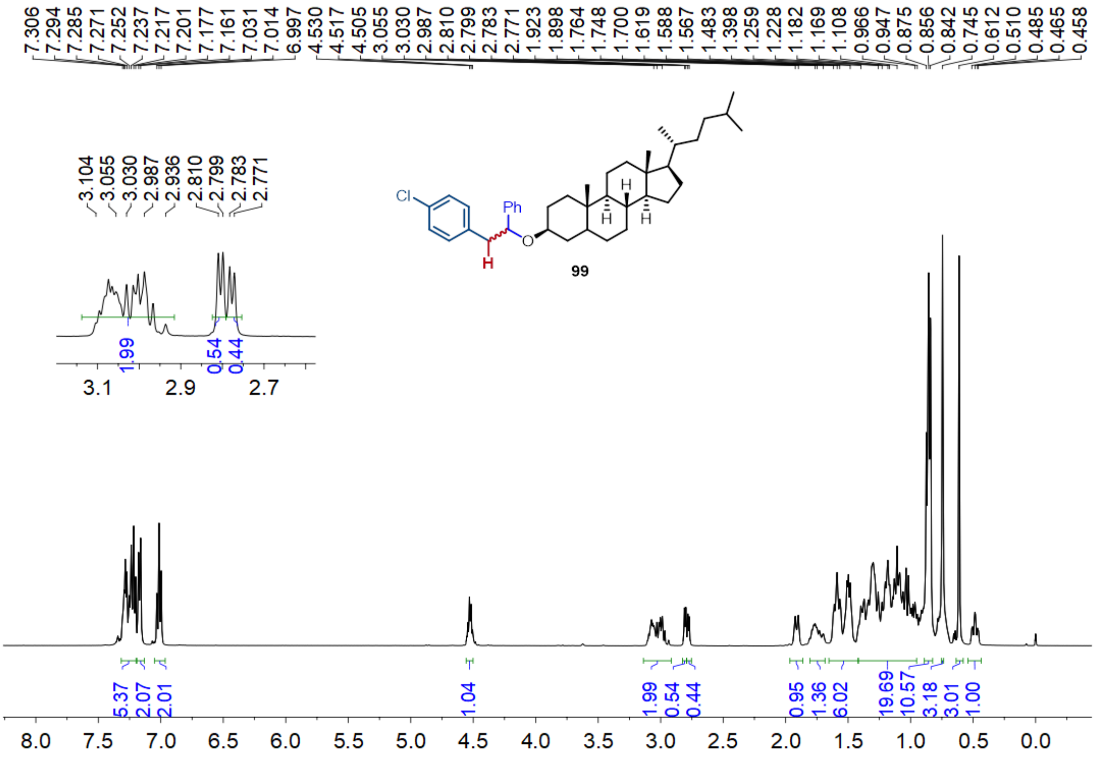

Supplementary Fig. 216 **^{1}H NMR** (500 MHz, $CDCl_3$) spectrum of compound **99**

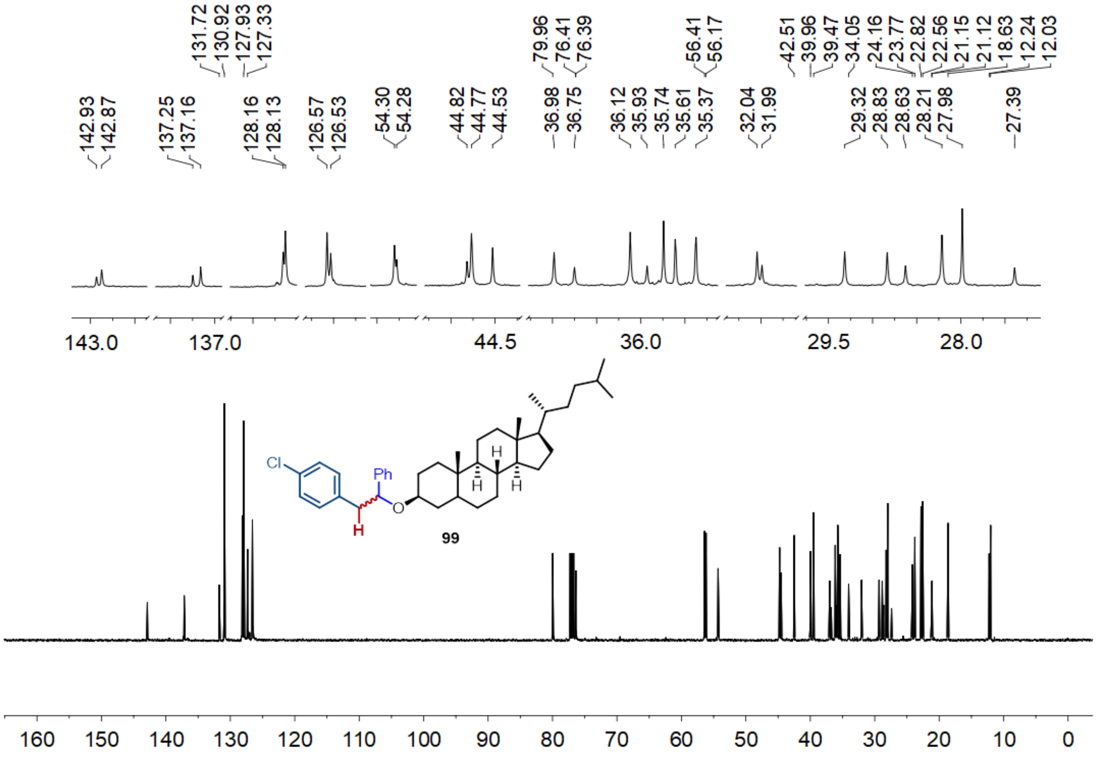

Supplementary Fig. 217 ^{13}C NMR (126 MHz, $CDCl_3$) spectrum of compound

99

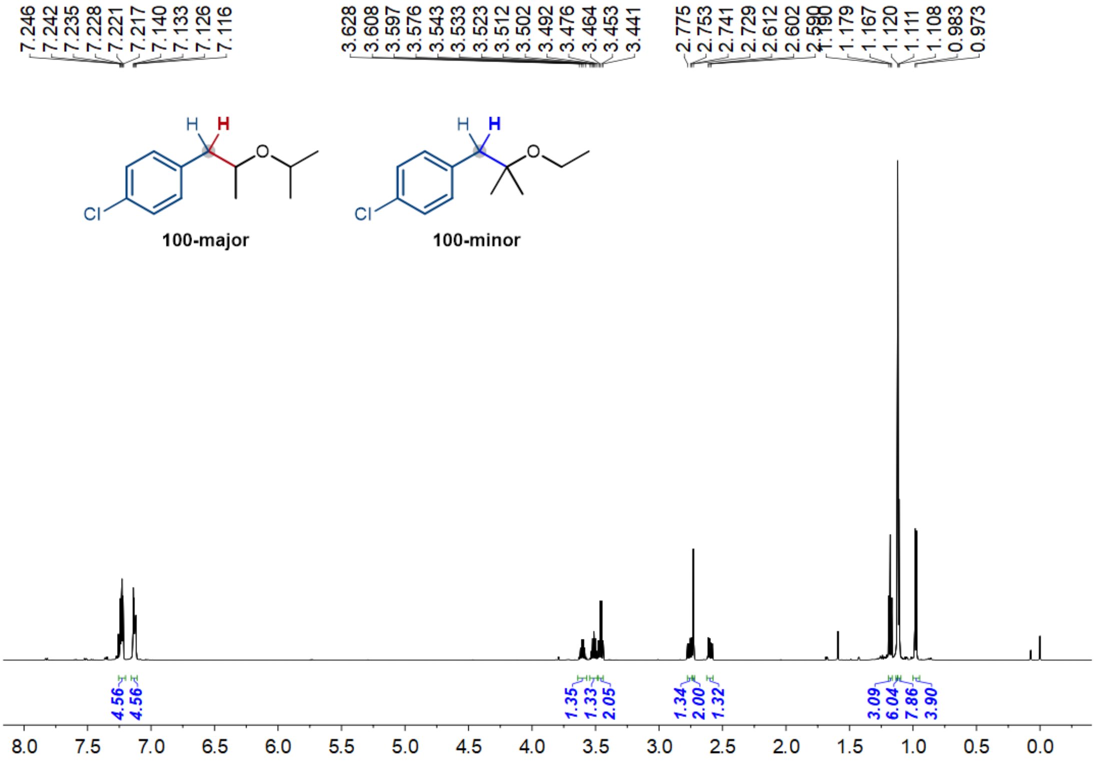

Supplementary Fig. 218 ^{1}**H NMR** (600 MHz, $CDCl_3$) spectrum of compound **100**

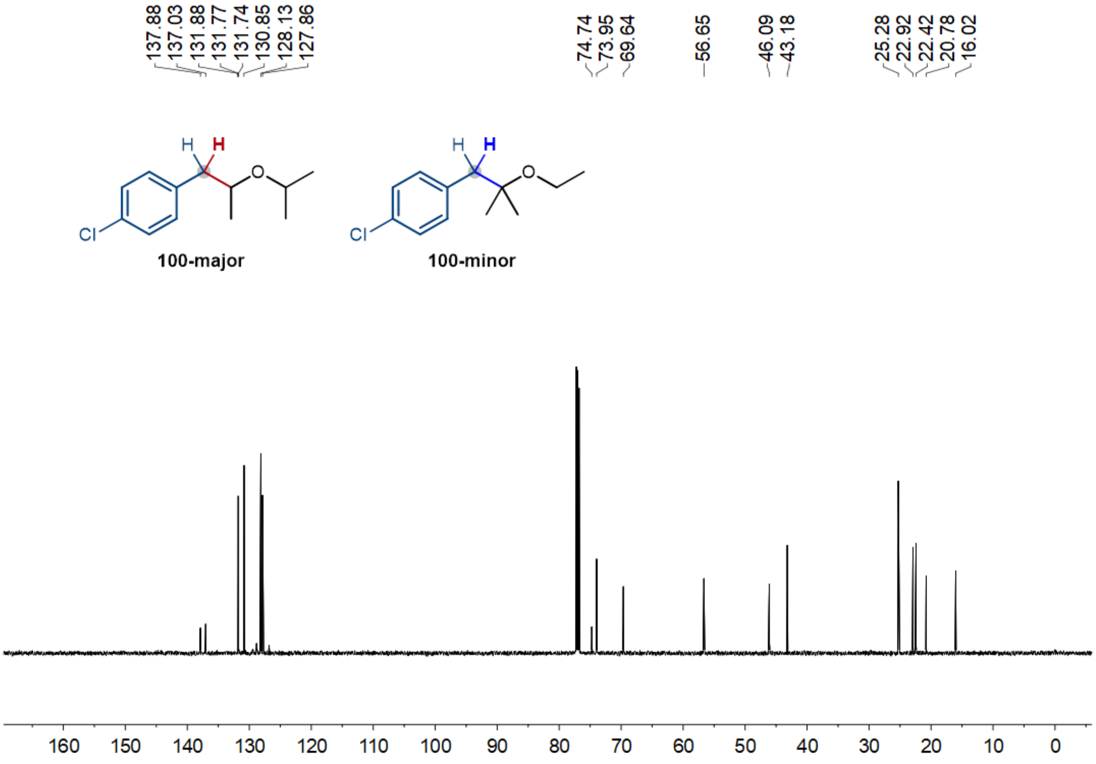

Supplementary Fig. 219 ^{13}**C NMR** (151 MHz, $CDCl_3$) spectrum of compound

100

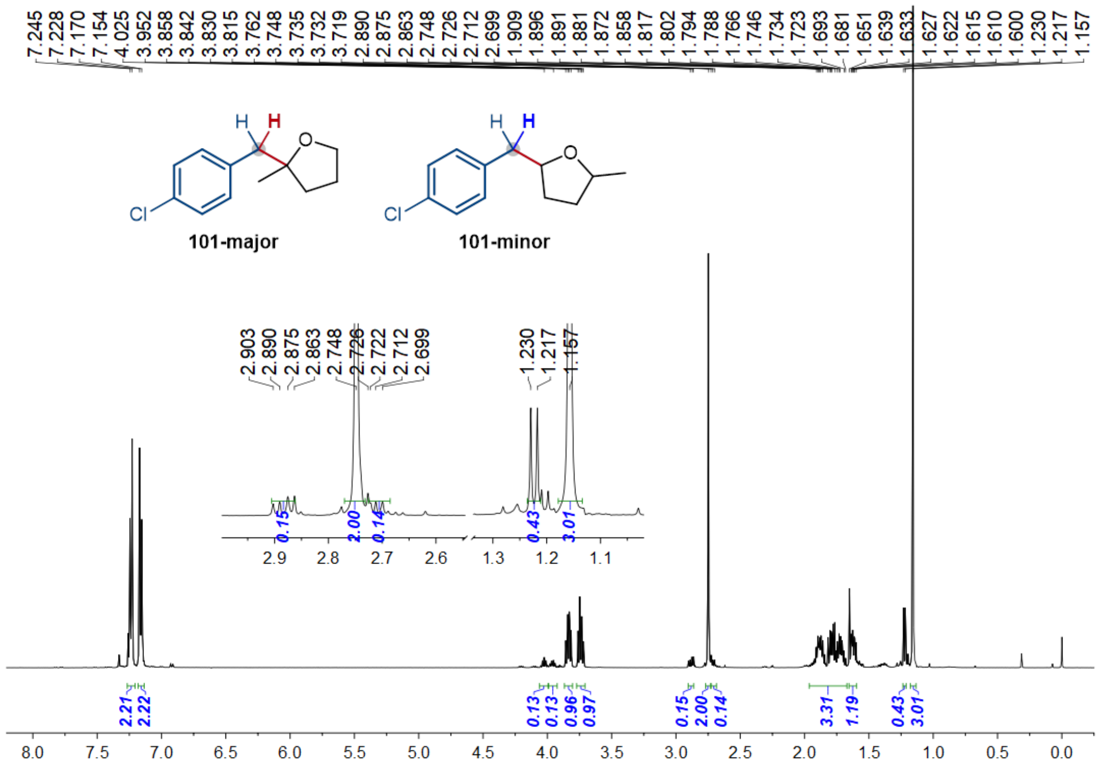

Supplementary Fig. 220 ^{1}H NMR (500 MHz, $CDCl_3$) spectrum of compound **101**

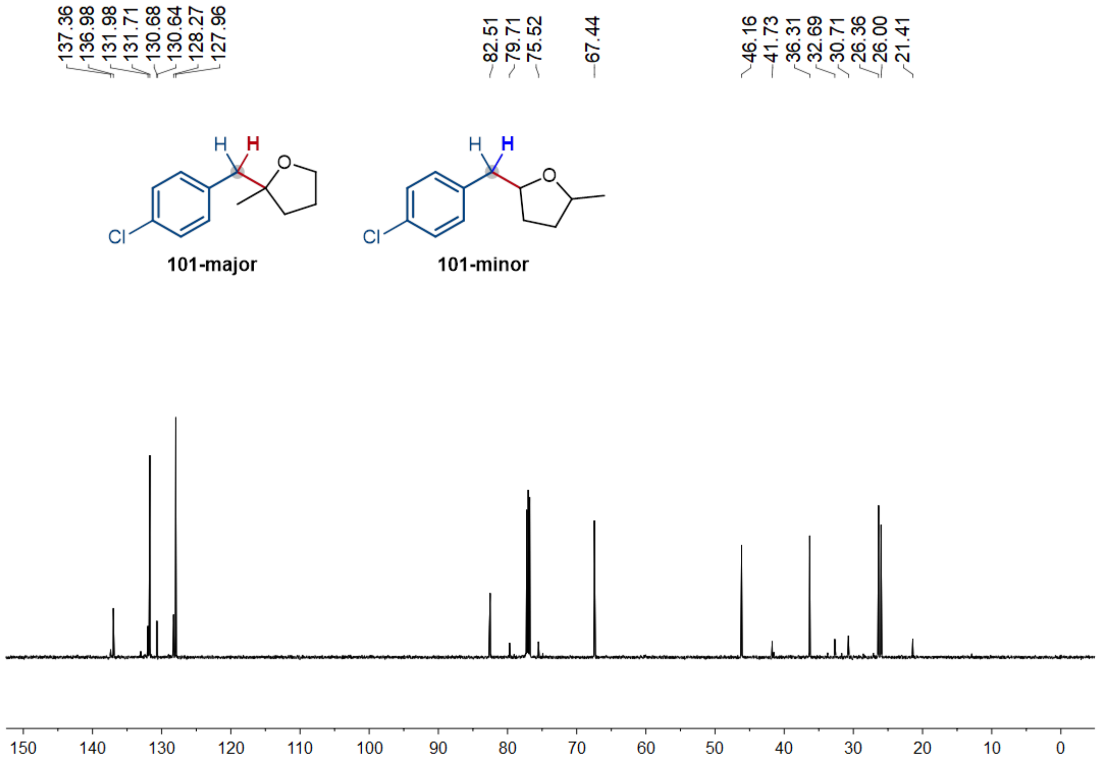

Supplementary Fig. 221 ^{13}C NMR (151 MHz, $CDCl_3$) spectrum of compound

101

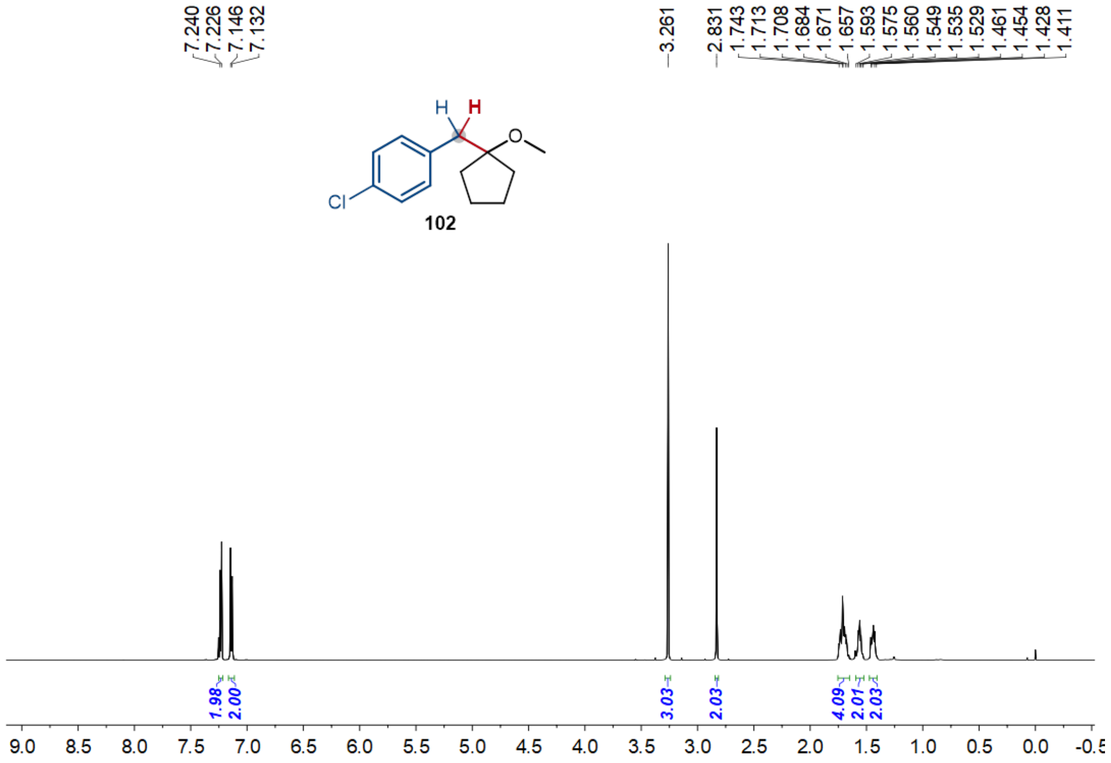

Supplementary Fig. 222 ^{1}H NMR (600 MHz, $CDCl_3$) spectrum of compound **102**

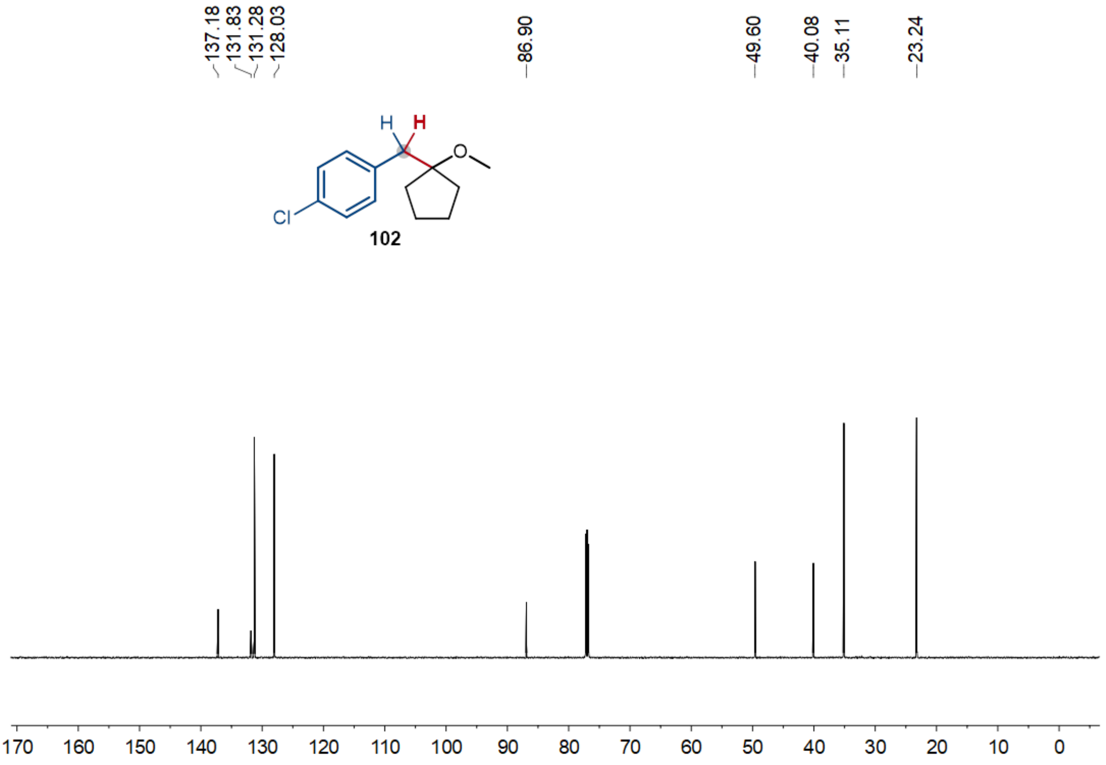

Supplementary Fig. 223 ^{13}C NMR (151 MHz, $CDCl_3$) spectrum of compound

102

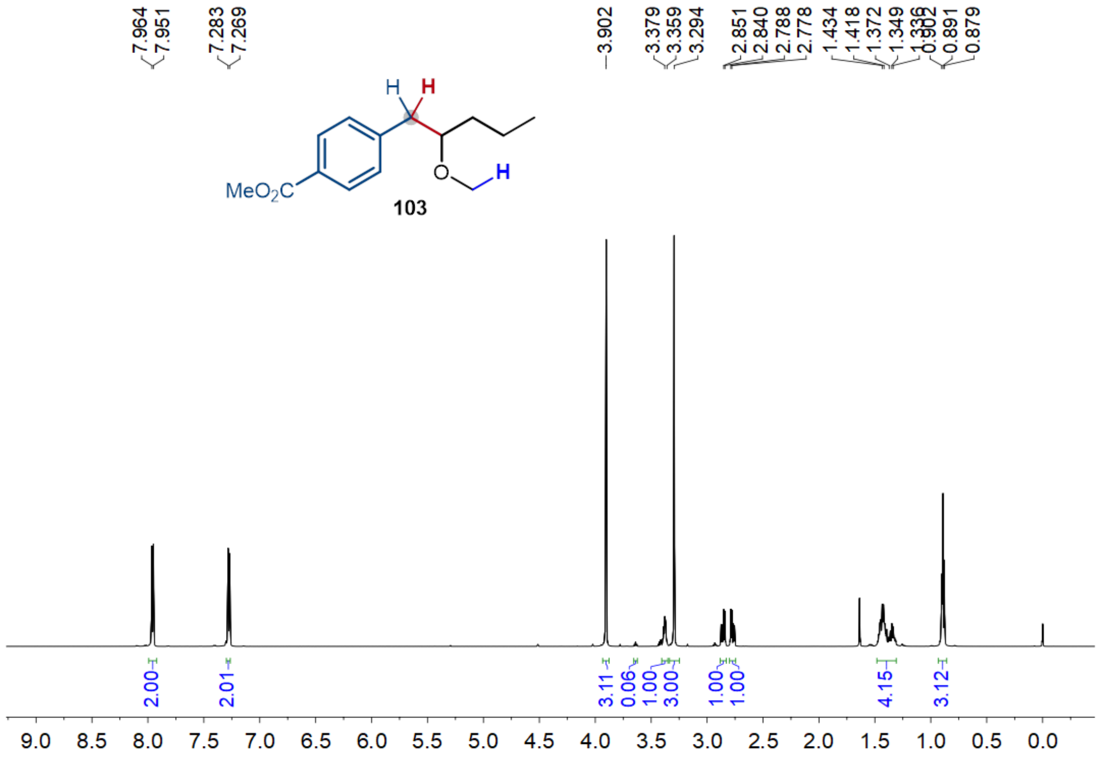

Supplementary Fig. 224 ^{1}H NMR (600 MHz, $CDCl_3$) spectrum of compound **103**

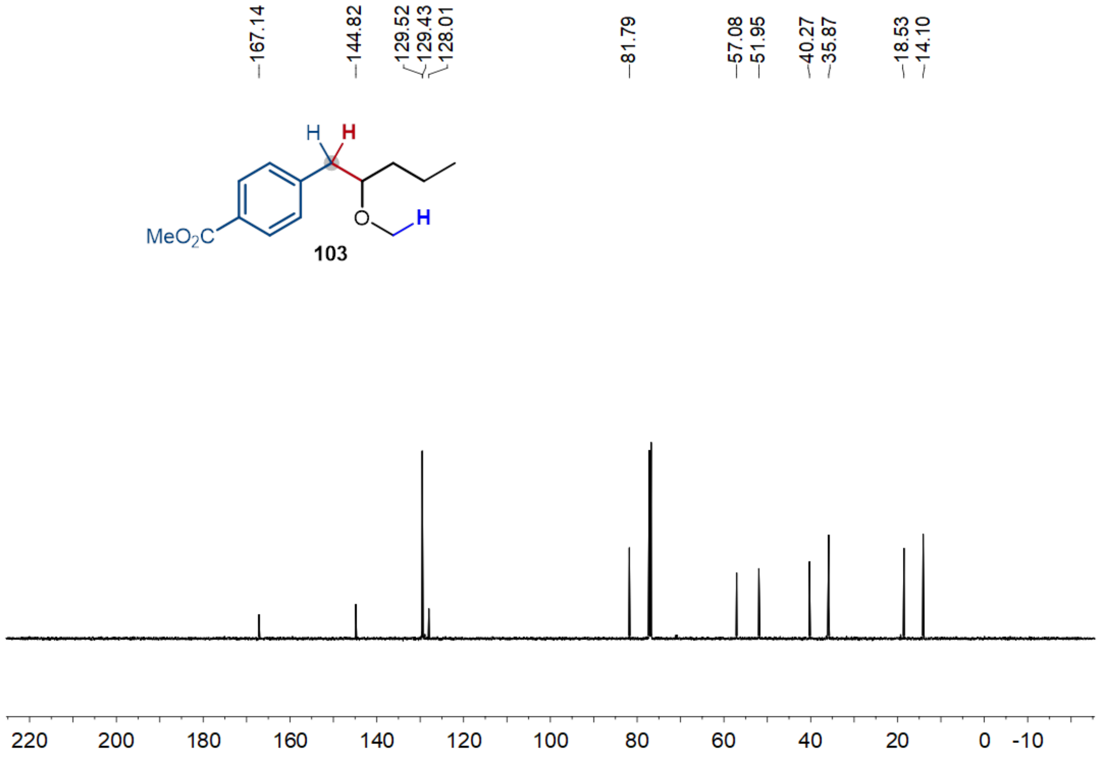

Supplementary Fig. 225 ^{13}C NMR (151 MHz, $CDCl_3$) spectrum of compound

103

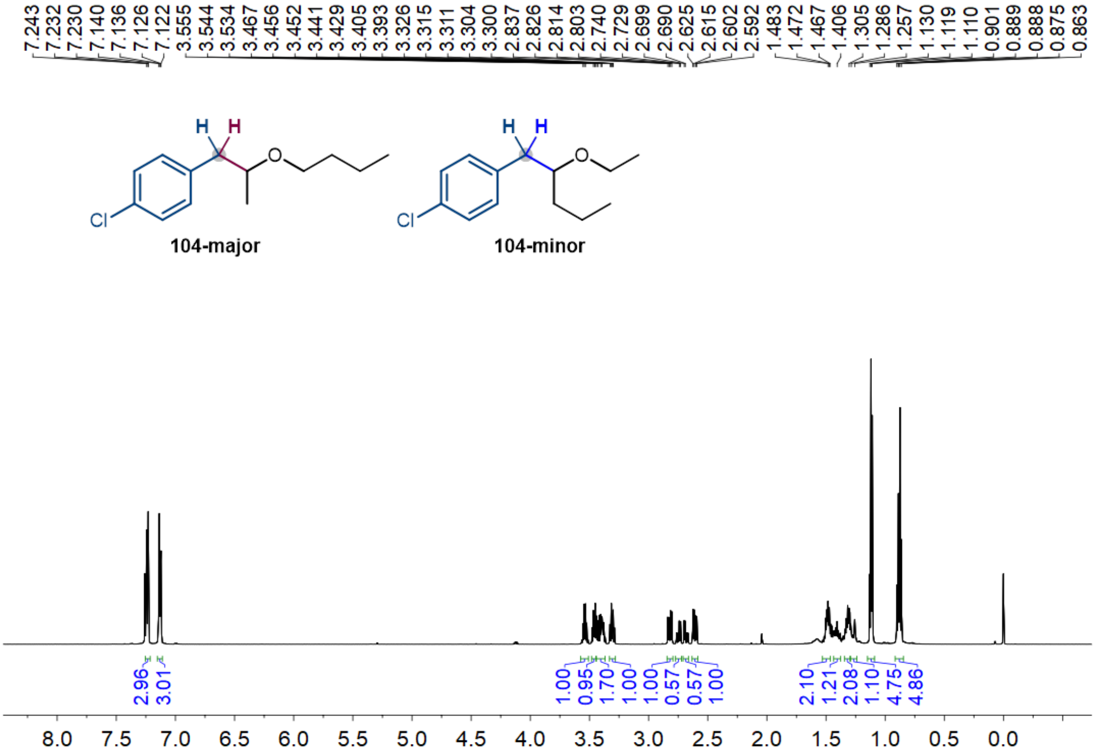

Supplementary Fig. 226 ^{1}H NMR (600 MHz, $CDCl_3$) spectrum of compound **104**

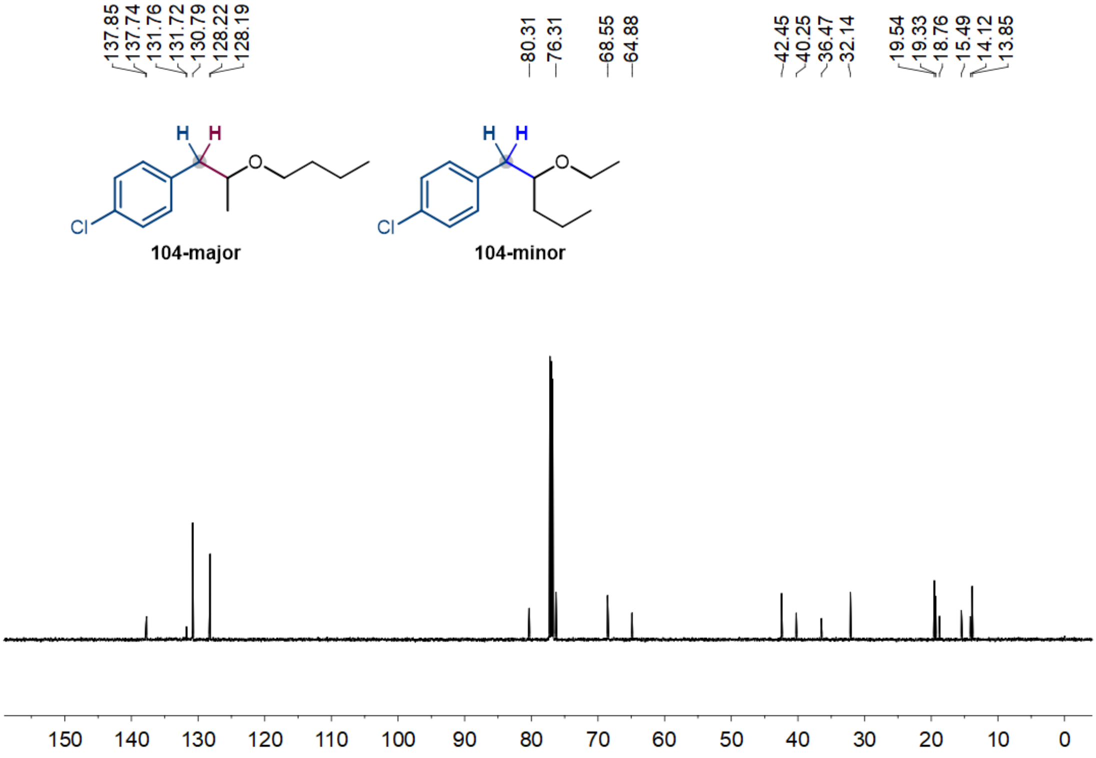

Supplementary Fig. 227 ^{13}C NMR (151 MHz, $CDCl_3$) spectrum of compound

104

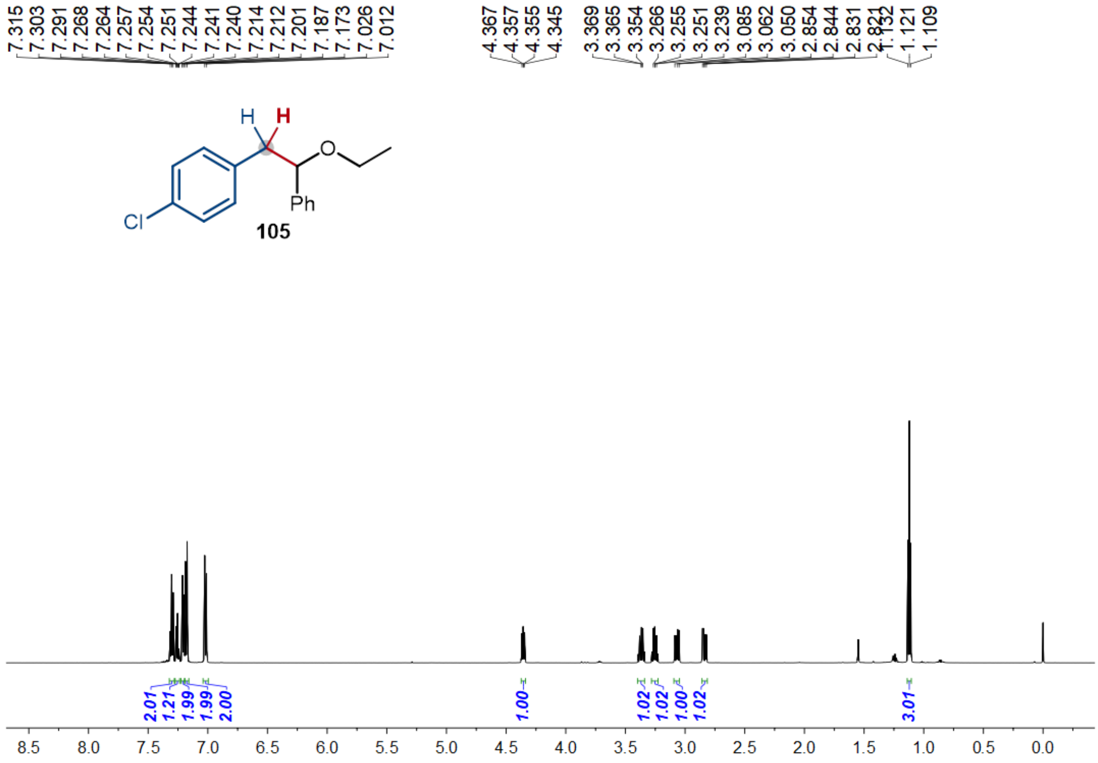

Supplementary Fig. 228 ^{1}**H NMR** (600 MHz, $CDCl_3$) spectrum of compound **105**

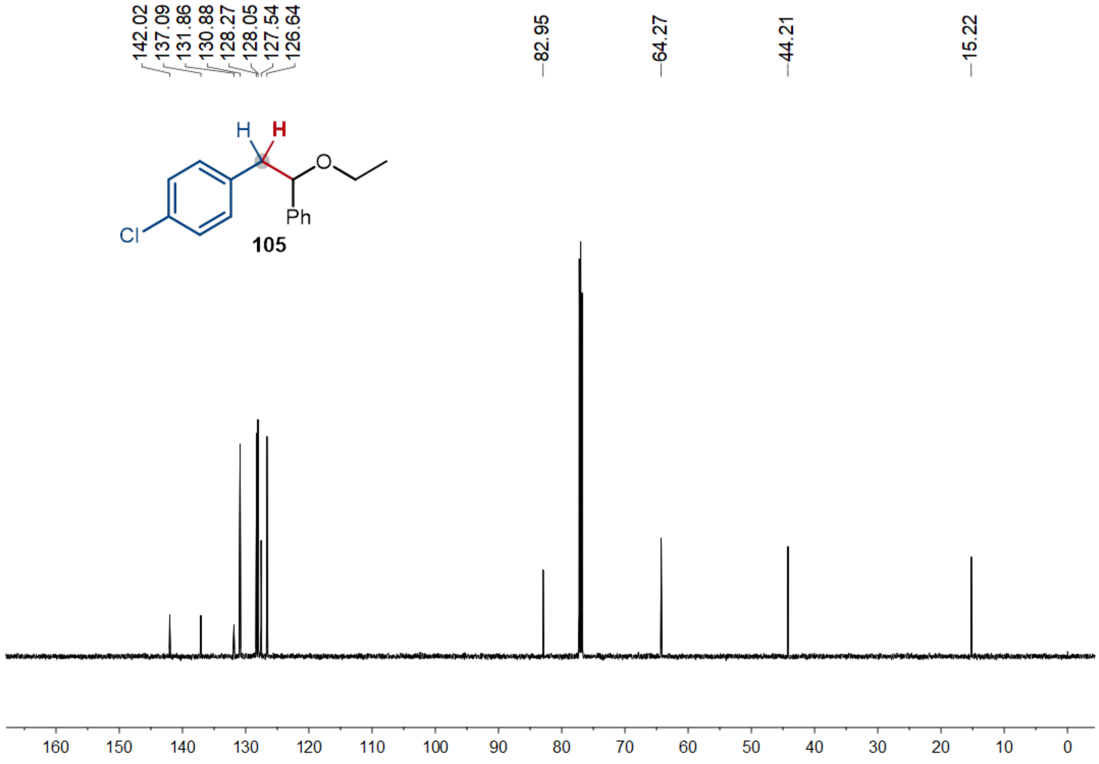

Supplementary Fig. 229 ^{13}**C NMR** (151 MHz, $CDCl_3$) spectrum of compound **105**

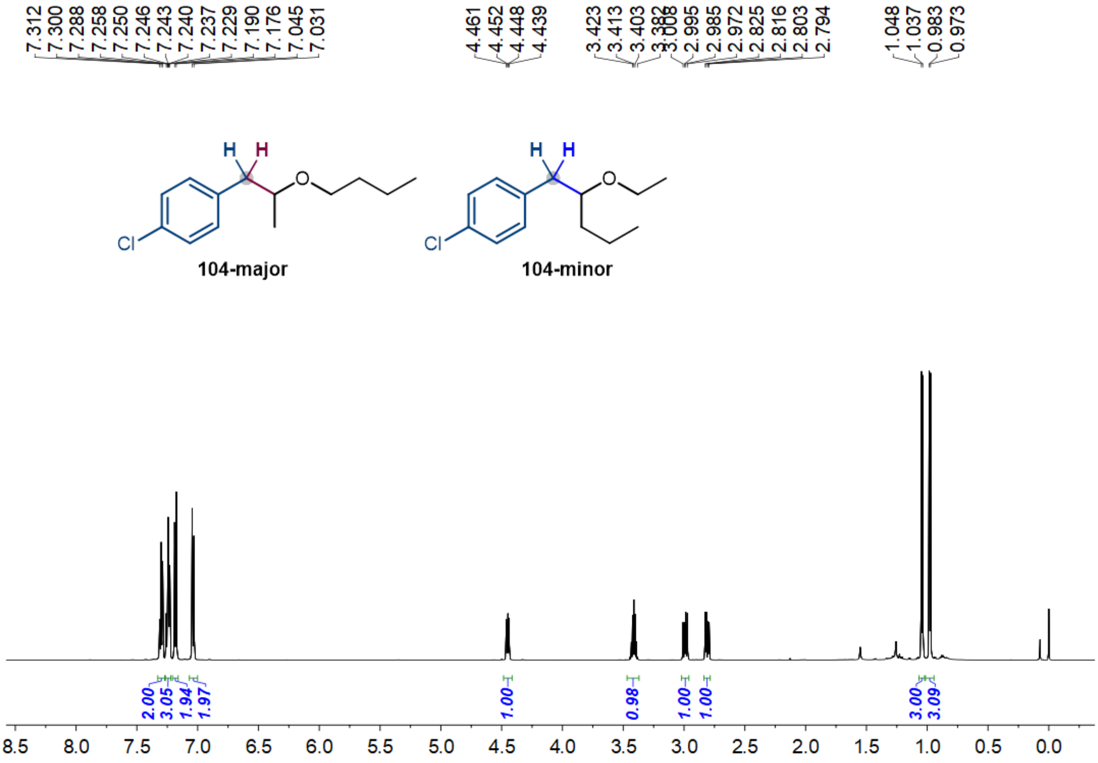

Supplementary Fig. 230 ^{1}**H NMR** (600 MHz, $CDCl_3$) spectrum of compound **106**

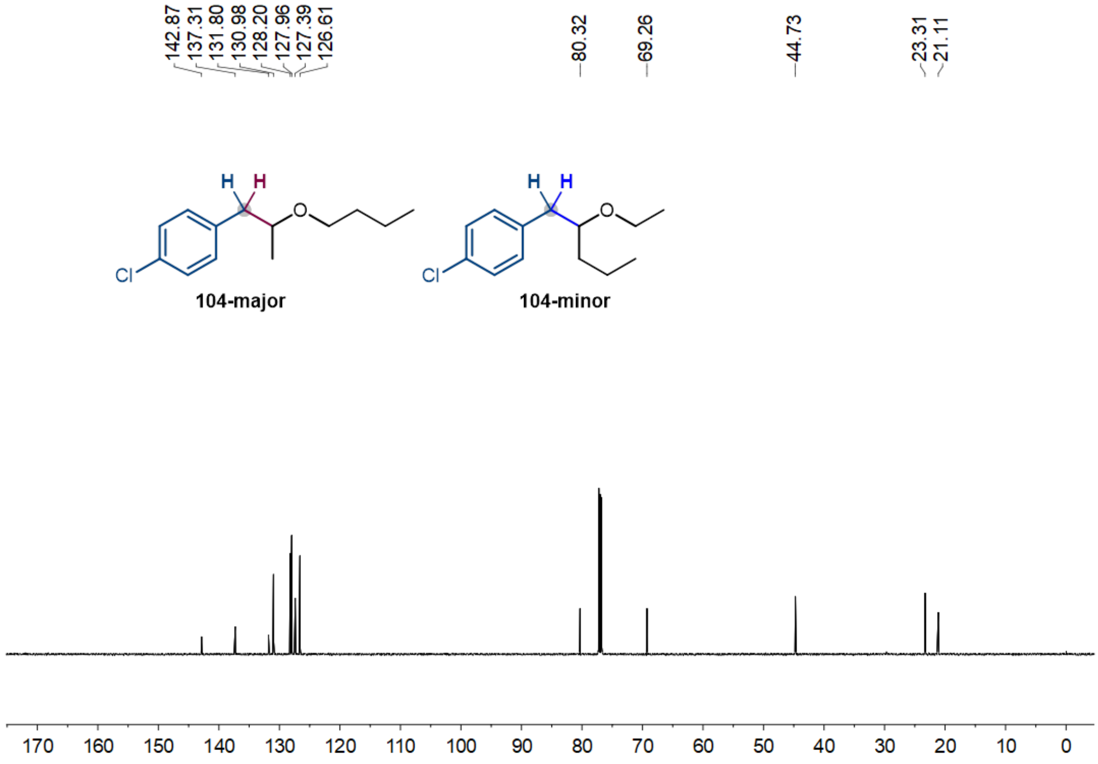

Supplementary Fig. 231 ^{13}**C NMR** (151 MHz, $CDCl_3$) spectrum of compound

106

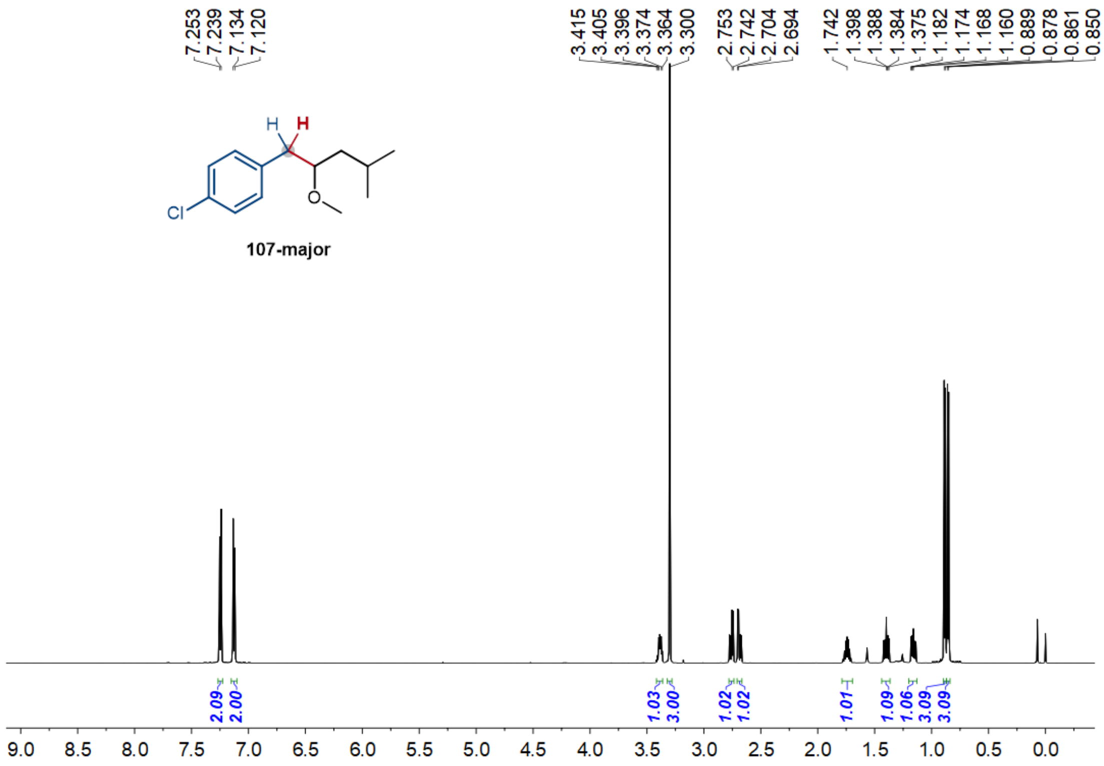

Supplementary Fig. 232 ^{1}H NMR (600 MHz, $CDCl_3$) spectrum of compound **107-major**

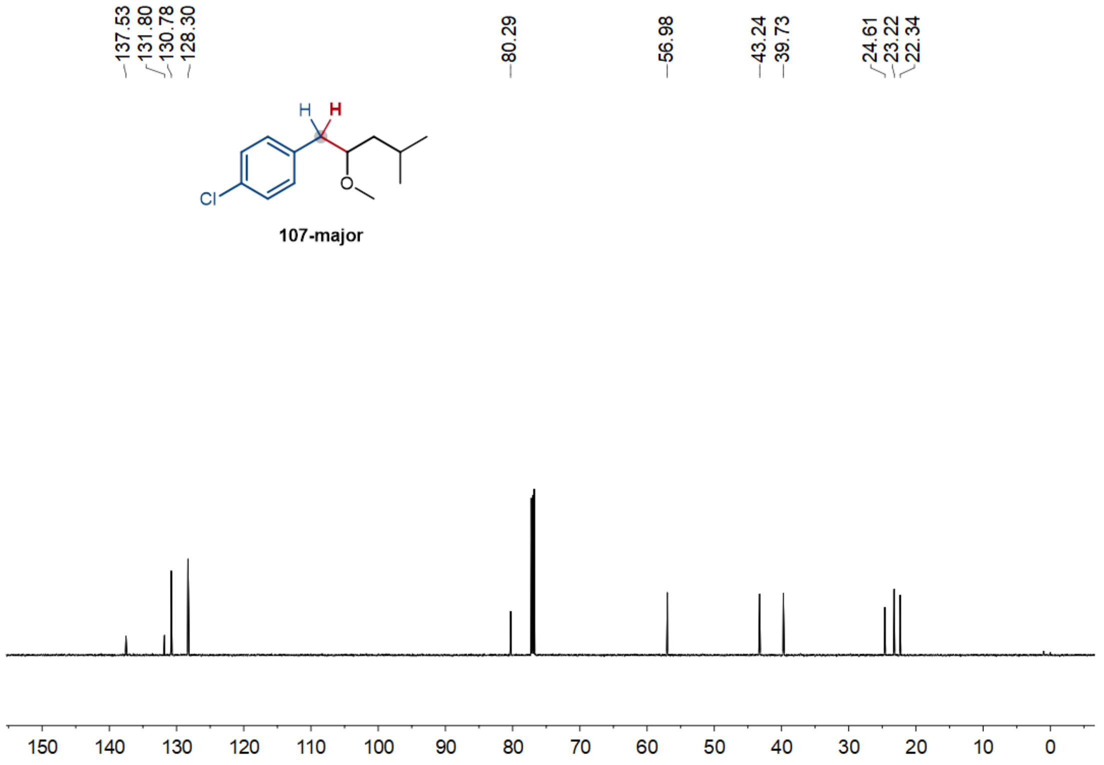

Supplementary Fig. 233 ^{13}C NMR (151 MHz, $CDCl_3$) spectrum of compound **107-major**

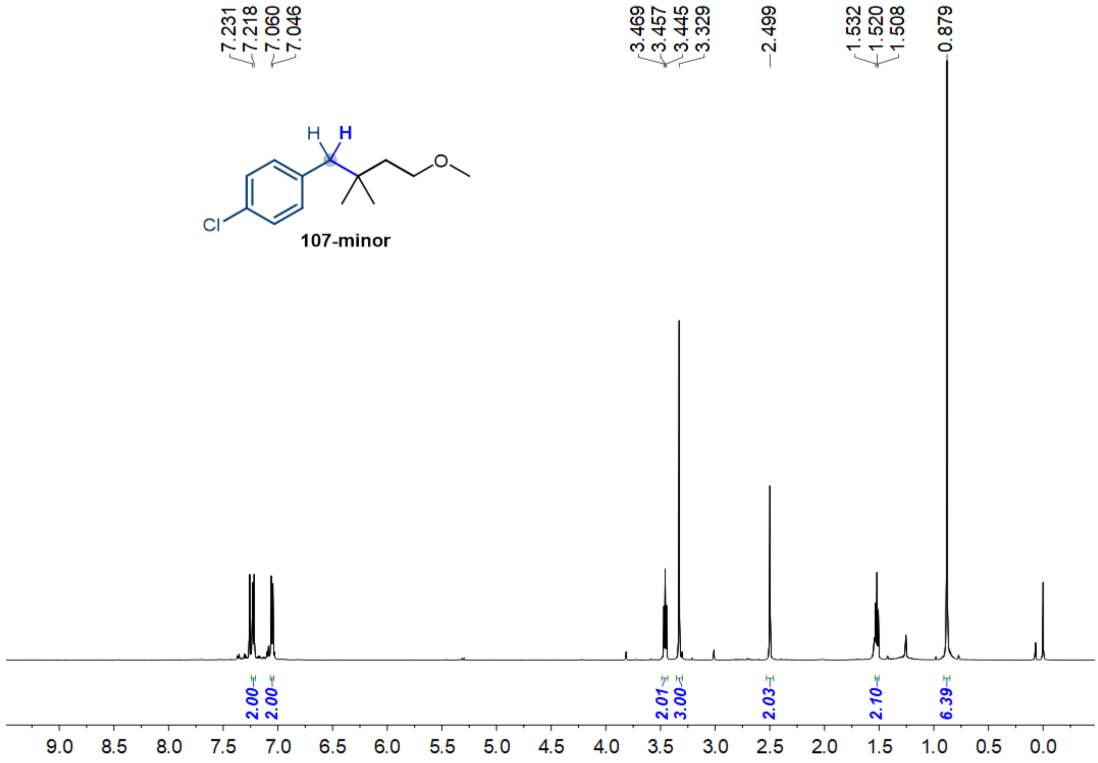

Supplementary Fig. 234 ^{1}H NMR (600 MHz, $CDCl_3$) spectrum of compound **107-minor**

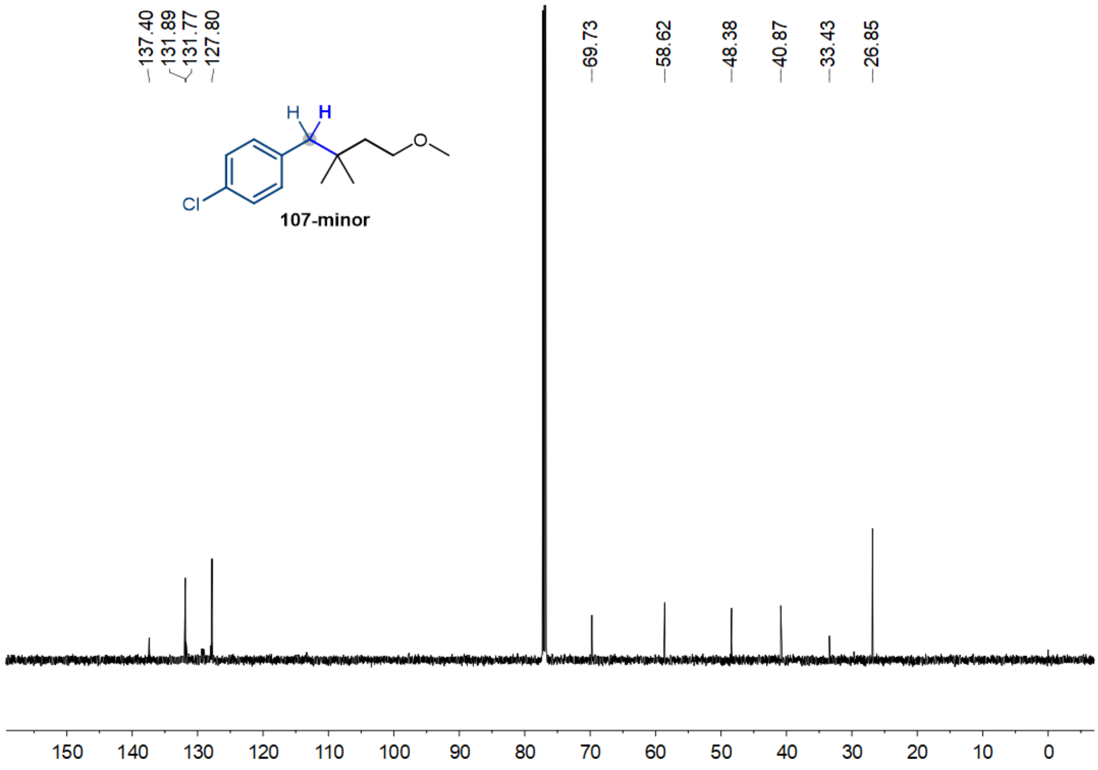

Supplementary Fig. 235 ^{13}C NMR (151 MHz, $CDCl_3$) spectrum of compound **107-minor**

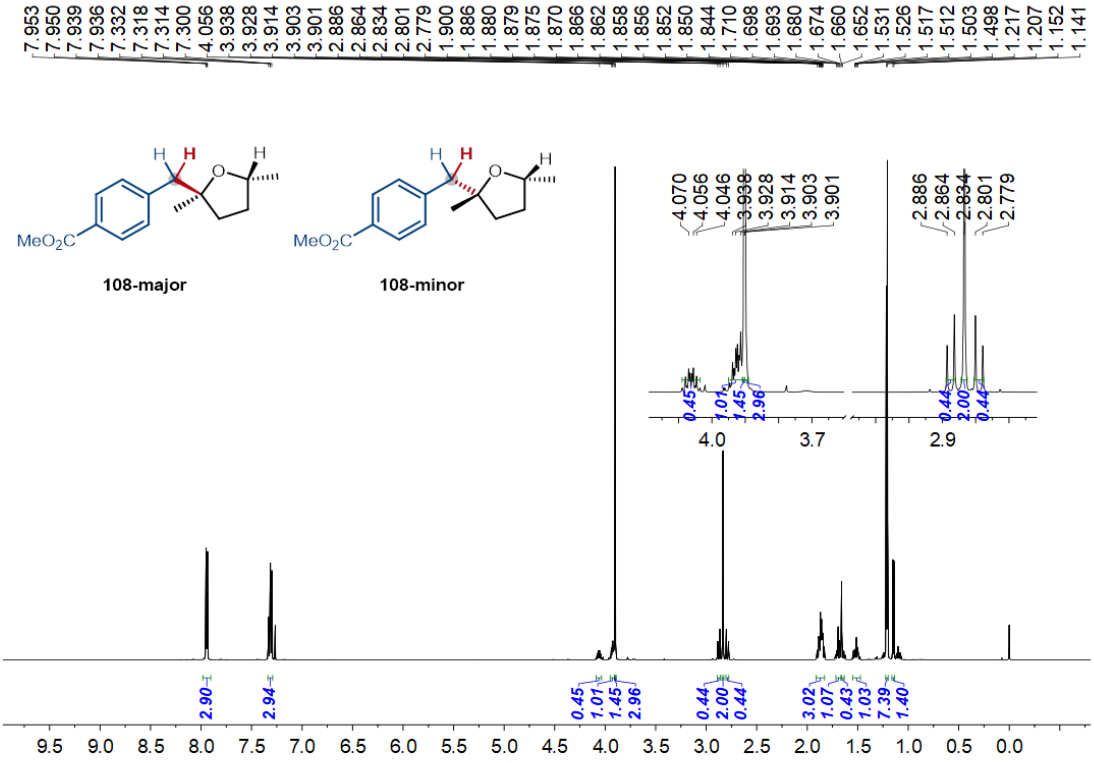

Supplementary Fig. 236 ^{1}**H NMR** (600 MHz, $CDCl_3$) spectrum of compound **108**

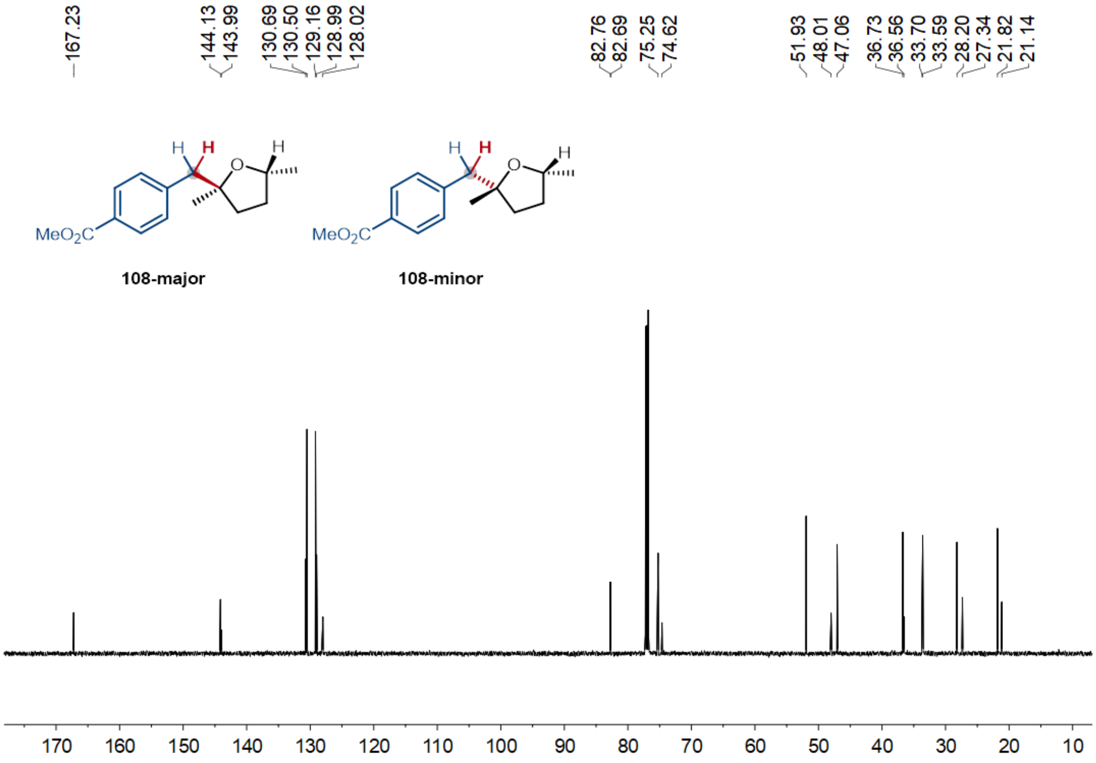

Supplementary Fig. 238 ^{13}**C NMR** (151 MHz, $CDCl_3$) spectrum of compound **108**

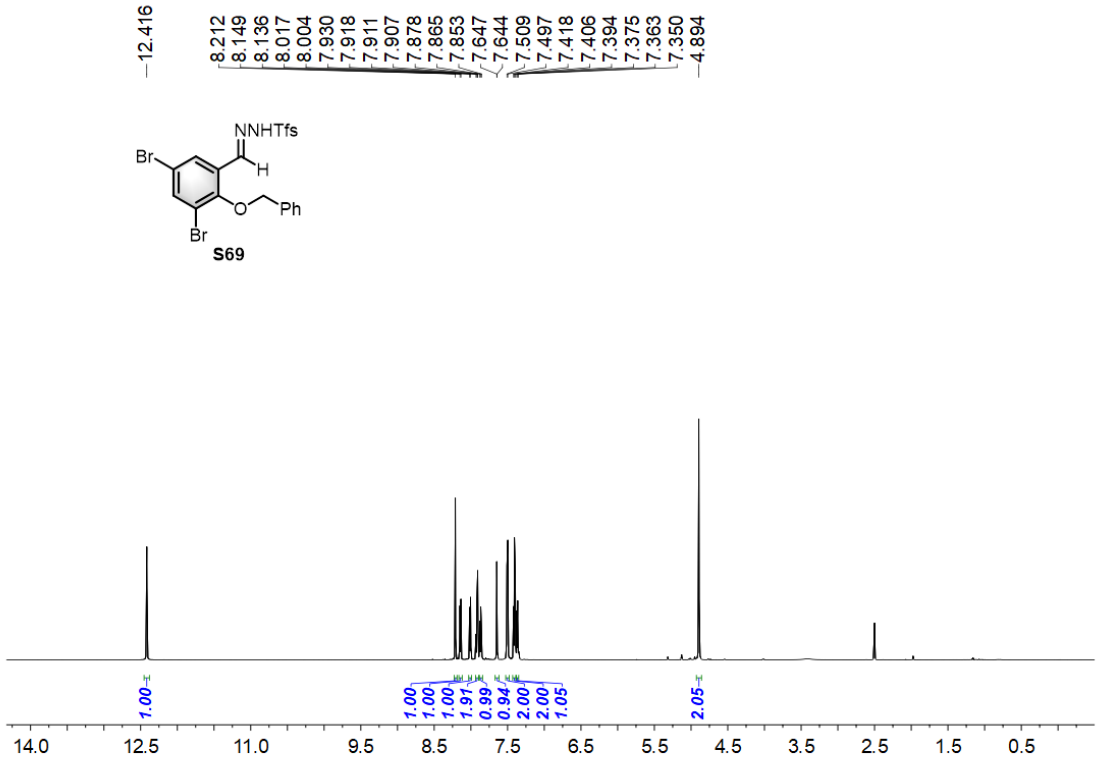

Supplementary Fig. 239 ^{1}H NMR (600 MHz, DMSO) spectrum of compound **S69**

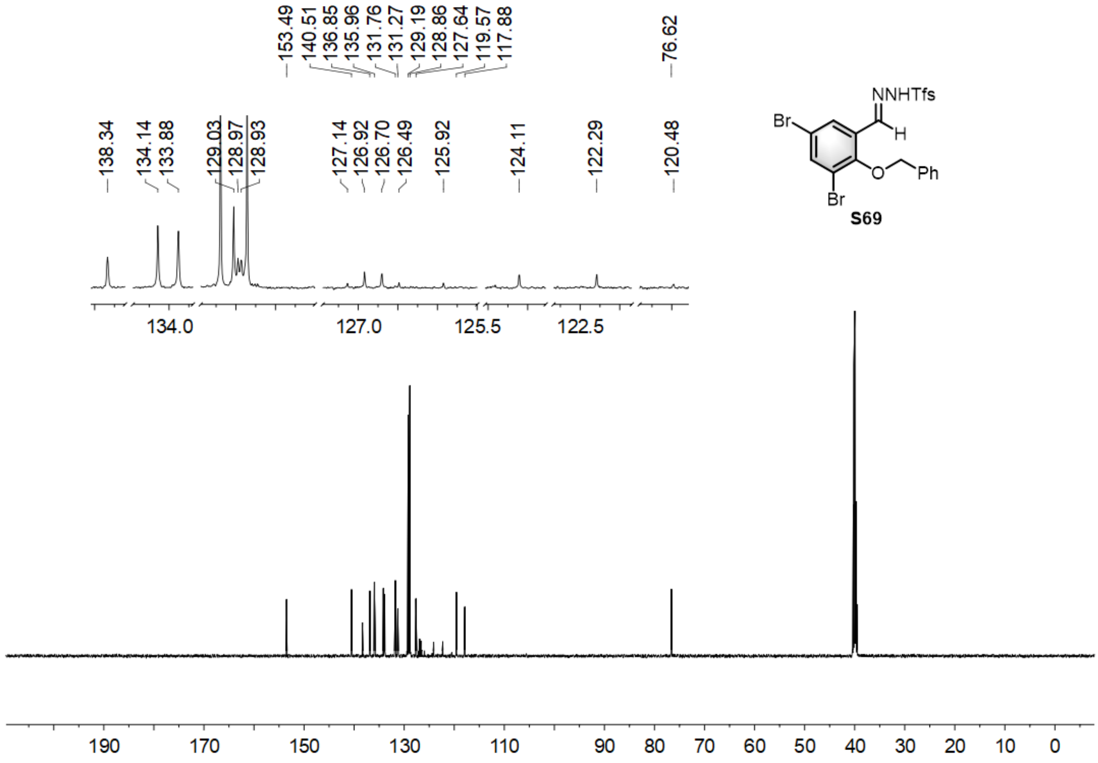

Supplementary Fig. 240 ^{13}C NMR (151 MHz, DMSO) spectrum of compound **S69**

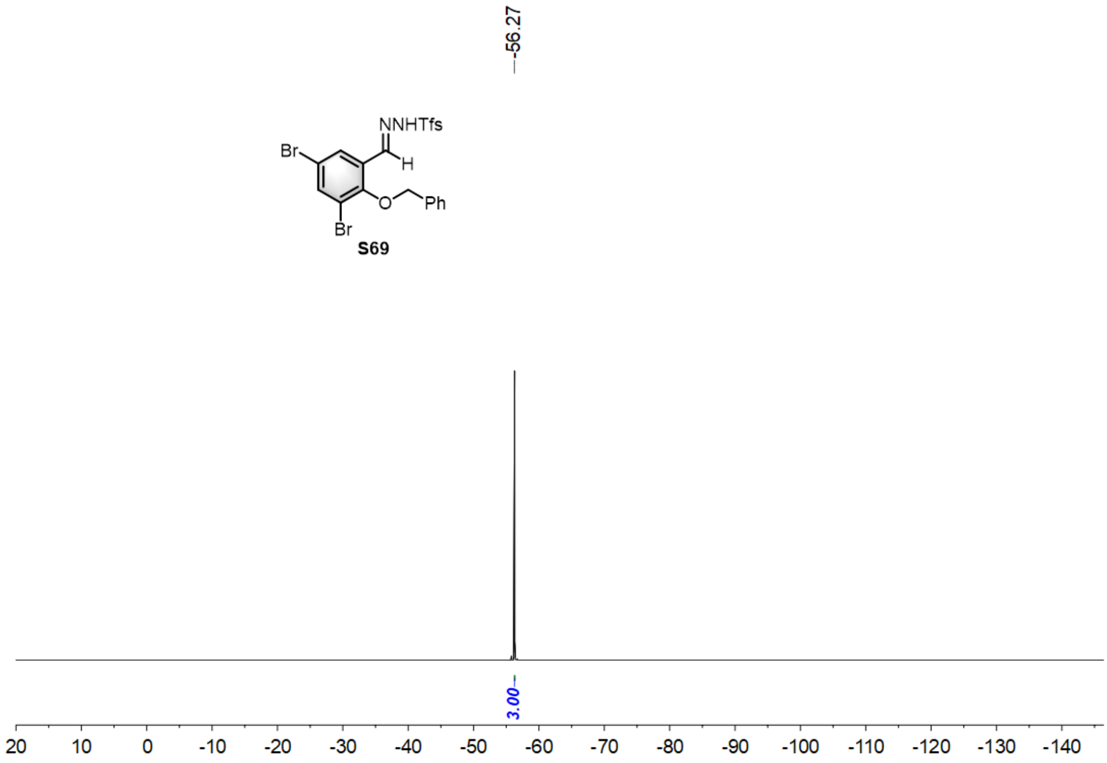

Supplementary Fig. 241 ^{19}**F NMR** (471 MHz, DMSO) spectrum of compound **S69**

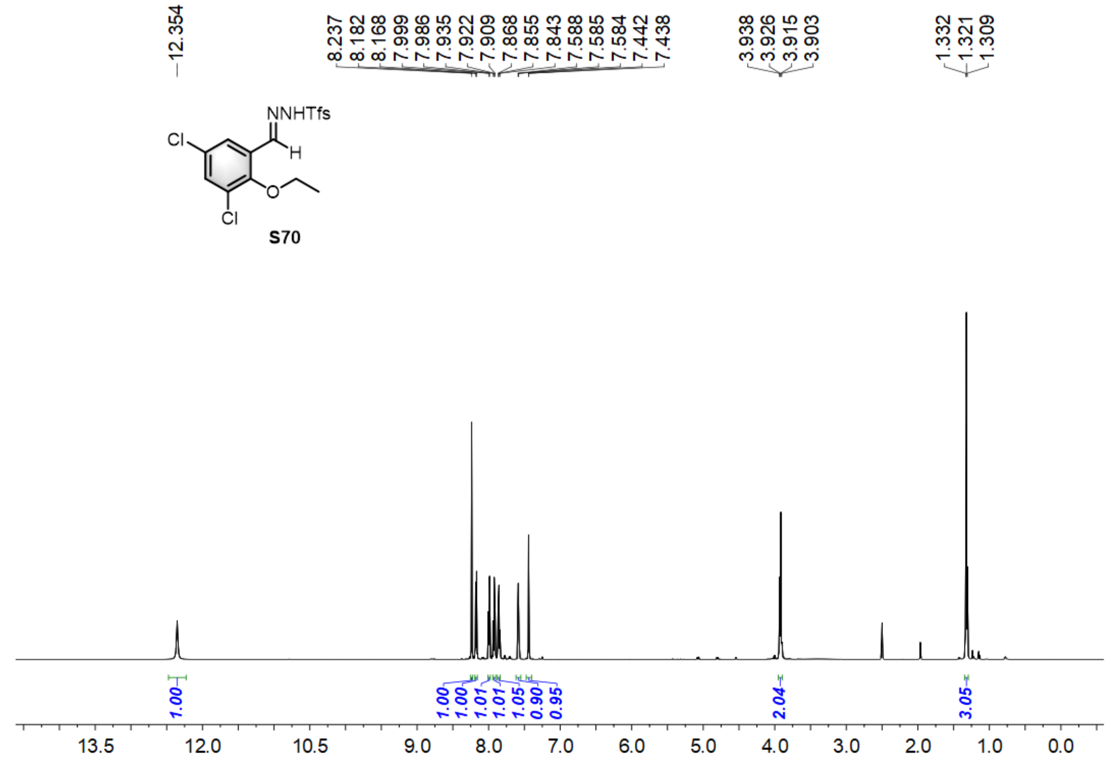

Supplementary Fig. 242 ^{1}**H NMR** (600 MHz, DMSO) spectrum of compound **S70**

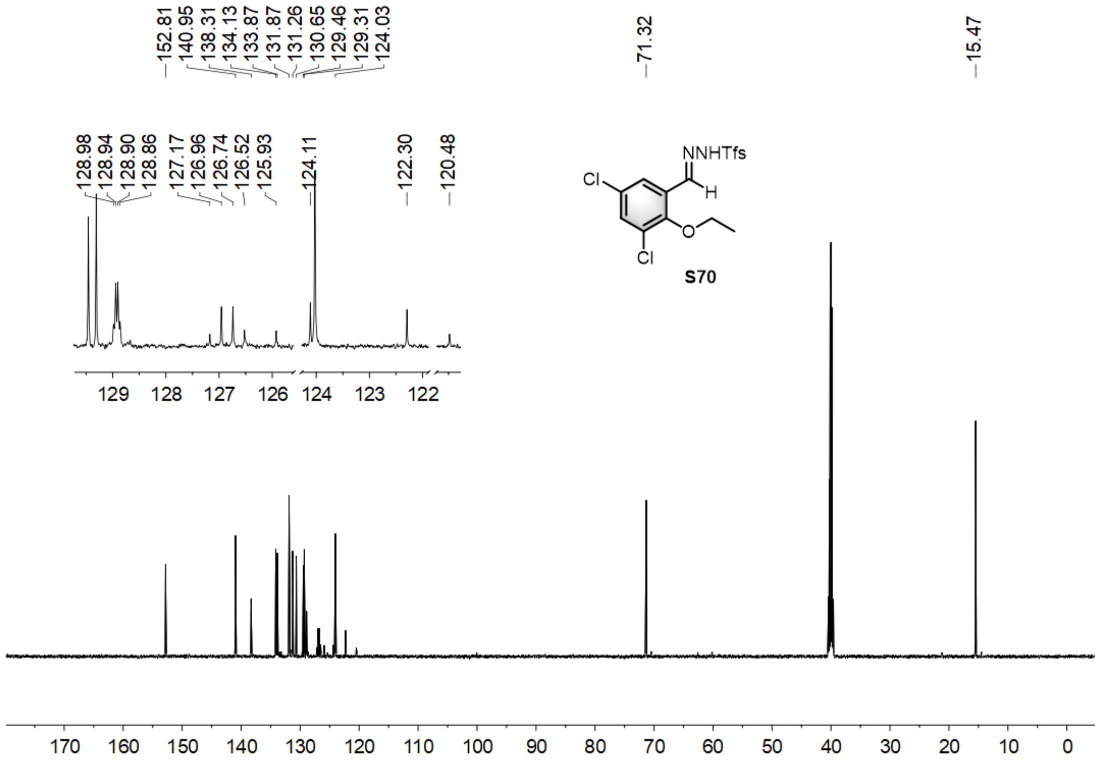

Supplementary Fig. 243 ^{13}**C NMR** (151 MHz, DMSO) spectrum of compound **S70**

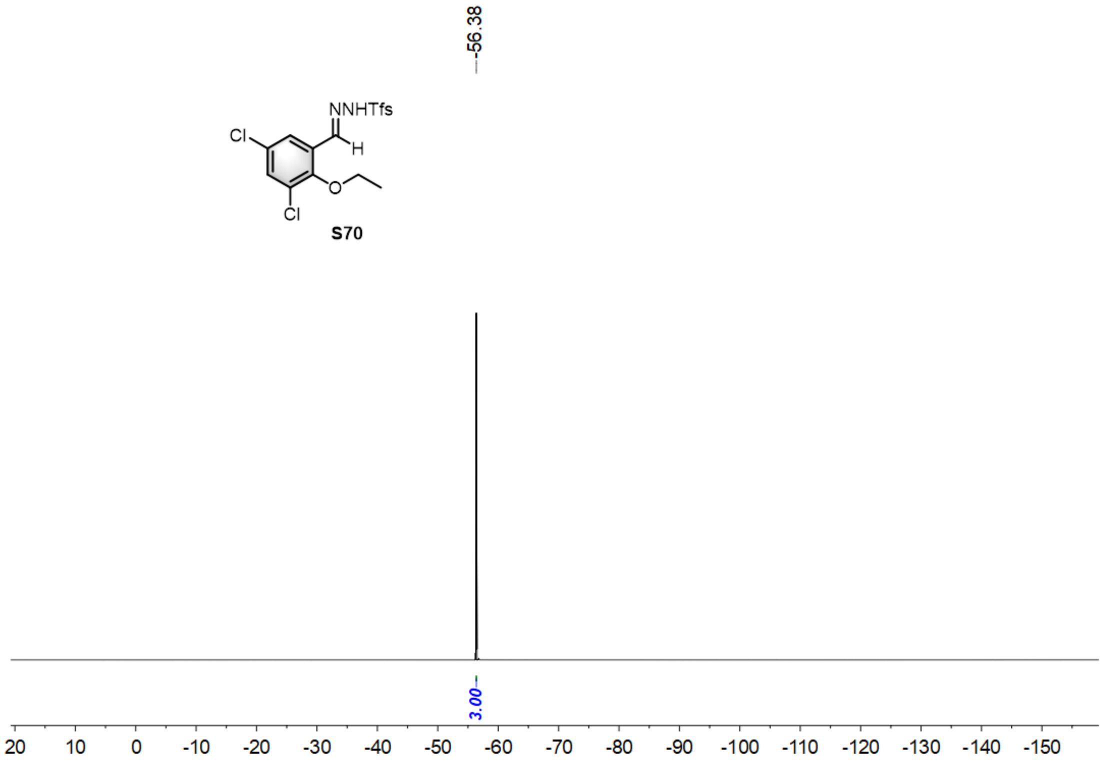

Supplementary Fig. 244 ^{19}**F NMR** (471 MHz, DMSO) spectrum of compound **S70**

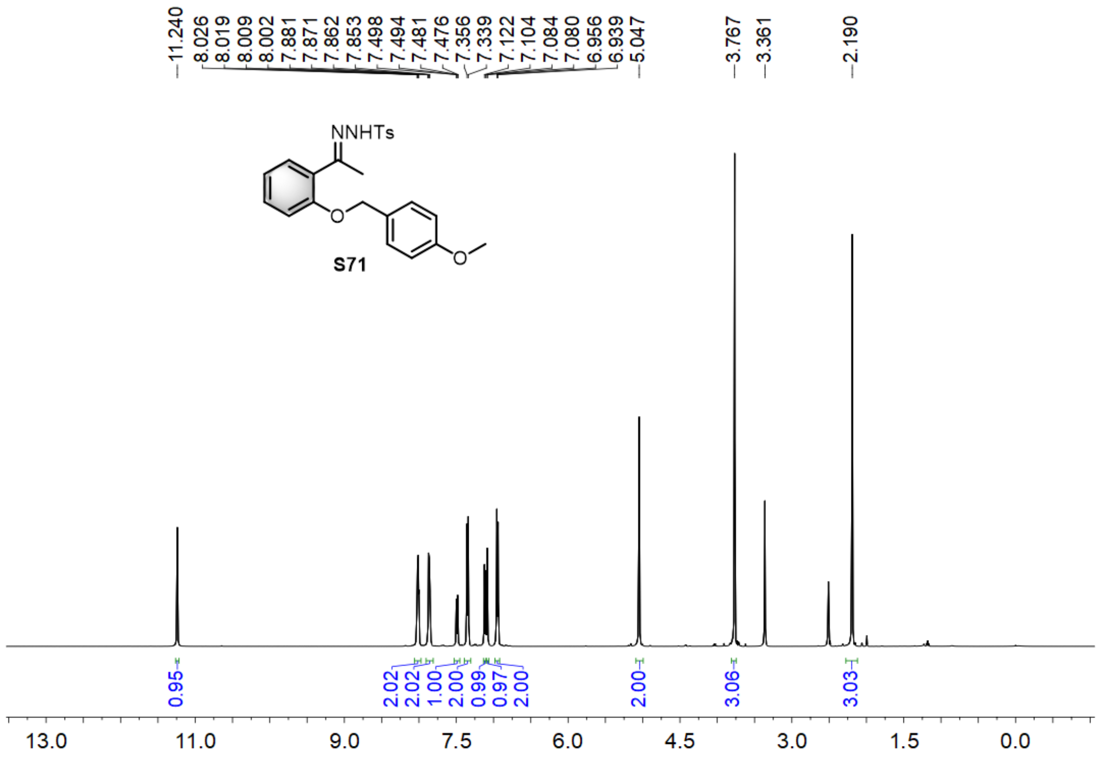

Supplementary Fig. 245 ^{1}H NMR (600 MHz, DMSO) spectrum of compound **S71**

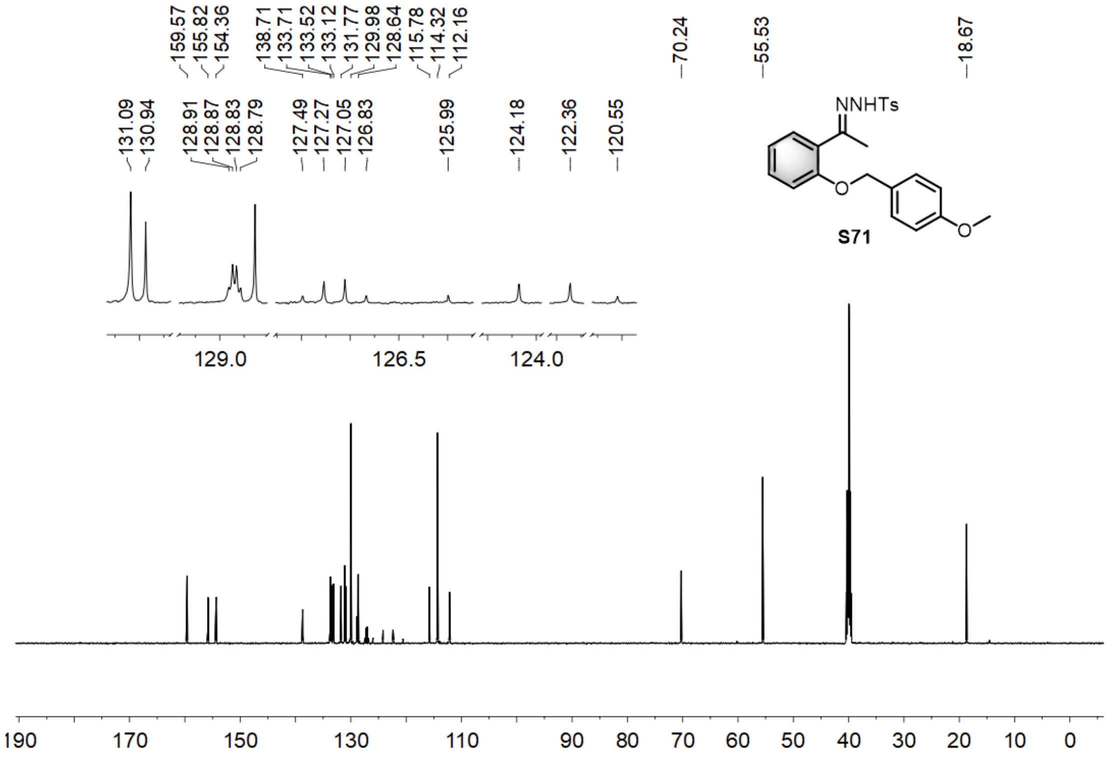

Supplementary Fig. 246 ^{13}C NMR (151 MHz, DMSO) spectrum of compound **S71**

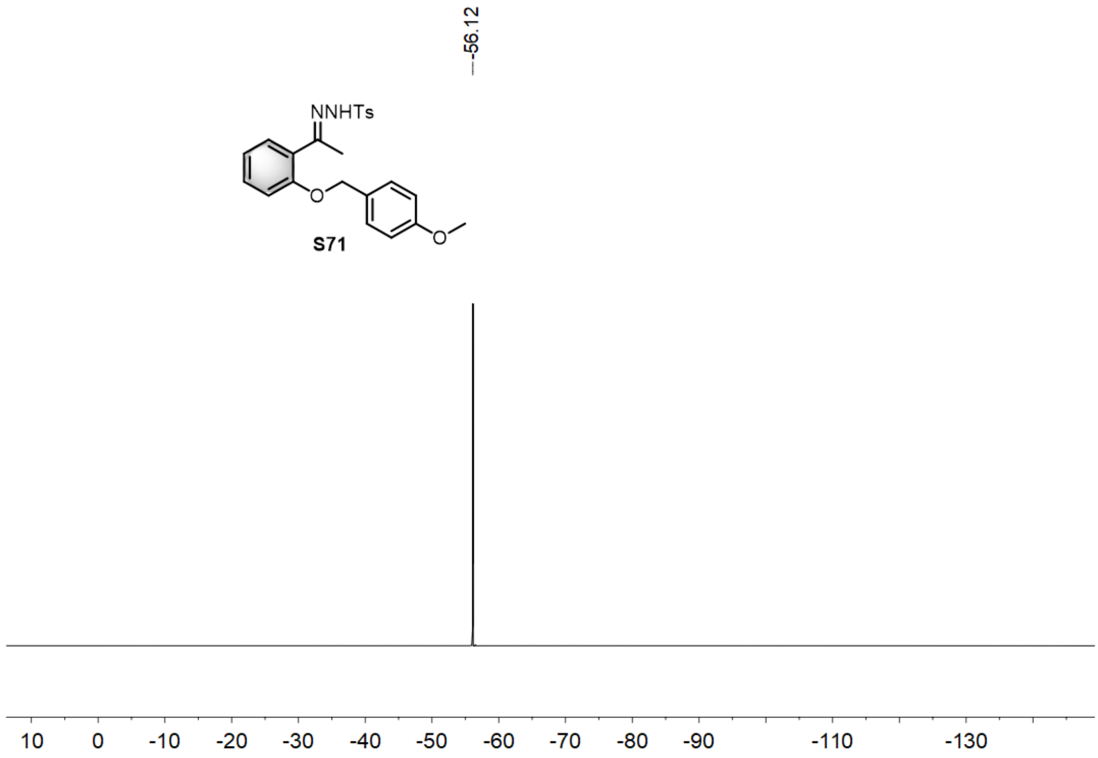

Supplementary Fig. 247 ^{19}F NMR (471 MHz, DMSO) spectrum of compound **S71**

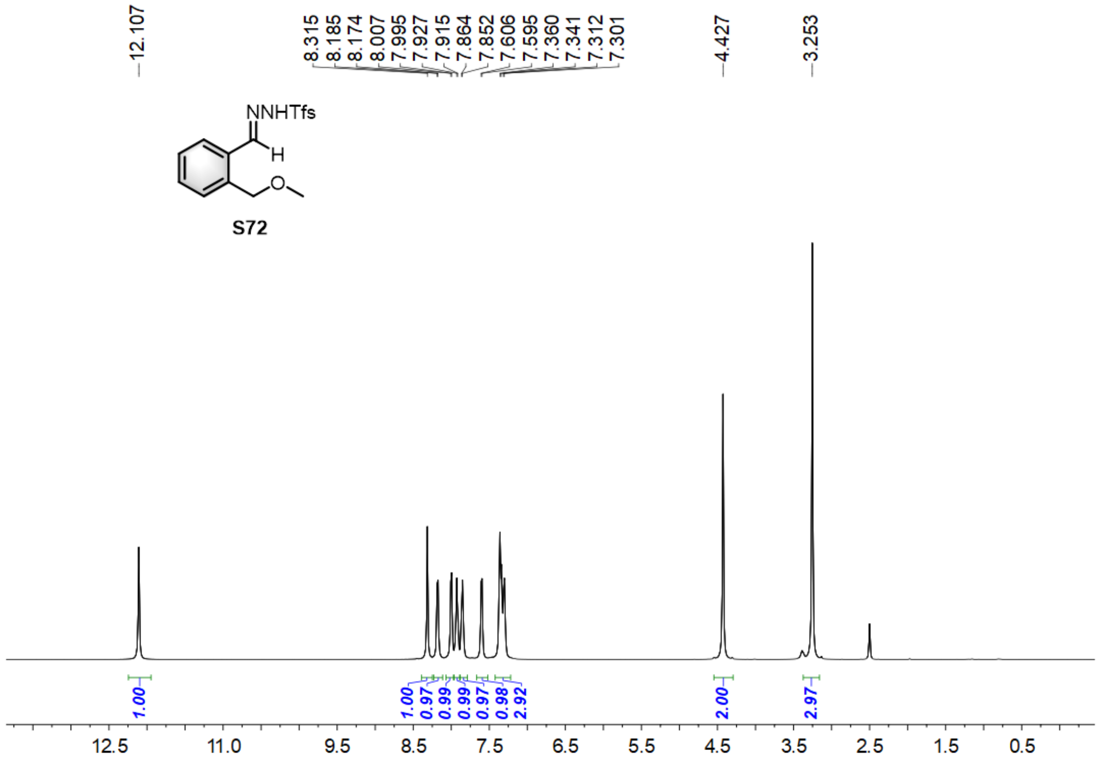

Supplementary Fig. 248 ^{1}H NMR (600 MHz, DMSO) spectrum of compound **S72**

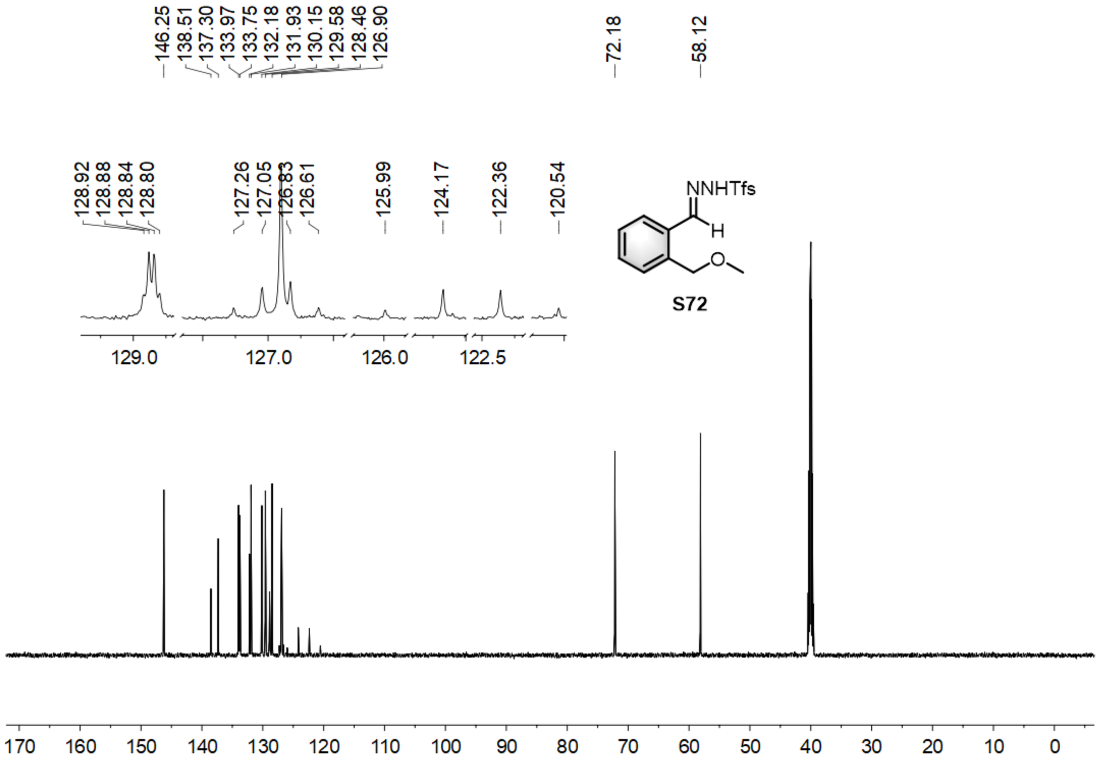

Supplementary Fig. 249 ^{13}C NMR (151 MHz, DMSO) spectrum of compound **S72**

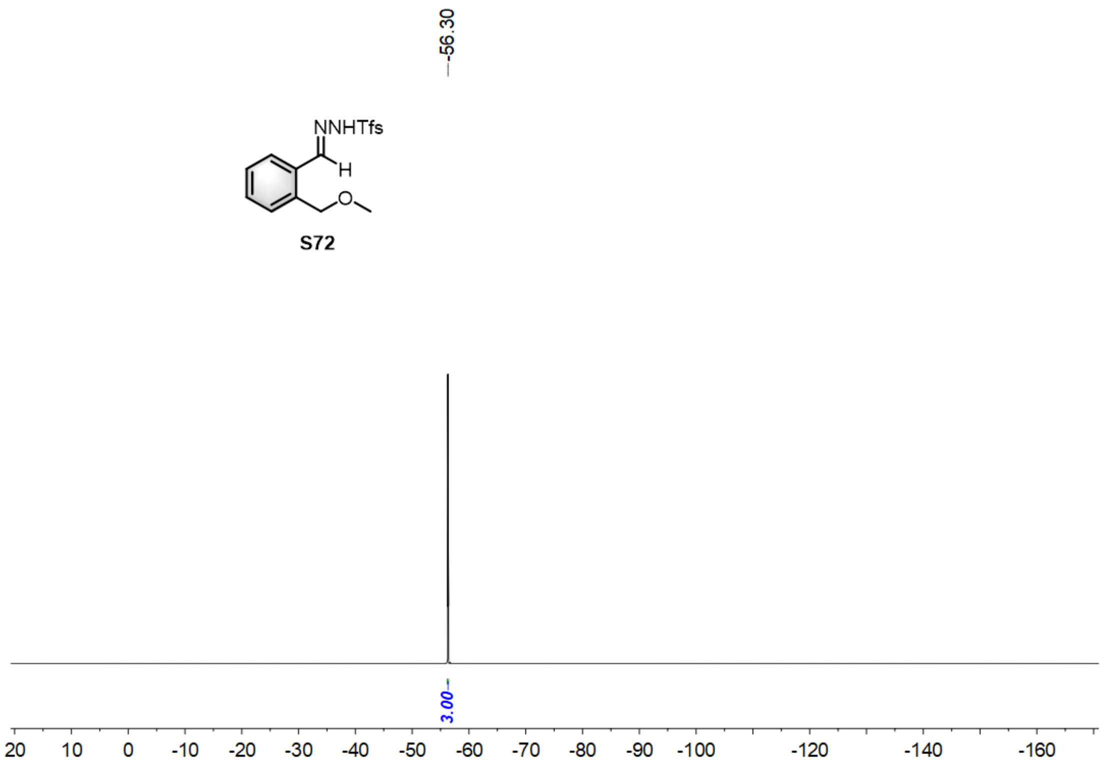

Supplementary Fig. 250 ^{19}F NMR (471 MHz, DMSO) spectrum of compound **S72**

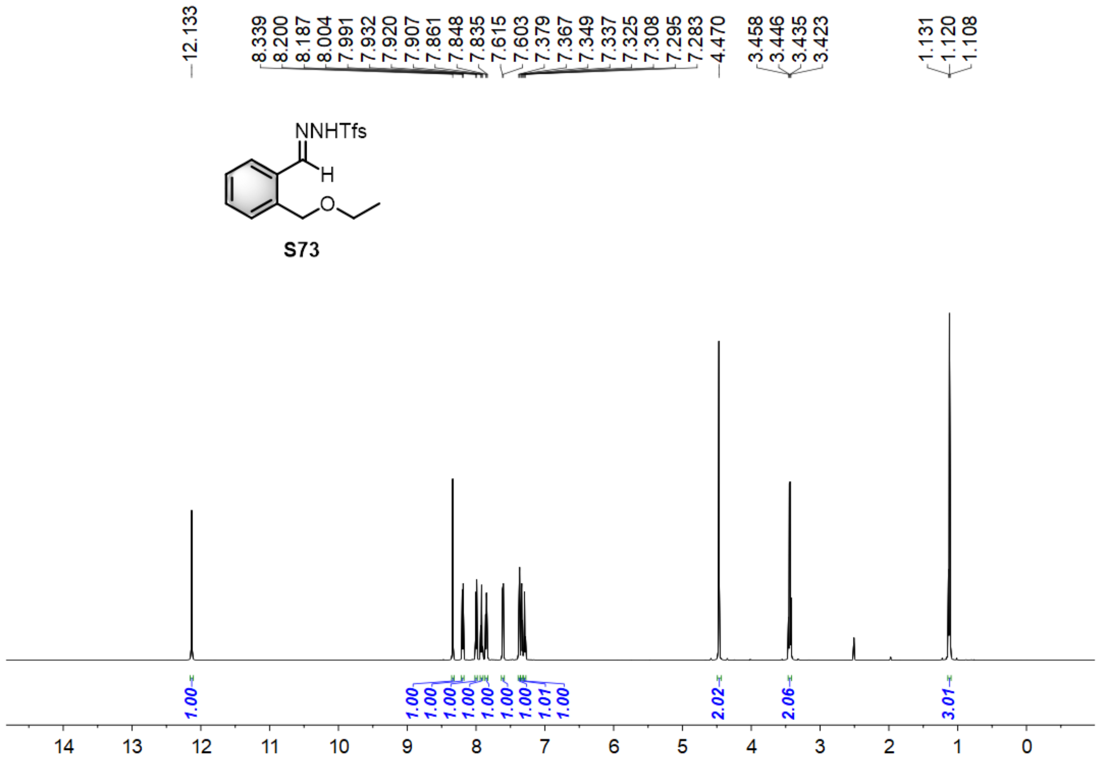

Supplementary Fig. 251 ^{1}**H NMR** (600 MHz, DMSO) spectrum of compound **S73**

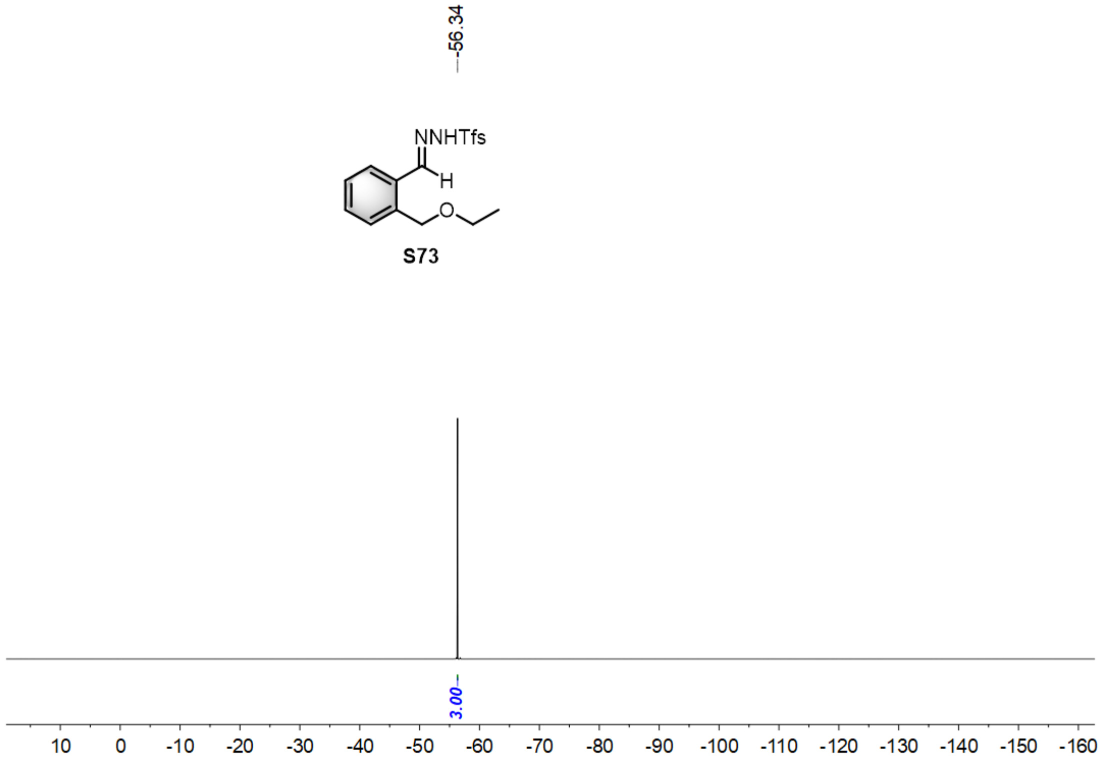

Supplementary Fig. 252 ^{19}**F NMR** (471 MHz, DMSO) spectrum of compound **S73**

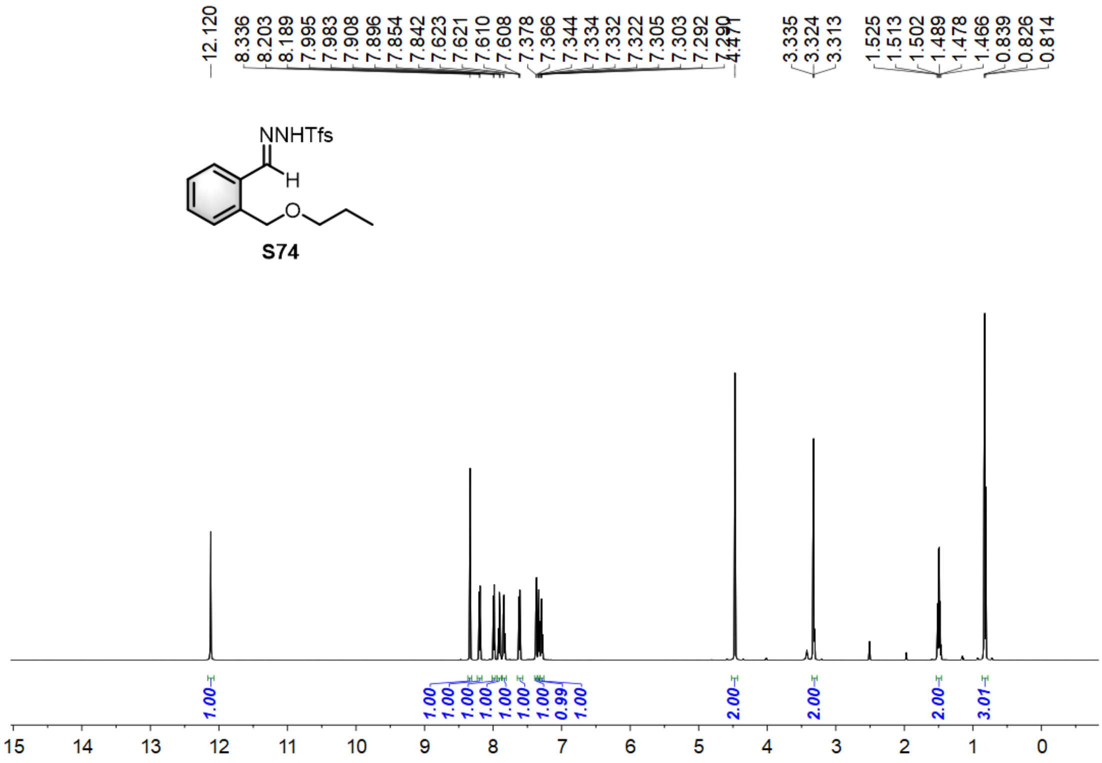

Supplementary Fig. 253 ^{1}H NMR (600 MHz, DMSO) spectrum of compound **S74**

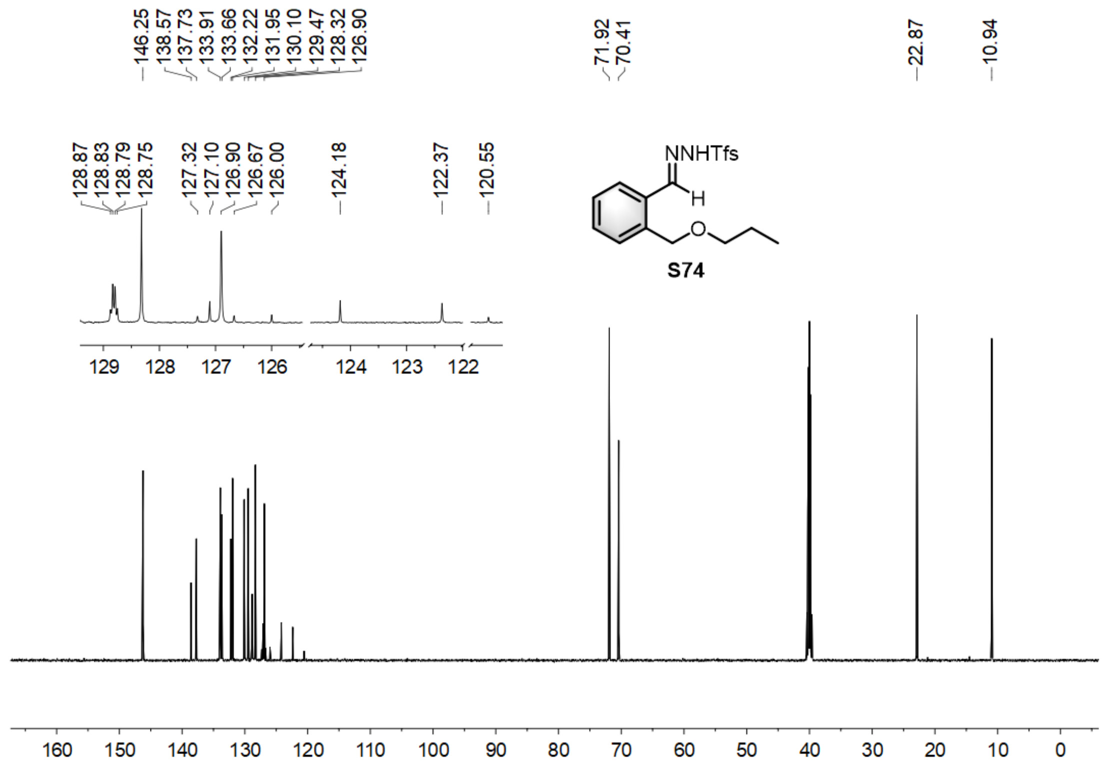

Supplementary Fig. 254 ^{13}C NMR (151 MHz, DMSO) spectrum of compound **S74**

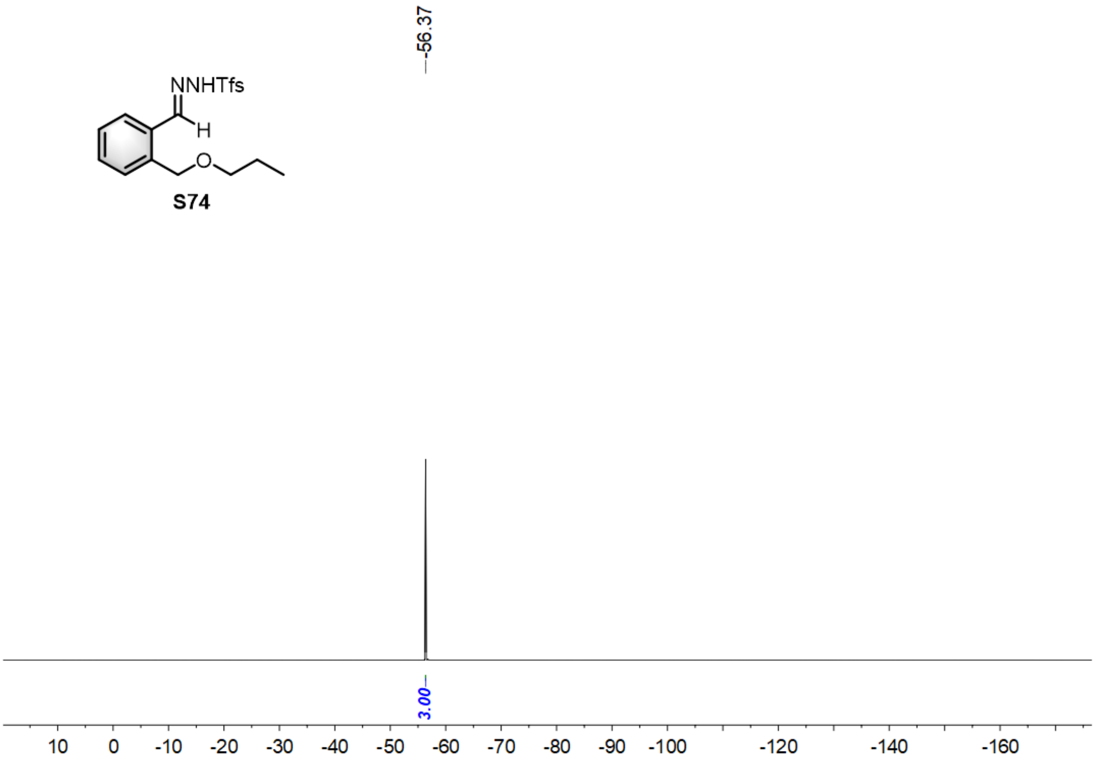

Supplementary Fig. 255 ^{19}**F NMR** (471 MHz, DMSO) spectrum of compound **S74**

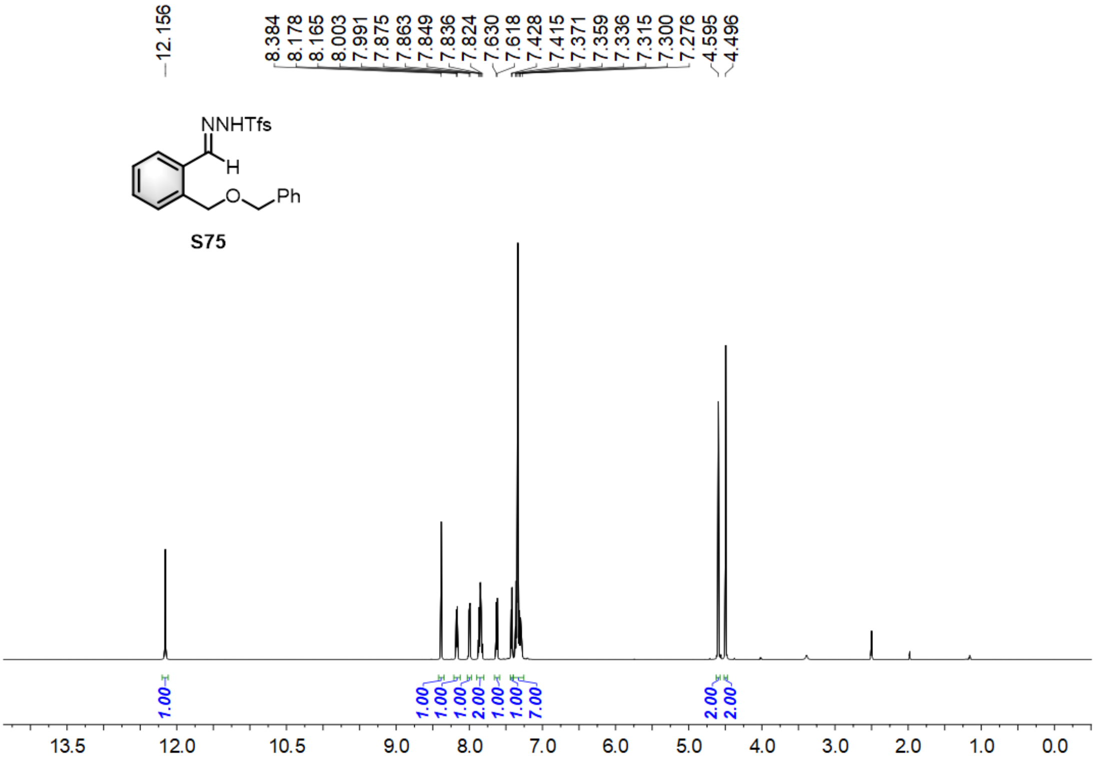

Supplementary Fig. 256 ^{1}**H NMR** (600 MHz, DMSO) spectrum of compound **S75**

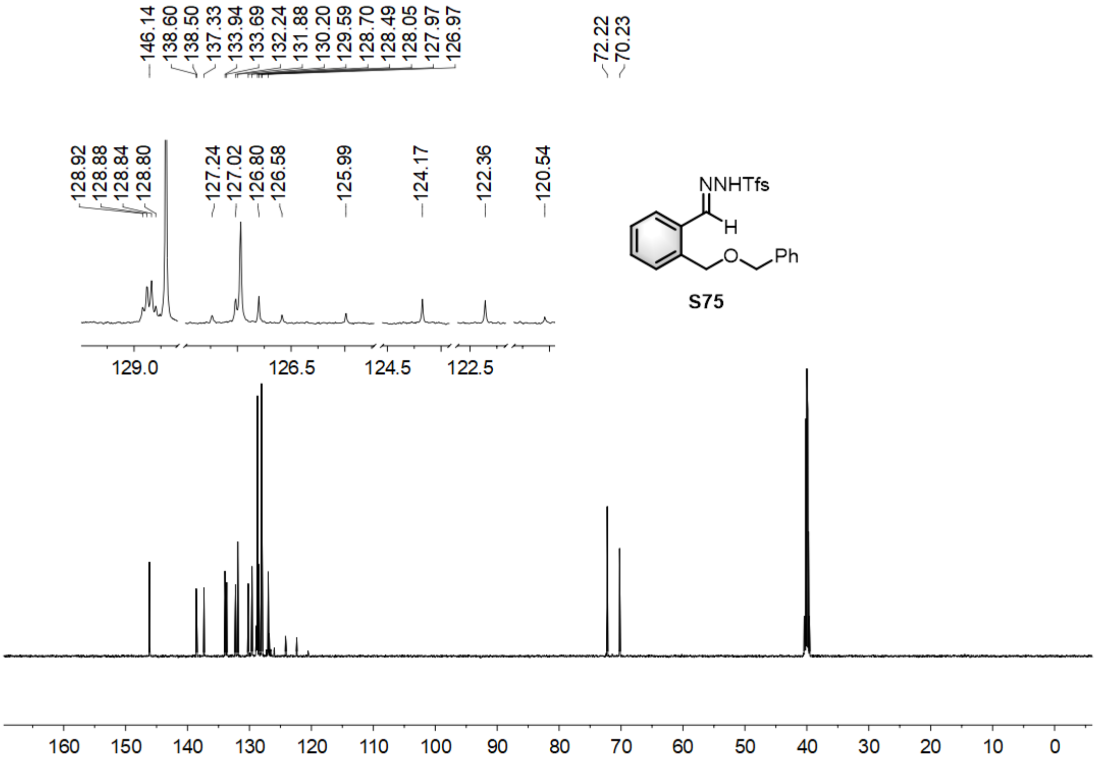

Supplementary Fig. 257 ^{13}C NMR (151 MHz, DMSO) spectrum of compound **S75**

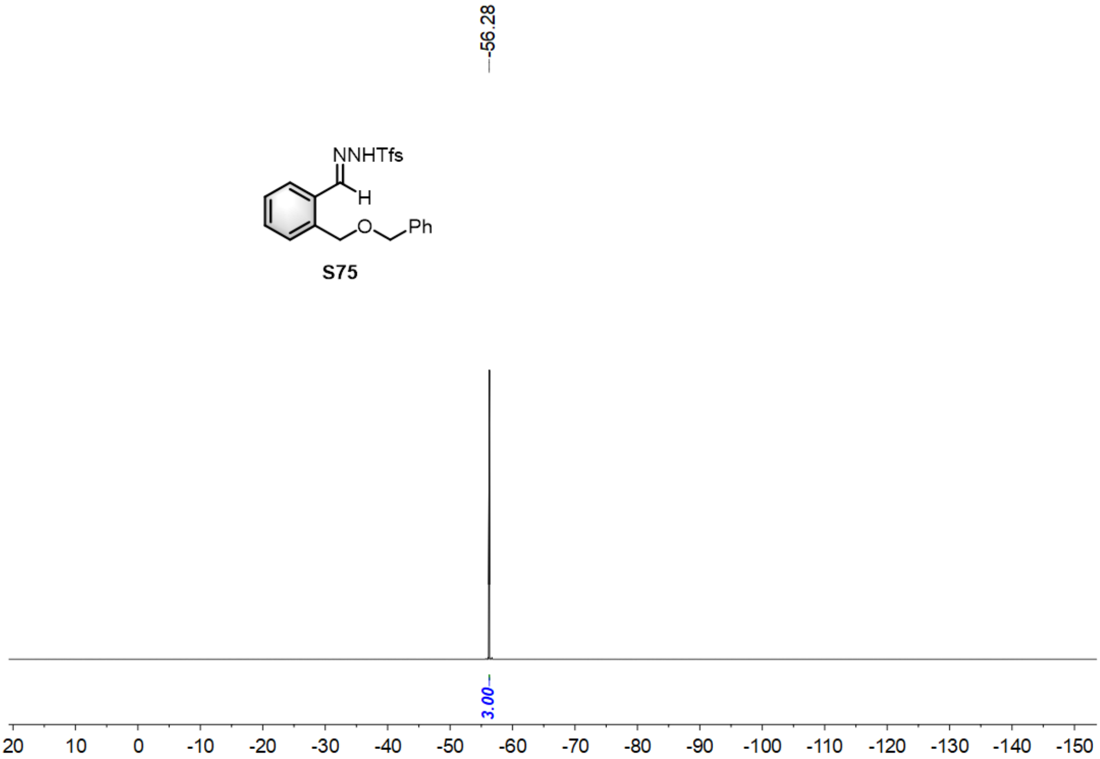

Supplementary Fig. 258 ^{19}F NMR (471 MHz, DMSO) spectrum of compound **S75**

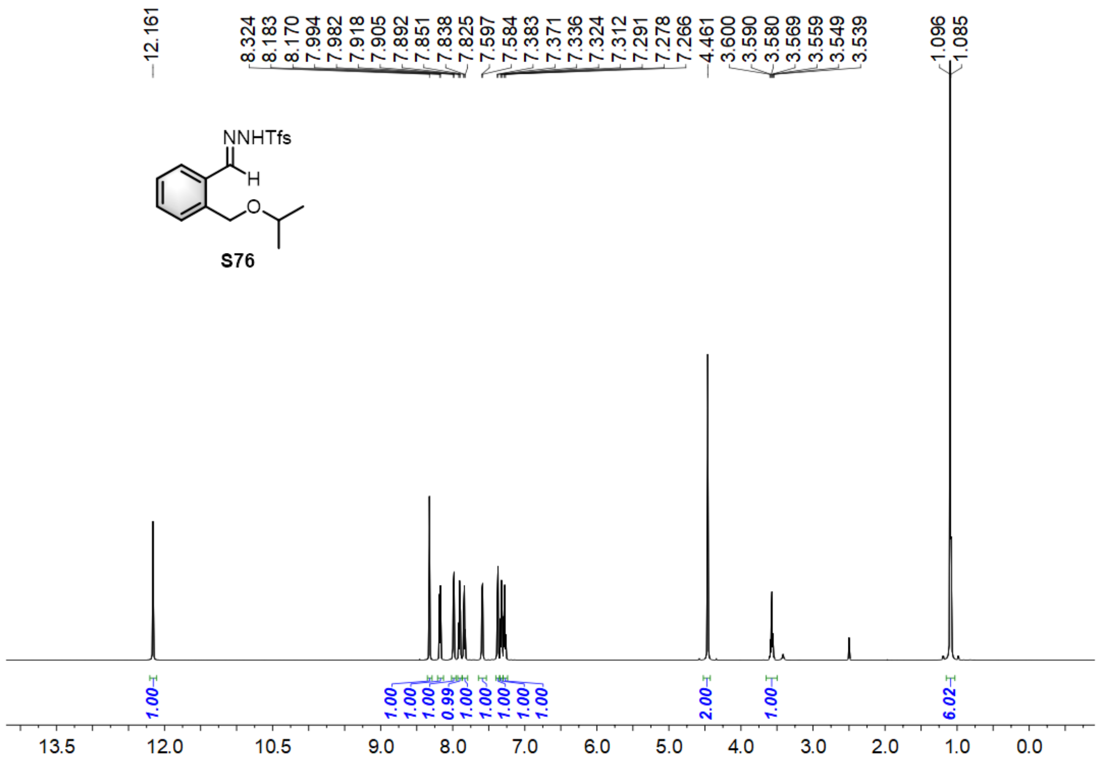

Supplementary Fig. 259 ^{1}**H NMR** (600 MHz, DMSO) spectrum of compound **S76**

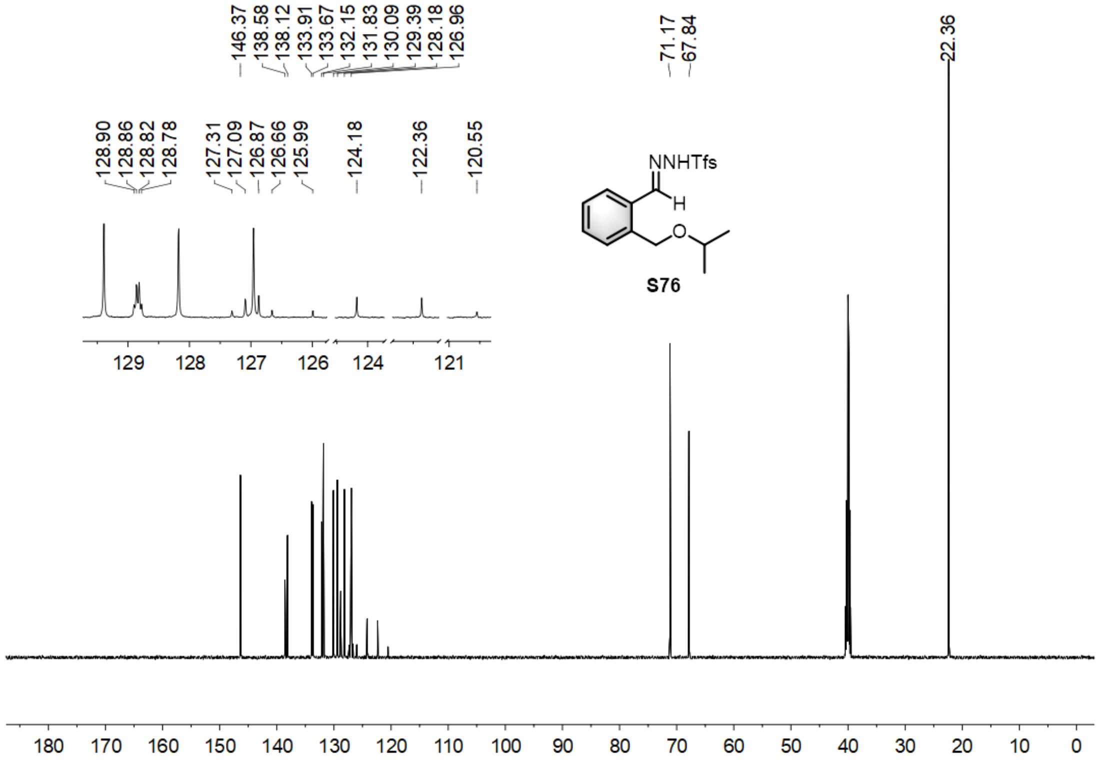

Supplementary Fig. 260 ^{13}**C NMR** (151 MHz, DMSO) spectrum of compound **S76**

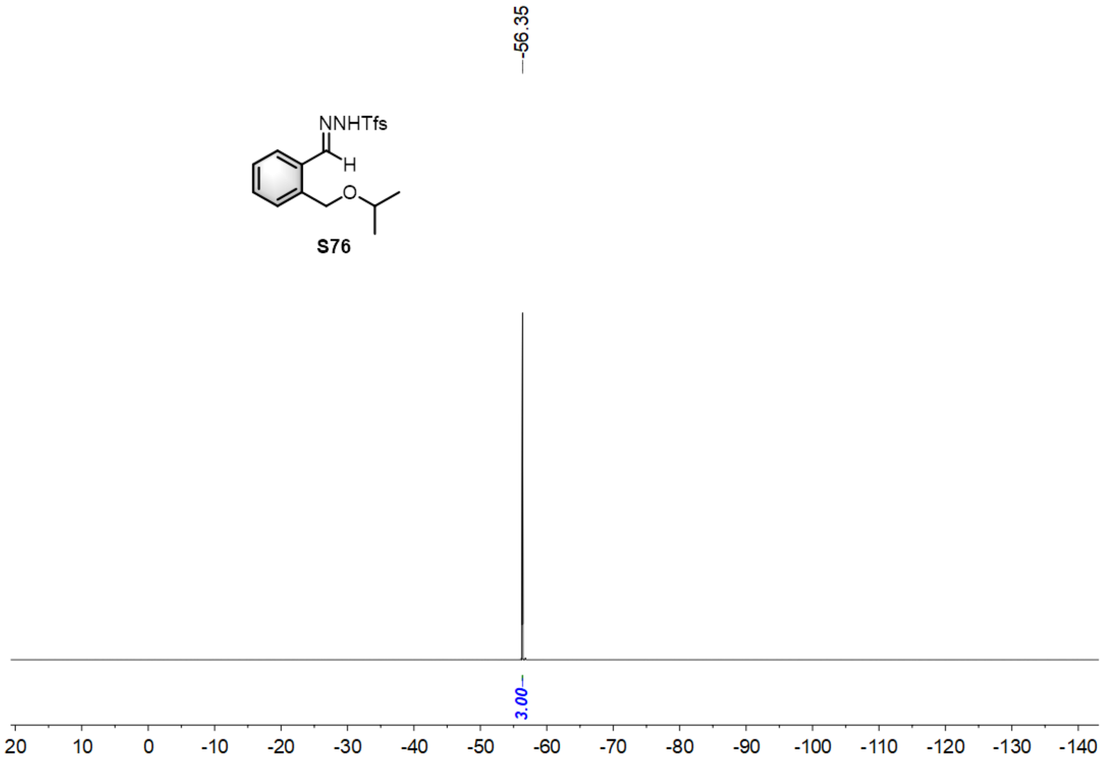

Supplementary Fig. 261 ^{19}F NMR (471 MHz, DMSO) spectrum of compound **S76**

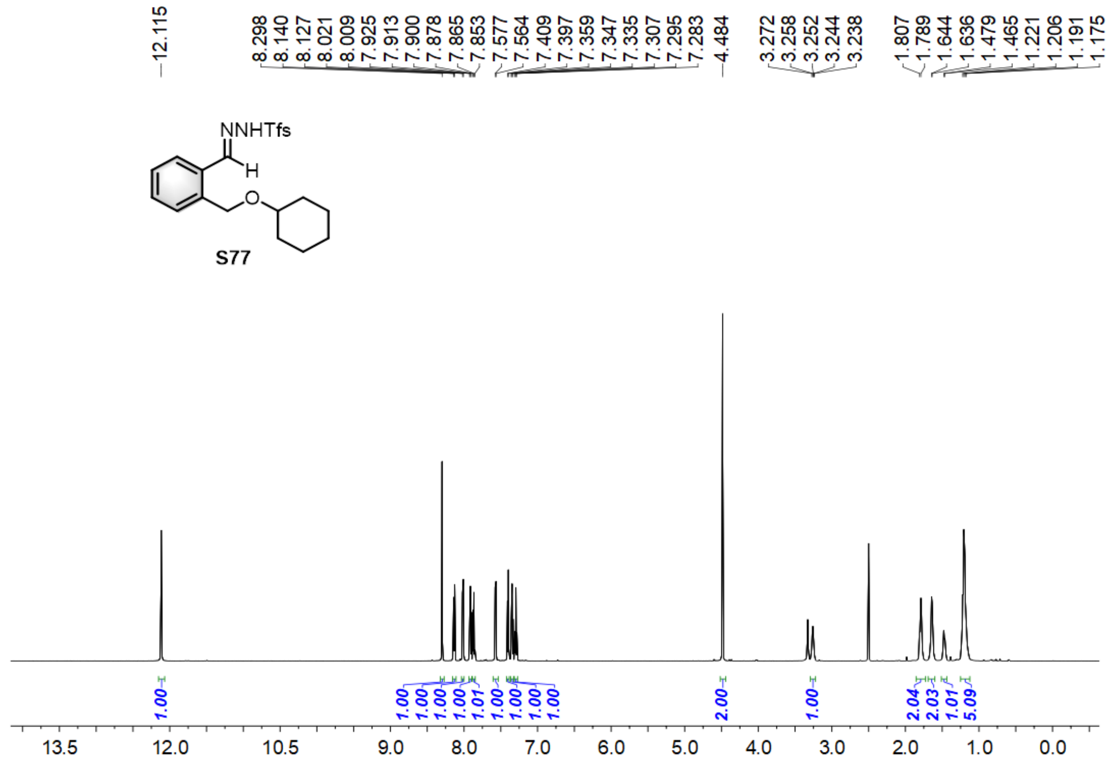

Supplementary Fig. 262 ^{1}H NMR (600 MHz, DMSO) spectrum of compound **S77**

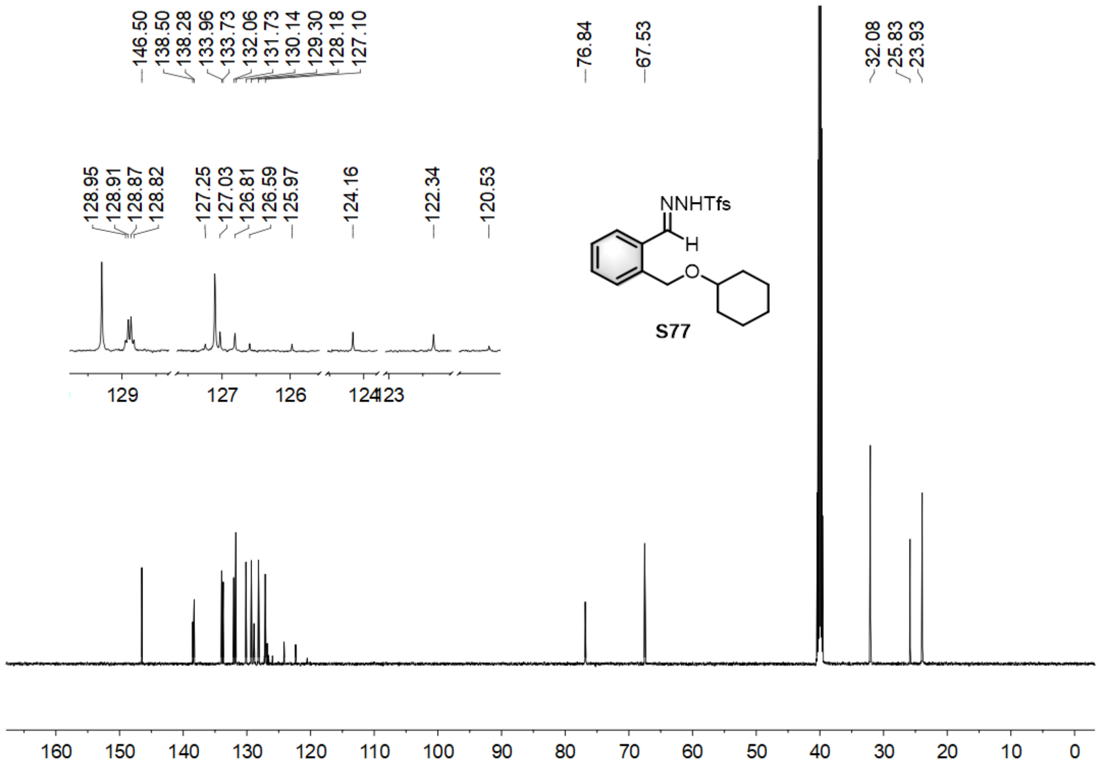

Supplementary Fig. 263 ^{13}**C NMR** (151 MHz, DMSO) spectrum of compound **S77**

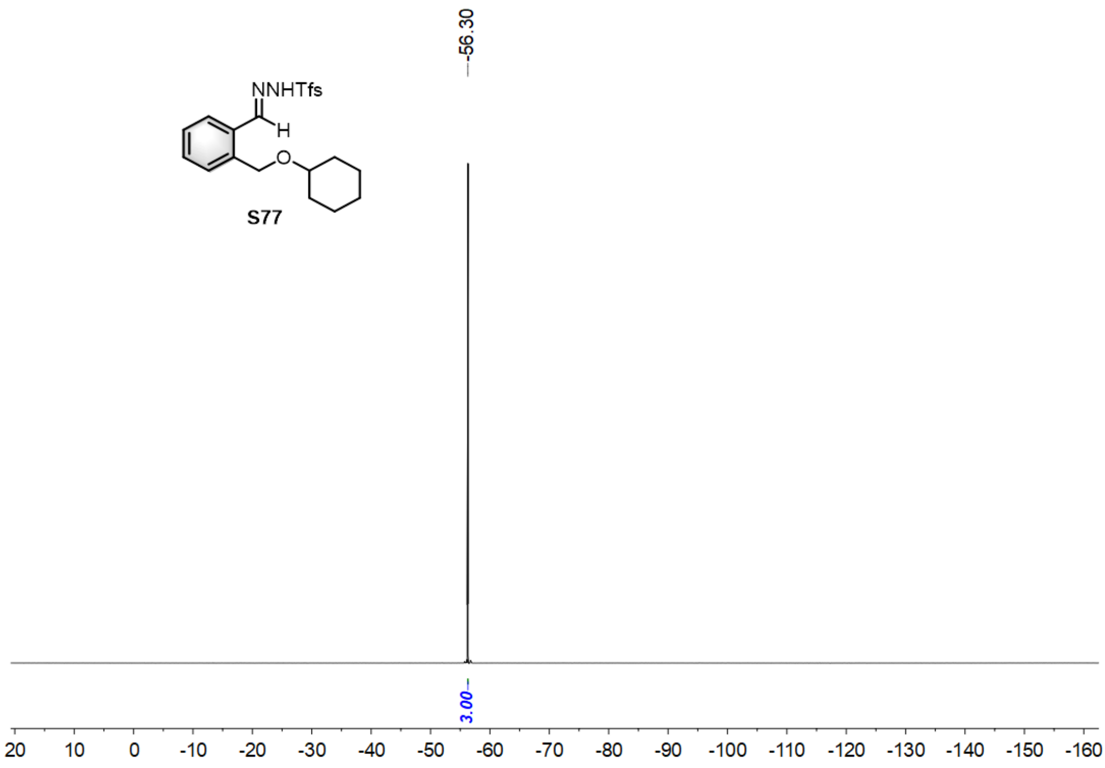

Supplementary Fig. 264 ^{19}**F NMR** (471 MHz, DMSO) spectrum of compound **S77**

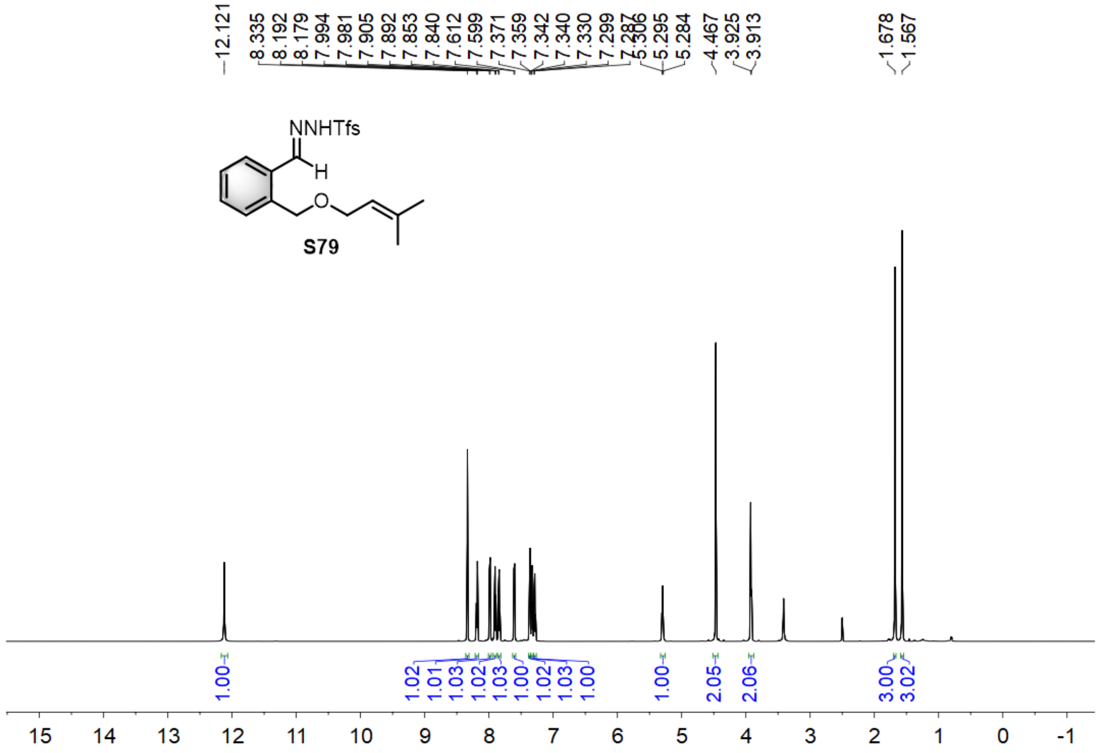

Supplementary Fig. 265 **^{1}H NMR** (600 MHz, DMSO) spectrum of compound **S79**

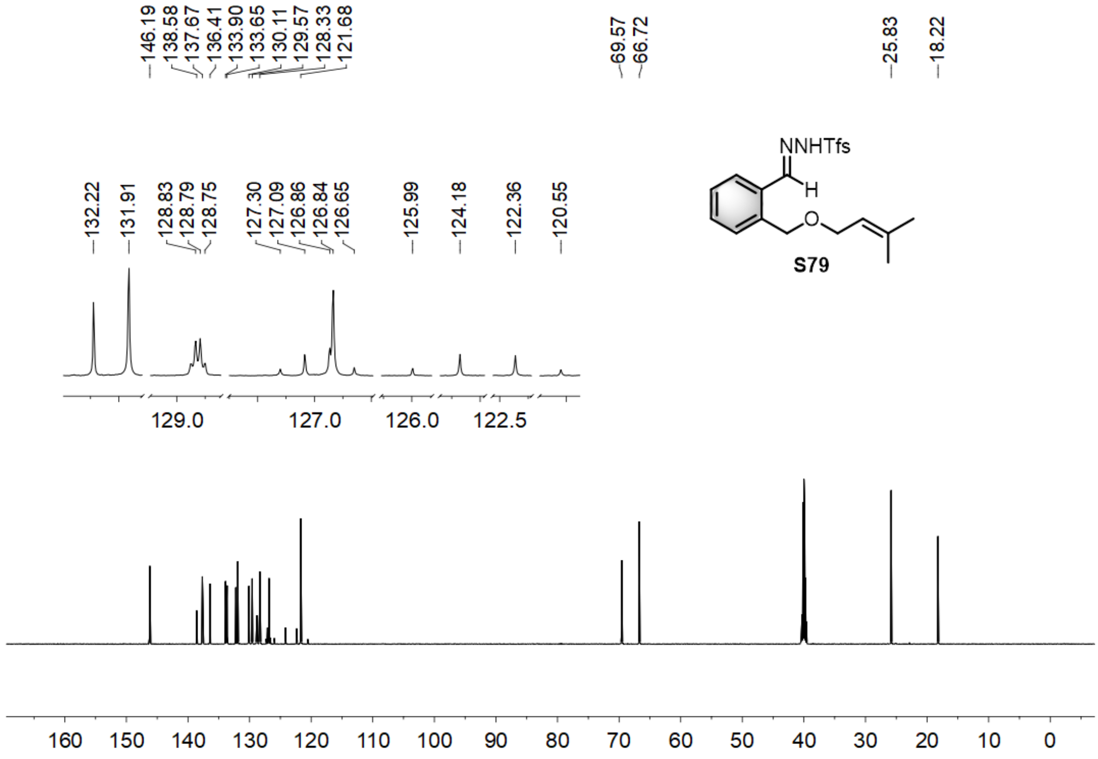

Supplementary Fig. 266 ^{13}C NMR (151 MHz, DMSO) spectrum of compound **S79**

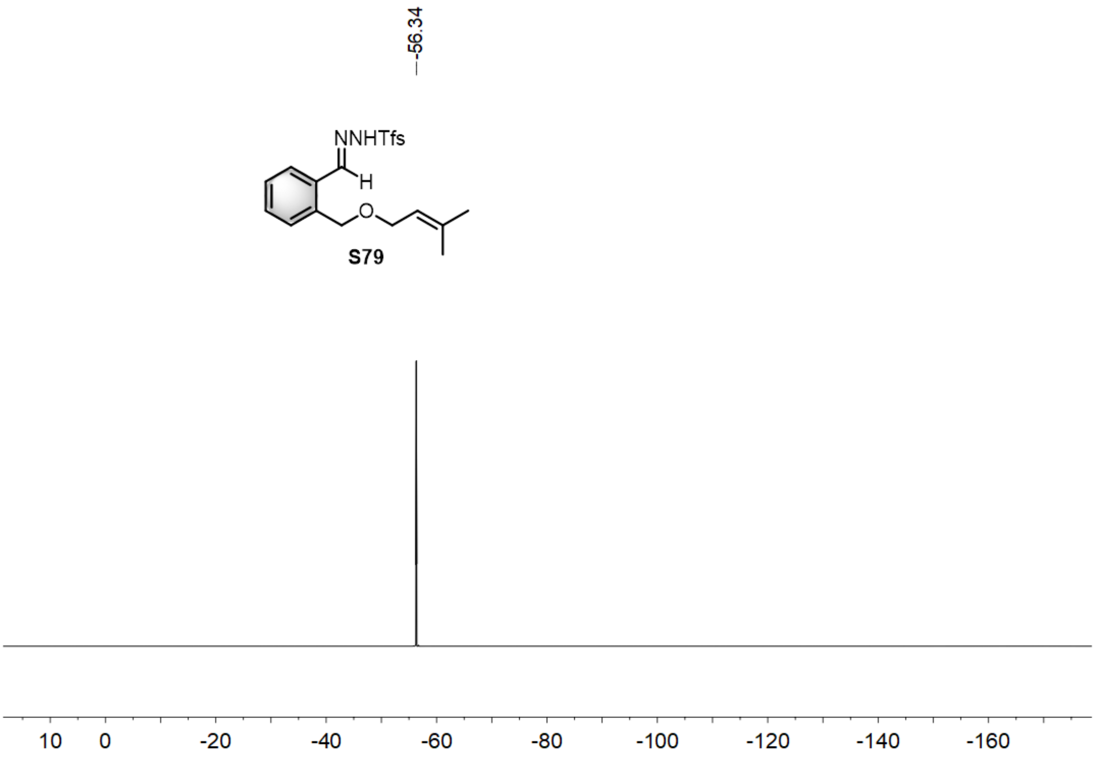

Supplementary Fig. 267 ^{19}F NMR (471 MHz, DMSO) spectrum of compound **S79**

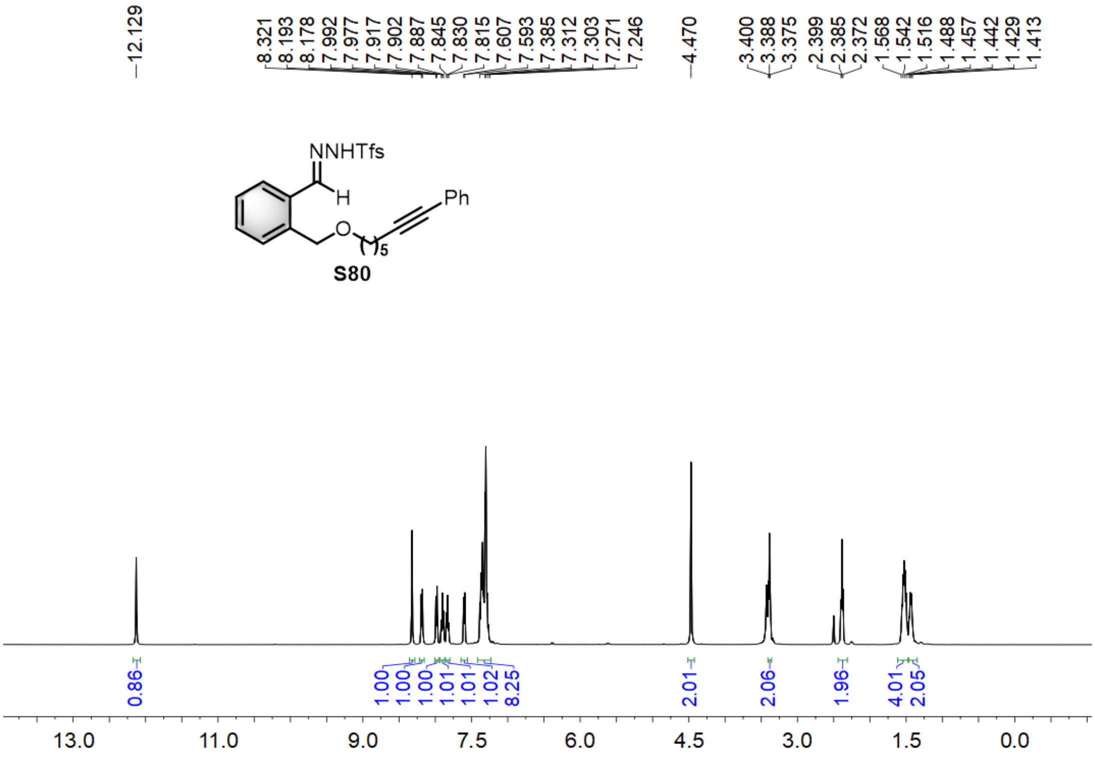

Supplementary Fig. 268 ^{1}H NMR (500 MHz, DMSO) spectrum of compound **S80**

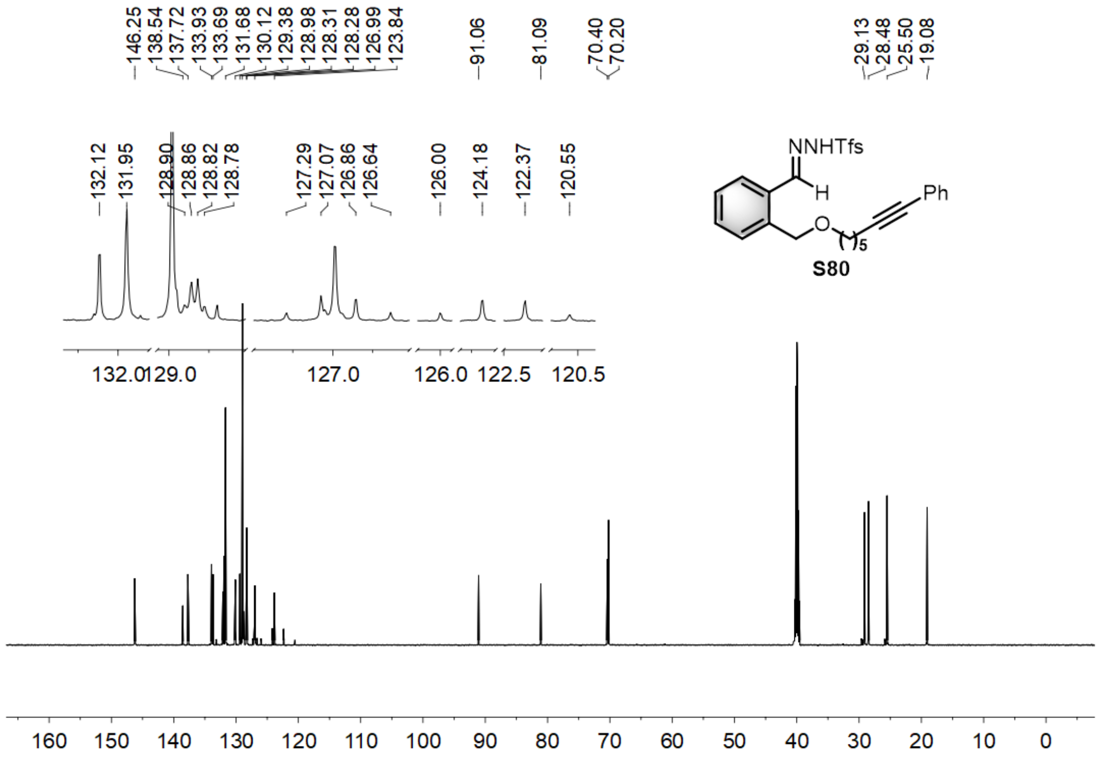

Supplementary Fig. 269 ^{13}**C NMR** (151 MHz, DMSO) spectrum of compound **S80**

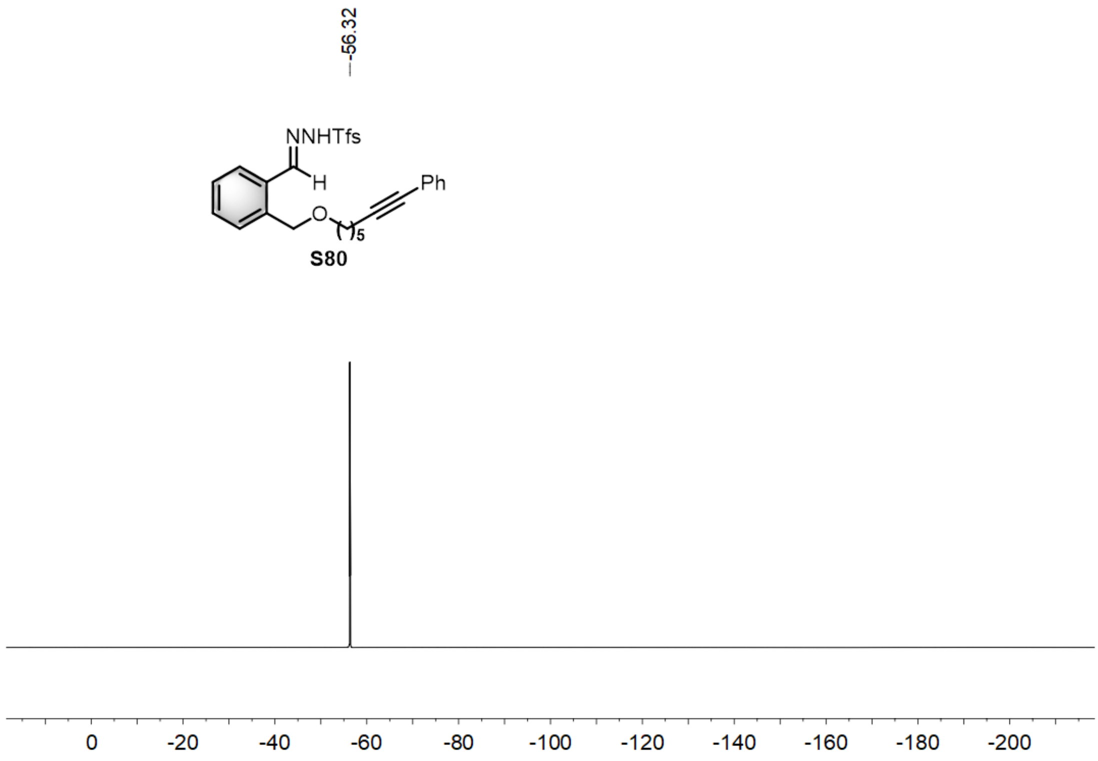

Supplementary Fig. 270 ^{19}**F NMR** (471 MHz, DMSO) spectrum of compound **S80**

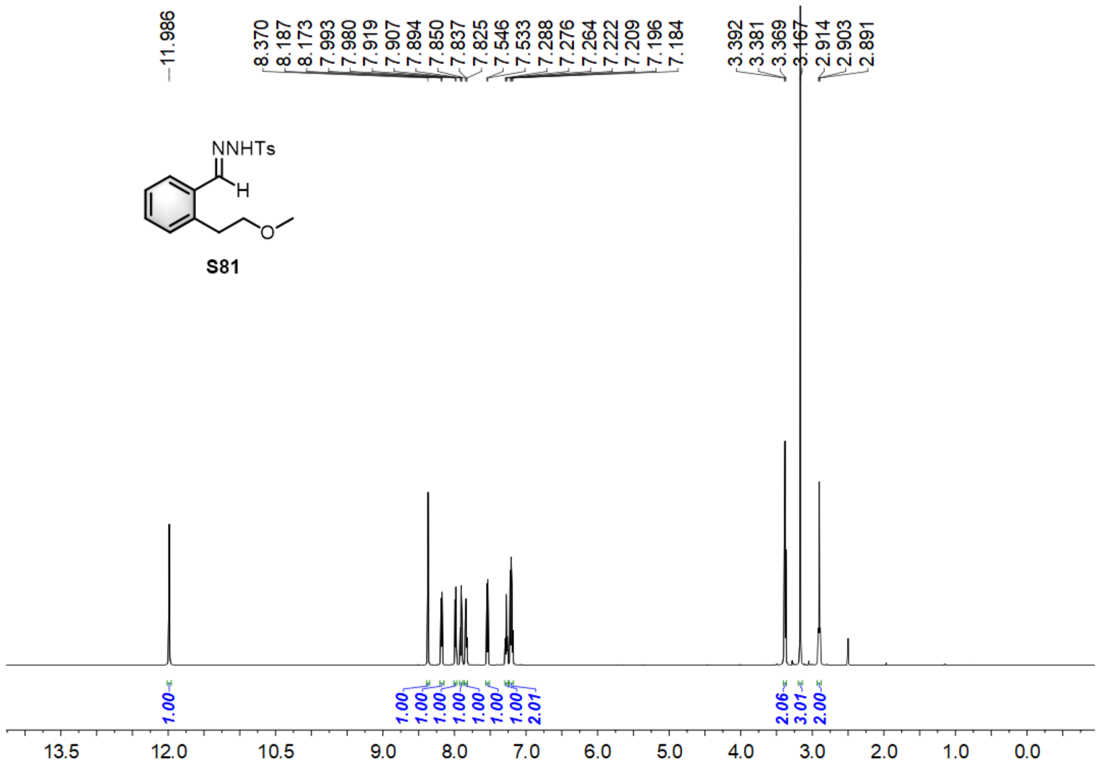

Supplementary Fig. 271 ^{1}**H NMR** (600 MHz, DMSO) spectrum of compound **S81**

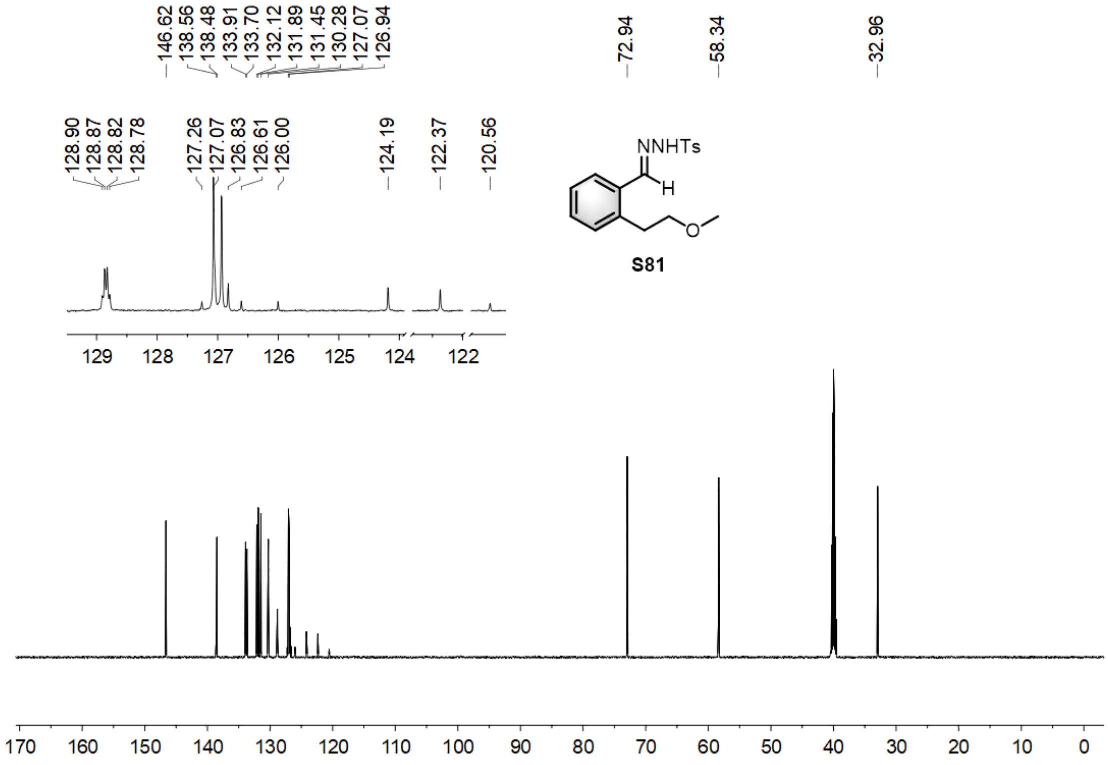

Supplementary Fig. 272 ^{13}**C NMR** (151 MHz, DMSO) spectrum of compound **S81**

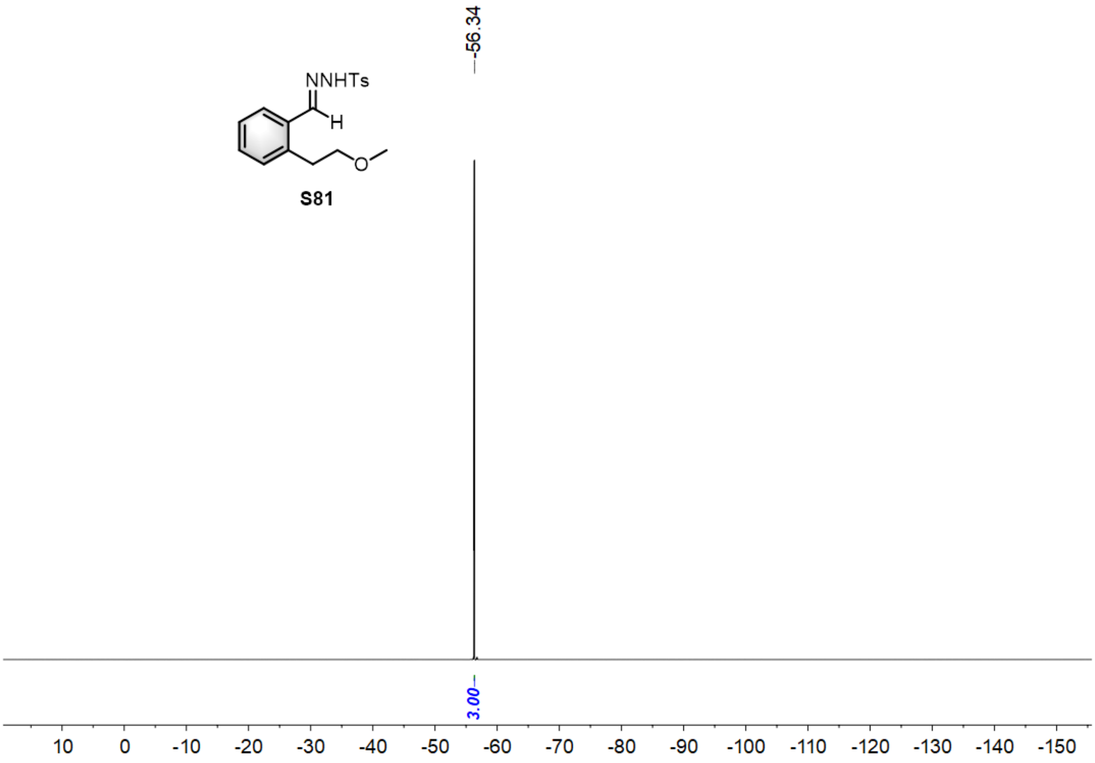

Supplementary Fig. 273 ^{19}F NMR (471 MHz, DMSO) spectrum of compound **S81**

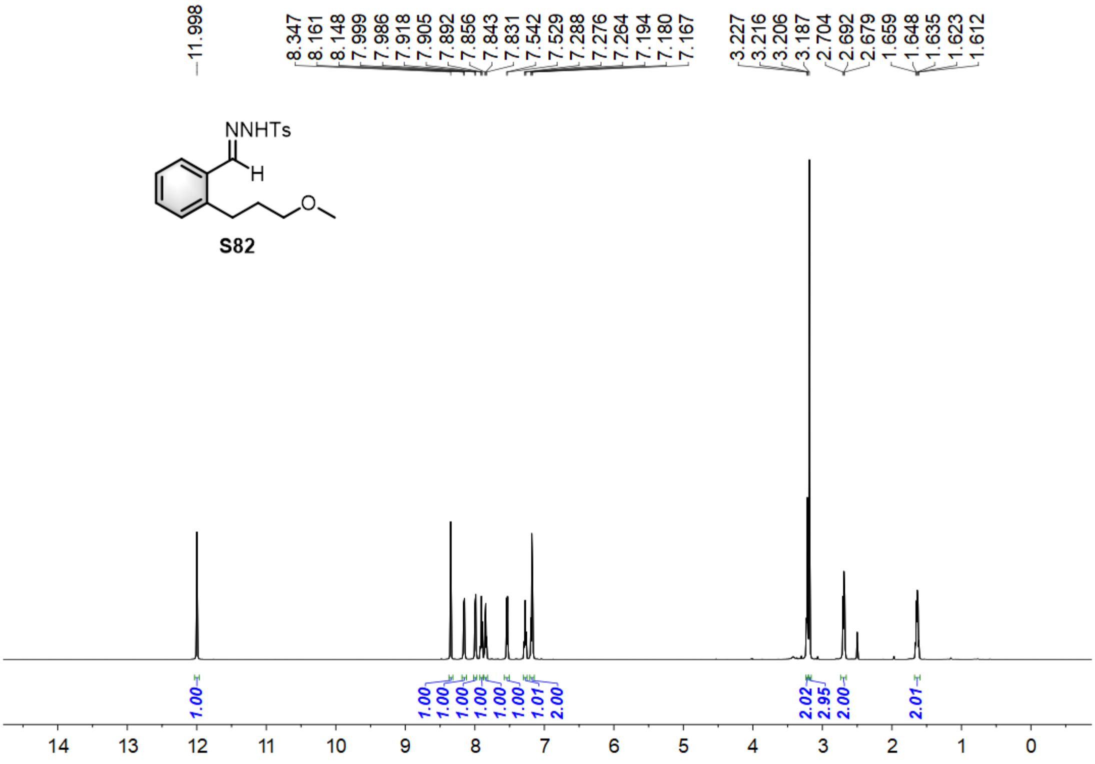

Supplementary Fig. 274 ^{1}H NMR (600 MHz, DMSO) spectrum of compound **S82**

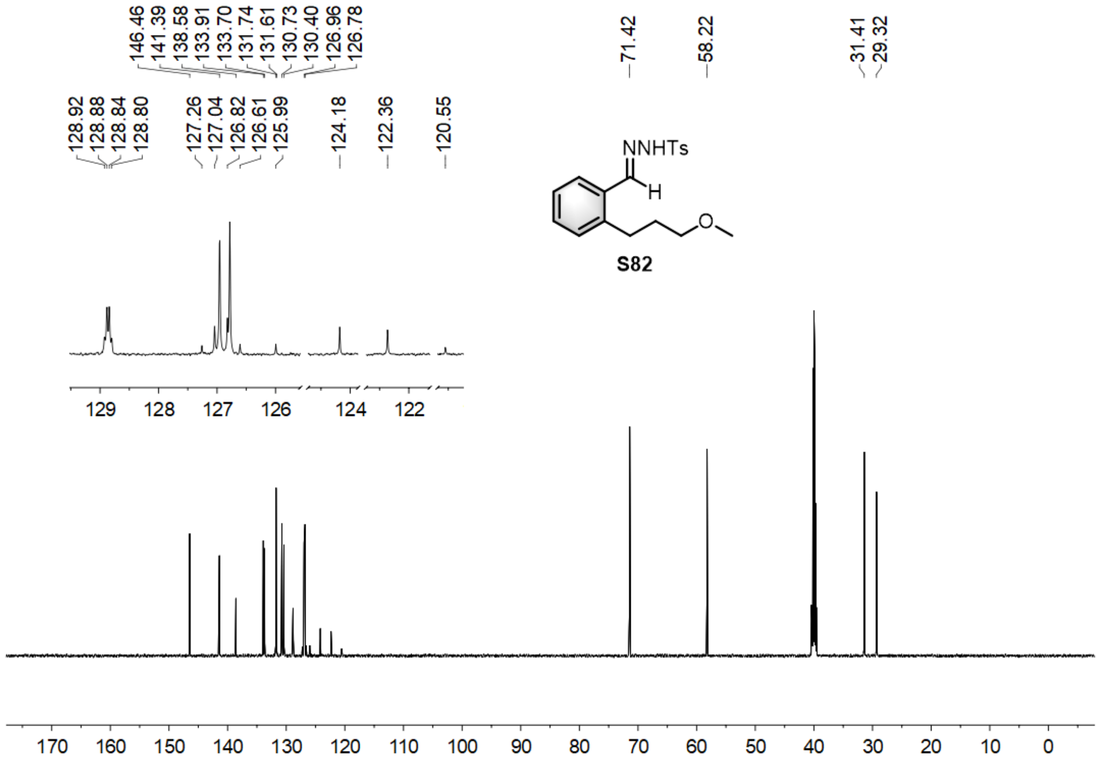

Supplementary Fig. 275 ^{13}C NMR (151 MHz, DMSO) spectrum of compound **S82**

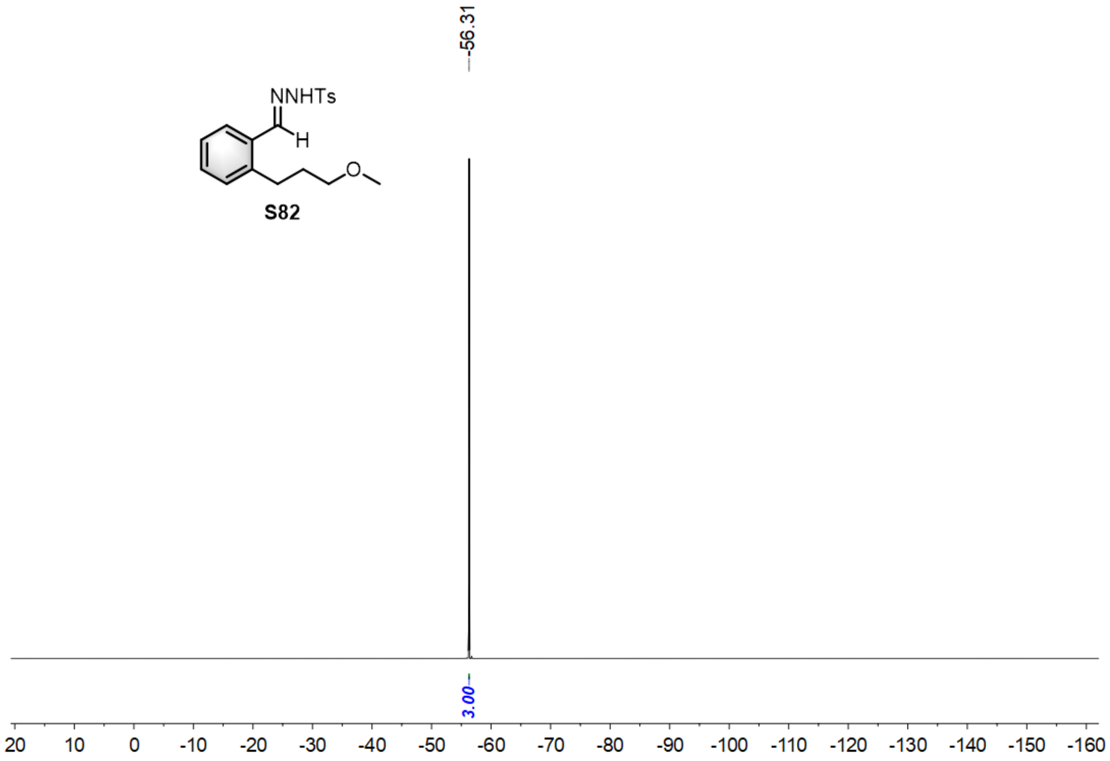

Supplementary Fig. 276 ^{19}F NMR (471 MHz, DMSO) spectrum of compound **82**

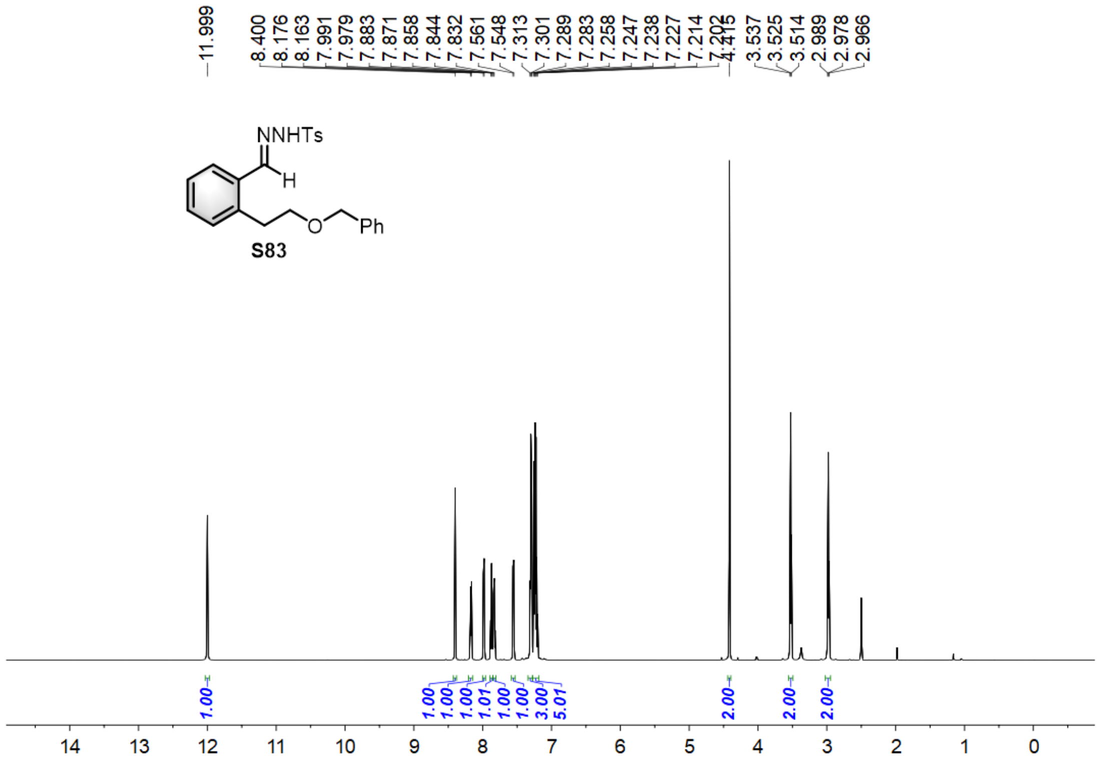

Supplementary Fig. 277 ^{1}H NMR (600 MHz, DMSO) spectrum of compound **S83**

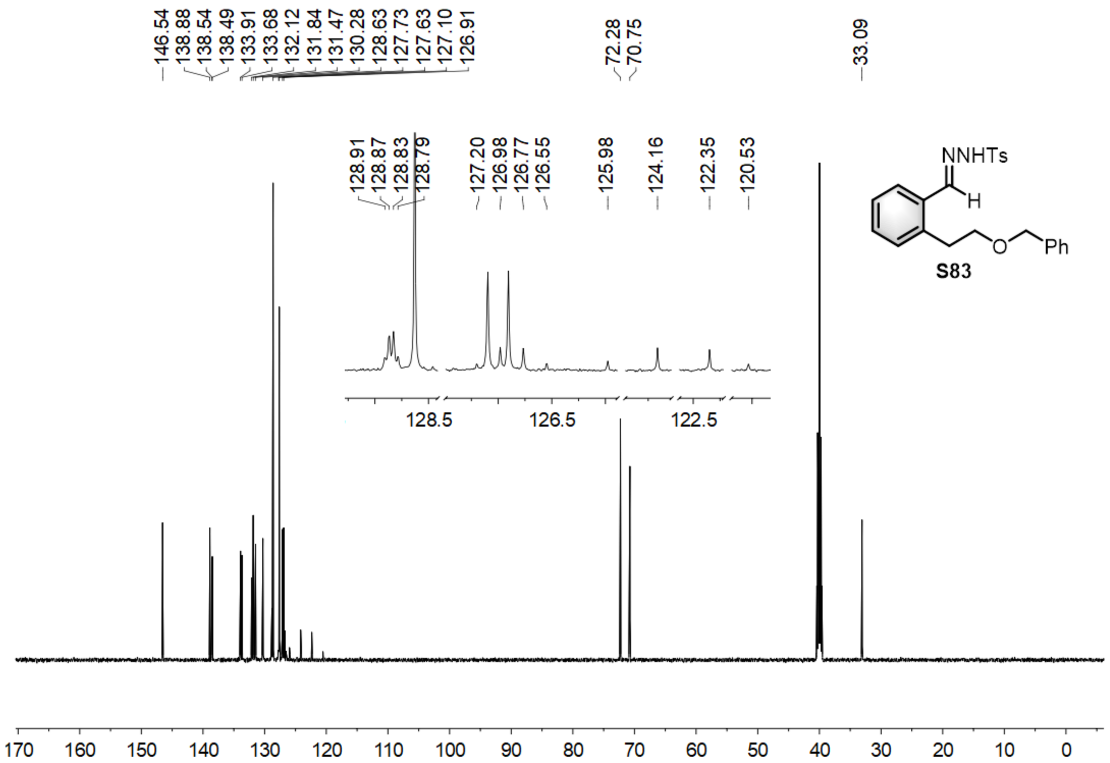

Supplementary Fig. 278 ^{13}C NMR (151 MHz, DMSO) spectrum of compound **S83**

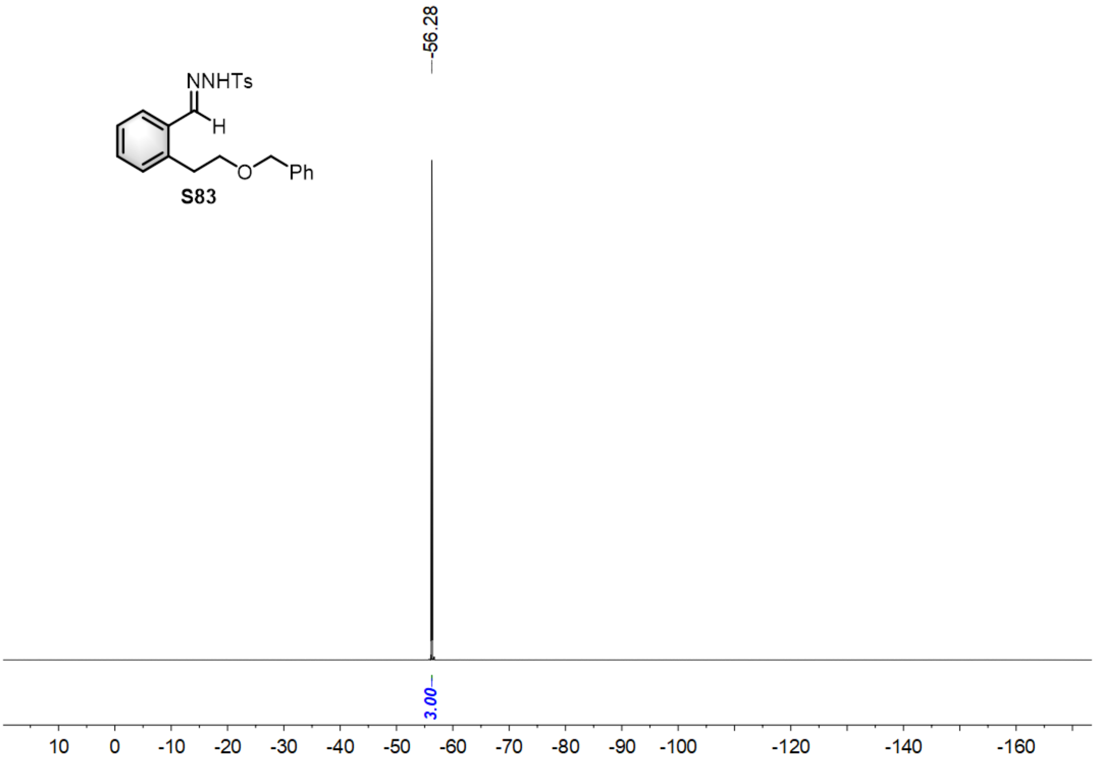

Supplementary Fig. 279 ^{19}**F NMR** (471 MHz, DMSO) spectrum of compound **S83**

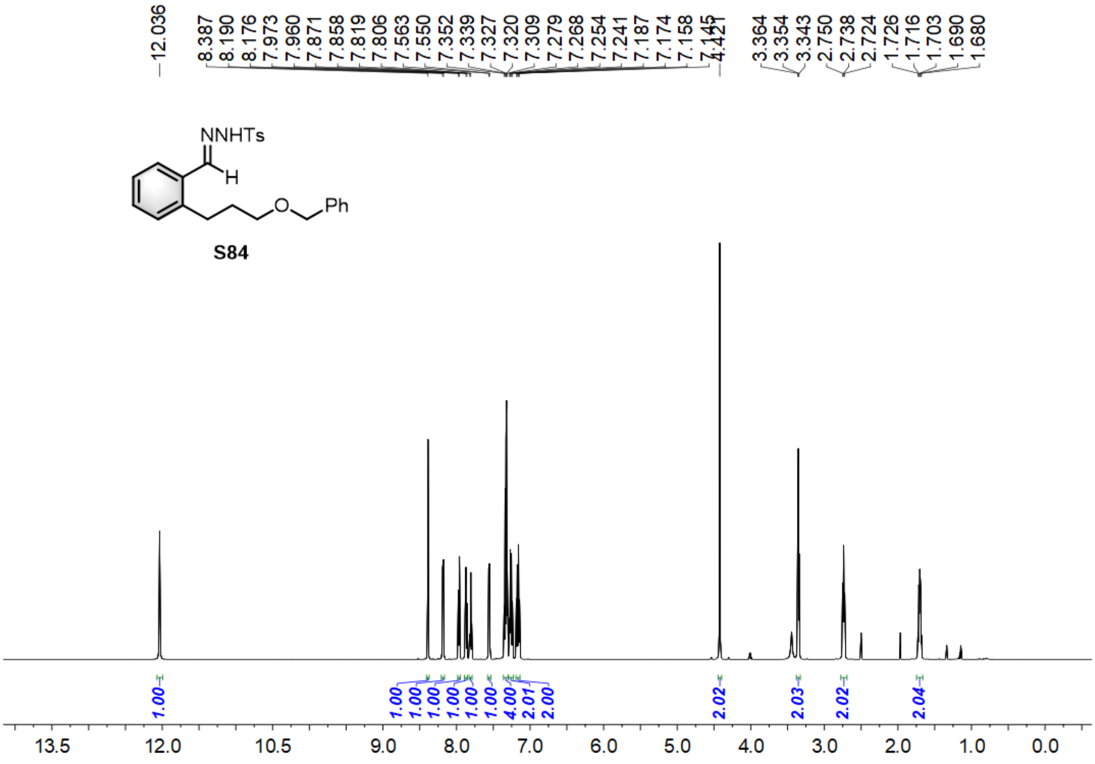

Supplementary Fig. 280 ^{1}**H NMR** (600 MHz, DMSO) spectrum of compound **S84**

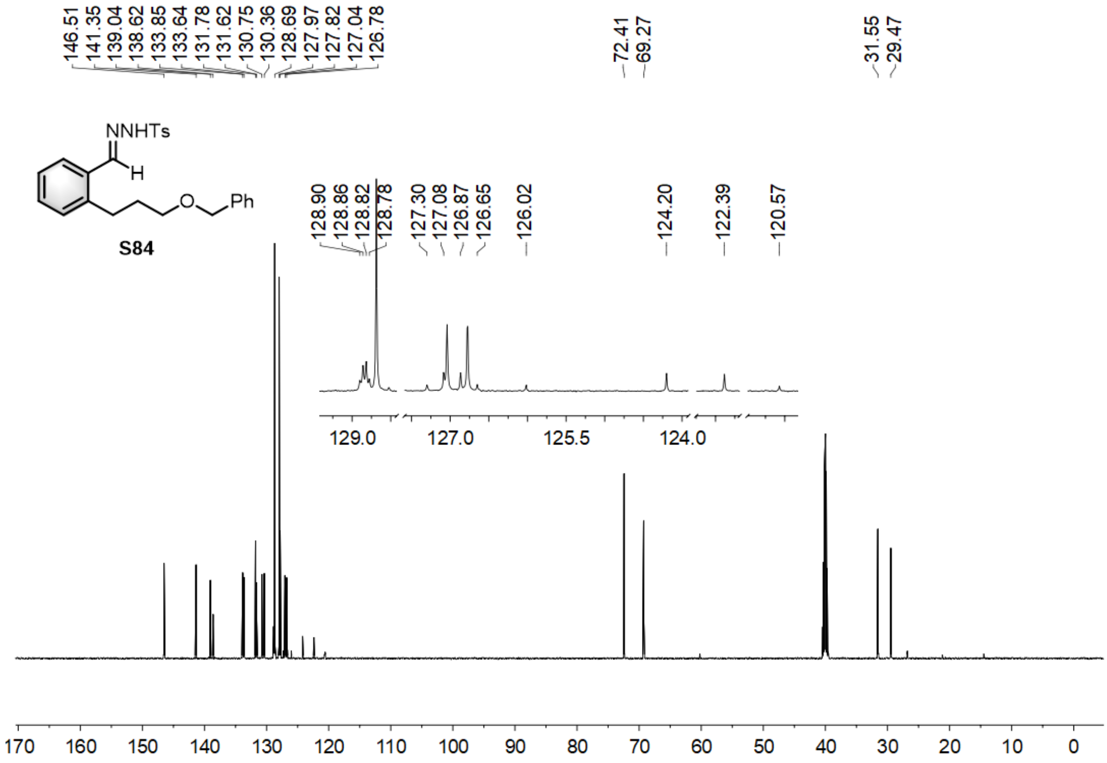

Supplementary Fig. 281 ^{13}C NMR (151 MHz, DMSO) spectrum of compound **S84**

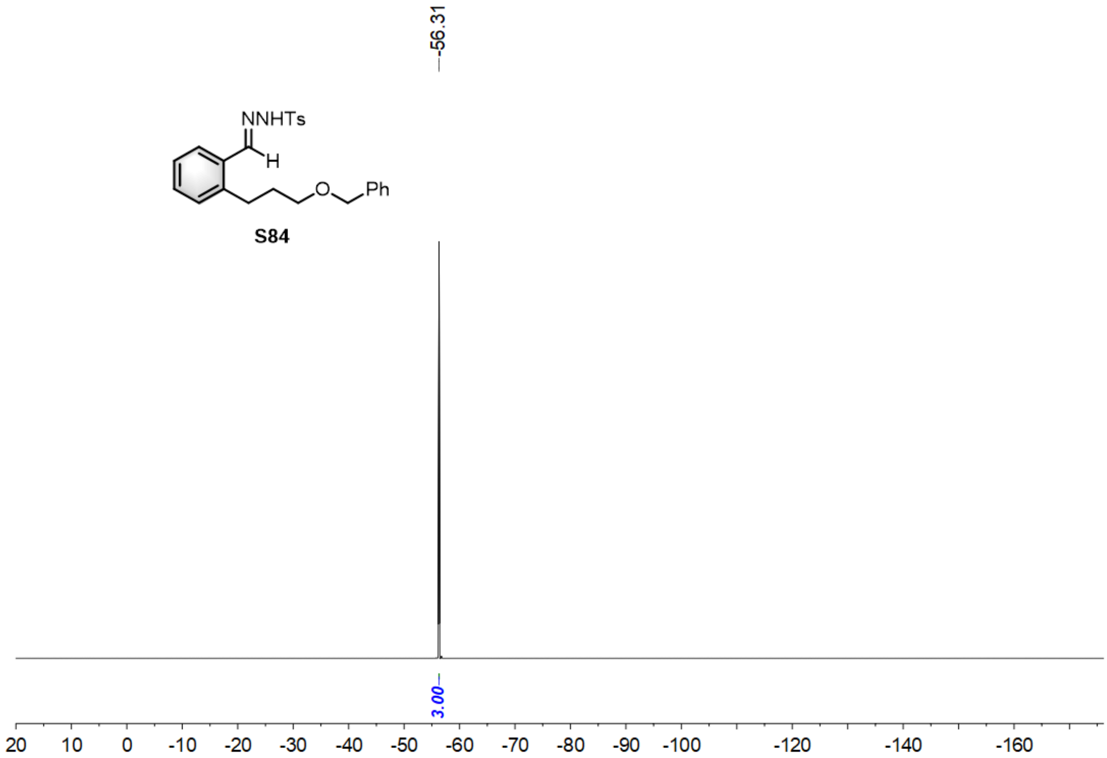

Supplementary Fig. 282 ^{19}F NMR (471 MHz, DMSO) spectrum of compound **84**

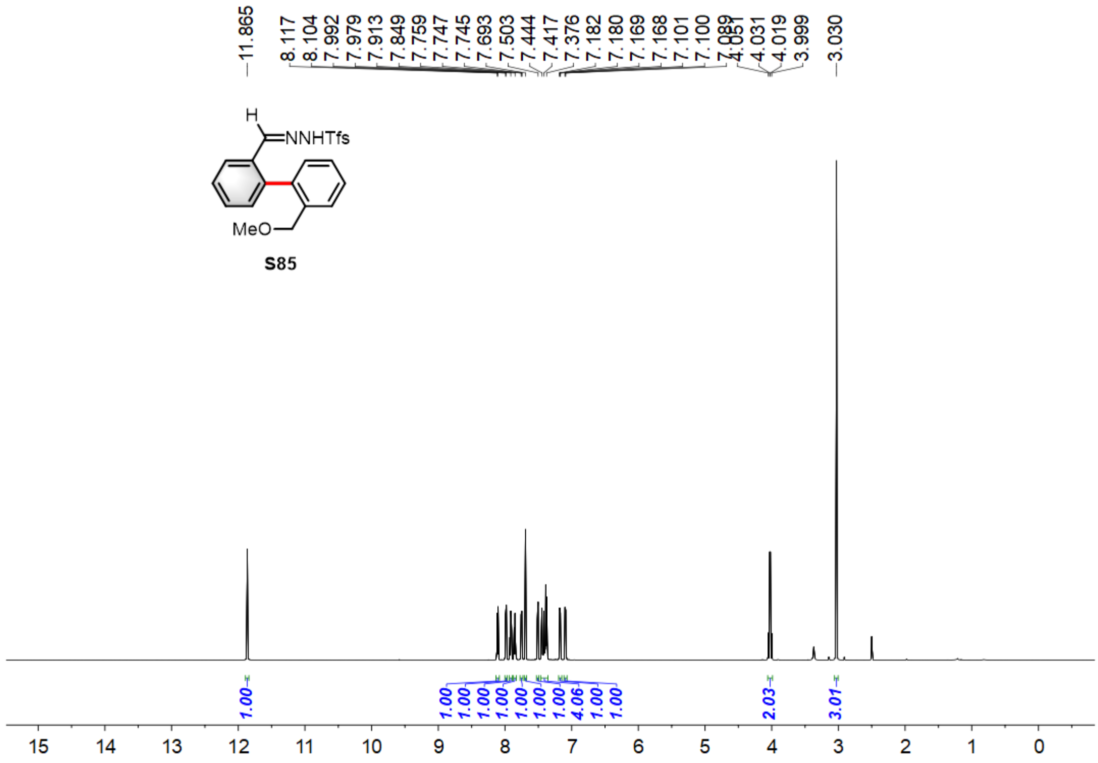

Supplementary Fig. 283 ^{1}H NMR (600 MHz, DMSO) spectrum of compound **S85**

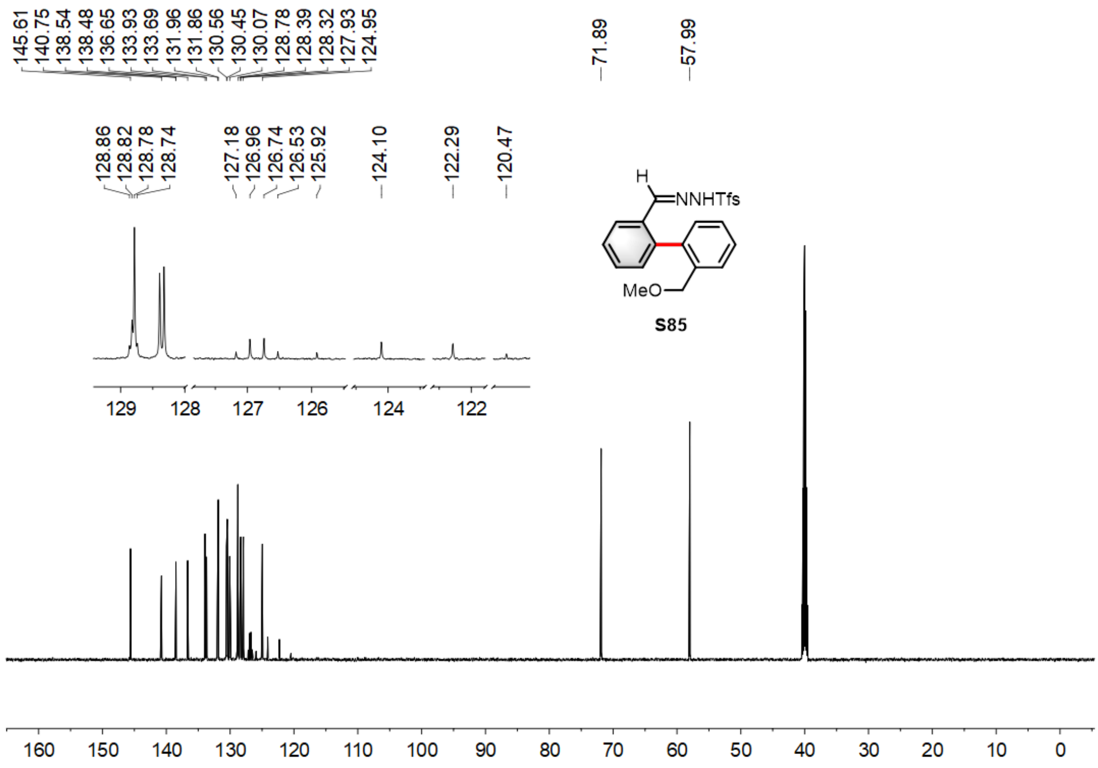

Supplementary Fig. 284 ^{13}C NMR (151 MHz, DMSO) spectrum of compound **S84**

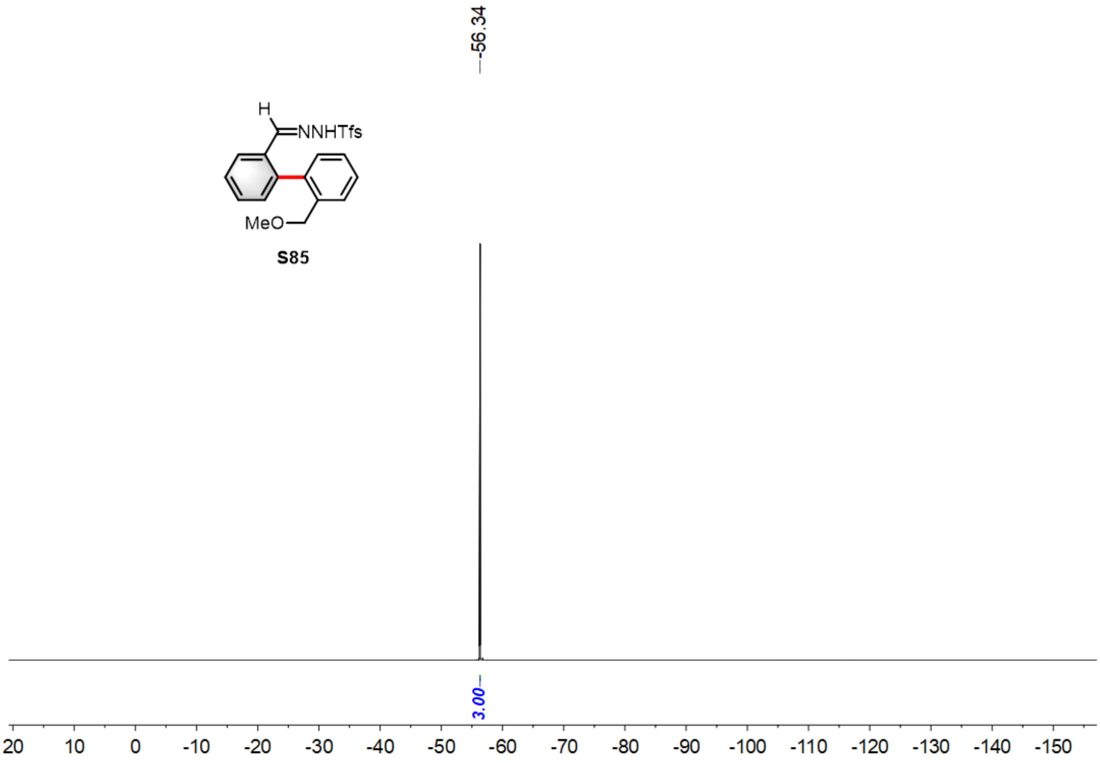

Supplementary Fig. 285 ^{19}**F NMR** (471 MHz, DMSO) spectrum of compound **S85**

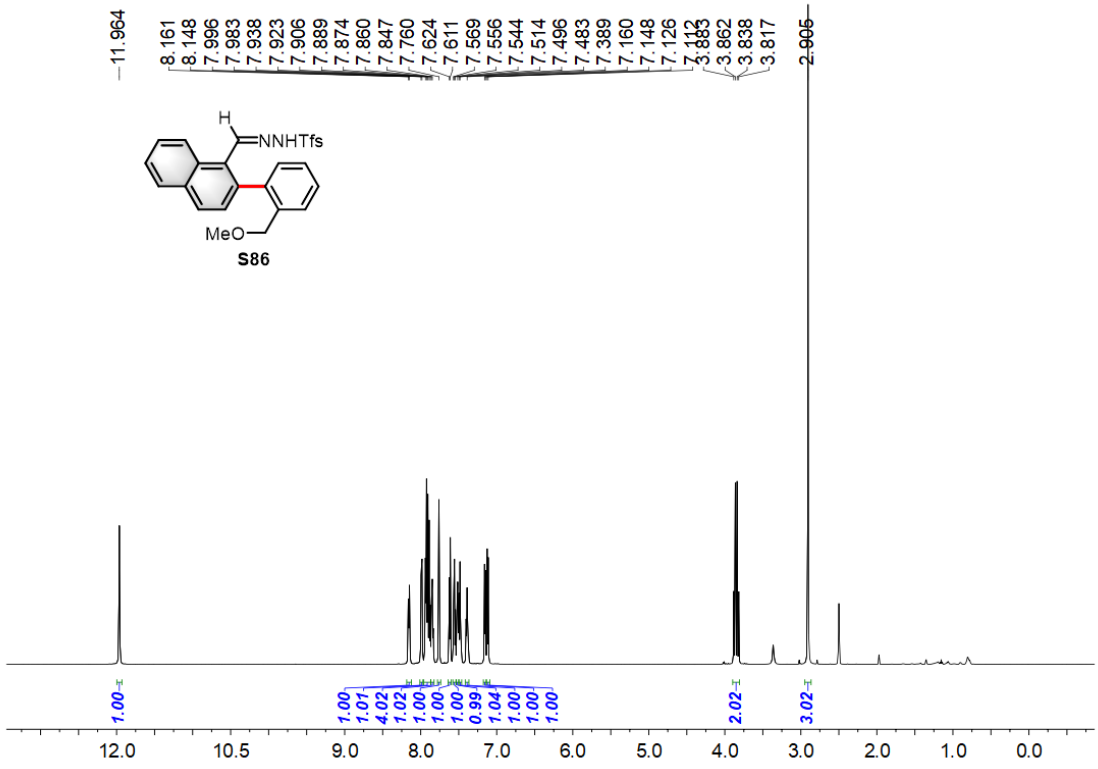

Supplementary Fig. 286 ^{1}**H NMR** (600 MHz, DMSO) spectrum of compound **86**

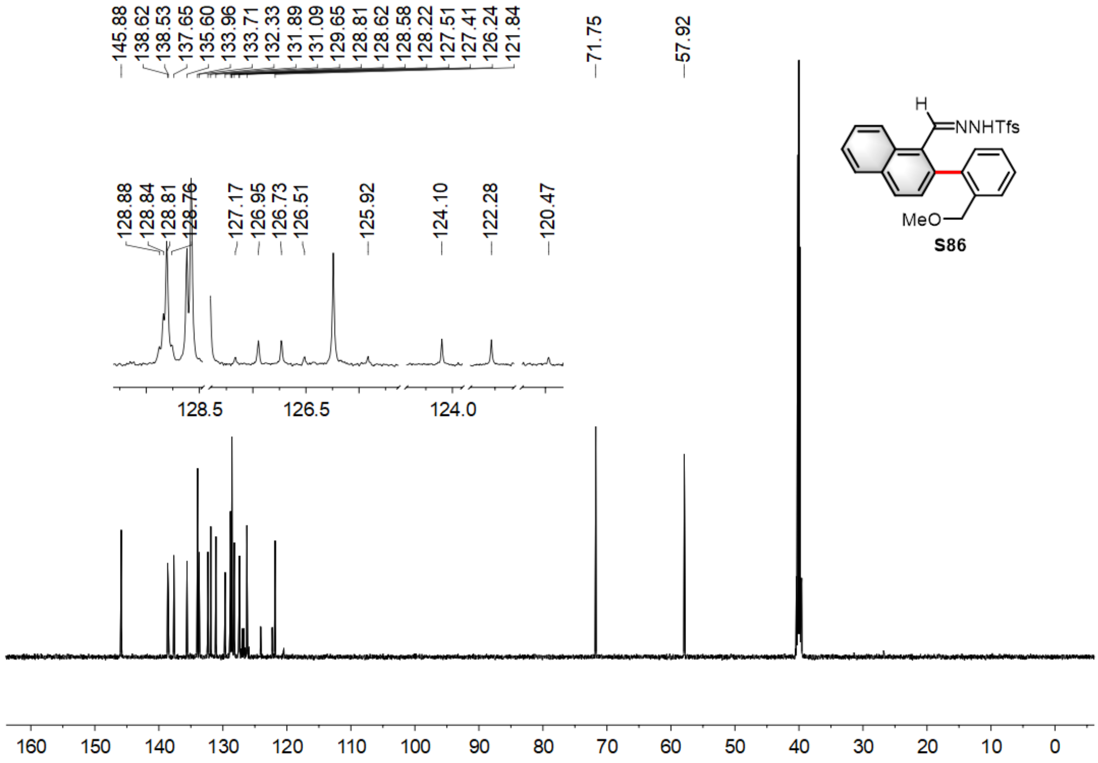

Supplementary Fig. 287 ^{13}**C NMR** (151 MHz, DMSO) spectrum of compound **S86**

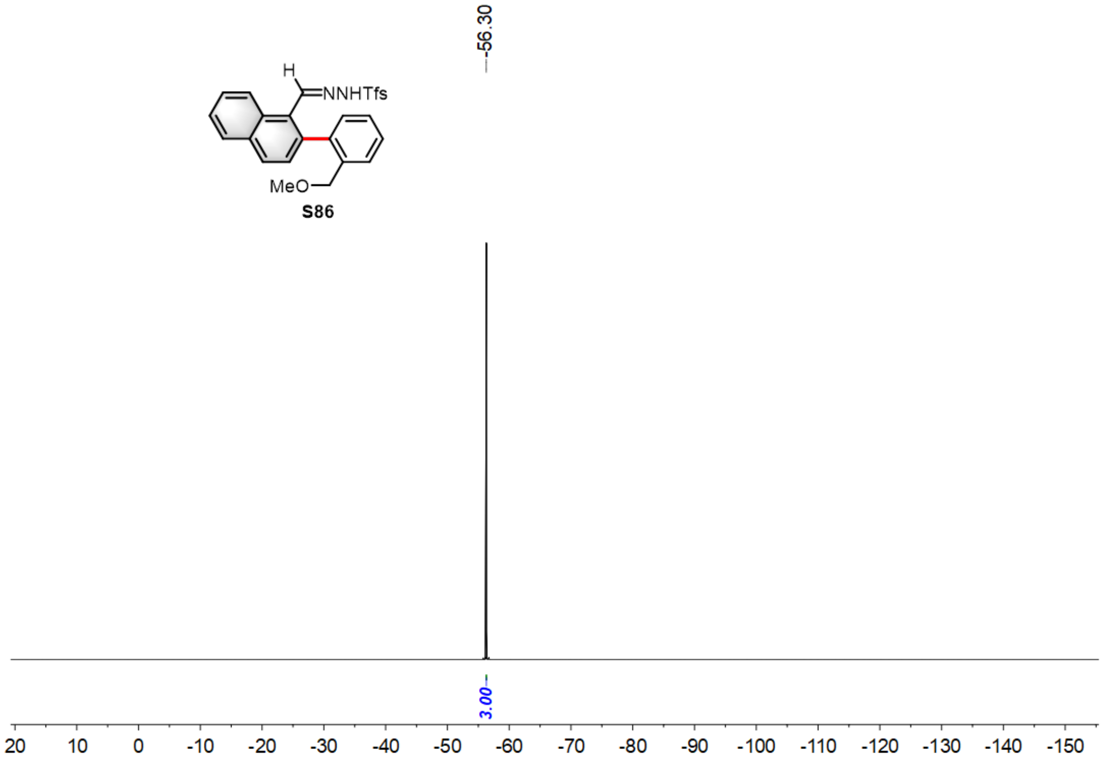

Supplementary Fig. 288 ^{19}**F NMR** (471 MHz, DMSO) spectrum of compound **S86**

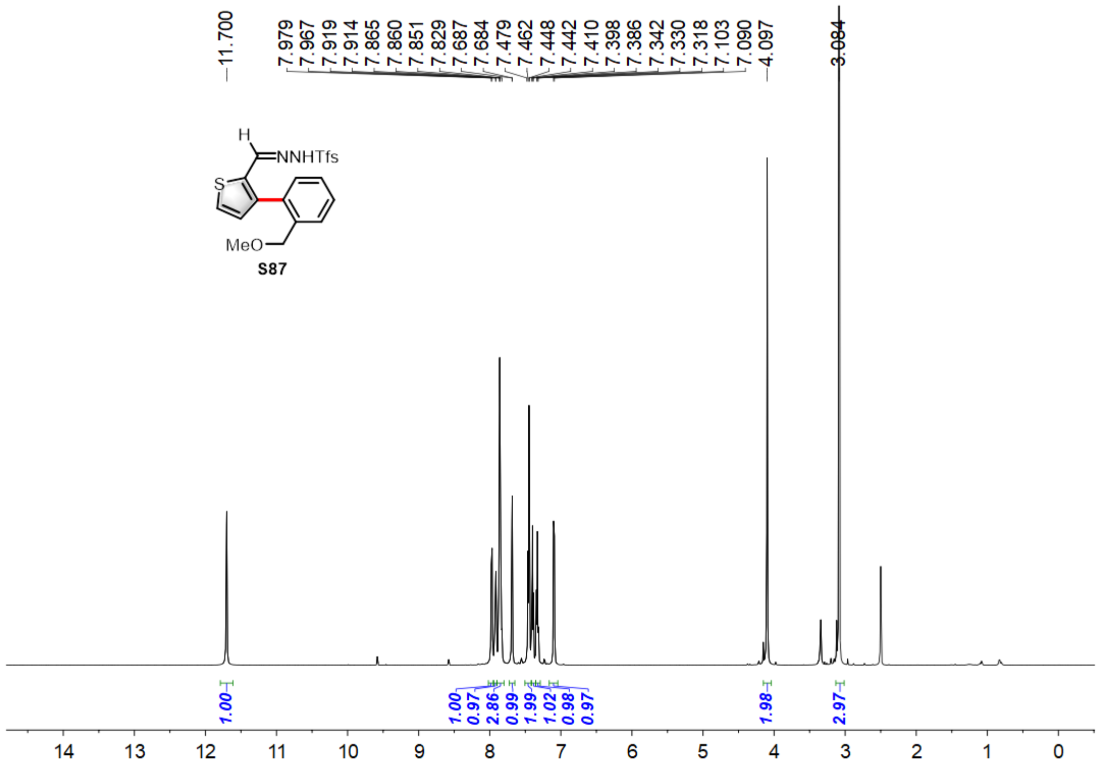

Supplementary Fig. 289 ^{1}**H NMR** (600 MHz, DMSO) spectrum of compound **S87**

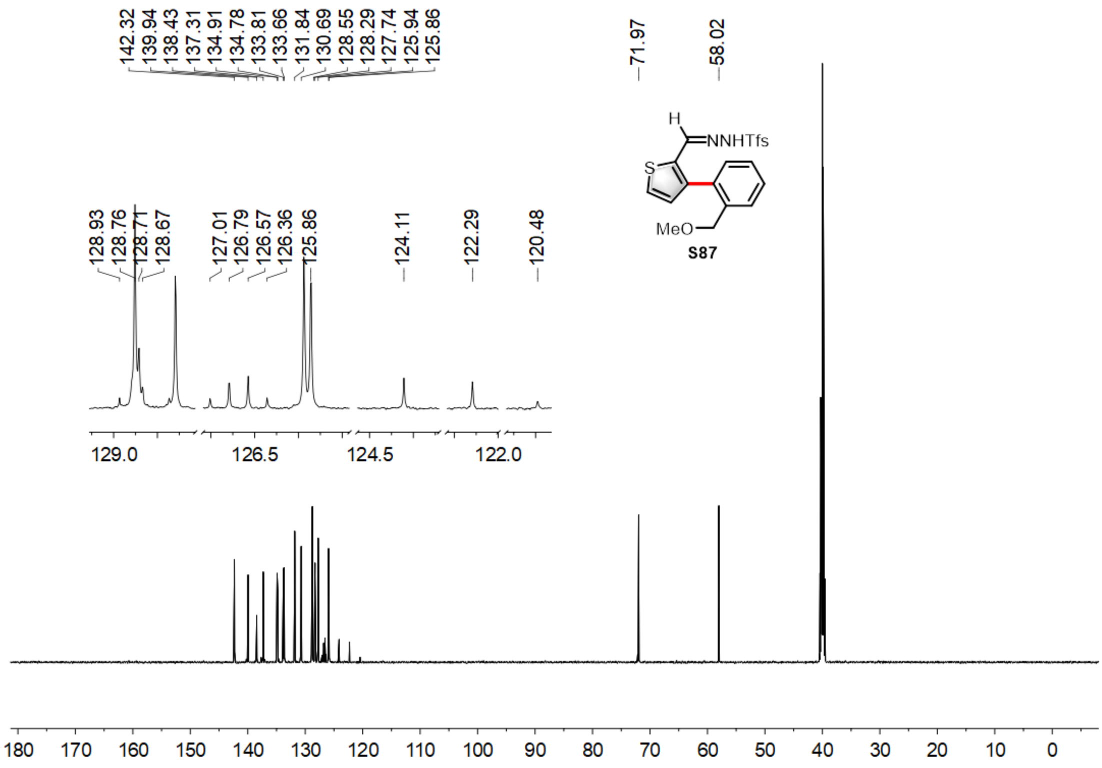

Supplementary Fig. 290 ^{13}**C NMR** (151 MHz, DMSO) spectrum of compound **S87**

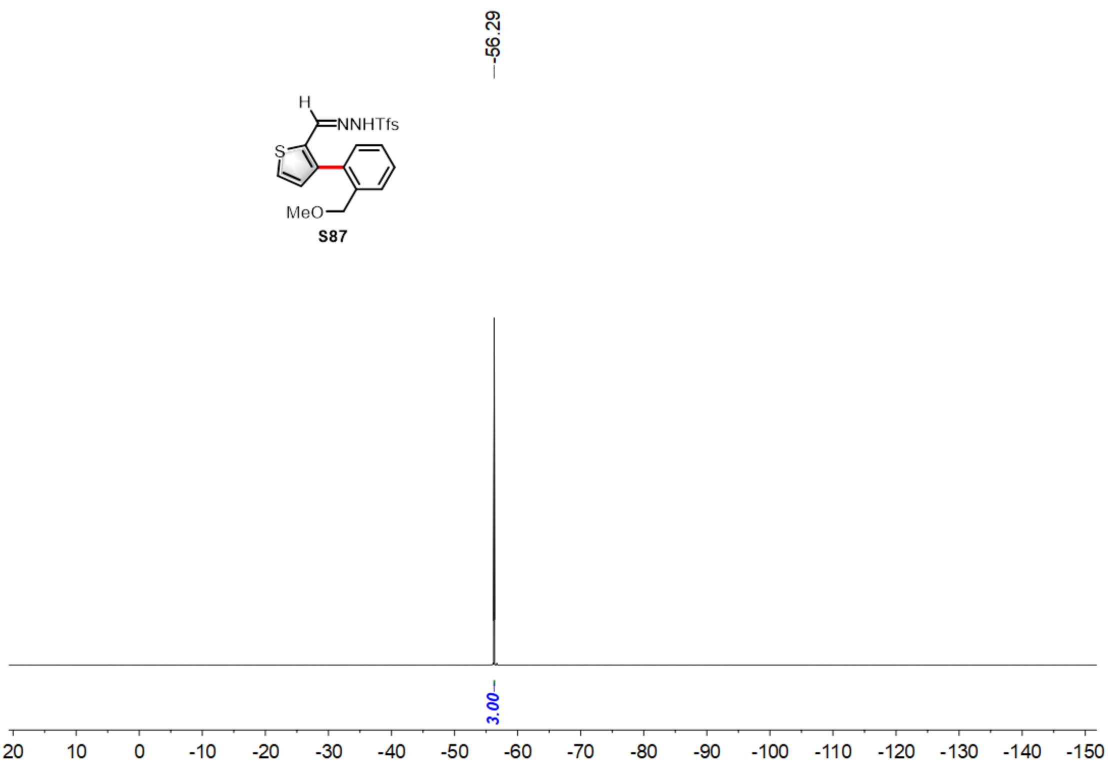

Supplementary Fig. 291 ^{19}F NMR (471 MHz, DMSO) spectrum of compound **S87**

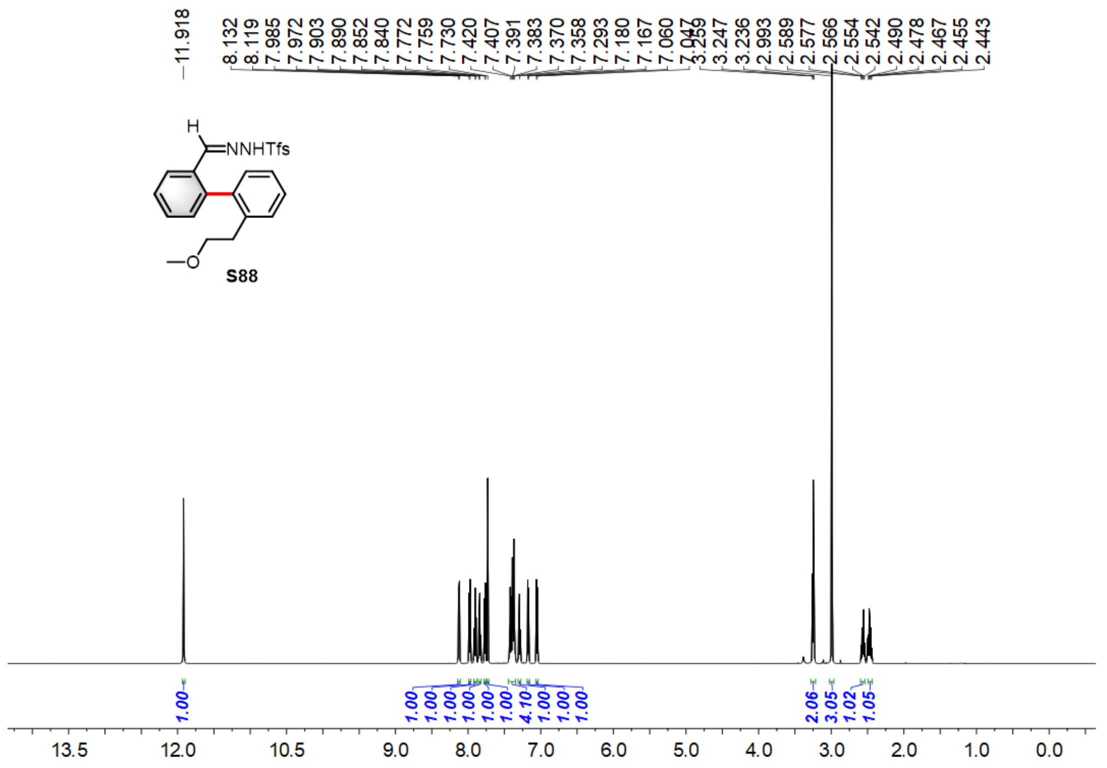

Supplementary Fig. 292 ^{1}H NMR (600 MHz, DMSO) spectrum of compound **S88**

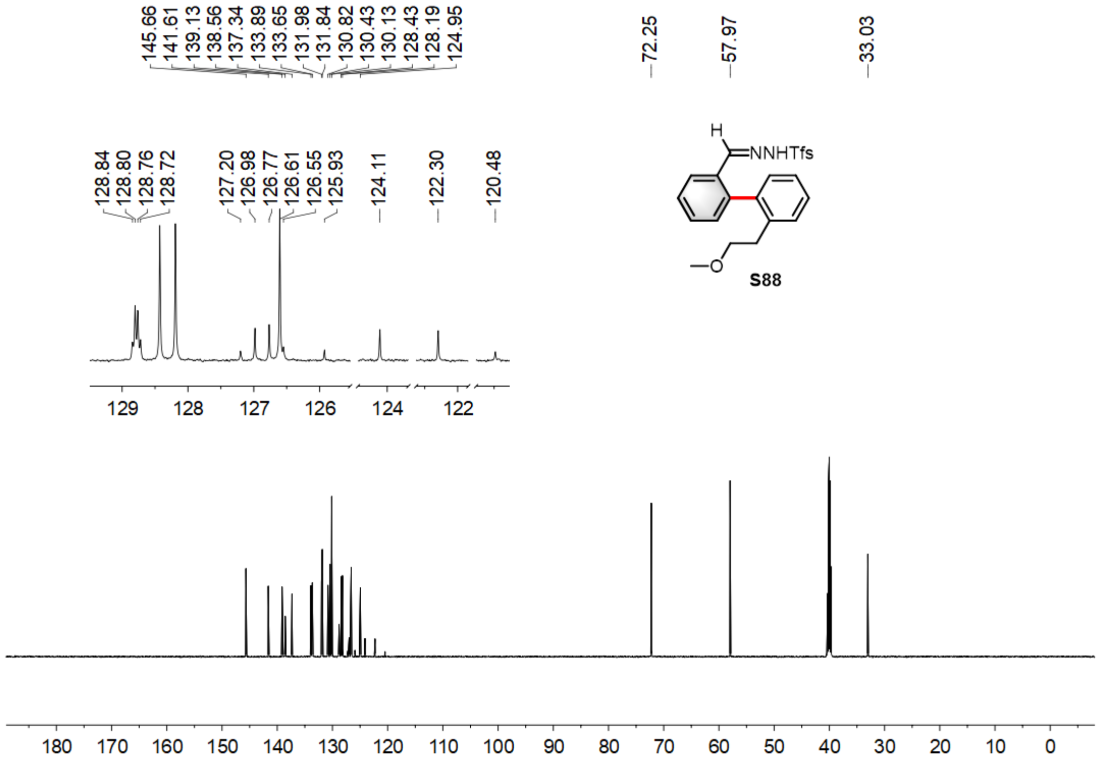

Supplementary Fig. 293 ^{13}**C NMR** (151 MHz, DMSO) spectrum of compound **S88**

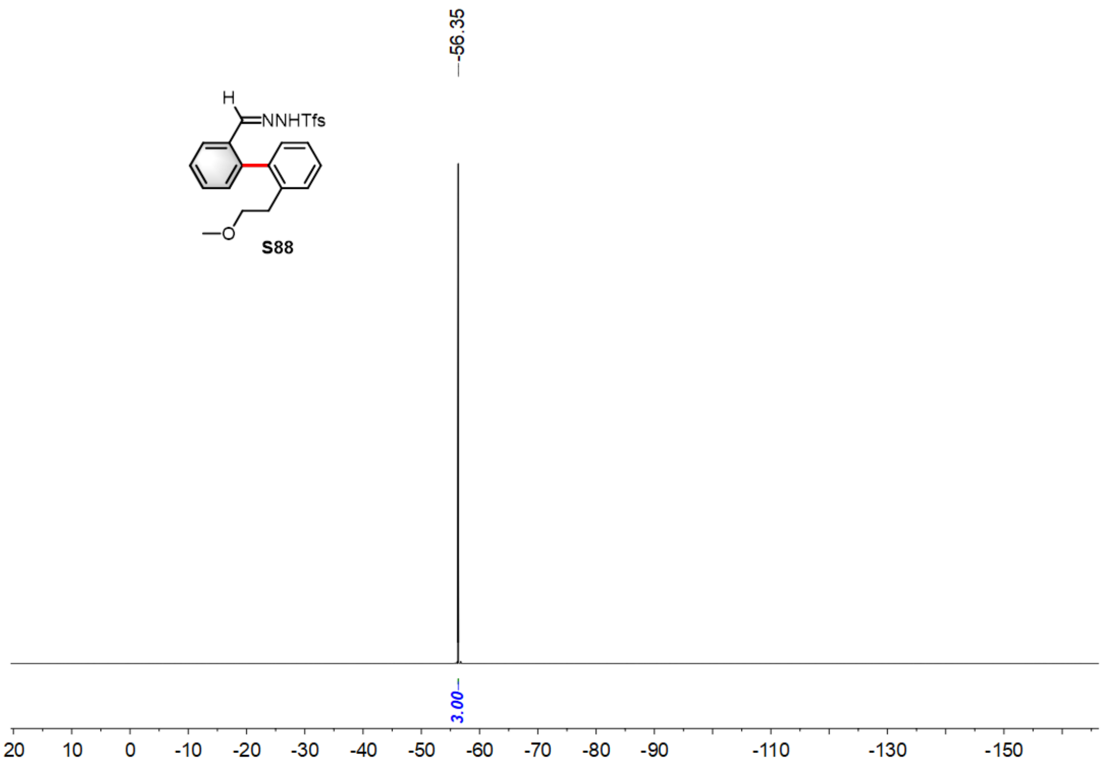

Supplementary Fig. 294 ^{19}**F NMR** (471 MHz, DMSO) spectrum of compound **S88**

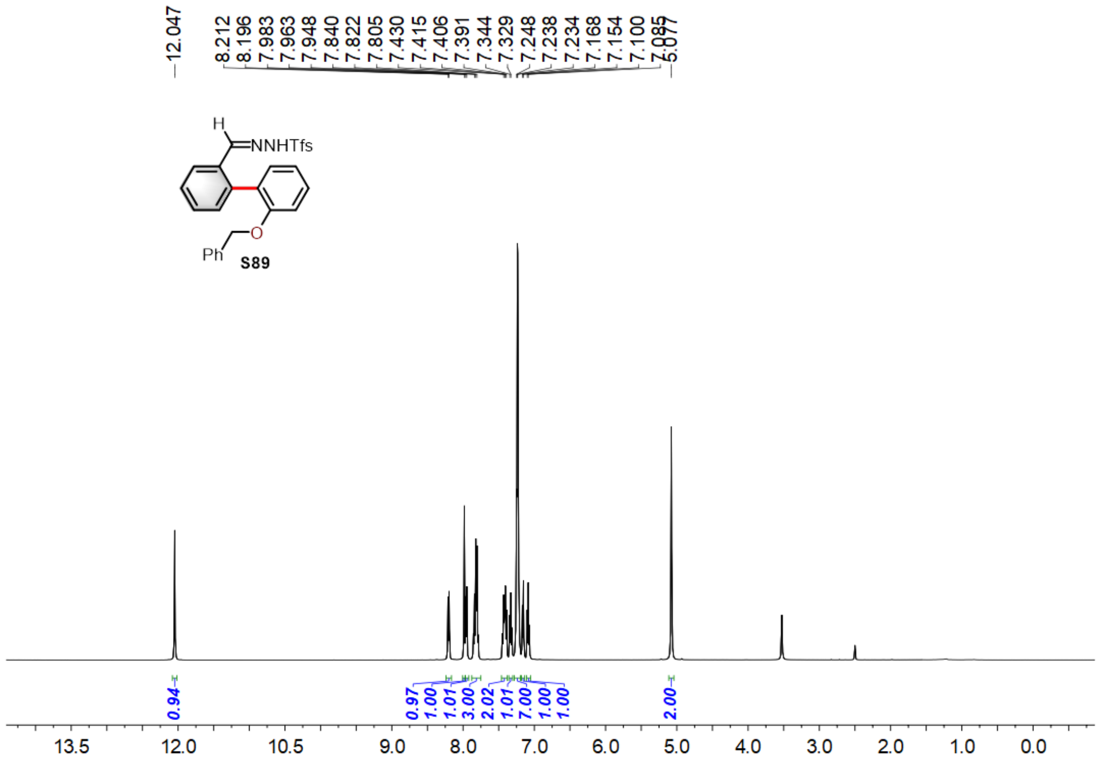

Supplementary Fig. 295 ^{1}**H NMR** (500 MHz, DMSO) spectrum of compound **S89**

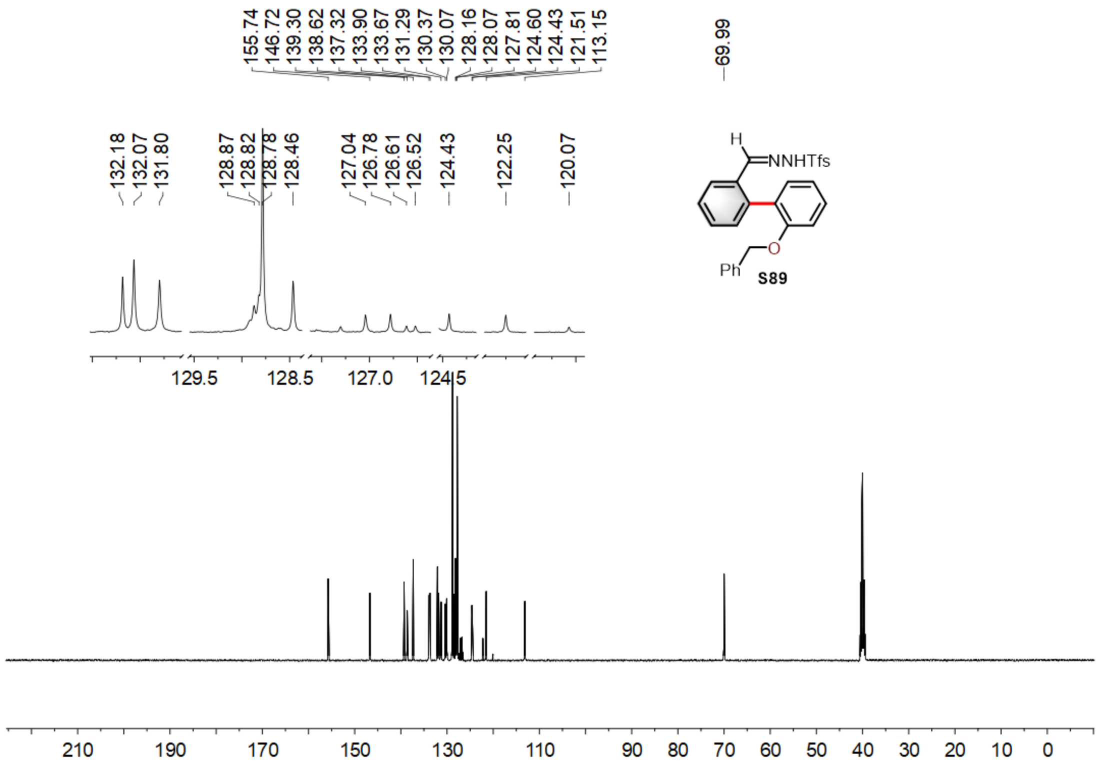

Supplementary Fig. 296 ^{13}**C NMR** (126 MHz, DMSO) spectrum of compound **S89**

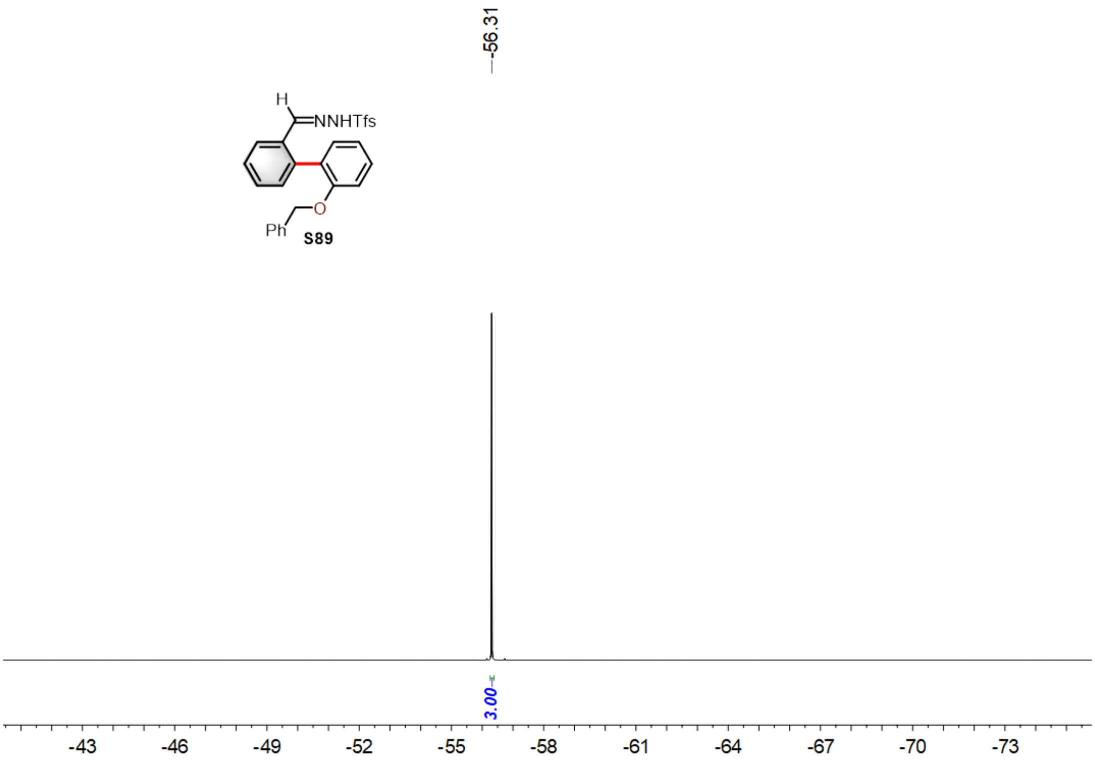

Supplementary Fig. 297 **^{19}F NMR** (471 MHz, DMSO) spectrum of compound **S89**

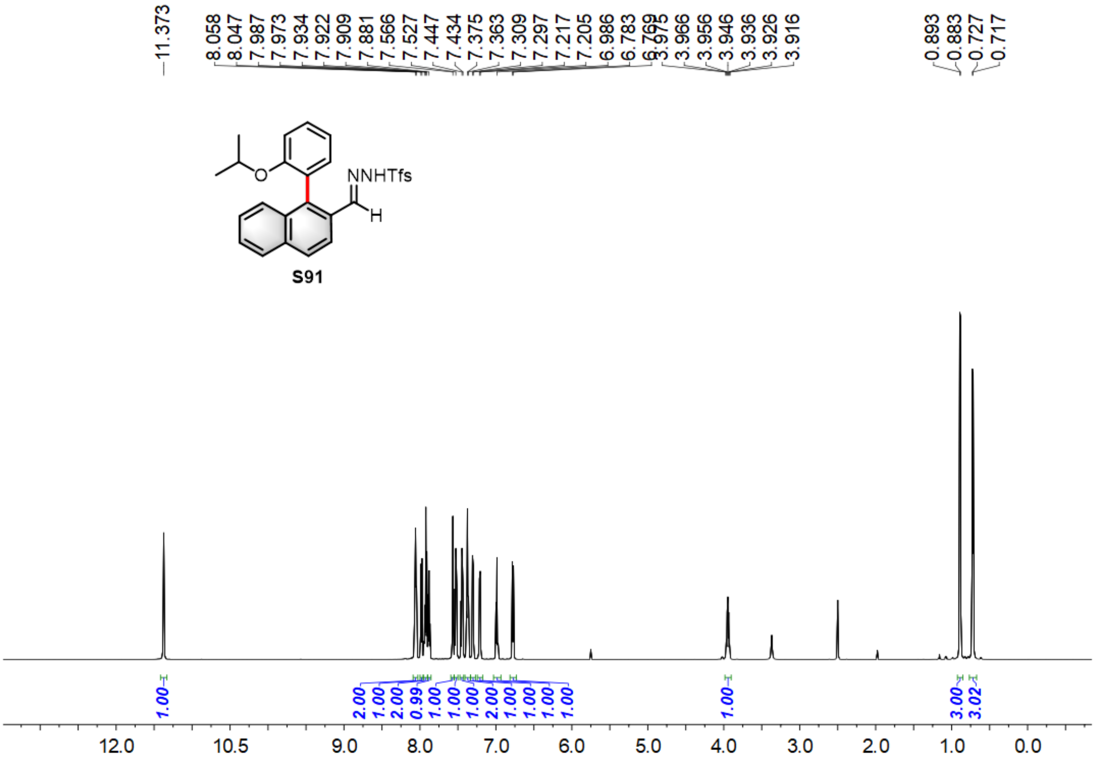

Supplementary Fig. 298 **^{1}H NMR** (600 MHz, DMSO) spectrum of compound **S91**

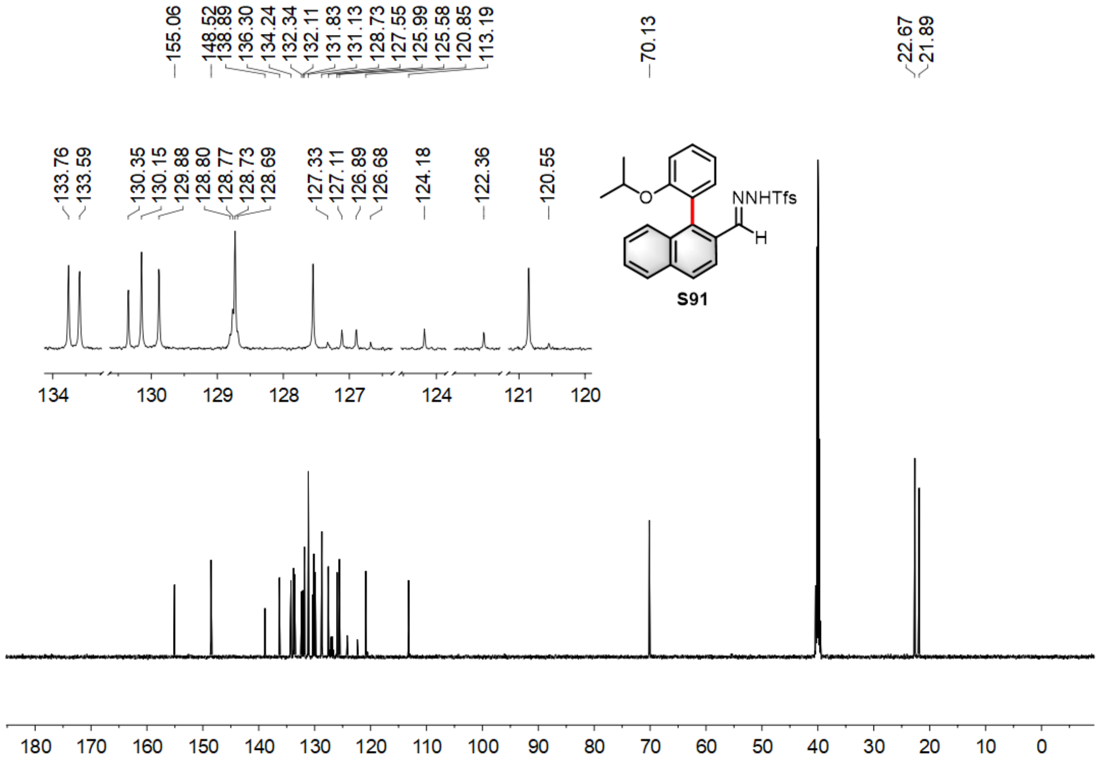

Supplementary Fig. 299 ^{13}**C NMR** (151 MHz, DMSO) spectrum of compound **S91**

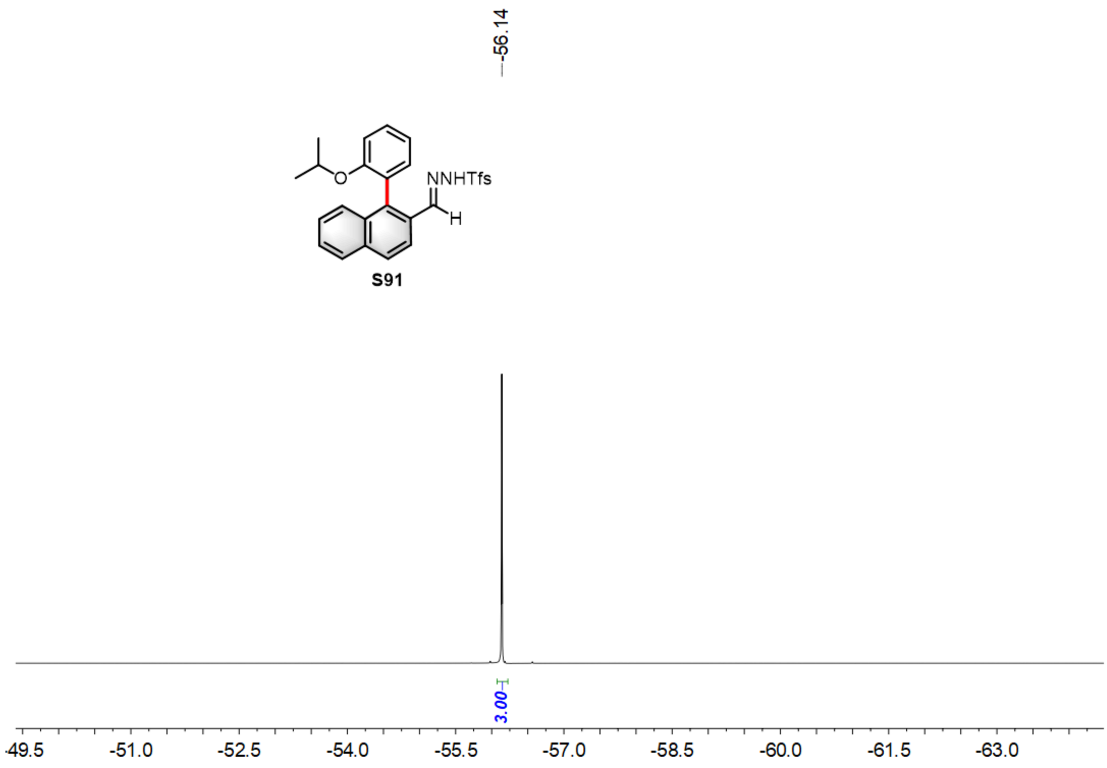

Supplementary Fig. 300 ^{19}**F NMR** (471 MHz, DMSO) spectrum of compound **S91**

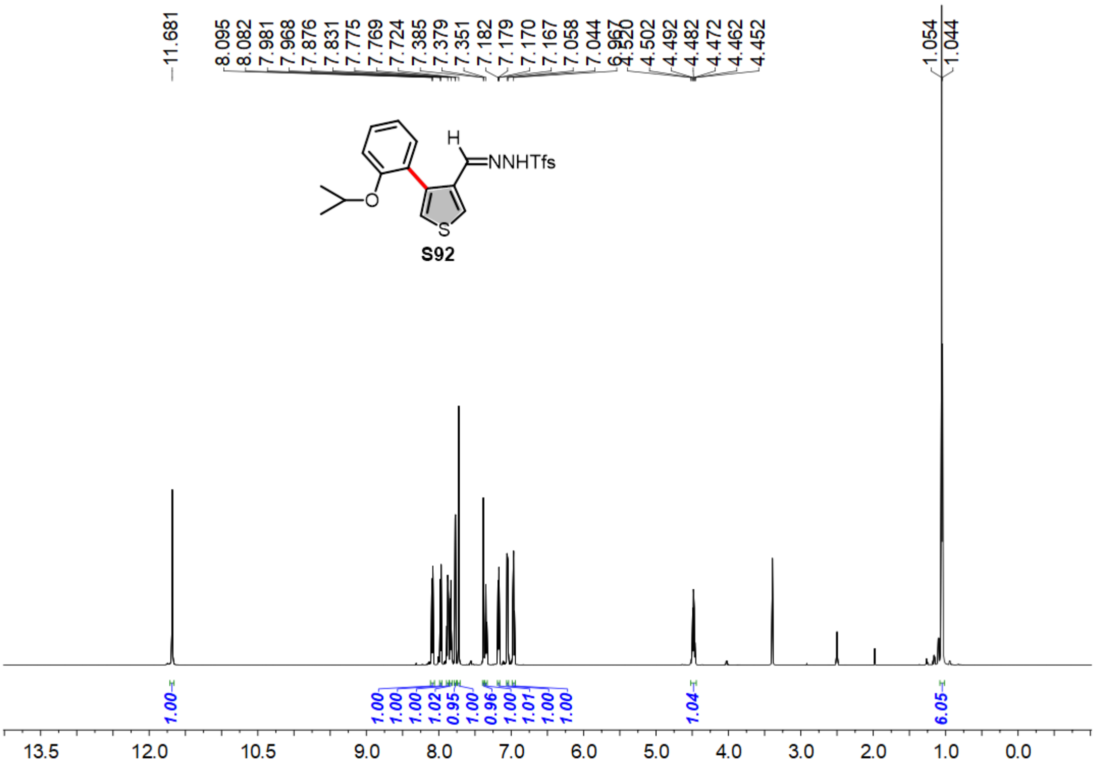

Supplementary Fig. 301 ^{1}H NMR (600 MHz, DMSO) spectrum of compound **S92**

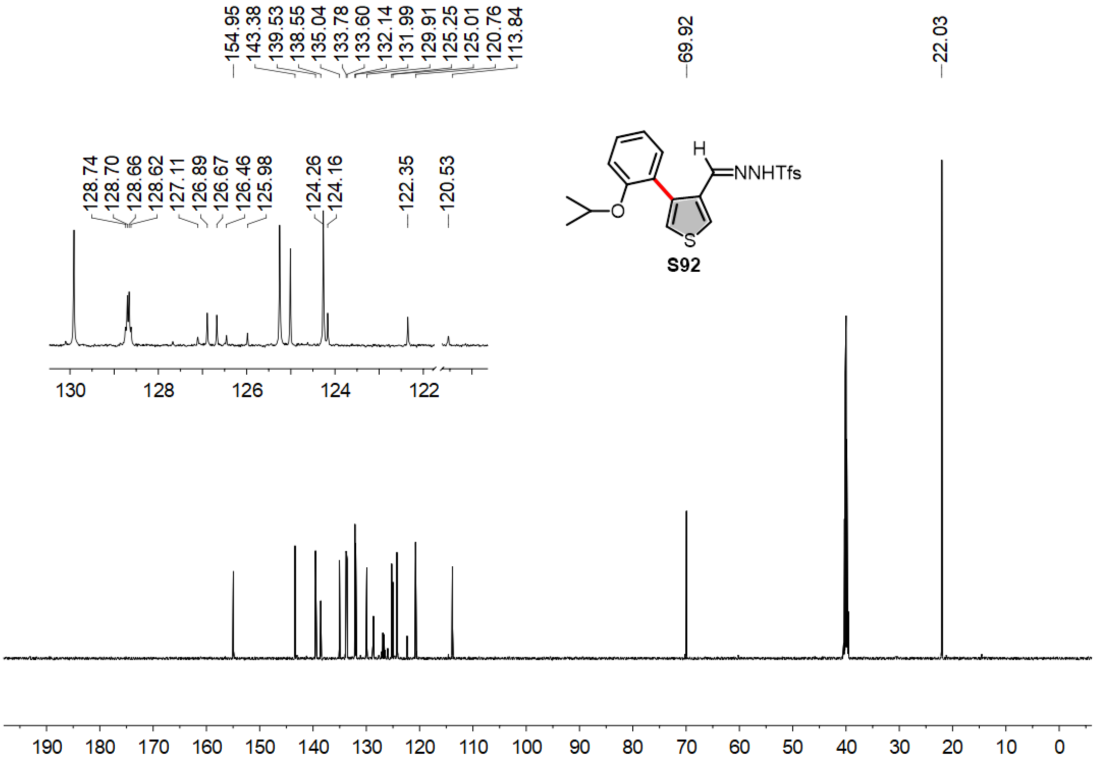

Supplementary Fig. 302 ^{13}C NMR (151 MHz, DMSO) spectrum of compound **S92**

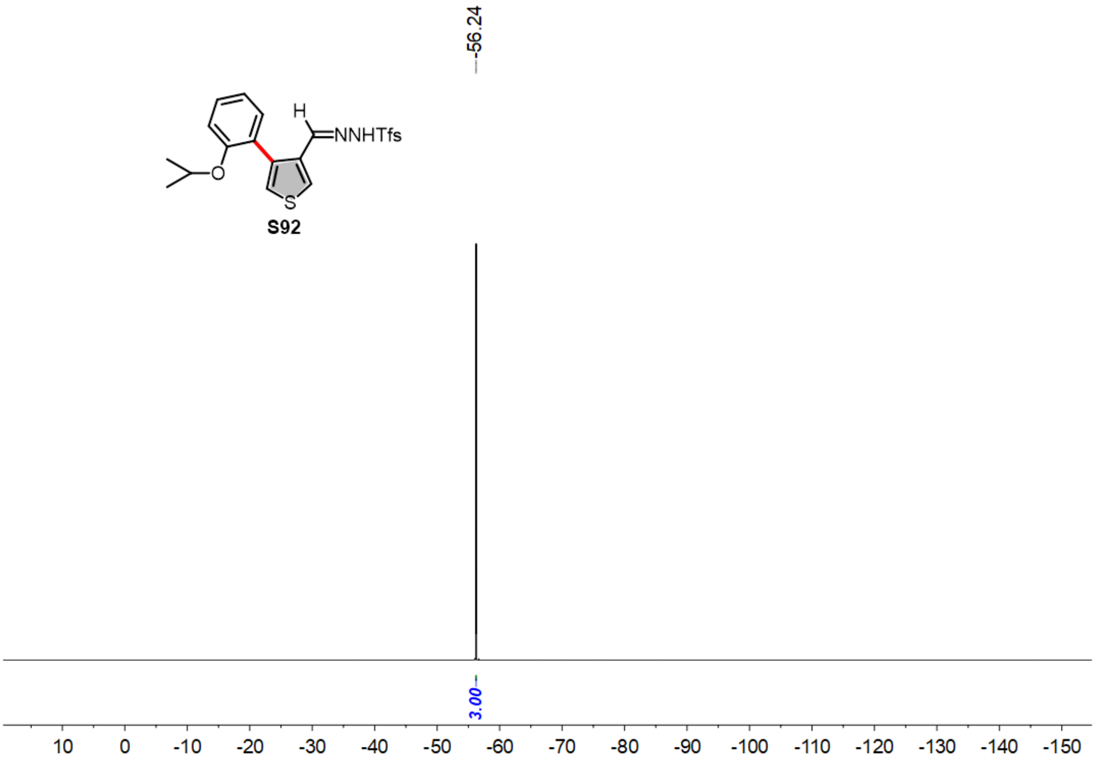

Supplementary Fig. 303 ^{19}F NMR (471 MHz, DMSO) spectrum of compound **S92**

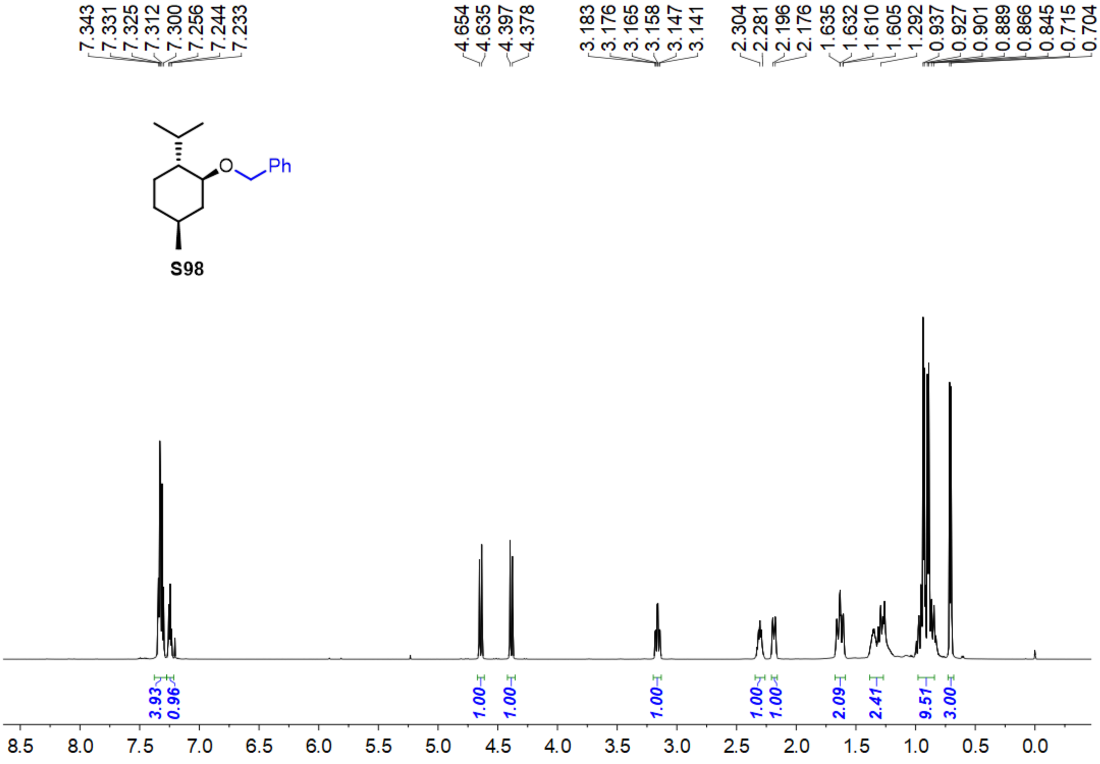

Supplementary Fig. 304 ^{1}H NMR (600 MHz, $CDCl_3$) spectrum of compound **S98**

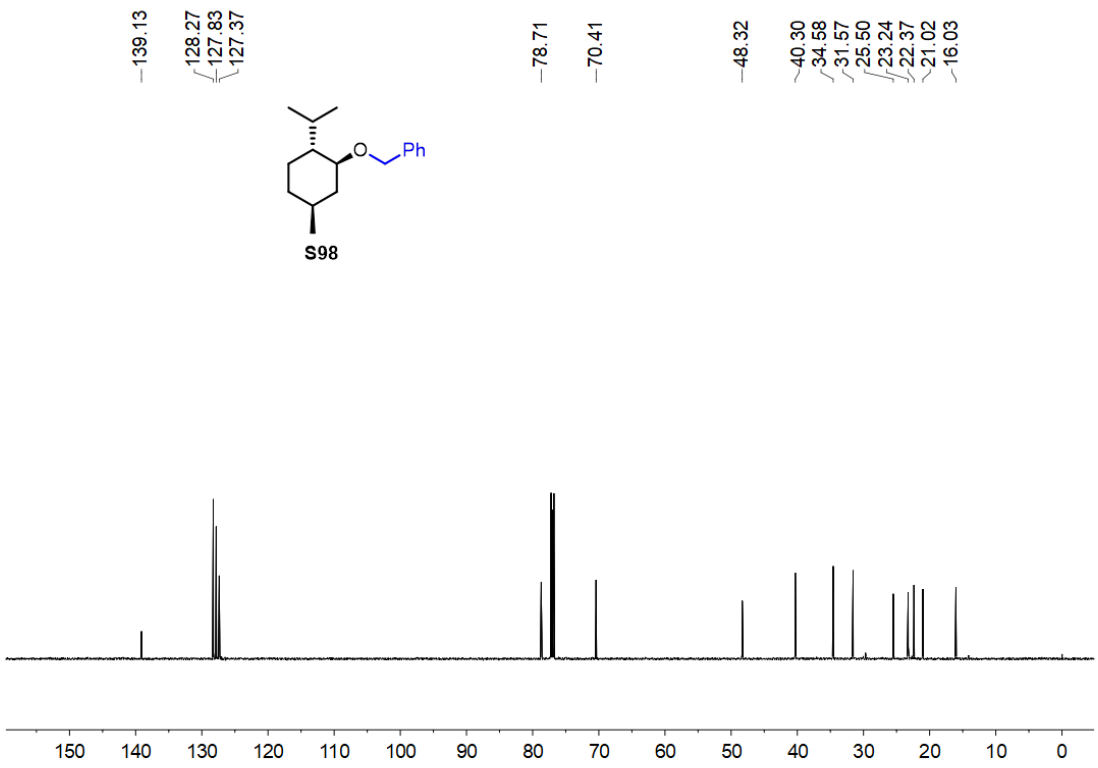

Supplementary Fig. 305 ^{13}C NMR (151 MHz, $CDCl_3$) spectrum of compound **S98**

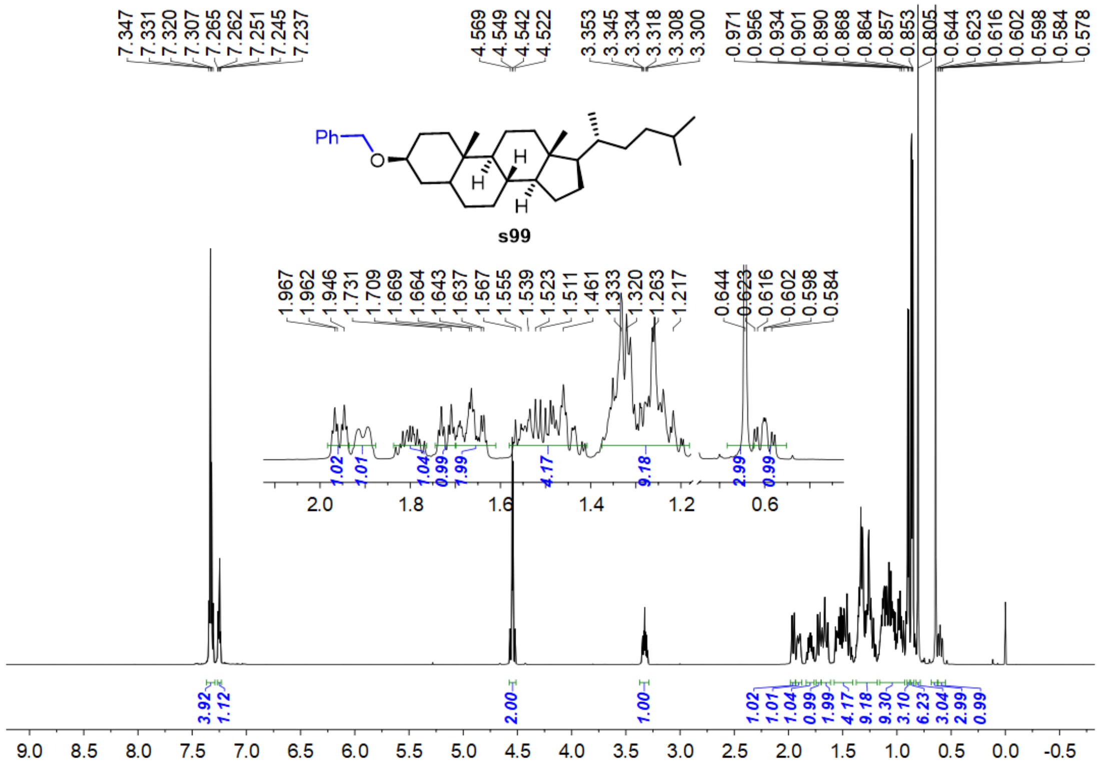

Supplementary Fig. 306 ^{1}H NMR (600 MHz, $CDCl_3$) spectrum of compound

S99

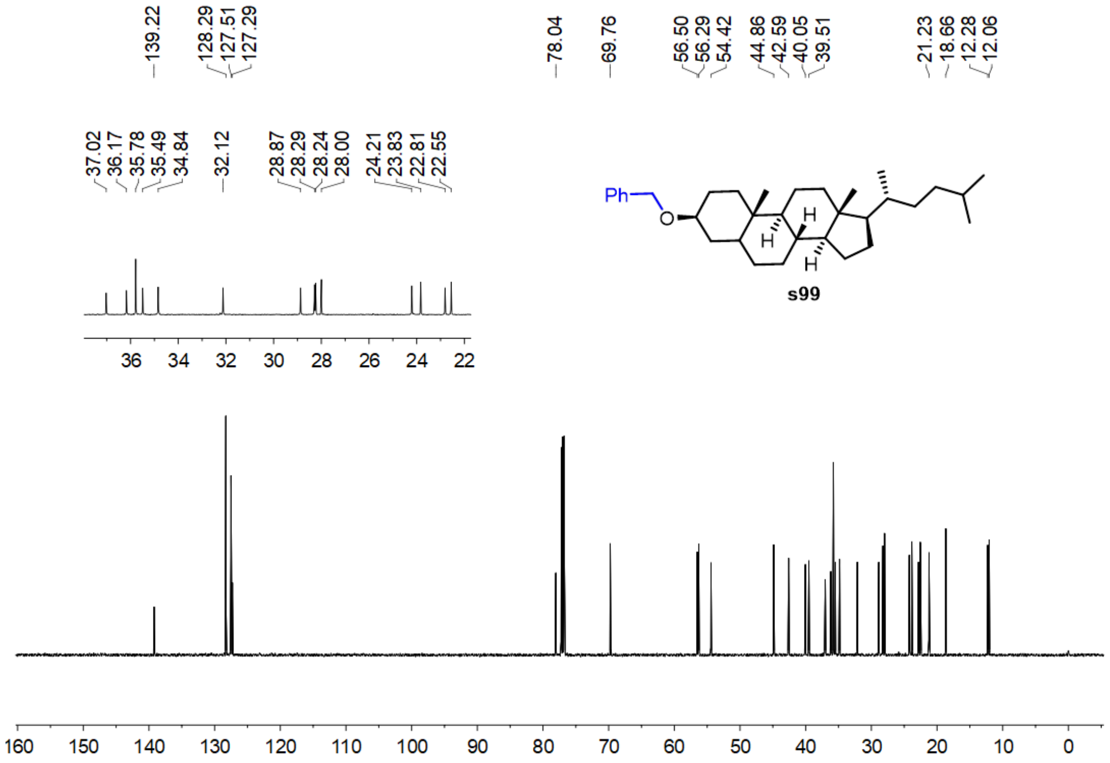

Supplementary Fig. 307 ^{13}**C NMR** (151 MHz, $CDCl_3$) spectrum of compound **S99**

II. Supplementary References

1. Urbano, J. *et al*. Functionalization of primary carbon-hydrogen bonds of alkanes by carbene insertion with a silver-based catalyst. *Organometallics*, **24**, 1528–1532 (2005).
2. Caballero, A. *et al.* Highly regioselective functionalization of aliphatic carbon-hydrogen bonds with a perbromohomoscorpionate copper(I) aatalyst. *J. Am. Chem. Soc.* **125**, 1446–1447 (2003).
3. Goh, S. H., Closs, L. E. & Closs, G. L. Carbenoid decomposition of aryldiazomethanes with lithium and zinc halides. Synthesis of arylcyclopropanes. *J. Org. Chem.* **34**, 25–31(1969).
4. González-Bobes F. & Fu, G. C. Amino alcohols as ligands for nickel-catalyzed Suzuki reactions of unactivated alkyl halides, including secondary alkyl chlorides, with arylboronic acids. *J. Am. Chem. Soc.* **128**, 5360–5361 (2006).
5. Chakraborty, N. *et al.* Combination of $NH_2OH·HCl$ and $NaIO_4$: an effective reagent for olecular iodine-free regioselective 1,2-difunctionalization of olefins and easy access of terminal acetals. *RSC Adv.* **5**, 56780–56788 (2015).
6. Asai, S., Kato, M., Monguchi, Y, Sajiki, H. & Sawama, Y. Cyclic ether synthesis from diols using trimethyl phosphate. *Chem. Commun.* **53**, 4787–4790 (2017).
7. Gonzalez-de-Castro, A., Robertson, C. M., & Xiao, J. Dehydrogenative α-oxygenation of ethers with an iron catalyst. *J. Am. Chem. Soc.* **136**, 8350−8360 (2014).
8. Azzena, U., Demartis, S. & Melloni G. Electron-transfer-induced reductive cleavage of phthalans: reactivity and synthetic applications. *J. Org. Chem.* **61**, 4913–4919 (1996).

9. Catti, L. & Tiefenbacher, K. Intramolecular hydroalkoxylation catalyzed inside a self-assembled cavity of an enzyme-like host structure. *Chem. Commun.* **51**, 892–894 (2015).

10. Kirmse, W. & Kund, K. Intramolecular generation of oxonium ylides from functionalized arylcarbenes. *J. Am. Chem. Soc.* **111**, 1465–1473 (1989).

11. Zheng, H., Shan, X., Qu, J. & Kang, Y. Strategy for overcoming full reversibility of intermolecular radical addition to aldehydes: tandem C−H and C−O bonds cleaving cyclization of (phenoxymethyl)arenes with carbonyls to benzofurans. *Org. Lett.* **20**, 3310−3313 (2018).

12. Zhu, Y. *et al.* Silver-catalyzed remote Csp^3-H functionalization of aliphatic alcohols. *Nat. Commun.* **9**, 2625 (2018).

13. Chen. Z. *et al.* Complex bioactive alkaloid-type polycycles through efficient catalytic asymmetric multicomponent aza-Diels–Alder reaction of indoles with oxetane as directing group. *Angew. Chem. Int. Ed.* **52**, 2027–2031 (2013).

14. Yao, Q., Zhang, S., Zhan, B. & Shi, B. Atroposelective synthesis of axially chiral biaryls by palladium-catalyzed asymmetric C−H olefination enabled by a transient chiral auxiliary. *Angew.Chem. Int. Ed.* **56**, 6617–6621(2017).

15. Liu, Z. *et al.* Site-selective C–H benzylation of alkanes with *N*-triftosylhydrazones leading to alkyl aromatics. *Chem* **6**, 2110–2114 (2020).

16. Frisch, M. J. *et al.* Gaussian 09, Revision D.01. Gaussian, Inc., Wallingford CT, USA, 2009.

17. Zhao, Y. & Truhlar, D. G. The M06 suite of density functionals for main group thermochemistry, thermochemical kinetics, noncovalent interactions, excited states, and transition elements: two new functionals and systematic testing of four M06-class functionals and 12 other functionals. *Theor. Chem. Acc*. **120**, 215-241 (2008).

18. Dolg, M., Wedig, U., Stoll, H. & Preuss, H. Energy-adjusted abinitio pseudopotentials for the first row transition elements. *J. Chem. Phys*. **86**, 866-872 (1987).

19. Dolg, M., Wedig, U., Stoll, H. & Preuss, H. Energy-adjusted abinitio pseudopotentials for the first row transition elements. *J. Chem. Phys*. **86**, 866-872 (1987).

20. Marenich, A. V., Cramer, C. J. & Truhlar, D. G. Universal solvation model based on solute electron density and on a continuum model of the solvent defined by the bulk dielectric constant and atomic surface tensions. *J. Phys. Chem. B* **113**, 6378-6396 (2009).

21. Fukui, K. Formulation of the reaction coordinate. *J. Phys. Chem*. **74**, 4161-4163 (1970).

22. Fukui, K. The path of chemical reactions-the IRC approach. *Acc. Chem. Res*. **14**, 363-368 (1981).

23. Reed, A. E., Weinstock, R. B. & Weinhold, F. Natural population analysis. *J. Chem. Phys.* **83**, 735-746 (1985).

24. Legault, C. Y. CYLview, 1.0b; Université de Sherbrooke: Canada, 2009. Available at: http://www.cylview.org (accessed September, 2014).
